# Disentangling the Puzzling Regiochemistry of Thiol Addition to *o*-Quinones

**DOI:** 10.1021/acs.joc.1c02911

**Published:** 2022-03-10

**Authors:** Maria L. Alfieri, Alice Cariola, Lucia Panzella, Alessandra Napolitano, Marco d’Ischia, Luca Valgimigli, Orlando Crescenzi

**Affiliations:** † Department of Chemical Sciences, University of Naples Federico II, Via Cintia 21, Naples I-80126, Italy; ‡ Department of Chemistry “Giacomo Ciamician”, University of Bologna, Via S. Giacomo 11, Bologna I-40126, Italy

## Abstract

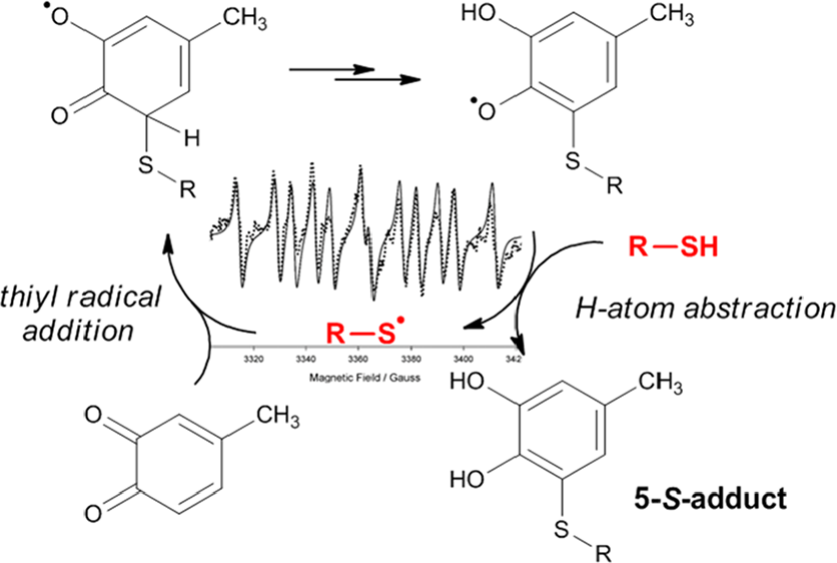

The addition of thiol compounds to *o*-quinones, as exemplified by the biologically relevant conjugation of cysteine to dopaquinone, displays an anomalous 1,6-type regiochemistry compared to the usual 1,4-nucleophilic addition, for example, by amines, which has so far eluded intensive investigations. By means of an integrated experimental and computational approach, herein, we provide evidence that the addition of glutathione, cysteine, or benzenethiol to 4-methyl-*o*-benzoquinone, modeling dopaquinone, proceeds by a free radical chain mechanism triggered by the addition of thiyl radicals to the *o*-quinone. In support of this conclusion, DFT calculations consistently predicted the correct regiochemistry only for the proposed thiyl radical-quinone addition pathway. These results would prompt a revision of the commonly accepted mechanisms for thiol-*o*-quinone conjugation and stimulate further work aimed at assessing the impact of the free radical processes in biologically relevant thiol–quinone interactions.

## 1 Introduction

**Scheme 1:**
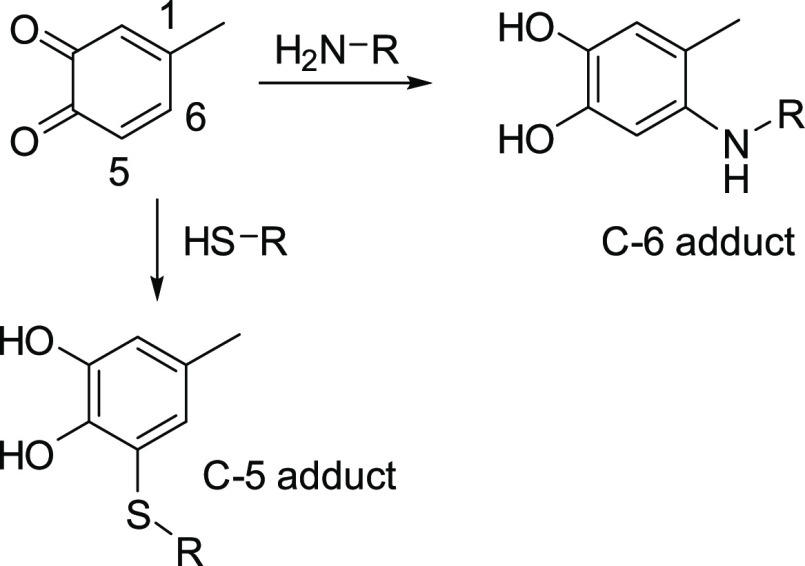
Oxidative Coupling Reactions of Catechols with Thiol or Amine Compounds Leading to C-5 or C-6 Conjugates, Respectively

The reactions of *o*-quinones with natural thiol compounds such as cysteine or glutathione^1^ are of central relevance to a variety of biological processes, including the synthesis of melanin pigments and of firefly luciferin,^2−7^ the metabolic transformation of xenobiotics,^8−10^ and the cross-linking mechanisms in sclerotized insect cuticles and byssal threads.^1,11−13^ It is implicated moreover in food, agricultural, and environmental chemistry,^14,15^ materials science,^16−20^ toxicology,^8,21,22^ and organic synthesis (e.g., for benzothiazine and related heterocyclic systems).^23,24^ A distinguishing, yet puzzling, feature of the thiol-*o*-quinone reaction is the anomalous regiochemistry of the coupling reaction leading mainly to C-5-linked adducts. This regiochemistry is in marked contrast with that of all other nucleophile addition pathways, which follow the usual path to C-6 conjugates (Scheme 1).

In this regard, a note on terminology is in order. While a rigorous nomenclature would assign positions 1 and 2 to the oxygen-bearing carbons of the catechol/quinone ring, it has become customary in the field of catecholamines, typical substrates for this chemistry, to refer to the chain-bearing carbon as the 1-position (hence the name 3,4-dihydroxyphenylalanine for DOPA). It follows that the major addition product of cysteine to DOPA is the 5-*S*-adduct, while attack to the alternate *ortho* site would lead to the 2-*S*-adduct.

This anomalous regiochemistry is exemplified by the conjugation of DOPA or catecholamines with cysteine or glutathione (GSH), leading mainly to 5-*S*-adducts and minor amounts of the 2-*S*- and the 2,5-*S*,*S*-diadducts.^25−28^ Replacement of the alkyl chain of catecholamines with electron-withdrawing substituents directs the attack of the thiol toward the more hindered 2-position of the *o*-quinone ring,^29−32^ but in no case is the typical regiochemistry via a 1,4-nucleophilic addition observed.

**Scheme 2:**
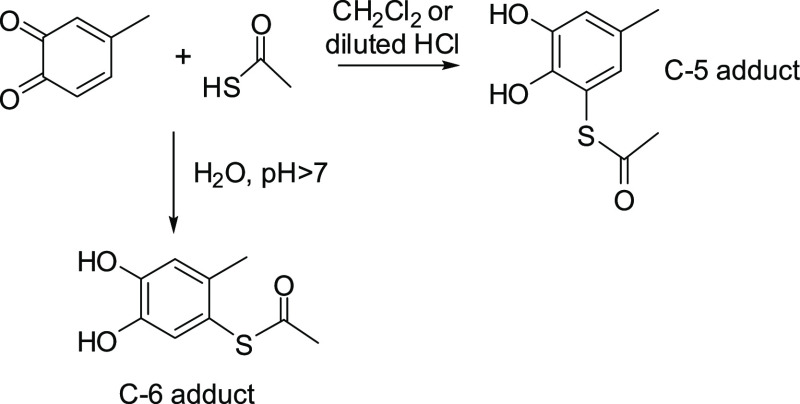
Schematic Reaction of 4-Methyl-*o*-benzoquinone with Thioacetic Acid under Different Reaction Conditions

The structural and mechanistic underpinnings of the unusual regiochemistry of this reaction have remained largely speculative. Early studies on the reaction of 4-methyl-*o*-benzoquinone (4-MBQ) with thioacetic acid (p*K*_a_ 3.3) showed that in an aqueous or alkaline medium, the “normal” 6-*S*-adduct prevailed, whereas the “anomalous” 5-*S*-adduct dominated at acidic pH or in the organic solvent (Scheme 2).^33^ This divergent behavior was attributed to the differential reactivity of the thiolate anion versus the thiol, the latter being less reactive and expected to react more selectively at the less sterically hindered position, benefitting also from greater resonance stabilization.

Interestingly, this regiochemistry seems apparently to be related to the typical reactivity of the thiol group since other sulfur nucleophiles, such as thioureas and derivatives, usually add to the “canonical” C-6 position.^34,35^

A detailed investigation of the reaction of *N*-acetylcysteine with 4-MBQ revealed that mixing preformed quinone with the thiol in the absence of oxygen does not result in any detectable addition product. However, significant product formation was observed when the parent catechol and the thiol were allowed to oxidize with ferricyanide in the presence of oxygen. It was concluded that addition of *N*-acetylcysteine to 4-methylcatechol in the presence of basic ferricyanide requires oxygen and does not proceed via nucleophilic addition of sulfur to the quinone but involves rather a free radical pathway, probably involving the nucleophilic attack of sulfur on 4-methyl-*o*-semiquinone.^36^ The possible importance of the energy levels for the lowest unoccupied molecular orbital of the quinone rather than resonance arguments in guiding mechanistic conclusions was also emphasized.^37^

In other studies, electron paramagnetic resonance (EPR) evidence indicated the generation of semiquinone radicals by reaction of a number of quinones, including *p*-benzoquinone and 1,4-naphthoquinone, with GSH.^38,39^ In most cases, the reactions of quinones with thiols proved to be complex, and not all the features were elucidated.^40^ The generation of thiyl-radicals, for example, from GSH, by redox exchange with semiquinone free radicals was also the subject of a detailed investigation.^41^

The appearance of GSSG by reactions of GSH and its radicals with benzoquinones was attributed to oxidation of the hydroquinone by oxygen and the resulting superoxide or H_2_O_2_ promoting the oxidation of GSH to GSSG.^42^

Pulse radiolysis studies aimed at elucidating the mechanisms of the reaction of various radicals with benzoquinone, and its methyl-substituted derivatives in non-polar media showed the competition of two mechanisms, free radical addition onto the quinones resulting in substituted semiquinone radicals and electron transfer reduction producing semiquinone radical anions.^43^

Kinetic analysis of the reactions of 4-MBQ with proteins, thiol, and amine compounds under pseudo-first-order conditions supported fast rates that were attributed, in the case of cysteine, to coordination of the amine group to the quinone oxygen directing addition of the thiol group onto the adjacent position.^44^ This conclusion was in line with an earlier kinetic study^45^ reporting the reversible generation of the postulated tetrahedral intermediate at C-5 in the attack of cysteine or mercaptoacetic acid to dopamine quinone. In that study, however, the origin of the observed regiochemistry was not addressed in detail.

Recently, a DFT investigation of the reaction of l-cysteine thiolate with dopaquinone indicated that the 2-*S*-linked intermediate was less stable than the 6-*S*-bonded isomer and that the most favorable addition pathway is at the two carbonyls (C3–C4 bridge site). The main conclusion was that the initial adduct evolves via migration of the thiolate to the adjacent 5- or 2-position through energetically viable reaction channels.^46^ In the same study, an alternate free radical-mediated addition mechanism was also considered, in which the reducing properties of thiols were suggested to account for the generation of semiquinone and thiyl radicals, which were amenable to recombination. However, the hypothesis was later abandoned because of the unfavorable energetic profile of the coupling process.

A clue supporting a possible role of free radical coupling processes was provided by the reaction of 1,4-benzoquinone diimine with thiophenol or decanethiol in chlorobenzene, which was suggested to proceed via a radical chain mechanism in which the addition of thiyl radicals was envisaged as the rate-determining step.^47^ Further studies of this reaction suggested roles of thiols both as reactants and as catalysts in the rate-determining propagation step.^48^ The chemical interaction between polyphenols and thiols was also investigated under free radical oxidation conditions using a stoichiometric amount of the 2,2-diphenyl-1-picrylhydrazyl (DPPH) radical. In this case, adduct formation was compatible with two possible mechanisms, namely, generation and coupling of semiquinone and thiyl radicals or disproportionation of semiquinone radicals to *o*-quinone followed by nucleophilic addition.^49^

Overall, the results of these and other studies have provided little more than circumstantial evidence in favor of homolytic mechanisms, whereby the thiol-*o*-quinone coupling issue has remained virtually unsettled under the loose definition of “anomalous nucleophilic addition”.^1^

In the frame of our continuing studies on the thiol–quinone coupling chemistry,^50,51^ we report herein a re-examination of the reaction of cysteine with 4-MBQ with a view to elucidating the key factors underlying the anomalous regiochemistry of coupling. Chemical experiments coupled with extensive DFT calculations prompted a revision of currently held models in favor of an unusual free radical addition mechanism propagating a chain process.

## 2 Results and Discussion

### 2.1 Rationale and Experiment Design

**Figure 1:**
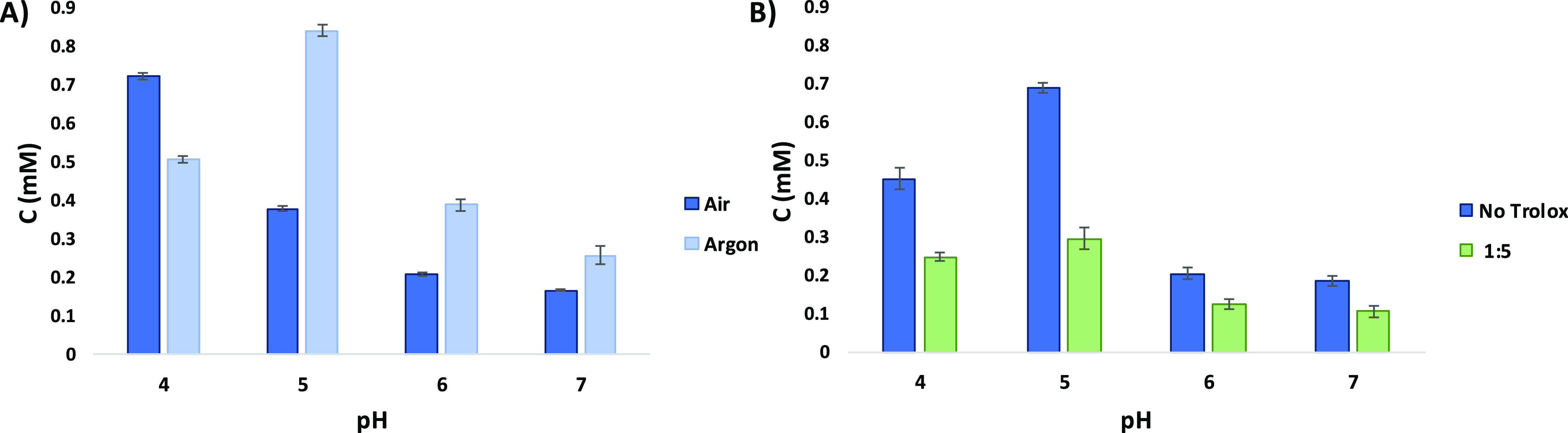
(A) Yields of the 5-*S*-cysteinyl adduct of the reaction of cysteine with 4-MBQ (10:1 M ratio) as a function of pH in the presence and in the absence of air. (B) Effect of Trolox on the yields of the 5-*S*-cysteinyl adduct at a 1:5 catechol-cysteine/Trolox ratio as a function of pH in the absence of air.

The reaction between 4-MBQ and cysteine was initially selected as a probe system because of its analogy with the biologically relevant conjugation of cysteine with dopaquinone and the availability of a solid set of literature data on this latter reaction.^27,36^ A 10-fold excess of the thiol was used to ensure a high product yield, and the reaction conditions were determined by proper selection of pH and using an air or an argon atmosphere when required. HPLC analysis of the reaction product showed a main component that was identified as the 5-*S*-isomer by comparison with an authentic sample^52^ along with minor species that were identified as the 2-*S* and 6-*S* isomers (Figure S1). This latter formed in very low amounts was identified by comparison with a standard prepared by heating 4-methylcatechol with cystine in aq. HBr followed by ion exchange chromatography purification as previously reported.^53^ Some amounts of 4-methylcatechol were also observed together with another species in very low amounts identified as a diadduct (LC–MS evidence). The quantitation data of conjugation products are shown in Figure 1 and S2 and were obtained by halting the reaction with sodium dithionite to prevent product decomposition.

Data shown in Figure 1 allowed us to draw important conclusions of mechanistic relevance.

First, in all the cases examined, the 5-*S*-isomer was by far the dominant product, in line with literature data, with much lower amounts of the 2-*S* and the 6-*S* isomers. The trace amounts of catechol observed evidently derive from reduction of the quinone during the reaction. Mass balance analysis indicated yields of the 5-*S*-isomer with respect to the *o*-quinone reaching 84% at pH 5.0 under argon, while the overall recovery of the three adducts reached 94% under these conditions.

Second, formation yields of the 5-*S* gradually decreased with increasing pH in air. A similar trend is also observed for the minor isomers. At pH 7.0, small amounts of the diadduct could be determined, which however did not account for the overall drop in yield even when 4-methylcatechol formation was considered. A possible explanation for the observed trend in air was based on consideration of the increasing instability of adducts to autoxidation with increasing pH. Consistent with this conclusion, much higher adduct yields were measured when the reaction was carried out in an argon atmosphere.

Third, and quite unexpectedly, adduct yields at pH 4.0 were found to be higher in air than under argon, these latter peaking at pH 5.0 and dropping down dramatically at pH 7.0 in all cases.

Overall, these data could be explained by assuming that

i air plays a positive role in promoting the reaction at moderately acidic pH, that is, pH 4.0, under conditions where autoxidation is not significant;
ii at a pH of around 5, the overall adduct yield increases in the oxygen-depleted medium with no significant effect on the regiochemistry, suggesting a significant product autoxidation in air;
iii at pH 7.0, the adduct yield intrinsically decreases due at least in part to both quinone reduction to catechol and diadduct formation.

In view of the extensive product decomposition in air, the air-depleted conditions were taken as a reference for further mechanistic discussions, although the promoting effect of oxygen on the reaction was considered.

The relationship between the bell-shaped curve with pH under argon and the nucleophilic addition mechanism was not obvious since this latter would be favored by an increase in pH following thiol conversion to thiolate, and a more complex air-dependent mechanism was indicated. In this connection, the small but detectable rise in catechol formation with pH could be due to competing side processes. It is possible that at pH 7.0, but not at pH 5.0 or 6.0, the unreacted quinone oxidizes the adducts, causing a decrease in their yields, but the reasons are unclear.

To gain more insight into the mechanism of the reaction, in further experiments, the effect of an established chain-breaking antioxidant, that is, Trolox, the water-soluble tocopherol analogue, on the course of the reaction was investigated under all the pH conditions examined. Indeed, in the case of the 5-*S*-adduct, the effect of the presence of Trolox was appreciable with a decrease in the formation of the 5-*S*-adduct of at least −40% using a 1:5 catechol-cysteine/Trolox ratio under all pH conditions and even more marked (up to −60%) at pH 5.0 corresponding to the maximal formation of the adduct (Figure 1). A similar effect was observed for the 2-*S* isomer while notably formation yields of the 6-*S*-isomer were not affected appreciably by the presence of Trolox (Figure S3).

Based on the above results, it was apparent that free radical species were involved to some extent in the coupling reaction. Accordingly, in another set of experiments, the generation and role of free radical species were investigated.

### 2.2 Investigations of Free Radical Intermediates

**Scheme 3:**
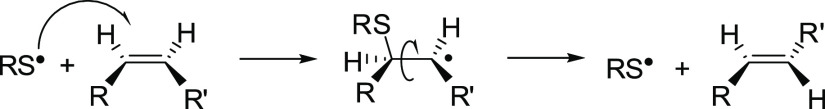
Thiyl Radical-Mediated Isomerization of Olefins

Experimental support to the intermediacy of thiyl radicals in the reaction was gained through investigation of *cis*–*trans* isomerization in olefins due to reversible addition to the C=C double bonds (Scheme 3), a reaction that was found to be key to the thiol-mediated isomerization of lipids in biomembranes, associated with oxidative stress related toxicity.^54^ Since this reaction is specific of thiyl radicals and not observed to any significative extent with other transient or persistent radicals (e.g., phenoxyl, aminyl, and nitroxyl),^55^ we endeavored to exploit this process to selectively detect the formation of thiyl radicals during the reaction of thiols with 4-MBQ. (*Z*)-Stilbene promptly reacts with thiyl radicals,^56^ and its *E*/*Z* isomers are easily distinguished by their UV spectra^57^ (Figure S4) or by GC–MS analysis (Figure S5); therefore, we used the *Z* → *E* isomerization of stilbene as a reporter of the formation of thiyl radicals.

When 1 mM 4-MBQ was incubated with 1–10 mM thiophenol (PhSH) as the nucleophile in the presence of 0.1 mM (*Z*)-stilbene in acetonitrile, formation of (*E*)-stilbene was observed by GC–MS analysis of the reaction mixture (Figures S6 and S7). Control experiments in the absence of 4-MBQ did not show significant isomerization (Figure S7), while experiments in which 4-MBQ was replaced by thermal radical initiator 2,2′-azobisisobutyronitrile (AIBN) showed marked *Z*→*E* isomerization detected using both GC–MS analysis and UV–vis spectroscopy (Figures S8 and S9).

Clearly, this observation supports the formation of thiyl radicals as transient intermediates in the reaction of 4-MBQ with PhSH. However, when the experiments were repeated, replacing thiophenol with mercaptoethanol (HSEtOH) or *N*-(*tert*-butoxycarbonyl)–(l)-cysteine methyl ester (LipCys), the lipophilic derivative of cysteine that models its reactivity,^58^ or thioacetic acid, no isomerization was observed (Figures S10–S12). This was attributed to the larger S–H bond dissociation enthalpy and shorter lifetime of aliphatic thiyl radicals, which reach a much lower steady-state concentration in the system. Indeed, no isomerization was detected even in control experiments using AIBN as the radical initiator in place of 4-MBQ (not shown), and only on using *tert*-butyl perbenzoate as the source of harsher initiating *t*BuO^•^ radicals could (*E*)-stilbene be detected in the reaction medium (Figure S10).

It was interesting to note that when using either PhSH or mercaptoethanol as the thiol reactant with 4-MBQ, GC–MS analysis of the reaction mixture revealed the formation of the corresponding disulfide and of a dimer of the quinone, along with unresolved thiol-quinone adducts, clearly suggesting the formation of transient radical species in the reaction mixture.

Based on these observations, the reaction of 4-MBQ with PhSH was carried out separately, and the main product was isolated and characterized as the 5-phenylthio adduct (5-methyl-3-(phenylthiol)benzene-1,2-diol) by complete spectral analysis (Figure S13–S17), confirming that the reaction with PhSH followed the same regiochemistry observed for l-cysteine.

On this basis, we then turned to spin-trapping experiments using EPR spectroscopy as a more sensitive method to detect the intermediacy of thiyl radicals.^59−61^ α-Phenyl *N*-tertiary-butyl nitrone (PBN)^62^ and 5-(diethoxyphosphoryl)-5-methyl-1-pyrroline-*N*-oxide (DEPMPO)^63^ are conventionally used as spin-traps for sulfur-centered radicals giving the species shown in Scheme S1, with a typical EPR spectrum. For this reason, by adding them to the reaction mixture of 4-MBQ and a thiol, they are supposed to form a nitroxyl radical adduct RS^•^/ST with a characteristic EPR signal, allowing to identify the trapped species.

**Figure 2:**
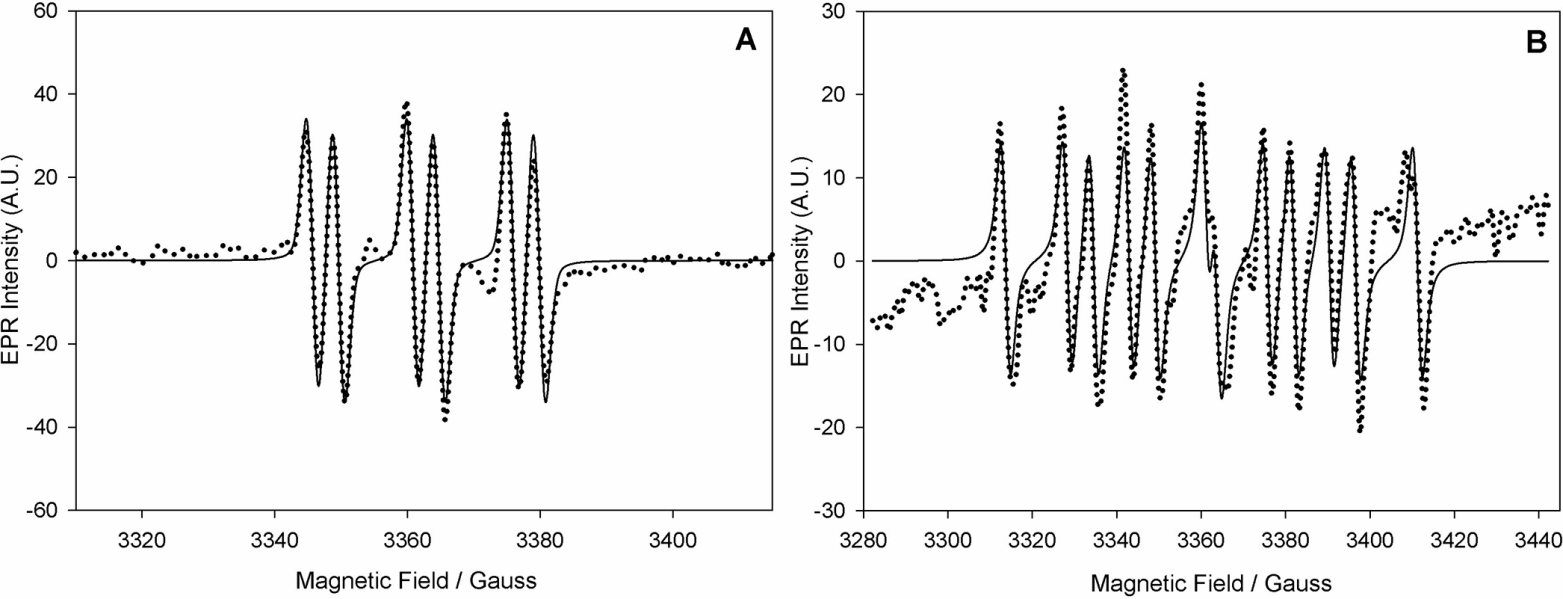
EPR spectra (dotted lines) recorded in spin-trapping experiments with (A) 30 mM PBN, 15 mM PhSH, 15 mM 4-MBQ in a 1:1 MeCN/acetate buffer (pH = 5.0) or (B) 30 mM DEPMPO, 15 mM PhSH, 15 mM 4-MBQ in a 1:1 MeCN/acetate buffer (pH = 5.0). Corresponding computer simulations (full lines) have been obtained with the following parameters: (A) *a*_N_ = 15.1 G, *a*_H_ = 3.9 G (*g* = 2.0067); (B) *a*_N_ = 14.6 G, *a*_H_ = 20.9 G, *a*_P_ = 47.4 G (*g* = 2.0064).

Experiments were initially carried using PhSH as the thiol, since it affords a more stable thiyl radical which is expected to reach higher steady-state concentrations, allowing for a more efficient trapping in solution. The reaction of PhSH with 4-MBQ was carried out in the cavity of the EPR spectrometer (in 1:1 MeCN/acetate buffer pH 5.0) in the presence of either PBN or DEPMPO spin-traps. In both cases, weak yet well detectable signals attributable to the trapping of PhS^•^ radicals were recorded (Figure 2, A and B, respectively), as assessed from the agreement of spectral parameters with the literature,^62−64^ proving the intermediacy of the thiyl radical in the reaction path to the quinone adduct.

**Figure 3:**
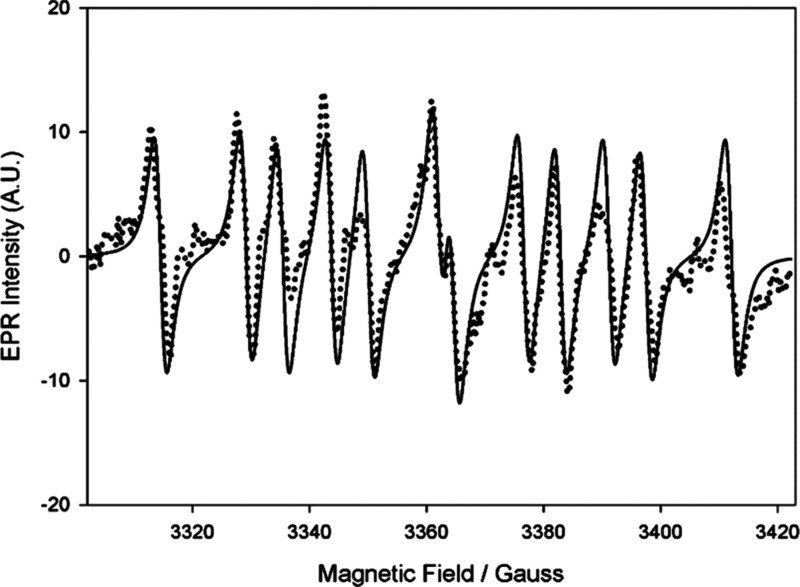
EPR spectrum (dotted lines) and corresponding computer simulation (full line) recorded in spin-trapping experiments with 50 mM DEPMPO, 8.5 mM cysteine, 5 mM 4-MBQ in 1:1 MeCN/acetate buffer (pH = 5.0), showing the trapping of the CyS^•^ radical.

We then turned to cysteine as the reacting thiol. Using PBN as the spin-trap, no clearly attributable EPR signals were recorded under typical settings, except in one experiment in which a very noisy EPR signal consistent with the expected spin adduct was recorded (Figure S18). Despite the good agreement of the spectral parameters (*a*_N_ = 15.1 G; *a*_H_ = 3.0 G; *g* = 2.0068) with the literature,^62^ the modest intensity of the spectrum and the lack of reproducibility did not provide unambiguous assignment. Using DEPMPO as the spin-trap, we detected EPR signals which were reproducible albeit weak (Figure 3) and whose spectral parameters (*a*_N_ = 14.4 G; *a*_H_ = 19.8 G; *a*_P_ = 47.0 G; *g* = 2.0064) are compatible with the trapping of a cysteine-derived thiyl radical.^63,64^

As previously noted for *Z*/*E* isomerization experiments, we attribute the modest signals obtained with cysteine in spin-trapping experiments to the marked instability and high reactivity of CyS^•^ (aliphatic) thiyl radicals in solution, which affords only low steady-state concentrations formed as the intermediate in the reaction of CySH with 4-MBQ.

In order to find independent confirmation of the intermediacy of thiyl radicals in the reaction of thiols such as cysteine with *ortho*-quinones, we reverted the approach and synthesized nitrosocysteine CyS-NO and the corresponding nitroso derivative of *N*-acetylcysteine (NAC-NO),^65^ which provide a well-documented photochemical source of the corresponding thiyl radicals.^66,67^ We then performed spin-trapping experiments by photolyzing the nitrosothiols in the cavity of the EPR spectrometer in MeCN/acetate buffer (pH = 5.0) in the presence of PBN and obtained good-quality EPR signals of the thiyl radical adducts, which were identified on the basis of their hyperfine structure (Figure S19).^62,64^

Having obtained clear evidence that thiyl radicals are formed under our experimental settings, we then replaced the spin trap with 4-MBQ and irradiated the mixture under identical settings. The reaction mixture was then subjected to HPLC–MS analysis to verify the formation of the thiyl adduct of the quinone. Although the analysis confirmed the formation of the expected adduct and of the disulfides of CySH and NAC, it was also apparent that our starting reactants (CyS-NO and NAC-NO) contained relevant amounts of the corresponding thiols and disulfides (Figures S20–S22); hence, our experiments could not provide unambiguous evidence that the observed adducts to the quinone derived from the thiyl radicals (instead of the thiols). Attempts to purify the starting nitrosoderivatives by removing the corresponding thiols were unsuccessful; therefore, we turned to glutathione as the probe thiol.

Synthesis of GS-NO afforded the nitroso thiol in pure form, without impurities of the starting thiol (Scheme S2 and Figure S23).

**Figure 4:**
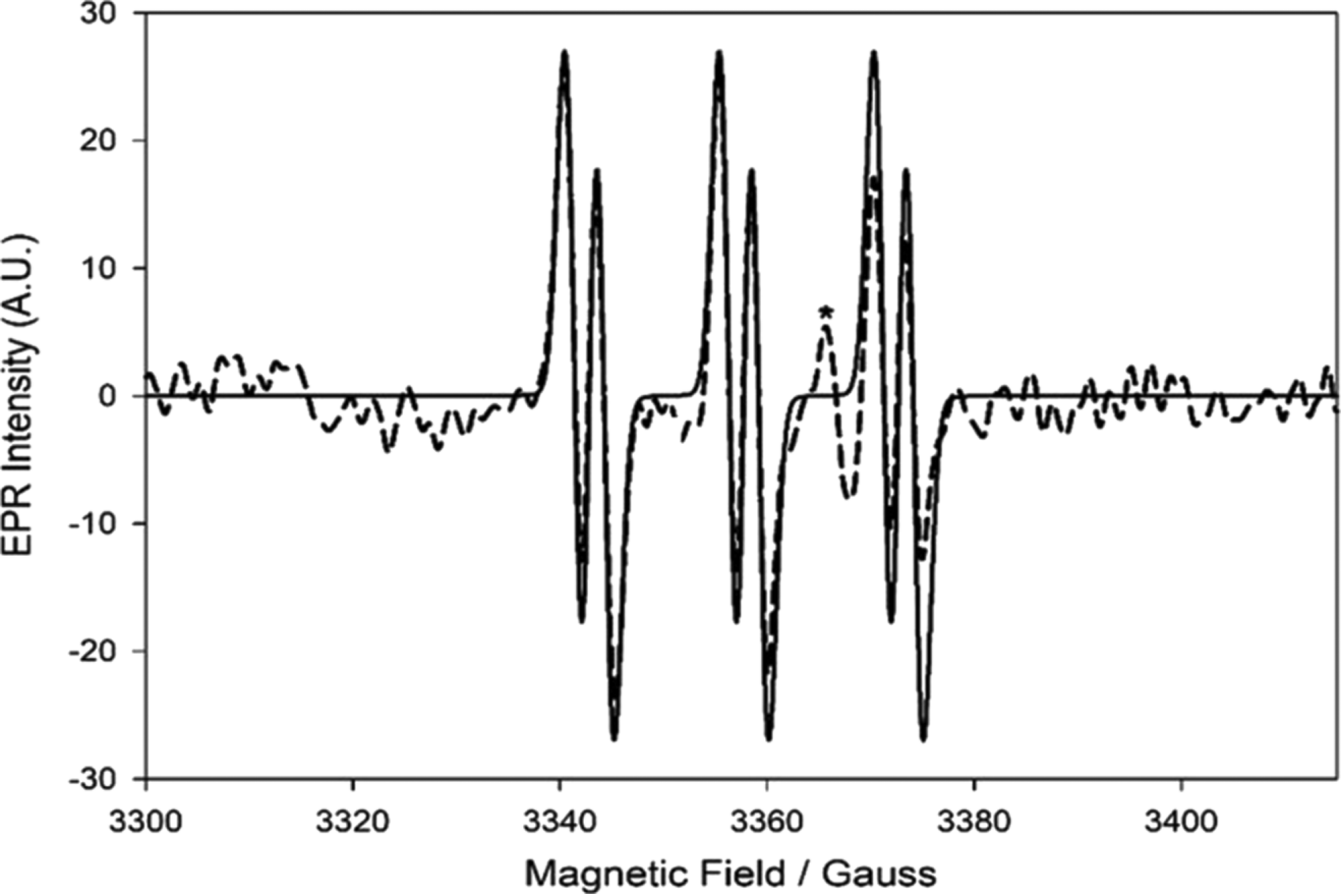
Spin trapping of the thiyl radical GS^•^ upon photolyzing a solution of GS-NO (1.7 mM) in the presence of PBN (9 mM) in MeCN/acetate buffer (pH = 5) at 30 °C (dashed line; the asterisk indicates the persistent signal of the EPR cavity Dewar). Simulated spectrum (full line): *a*_N_ = 15.1 G, *a*_H_ = 3.1 G, *g*-factor = 2.0070.

Spin trapping with PBN afforded good EPR signals, which were unambiguously assigned to the trapped GS^•^ thiyl radical (Figure 4) on the basis of its spectroscopic parameters.^64,67,68^

**Scheme 4:**
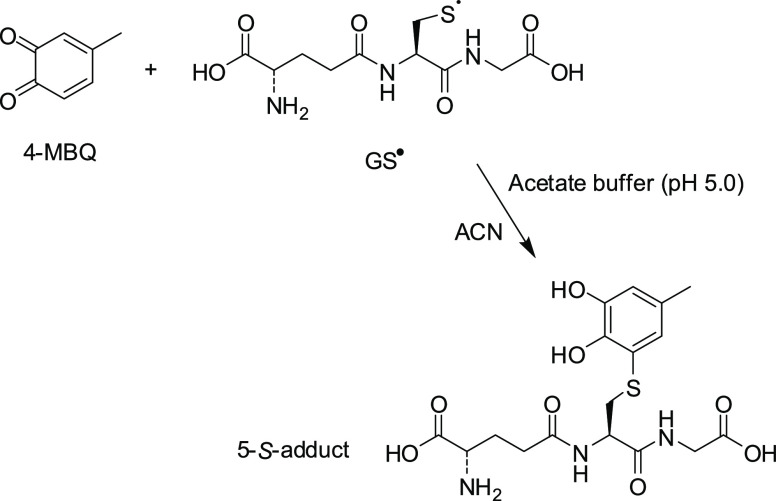
Generation of 4-MBQ/GS Adduct under Radical Conditions

To confirm the generation of 4-MBQ/GS adduct under radical conditions (Scheme 4), two matched reaction mixtures were prepared:

1 a solution containing 1 mM 4-MBQ and 1 mM GS-NO in acetate buffer/MeCN (pH 5.0), which was irradiated with a 400 mW Hamamatsu UV-lamp (4500 mW/cm^2^, set at 50%) for 5 min at 30 °C;
2 a solution containing 1 mM 4-MBQ and 1 mM GSH in acetate buffer/MeCN (pH 5.0), which was incubated in the dark at 30 °C.

Both reaction mixtures were subjected to HPLC-Q-TOF analysis. The analysis showed the generation of the same adduct in the reaction mixtures 1 and 2 (Figures S24 and S25) with a characteristic ion signal at *m*/*z* 430 and an identical MS/MS fragmentation pattern (*m*/*z* 130, 181, 198, 284 see Figures S24 and S25), proving that the two reaction mixtures afford an identical 4-MBQ/GS adduct that was identified as the 5-GS adduct by comparison of the chromatographic behavior with that of an authentic sample.^28^ This provides confirmation that the reaction of thiols such as GSH and *ortho*-quinones such as 4-MBQ proceeds via a radical mechanism where attack is carried out by thiyl radicals.

### 2.3 DFT Calculations

**Scheme 5:**
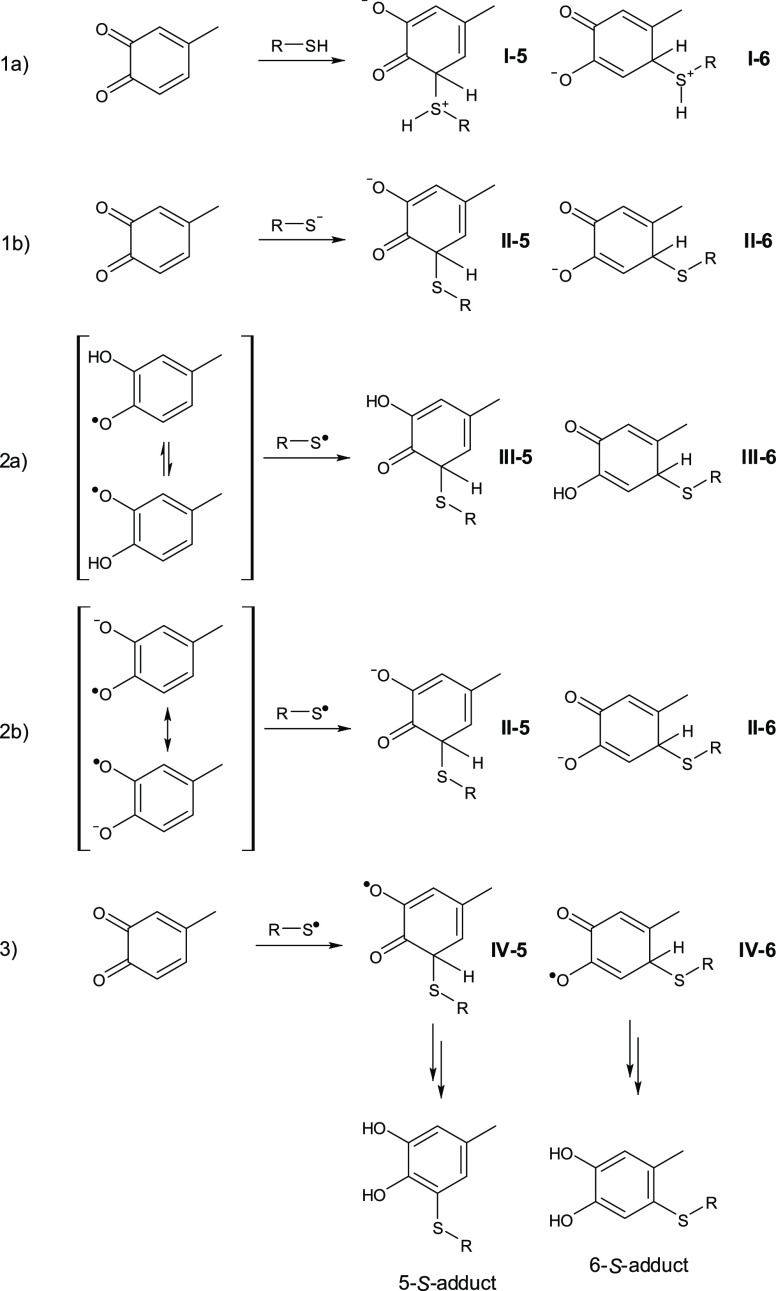
Proposed Pathways of the 4-MBQ Reactions with Thiols

To support the main conclusions from the above sets of experiments and to gain a deeper insight into the origin of the anomalous regiochemistry, a systematic computational investigation was carried out at the DFT level of theory (see the Supporting Information). Initially, the relative energies were computed for the various regioisomers that can be formed by the reaction of the thiol with the *o*-quinone via three alternate pathways: (**1a**) nucleophilic addition of thiol or (**1b**) of thiolate to quinone; (**2a**) thiyl radical-phenoxyl radical and (**2b**) thiyl radical-semiquinone radical anion coupling, and (**3**) addition of thiyl radical to quinone (Scheme 5). Coupling pathways **2** and **3** would be consequent to a preliminary electron transfer step between the thiol and the quinone, leading to thiyl radicals and semiquinones.

Free energies were computed in vacuo and in water (PCM)^69^ using the PBE0^70^ density functional and the 6-31+G(d,p) basis set. For comparison, only the initially formed adducts were considered under the reasonable assumption of kinetically controlled processes (see Supporting Information Tables S5–S8).

Table 1 reports relative free energies (kcal mol^–1^) for the regioisomers corresponding to structures **I-IV** produced by pathways **1a**, **1b**, **2a**, **2b**, and **3** (detailed computational results are provided as the Supporting Information).

**Table 1:** Relative Free Energies (kcal mol^–1^) for the Regioisomers Depicted in Scheme 5 Calculated at Three Different DFT Levels in Vacuo and in Water

path	species	PBE0/6-31+G(d,p)		ωB97X-D/6-31+G(d,p)		M06-2X/6-311++G(2d,2p)^a^	
		in vacuo	in water (PCM)	in vacuo	in water (PCM)	in vacuo	in water (SMD)
**1a**	**I-5**	n.d.^b^	unstable^c^	n.d.^b^	unstable^c^	n.d.^b^	n.d.
	**I-6**	n.d.^b^	unstable^d^	n.d.^b^	unstable^d^	n.d.^b^	n.d.
**1b/2b**	**II-5**	n.d.^b^	0.3	n.d.^b^	0.8	n.d.^b^	2.4
	**II-6**	n.d.^b^	0.0	n.d.^b^	0.0	n.d.^b^	0.0
**2a**	**III-5**	3.5	4.5	3.0	4.9	4.3	5.0
	**III-6**	0.0	0.0	0.0	0.0	0.0	0.0
**3**	**IV-5**	0.0	0.0	0.0	0.0	0.0	0.0
	**IV-6**	6.3	5.2	6.2	4.3	4.8	2.8

a At the PBE0/6-31+G(d,p) (in vacuo) or PBE0/6-31+G(d,p), PCM (in water) geometries.

b Charged/zwitterionic species were examined only in water.

c Most starting structures of the zwitterionic adduct dissociate during optimization; only few minima have been identified. The computed Δ_r_*G*^0^ for the reaction (PBE0 level) is 34.5 kcal mol^–1^.

d All starting structures examined dissociate during optimization.

The data predict that reaction path **1a** (leading to a zwitterionic adduct) should be energetically unfeasible. As for the other paths, the initial adduct at C-6 is more stable than the isomer at C-5 in pathways **1b/2b** and **2a**, whereas *only in route***3** the adduct at C-5 (**IV-5** in Scheme 5) is the favored isomer on an energetic basis.

The whole set of calculations was repeated using the ωB97X-D functional (Tables S11–S14),^71^ which has been specifically validated to model thio-Michael additions.^72^ Moreover, single-point calculations employing the M06-2X functional,^73^ a large basis set, and the Solvation Model based on Density (SMD)^74^ parameterization for the solvent were performed (at the PBE0 geometries). However, the results (Table 1) displayed the same overall trend. Further validation of the computational level chosen was obtained by single-point calculations at the CCSD(T) level in vacuo (Table S18), which displayed a satisfactory agreement with the corresponding DFT results.

A set of control calculations was carried out on the analogous adducts resulting from the addition of methylamine and thiourea to 4-methyl-*o*-quinone by an ionic mechanism (Tables S9 and S10). The results confirmed the greater stability of adducts at C-6, consistent with experimental evidence. Interestingly, preferential addition at C-5 is predicted when a radical addition akin to route **3** is modeled with aminyl or isothioureyl radicals.

**Scheme 6:**
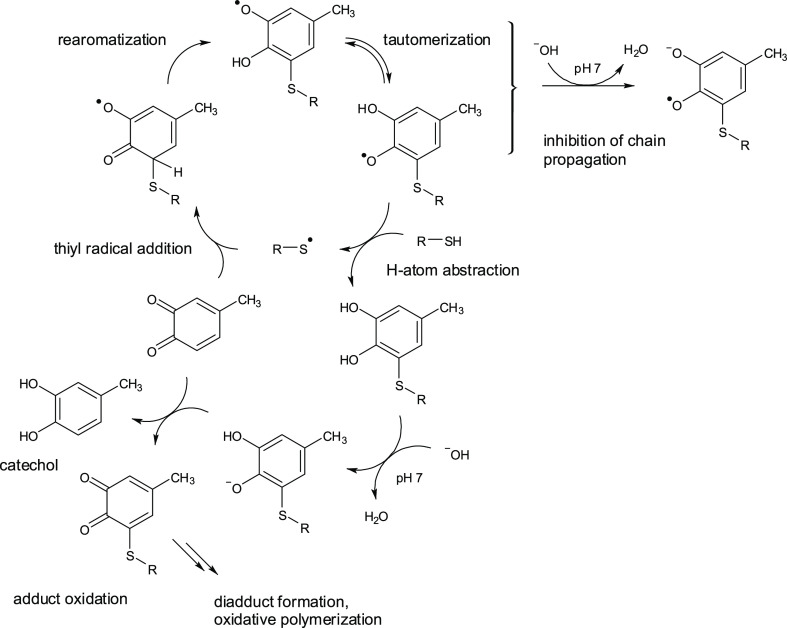
Proposed Mechanism for the Formation of the 5-*S*-Adduct

The above computational analysis disclosed thiyl radical addition to the quinone as the sole mechanism compatible with the anomalous regiochemistry via 1,6-addition. Verification of the proposed mechanism in the light of the previous and present experimental data, including the observed bell-shaped adduct yield profile, requires however a more complex analysis that takes several factors into consideration, including the role of oxygen, substrate and adduct protonation/deprotonation, and redox exchange between the various species. A plausible mechanism that may be proposed to account for most of the experimental evidence is sketched in Scheme 6 (for simplicity, only the prevalent 5-*S*-adduct is reported).

In this scheme, a thiyl radical, even if produced in trace amounts by oxidizing agents present in the medium (including the starting quinone or a radical derived therefrom), would initiate a free radical chain process by adding to the *o*-quinone to form adduct **IV-5** as the main product. Two alternative reaction channels available to the first-formed adduct have been explored, namely, (i) H-atom abstraction from methanethiol or (ii) rearomatization to the corresponding semiquinone. This latter process was modeled under conditions of general base catalysis in water, using fluoride as a weak base on account of the lesser computational complexity involved. As it turns out, path (i) displays a sizeable barrier (Table S19), whereas path (ii) features very small barriers or is even barrierless (Figure S26) and should therefore represent the main fate of **IV-5**. The resulting alkylthiophenoxyl radical would then abstract an H atom from the thiol, thus regenerating the thiyl radical and completing a chain cycle. With a computed Δ_r_*G*° (in water) of 7.2 kcal mol^–1^, this is likely the rate-limiting step of the whole reaction chain.

The ratio of formation of the final products (5-*S* vs 6-*S*) is determined in the step of addition of the thiyl radical to the *o*-quinone, which features low but measurable activation free energies and is significantly exergonic. At the theory level generally adopted in this study (M06-2X/6-311++G(2d,2p), SMD//PBE0/6-31+G(d,p), PCM), the transition state for addition to position 5 has a free energy of 4.9 kcal mol^–1^ relative to the 4-MBQ and thiyl reactants, to compare with 6.0 kcal mol^–1^ for addition to position 6. We also examined this reaction step at a higher theory level, using M06-2X/6-311++G(2d,2p), SMD geometry optimizations to locate the transition structures and performing single point electronic energy evaluations at the DLPNO-CCSD(T)-F12/cc-pVTZ-F12 level:^75,76^ in this case, the computed activation free energy for addition to the 5-position is 6.0 kcal mol^–1^, as opposed to 7.4 kcal mol^–1^ for addition to the 6-position.

Table 2 presents computed free energies (kcal mol^–1^) for the main reaction steps (considering only neutral species).

**Table 2:** Reaction Free Energies Δ_r_*G*° and Selected Activation Free Energies Δ^‡^*G*° (kcal mol^–1^) in Water^a^ for the Reaction Steps Proposed in Scheme 6^b^

step	Δ_r_*G*°	Δ^‡^*G*°
thiyl radical addition	–11.5	4.9 (6.0)^c^
rearomatization and tautomerization	–23.2	d
H-atom abstraction	7.2	

a M06-2X/6-311++G(2d,2p), SMD//PBE0/6-31+G(d,p), PCM.

b All energies are referred to the reactants of the individual step.

c In parentheses, the value computed at the DLPNO-CCSD(T)-F12/cc-pVTZ-F12//M06-2X/6-311++G(2d,2p), SMD level.

d Under conditions of general base catalysis, C-5 deprotonation of **IV-5** is a low barrier or a barrierless step.

While not all aspects of the observed reactivity can be completely clarified with the available data, the mechanism depicted in Scheme 6 would also be consistent with the following observations:

1 At pH 7.0, the rearranged alkylthiophenoxyl radical would undergo deprotonation to give a semiquinone radical anion, thus disfavoring H-atom transfer from the thiol and effectively inhibiting the progression of the chain mechanism. Moreover, deprotonation of the alkylthiocatechol product would increase its ease of oxidation via redox exchange with the starting quinone resulting in both 4-methylcatechol accumulation and adduct conversion to the corresponding quinone. This latter step would account for diadduct formation and the general decrease in the adduct yield at pH 7.0.
2 At pH 4.0, when both autoxidation and anion formation are minimized, allowance of oxygen into the medium would ensure both a greater efficiency of the thiyl radical generating initiation steps and the re-oxidation of any catechol/semiquinone produced by redox exchange, thus regenerating the quinone and enhancing the key coupling step.

## 3 Conclusions

The anomalous regiochemistry of the coupling reaction of thiols, for example, cysteine, with *o*-quinones was re-examined herein by an integrated experimental and computational approach. Both experimental and theoretical results pointed strongly toward a mechanism based on generation of thiyl radicals and their subsequent addition to the *o*-quinone. This latter step would promote a free radical chain process in which alkylthiophenoxyl radicals would continuously produce thiyl radicals from the thiol via H-atom abstraction. The possibility that, depending on specific pH regimes and conditions, this pathway may coexist with other routes cannot be ruled out at this stage. However, the proposed free radical mechanism would be compatible with the observed dependence of product yield and distribution on both air and pH and would open new vistas into a range of reactions and processes of biological relevance.

## Supporting Information Available

UV spectra of (*Z*)-stilbene and (*E*)-stilbene; HPLC profile of the reaction at pH 5; formation yields of all the adducts in the presence of Trolox at various pHs; GC–MS and HPLC-Q-TOF(MS) analysis; NMR spectra of 4-MBQ, GS-NO, and 5-methyl-3-(phenylthiol)benzene-1,2-diol; EPR spin-trapping experiments; and computational analysis (PDF)
