# Supplementary material for: Disentangling the Puzzling Regiochemistry of Thiol Addition to o-Quinones
Source: J Org Chem. 2022 Mar 10;87(7):4580–9. doi: 10.1021/acs.joc.1c02911 (PMC8981336; doi:10.1021/acs.joc.1c02911)
Supplement: Supplementary file 1 — jo1c02911_si_001.pdf [file jo1c02911_si_001.pdf]

Supporting Information

Disentangling the Puzzling Regiochemistry of Thiol Addition to *o*-Quinones

Maria L. Alfieri‡1, Alice Cariola‡2, Lucia Panzella[1], Alessandra Napolitano[1], Marco d'Ischia[1], Luca Valgimigli[2*], and Orlando Crescenzi[1*]

[1]Department of Chemical Sciences, University of Naples Federico II, Via Cintia 21, I-80126 Naples, Italy
[2]Department of Chemistry "Giacomo Ciamician", University of Bologna, Via S. Giacomo 11, I-40126 Bologna, Italy
‡These authors contributed equally to this work.

Corresponding Author: luca.valgimigli@unibo.it, orlando.crescenzi@unina.it

Table of Contents

**Experimental Procedures**	p. S3
**Figure S1.** HPLC profile of the reaction cysteine with 4-MBQ 10:1 molar ratio at pH 5.0.	p. S6
**Figure S2.** Formation yields of the products of the reaction of cysteine with 4-MBQ 10:1 molar ratio in the presence and in the absence of air at various pH.	p. S7
**Figure S3.** Effect of Trolox on the formation yields of the adducts at various pHs.	p. S8
**Figure S4.** UV spectra of (*Z*)-stilbene and (*E*)-stilbene in MeCN.	p. S9
**Figure S5.** GC-MS analysis of (*Z*)-stilbene and (*E*)-stilbene in MeCN.	p. S10
**Figure S6.** GC-MS analysis of the reaction mixture of PhSH (1 mM) and 4-MBQ in the presence of (*Z*)-stilbene in MeCN.	p. S11
**Figure S7.** GC-MS analysis of the reaction mixture of PhSH (10 mM) and (*Z*)-stilbene in MeCN with or without 4-MBQ.	p. S12
**Figure S8.** GC-MS analysis of the reaction mixture of PhSH, (*Z*)-stilbene and AIBN in MeCN.	p. S13
**Figure S9.** Time evolution of the UV spectrum of a solution of (*Z*)-stilbene, PhSH and AIBN in MeCN.	p. S14
**Figure S10.** GC-MS analysis of the reaction mixture of HSEtOH and (*Z*)-stilbene with 4-MBQ or TBPB.	p. S15
**Figure S11.** GC-MS analysis of the reaction mixture of LipCys, (*Z*)-stilbene and 4-MBQ in MeCN.	p. S16
**Figure S12.** GC-MS analysis of the reaction mixture of thioacetic acid, (*Z*)-stilbene and 4-MBQ in MeCN.	p. S17
**Figure S13.** ^{1}H NMR spectrum of 5-methyl-3-(phenylthiol)benzene-1,2-diol (400 MHz, DMSO-d6).	p. S18
**Figure S14.** ^{13}C NMR spectrum of 5-methyl-3-(phenylthiol)benzene-1,2-diol (400 MHz, DMSO-d6).	p. S18
**Figure S15.** ^{1}H,^{1}H COSY spectrum of 5-methyl-3-(phenylthiol)benzene-1,2-diol (400 MHz, DMSO-d6).	p. S19
**Figure S16.** ^{1}H,^{13}C HSQC spectrum of 5-methyl-3-(phenylthiol)benzene-1,2-diol (400 MHz, DMSO-d6).	p. S19
**Figure S17.** ^{1}H,^{13}C HMBC spectrum of 5-methyl-3-(phenylthiol)benzene-1,2-diol (400 MHz, DMSO-d6).	p. S20
**Scheme S1.** Spin trapping of thiyl radicals.	p. S21
**Figure S18.** EPR spectrum of a solution of PBN, CySH and 4-MBQ and corresponding simulated spectrum.	p. S22
**Figure S19.** Spin-trapping experiments by photolyzing nitrosothiols (Cys-NO and NAC-NO) in the cavity of the EPR spectrometer in MeCN/acetate buffer (pH = 5.0) and corresponding simulated spectra	p. S23
**Figure S20.** UPLC-Q-TOF chromatogram of *S*-nitroso-*N*-acetylcysteine NAC-NO in acetate buffer/MeCN (pH 5.0).	p. S24
**Figure S21.** Q-TOF mass spectra of NAC-NO, NAC and the corresponding disulphide.	p. S25
**Figure S22.** UPLC-Q-TOF chromatogram of *S*-nitrosocysteine in acetate buffer/MeCN (pH 5.0).	p. S26
**Scheme S2.** Synthesis of *S*-nitrosoglutathione and reaction scheme of spin-trapping experiments of GSNO in the presence of PBN.	p. S27
**Figure S23.** UPLC-Q-TOF chromatogram of *S*-nitrosoglutathione in acetate buffer/MeCN (pH 5.0).	p. S28
**Figure S24.** UPLC-Q-TOF chromatogram of the adduct obtained by irradiating a solution of 4-MBQ and GS-NO.	p. S29
**Figure S25.** UPLC-Q-TOF chromatogram of the adduct obtained by mixing 4-MBQ with GSH.	p. S30
**Computational studies:** Part 1-19<br>**Part 1 (Tables S1.1-1.5).** Conformational exploration of methanethiol in vacuo or in water.<br>**Part 2 (Tables S2.1-2.5).** Conformational exploration of thioacetic acid in vacuo or in water.<br>**Part 3 (Tables S3.1-3.5).** Conformational exploration of thiourea in vacuo or in water.<br>**Part 4 (Tables S4.1-4.9).** Conformational exploration of 4-methylcatechol in vacuo or in water.<br>**Part 5 (Tables S5.1-5.9).** Conformational exploration of 4-methylcatechol-methanethiol adducts in vacuo or in water.<br>**Part 6 (Tables S6.1-6.8).** Conformational exploration of 4-methylcatechol-methanethiol rearomatized adducts in vacuo or in water.<br>**Part 7 (Tables S7.1-7.6).** Conformational exploration of 4-methylcatechol-thioacetic acid adducts in vacuo or in water.<br>**Part 8 (Table S8.1).** Conformational exploration of 4-methylcatechol-azide adducts in water.<br>**Part 9 (Tables S9.1-9.6).** Conformational exploration of 4-methylcatechol-methylamine adducts in vacuo or in water.<br>**Part 10 (Tables S10.1-10.6).** Conformational exploration of 4-methylcatechol-thiourea adducts in vacuo or in water.<br>**Part 11 (Tables S11.1-11.7).** Conformational exploration of 4-methylcatechol-methanethiol adducts in vacuo or in water using the ωB97X-D functional.	p. S31<br>p. S32<br>p. S34<br>p. S39<br>p. S44<br>p. S63<br>p. S99<br>p. S155<br>p. S190<br>p. S194<br>p. S236<br>p. S338

**Part 12 (Tables S12.1-12.8).** Conformational exploration of 4-methylcatechol-methanethiol rearomatized adducts in vacuo or in water using the ωB97X-D functional.<br>**Part 13 (Tables S13.1-13.6).** Conformational exploration of 4-methylcatechol-thioacetic acid adducts in vacuo or in water using the ωB97X-D functional.<br>**Part 14 (Table S14.1).** Conformational exploration of 4-methylcatechol-azide adducts in water using the ωB97X-D functional.<br>**Part 15 (Tables S15.1-15.2).** Conformational exploration of 4-methylcatechol-methanethiol adducts in water at the DLPNO-CCSD(T)-F12 / cc-pVTZ-F12 // M06-2X / 6-311++G(2d,2p), SMD level.<br>**Part 16 (Table S16.1).** Conformational exploration of methanethiol in water at the DLPNO-CCSD(T)-F12 / cc-pVTZ-F12 // M06-2X / 6-311++G(2d,2p), SMD level.<br>**Part 17 (Table S17.1).** Conformational exploration of 4-methylcatechol in water at the DLPNO-CCSD(T)-F12 / cc-pVTZ-F12 // M06-2X / 6-311++G(2d,2p), SMD level.<br>**Part 18 (Table S18.1).** Reference conventional coupled-cluster calculations in vacuo (at the PBE0/6-31+G(d,p) geometries).<br>**Part 19 (Table S19.1).** Transition structures for the process of H-atom abstraction form methanethiol by the initial 5-S-adduct.	p. S372<br>p. S432<br>p. S480<br>p. S484<br>p. S490<br>p. S491<br>p. S492<br>p. S494
**Figure S26.** Relaxed potential energy scans for the deprotonation of the methanethiyl - 4-methyl-*o*-benzoquinone 5-*S*-adduct.	p. S496
**Figure S27.** UPLC-Q-TOF chromatogram of 4-MBQ in acetate buffer/MeCN (pH 5.0).	p. S498
**Figure S28.** ^{1}H-NMR spectrum of 4-MBQ (400 MHz, $CDCl_3$).	p. S499
**Figure S29.** Effect of Trolox on the formation yields of 5-*S*-adduct at pH 4.0.	p. S500
**Figure S30.** ^{1}H-NMR spectrum of GS-NO in D_2O at 400 MHz.	p. S501
**References**	p. S502

Experimental Procedures

Materials and methods

4-methyl-*o*-catechol, L-cysteine, thiophenol (PhSH), 6-hydroxy-2,5,7,8-tetramethylchromane-2-carboxylic acid (Trolox), (*Z*)-stilbene, (*E*)-stilbene, 2,2'-azobisisobutirronitrile (AIBN), mercaptoethanol (HSEtOH), *N*-(*tert*-butoxycarbonyl)-(*L*)-cysteine methyl ester (LipCys), *tert*-butyl perbenzoate, α-phenyl *N*-tertiary-butyl nitrone (PBN), 5-(diethoxyphosphoryl)-5-methyl-1-pyrroline-*N*-oxide (DEPMPO), sodium nitrite, *N*-acetylcysteine and glutathione were purchased from Sigma Aldrich (Milan, Italy), sodium periodate was purchased from Carlo Erba (Milan, Italy). 6-*S*-cysteinyl isomer was prepared as previously described.[1]
UV-vis spectra were recorded with a Jasco V-560 UV-vis spectrophotometer (Lecco, Italy) or with a Biomate 5 (Thermo Scientific, Milan, Italy) coupled with a Heto DBT Hetotherm (Birkerød, Denmark) thermostatting water circulator for temperature control set at 30°C.
^{1}H NMR spectra were recorded in $CDCl_3$, in D_2O, or in DMSO at 400 MHz on a Bruker spectrometer. ^{13}C NMR spectra were recorded in $CDCl_3$ at 400 MHz on a Bruker 400 MHz spectrometer. ^{1}H,^{1}H COSY, ^{1}H,^{13}C HSQC, and ^{1}H,^{13}C HMBC were run at 400 MHz using Bruker standard pulse programs. Structural assignments were made with additional information from gCOSY, gHSQC, and gHMBC experiments.
Analytical and preparative TLC were carried out on silica gel plates (0.25 and 0.50 mm, respectively) from Merck using chloroform/methanol 9:1 v/v as the eluant.
HPLC analyses were performed on an Agilent 1100 binary pump instrument (Agilent Technologies, Milan, Italy) equipped with a SPD-10AV VP UV-visible detector using a Phenomenex Sinergy 4U Hydro-RP column (250 mm × 4.6 mm, 4 μm particle size) at 0.7 mL/min. Detection wavelength was set at 254 nm. Eluant system: 0.1 % formic acid - methanol 75:25 v/v.
LC-MS analyses were performed in positive ion mode using an Agilent 1260/6230DA ESI-TOF instrument in the following conditions: nebulizer pressure 35 psig; drying gas (nitrogen) flushed at 5 L / min at a temperature of 325 ° C; capillary voltage 3500 V; fragmentor voltage 175 V. An Eclipse Plus C18 column (150 × 4.6 mm, 5 μm) at a flow rate of 0.4 mL / min was used, using the same eluant as above.

Synthesis of 4-methyl-o-quinone (4-MBQ)

The title compound was prepared following a procedure previously reported with modifications.[2]
To a solution of 4-methylcatechol (4 mmol, 40 mL) in cold water, sodium periodate (4.4 mmol), previously dissolved in cold water, was rapidly added, and the mixture was kept under vigorous stirring in an ice bath. After 30 s the mixture was extracted with cold dichloromethane (3×50 mL). The organic layers were dried over anhydrous sodium sulfate and taken to dryness to give a red-brown powder in 96 % yield and pure form as confirmed by UV-vis analysis, HPLC-Q-TOF analysis (Figure S27) and ^{1}H-NMR (Figure S28).
^{1}H NMR ($CDCl_3$) δ (ppm): 6.72 (d, 1H), 6.33 (d, 1H), 6.20 (s, 1H), 2.21 (s, 3H).

GC-MS analysis of stilbene isomerization

GC-MS analyses were performed in a Agilent 6890N-5973N system equipped with 7683B autosampler (Agilent Technologies, Milan, Italy) mounting a HP-5MS 0.25 mm × 30 m × 0.25 μm column, using He as carrier gas at flow rate 1 mL/min. Temperature programming was: 100°C, hold 1 min, ramp to 270°C at 30°C/min, hold 8 min. Injector and detector temperatures were 250°C and 270°C respectively, injection volume 1 μL, split ratio 20:1. Mass spectra were recorded in EI$^+$ (70 eV) mode in the range 32-800 *m/z*.
For $Z \rightarrow E$ isomerization studies a mixture of 0.1 mM (*Z*)-stilbene was incubated with PhSH (1-10 mM) and 1 mM 4-MBQ in MeCN at 30 °C and analyzed at different time-intervals. Control experiments lacking 4-MBQ were performed. Thioacetic acid, HSEtOH and LipCys were also tested as the thiol reactant in place of PhSH. As a positive control 0.1 mM (*Z*)-stilbene was incubated with a thiol (1-10 mM) and AIBN or *tert*-butyl perbenzoate (TBPB) (5-10 mM) at 30-70°C.

HPLC-Q-TOF(MS) analysis of quinone/thiol reactions

Analyses were performed on a Acquity UPCL H-Class Plus coupled to a Xevo G2-XS QTOF spectrometer (Waters, Milan Italy) mounting a SepaChrom Adams C18-classic (250 mm × 4.6 mm, 5 μm particle size) column eluted isocratically with 30% A (0.1% trifluoroacetic acid in MeCN) and 70% B (0.1% trifluoroacetic acid in water) at flow rate 0.6 mL/min. ESI$^+$: cone 30V, capillary 0.8 kV, temp. 120°C, desolv. temp. 600°C, cone gas flow 50 L/h, desolv. flow 1000 L/h. Mass range 50 to 1250 m/z, High CE ramp 20 to 30 eV. MS/MS: CID 20 to 30 eV with Ar. TOF: acceleration 10/100V, pusher/puller 1900V/1400V, flight tube 9 kV, reflectron 1.6 kV.
In experiments probing quinone/thiol reaction a mixture of 1 mM 4-MBQ and the S-nitroso thiol (GS-NO, NAC-NO or CyS-NO) at 1 mM contained in a quartz cuvette was irradiated using Hamamatsu UV - lamp (4500 mW/cm^2 at 240-400 nm) provided with an optical fiber, at 30-70% power for 5 minutes at 30°C, then immediately subjected to analysis, alternatively a mixture of 4-MBQ 1 mM and glutathione 1 mM was incubated in the dark at 30 °C for 5-15 min then immediately analyzed.

Reaction of 4-MBQ with cysteine

To a solution of 10 mM cysteine (0.02 mmol) in 50 mM phosphate buffer (2 mL) at pH 7.0, 4-MBQ dissolved in a minimal amount of acetonitrile was added up final concentration 1 mM, and the mixture was kept in air under stirring (400 rpm). After 15 min aliquots were withdrawn, treated with an excess of solid sodium dithionite and analyzed by HPLC and UV-vis spectrophotometry.

The same experiments were also run in 50 mM phosphate buffer at different pHs (in the range 4-7) both in air and under an argon atmosphere.

All experiments were run in triplicate. Identification of 4-MBQ-cysteine adducts was achieved by comparison of their chromatographic behavior with those of authentic samples.[3] Control experiments were run in the absence of cysteine. Selected reaction mixtures were analysed by LC-MS under the conditions detailed above. In other experiments to a solution of cysteine and 4-MBQ both at 1 mM, in 50 mM phosphate buffer at pH 4.0, different amounts of trolox (0.5-5 mM) were added and the mixture was kept in air under stirring. After 3 min withdrawn aliquots were analyzed by HPLC as above (Figure S29). The same experiments were also run at 1:5 catechol-cysteine/Trolox ratio at different pHs (in the range 4-7) under an argon atmosphere and after 3 min were analyzed as above.

Reaction of 4-MBQ with thiophenol

To a solution of 10 mM PhSH (8.2 mmol) in 50 mM phosphate buffer at pH 4.0, a solution of 4-MBQ (0.82 mmol) in acetonitrile was added and the mixture was kept under stirring (400 rpm) in air. After 15 min the mixture was analyzed by HPLC. The chromatographic profile revealed the presence of a major component eluted at t_R =16 min. The mixture was then extracted with ethyl acetate (3x800 mL), and the combined organic phases were taken to dryness after treatment with anhydrous sodium sulphate. TLC analysis showed the presence of a main product (R_f = 0.6, eluant $CHCl_3$/MeOH 9:1 v/v). Purification was run by preparative TLC using $CHCl_3$: MeOH 9:1 v/v containing 1 % acetic acid as eluant to give the product (30% yield) in pure form.

$UV_{\lambda max}$ (MeOH): 256 nm (ε 3630 $M^{-1}cm^{-1}$), 295 nm (ε 5899 $M^{-1}cm^{-1}$).

^1H NMR (DMSO-d_6) δ (ppm): 2.14 (s, 3H), 6.63 (s, 1H), 7.48 (s, 1H), 7.53 (m, 1H), 7.57 (m, 2H), 7.73 (d, 2H).

$^{13}C\{^1H\}$ NMR (DMSO-d_6) δ (ppm): 19.27 (CH_3), 116.87 (CH), 119.80 (CH), 127.29 (CH), 128.15 (C), 129.56 (C), 129.86 (CH), 133.40 (CH), 142.39 (C), 143.82 (C), 151.02 (C).

Synthesis of Cys-NO and NAC-NO[4]

The title compounds were prepared accordingly to protocols previously reported with modifications. Briefly, L-cysteine (0.18 mmol) and sodium nitrite (0.18 mmol) were dissolved in 0.5 M HCl (5 mL) and the mixture was kept under stirring in an ice bath. After 30 min a 0.1 M NaOH solution was added to the mixture up to pH 5.0. The concentration of Cys-NO was estimated by measuring absorbance at 332 nm using a molar coefficient of 0.75 $mM^{-1}cm^{-1}$ as 15.2 mM, corresponding to a yield of ~50%. Similarly, the concentration of NAC-NO was estimated from absorbance at 330 nm using a molar coefficient of 0.73$mM^{-1}cm^{-1}$. HPLC-Q-TOF analysis revealed in both cases the presence of the starting thiol and the corresponding disulphide. The solution was used for product study and spin trapping experiments.

Synthesis of GS-NO[5,6]

To a stirred ice-cold solution of GSH (500 mg, 1.7 mmol) in water (2.5 mL) $NaNO_2$ (120 mg, 1.7 mmol) was added and then acidified by adding HCl 2N (0.9 mL). After 40 minutes during which the temperature was maintained from 0 to 5 °C the red solution was treated with acetone (3.5 mL) and stirred for further 10 minutes. The resulting fine pale red precipitate was filtered off and washed successively with ice-cold water (2 mL), acetone (2 mL) and diethyl ether (2 mL) to afford *S*-nitrosoglutathione (300 mg, 76%), whose identity and purity was assessed by UV-Vis, ^1H NMR (Figure S30), and HPLC-Q-TOF analysis (Figure S23).

UV-$Vis_{\lambda max}$ (H_2O): 335 nm (ε 922 $M^{-1}cm^{-1}$), 545 nm (ε 15.9 $M^{-1}cm^{-1}$).

^1H NMR (D_2O) δ (ppm): 4.60−4.50 (m, 1H), 4.10−3.85 (m, 2H), 3.80 (s, 2H), 3.65 (t, 1H), 2.30 (t, 2H), 2.10−1.95 (m, 2H).

Q-TOF (ESI^+) *m/z*: 337 ($[M+H]^+$, 2%), 307, 289 (bp), 232, 118.

EPR spin-trapping experiments

The X-band EPR spectra were collected in 1 mm quartz tubes with Elexsys 500 (Bruker, Milan, Italy) and a MiniScope MS 5000 (Magnettech, Freiberg, Germany), both equipped with temperature control, and all spectra were recorded at 30°C. When needed UV irradiation in cavity was provided by an optical fiber from a mercury-xenon lamp (Hamamatsu Lightningcure LC8, 240-400 nm, max 4500 mW/cm^2). Spectra were analysed by the WinESR program (developed by Prof. Marco Lucarini, University of Bologna) and subjected to iterative simulation-matching based on the systematic application of MonteCarlo method. Measured *g*-factors were corrected with respect of 2,2,6,6-tetramethylpiperidine-N-oxyl (TEMPO) radical, *g* = 2.0061,[7] and that of 2,2-diphenyl-1-picrylhydrazyl (DPPH) radical in acetonitrile, *g* = 2.0036.[8] Spectra were recorded with the following settings: modulation amplitude 0.1-0.2 mT, sweep width 12 mT, modulation frequency 100 kHz, frequency 9.76 Hz, sweep time 60s, microwave power 10 mW. For spin-trapping experiments 10-100 mM spin trap (PBN or DEPMPO) was added to a mixture of the thiol 1-20 mM and 4-MBQ 1-20 mM in MeCN/acetate buffer (pH = 5.0) 1:1 in a quartz tube in the cavity of the EPR spectrometer (30°C) and spectra were recorded every 5 minutes. For experiments with *S*-nitroso derivatives a mixture of Cys-NO, NAC-NO or GS-NO 1-25 mM and PBN 5-10 mM in MeCN/acetate buffer (pH = 5.0) was briefly irradiated (20-40s, 50% power) in the spectrometer cavity (30°C) and the spectra recorded immediately after.

Computational analysis

Calculations were mostly performed with the Gaussian 09 package of programs;[9] only DLPNO-CCSD(T)-F12 calculations were performed with Orca 4.2.1.[10] The spin-unrestricted formulation was used for open-shell species. All structures were optimized with the PBE0 functional,[11] the PBE hybrid including 25% exact exchange. In several cases, independent optimizations were also carried out with the ωB97X-D functional,[12] a long-range-corrected functional including empirical dispersion, which in a recent study[13] has been specifically validated to model thio-Michael additions. For each species, different tautomers / conformers, as well as different protonation states were explored. In those cases where enantiomers exist, a single enantiomeric series has been explored. All conformers identified were further characterized by single point energy evaluations with the M06-2X functional[14] in conjunction with a much larger basis set, 6-311++G(2d,2p).
Reference conventional coupled-cluster singles and doubles calculations were also carried out,[15] including non-iterative treatment of triple excitations,[16] with the cc-pVTZ basis set[17] or with basis sets of the "calendar" series.[18] For open-shell species, a restricted open-shell reference wave function was used.
Domain-based local pair natural orbital coupled-cluster (DLPNO-CCSD(T)) calculations[19] with inclusion of explicit correlation (F12) in the wavefunction[20] were performed with the cc-pVTZ-F12 basis set[21] and with a "TightPNO" pair natural orbital setting. For a uniform treatment of open- and closed-shell species, the "UseFullLMP2Guess" keyword was set to "false" for the closed shell calculations.
Computations were performed either in vacuo, or by adoption of a polarizable continuum medium (PCM)[22] to account for the influence of the solution environment. In view of the faster convergence, a scaled van der Waals cavity based on universal force field (UFF) radii;[23] non-electrostatic contributions to the solvation free energy were disregarded at this stage: these terms were accounted for in single-point PCM calculations (at the PCM geometries) employing radii and non-electrostatic terms of the SMD solvation model.[24] Vibrational-rotational contributions to the free energy at 298.15 K were also computed. *G* values reported in the tables are referred to a 1 atm standard state; however, they were converted to a 1 M standard state in order to compute the reaction free energies discussed in the paper (see *e.g.* Reference 20).[25]

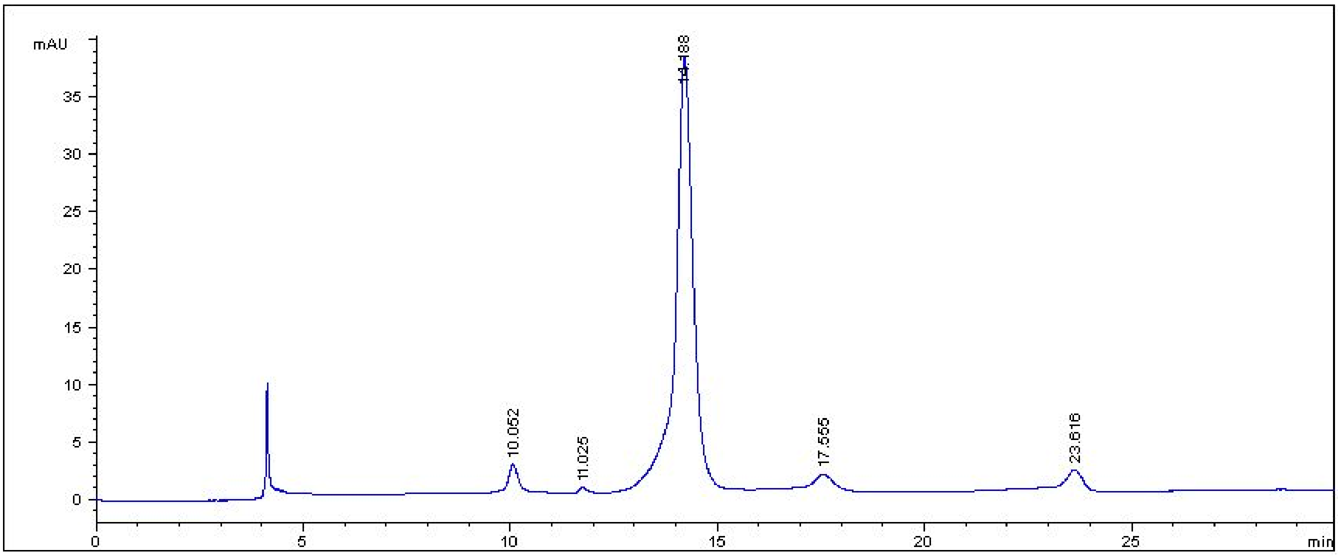

Figure S1. Elutographic profile of the reaction of cysteine with 4-MBQ 10:1 molar ratio at pH 5.0. R.t.14.2 min 5-*S*-isomer, r.t. 17.5 min 2-*S*-isomer, r.t. 10.1 min 6-*S* isomer, r.t. 11 min diadduct and r.t. 23.6 min 4-methylcatechol. Detection wavelength: 254 nm.

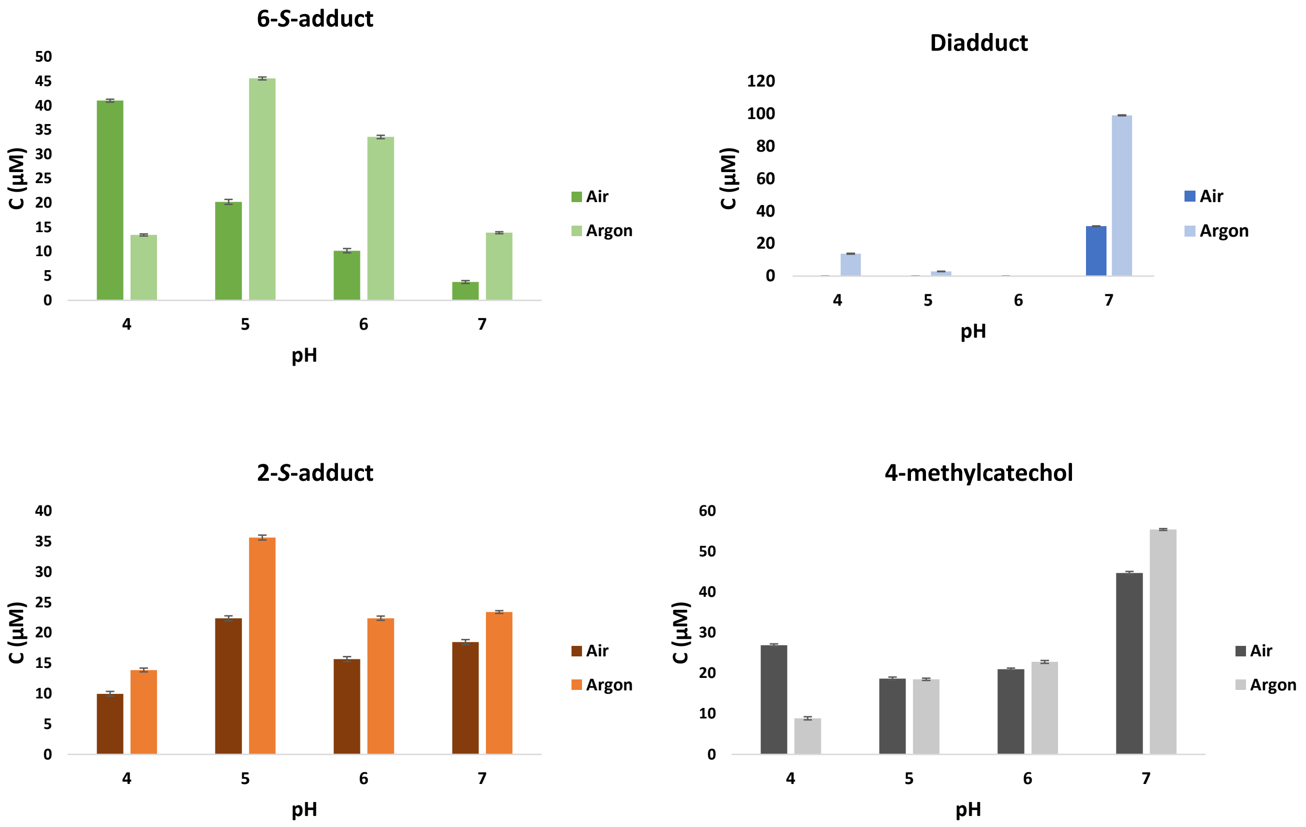

Figure S2. Yields of products of the reaction of cysteine with 4-MBQ 10:1 molar ratio as a function of pH in the presence and in the absence of air.

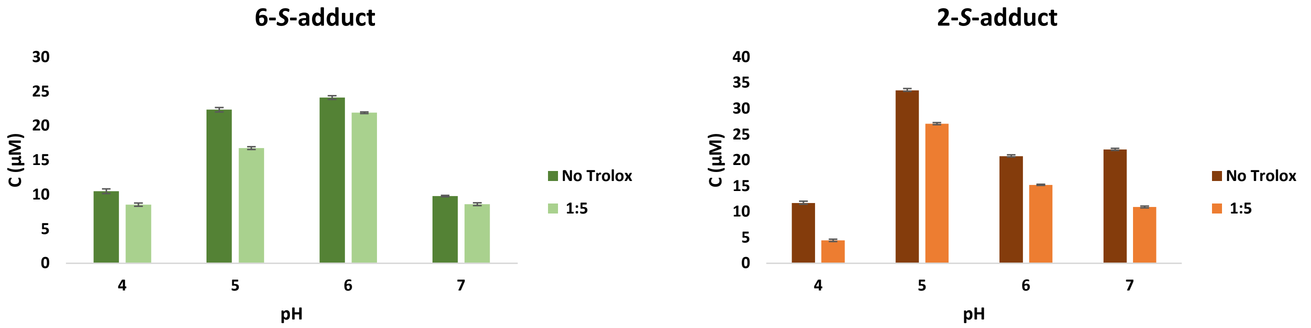

Figure S3. Effect of Trolox on the yields of 2-*S*- and the 6-*S* isomers at 1:5 catechol-cysteine/Trolox ratio as a function of pH in the absence of air.

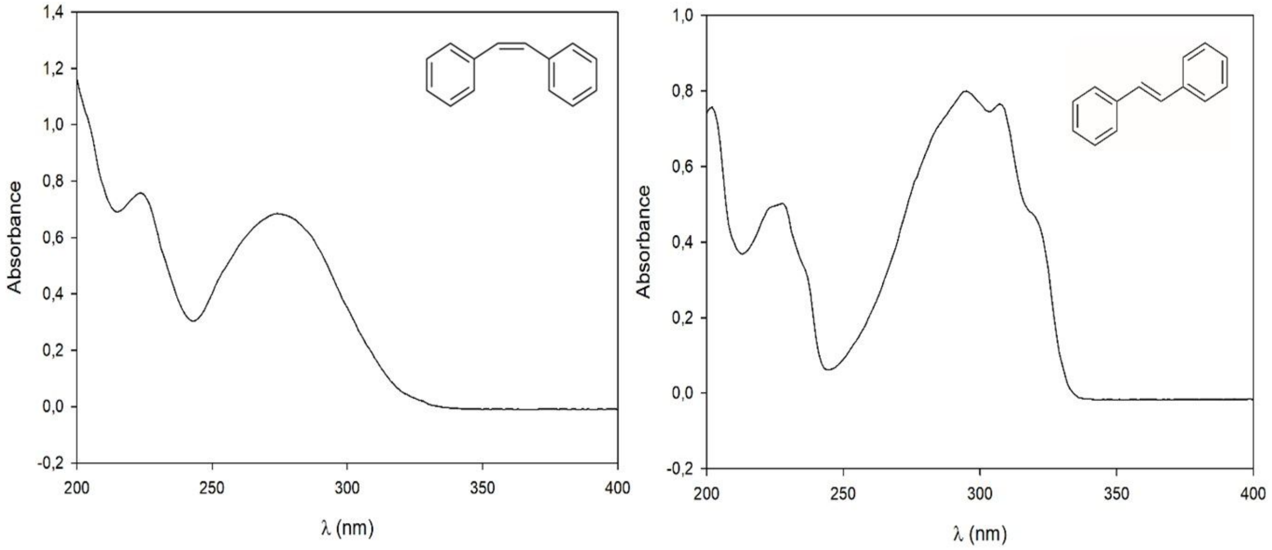

Figure S4. UV spectra of 0.036 mM (*Z*)-stilbene (left) and 0.036 mM (*E*)-stilbene (right) in MeCN.

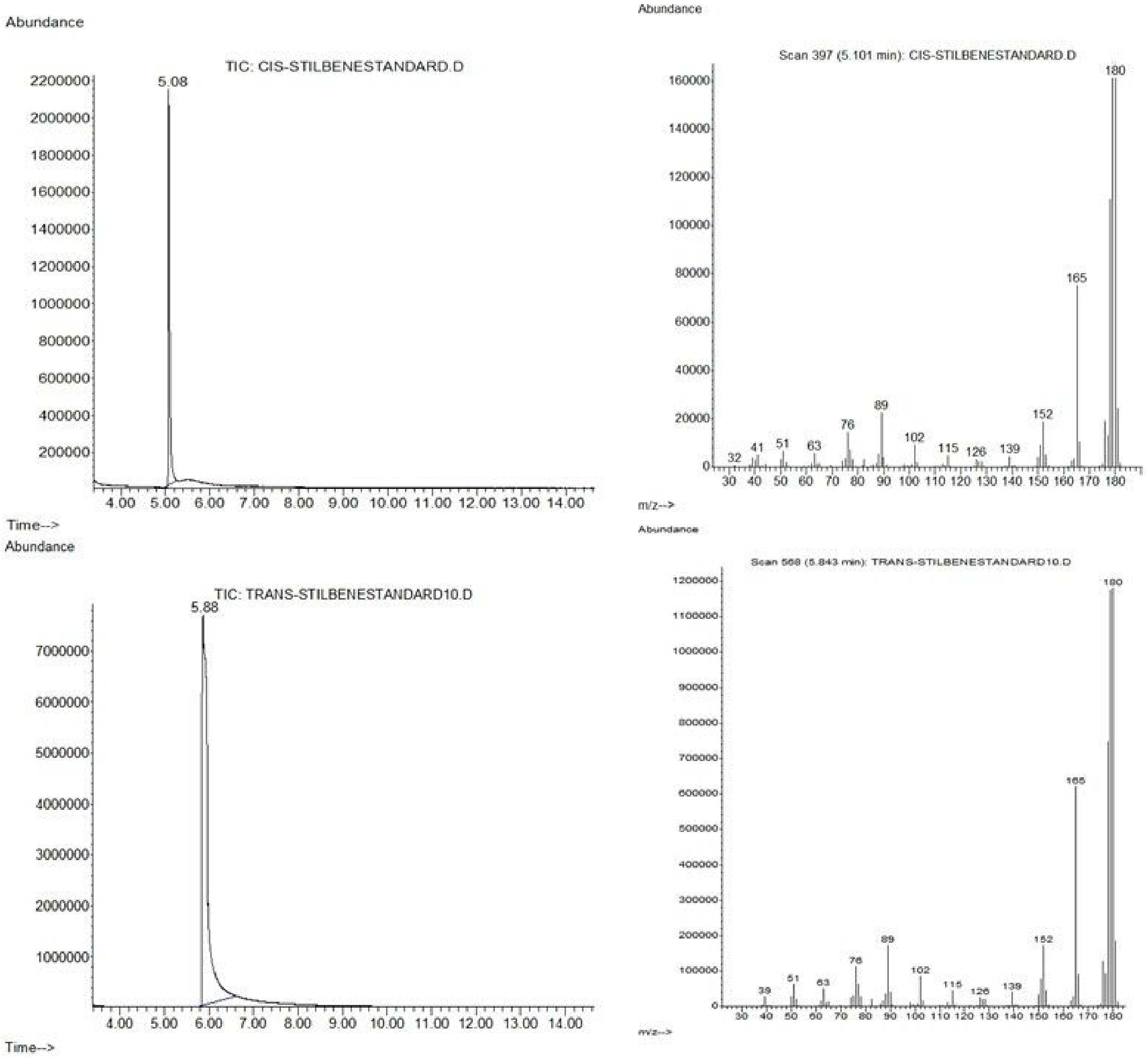

Figure S5. GC-MS analysis of reference standards of (*Z*)-stilbene (top left) and (*E*)-stilbene (bottom left) in MeCN, along with the corresponding mass spectra (top and bottom right, respectively).

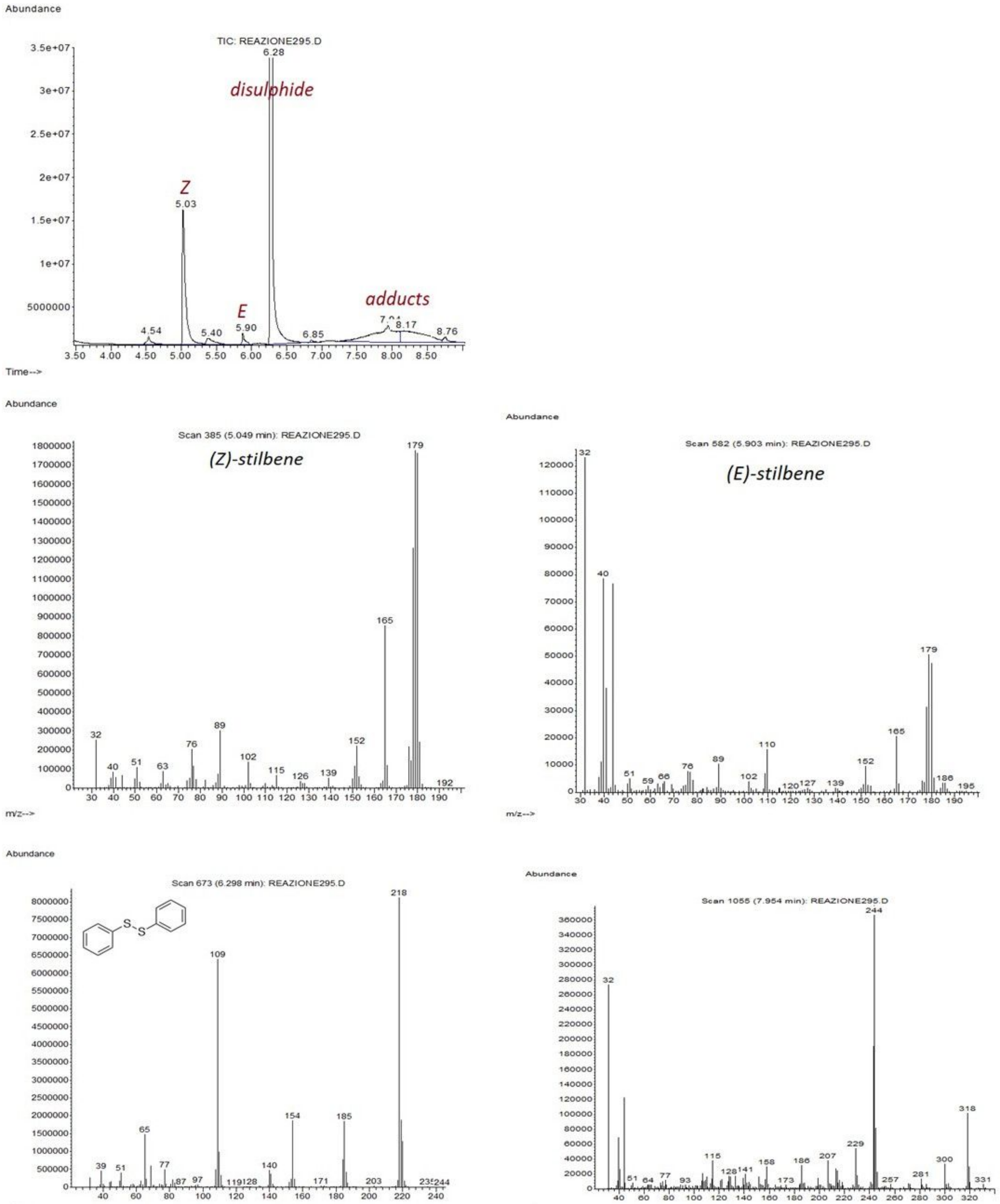

Figure S6. (Top) GC-MS analysis of the reaction mixture of 1 mM PhSH, 0.1 mM (*Z*)-stilbene and 1 mM 4-methylbenzoquinone (4-MBQ) in MeCN after 1 hour at 30°C showing isomerization of (*Z*)-stilbene to (*E*)-stilbene (r.t. 5.90 min), formation of phenyldisulphide (r.t. 6.28 min) and of unresolved quinone adducts and dimers; and (middle and bottom) the corresponding mass spectra.

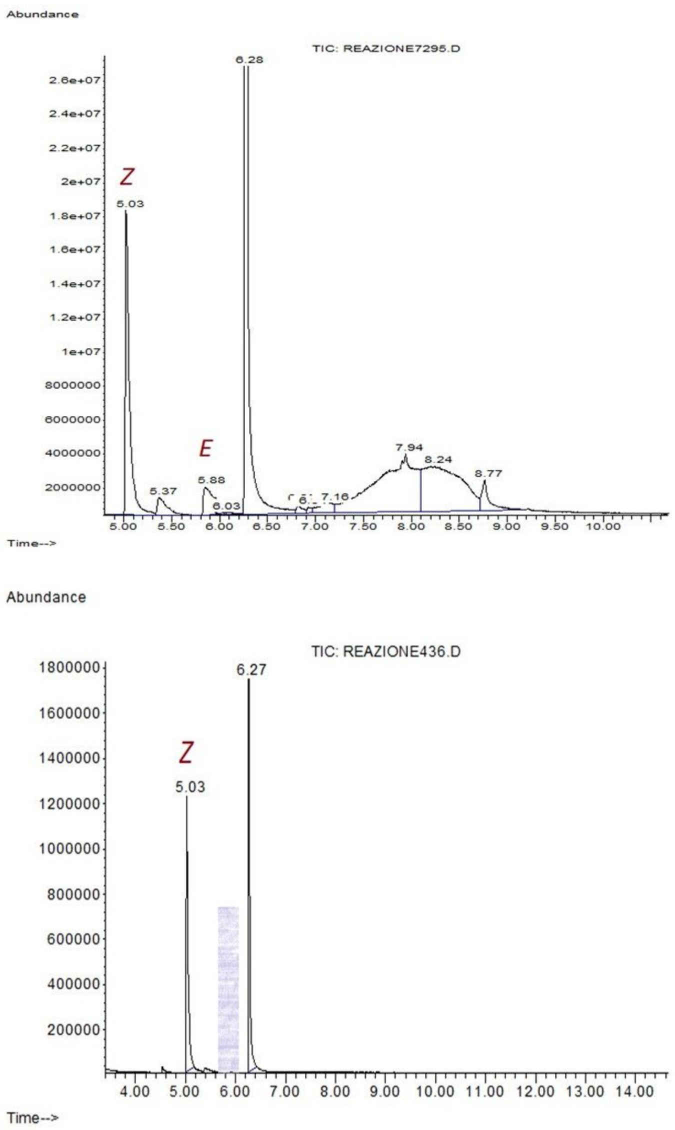

Figure S7. (Top) GC-MS analysis of the reaction mixture of 10 mM PhSH, 0.1 mM (*Z*)-stilbene and 1 mM 4-methylbenzoquinone (4-MBQ) in MeCN after 1 hour at 30°C showing isomerization of (*Z*)-stilbene to (*E*)-stilbene (r.t. 5.88 min), and (bottom) parallel experiment without 4-MBQ showing no isomerization (*E*-isomer would fall in the shaded violet area).

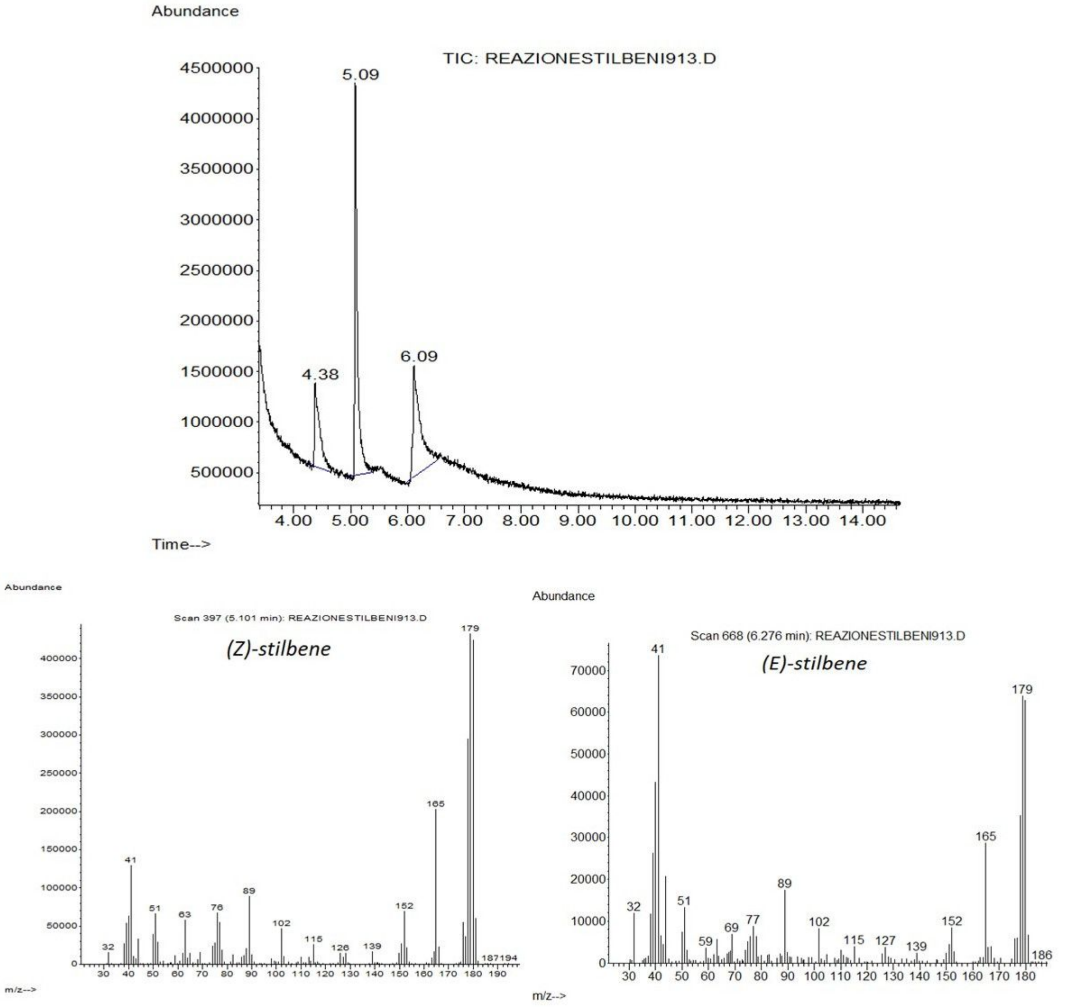

Figure S8. (Top) GC-MS analysis of the reaction mixture of 0.2 mM PhSH, 0.1 mM (*Z*)-stilbene and 5 mM AIBN in MeCN after 1 hour incubation at 60°C showing thiyl radical promoted isomerization from (*Z*)-stilbene (r.t. 5.09 min) to (*E*)-stilbene (r.t. 6.09 min) and (bottom) the mass spectra proving the formation of (*E*)-stilbene (bottom right) from the *Z* isomer (left).

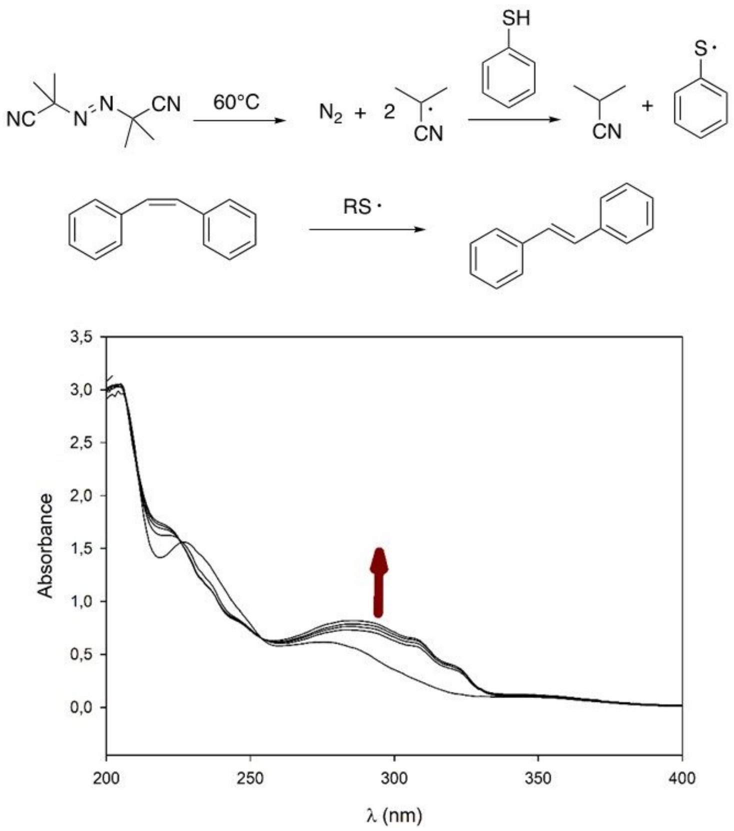

Figure S9. Time evolution of the UV spectrum of a solution of 0.05 mM (*Z*)-stilbene, 0.1 mM PhSH and 7 mM AIBN in MeCN at 60°C showing the $Z \rightarrow E$ isomerization of stilbene promoted by thiyl radicals.

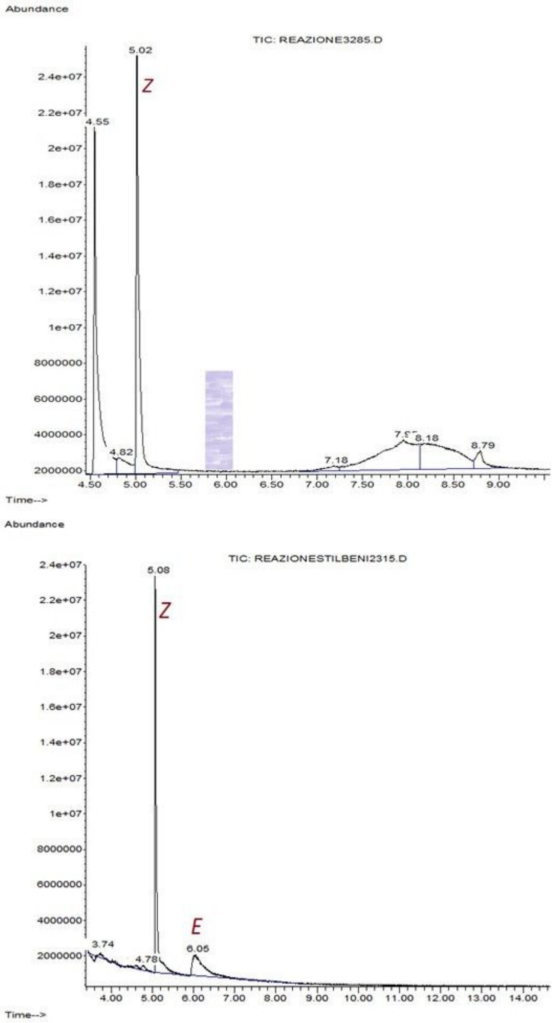

Figure S10. (Top) GC-MS analysis of the reaction mixture of 1 mM HSEtOH, 0.1 mM (*Z*)-stilbene and 1 mM 4-methylbenzoquinone (4-MBQ) in MeCN after 1 hour at 30°C showing no isomerization of (*Z*)-stilbene to (*E*)-stilbene (*E*-isomer would fall in the shaded violet area), and (bottom) parallel experiment where 4-MBQ was replaced by 10 mM *tert*-butyl peroxybenzoate (TBPB) and incubated at 60°C, showing formation of (*E*)-stilbene at r.t. 6.05 min.

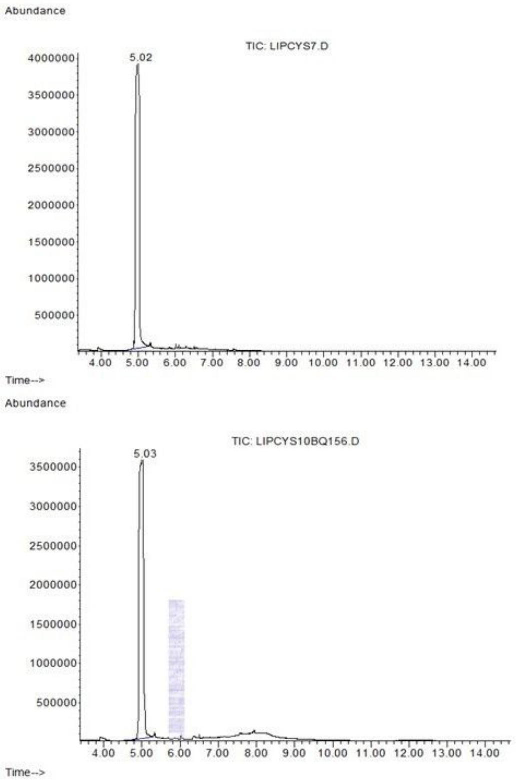

Figure S11. (Top) GC-MS analysis of 7.6 mM LipCys in MeCN and (bottom) of the reaction mixture of 10 mM LipCys, 0.1 mM (*Z*)-stilbene and 1 mM 4-MBQ in MeCN after 1 hour at 30°C showing no isomerization of (*Z*)-stilbene to (*E*)-stilbene (*E*-isomer would fall in the shaded violet area). The retention time of LipCys is identical to (*Z*)-stilbene, therefore they are coeluted at 5.03 min.

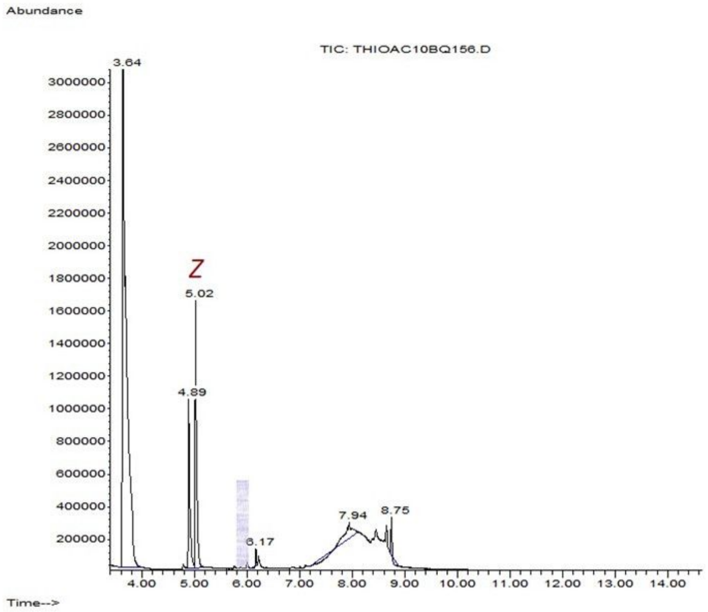

Figure S12. GC-MS analysis of the reaction mixture of 10 mM thioacetic acid, 0.1 mM (*Z*)-stilbene and 1 mM 4-MBQ in MeCN after 1 hour at 30°C showing no isomerization of (*Z*)-stilbene to (*E*)-stilbene (*E*-isomer would fall in the shaded violet area).

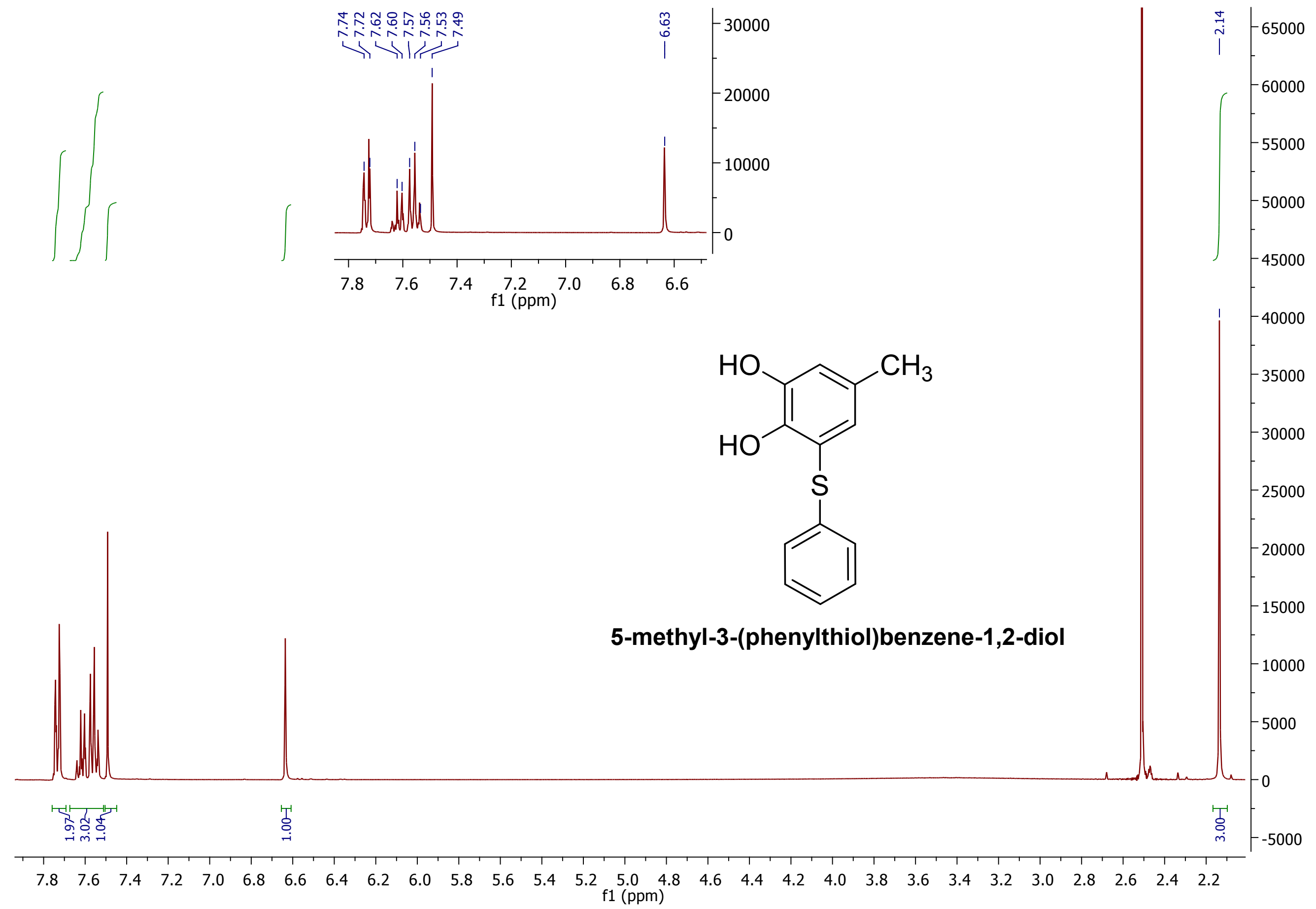

Figure S13. ^{1}H NMR spectrum of 5-methyl-3-(phenylthiol)benzene-1,2-diol (400 MHz, DMSO-d_6).

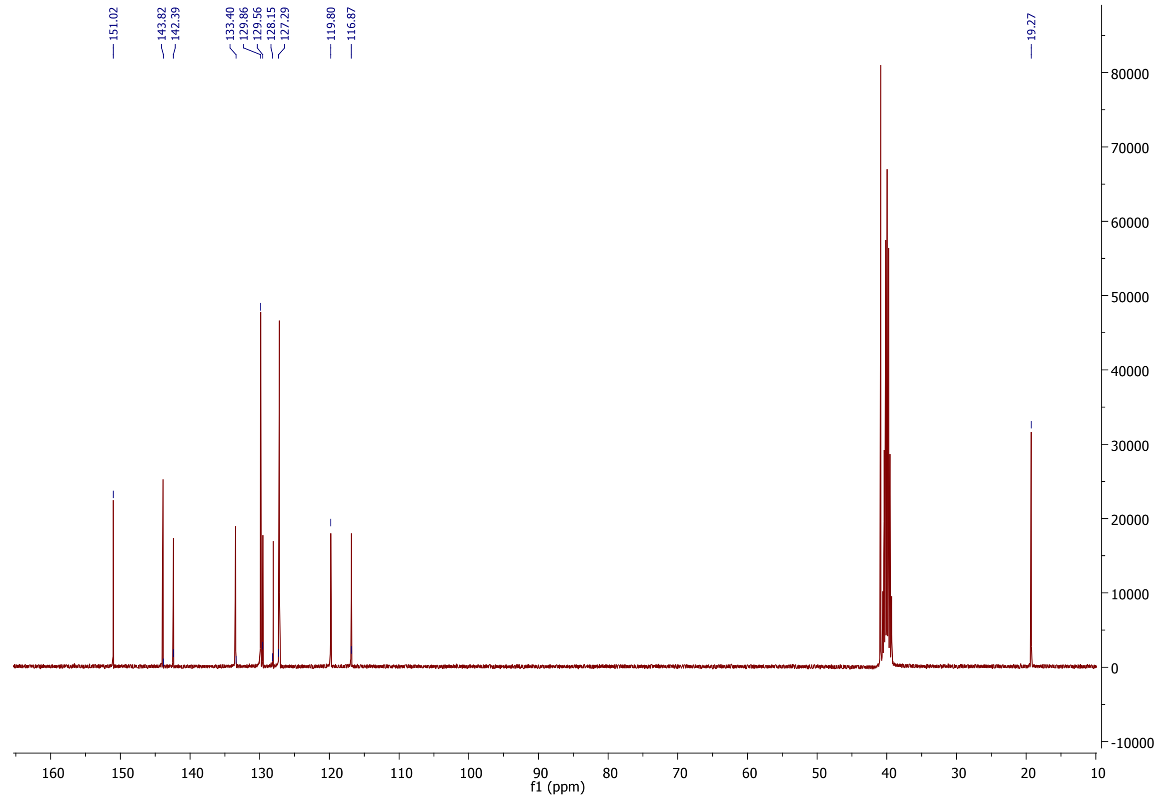

Figure S14. $^{13}C\{^{1}H\}$ NMR spectrum of 5-methyl-3-(phenylthiol)benzene-1,2-diol (400 MHz, DMSO-d6).

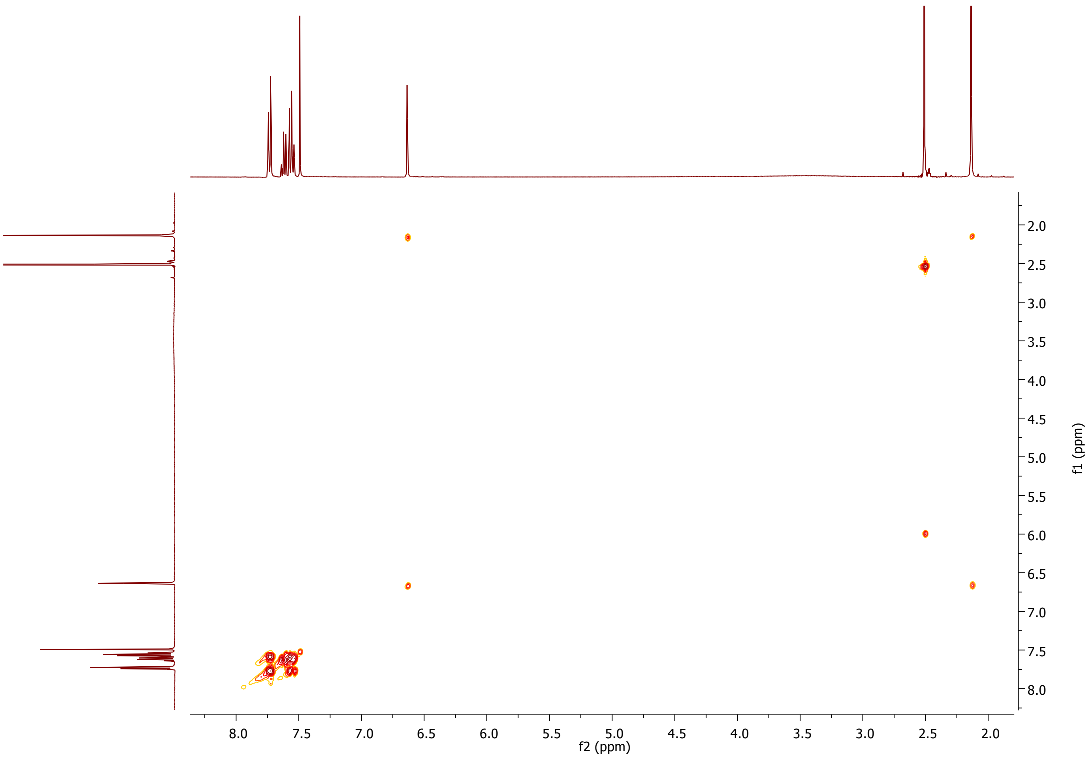

Figure S15. $^{1}H,^{1}H$ COSY spectrum of 5-methyl-3-(phenylthiol)benzene-1,2-diol (400 MHz, DMSO-d6).

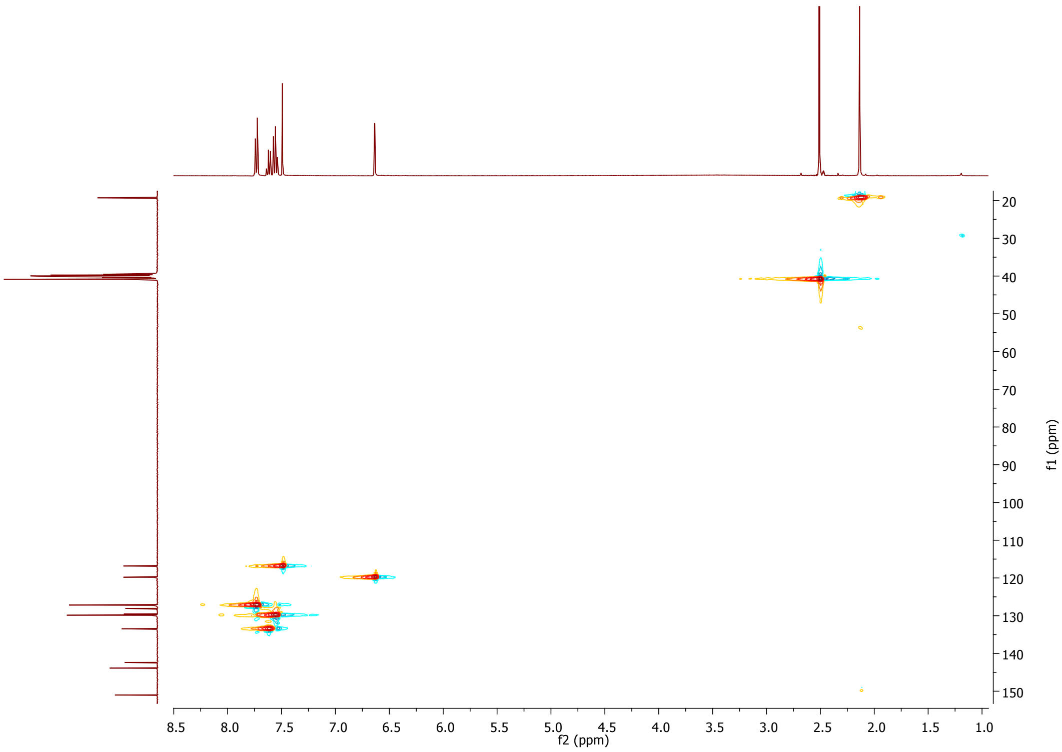

Figure S16. $^{1}H,^{13}C$ HSQC spectrum of 5-methyl-3-(phenylthiol)benzene-1,2-diol (400 MHz, DMSO-d6).

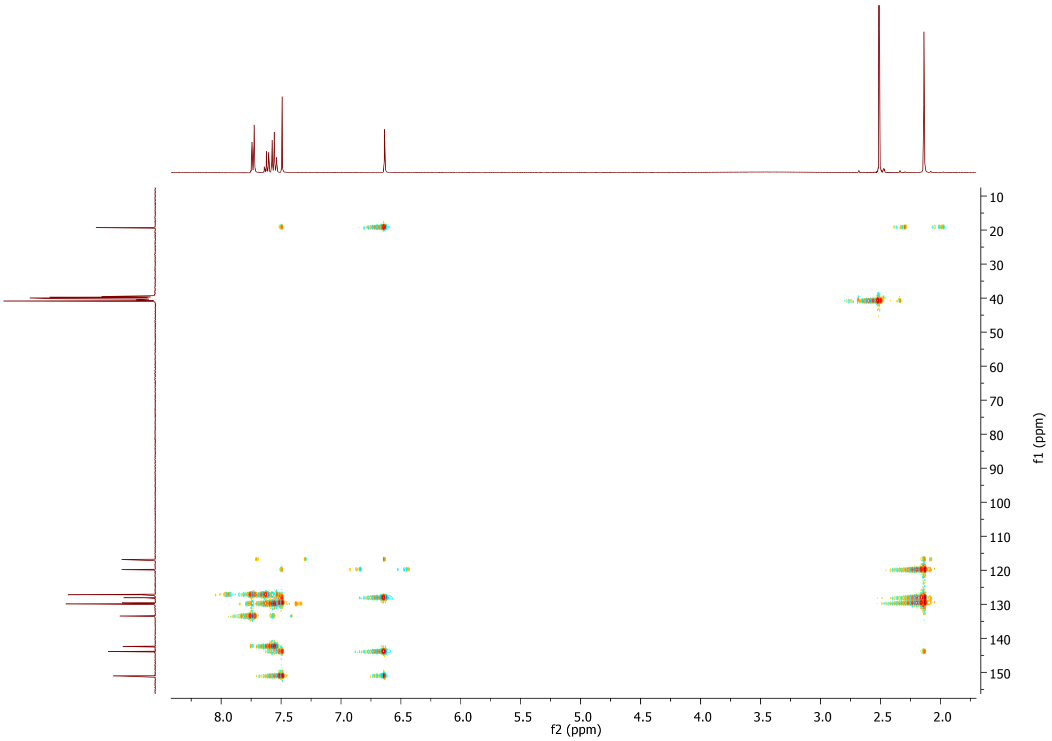

Figure S17. $^{1}H,^{13}C$ HMBC spectrum of 5-methyl-3-(phenylthiol)benzene-1,2-diol (400 MHz, DMSO-d6).

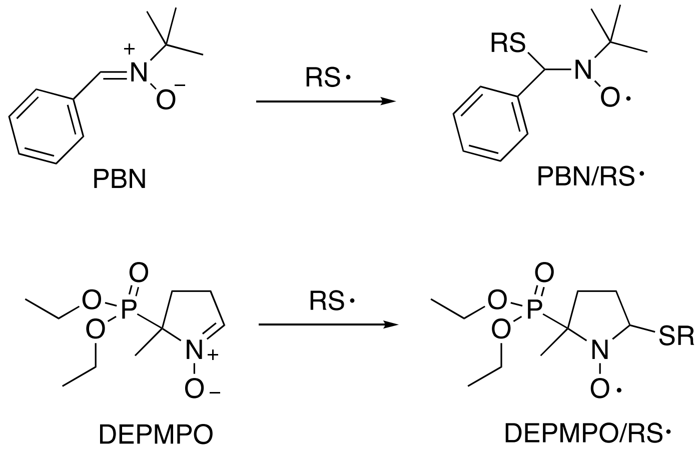

Scheme S1. Spin trapping of thiyl radicals.

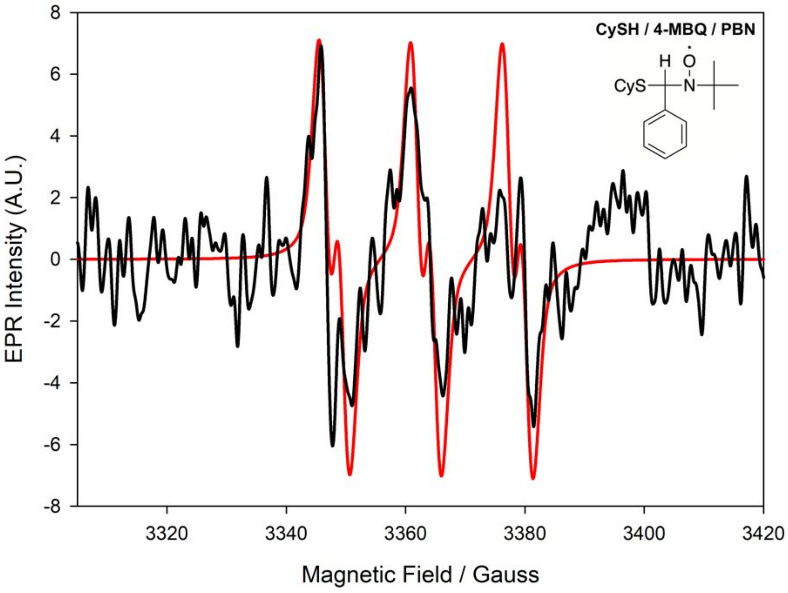

Figure S18. Spin adduct obtained by incubating 85 mM PBN, 16 mM CySH, 16 mM 4-MBQ in 1:1 MeCN/acetate buffer (pH = 5). Spectrum (black) was recorded with the following settings: modulation amplitude 1 G, sweep time 60 s, microwave power 10 mW, frequency 9.76 GHz (*g* = 2.0068), while its computer simulation (red) was obtained using the following parameters: a_N = 15.1 G, a_H = 3.0 G.

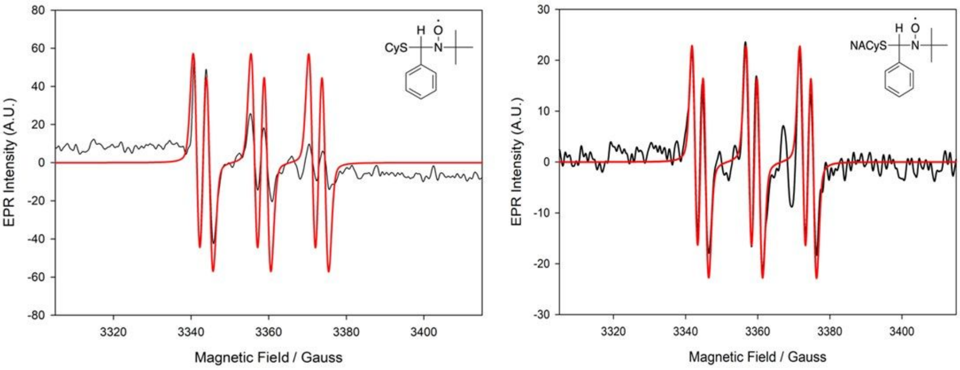

Figure S19. Spin trapping of the thiyl radical from CySH and NACys upon photolyzing in the EPR cavity a solution of the corresponding nitroso CyS-NO and NAC-NO (25 mM) in the presence of PBN (6 mM) in MeCN/acetate buffer (pH = 5.0) 7:3 at 30°C. Photolysis was performed at 240-400 nm (2200-3000 mW/cm^2) for 40 s. Experimental spectra (black) were recorded with the following settings: modulation amplitude 1 G, sweep time 60 s, microwave power 5 mW, frequency 9.76 GHz (g = 2.0068), while their computer simulations (red) were obtained using the following parameters: (CyS-PBN) a_N = 15.0 G, a_H = 3.2 G; (NACyS-PBN) a_N = 14.9 G, a_H = 2.9 G.

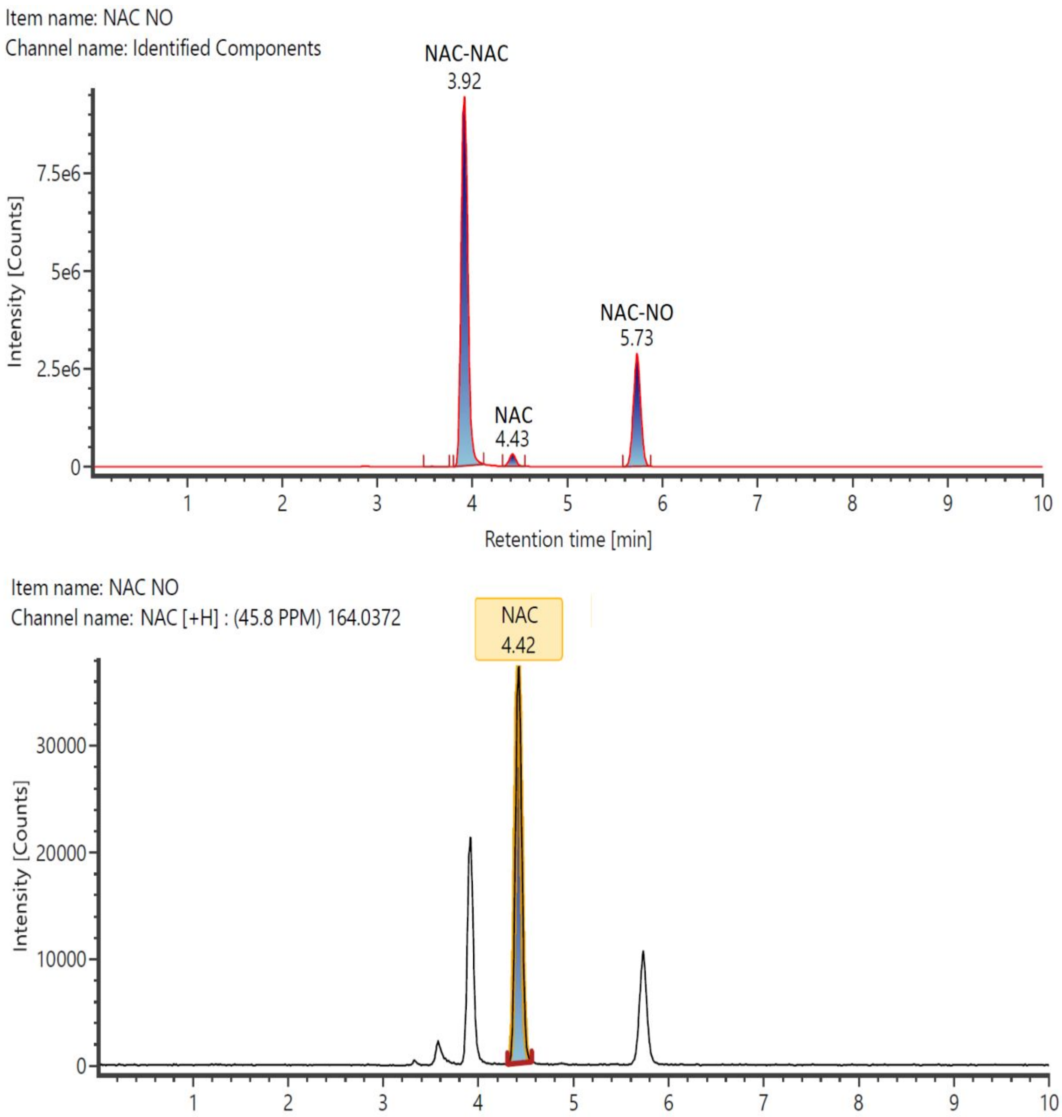

Figure S20. (Top) UPLC-Q-TOF chromatogram of S-Nitroso-N-acetylcysteine NAC-NO (r.t. 5.73 min), in acetate buffer/MeCN (pH 5.0), showing residues of the starting N-acetylcysteine NAC (r.t. 4.43 min) and the disulphide dimer NAN-NAC (r.t. 3.92 min) originated from the synthesis. (Bottom) Chromatogram built at *m/z* 164 corresponding to $[M+H]^+$ ion of NAC, proving the relevant impurity of the starting thiol in the synthesized NACNO. Mass spectra are reported in Figure S21.

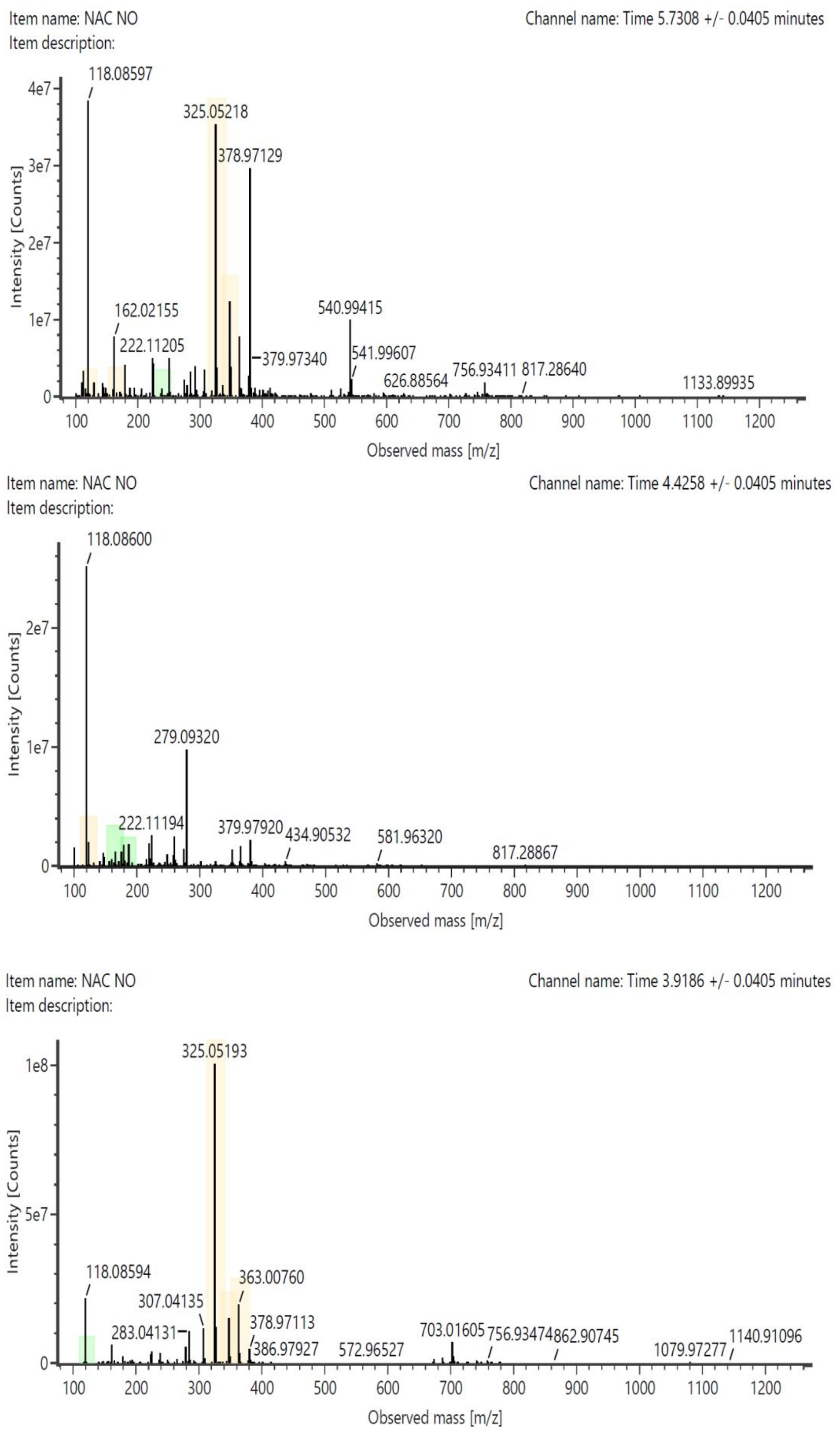

Figure S21. Q-TOF mass spectra of the components identified in Figure S20: (top) S-Nitroso-N-acetylcysteine NAC-NO, (middle) N-acetylcysteine, and (bottom) the corresponding disulphide.

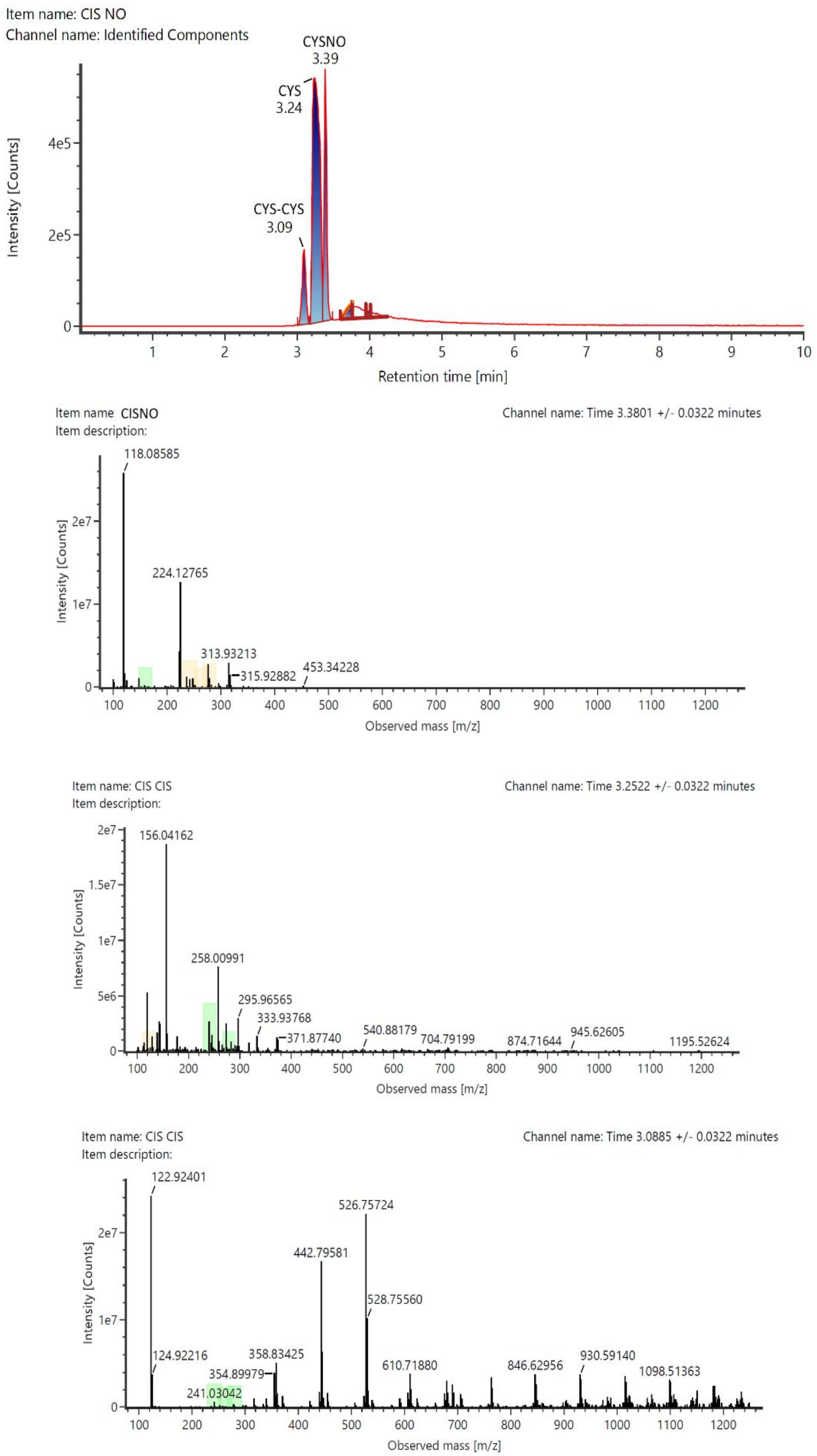

Figure S22. UPLC-Q-TOF chromatogram of S-Nitrosocysteine (CYS-NO) (r.t. 3.39 min), in acetate buffer/MeCN (pH 5.0), showing residues of the starting cysteine (CYS) (r.t. 3.24 min) and the disulphide dimer (CYS-CYS) (r.t. 3.09 min) originated from the synthesis. The corresponding mass spectra are shown below the chromatogram.

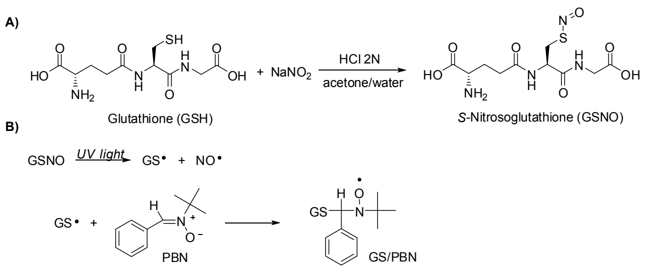

Scheme S2. A) Synthesis of S-nitrosoglutathione; B) reaction scheme of spin-trapping experiments by photolyzing the nitrosoglutathione in the cavity of the EPR spectrometer in the presence of PBN.

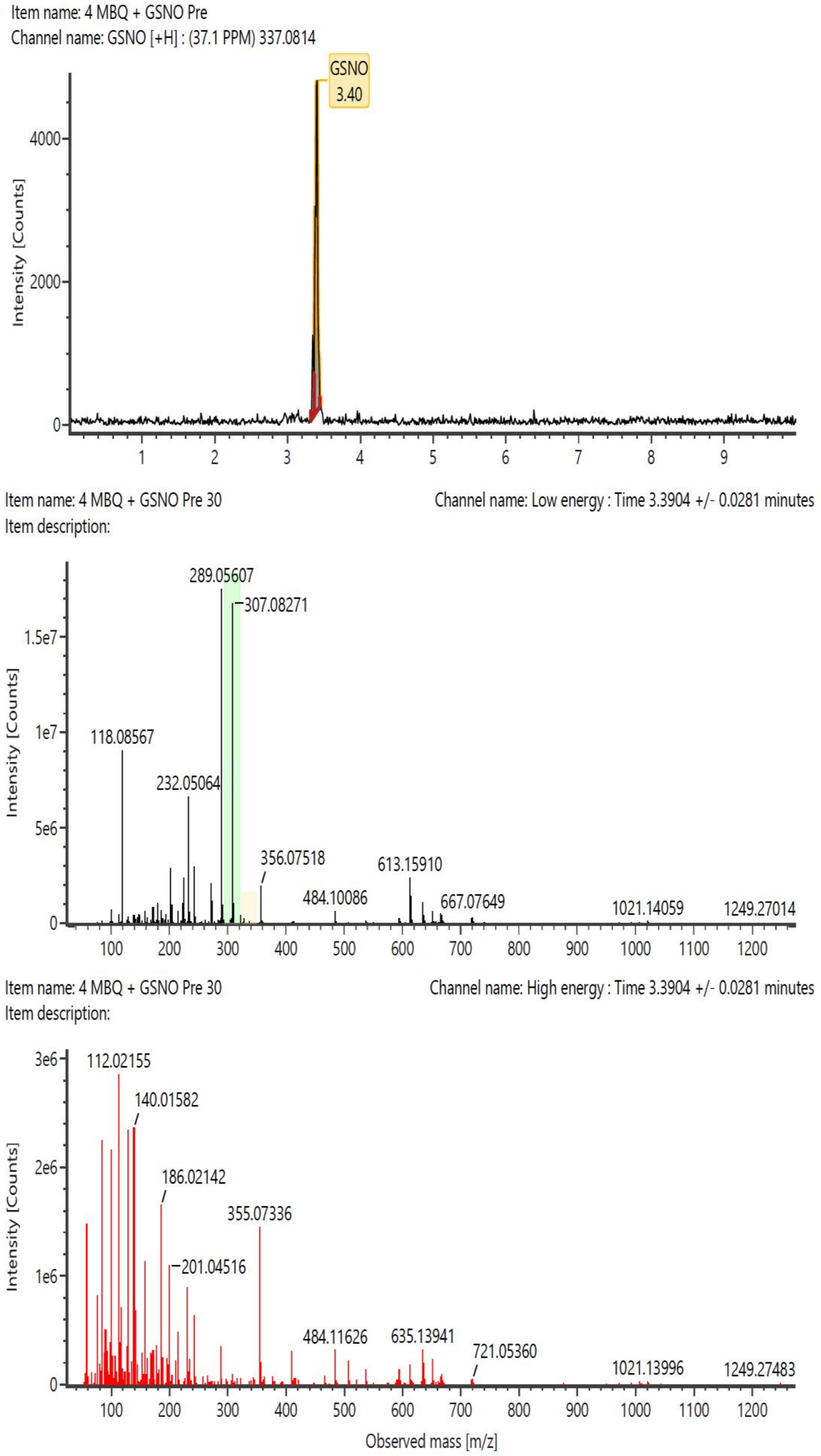

Figure S23. (Top) UPLC-Q-TOF chromatogram of *S*-Nitrosoglutathione (GS-NO) $[M+H]^+$, in acetate buffer/MeCN (pH 5.0), before reaction with quinone, (middle) corresponding MS spectrum and (bottom) MS/MS fragmentation pattern of characteristic ion at *m/z* 307.

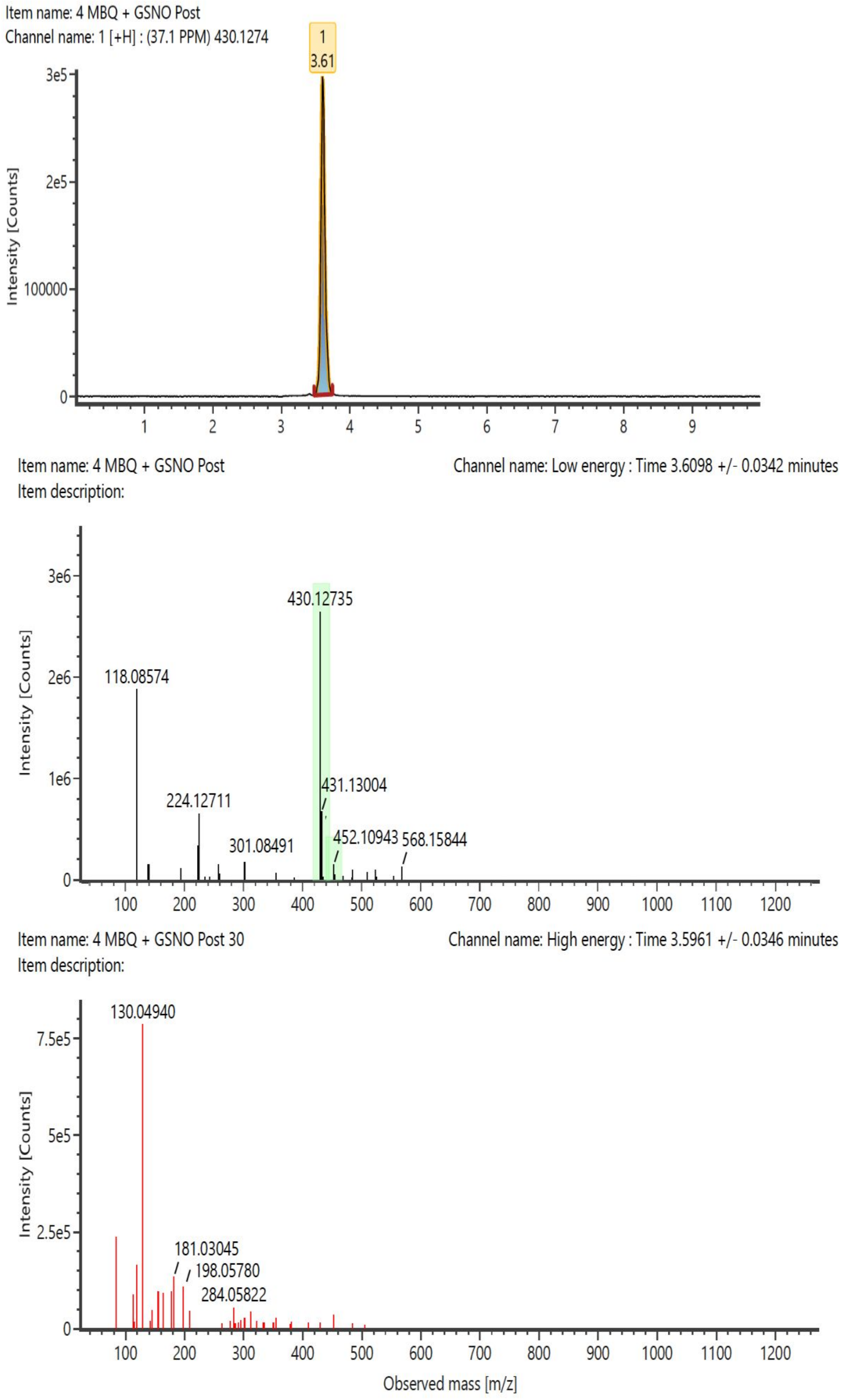

Figure S24. (Top) UPLC-Q-TOF chromatogram of the adduct obtained by irradiating a solution of 1 mM 4-MBQ and 1 mM GS-NO in acetate buffer/MeCN (pH 5.0), with a Hamamatsu UV - lamp (4500 mW/cm^2 at 240-400 nm, power 50%) for 5 minutes at 30°C, (middle) corresponding MS spectrum and (bottom) MS/MS fragmentation pattern of characteristic ion at *m/z* 430.

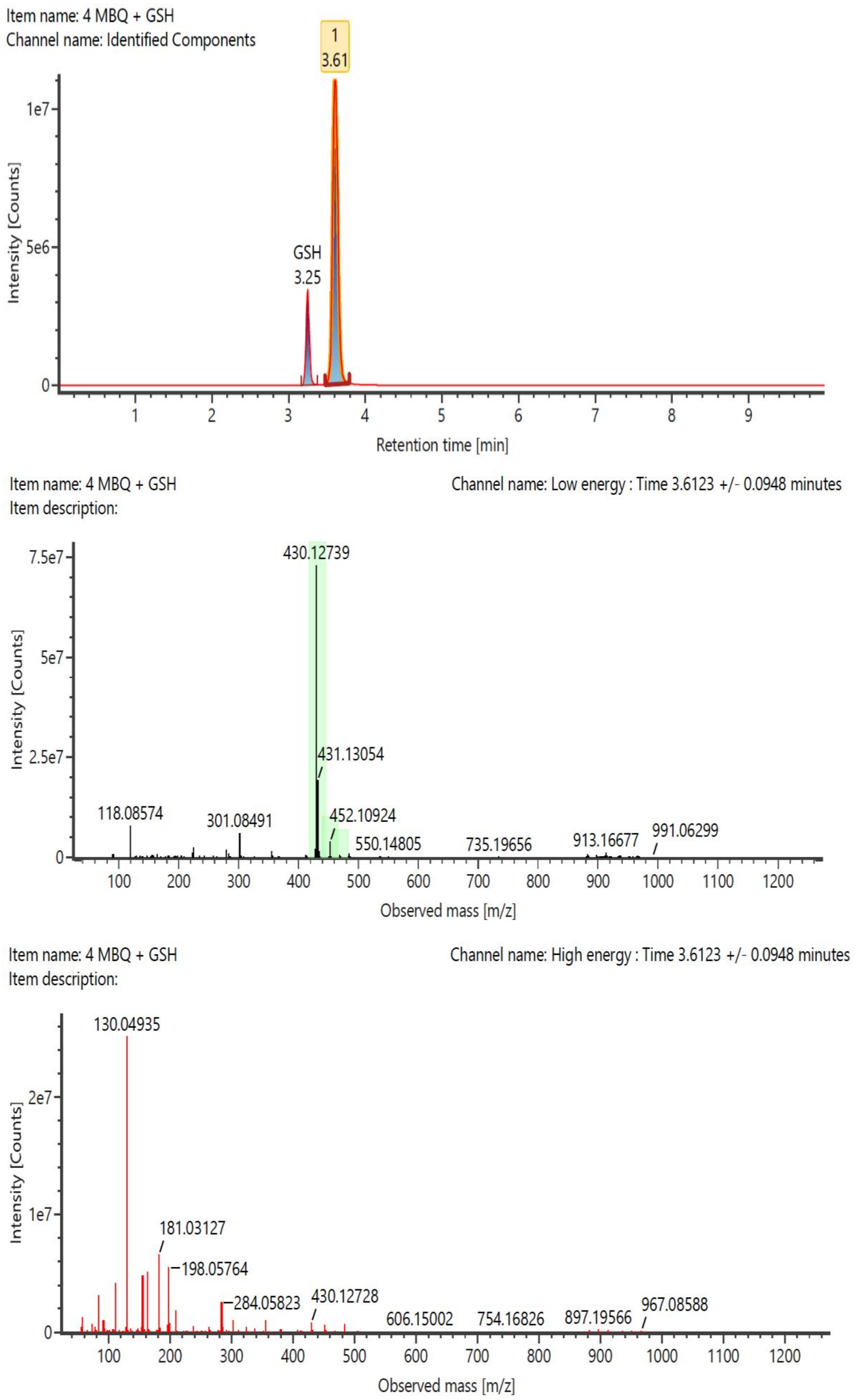

Figure S25. (Top) UPLC-Q-TOF chromatogram of the adduct obtained by mixing 1 mM 4-MBQ and 1 mM glutathione (GSH) in acetate buffer/MeCN (pH 5.0), and incubation at 30°C for 15 min, (middle) corresponding MS spectrum and (bottom) MS/MS fragmentation pattern of characteristic ion at *m/z* 430.

Computational studies

Table legend

Energy data are reported in hartrees; in parentheses, relative values (kcal mol^{-1}) referred to the most stable form identified at the specified level.

Structure: Isomer / tautomer / conformer.

In vacuo:
E: Electronic energy, PBE0 / 6-31+G(d,p) level (ωB97X-D / 6-31+G(d,p) level, respectively).
H_{RRHO}: Sum of electronic and thermal enthalpies, PBE0 / 6-31+G(d,p) level (ωB97X-D / 6-31+G(d,p) level, respectively).
G_{RRHO}: Sum of electronic and thermal free energies, PBE0 / 6-31+G(d,p) level (ωB97X-D / 6-31+G(d,p) level, respectively).
$E_{M06\text{-}2X,large}$: Electronic energy, M06-2X / 6-311++G(2d,2p) // PBE0 / 6-31+G(d,p) level.
$E_{\omega B97X\text{-}D,large:}$ Electronic energy, ωB97X-D / 6-311++G(2d,2p) // ωB97X-D / 6-31+G(d,p) level.
$G_{RRHO,M06\text{-}2X,large}$: $E_{M06\text{-}2X,large} + G_{RRHO} – E$.
$G_{RRHO,\omega B97X\text{-}D,large}$: $E_{\omega B97X\text{-}D,large} + G_{RRHO} – E$.
$E_{(RO)CCSD(T)/cc\text{-}pVTZ}$: Electronic energy, (RO)CCSD(T) / cc-pVTZ // PBE0 / 6-31+G(d,p) level.
$G_{RRHO,(RO)CCSD(T)/cc\text{-}pVTZ}$: $E_{(RO)CCSD(T)/cc\text{-}pVTZ} + G_{RRHO} – E$.
$E_{(RO)CCSD(T)/jun\text{-}cc\text{-}pVTZ}$: Electronic energy, (RO)CCSD(T) / jun-cc-pVTZ // PBE0 / 6-31+G(d,p) level.
$G_{RRHO,(RO)CCSD(T)/jun\text{-}cc\text{-}pVTZ}$: $E_{(RO)CCSD(T)/jun\text{-}cc\text{-}pVTZ} + G_{RRHO} – E$.

In water at the PBE0 / 6-31+G(d,p), PCM geometries (ωB97X-D / 6-31+G(d,p), PCM geometries, respectively):
G_{PCM}: Electronic energy, PBE0 / 6-31+G(d,p), PCM level (ωB97X-D / 6-31+G(d,p), PCM level, respectively), including electrostatic PCM contribution.
$H_{PCM,RRHO}$: Sum of electronic and thermal enthalpies, PBE0 / 6-31+G(d,p), PCM level (ωB97X-D / 6-31+G(d,p), PCM level, respectively), including electrostatic PCM contributions.
$G_{PCM,RRHO}$: Sum of electronic and thermal free energies, PBE0 / 6-31+G(d,p), PCM level (ωB97X-D / 6-31+G(d,p), PCM level, respectively), including electrostatic PCM contributions.
$G_{SMD,M06\text{-}2X,large}$: Electronic energy, M06-2X / 6-311++G(2d,2p), SMD // PBE0 / 6-31+G(d,p), PCM level, including electrostatic and non-electrostatic PCM contributions.
$G_{SMD,\omega B97X\text{-}D,large}$: Electronic energy, ωB97X-D / 6-311++G(2d,2p), SMD // PBE0 / 6-31+G(d,p), PCM level, including electrostatic and non-electrostatic PCM contributions.
$G_{SMD,RRHO,M06\text{-}2X,large}$: $G_{SMD,M06\text{-}2X,large} + G_{PCM,RRHO} – G_{PCM}$.
$G_{SMD,RRHO,\omega B97X\text{-}D,large}$: $G_{SMD,\omega B97X\text{-}D,large} + G_{PCM,RRHO} – G_{PCM}$.

In water at the M06-2X / 6-311++G(2d,2p), SMD geometries:
$G_{SMD,M06\text{-}2X,large}$: Electronic energy, M06-2X / 6-311++G(2d,2p), SMD level, including electrostatic and non-electrostatic PCM contributions.
$H_{SMD,RRHO,M06\text{-}2X,large}$: Sum of electronic and thermal enthalpies, M06-2X / 6-311++G(2d,2p), SMD level, including electrostatic and non-electrostatic PCM contributions.
$G_{SMD,RRHO,M06\text{-}2X,large}$: Sum of electronic and thermal free energies, M06-2X / 6-311++G(2d,2p), SMD level, including electrostatic and non-electrostatic PCM contributions.
$E_{vacuo,DLPNO\text{-}CCSD(T)\text{-}F12/cc\text{-}pVTZ\text{-}F12}$: Electronic energy, DLPNO-CCSD(T)-F12 / cc-pVTZ-F12, vacuo // M06-2X / 6-311++G(2d,2p), SMD level.
$E_{vacuo,M06\text{-}2X,large}$: Electronic energy, M06-2X / 6-311++G(2d,2p), vacuo // M06-2X / 6-311++G(2d,2p), SMD level.
$G_{SMD,RRHO,DLPNO\text{-}CCSD(T)\text{-}F12/cc\text{-}pVTZ\text{-}F12}$: $E_{vacuo,DLPNO\text{-}CCSD(T)\text{-}F12/cc\text{-}pVTZ\text{-}F12} + G_{SMD,RRHO,M06\text{-}2X,large} – E_{vacuo,M06\text{-}2X,large}$.

Part 1: Methanethiol

Table S1.1. Reduced neutral form in vacuo.

Structure	Schematic drawing	Symmetry	E	H_{RRHO}	G_{RRHO}	$E_{M06-2X,large}$	$G_{RRHO,M06-2X,large}$
conf1	H_3C—SH<br>methanethiol<br><br>S -0.04779 -0.66550 0.00000<br>C -0.04779 1.15178 0.00000<br>H 0.43016 1.55484 0.89502<br>H -1.09564 1.45877 0.00000<br>H 0.43016 1.55484 -0.89502<br>H 1.28673 -0.83116 0.00000	C_s	-438.49480 (0.0)	-438.44375 (0.0)	-438.47242 (0.0)	-438.68119 (0.0)	-438.65881 (0.0)

Table S1.2. Reduced neutral form in water.

Structure	Schematic drawing	Symmetry	G_{PCM}	$H_{PCM,RRHO}$	$G_{PCM,RRHO}$	$G_{SMD,M06-2X,large}$	$G_{SMD,RRHO,M06-2X,large}$
conf1	H_3C—SH<br>methanethiol<br><br>S -0.04823 -0.66494 0.00000<br>C -0.04823 1.15384 0.00000<br>H 0.43606 1.54836 0.89444<br>H -1.09487 1.46342 0.00000<br>H 0.43606 1.54836 -0.89444<br>H 1.28378 -0.84422 0.00000	C_s	-438.49893 (0.0)	-438.44791 (0.0)	-438.47661 (0.0)	-438.68286 (0.0)	-438.66054 (0.0)

Table S1.3. Reduced anionic form in water.

Structure	Schematic drawing	Symmetry	G_{PCM}	$H_{PCM,RRHO}$	$G_{PCM,RRHO}$	$G_{SMD,M06-2X,large}$	$G_{SMD,RRHO,M06-2X,large}$
conf1	H_3C—S^-<br>methanethiolate<br><br>S 0.00000 0.00000 0.70966<br>C 0.00000 0.00000 -1.12826<br>H 0.00000 1.01981 -1.52832<br>H -0.88318 -0.50990 -1.52832<br>H 0.88318 -0.50990 -1.52832	C_{3v}	-438.02560 (0.0)	-437.98474 (0.0)	-438.01130 (0.0)	-438.20346 (0.0)	-438.18916 (0.0)

Table S1.4. One-electron oxidized neutral form in vacuo.

Structure	Schematic drawing	Symmetry	E	H_{RRHO}	G_{RRHO}	$E_{M06\text{-}2X,large}$	$G_{RRHO,M06\text{-}2X,large}$
conf1	$H_3C—S^\bullet$<br>methylsulfanyl	C_{3v}	lowers symmetry				
conf2	S -0.00461 -0.69099 0.00000<br>C -0.00461 1.10246 0.00000<br>H 1.04700 1.41988 0.00000<br>H -0.47281 1.51063 0.89921<br>H -0.47281 1.51063 -0.89921	C_s	-437.85537 (0.0)	-437.81536 (0.0)	-437.84375 (0.0)	-438.03925 (0.0)	-438.02763 (0.0)

Table S1.5. One-electron oxidized neutral form in water.

Structure	Schematic drawing	Symmetry	G_{PCM}	$H_{PCM,RRHO}$	$G_{PCM,RRHO}$	$G_{SMD,M06\text{-}2X,large}$	$G_{SMD,RRHO,M06\text{-}2X,large}$
conf1	$H_3C—S^\bullet$<br>methylsulfanyl	C_{3v}	lowers symmetry				
conf2	S -0.00454 -0.68998 0.00000<br>C -0.00454 1.10177 0.00000<br>H 1.04773 1.41730 0.00000<br>H -0.47390 1.50591 0.89962<br>H -0.47390 1.50591 -0.89962	C_s	-437.85866 (0.0)	-437.81874 (0.0)	-437.84713 (0.0)	-438.04155 (0.0)	-438.03001 (0.0)

Part 2: Thioacetic acid

Table S2.1. Reduced neutral form in vacuo.

Structure	Schematic drawing	Symmetry	E	H_{RRHO}	G_{RRHO}	$E_{M06\text{-}2X,large}$	$G_{RRHO,M06\text{-}2X,large}$
conf1	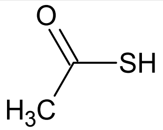<br>ethanethioic *S*-acid<br><br>C 1.48393 0.77299 0.00000<br>H 1.95112 0.33051 0.88519<br>H 1.64409 1.85227 0.00000<br>H 1.95112 0.33051 -0.88519<br>C 0.00000 0.50746 0.00000<br>O -0.83905 1.37170 0.00000<br>S -0.52728 -1.21274 0.00000<br>H 0.69902 -1.76571 0.00000	C_s	-551.71542 (1.9)	-551.65295 (1.9)	-551.68723 (2.3)	-552.01387 (1.0)	-551.98567 (1.4)
conf2	C -1.39802 -0.93490 0.00026<br>H -1.05618 -1.73949 -0.65601<br>H -2.38655 -0.58846 -0.30503<br>H -1.45835 -1.33336 1.01886<br>C -0.44649 0.23122 -0.01020<br>O -0.78782 1.38740 -0.00083<br>S 1.28287 -0.26334 -0.00269<br>H 1.74479 0.99774 0.05146	C_1	-551.71848 (0.0)	-551.65593 (0.0)	-551.69088 (0.0)	-552.01548 (0.0)	-551.98788 (0.0)
conf3	C 1.47227 0.81378 0.00000<br>H 1.95051 0.37933 0.88367<br>H 1.60667 1.89604 0.00000<br>H 1.95051 0.37933 -0.88367<br>C 0.00000 0.50390 0.00000<br>O -0.87234 1.33626 0.00000<br>S -0.35469 -1.26016 0.00000<br>H -1.68762 -1.08839 0.00000	C_s	-551.71847 (0.0)	saddle point			

Table S2.2. Reduced neutral form in water.

Structure	Schematic drawing	Symmetry	G_{PCM}	$H_{PCM,RRHO}$	$G_{PCM,RRHO}$	$G_{SMD,M06\text{-}2X,large}$	$G_{SMD,RRHO,M06\text{-}2X,large}$
conf1	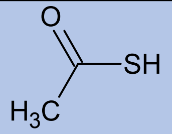<br>ethanethioic *S*-acid<br><br>C 1.47348 0.78969 0.00000<br>H 1.94536 0.34982 0.88353<br>H 1.62532 1.86998 0.00000<br>H 1.94536 0.34982 -0.88353<br>C 0.00000 0.50322 0.00000<br>O -0.85985 1.35781 0.00000<br>S -0.51189 -1.21382 0.00000<br>H 0.71212 -1.76854 0.00000	C_s	-551.72397 (0.5)	-551.66156 (0.5)	-551.69565 (0.9)	-552.02128 (0.0)	-551.99297 (0.2)
conf2	C -1.39856 -0.93096 0.00027<br>H -1.01031 -1.78850 -0.55353<br>H -2.35597 -0.61185 -0.41489<br>H -1.55231 -1.24080 1.03996<br>C -0.44300 0.22657 -0.01190<br>O -0.78291 1.38959 -0.00121<br>S 1.27938 -0.26486 -0.00348<br>H 1.76108 0.98846 0.06361	C_1	-551.72474 (0.0)	-551.66239 (0.0)	-551.69706 (0.0)	-552.02092 (0.2)	-551.99325 (0.0)
conf3	C 1.46557 0.82356 0.00000<br>H 1.94644 0.39147 0.88281<br>H 1.59680 1.90616 0.00000<br>H 1.94644 0.39147 -0.88281<br>C 0.00000 0.50096 0.00000<br>O -0.88457 1.32865 0.00000<br>S -0.34531 -1.25947 0.00000<br>H -1.68150 -1.11397 0.00000	C_s	-551.72470 (0.0)	saddle point			

Table S2.3. Reduced anionic form in water.

Structure	Schematic drawing	Symmetry	G_{PCM}	$H_{PCM,RRHO}$	$G_{PCM,RRHO}$	$G_{SMD,M06\text{-}2X,large}$	$G_{SMD,RRHO,M06\text{-}2X,large}$
conf1	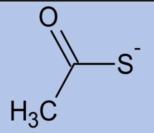<br>ethanethioate<br><br>C 1.50059 0.65896 0.00000<br>H 1.95625 0.19424 0.87957<br>H 1.71577 1.73103 0.00000<br>H 1.95625 0.19424 -0.87957<br>C 0.00000 0.42238 0.00000<br>O -0.75940 1.39876 0.00000<br>S -0.53479 -1.23735 0.00000	C_s	-551.27242 (0.0)	-551.22001 (0.0)	-551.25403 (0.0)	-551.56823 (0.0)	-551.54984 (0.0)
conf2	C -1.49538 0.68501 0.00000<br>H -1.74384 1.28679 0.88153<br>H -2.09704 -0.22364 0.00000<br>H -1.74384 1.28679 -0.88153<br>C 0.00000 0.40972 0.00000<br>O 0.76400 1.38407 0.00000<br>S 0.52781 -1.24943 0.00000	C_s	-551.27205 (0.2)	saddle point			

Table S2.4. One-electron oxidized neutral form in vacuo.

Structure	Schematic drawing	Symmetry	E	H_{RRHO}	G_{RRHO}	$E_{M06\text{-}2X,large}$	$G_{RRHO,M06\text{-}2X,large}$
conf1	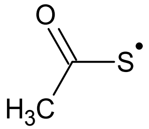<br>acetylsulfanyl<br><br>C -1.42396 -0.87867 -0.00125<br>H -1.21245 -1.61315 -0.78402<br>H -2.41148 -0.43801 -0.15503<br>H -1.40179 -1.39724 0.96297<br>C -0.39348 0.20806 -0.00553<br>O -0.63653 1.39739 0.00131<br>S 1.31391 -0.23169 0.00040	C_1	-551.07614 (0.0)	-551.02364 (0.0)	-551.05941 (0.0)	-551.37008 (0.0)	-551.35335 (0.0)
conf2	C 1.44244 0.84807 0.00000	C_s	-551.07614 (0.0)	saddle point			

	H 1.94405 0.44363 0.88484<br>H 1.51152 1.93807 0.00000<br>H 1.94405 0.44363 -0.88484<br>C 0.00000 0.44561 0.00000<br>O -0.93819 1.21590 0.00000<br>S -0.40930 -1.26967 0.00000						
conf3	C -1.44595 0.83704 0.00000<br>H -1.63948 1.45105 0.88496<br>H -2.11468 -0.02697 0.00000<br>H -1.63948 1.45105 -0.88496<br>C 0.00000 0.43923 0.00000<br>O 0.92470 1.22598 0.00000<br>S 0.41698 -1.27129 0.00000	C_s	-551.07595 (0.1)	saddle point			

Table S2.5. One-electron oxidized neutral form in water.

Structure	Schematic drawing	Symmetry	G_{PCM}	$H_{PCM,RRHO}$	$G_{PCM,RRHO}$	$G_{SMD,M06\text{-}2X,large}$	$G_{SMD,RRHO,M06\text{-}2X,large}$
conf1	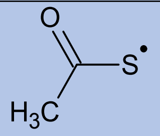<br>acetylsulfanyl<br><br>C -1.41320 -0.88623 -0.00195<br>H -1.15980 -1.65855 -0.73329<br>H -2.39777 -0.46690 -0.21973<br>H -1.42899 -1.35413 0.98819<br>C -0.39585 0.20617 -0.00752<br>O -0.64610 1.39916 0.00178<br>S 1.31311 -0.22708 0.00046	C_1	-551.08213 (0.0)	-551.02978 (0.0)	-551.06506 (0.0)	-551.37618 (0.0)	-551.35912 (0.0)
conf2	C 1.43879 0.84423 0.00000<br>H 1.93860 0.43579 0.88374<br>H 1.51559 1.93347 0.00000<br>H 1.93860 0.43579 -0.88374<br>C 0.00000 0.44734 0.00000<br>O -0.94346 1.21907 0.00000<br>S -0.40486 -1.26919 0.00000	C_s	-551.08212 (0.0)	saddle point			
conf3	C -1.44503 0.82506 0.00000<br>H -1.64387 1.43616 0.88593<br>H -2.10661 -0.04369 0.00000<br>H -1.64387 1.43616 -0.88593	C_s	-551.08197 (0.1)	saddle point			

	C 0.00000 0.44108 0.00000<br>O 0.92502 1.23520 0.00000<br>S 0.41652 -1.26919 0.00000						

Part 3: Thiourea

Table S3.1. Reduced neutral form in vacuo.

Structure	Schematic drawing	Symmetry	E	H_{RRHO}	G_{RRHO}	$E_{M06\text{-}2X,large}$	$G_{RRHO,M06\text{-}2X,large}$
conf1	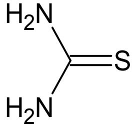<br>thiourea<br><br>C 0.00000 0.00000 -0.31486<br>S 0.00000 0.00000 1.35121<br>N 0.00000 1.14517 -1.04697<br>N 0.00000 -1.14517 -1.04697<br>H -0.30760 1.14066 -2.00786<br>H -0.13144 1.99837 -0.52848<br>H 0.30760 -1.14066 -2.00786<br>H 0.13144 -1.99837 -0.52848	C_2	-547.91331 (0.0)	-547.84571 (0.0)	-547.87832 (0.0)	-548.20584 (0.0)	-548.17086 (0.0)
conf2	C 0.00000 0.00000 -0.31875<br>S 0.00000 0.00000 1.35156<br>N 0.00000 1.14538 -1.03958<br>N 0.00000 -1.14538 -1.03958<br>H 0.00000 1.16753 -2.04640<br>H 0.00000 2.01375 -0.53277<br>H 0.00000 -1.16753 -2.04640<br>H 0.00000 -2.01375 -0.53277	C_{2v}	-547.91294 (0.2)	saddle point			
conf1	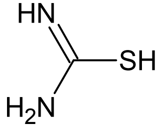<br>carbamimidothioic acid<br><br>C -0.47066 0.12328 -0.00030<br>S 1.29604 -0.12102 0.00994<br>N -1.15292 -1.07152 0.05825<br>N -0.94203 1.30642 -0.00101<br>H -2.14487 -1.03617 -0.12064<br>H -0.69378 -1.90377 -0.27868<br>H -1.96205 1.30537 -0.01441<br>H 1.55258 1.18695 -0.14414	C_1	-547.88448 (18.1)	-547.82046 (15.8)	-547.85421 (15.1)	-548.18039 (16.0)	-548.15012 (13.0)
conf2	C -0.47485 -0.12077 -0.00742	C_1	-547.88055 (20.6)	-547.81653 (18.3)	-547.85012 (17.7)	-548.17801 (17.5)	-548.14758 (14.6)

	S 1.30742 -0.06616 0.03454<br>N -1.09407 1.11553 -0.04976<br>N -1.00986 -1.27441 -0.02313<br>H -2.09923 1.10096 0.05476<br>H -0.64693 1.87085 0.44804<br>H -2.02597 -1.21677 0.04845<br>H 1.43008 1.14029 -0.54909						
conf3	C -0.49343 0.12756 -0.00610<br>S 1.29336 -0.13063 0.04153<br>N -1.15054 -1.07334 0.04358<br>N -1.12389 1.23176 -0.00496<br>H -2.15566 -0.99487 -0.02101<br>H -0.73828 -1.86123 -0.43062<br>H -0.51388 2.03519 0.10240<br>H 1.59567 1.03661 -0.54900	C_1	-547.88725 (16.4)	-547.82280 (14.4)	-547.85624 (13.9)	-548.18292 (14.4)	-548.15190 (11.9)
conf4	C 0.49469 0.12137 -0.00173<br>S -1.30403 0.02519 0.04579<br>N 1.12572 -1.09878 -0.04981<br>N 1.14851 1.21094 -0.00539<br>H 2.13167 -1.02479 0.02965<br>H 0.71484 -1.85026 0.48271<br>H 0.54288 2.01979 -0.10746<br>H -1.41261 -1.06112 -0.74073	C_1	-547.88683 (16.6)	-547.82242 (14.6)	-547.85589 (14.1)	-548.18304 (14.3)	-548.15210 (11.8)

Table S3.2. Reduced neutral form in water.

Structure	Schematic drawing	Symmetry	G_{PCM}	$H_{PCM,RRHO}$	$G_{PCM,RRHO}$	$G_{SMD,M06\text{-}2X,large}$	$G_{SMD,RRHO,M06\text{-}2X,large}$
conf1	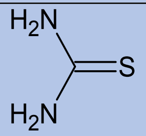<br><br>thiourea<br><br>C 0.00000 0.00000 -0.34013<br>S 0.00000 0.00000 1.36078<br>N 0.00000 1.14306 -1.03925<br>N 0.00000 -1.14306 -1.03925<br>H 0.00000 1.16486 -2.04822<br>H 0.00000 2.01839 -0.54292<br>H 0.00000 -1.16486 -2.04822<br>H 0.00000 -2.01839 -0.54292	C_{2v}	-547.93226 (0.0)	-547.86492 (0.0)	-547.89890 (0.0)	-548.22794 (0.0)	-548.19458 (0.0)

conf1	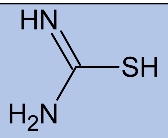<br>carbamimidothioic acid<br><br>C -0.47222 0.11775 -0.00216<br>S 1.29864 -0.11844 0.00712<br>N -1.15003 -1.06616 0.05352<br>N -0.95099 1.30526 -0.00102<br>H -2.14575 -1.04262 -0.11244<br>H -0.69094 -1.91685 -0.23551<br>H -1.97132 1.28358 -0.01559<br>H 1.57013 1.19064 -0.10495	C_1	-547.89657 (22.4)	-547.83264 (20.3)	-547.86651 (20.3)	-548.19561 (20.3)	-548.16555 (18.2)
conf2	C -0.47539 -0.10992 -0.00303<br>S 1.30832 -0.06727 0.02004<br>N -1.11302 1.09787 -0.05030<br>N -0.99682 -1.27921 -0.01297<br>H -2.11457 1.08672 0.08722<br>H -0.65249 1.91573 0.31904<br>H -2.01401 -1.22483 0.03659<br>H 1.46922 1.22754 -0.30238	C_1	-547.89537 (23.1)	-547.83133 (21.1)	-547.86521 (21.1)	-548.19559 (20.3)	-548.16544 (18.3)
conf3	C -0.49164 0.12585 -0.00581<br>S 1.28686 -0.14747 0.02577<br>N -1.16759 -1.05840 0.04970<br>N -1.10100 1.24945 -0.00507<br>H -2.16956 -0.98893 -0.06086<br>H -0.74587 -1.88155 -0.35283<br>H -0.46395 2.03681 0.06164<br>H 1.61960 1.10065 -0.33784	C_1	-547.89806 (21.5)	-547.83401 (19.4)	-547.86784 (19.5)	-548.19672 (19.6)	-548.16651 (17.6)
conf4	C 0.49337 0.11547 -0.00166<br>S -1.29864 0.02587 0.02820<br>N 1.14452 -1.08287 -0.05125<br>N 1.12525 1.22755 -0.00077<br>H 2.14701 -1.02305 0.06835<br>H 0.71519 -1.88979 0.37471<br>H 0.49444 2.02049 -0.07781<br>H -1.42711 -1.22711 -0.44239	C_1	-547.89750 (21.8)	-547.83338 (19.8)	-547.86713 (19.9)	-548.19658 (19.7)	-548.16621 (17.8)

Table S3.3. Reduced anionic form in water.

Structure	Schematic drawing	Symmetry	G_{PCM}	$H_{PCM,RRHO}$	$G_{PCM,RRHO}$	$G_{SMD,M06\text{-}2X,large}$	$G_{SMD,RRHO,M06\text{-}2X,large}$
conf1	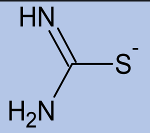<br>carbamimidothioate<br><br>C 0.39917 -0.12037 0.00245<br>S -1.35261 -0.00621 -0.00299<br>N 1.06306 1.09851 0.07562<br>N 1.03003 -1.25654 -0.00871<br>H 2.02733 1.11269 -0.23068<br>H 0.52857 1.90555 -0.20792<br>H 2.03926 -1.09045 0.00341	C_1	-547.44126 (3.3)	-547.38714 (3.1)	-547.41952 (3.1)	-547.73735 (2.7)	-547.71561 (2.4)
conf2	C 0.41815 0.11653 -0.00444<br>S -1.33314 -0.05984 0.00201<br>N 1.13759 -1.06433 -0.07653<br>N 1.09224 1.22673 0.00982<br>H 2.10874 -0.99146 0.19668<br>H 0.66588 -1.89167 0.25287<br>H 0.43795 2.00455 0.01194	C_1	-547.44656 (0.0)	-547.39208 (0.0)	-547.42444 (0.0)	-547.74158 (0.0)	-547.71945 (0.0)

Table S3.4. One-electron oxidized neutral form in vacuo.

Structure	Schematic drawing	Symmetry	E	H_{RRHO}	G_{RRHO}	$E_{M06\text{-}2X,large}$	$G_{RRHO,M06\text{-}2X,large}$
conf1	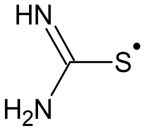<br>carbamimidoylsulfanyl<br><br>C 0.40905 -0.10035 -0.03558<br>S -1.32084 0.07586 0.00566<br>N 1.17778 1.02450 0.03572<br>N 0.81994 -1.32665 -0.03014<br>H 2.15955 0.97073 -0.18543<br>H 0.74070 1.93043 -0.00238<br>H 1.79495 -1.39771 0.27169	C_1	-547.24205 (6.1)	-547.18890 (5.7)	-547.22307 (5.2)	-547.53349 (4.3)	-547.51451 (3.5)
conf2	C 0.40995 0.09547 0.04327	C_1	-547.25176 (0.0)	-547.19804 (0.0)	-547.23140 (0.0)	-547.54041 (0.0)	-547.52005 (0.0)

	S -1.29327 -0.16625 -0.00843<br>N 1.27887 -0.93932 0.05190<br>N 0.84678 1.32140 -0.06405<br>H 2.25272 -0.72426 -0.10400<br>H 0.94959 -1.86251 -0.17676<br>H 0.15085 1.99937 0.24106						

Table S3.5. One-electron oxidized neutral form in water.

Structure	Schematic drawing	Symmetry	G_{PCM}	$H_{PCM,RRHO}$	$G_{PCM,RRHO}$	$G_{SMD,M06\text{-}2X,large}$	$G_{SMD,RRHO,M06\text{-}2X,large}$
conf1	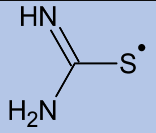<br><br>carbamimidoylsulfanyl<br><br>C 0.41054 -0.08621 -0.04244<br>S -1.31777 0.09229 0.01094<br>N 1.20084 1.00087 -0.00077<br>N 0.78114 -1.33652 -0.03733<br>H 2.20591 0.91125 -0.01967<br>H 0.80660 1.92674 0.04915<br>H 1.73460 -1.44769 0.31687	C_1	-547.25491 (3.1)	-547.20183 (3.0)	-547.23617 (2.5)	-547.54934 (1.7)	-547.53060 (1.1)
conf2	C 0.41416 0.08880 -0.04185<br>S -1.28635 -0.18627 0.00873<br>N 1.30180 -0.90988 -0.02277<br>N 0.79818 1.34232 0.06962<br>H 2.28737 -0.69735 0.03210<br>H 1.00432 -1.86849 0.06094<br>H 0.10508 1.98623 -0.30945	C_1	-547.25992 (0.0)	-547.20656 (0.0)	-547.24022 (0.0)	-547.55198 (0.0)	-547.53228 (0.0)

Part 4: 4-Methylcatechol

Table S4.1. Reduced neutral form in vacuo.

Structure	Schematic drawing	Symmetry	E	H_{RRHO}	G_{RRHO}	$E_{M06\text{-}2X,large}$	$G_{RRHO,M06\text{-}2X,large}$
conf1	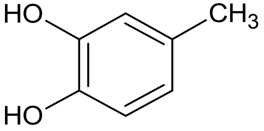 4-methylbenzene-1,2-diol<br><br>C 1.24078 0.40418 0.00000<br>C 0.00000 1.04876 0.00000<br>C -1.17899 0.31329 0.00000<br>C -1.15254 -1.08504 0.00000<br>C 0.09313 -1.71836 0.00000<br>C 1.27728 -0.98473 0.00000<br>H -2.13414 0.83717 0.00000<br>H 0.14301 -2.80428 0.00000<br>H 2.24438 -1.47802 0.00000<br>O 2.39486 1.12018 0.00000<br>O 0.05576 2.41802 0.00000<br>C -2.42894 -1.88179 0.00000<br>H -2.49546 -2.52835 0.88209<br>H -3.30702 -1.22978 0.00000<br>H -2.49546 -2.52835 -0.88209<br>H -0.83152 2.78944 0.00000<br>H 2.16683 2.05881 0.00000	C_s	-421.55849 (0.3)	saddle point			
conf2	C -1.24320 0.39958 0.00000<br>C 0.00000 1.04629 0.00000<br>C 1.17767 0.31415 0.00000<br>C 1.15169 -1.08725 0.00000<br>C -0.09102 -1.71990 0.00000<br>C -1.27887 -0.98672 0.00000<br>H 2.13265 0.83898 0.00000<br>H -0.13932 -2.80556 0.00000<br>H -2.24480 -1.48212 0.00000<br>O -2.39546 1.11797 0.00000<br>O -0.06067 2.41503 0.00000<br>C 2.43576 -1.87062 0.00000<br>H 3.04469 -1.64422 0.88275<br>H 2.23948 -2.94616 0.00000	C_s	-421.55868 (0.1)	-421.41183 (0.1)	-421.45486 (0.1)	-421.97231 (0.1)	-421.86849 (0.1)

	H 3.04469 -1.64422 -0.88275<br>H 0.82522 2.78985 0.00000<br>H -2.16568 2.05619 0.00000						
conf3	C 1.22122 0.38519 0.00000<br>C 0.00000 1.06700 0.00000<br>C -1.18938 0.34729 0.00000<br>C -1.18785 -1.05032 0.00000<br>C 0.04110 -1.71570 0.00000<br>C 1.23940 -1.00265 0.00000<br>H -2.12282 0.90323 0.00000<br>H 0.06958 -2.80214 0.00000<br>H 2.19092 -1.53108 0.00000<br>O 2.33617 1.18382 0.00000<br>O -0.03035 2.42382 0.00000<br>C -2.48049 -1.82098 0.00000<br>H -2.55972 -2.46584 0.88228<br>H -3.34330 -1.14962 0.00000<br>H -2.55972 -2.46584 -0.88228<br>H 0.88089 2.74490 0.00000<br>H 3.13367 0.64628 0.00000	C_s	-421.55863 (0.2)	saddle point			
conf4	C -1.22322 0.38014 0.00000<br>C 0.00000 1.06472 0.00000<br>C 1.18783 0.34812 0.00000<br>C 1.18681 -1.05249 0.00000<br>C -0.03888 -1.71759 0.00000<br>C -1.24090 -1.00510 0.00000<br>H 2.12140 0.90450 0.00000<br>H -0.06561 -2.80375 0.00000<br>H -2.19130 -1.53535 0.00000<br>O -2.33657 1.18036 0.00000<br>O 0.02498 2.42148 0.00000<br>C 2.48727 -1.80853 0.00000<br>H 3.08996 -1.56511 0.88217<br>H 2.31715 -2.88863 0.00000<br>H 3.08996 -1.56511 -0.88217<br>H -0.88748 2.73915 0.00000<br>H -3.13494 0.64399 0.00000	C_s	-421.55890 (0.0)	-421.41204 (0.0)	-421.45497 (0.0)	-421.97253 (0.0)	-421.86860 (0.0)

Table S4.2. Reduced neutral form in water.

Structure	Schematic drawing	Symmetry	G_{PCM}	$H_{PCM,RRHO}$	$G_{PCM,RRHO}$	$G_{SMD,M06\text{-}2X,large}$	$G_{SMD,RRHO,M06\text{-}2X,large}$
conf1	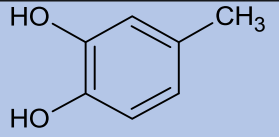<br>4-methylbenzene-1,2-diol<br><br>C 1.23938 0.40162 0.00000<br>C 0.00000 1.05181 0.00000<br>C -1.18030 0.31547 0.00000<br>C -1.15391 -1.08407 0.00000<br>C 0.09099 -1.72151 0.00000<br>C 1.27692 -0.98761 0.00000<br>H -2.13304 0.84151 0.00000<br>H 0.13985 -2.80747 0.00000<br>H 2.24199 -1.48625 0.00000<br>O 2.39788 1.11895 0.00000<br>O 0.05987 2.41654 0.00000<br>C -2.43235 -1.87830 0.00000<br>H -2.49803 -2.52601 0.88113<br>H -3.30772 -1.22343 0.00000<br>H -2.49803 -2.52601 -0.88113<br>H -0.82504 2.79940 0.00000<br>H 2.17364 2.05989 0.00000	C_s	-421.56752 (0.2)	saddle point			
conf2	C -1.24179 0.39675 0.00000<br>C 0.00000 1.04923 0.00000<br>C 1.17887 0.31632 0.00000<br>C 1.15304 -1.08622 0.00000<br>C -0.08881 -1.72329 0.00000<br>C -1.27858 -0.98986 0.00000<br>H 2.13161 0.84283 0.00000<br>H -0.13626 -2.80906 0.00000<br>H -2.24248 -1.49061 0.00000<br>O -2.39834 1.11647 0.00000<br>O -0.06533 2.41332 0.00000<br>C 2.43901 -1.86715 0.00000<br>H 3.04736 -1.63513 0.88131<br>H 2.24560 -2.94311 0.00000<br>H 3.04736 -1.63513 -0.88131<br>H 0.81790 2.80015 0.00000<br>H -2.17209 2.05698 0.00000	C_s	-421.56775 (0.0)	-421.42125 (0.0)	-421.46421 (0.0)	-421.98478 (0.0)	-421.88124 (0.0)

conf3	C 1.22492 0.38733 0.00000<br>C 0.00000 1.06483 0.00000<br>C -1.19097 0.34635 0.00000<br>C -1.18936 -1.05279 0.00000<br>C 0.04246 -1.71512 0.00000<br>C 1.24211 -1.00233 0.00000<br>H -2.12724 0.89868 0.00000<br>H 0.07281 -2.80164 0.00000<br>H 2.19323 -1.52956 0.00000<br>O 2.33425 1.18662 0.00000<br>O -0.03203 2.42584 0.00000<br>C -2.48148 -1.82480 0.00000<br>H -2.55822 -2.47137 0.88108<br>H -3.34503 -1.15451 0.00000<br>H -2.55822 -2.47137 -0.88108<br>H 0.87879 2.75215 0.00000<br>H 3.13992 0.65714 0.00000	C_s	-421.56757 (0.2)	saddle point			
conf4	C -1.22706 0.38224 0.00000<br>C 0.00000 1.06247 0.00000<br>C 1.18945 0.34726 0.00000<br>C 1.18847 -1.05484 0.00000<br>C -0.04019 -1.71692 0.00000<br>C -1.24370 -1.00473 0.00000<br>H 2.12576 0.89998 0.00000<br>H -0.06907 -2.80323 0.00000<br>H -2.19367 -1.53388 0.00000<br>O -2.33449 1.18343 0.00000<br>O 0.02649 2.42328 0.00000<br>C 2.48798 -1.81318 0.00000<br>H 3.09190 -1.56966 0.88121<br>H 2.31452 -2.89257 0.00000<br>H 3.09190 -1.56966 -0.88121<br>H -0.88565 2.74602 0.00000<br>H -3.14133 0.65562 0.00000	C_s	-421.56782 (0.0)	-421.42129 (0.0)	-421.46423 (0.0)	-421.98481 (0.0)	-421.88122 (0.0)

Table S4.3. Reduced anionic form in water.

Structure	Schematic drawing	Symmetry	G_{PCM}	$H_{PCM,RRHO}$	$G_{PCM,RRHO}$	$G_{SMD,M06\text{-}2X,large}$	$G_{SMD,RRHO,M06\text{-}2X,large}$
3-phenolate, conf1	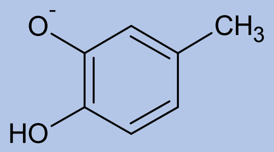<br>2-hydroxy-5-methylphenolate<br><br>C 1.23135 0.40988 0.00000<br>C 0.00000 1.13919 0.00000<br>C -1.18085 0.36466 0.00000<br>C -1.15602 -1.04032 0.00000<br>C 0.07408 -1.70326 0.00000<br>C 1.27547 -0.96988 0.00000<br>H -2.13808 0.88573 0.00000<br>H 0.10827 -2.79071 0.00000<br>H 2.23651 -1.48075 0.00000<br>O 2.34794 1.20113 0.00000<br>O 0.08342 2.43806 0.00000<br>C -2.44724 -1.82184 0.00000<br>H -2.52819 -2.47021 0.88059<br>H -3.31438 -1.15444 0.00000<br>H -2.52819 -2.47021 -0.88059<br>H 1.93236 2.09642 0.00000	C_s	-421.09381 (0.4)	saddle point			
3-phenolate, conf2	C -1.23349 0.40440 0.00000<br>C 0.00000 1.13739 0.00000<br>C 1.18028 0.36730 0.00000<br>C 1.15604 -1.04025 0.00000<br>C -0.07055 -1.70420 0.00000<br>C -1.27668 -0.97330 0.00000<br>H 2.13731 0.88928 0.00000<br>H -0.10115 -2.79153 0.00000<br>H -2.23618 -1.48680 0.00000<br>O -2.34733 1.19708 0.00000<br>O -0.09172 2.43537 0.00000<br>C 2.45192 -1.81132 0.00000<br>H 3.06227 -1.57588 0.88040<br>H 2.27131 -2.89072 0.00000	C_s	-421.09440 (0.0)	-420.96163 (0.0)	-421.00299 (0.0)	-421.51311 (0.0)	-421.42170 (0.0)

	H 3.06227 -1.57588 -0.88040<br>H -1.92852 2.09183 0.00000						
4-phenolate, conf1	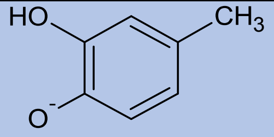<br>2-hydroxy-4-methylphenolate<br><br>C 1.29751 0.46157 0.00000<br>C 0.00000 1.06397 0.00000<br>C -1.17343 0.33189 0.00000<br>C -1.13275 -1.07739 0.00000<br>C 0.12164 -1.69072 0.00000<br>C 1.31231 -0.94798 0.00000<br>H -2.12966 0.85354 0.00000<br>H 0.18076 -2.77887 0.00000<br>H 2.27283 -1.46102 0.00000<br>O 2.32343 1.26683 0.00000<br>O 0.02636 2.43028 0.00000<br>C -2.40541 -1.88625 0.00000<br>H -2.47919 -2.53621 0.88069<br>H -3.28556 -1.23549 0.00000<br>H -2.47919 -2.53621 -0.88069<br>H 1.00246 2.58680 0.00000	C_s	-421.09300 (0.9)	saddle point			
4-phenolate, conf2	C -1.29974 0.45594 0.00000<br>C 0.00000 1.06256 0.00000<br>C 1.17325 0.33506 0.00000<br>C 1.13340 -1.07694 0.00000<br>C -0.11759 -1.69150 0.00000<br>C -1.31303 -0.95107 0.00000<br>H 2.12863 0.85854 0.00000<br>H -0.17274 -2.77964 0.00000<br>H -2.27178 -1.46720 0.00000<br>O -2.32327 1.26345 0.00000<br>O -0.03358 2.42743 0.00000<br>C 2.41068 -1.87526 0.00000<br>H 3.02941 -1.65997 0.88050<br>H 2.20144 -2.94966 0.00000<br>H 3.02941 -1.65997 -0.88050<br>H -1.01138 2.57804 0.00000	C_s	-421.09369 (0.4)	-420.96094 (0.4)	-421.00223 (0.5)	-421.51218 (0.6)	-421.42072 (0.6)

Table S4.4. One-electron oxidized neutral form in vacuo.

Structure	Schematic drawing	Symmetry	*E*	H_{RRHO}	G_{RRHO}	$E_{M06-2X,large}$	$G_{RRHO,M06-2X,large}$
benzyl, conf1	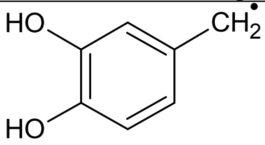<br>3,4-dihydroxybenzyl<br><br>C -1.21029 0.29999 0.00000<br>C -0.00000 1.01993 0.00000<br>C 1.21202 0.36514 -0.00000<br>C 1.27389 -1.05862 -0.00000<br>C 0.03691 -1.76231 -0.00000<br>C -1.17471 -1.09451 0.00000<br>H 2.13728 0.93850 -0.00000<br>H 0.05139 -2.84832 -0.00000<br>H -2.11621 -1.63488 0.00000<br>O -2.39704 0.95056 0.00000<br>O -0.15107 2.38073 0.00000<br>C 2.50291 -1.73697 -0.00000<br>H 2.53821 -2.82072 -0.00000<br>H 3.44333 -1.19652 -0.00000<br>H 0.70757 2.81476 0.00000<br>H -2.22100 1.90101 0.00000	C_s	-420.91101<br>(13.9)	-420.77789<br>(13.2)	-420.81958<br>(13.7)	-421.32060<br>(12.1)	-421.22917<br>(11.9)
benzyl, conf2	C -1.18877 0.28608 0.00000<br>C 0.00000 1.04055 0.00000<br>C 1.22047 0.39728 0.00000<br>C 1.30511 -1.02261 0.00000<br>C 0.08740 -1.75578 0.00000<br>C -1.13648 -1.10578 0.00000<br>H 2.12418 0.99931 0.00000<br>H 0.12124 -2.84121 0.00000<br>H -2.06150 -1.67921 0.00000<br>O -2.34185 1.02144 0.00000<br>O -0.05697 2.39564 0.00000<br>C 2.54883 -1.67950 0.00000<br>H 2.60626 -2.76266 0.00000<br>H 3.47787 -1.11987 0.00000<br>H -0.98560 2.66175 0.00000<br>H -3.11123 0.44379 0.00000	C_s	-420.91005<br>(14.5)	-420.77704<br>(13.8)	-420.81883<br>(14.2)	-421.31977<br>(12.6)	-421.22855<br>(12.3)

3-phenoxyl, conf1	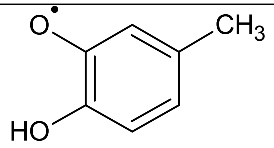<br><br>(2-hydroxy-5-methylphenyl)oxidanyl<br><br>C 1.26215 0.40804 0.00000<br>C 0.00000 1.14746 0.00000<br>C -1.21606 0.38255 0.00000<br>C -1.18750 -0.98981 0.00000<br>C 0.07504 -1.66130 0.00000<br>C 1.28450 -0.98168 0.00000<br>H -2.15168 0.93441 0.00000<br>H 0.08166 -2.74857 0.00000<br>H 2.23294 -1.50907 0.00000<br>O 2.36354 1.14617 0.00000<br>O 0.07235 2.39951 0.00000<br>C -2.44586 -1.80923 0.00000<br>H -2.49231 -2.45833 0.88184<br>H -3.33467 -1.17405 0.00000<br>H -2.49231 -2.45833 -0.88184<br>H 2.03568 2.07225 0.00000	C_s	-420.93181 (0.8)	-420.79771 (0.8)	-420.83961 (1.1)	-421.33846 (0.9)	-421.24625 (1.2)
3-phenoxyl, conf2	C -1.26535 0.40741 0.00000<br>C 0.00000 1.14381 0.00000<br>C 1.21303 0.37775 0.00000<br>C 1.18355 -0.99722 0.00000<br>C -0.07845 -1.66227 0.00000<br>C -1.28933 -0.98004 0.00000<br>H 2.14933 0.92926 0.00000<br>H -0.08898 -2.74917 0.00000<br>H -2.23758 -1.50762 0.00000<br>O -2.36450 1.14909 0.00000<br>O -0.06956 2.39657 0.00000<br>C 2.45458 -1.80265 0.00000<br>H 3.06348 -1.57824 0.88205<br>H 2.24911 -2.87626 0.00000<br>H 3.06348 -1.57824 -0.88205<br>H -2.03450 2.07423 0.00000	C_s	-420.93081 (1.5)	saddle point			

4-phenoxyl, conf1	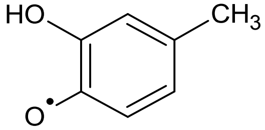 (2-hydroxy-4-methylphenyl)oxidanyl<br><br>C 1.30397 0.43728 0.00000<br>C 0.00000 1.09549 0.00000<br>C -1.18387 0.37252 0.00000<br>C -1.13686 -1.02189 0.00000<br>C 0.12468 -1.68896 0.00000<br>C 1.30757 -0.99965 0.00000<br>H -2.13297 0.90042 0.00000<br>H 0.13326 -2.77640 0.00000<br>H 2.26909 -1.50333 0.00000<br>O 2.32234 1.17055 0.00000<br>O 0.02616 2.42262 0.00000<br>C -2.39490 -1.83473 0.00000<br>H -2.43834 -2.48649 0.88059<br>H -3.28544 -1.20222 0.00000<br>H -2.43834 -2.48649 -0.88059<br>H 0.98116 2.64883 0.00000	C_s	-420.93312 (0.0)	-420.79901 (0.0)	-420.84139 (0.0)	-421.33984 (0.0)	-421.24811 (0.0)
4-phenoxyl, conf2	C -1.30582 0.43364 0.00000<br>C 0.00000 1.09261 0.00000<br>C 1.17939 0.36991 0.00000<br>C 1.13175 -1.02903 0.00000<br>C -0.12656 -1.69384 0.00000<br>C -1.31087 -1.00195 0.00000<br>H 2.12985 0.89617 0.00000<br>H -0.13683 -2.78076 0.00000<br>H -2.27261 -1.50515 0.00000<br>O -2.32383 1.16712 0.00000<br>O -0.02758 2.42040 0.00000<br>C 2.40473 -1.82029 0.00000<br>H 3.01280 -1.58389 0.88077<br>H 2.21174 -2.89560 0.00000<br>H 3.01280 -1.58389 -0.88077<br>H -0.98220 2.64657 0.00000	C_s	-420.93271 (0.3)	saddle point			

Table S4.5. One-electron oxidized neutral form in water.

Structure	Schematic drawing	Symmetry	G_{PCM}	$H_{PCM,RRHO}$	$G_{PCM,RRHO}$	$G_{SMD,M06-2X,large}$	$G_{SMD,RRHO,M06-2X,large}$
benzyl, conf1	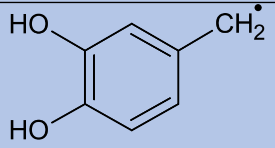<br>3,4-dihydroxybenzyl<br><br>C -1.20842 0.29744 -0.00000<br>C -0.00000 1.02386 0.00000<br>C 1.21366 0.36844 0.00000<br>C 1.27463 -1.05643 0.00000<br>C 0.03981 -1.76539 -0.00000<br>C -1.17348 -1.09728 -0.00000<br>H 2.13711 0.94339 0.00000<br>H 0.05479 -2.85156 -0.00000<br>H -2.11267 -1.64267 -0.00000<br>O -2.39914 0.94732 -0.00000<br>O -0.15685 2.37897 0.00000<br>C 2.50639 -1.73390 0.00000<br>H 2.54104 -2.81819 0.00000<br>H 3.44482 -1.18945 0.00000<br>H 0.69698 2.82722 0.00000<br>H -2.22959 1.90048 -0.00000	C_s	-420.92015 (13.1)	-420.78737 (12.4)	-420.82907 (12.8)	-421.33243 (10.7)	-421.24135 (10.3)
benzyl, conf2	C -1.19288 0.28673 0.00000<br>C 0.00000 1.03824 0.00000<br>C 1.22211 0.39791 0.00000<br>C 1.30671 -1.02389 0.00000<br>C 0.08631 -1.75559 0.00000<br>C -1.13902 -1.10699 0.00000<br>H 2.12824 0.99738 0.00000<br>H 0.11885 -2.84133 0.00000<br>H -2.06361 -1.67914 0.00000<br>O -2.33807 1.02317 0.00000<br>O -0.05772 2.39674 0.00000<br>C 2.55053 -1.68150 0.00000<br>H 2.60494 -2.76514 0.00000<br>H 3.47977 -1.12123 0.00000<br>H -0.98708 2.66590 0.00000<br>H -3.11740 0.45497 0.00000	C_s	-420.91976 (13.4)	-420.78689 (12.7)	-420.82848 (13.2)	-421.33219 (10.8)	-421.24092 (10.6)

3-phenoxyl, conf1	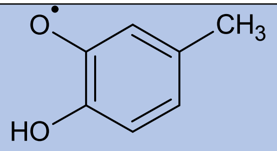<br>(2-hydroxy-5-methylphenyl)oxidanyl<br><br>C 1.26066 0.40691 0.00000<br>C 0.00000 1.14753 0.00000<br>C -1.21600 0.38518 0.00000<br>C -1.19041 -0.98749 0.00000<br>C 0.07320 -1.66203 0.00000<br>C 1.28188 -0.98304 0.00000<br>H -2.15400 0.93350 0.00000<br>H 0.07933 -2.74878 0.00000<br>H 2.22813 -1.51446 0.00000<br>O 2.37039 1.13753 0.00000<br>O 0.07013 2.40407 0.00000<br>C -2.44766 -1.80529 0.00000<br>H -2.48816 -2.45603 0.88058<br>H -3.33654 -1.17079 0.00000<br>H -2.48816 -2.45603 -0.88058<br>H 2.06529 2.06917 0.00000	C_s	-420.93956 (0.9)	-420.80570 (0.9)	-420.84762 (1.2)	-421.34769 (1.1)	-421.25576 (1.3)
3-phenoxyl, conf2	C -1.26408 0.40635 0.00000<br>C 0.00000 1.14355 0.00000<br>C 1.21285 0.37966 0.00000<br>C 1.18615 -0.99535 0.00000<br>C -0.07720 -1.66319 0.00000<br>C -1.28727 -0.98130 0.00000<br>H 2.15170 0.92708 0.00000<br>H -0.08791 -2.74962 0.00000<br>H -2.23340 -1.51283 0.00000<br>O -2.37130 1.14102 0.00000<br>O -0.06715 2.40064 0.00000<br>C 2.45646 -1.79918 0.00000<br>H 3.06421 -1.57031 0.88147<br>H 2.25084 -2.87209 0.00000<br>H 3.06421 -1.57031 -0.88147<br>H -2.06349 2.07163 0.00000	C_s	-420.93841 (1.6)	saddle point			

4-phenoxyl, conf1	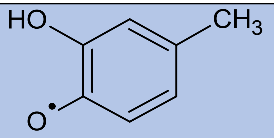 (2-hydroxy-4-methylphenyl)oxidanyl<br><br>C 1.30343 0.43744 0.00000<br>C 0.00000 1.09464 0.00000<br>C -1.18333 0.37206 0.00000<br>C -1.13744 -1.02306 0.00000<br>C 0.12673 -1.68975 0.00000<br>C 1.30791 -0.99883 0.00000<br>H -2.13388 0.89738 0.00000<br>H 0.13668 -2.77666 0.00000<br>H 2.26717 -1.50738 0.00000<br>O 2.32799 1.16982 0.00000<br>O 0.01676 2.42540 0.00000<br>C -2.39251 -1.83610 0.00000<br>H -2.42884 -2.48974 0.87929<br>H -3.28393 -1.20582 0.00000<br>H -2.42884 -2.48974 -0.87929<br>H 0.96484 2.67177 0.00000	C_s	-420.94103 (0.0)	-420.80717 (0.0)	-420.84947 (0.0)	-421.34941 (0.0)	-421.25785 (0.0)
4-phenoxyl, conf2	C -1.30531 0.43393 0.00000<br>C 0.00000 1.09141 0.00000<br>C 1.17853 0.36878 0.00000<br>C 1.13182 -1.03089 0.00000<br>C -0.12933 -1.69496 0.00000<br>C -1.31165 -1.00118 0.00000<br>H 2.13076 0.89166 0.00000<br>H -0.14163 -2.78141 0.00000<br>H -2.27133 -1.50887 0.00000<br>O -2.32935 1.16657 0.00000<br>O -0.01791 2.42310 0.00000<br>C 2.40230 -1.82205 0.00000<br>H 3.00961 -1.58083 0.87975<br>H 2.20859 -2.89661 0.00000<br>H 3.00961 -1.58083 -0.87975<br>H -0.96568 2.66931 0.00000	C_s	-420.94053 (0.3)	saddle point			

Table S4.6. One-electron oxidized anionic form in water.

Structure	Schematic drawing	Symmetry	G_{PCM}	$H_{PCM,RRHO}$	$G_{PCM,RRHO}$	$G_{SMD,M06-2X,large}$	$G_{SMD,RRHO,M06-2X,large}$
benzyl, 3-phenolate, conf1	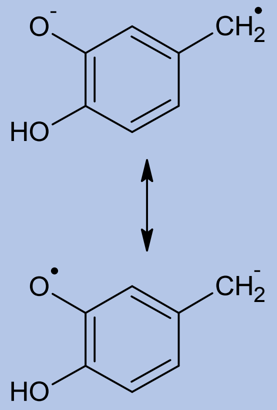<br>4-hydroxy-3-oxidobenzyl<br><br>C 1.19964 -0.30706 0.00000<br>C 0.00000 -1.11286 0.00000<br>C -1.21481 -0.42171 0.00000<br>C -1.27749 1.00761 0.00000<br>C -0.05438 1.73990 0.00000<br>C 1.17474 1.07890 0.00000<br>H -2.14394 -0.98990 0.00000<br>H -0.08726 2.82655 0.00000<br>H 2.10822 1.63736 0.00000<br>O 2.34207 -1.03736 0.00000<br>O 0.18788 -2.39944 0.00000<br>C -2.50826 1.69000 0.00000<br>H -3.45093 1.15066 0.00000<br>H -2.54353 2.77544 0.00000<br>H 1.96122 -1.95441 0.00000	C_s	-420.44908 (15.7)	-420.33005 (14.7)	-420.37055 (15.7)	-420.86176 (18.2)	-420.78324 (18.2)

benzyl, 4-phenolate, conf1	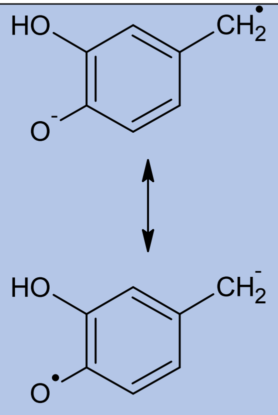 3-hydroxy-4-oxidobenzyl<br><br>C -1.27199 -0.35324 0.00000<br>C 0.00000 -1.03837 0.00000<br>C 1.20507 -0.38701 0.00000<br>C 1.25695 1.04839 0.00000<br>C 0.00407 1.73470 0.00000<br>C -1.21043 1.06241 0.00000<br>H 2.13093 -0.95906 0.00000<br>H 0.01308 2.82327 0.00000<br>H -2.14511 1.62024 0.00000<br>O -2.33658 -1.08665 0.00000<br>O -0.11122 -2.39613 0.00000<br>C 2.47498 1.74101 0.00000<br>H 3.42344 1.21207 0.00000<br>H 2.49880 2.82674 0.00000<br>H -1.09059 -2.50819 0.00000	C_s	-420.45293 (13.2)	-420.33354 (12.5)	-420.37407 (13.5)	-420.86601 (15.5)	-420.78715 (15.7)
phenoxyl, phenolate, conf1	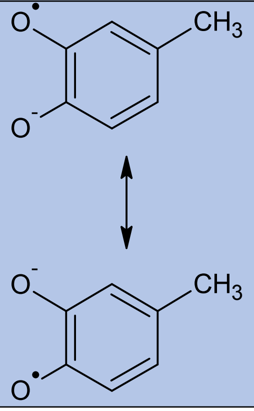	C_s	-420.47402 (0.0)	-420.35350 (0.0)	-420.39553 (0.0)	-420.89073 (0.0)	-420.81224 (0.0)

	(5-methyl-2-oxidophenyl)oxidanyl<br><br>C -1.32983 -0.52784 0.00000<br>C 0.00000 -1.20848 0.00000<br>C 1.16842 -0.37059 0.00000<br>C 1.11370 1.00652 0.00000<br>C -0.16464 1.64351 0.00000<br>C -1.32590 0.90942 0.00000<br>H 2.13174 -0.87948 0.00000<br>H -0.20993 2.73117 0.00000<br>H -2.29514 1.40475 0.00000<br>O -2.40693 -1.19311 0.00000<br>O 0.08411 -2.47123 0.00000<br>C 2.35697 1.85027 0.00000<br>H 2.39192 2.50403 0.87997<br>H 3.25969 1.23346 0.00000<br>H 2.39192 2.50403 -0.87997						
phenoxyl, phenolate, conf2	C 1.33244 -0.52461 0.00000<br>C 0.00000 -1.20606 0.00000<br>C -1.16581 -0.36900 0.00000<br>C -1.11083 1.01029 0.00000<br>C 0.16614 1.64508 0.00000<br>C 1.32943 0.91127 0.00000<br>H -2.12949 -0.87788 0.00000<br>H 0.21195 2.73248 0.00000<br>H 2.29811 1.40753 0.00000<br>O 2.40747 -1.19181 0.00000<br>O -0.08099 -2.46890 0.00000<br>C -2.36610 1.84128 0.00000<br>H -2.98396 1.63175 0.88076<br>H -2.13611 2.91054 0.00000<br>H -2.98396 1.63175 -0.88076	C_s	-420.47307 (0.6)	saddle point			

Table S4.7. Two-electron oxidized neutral form in vacuo.

**Structure**	**Schematic drawing**	**Symmetry**	***E***	H_{RRHO}	G_{RRHO}	$E_{M06\text{-}2X,large}$	$G_{RRHO,M06\text{-}2X,large}$
quinone, conf1	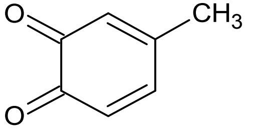	C_s	-420.30541 (8.8)	-420.18316 (8.4)	-420.22485 (7.2)	-420.71661 (9.7)	-420.63606 (8.1)

	4-methylcyclohexa-3,5-diene-1,2-dione<br><br>C 1.36717 0.49719 0.00000<br>C 0.00000 1.23393 0.00000<br>C -1.20941 0.40867 0.00000<br>C -1.14688 -0.94243 0.00000<br>C 0.15435 -1.62527 0.00000<br>C 1.33134 -0.97232 0.00000<br>H -2.15804 0.93900 0.00000<br>H 0.14112 -2.71362 0.00000<br>H 2.28599 -1.49015 0.00000<br>O 2.40303 1.13005 0.00000<br>O -0.02809 2.44918 0.00000<br>C -2.36813 -1.80594 0.00000<br>H -2.37765 -2.46014 0.88029<br>H -3.28395 -1.21180 0.00000<br>H -2.37765 -2.46014 -0.88029						
quinone, conf2	C -1.37014 0.49916 0.00000<br>C 0.00000 1.22954 0.00000<br>C 1.20458 0.39941 0.00000<br>C 1.13998 -0.95291 0.00000<br>C -0.16394 -1.62746 0.00000<br>C -1.33921 -0.96964 0.00000<br>H 2.15478 0.92790 0.00000<br>H -0.15776 -2.71538 0.00000<br>H -2.29508 -1.48513 0.00000<br>O -2.40354 1.13642 0.00000<br>O 0.03389 2.44507 0.00000<br>C 2.38143 -1.80024 0.00000<br>H 2.99588 -1.58959 0.88136<br>H 2.14737 -2.86737 0.00000<br>H 2.99588 -1.58959 -0.88136	C_s	-420.30287<br>(10.4)	saddle point			
quinone methide, conf1	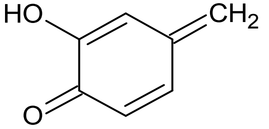<br>2-hydroxy-4-methylidenecyclohexa-2,5-dien-1-one<br><br>C -1.26924 -0.31460 -0.00000<br>C 0.00000 -1.07848 0.00000<br>C 1.20061 -0.45781 0.00000<br>C 1.27091 0.98975 0.00000	C_s	-420.31950<br>(0.0)	-420.19651<br>(0.0)	-420.23639<br>(0.0)	-420.73211<br>(0.0)	-420.64899<br>(0.0)

	C 0.02421 1.74684 0.00000<br>C -1.18054 1.13894 -0.00000<br>H 2.11920 -1.03698 0.00000<br>H 0.09563 2.83181 0.00000<br>H -2.11424 1.69241 -0.00000<br>O -2.32647 -0.94999 -0.00000<br>O -0.15497 -2.40692 -0.00000<br>C 2.45172 1.65185 0.00000<br>H 2.48786 2.73701 0.00000<br>H 3.39921 1.12141 0.00000<br>H -1.12217 -2.54928 -0.00000						

Table S4.8. Two-electron oxidized neutral form in water.

Structure	Schematic drawing	Symmetry	G_{PCM}	$H_{PCM,RRHO}$	$G_{PCM,RRHO}$	$G_{SMD,M06\text{-}2X,large}$	$G_{SMD,RRHO,M06\text{-}2X,large}$
quinone, conf1	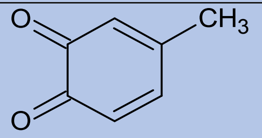<br><br>4-methylcyclohexa-3,5-diene-1,2-dione<br><br>C -1.36450 -0.48022 0.00000<br>C 0.00000 -1.22643 0.00000<br>C 1.20995 -0.42035 0.00000<br>C 1.15249 0.93353 0.00000<br>C -0.14633 1.62554 0.00000<br>C -1.32866 0.98126 0.00000<br>H 2.15859 -0.95003 0.00000<br>H -0.12558 2.71277 0.00000<br>H -2.27723 1.50938 0.00000<br>O -2.39970 -1.12405 0.00000<br>O 0.00259 -2.44848 0.00000<br>C 2.37370 1.79014 0.00000<br>H 2.37657 2.44621 0.87872<br>H 3.28811 1.19494 0.00000<br>H 2.37657 2.44621 -0.87872	C_s	-420.31923 (5.7)	-420.19707 (5.3)	-420.23871 (4.3)	-420.73127 (6.5)	-420.65075 (5.1)
quinone, conf2	C 1.36781 -0.48203 0.00000<br>C 0.00000 -1.22152 0.00000<br>C -1.20487 -0.41030 0.00000<br>C -1.14516 0.94479 0.00000<br>C 0.15656 1.62816 0.00000	C_s	-420.31662 (7.3)	saddle point			

	C 1.33719 0.97895 0.00000<br>H -2.15538 -0.93732 0.00000<br>H 0.14316 2.71504 0.00000<br>H 2.28714 1.50452 0.00000<br>O 2.40046 -1.13022 0.00000<br>O -0.00848 -2.44402 0.00000<br>C -2.38715 1.78412 0.00000<br>H -2.99927 1.56364 0.88053<br>H -2.15855 2.85138 0.00000<br>H -2.99927 1.56364 -0.88053						
quinone methide, conf1	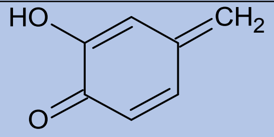<br>2-hydroxy-4-methylidenecyclohexa-2,5-dien-1-one<br><br>C -1.26625 -0.31532 0.00000<br>C -0.00000 -1.07446 0.00000<br>C 1.20036 -0.45285 -0.00000<br>C 1.26676 0.99540 -0.00000<br>C 0.02129 1.74894 -0.00000<br>C -1.18277 1.13385 0.00000<br>H 2.12236 -1.02698 -0.00000<br>H 0.08684 2.83381 -0.00000<br>H -2.11482 1.69034 0.00000<br>O -2.32818 -0.95710 0.00000<br>O -0.14653 -2.41088 0.00000<br>C 2.44959 1.65570 -0.00000<br>H 2.48502 2.74078 -0.00000<br>H 3.39577 1.12279 -0.00000<br>H -1.11133 -2.56453 0.00000	C_s	-420.32829 (0.0)	-420.20557 (0.0)	-420.24554 (0.0)	-420.74159 (0.0)	-420.65883 (0.0)

Table S4.9. Two-electron oxidized anionic form in water.

Structure	Schematic drawing	Symmetry	G_{PCM}	$H_{PCM,RRHO}$	$G_{PCM,RRHO}$	$G_{SMD,M06\text{-}2X,large}$	$G_{SMD,RRHO,M06\text{-}2X,large}$
conf1	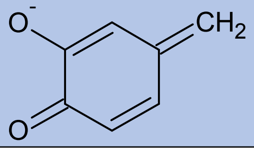	C_s	-419.84888 (0.0)	-419.74009 (0.0)	-419.78021 (0.0)	-420.26912 (0.0)	-420.20045 (0.0)

	3-methylidene-6-oxocyclohexa-1,4-dien-1-olate<br><br>C -1.28511 -0.39233 0.00000<br>C 0.00000 -1.20438 0.00000<br>C 1.18566 -0.47481 0.00000<br>C 1.24695 0.96039 0.00000<br>C -0.00830 1.70701 0.00000<br>C -1.19717 1.06993 0.00000<br>H 2.12568 -1.02534 0.00000<br>H 0.04275 2.79375 0.00000<br>H -2.13866 1.61467 0.00000<br>O -2.38988 -0.95018 0.00000<br>O -0.09670 -2.47401 0.00000<br>C 2.40664 1.67738 0.00000<br>H 2.39381 2.76328 0.00000<br>H 3.37703 1.18808 0.00000						

Part 5: 4-Methylcatechol – methanethiol adducts

Table S5.1. Reduced neutral form in vacuo.

Structure	Schematic drawing	Symmetry	*E*	H_{RRHO}	G_{RRHO}	$E_{M06-2X,large}$	$G_{RRHO,M06-2X,large}$
6-adduct, conf1	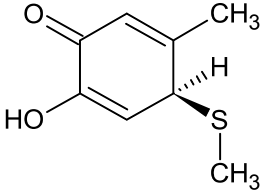<br>(4*S*)-2-hydroxy-5-methyl-4-(methylsulfanyl)cyclohexa-2,5-dien-1-one<br><br>C -1.54663 -0.65150 -0.42495<br>C -1.72640 0.62904 0.29538<br>C -0.60865 1.56156 0.31063<br>C 0.57470 1.27391 -0.27169<br>C 0.80699 -0.06871 -0.90424<br>C -0.37802 -0.96011 -1.01195<br>H -0.78821 2.52134 0.78801<br>H 1.24795 0.08636 -1.89951<br>H -0.25303 -1.90333 -1.53562<br>O -2.63380 -1.43874 -0.44243<br>O -2.81821 0.85101 0.82141<br>C 1.69486 2.26336 -0.31437<br>H 1.95909 2.49762 -1.35304<br>H 1.42117 3.19341 0.18839<br>H 2.59521 1.85238 0.15489<br>S 2.19164 -0.96522 -0.04713<br>C 1.46170 -1.24734 1.58175<br>H 2.20257 -1.81531 2.14972<br>H 1.26138 -0.30802 2.10352<br>H 0.54499 -1.83725 1.50775<br>H -3.31253 -0.94301 0.05469	C_1	-858.84447 (0.0)	-858.66558 (0.0)	-858.71576 (0.0)	-859.44565 (0.0)	-859.31693 (0.0)
6-adduct, conf2	C 1.46103 -0.98542 0.18384<br>C 2.04511 0.32639 -0.16632<br>C 1.17808 1.49289 -0.09311<br>C -0.12609 1.40295 0.23908<br>C -0.75272 0.06681 0.52943<br>C 0.16808 -1.10088 0.52818	C_1	-858.84031 (2.6)	-858.66143 (2.6)	-858.71201 (2.4)	-859.44052 (3.2)	-859.31222 (3.0)

	H 1.64354 2.45293 -0.29924<br>H -1.25984 0.14238 1.50344<br>H -0.23457 -2.07411 0.79230<br>O 2.32369 -2.01170 0.13215<br>O 3.23591 0.36920 -0.47895<br>C -0.99352 2.61717 0.35338<br>H -1.47800 2.65660 1.33617<br>H -0.40914 3.52977 0.21738<br>H -1.79806 2.59886 -0.38974<br>S -2.13133 -0.18065 -0.69325<br>C -3.02924 -1.52884 0.10768<br>H -3.93252 -1.68867 -0.48623<br>H -2.45609 -2.45910 0.11703<br>H -3.32753 -1.26015 1.12533<br>H 3.17241 -1.61454 -0.14292						
6-adduct, conf3	C 1.70450 -0.85496 0.19329<br>C 2.05101 0.54429 -0.13987<br>C 0.98334 1.53228 -0.10507<br>C -0.29114 1.20421 0.19059<br>C -0.67255 -0.22060 0.48325<br>C 0.44478 -1.20557 0.49933<br>H 1.26890 2.55809 -0.32192<br>H -1.18530 -0.24391 1.45692<br>H 0.20228 -2.23633 0.73889<br>O 2.74129 -1.70595 0.17288<br>O 3.22404 0.80427 -0.41055<br>C -1.37091 2.23837 0.23958<br>H -1.89153 2.22023 1.20455<br>H -0.96331 3.24020 0.08863<br>H -2.11719 2.04793 -0.54043<br>S -1.95637 -0.83695 -0.71263<br>C -3.48303 -0.59355 0.22970<br>H -4.28531 -1.01741 -0.37985<br>H -3.45280 -1.13301 1.18060<br>H -3.70444 0.46186 0.40631<br>H 3.51193 -1.15994 -0.07508	C_1	-858.83960 (3.1)	-858.66080 (3.0)	-858.71064 (3.2)	-859.44021 (3.4)	-859.31124 (3.6)

5-adduct, conf1	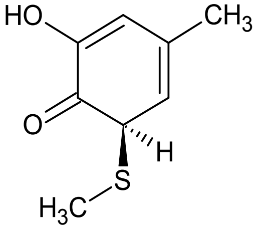<br>(6*R*)-2-hydroxy-4-methyl-6-(methylsulfanyl)cyclohexa-2,4-dien-1-one<br><br>C -0.44469 1.10035 -0.53786<br>C 0.79066 1.42638 0.19038<br>C 1.76720 0.50386 0.35345<br>C 1.65011 -0.84654 -0.18214<br>C 0.52543 -1.23021 -0.82064<br>C -0.65813 -0.33657 -0.96128<br>H 2.67633 0.78602 0.87919<br>H 0.42524 -2.23639 -1.21854<br>H -1.01862 -0.31575 -1.99779<br>O -1.25468 1.99561 -0.75012<br>O 0.87354 2.68903 0.63867<br>C 2.82884 -1.75951 -0.01023<br>H 3.72127 -1.34026 -0.48954<br>H 3.06959 -1.89537 1.05081<br>H 2.63760 -2.74321 -0.44569<br>S -2.11375 -1.04807 -0.07481<br>C -1.52748 -1.02199 1.63572<br>H -2.25763 -1.59264 2.21470<br>H -0.55184 -1.50678 1.72575<br>H -1.48219 -0.00503 2.03357<br>H 0.05779 3.12674 0.33172	C_1	-858.83713 (4.6)	-858.65837 (4.5)	-858.70845 (4.6)	-859.43766 (5.0)	-859.30898 (5.0)
5-adduct, conf2	C 0.40373 0.91181 0.47946<br>C -0.80415 1.42975 -0.18232<br>C -1.89342 0.64424 -0.33934<br>C -1.90405 -0.74659 0.09478<br>C -0.79705 -1.30923 0.62134<br>C 0.49149 -0.56875 0.72671<br>H -2.79198 1.06657 -0.78205<br>H -0.79273 -2.35163 0.92724<br>H 1.00465 -0.74975 1.67808<br>O 1.30263 1.69918 0.77073<br>O -0.75950 2.72609 -0.52382	C_1	-858.83911 (3.4)	-858.66029 (3.3)	-858.71016 (3.5)	-859.43908 (4.1)	-859.31013 (4.3)

	C -3.18971 -1.50340 -0.06099<br>H -4.00143 -1.01380 0.48968<br>H -3.49255 -1.54086 -1.11404<br>H -3.09694 -2.52884 0.30440<br>S 1.57621 -1.24801 -0.61713<br>C 3.18316 -0.54361 -0.18022<br>H 3.87521 -0.87931 -0.95720<br>H 3.15236 0.54788 -0.16888<br>H 3.52751 -0.91448 0.78887<br>H 0.11144 3.04495 -0.22377						
5-adduct, conf3	C 0.40655 -1.20347 0.40069<br>C 1.68100 -0.72824 -0.15340<br>C 1.93080 0.59483 -0.28955<br>C 0.95546 1.60309 0.09396<br>C -0.25761 1.23764 0.55796<br>C -0.68207 -0.18539 0.66234<br>H 2.89540 0.91229 -0.67792<br>H -0.97759 1.99571 0.85357<br>H -1.11370 -0.41089 1.64607<br>O 0.26093 -2.40410 0.60313<br>O 2.56643 -1.68971 -0.45415<br>C 1.36281 3.04057 -0.04342<br>H 2.26198 3.25224 0.54683<br>H 1.59976 3.27956 -1.08696<br>H 0.56931 3.71452 0.28862<br>S -1.99709 -0.59276 -0.58333<br>C -3.28279 0.57783 -0.09186<br>H -4.16399 0.31723 -0.68388<br>H -3.53665 0.46873 0.96677<br>H -3.01333 1.61321 -0.31164<br>H 2.12857 -2.52908 -0.22034	C_1	-858.83362 (6.8)	-858.65483 (6.7)	-858.70559 (6.4)	-859.43335 (7.7)	-859.30532 (7.3)

Table S5.2. Reduced neutral form in water.

Structure	Schematic drawing	Symmetry	G_{PCM}	$H_{PCM,RRHO}$	$G_{PCM,RRHO}$	$G_{SMD,M06\text{-}2X,large}$	$G_{SMD,RRHO,M06\text{-}2X,large}$
6-adduct, conf1	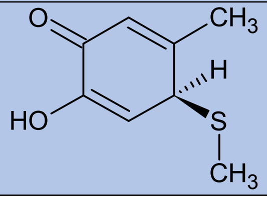	C_1	-858.85423 (0.0)	-858.67577 (0.0)	-858.72624 (0.0)	-859.45654 (0.0)	-859.32855 (0.0)

	(4*S*)-2-hydroxy-5-methyl-4-(methylsulfanyl)cyclohexa-2,5-dien-1-one<br><br>C -1.55510 -0.66387 -0.40072<br>C -1.73003 0.63135 0.28590<br>C -0.61517 1.56030 0.27870<br>C 0.56999 1.25796 -0.29708<br>C 0.79401 -0.09321 -0.91085<br>C -0.39034 -0.98800 -0.98567<br>H -0.78543 2.53067 0.73752<br>H 1.21646 0.04521 -1.91590<br>H -0.26919 -1.93907 -1.49605<br>O -2.64592 -1.45653 -0.40047<br>O -2.82674 0.86855 0.80959<br>C 1.69313 2.24098 -0.34573<br>H 2.00959 2.40945 -1.38164<br>H 1.40003 3.19726 0.09156<br>H 2.56746 1.85702 0.19126<br>S 2.18451 -0.98190 -0.05861<br>C 1.52793 -1.13971 1.61809<br>H 2.25945 -1.73053 2.17426<br>H 1.41919 -0.16523 2.09997<br>H 0.57203 -1.66880 1.61990<br>H -3.32705 -0.95659 0.08810						
6-adduct, conf2	C 1.46328 -0.98401 0.18032<br>C 2.04793 0.32671 -0.15684<br>C 1.18530 1.49101 -0.08568<br>C -0.12413 1.40103 0.23575<br>C -0.75047 0.06545 0.51958<br>C 0.16830 -1.10331 0.51515<br>H 1.64838 2.45427 -0.28181<br>H -1.26096 0.13498 1.49106<br>H -0.24084 -2.07558 0.77388<br>O 2.32584 -2.01902 0.12659<br>O 3.24686 0.36969 -0.46395<br>C -0.98823 2.61557 0.34689<br>H -1.50291 2.63507 1.31403<br>H -0.39729 3.52776 0.24365<br>H -1.76775 2.61342 -0.42280<br>S -2.12283 -0.19881 -0.70646<br>C -3.07108 -1.48724 0.13715<br>H -3.96218 -1.65552 -0.47218<br>H -2.51716 -2.42568 0.20778	C_1	-858.85142 (1.8)	-858.67292 (1.8)	-858.72372 (1.6)	-859.45288 (2.3)	-859.32517 (2.1)

	H -3.38173 -1.15612 1.13118<br>H 3.18069 -1.62829 -0.13650						
6-adduct, conf3	C 1.69466 -0.85042 0.20559<br>C 2.03845 0.53922 -0.15580<br>C 0.98106 1.53109 -0.11780<br>C -0.29319 1.21238 0.20106<br>C -0.67649 -0.20883 0.49963<br>C 0.43851 -1.19412 0.53147<br>H 1.26401 2.55582 -0.34308<br>H -1.20499 -0.23245 1.46314<br>H 0.19499 -2.21716 0.80240<br>O 2.73185 -1.71143 0.19188<br>O 3.21479 0.78911 -0.44996<br>C -1.36216 2.25339 0.27456<br>H -1.84188 2.24807 1.25996<br>H -0.95499 3.24950 0.09119<br>H -2.14411 2.05724 -0.46786<br>S -1.93574 -0.82738 -0.72202<br>C -3.48432 -0.62619 0.19523<br>H -4.26922 -1.03007 -0.44896<br>H -3.46448 -1.19806 1.12586<br>H -3.70574 0.42273 0.40172<br>H 3.50588 -1.17806 -0.07103	C_1	-858.85108 (2.0)	-858.67264 (2.0)	-858.72289 (2.1)	-859.45281 (2.3)	-859.32462 (2.5)
5-adduct, conf1	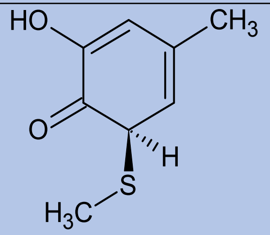<br>(6*R*)-2-hydroxy-4-methyl-6-(methylsulfanyl)cyclohexa-2,4-dien-1-one<br><br>C -0.40342 1.11701 -0.51817<br>C 0.85129 1.40559 0.17876<br>C 1.79666 0.44954 0.33928<br>C 1.63041 -0.90229 -0.17727<br>C 0.48600 -1.24920 -0.80158<br>C -0.65929 -0.30932 -0.95211<br>H 2.72066 0.70223 0.85422<br>H 0.34661 -2.25234 -1.19498<br>H -0.99274 -0.27208 -1.99771	C_1	-858.84692 (4.6)	-858.66858 (4.5)	-858.71902 (4.5)	-859.44853 (5.0)	-859.32063 (5.0)

	O -1.20410 2.03270 -0.70600<br>O 0.99510 2.67019 0.62241<br>C 2.77573 -1.85485 0.00038<br>H 3.68187 -1.46961 -0.48094<br>H 3.01014 -1.98817 1.06275<br>H 2.54754 -2.83373 -0.42755<br>S -2.15808 -0.96852 -0.09907<br>C -1.60376 -1.00280 1.62322<br>H -2.38054 -1.53113 2.18066<br>H -0.66415 -1.55185 1.72033<br>H -1.49853 0.00423 2.03331<br>H 0.18869 3.14355 0.34857						
5-adduct, conf2	C 0.40494 0.91221 0.49202<br>C -0.79257 1.42713 -0.18460<br>C -1.88254 0.64350 -0.34884<br>C -1.90508 -0.74572 0.09259<br>C -0.80070 -1.30804 0.62625<br>C 0.48816 -0.56881 0.73485<br>H -2.77746 1.06414 -0.80095<br>H -0.80096 -2.34638 0.94557<br>H 1.00477 -0.76138 1.68132<br>O 1.30270 1.69863 0.79823<br>O -0.74947 2.72614 -0.53680<br>C -3.19547 -1.49433 -0.06043<br>H -4.00234 -0.99596 0.48896<br>H -3.50028 -1.52984 -1.11269<br>H -3.10773 -2.51880 0.30825<br>S 1.56612 -1.24256 -0.61940<br>C 3.18609 -0.56063 -0.19528<br>H 3.87472 -0.94280 -0.95327<br>H 3.18248 0.53037 -0.22850<br>H 3.50892 -0.90571 0.78989<br>H 0.11715 3.05734 -0.23909	C_1	-858.84719 (4.4)	-858.66879 (4.4)	-858.71890 (4.6)	-859.44818 (5.2)	-859.31990 (5.4)
5-adduct, conf3	C 0.36450 -1.20852 0.36910<br>C 1.67616 -0.78243 -0.11636<br>C 1.97905 0.53106 -0.24879<br>C 1.02899 1.58173 0.08009<br>C -0.21323 1.26157 0.49959<br>C -0.68288 -0.14609 0.61557<br>H 2.97011 0.81146 -0.59786<br>H -0.92160 2.04313 0.75892<br>H -1.11693 -0.33845 1.60563<br>O 0.15092 -2.40936 0.53923	C_1	-858.84466 (6.0)	-858.66628 (6.0)	-858.71737 (5.6)	-859.44519 (7.1)	-859.31790 (6.7)

	O 2.54865 -1.77347 -0.38444<br>C 1.49369 3.00113 -0.06105<br>H 2.37010 3.18976 0.56935<br>H 1.79202 3.21113 -1.09469<br>H 0.70830 3.70641 0.22007<br>S -2.03348 -0.50196 -0.60553<br>C -3.32965 0.60215 0.00289<br>H -4.21902 0.37074 -0.58830<br>H -3.54716 0.40634 1.05586<br>H -3.07825 1.65413 -0.14335<br>H 2.08174 -2.60431 -0.18172						

Table S5.3. Reduced zwitterionic form in water.

Structure	Schematic drawing	Symmetry	G_{PCM}	$H_{PCM,RRHO}$	$G_{PCM,RRHO}$	$G_{SMD,M06\text{-}2X,large}$	$G_{SMD,RRHO,M06\text{-}2X,large}$
6-adduct, several starting structures	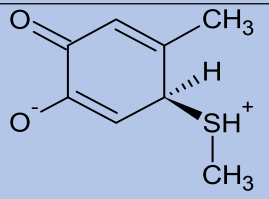(3*S*)-4-methyl-3-(methylsulfonio)-6-oxocyclohexa-1,4-dien-1-olate	C_1	dissociates to neutral molecules				
5-adduct, several structures	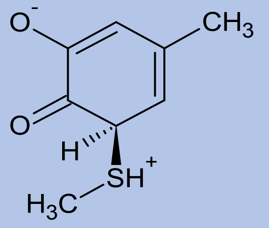(5*R*)-3-methyl-5-(methylsulfonio)-6-oxocyclohexa-1,3-dien-1-olate	C_1	most starting structures dissociate to neutral molecules				
5-adduct, conf1	C -0.17668 1.15578 -0.31652<br>C 1.26468 1.42696 0.04685<br>C 2.03139 0.27813 0.24212<br>C 1.60025 -1.06422 -0.04663<br>C 0.36458 -1.35119 -0.53752<br>C -0.54602 -0.22995 -0.85259<br>H 3.03587 0.41357 0.63903<br>H 0.10247 -2.35047 -0.87200	C_1	-858.78173 (1.5)	-858.60663 (1.9)	-858.65736 (1.8)	-859.38778 (0.0)	-859.26340 (0.0)

	H -0.66222 -0.10565 -1.94201<br>O -1.06124 1.98871 -0.16625<br>O 1.60837 2.62473 0.26032<br>C 2.60425 -2.16282 0.16358<br>H 3.48677 -2.00386 -0.46640<br>H 2.95278 -2.16805 1.20264<br>H 2.18583 -3.14547 -0.06698<br>S -2.30870 -0.48656 -0.31307<br>C -2.12040 -0.68871 1.46935<br>H -3.01564 -1.19116 1.83669<br>H -1.22294 -1.27695 1.67090<br>H -2.05080 0.31218 1.89466<br>H -2.38233 -1.79056 -0.65180						
5-adduct, conf2	C -0.15195 1.17622 -0.24746<br>C 1.29687 1.41372 0.08007<br>C 2.04674 0.24550 0.22360<br>C 1.58954 -1.08951 -0.07073<br>C 0.34009 -1.34473 -0.53435<br>C -0.55230 -0.19368 -0.81266<br>H 3.06441 0.35904 0.59348<br>H 0.03574 -2.33483 -0.85866<br>H -0.61375 -0.02890 -1.90287<br>O -1.01623 2.03359 -0.08420<br>O 1.67346 2.59974 0.31037<br>C 2.57805 -2.20669 0.11350<br>H 3.45274 -2.05833 -0.52986<br>H 2.94325 -2.22946 1.14658<br>H 2.13786 -3.17918 -0.11992<br>S -2.30919 -0.49353 -0.36562<br>C -2.19262 -0.92051 1.38449<br>H -3.21327 -0.96917 1.76604<br>H -1.72001 -1.90087 1.44557<br>H -1.61233 -0.17001 1.92064<br>H -2.51190 0.85956 -0.23916	C_1	-858.78414 (0.0)	-858.60963 (0.0)	-858.66021 (0.0)	-859.38699 (0.5)	-859.26306 (0.2)
5-adduct, conf3	C 0.14597 1.05103 0.05035<br>C -1.30601 1.46525 0.06021<br>C -2.20030 0.39752 -0.02961<br>C -1.83799 -0.99459 0.03080<br>C -0.55578 -1.41503 0.20030<br>C 0.49113 -0.38848 0.41687<br>H -3.25279 0.64103 -0.16376<br>H -0.31484 -2.45915 0.37490<br>H 0.80545 -0.36483 1.47353	C_1	-858.78110 (1.9)	-858.60598 (2.3)	-858.65656 (2.3)	-859.38618 (1.0)	-859.26165 (1.1)

	O 1.05479 1.81203 -0.25788<br>O -1.57629 2.70030 0.02246<br>C -2.95628 -1.99589 -0.04674<br>H -3.66004 -1.85276 0.78109<br>H -3.52526 -1.86345 -0.97396<br>H -2.58464 -3.02284 -0.01161<br>S 2.03309 -0.77150 -0.53164<br>C 3.40227 -0.20073 0.50061<br>H 4.31663 -0.61142 0.06981<br>H 3.40262 0.88612 0.43502<br>H 3.26797 -0.54008 1.52766<br>H 2.08943 -2.08193 -0.21975						
5-adduct, conf4	C 0.20749 -1.24187 0.25689<br>C 1.68704 -1.12803 -0.01281<br>C 2.15866 0.17558 -0.16946<br>C 1.38249 1.37214 0.02296<br>C 0.06232 1.34366 0.34426<br>C -0.60467 0.03782 0.53203<br>H 3.20693 0.29173 -0.43909<br>H -0.48943 2.25050 0.57156<br>H -1.02361 -0.07316 1.54239<br>O -0.38461 -2.31402 0.25267<br>O 2.34858 -2.19969 -0.13089<br>C 2.10438 2.68336 -0.10917<br>H 2.92459 2.74769 0.61474<br>H 2.55243 2.77459 -1.10526<br>H 1.43499 3.53244 0.04849<br>S -2.06255 -0.10919 -0.59932<br>C -3.34806 0.85181 0.22229<br>H -4.27477 0.67102 -0.32447<br>H -3.44317 0.54948 1.26493<br>H -3.07228 1.90305 0.13619<br>H -2.42461 -1.35735 -0.21663	C_1	-858.78135 (1.8)	-858.60625 (2.1)	-858.65697 (2.0)	-859.38662 (0.7)	-859.26224 (0.7)

Table S5.4. Reduced anionic form in water.

Structure	Schematic drawing	Symmetry	G_{PCM}	$H_{PCM,RRHO}$	$G_{PCM,RRHO}$	$G_{SMD,M06\text{-}2X,large}$	$G_{SMD,RRHO,M06\text{-}2X,large}$

6-adduct, conf1	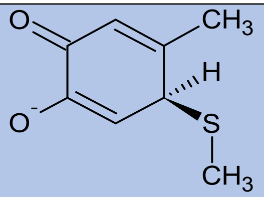<br>(3*S*)-4-methyl-3-(methylsulfanyl)-6-oxocyclohexa-1,4-dien-1-olate<br><br>C -1.66141 -0.72613 -0.43234<br>C -1.75764 0.59286 0.30607<br>C -0.62700 1.52451 0.24961<br>C 0.55273 1.23645 -0.33649<br>C 0.75986 -0.12219 -0.93080<br>C -0.43913 -0.98492 -1.02091<br>H -0.79464 2.49822 0.70799<br>H 1.24015 -0.00640 -1.91314<br>H -0.31269 -1.92823 -1.55035<br>O -2.69773 -1.47842 -0.46874<br>O -2.77839 0.90612 0.93139<br>C 1.66759 2.23088 -0.41105<br>H 1.95561 2.40929 -1.45425<br>H 1.37741 3.18442 0.03633<br>H 2.56034 1.85603 0.10202<br>S 2.15351 -0.98215 0.00089<br>C 1.41975 -1.12149 1.64520<br>H 2.03133 -1.82618 2.21327<br>H 1.40478 -0.15935 2.16308<br>H 0.40194 -1.51483 1.56396	C_1	-858.37067 (0.0)	-858.20606 (0.0)	-858.25647 (0.0)	-858.98089 (0.0)	-858.86669 (0.0)
6-adduct, conf2	C 1.50145 -1.10920 0.21534<br>C 2.06408 0.22725 -0.21567<br>C 1.22302 1.42195 -0.10649<br>C -0.07042 1.39916 0.27239<br>C -0.71718 0.08457 0.58589<br>C 0.17777 -1.09310 0.61256<br>H 1.71341 2.36588 -0.34026<br>H -1.26216 0.18718 1.53606<br>H -0.26120 -2.03178 0.94600<br>O 2.27757 -2.12734 0.19965<br>O 3.22419 0.33260 -0.63336<br>C -0.88021 2.65142 0.40866<br>H -1.32055 2.71965 1.41045<br>H -0.26340 3.53711 0.23880	C_1	-858.36829 (1.5)	-858.20352 (1.6)	-858.25422 (1.4)	-858.97745 (2.2)	-858.86339 (2.1)

	H -1.71500 2.66797 -0.30110<br>S -2.10576 -0.09910 -0.67373<br>C -2.93716 -1.56764 -0.02527<br>H -3.83184 -1.71513 -0.63506<br>H -2.30713 -2.45638 -0.10416<br>H -3.24223 -1.41748 1.01416						
6-adduct, conf3	C 1.81548 -0.93487 0.19596<br>C 2.09907 0.50802 -0.16115<br>C 1.00888 1.48418 -0.09467<br>C -0.26532 1.17209 0.21694<br>C -0.62401 -0.25096 0.52021<br>C 0.50482 -1.21583 0.52607<br>H 1.28663 2.51424 -0.31400<br>H -1.15952 -0.27050 1.48269<br>H 0.25283 -2.24016 0.79599<br>O 2.78916 -1.76723 0.18145<br>O 3.23299 0.87879 -0.48938<br>C -1.33459 2.21800 0.27719<br>H -1.87511 2.17892 1.22989<br>H -0.91078 3.21817 0.16033<br>H -2.07280 2.06676 -0.51928<br>S -1.91262 -0.87537 -0.68638<br>C -3.47996 -0.48975 0.14035<br>H -4.26358 -0.98114 -0.44200<br>H -3.49118 -0.89948 1.15406<br>H -3.68801 0.58138 0.17242	C_1	-858.36772 (1.9)	-858.20304 (1.9)	-858.25312 (2.1)	-858.97774 (2.0)	-858.86314 (2.2)
5-adduct, conf1	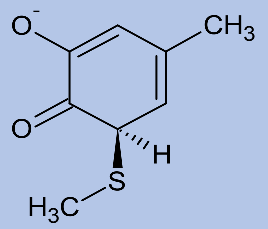<br>(5*R*)-3-methyl-5-(methylsulfanyl)-6-oxocyclohexa-1,3-dien-1-olate<br><br>C -0.52746 1.12489 -0.57001<br>C 0.63023 1.55575 0.30640<br>C 1.67470 0.64028 0.38916<br>C 1.68054 -0.66621 -0.23039<br>C 0.60046 -1.14626 -0.89404<br>C -0.64154 -0.33997 -0.98109	C_1	-858.36924 (0.9)	-858.20466 (0.9)	-858.25478 (1.1)	-858.97705 (2.4)	-858.86258 (2.6)

	H 2.55924 0.93981 0.95094<br>H 0.59672 -2.13880 -1.33709<br>H -1.07255 -0.35148 -1.98975<br>O -1.37897 1.92457 -0.94564<br>O 0.58677 2.71078 0.84388<br>C 2.94160 -1.47707 -0.10927<br>H 2.84515 -2.45004 -0.59895<br>H 3.79094 -0.94788 -0.55806<br>H 3.19743 -1.64541 0.94394<br>S -2.02465 -1.10855 0.00046<br>C -1.30409 -1.19872 1.65771<br>H -1.93769 -1.87278 2.23888<br>H -0.29448 -1.61451 1.60210<br>H -1.27941 -0.22109 2.14387						
5-adduct, conf2	C 0.42589 0.91793 0.55986<br>C -0.70219 1.52710 -0.25305<br>C -1.82850 0.71905 -0.37977<br>C -1.91569 -0.64069 0.09680<br>C -0.85286 -1.27699 0.65251<br>C 0.45723 -0.58458 0.75288<br>H -2.70443 1.14779 -0.86582<br>H -0.91160 -2.31307 0.97450<br>H 1.00398 -0.81545 1.67385<br>O 1.31818 1.61195 1.04121<br>O -0.57110 2.72301 -0.66731<br>C -3.24026 -1.33766 -0.05277<br>H -3.20465 -2.36111 0.33047<br>H -4.02911 -0.79578 0.48266<br>H -3.54313 -1.37475 -1.10618<br>S 1.50297 -1.21987 -0.65225<br>C 3.14245 -0.55136 -0.27520<br>H 3.80110 -0.88459 -1.08142<br>H 3.13214 0.54038 -0.24603<br>H 3.51528 -0.94189 0.67524	C_1	-858.37072 (0.0)	-858.20614 (0.0)	-858.25596 (0.3)	-858.97761 (2.1)	-858.86285 (2.4)
5-adduct, conf3	C 0.36641 -1.25965 0.47343<br>C 1.64375 -0.91415 -0.25961<br>C 1.91505 0.44820 -0.35909<br>C 1.05207 1.49747 0.12599<br>C -0.16832 1.22971 0.65562<br>C -0.66512 -0.16474 0.72497<br>H 2.86201 0.73679 -0.81470<br>H -0.81400 2.02713 1.01244<br>H -1.16429 -0.38825 1.67508	C_1	-858.36750 (2.0)	-858.20288 (2.0)	-858.25340 (1.9)	-858.97426 (4.2)	-858.86015 (4.1)

	O 0.12628 -2.40626 0.83993<br>O 2.38545 -1.86805 -0.66114<br>C 1.55867 2.90974 0.01910<br>H 0.83862 3.62848 0.42003<br>H 2.50388 3.02911 0.56209<br>H 1.76056 3.17277 -1.02642<br>S -1.94280 -0.51443 -0.59723<br>C -3.15748 0.77668 -0.23908<br>H -3.99817 0.60164 -0.91523<br>H -3.51409 0.70486 0.79198<br>H -2.75379 1.77333 -0.42794						

Table S5.5. Reduced cationic form in water.

Structure	Schematic drawing	Symmetry	G_{PCM}	$H_{PCM,RRHO}$	$G_{PCM,RRHO}$	$G_{SMD,M06\text{-}2X,large}$	$G_{SMD,RRHO,M06\text{-}2X,large}$
6-adduct, conf1	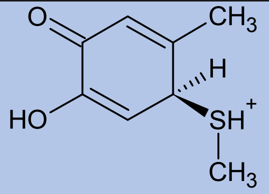<br>[(1*S*)-5-hydroxy-2-methyl-4-oxocyclohexa-2,5-dien-1-yl](methyl)sulfonium<br><br>C -1.55764 -0.70037 -0.39759<br>C -1.80434 0.58740 0.30241<br>C -0.73299 1.57239 0.31960<br>C 0.47316 1.33516 -0.23215<br>C 0.73796 0.00921 -0.90008<br>C -0.37092 -0.96759 -0.96972<br>H -0.95794 2.52977 0.78031<br>H 1.16735 0.18843 -1.89551<br>H -0.21284 -1.89322 -1.51565<br>O -2.60107 -1.53167 -0.43325<br>O -2.91189 0.76587 0.80637<br>C 1.56136 2.35904 -0.25230<br>H 1.93308 2.50869 -1.27177<br>H 1.19882 3.31244 0.13428<br>H 2.42009 2.05438 0.35791<br>S 2.23950 -0.73835 -0.03303<br>C 1.57907 -1.40171 1.50551	C_1	-859.25302 (0.0)	-859.06355 (0.0)	-859.11378 (0.0)	-859.85974 (0.5)	-859.72050 (0.8)

	H 2.36393 -2.03058 1.92897<br>H 1.38744 -0.55605 2.16635<br>H 0.67315 -1.97698 1.31597<br>H -3.32580 -1.08156 0.04190<br>H 2.31052 -1.87653 -0.75337						
6-adduct, conf2	C -1.58696 -0.69633 -0.36995<br>C -1.78079 0.61855 0.29224<br>C -0.67794 1.56949 0.26082<br>C 0.51350 1.27874 -0.29878<br>C 0.72698 -0.07081 -0.92576<br>C -0.41900 -1.01744 -0.95064<br>H -0.86982 2.54965 0.68762<br>H 1.14252 0.05306 -1.93528<br>H -0.29147 -1.96548 -1.46468<br>O -2.65553 -1.49671 -0.36248<br>O -2.87259 0.85079 0.80816<br>C 1.62369 2.27875 -0.36660<br>H 2.09598 2.28294 -1.35398<br>H 1.24566 3.28041 -0.15556<br>H 2.40949 2.07573 0.37228<br>S 2.12143 -1.01025 -0.08881<br>C 1.63403 -1.06272 1.64474<br>H 2.50182 -1.42836 2.19589<br>H 1.33219 -0.07743 1.99981<br>H 0.81793 -1.78151 1.72144<br>H -3.35820 -1.01093 0.11006<br>H 3.05485 -0.03606 -0.03850	C_1	-859.25183 (0.8)	-859.06215 (0.9)	-859.11329 (0.3)	-859.86031 (0.1)	-859.72177 (0.0)
6-adduct, conf3	C -1.56679 -0.70556 -0.38594<br>C -1.80383 0.59218 0.29871<br>C -0.72543 1.56984 0.30311<br>C 0.47725 1.32007 -0.25083<br>C 0.73029 -0.01305 -0.90743<br>C -0.38318 -0.98591 -0.95807<br>H -0.94237 2.53276 0.75608<br>H 1.15171 0.15317 -1.90855<br>H -0.23167 -1.91784 -1.49502<br>O -2.61495 -1.53131 -0.40901<br>O -2.90916 0.78419 0.80248<br>C 1.57145 2.33751 -0.28311<br>H 1.21630 3.29494 0.10045<br>H 2.43260 2.03326 0.32433<br>H 1.93844 2.47952 -1.30528<br>S 2.23425 -0.76647 -0.04934	C_1	-859.25304 (0.0)	-859.06342 (0.1)	-859.11296 (0.5)	-859.85997 (0.3)	-859.71988 (1.2)

	C 1.60969 -1.30162 1.55358<br>H 2.36390 -1.97258 1.96819<br>H 1.52209 -0.41326 2.17948<br>H 0.65219 -1.81023 1.44019<br>H -3.33615 -1.07128 0.06189<br>H 2.22115 -1.94859 -0.70020						
6-adduct, conf4	C -1.60219 -0.71264 -0.34265<br>C -1.79580 0.61994 0.28275<br>C -0.69046 1.56710 0.23012<br>C 0.50121 1.26042 -0.32030<br>C 0.71029 -0.10121 -0.92152<br>C -0.43358 -1.05071 -0.91213<br>H -0.87886 2.55617 0.63745<br>H 1.10825 0.00470 -1.94060<br>H -0.30642 -2.01253 -1.39991<br>O -2.67077 -1.51268 -0.31338<br>O -2.88814 0.86749 0.79045<br>C 1.61746 2.25171 -0.40191<br>H 1.25079 3.25662 -0.18662<br>H 2.40996 2.04070 0.32753<br>H 2.07869 2.25154 -1.39465<br>S 2.12132 -1.01895 -0.09354<br>C 1.72539 -0.94731 1.66275<br>H 2.58220 -1.37343 2.18676<br>H 1.53931 0.07696 1.98482<br>H 0.84899 -1.57867 1.81312<br>H -3.37393 -1.01365 0.14429<br>H 3.07735 -0.06749 -0.15472	C_1	-859.25187 (0.7)	-859.06250 (0.7)	-859.11320 (0.4)	-859.86048 (0.0)	-859.72181 (0.0)
6-adduct, conf5	C -1.56001 -0.70237 -0.39395<br>C -1.80555 0.58861 0.30045<br>C -0.73285 1.57212 0.31445<br>C 0.47302 1.33146 -0.23644<br>C 0.73571 0.00378 -0.90142<br>C -0.37354 -0.97309 -0.96496<br>H -0.95611 2.53099 0.77287<br>H 1.16179 0.18006 -1.89883<br>H -0.21661 -1.90095 -1.50742<br>O -2.60415 -1.53291 -0.42595<br>O -2.91303 0.77044 0.80335<br>C 1.56354 2.35295 -0.25658<br>H 1.94123 2.49699 -1.27460<br>H 1.20122 3.30893 0.12380<br>H 2.41816 2.04930 0.35998	C_1	-859.25302 (0.0)	saddle point			

	S 2.23986 -0.74119 -0.03726<br>C 1.58720 -1.38159 1.51438<br>H 2.36926 -2.01418 1.93747<br>H 1.41080 -0.52648 2.16752<br>H 0.67368 -1.94943 1.33893<br>H -3.32849 -1.07995 0.04708<br>H 2.29958 -1.88763 -0.74546						
6-adduct, conf6	C 1.44881 -0.98208 0.21759<br>C 2.03182 0.31352 -0.21637<br>C 1.19439 1.50032 -0.13299<br>C -0.09459 1.44839 0.25611<br>C -0.70998 0.12233 0.62300<br>C 0.17161 -1.07087 0.62654<br>H 1.66607 2.44726 -0.37850<br>H -1.25758 0.20985 1.56918<br>H -0.22592 -2.01140 0.99375<br>O 2.29352 -2.01412 0.19971<br>O 3.20332 0.32165 -0.59084<br>C -0.94443 2.67462 0.36744<br>H -1.51890 2.67689 1.29933<br>H -0.32364 3.57156 0.33771<br>H -1.65895 2.75443 -0.46172<br>S -2.05407 -0.20821 -0.65461<br>C -2.89683 -1.67030 -0.01270<br>H -3.84599 -1.73447 -0.54746<br>H -2.28119 -2.53501 -0.25739<br>H -3.06212 -1.57952 1.06051<br>H 3.14600 -1.66460 -0.12413<br>H -2.95200 0.72052 -0.26025	C_1	-859.25040 (1.7)	-859.06060 (1.9)	-859.11054 (2.0)	-859.85844 (1.3)	-859.71858 (2.0)
6-adduct, conf7	C 1.44497 -0.97826 0.24790<br>C 2.01800 0.30158 -0.24357<br>C 1.18439 1.49255 -0.17694<br>C -0.09317 1.45420 0.24934<br>C -0.70815 0.13184 0.64291<br>C 0.17514 -1.05554 0.68233<br>H 1.65342 2.43244 -0.45236<br>H -1.25460 0.25328 1.58774<br>H -0.21160 -1.98426 1.08843<br>O 2.29116 -2.01045 0.24870<br>O 3.18084 0.29618 -0.64447<br>C -0.92766 2.68461 0.39770<br>H -1.25595 2.79481 1.43749<br>H -0.35992 3.57209 0.11476	C_1	-859.25168 (0.9)	-859.06228 (0.8)	-859.11279 (0.6)	-859.85789 (1.6)	-859.71900 (1.8)

	H -1.83305 2.64770 -0.21803<br>S -2.13597 -0.12413 -0.56603<br>C -2.90256 -1.68047 -0.07447<br>H -3.67398 -1.87967 -0.82035<br>H -2.17431 -2.48897 -0.04806<br>H -3.36604 -1.51921 0.89920<br>H 3.13673 -1.67095 -0.10244<br>H -1.40675 -0.56016 -1.61490						
6-adduct, conf8	C 1.74007 -0.71768 0.27227<br>C 1.87374 0.61436 -0.37755<br>C 0.75279 1.53635 -0.27474<br>C -0.40731 1.19612 0.32410<br>C -0.60789 -0.21539 0.80296<br>C 0.58963 -1.09686 0.85514<br>H 0.92104 2.54845 -0.63076<br>H -1.15522 -0.23189 1.75231<br>H 0.49663 -2.06276 1.34161<br>O 2.84300 -1.46747 0.25157<br>O 2.94611 0.88697 -0.91410<br>C -1.45727 2.20960 0.64332<br>H -1.38583 2.46069 1.70946<br>H -1.30798 3.12469 0.06796<br>H -2.47536 1.85218 0.48231<br>S -1.75503 -1.21882 -0.31671<br>C -3.30191 -0.35123 -0.65864<br>H -3.86773 -1.03415 -1.29663<br>H -3.82700 -0.22361 0.28852<br>H -3.13095 0.59420 -1.16906<br>H 3.51984 -0.94594 -0.22124<br>H -1.11093 -0.98828 -1.47801	C_1	-859.24736 (3.6)	-859.05758 (3.7)	-859.10685 (4.3)	-859.85378 (4.2)	-859.71328 (5.4)
6-adduct, conf9	C 1.74103 -0.81646 0.21182<br>C 2.02752 0.57828 -0.21167<br>C 0.94768 1.54876 -0.13088<br>C -0.30147 1.21220 0.24560<br>C -0.61006 -0.21353 0.62331<br>C 0.51354 -1.18388 0.61688<br>H 1.19760 2.57702 -0.37545<br>H -1.14611 -0.24266 1.57977<br>H 0.32703 -2.19299 0.97226<br>O 2.79034 -1.63919 0.19036<br>O 3.17089 0.84615 -0.57704<br>C -1.39675 2.22713 0.33284<br>H -2.01312 2.08319 1.22530	C_1	-859.24911 (2.5)	-859.05930 (2.7)	-859.10896 (3.0)	-859.85702 (2.2)	-859.71688 (3.1)

	H -0.97893 3.23503 0.35667<br>H -2.05618 2.17928 -0.54228<br>S -1.85704 -0.89503 -0.62133<br>C -3.50877 -0.44893 -0.03170<br>H -4.19638 -1.13200 -0.53467<br>H -3.57478 -0.56297 1.04996<br>H -3.71757 0.57245 -0.34381<br>H 3.54710 -1.10903 -0.12610<br>H -1.84220 -2.16398 -0.16422						
6-adduct, conf10	C 1.75537 -0.77635 0.22771<br>C 1.97508 0.60549 -0.26826<br>C 0.87270 1.54792 -0.16381<br>C -0.34785 1.19561 0.28865<br>C -0.60039 -0.22880 0.70197<br>C 0.56048 -1.16090 0.70576<br>H 1.08674 2.57654 -0.43981<br>H -1.12042 -0.25435 1.66817<br>H 0.40942 -2.16243 1.09644<br>O 2.82883 -1.56911 0.18799<br>O 3.08912 0.89285 -0.70400<br>C -1.44292 2.20675 0.43783<br>H -1.01597 3.19020 0.64587<br>H -2.02084 2.30651 -0.48833<br>H -2.13171 1.95189 1.24702<br>S -1.82673 -1.12303 -0.41667<br>C -3.45976 -0.36029 -0.30914<br>H -4.08803 -0.93970 -0.98884<br>H -3.81153 -0.49512 0.71393<br>H -3.44639 0.68686 -0.59776<br>H 3.55163 -1.03082 -0.18771<br>H -1.40511 -0.62759 -1.59837	C_1	-859.24749 (3.5)	-859.05784 (3.6)	-859.10797 (3.6)	-859.85398 (4.1)	-859.71446 (4.6)
6-adduct, conf11	C 1.72576 -0.84418 0.20533<br>C 2.05396 0.55124 -0.18407<br>C 0.99264 1.54375 -0.11951<br>C -0.27042 1.22857 0.22626<br>C -0.61766 -0.19444 0.58261<br>C 0.48115 -1.19171 0.57309<br>H 1.26793 2.56787 -0.35365<br>H -1.15881 -0.22159 1.53683<br>H 0.26420 -2.20277 0.90552<br>O 2.75656 -1.69101 0.19264<br>O 3.21243 0.80032 -0.51318<br>C -1.35158 2.25803 0.29701	C_1	-859.24918 (2.4)	-859.05975 (2.4)	-859.10993 (2.4)	-859.85711 (2.1)	-859.71785 (2.5)

	H -1.89919 2.19760 1.24310<br>H -0.93188 3.26060 0.20155<br>H -2.07777 2.13744 -0.51687<br>S -1.90383 -0.78934 -0.67295<br>C -3.51720 -0.54910 0.10786<br>H -4.22927 -1.11384 -0.49683<br>H -3.50390 -0.91443 1.13433<br>H -3.75959 0.51130 0.06202<br>H 3.53328 -1.17250 -0.09291<br>H -1.77535 -2.10765 -0.42313						
5-adduct, conf1	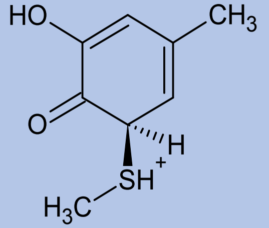<br>[(1*R*)-5-hydroxy-3-methyl-6-oxocyclohexa-2,4-dien-1-yl](methyl)sulfonium<br><br>C -0.19545 1.12290 -0.34123<br>C 1.18669 1.32119 0.07293<br>C 2.01481 0.25752 0.22011<br>C 1.61381 -1.10921 -0.08338<br>C 0.37552 -1.37640 -0.54608<br>C -0.57360 -0.26606 -0.83418<br>H 3.01891 0.42894 0.59940<br>H 0.08028 -2.38355 -0.82444<br>H -0.67696 -0.16786 -1.92857<br>O -1.00913 2.04114 -0.31014<br>O 1.54464 2.57431 0.39400<br>C 2.63616 -2.18448 0.12312<br>H 3.51418 -2.00511 -0.50694<br>H 2.98159 -2.18921 1.16268<br>H 2.23265 -3.17038 -0.11509<br>S -2.28692 -0.65114 -0.30934<br>C -2.14371 -0.80635 1.48330<br>H -3.15931 -0.93154 1.86134<br>H -1.55916 -1.70589 1.67905<br>H -1.67545 0.07825 1.91332<br>H 0.77278 3.14899 0.25922<br>H -2.70848 0.63726 -0.38880	C_1	-859.24781 (3.3)	-859.05890 (2.9)	-859.10918 (2.9)	-859.85200 (5.3)	-859.71337 (5.3)

5-adduct, conf2	C -0.12233 1.17939 -0.37525<br>C 1.25249 1.25974 0.11352<br>C 1.99764 0.13896 0.26280<br>C 1.51429 -1.18273 -0.11546<br>C 0.27423 -1.34742 -0.62105<br>C -0.60843 -0.17875 -0.86895<br>H 2.99568 0.22436 0.68429<br>H -0.06949 -2.32245 -0.95418<br>H -0.78546 -0.06071 -1.94985<br>O -0.84308 2.16952 -0.38189<br>O 1.67853 2.47886 0.47572<br>C 2.46231 -2.32896 0.05659<br>H 3.36416 -2.17287 -0.54551<br>H 2.78335 -2.40447 1.10135<br>H 2.00546 -3.27540 -0.23840<br>S -2.33087 -0.37046 -0.22229<br>C -2.08491 -0.86240 1.49646<br>H -3.03546 -1.27553 1.83711<br>H -1.28455 -1.59965 1.56574<br>H -1.85131 0.04141 2.05920<br>H 0.95110 3.10367 0.31284<br>H -2.57487 -1.58503 -0.75848	C_1	-859.24583 (4.5)	-859.05667 (4.3)	-859.10757 (3.9)	-859.85086 (6.0)	-859.71260 (5.8)
5-adduct, conf3	C 0.16637 1.02035 0.14222<br>C -1.25044 1.34809 0.01327<br>C -2.17623 0.36183 -0.05689<br>C -1.83184 -1.04907 0.05885<br>C -0.55371 -1.43676 0.24770<br>C 0.52263 -0.42550 0.43824<br>H -3.21633 0.62911 -0.22380<br>H -0.30166 -2.48326 0.39017<br>H 0.86885 -0.45591 1.48343<br>O 1.02864 1.88365 0.02048<br>O -1.54782 2.64769 -0.13609<br>C -2.95654 -2.03491 -0.01921<br>H -3.68024 -1.85466 0.78325<br>H -3.49450 -1.92579 -0.96727<br>H -2.59548 -3.06187 0.06159<br>S 2.01214 -0.80136 -0.58645<br>C 3.41206 -0.25816 0.41824<br>H 4.30987 -0.61113 -0.09177<br>H 3.37996 0.83060 0.42554<br>H 3.34160 -0.67353 1.42294<br>H -0.71334 3.14600 -0.10684	C_1	-859.24517 (4.9)	-859.05594 (4.8)	-859.10655 (4.5)	-859.84952 (6.9)	-859.71090 (6.8)

	H 2.06659 -2.12356 -0.32369						
5-adduct, conf4	C 0.31440 0.97197 0.44302<br>C -0.94894 1.42961 -0.13750<br>C -1.97672 0.56837 -0.32359<br>C -1.89746 -0.83607 0.05737<br>C -0.75538 -1.35453 0.55473<br>C 0.46260 -0.51483 0.72412<br>H -2.90512 0.93934 -0.74957<br>H -0.69225 -2.39338 0.86314<br>H 0.91185 -0.64051 1.71718<br>O 1.21645 1.76949 0.67077<br>O -1.01500 2.73220 -0.44894<br>C -3.13949 -1.65662 -0.10043<br>H -3.95869 -1.22955 0.48839<br>H -3.46409 -1.65805 -1.14690<br>H -2.98137 -2.68864 0.21804<br>S 1.74247 -1.26469 -0.39944<br>C 3.25175 -0.28461 -0.26409<br>H 3.90597 -0.64772 -1.05926<br>H 3.04092 0.77782 -0.37098<br>H 3.68621 -0.51030 0.70993<br>H -0.16537 3.13541 -0.19999<br>H 1.26627 -0.80264 -1.57546	C_1	-859.24517 (4.9)	-859.05599 (4.7)	-859.10613 (4.8)	-859.84907 (7.2)	-859.71003 (7.4)
5-adduct, conf5	C 0.35749 -1.21421 0.30853<br>C 1.72139 -0.83975 -0.06012<br>C 2.06670 0.46114 -0.20445<br>C 1.13529 1.55200 0.04939<br>C -0.14311 1.30514 0.40414<br>C -0.63449 -0.08426 0.58169<br>H 3.07933 0.70556 -0.51388<br>H -0.81627 2.12094 0.64608<br>H -1.00830 -0.24377 1.60374<br>O 0.03154 -2.39178 0.38209<br>O 2.56899 -1.85491 -0.28372<br>C 1.66950 2.94506 -0.07785<br>H 2.50345 3.10075 0.61535<br>H 2.05762 3.11286 -1.08865<br>H 0.89975 3.69092 0.12876<br>S -2.12491 -0.49297 -0.44388<br>C -3.35278 0.73403 0.04360<br>H -4.19775 0.59289 -0.63252<br>H -3.65428 0.49724 1.06417<br>H -2.95879 1.74520 -0.03814	C_1	-859.24437 (5.4)	-859.05522 (5.2)	-859.10649 (4.6)	-859.84922 (7.1)	-859.71135 (6.6)

	H 2.07862 -2.68227 -0.13778<br>H -1.70920 0.06580 -1.60153						
5-adduct, conf6	C 0.07246 -1.13938 0.13935<br>C 1.52589 -1.14059 0.07435<br>C 2.20852 0.02799 -0.01187<br>C 1.55940 1.33035 0.01566<br>C 0.22139 1.42660 0.15183<br>C -0.60542 0.20752 0.36596<br>H 3.28848 -0.00975 -0.12945<br>H -0.26957 2.39246 0.21544<br>H -0.93640 0.18059 1.41712<br>O -0.57157 -2.18076 0.04451<br>O 2.12450 -2.34054 0.00317<br>C 2.43878 2.53755 -0.10262<br>H 3.16242 2.56789 0.71922<br>H 3.01222 2.50383 -1.03545<br>H 1.85498 3.45986 -0.08582<br>S -2.15237 0.19249 -0.62919<br>C -3.44518 0.70708 0.52230<br>H -4.39286 0.52210 0.01369<br>H -3.38045 0.13643 1.44792<br>H -3.32373 1.77575 0.69785<br>H 1.43277 -3.02212 0.02978<br>H -2.28844 -1.15920 -0.53439	C_1	-859.24596 (4.4)	-859.05703 (4.1)	-859.10723 (4.1)	-859.84796 (7.9)	-859.70924 (7.9)
5-adduct, conf7	C 0.02395 -1.11620 0.07787<br>C 1.47656 -1.18660 0.10192<br>C 2.21567 -0.05069 0.04220<br>C 1.62698 1.27998 0.01570<br>C 0.29015 1.43803 0.09335<br>C -0.59161 0.25800 0.31053<br>H 3.29728 -0.13945 -0.02007<br>H -0.16197 2.42424 0.11484<br>H -0.88869 0.23951 1.37321<br>O -0.66387 -2.12085 -0.08255<br>O 2.02352 -2.41288 0.06347<br>C 2.56458 2.44361 -0.09407<br>H 3.25018 2.46671 0.76000<br>H 3.17800 2.35796 -0.99760<br>H 2.02277 3.39069 -0.12998<br>S -2.17073 0.32621 -0.62573<br>C -3.42060 0.64409 0.63900<br>H -4.38792 0.53837 0.14565<br>H -3.32018 -0.06464 1.46032	C_1	-859.24585 (4.5)	-859.05706 (4.1)	-859.10766 (3.8)	-859.84744 (8.2)	-859.70925 (7.9)

	H -3.28713 1.67246 0.97538<br>H 1.30256 -3.06354 0.05484<br>H -2.26456 -1.03329 -0.69133						
5-adduct, conf8	C 0.38872 -1.21489 0.33832<br>C 1.72223 -0.80078 -0.09469<br>C 2.02364 0.50993 -0.24843<br>C 1.07681 1.57282 0.06177<br>C -0.17860 1.28733 0.46656<br>C -0.62742 -0.11511 0.64360<br>H 3.01308 0.78411 -0.60446<br>H -0.86077 2.07979 0.75601<br>H -0.98626 -0.29051 1.66785<br>O 0.10063 -2.40186 0.42356<br>O 2.58656 -1.79066 -0.36247<br>C 1.57088 2.98086 -0.06090<br>H 2.42897 3.14516 0.60011<br>H 1.91224 3.17666 -1.08350<br>H 0.79206 3.70283 0.19143<br>S -2.11726 -0.57651 -0.36903<br>C -3.28321 0.77682 -0.12256<br>H -4.13491 0.55178 -0.76708<br>H -3.59519 0.75056 0.92169<br>H -2.83852 1.73357 -0.38966<br>H 2.12689 -2.63227 -0.19913<br>H -1.63724 -0.21931 -1.57953	C_1	-859.24425 (5.5)	-859.05484 (5.5)	-859.10538 (5.3)	-859.84912 (7.1)	-859.71026 (7.2)

Table S5.6. One-electron oxidized neutral form in vacuo.

Structure	Schematic drawing	Symmetry	E	H_{RRHO}	G_{RRHO}	$E_{M06\text{-}2X,large}$	$G_{RRHO,M06\text{-}2X,large}$
6-adduct, conf1	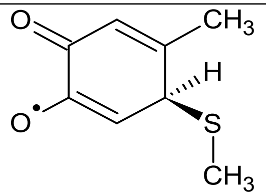<br>[(3*S*)-4-methyl-3-(methylsulfanyl)-6-oxocyclohexa-1,4-dien-1-yl]oxidanyl<br><br>C -1.59983 -0.66497 -0.49726<br>C -1.73140 0.61771 0.33205<br>C -0.59767 1.55087 0.30227	C_1	-858.18922 (6.1)	-858.02408 (6.1)	-858.07527 (6.3)	-858.78554 (4.6)	-858.67159 (4.8)

	C 0.58147 1.28595 -0.29700<br>C 0.83706 -0.05008 -0.93055<br>C -0.35251 -0.89662 -1.14367<br>H -0.76940 2.50680 0.79218<br>H 1.39133 0.07674 -1.87024<br>H -0.25639 -1.78931 -1.75623<br>O -2.55630 -1.44279 -0.60864<br>O -2.74766 0.84780 0.95875<br>C 1.69633 2.28094 -0.34747<br>H 1.90579 2.56367 -1.38701<br>H 1.44898 3.18648 0.21014<br>H 2.61887 1.85318 0.05974<br>S 2.06162 -1.04349 0.06496<br>C 1.17323 -1.27806 1.62287<br>H 1.88457 -1.76715 2.29307<br>H 0.87925 -0.32242 2.06414<br>H 0.30256 -1.92679 1.50643						
6-adduct, conf2	C 1.48808 -1.10269 0.25591<br>C 2.08353 0.21958 -0.23628<br>C 1.24135 1.41745 -0.11360<br>C -0.04500 1.40017 0.28675<br>C -0.74211 0.10248 0.58819<br>C 0.13408 -1.08049 0.69870<br>H 1.73280 2.35658 -0.35720<br>H -1.37602 0.22035 1.47696<br>H -0.28003 -2.01457 1.06969<br>O 2.17699 -2.12843 0.29316<br>O 3.21542 0.26538 -0.67663<br>C -0.85282 2.64905 0.44547<br>H -1.14399 2.78291 1.49513<br>H -0.28871 3.52988 0.13257<br>H -1.77690 2.59455 -0.14003<br>S -1.92342 -0.25273 -0.81194<br>C -3.14153 -1.27966 0.04267<br>H -3.90383 -1.53147 -0.69873<br>H -2.70376 -2.21056 0.41108<br>H -3.61763 -0.73481 0.86245	C_1	-858.18584 (8.2)	-858.02075 (8.2)	-858.07257 (8.0)	-858.78085 (7.5)	-858.66758 (7.4)

5-adduct, conf1	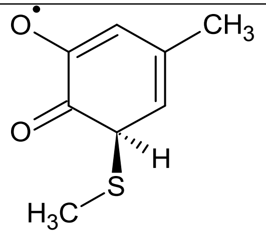<br><br>[(5*R*)-3-methyl-5-(methylsulfanyl)-6-oxocyclohexa-1,3-dien-1-yl]oxidanyl<br><br>C -0.66373 1.06199 -0.65351<br>C 0.42522 1.56812 0.30534<br>C 1.61039 0.75738 0.41619<br>C 1.75634 -0.49777 -0.20838<br>C 0.70249 -1.05262 -0.90858<br>C -0.63474 -0.41709 -1.00085<br>H 2.42332 1.17549 1.00561<br>H 0.81577 -2.02665 -1.37767<br>H -1.06831 -0.52924 -2.00111<br>O -1.49150 1.81326 -1.10906<br>O 0.27453 2.63114 0.90032<br>C 3.07179 -1.21938 -0.09860<br>H 3.87693 -0.62643 -0.54515<br>H 3.33776 -1.38860 0.95023<br>H 3.04273 -2.18823 -0.60252<br>S -1.84574 -1.35370 0.03859<br>C -1.23060 -1.06816 1.71654<br>H -1.82244 -1.72442 2.35951<br>H -0.17849 -1.34698 1.81471<br>H -1.38264 -0.03579 2.03988	C_1	-858.19538 (2.2)	-858.03020 (2.3)	-858.08171 (2.3)	-858.79012 (1.7)	-858.67646 (1.8)
5-adduct, conf2	C -0.17679 1.17947 -0.17138<br>C 1.34031 1.37608 0.00751<br>C 2.13310 0.18433 0.15569<br>C 1.61142 -1.11251 -0.00447<br>C 0.30443 -1.29139 -0.41409<br>C -0.61726 -0.16521 -0.75595<br>H 3.17492 0.33121 0.43109<br>H -0.08852 -2.29500 -0.55587<br>H -0.56186 -0.01097 -1.84943<br>O -0.94490 2.05473 0.13607<br>O 1.79886 2.51122 0.07897<br>C 2.50478 -2.29731 0.24355	C_1	-858.19327 (3.5)	-858.02864 (3.2)	-858.08049 (3.0)	-858.78702 (3.7)	-858.67425 (3.2)

	H 3.37111 -2.27568 -0.42604<br>H 2.88693 -2.28807 1.26966<br>H 1.97610 -3.23992 0.08431<br>S -2.37396 -0.56175 -0.49584<br>C -2.45282 -0.55347 1.31152<br>H -3.46083 -0.88233 1.57464<br>H -1.73413 -1.25262 1.74807<br>H -2.29508 0.45382 1.70242						
5-adduct, conf3	C 0.49207 0.91631 0.60995<br>C -0.61482 1.51819 -0.26835<br>C -1.80907 0.72171 -0.41842<br>C -1.94531 -0.58001 0.10259<br>C -0.86824 -1.20457 0.70108<br>C 0.47946 -0.58542 0.76048<br>H -2.63875 1.19636 -0.93703<br>H -0.97017 -2.21512 1.08738<br>H 1.03267 -0.87851 1.65889<br>O 1.34575 1.60832 1.11867<br>O -0.49113 2.64381 -0.73595<br>C -3.27115 -1.27963 -0.01563<br>H -4.06366 -0.69681 0.46491<br>H -3.54889 -1.40211 -1.06795<br>H -3.24660 -2.26954 0.44581<br>S 1.43034 -1.19465 -0.71043<br>C 3.10008 -0.66368 -0.26436<br>H 3.73490 -0.91348 -1.11812<br>H 3.13772 0.41498 -0.09146<br>H 3.46244 -1.19592 0.61871	C_1	-858.19889 (0.0)	-858.03378 (0.0)	-858.08534 (0.0)	-858.79286 (0.0)	-858.67931 (0.0)
5-adduct, conf4	C 0.22873 -1.27656 -0.04484<br>C 1.73353 -1.06639 0.19603<br>C 2.20791 0.28910 0.09509<br>C 1.34726 1.39069 -0.06639<br>C -0.02162 1.21895 0.00509<br>C -0.66455 -0.09357 0.31655<br>H 3.28688 0.42486 0.09993<br>H -0.67382 2.07775 -0.12718<br>H -0.78119 -0.16994 1.41447<br>O -0.19097 -2.31602 -0.48448<br>O 2.46083 -2.03414 0.38912<br>C 1.93817 2.75472 -0.29716<br>H 2.60699 3.03118 0.52470<br>H 2.53032 2.77010 -1.21811<br>H 1.16370 3.52103 -0.37783	C_1	-858.19002 (5.6)	-858.02554 (5.2)	-858.07778 (4.7)	-858.78337 (6.0)	-858.67113 (5.1)

	S -2.32276 -0.30924 -0.41279<br>C -3.23198 0.89716 0.58222<br>H -4.27384 0.83419 0.25885<br>H -3.18768 0.65085 1.64736<br>H -2.89085 1.92452 0.42574						

Table S5.7. Transition structures for the addition of methanethiyl radical to 4-methyl-*o*-benzoquinone in vacuo.

Structure	Schematic drawing	Symmetry	E	H_{RRHO}	G_{RRHO}	$E_{M06\text{-}2X,large}$	$G_{RRHO,M06\text{-}2X,large}$
6-adduct, conf1	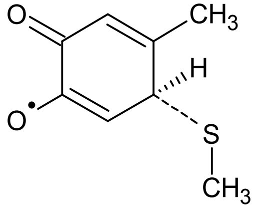<br><br>C 1.51983 -0.65692 0.63860<br>C 1.73282 0.39788 -0.47730<br>C 0.72080 1.44835 -0.61428<br>C -0.36133 1.51513 0.19744<br>C -0.55451 0.50533 1.24652<br>C 0.33861 -0.49504 1.48575<br>H 0.89392 2.18580 -1.39389<br>H -1.39214 0.64996 1.92456<br>H 0.19983 -1.20831 2.29247<br>O 2.31801 -1.56400 0.78451<br>O 2.71198 0.32811 -1.19510<br>C -1.38646 2.59822 0.09108<br>H -1.43133 3.17598 1.02231<br>H -1.16340 3.28362 -0.72885<br>H -2.37953 2.16445 -0.07010<br>S -2.29161 -1.12284 -0.07581<br>C -1.21682 -1.55669 -1.44733<br>H -1.86711 -1.81434 -2.29211<br>H -0.60469 -0.70239 -1.75755<br>H -0.58737 -2.41975 -1.22199<br><br>ν_{imagin} = 93.1*i* cm^{-1}	C_1	-858.16436 (1.5)	-858.00043 (1.6)	-858.05317 (1.2)	-858.76258 (0.8)	-858.65139 (0.5)
6-adduct, conf2	C 1.34571 -1.15949 0.40411<br>C 2.01221 -0.00998 -0.39144<br>C 1.37513 1.30868 -0.33454	C_1	-858.16201 (3.0)	-857.99817 (3.1)	-858.05205 (1.9)	-858.75827 (3.5)	-858.64831 (2.4)

	C 0.22417 1.52525 0.34111<br>C -0.43630 0.41180 1.04149<br>C 0.10698 -0.83930 1.11254<br>H 1.87998 2.10954 -0.86840<br>H -1.30591 0.65704 1.64707<br>H -0.35694 -1.63623 1.68643<br>O 1.85958 -2.26155 0.44343<br>O 3.03058 -0.22161 -1.02045<br>C -0.43317 2.86571 0.41720<br>H -0.50423 3.20146 1.45900<br>H 0.12035 3.61671 -0.14971<br>H -1.45442 2.80839 0.02381<br>S -2.03884 -0.23928 -0.93139<br>C -3.24820 -1.22673 -0.03279<br>H -4.06897 -1.43289 -0.72972<br>H -2.82884 -2.18821 0.27348<br>H -3.66010 -0.69752 0.83034<br><br>ν_{imagin} = 141.2*i* cm^{-1}						
5-adduct, conf1	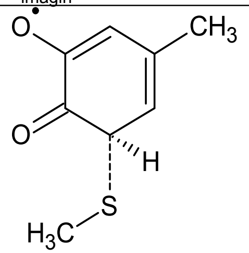<br>C -0.39931 1.20998 -0.80431<br>C 0.53099 1.50942 0.40064<br>C 1.61366 0.56362 0.66235<br>C 1.76631 -0.57144 -0.06941<br>C 0.84111 -0.88460 -1.15000<br>C -0.22853 -0.10004 -1.46425<br>H 2.28920 0.81205 1.47674<br>H 1.01427 -1.80067 -1.70979<br>H -0.86514 -0.30405 -2.31895<br>O -1.19513 2.04132 -1.18511<br>O 0.33416 2.50031 1.07913<br>C 2.87115 -1.54657 0.19177<br>H 3.48835 -1.67745 -0.70492<br>H 3.51523 -1.21906 1.01018<br>H 2.46132 -2.53219 0.44230	C_1	-858.16259 (2.6)	-857.99872 (2.7)	-858.05192 (2.0)	-858.75983 (2.6)	-858.64916 (1.9)

	S -1.94607 -1.54761 0.03049<br>C -1.77091 -0.70846 1.61342<br>H -2.36547 -1.26788 2.34505<br>H -0.73073 -0.73654 1.95590<br>H -2.12895 0.32303 1.58222<br><br>ν_{imagin} = 133.6i cm^{-1}						
5-adduct, conf2	C 0.50710 0.80270 0.83927<br>C -0.25704 1.39079 -0.36514<br>C -1.53078 0.75264 -0.71792<br>C -2.05588 -0.27649 -0.00393<br>C -1.31125 -0.84345 1.10896<br>C -0.08070 -0.39559 1.46811<br>H -2.06139 1.18466 -1.56263<br>H -1.75513 -1.68121 1.64268<br>H 0.46567 -0.82679 2.30279<br>O 1.50705 1.34367 1.27939<br>O 0.17124 2.36878 -0.94856<br>C -3.38569 -0.88299 -0.33295<br>H -3.85270 -0.38965 -1.18775<br>H -3.27898 -1.94925 -0.56484<br>H -4.06662 -0.80877 0.52316<br>S 1.34598 -1.30878 -0.79707<br>C 3.02429 -0.87530 -0.35692<br>H 3.55325 -0.64857 -1.29146<br>H 3.03408 0.02730 0.26765<br>H 3.53944 -1.70077 0.13997<br><br>ν_{imagin} = 92.6i cm^{-1}	C_1	-858.16681 (0.0)	-858.00305 (0.0)	-858.05503 (0.0)	-858.76391 (0.0)	-858.65213 (0.0)

Table S5.8. One-electron oxidized neutral form in water.

Structure	Schematic drawing	Symmetry	G_{PCM}	$H_{PCM,RRHO}$	$G_{PCM,RRHO}$	$G_{SMD,M06\text{-}2X,large}$	$G_{SMD,RRHO,M06\text{-}2X,large}$
6-adduct, conf1	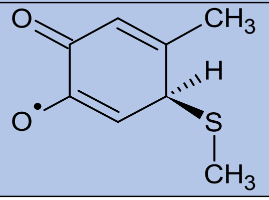	C_1	-858.20642 (4.8)	-858.04117 (4.8)	-858.09212 (5.2)	-858.80587 (2.4)	-858.69157 (2.8)

	[(3*S*)-4-methyl-3-(methylsulfanyl)-6-oxocyclohexa-1,4-dien-1-yl]oxidanyl<br><br>C -1.59120 -0.68341 -0.47779<br>C -1.72972 0.60999 0.32753<br>C -0.60983 1.54248 0.30218<br>C 0.56984 1.28113 -0.30496<br>C 0.82620 -0.05179 -0.93978<br>C -0.35011 -0.91781 -1.12569<br>H -0.77729 2.49939 0.79067<br>H 1.36637 0.07290 -1.88664<br>H -0.24858 -1.80741 -1.74112<br>O -2.55580 -1.46255 -0.57274<br>O -2.76817 0.83934 0.93408<br>C 1.67273 2.28396 -0.36490<br>H 1.86671 2.56061 -1.40866<br>H 1.42336 3.18680 0.19497<br>H 2.60339 1.86306 0.03007<br>S 2.05585 -1.04534 0.04698<br>C 1.23747 -1.21147 1.65129<br>H 1.96448 -1.71855 2.29042<br>H 1.00908 -0.23661 2.08761<br>H 0.33826 -1.82760 1.59299						
6-adduct, conf2	C 1.49480 -1.07773 0.27564<br>C 2.06206 0.24706 -0.23497<br>C 1.21430 1.42921 -0.13486<br>C -0.07085 1.39530 0.28031<br>C -0.73813 0.09103 0.60214<br>C 0.14971 -1.07749 0.72749<br>H 1.68266 2.37399 -0.39971<br>H -1.40023 0.20319 1.46776<br>H -0.25610 -2.00412 1.12426<br>O 2.21923 -2.08766 0.31311<br>O 3.20243 0.29013 -0.67879<br>C -0.89566 2.62907 0.43448<br>H -1.12770 2.78858 1.49481<br>H -0.37338 3.51009 0.05759<br>H -1.85171 2.52810 -0.08981<br>S -1.84953 -0.35852 -0.82636<br>C -3.20999 -1.17739 0.03816<br>H -3.91869 -1.48346 -0.73485<br>H -2.86895 -2.06703 0.57151<br>H -3.70411 -0.48711 0.72518	C_1	-858.20548 (5.3)	-858.04018 (5.4)	-858.09159 (5.5)	-858.80284 (4.3)	-858.68895 (4.5)

5-adduct, conf1	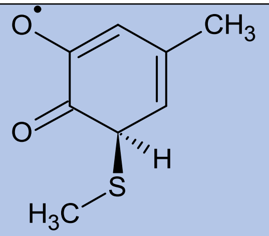<br>[(5*R*)-3-methyl-5-(methylsulfanyl)-6-oxocyclohexa-1,3-dien-1-yl]oxidanyl<br><br>C -0.62087 1.10298 -0.60501<br>C 0.52970 1.56409 0.29740<br>C 1.65936 0.69202 0.40405<br>C 1.73260 -0.57105 -0.21676<br>C 0.64580 -1.06533 -0.91305<br>C -0.65398 -0.35901 -0.99728<br>H 2.50387 1.06053 0.98157<br>H 0.70417 -2.03859 -1.39228<br>H -1.07958 -0.42935 -2.00493<br>O -1.46515 1.89029 -0.97679<br>O 0.45491 2.66354 0.85673<br>C 3.00361 -1.36381 -0.10963<br>H 3.84482 -0.80031 -0.52576<br>H 3.23931 -1.57144 0.93931<br>H 2.92798 -2.31490 -0.64034<br>S -1.92283 -1.25603 0.00954<br>C -1.26614 -1.13731 1.69271<br>H -1.90427 -1.78555 2.29797<br>H -0.24095 -1.50914 1.75056<br>H -1.32850 -0.12100 2.08707	C_1	-858.21164 (1.5)	-858.04641 (1.5)	-858.09814 (1.4)	-858.80896 (0.5)	-858.69546 (0.4)
5-adduct, conf2	C 0.48495 0.91611 0.60812<br>C -0.62795 1.50696 -0.26556<br>C -1.81018 0.70833 -0.41973<br>C -1.94055 -0.59350 0.10299<br>C -0.85845 -1.20952 0.70470<br>C 0.48215 -0.57833 0.76694<br>H -2.64541 1.17121 -0.93975<br>H -0.95069 -2.21800 1.09695<br>H 1.04500 -0.88042 1.65501<br>O 1.33677 1.62836 1.10324<br>O -0.50479 2.64581 -0.72321<br>C -3.26062 -1.29947 -0.01390	C_1	-858.21399 (0.0)	-858.04882 (0.0)	-858.10034 (0.0)	-858.80975 (0.0)	-858.69610 (0.0)

	H -4.05194 -0.72056 0.47271<br>H -3.54129 -1.41319 -1.06614<br>H -3.22624 -2.29086 0.44203<br>S 1.42804 -1.15277 -0.72427<br>C 3.11258 -0.70503 -0.24798<br>H 3.74693 -0.97219 -1.09641<br>H 3.19579 0.36866 -0.06204<br>H 3.43188 -1.26707 0.63237						
5-adduct, conf3	C 0.18266 -1.29085 0.43562<br>C 1.56886 -1.09430 -0.18590<br>C 2.05766 0.24653 -0.27557<br>C 1.31704 1.37569 0.11688<br>C 0.01619 1.23261 0.56672<br>C -0.69758 -0.06620 0.59980<br>H 3.07016 0.36823 -0.65318<br>H -0.54509 2.11120 0.87280<br>H -1.27134 -0.19601 1.52401<br>O -0.18710 -2.39285 0.78010<br>O 2.21214 -2.08675 -0.54541<br>C 1.95304 2.73342 0.02856<br>H 2.89368 2.75815 0.58753<br>H 2.18734 2.97757 -1.01314<br>H 1.29481 3.51036 0.42243<br>S -1.95241 -0.08910 -0.77037<br>C -3.34294 0.71531 0.06256<br>H -4.15317 0.74489 -0.66985<br>H -3.66719 0.13605 0.92983<br>H -3.10062 1.73863 0.35589	C_1	-858.20931 (2.9)	-858.04413 (2.9)	-858.09718 (2.0)	-858.80474 (3.1)	-858.69261 (2.2)
5-adduct, conf4	C 0.23477 -1.28876 0.09051<br>C 1.74961 -1.04179 0.09194<br>C 2.19494 0.31128 -0.02559<br>C 1.32406 1.41535 -0.02630<br>C -0.03272 1.22869 0.15615<br>C -0.66739 -0.09985 0.37922<br>H 3.26644 0.46314 -0.13087<br>H -0.68537 2.09620 0.18638<br>H -0.90953 -0.19334 1.45311<br>O -0.19249 -2.40317 -0.11633<br>O 2.50989 -2.01385 0.13798<br>C 1.89248 2.79479 -0.20084<br>H 2.61780 3.01418 0.58924<br>H 2.41944 2.87425 -1.15718<br>H 1.11139 3.55710 -0.17298	C_1	-858.20825 (3.6)	-858.04342 (3.4)	-858.09618 (2.6)	-858.80393 (3.7)	-858.69186 (2.7)

	S -2.25629 -0.28235 -0.52037<br>C -3.30169 0.83067 0.44959<br>H -4.30551 0.74318 0.02764<br>H -3.33263 0.52367 1.49789<br>H -2.98511 1.87314 0.37137						

Table S5.9. Transition structures for the addition of methanethiyl radical to 4-methyl-*o*-benzoquinone in water.

Structure	Schematic drawing	Symmetry	G_{PCM}	$H_{PCM,RRHO}$	$G_{PCM,RRHO}$	$G_{SMD,M06-2X,large}$	$G_{SMD,RRHO,M06-2X,large}$
6-addition, conf1	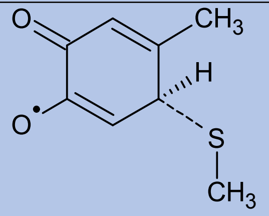<br>C -1.51883 -0.68957 -0.63756<br>C -1.73851 0.33291 0.50919<br>C -0.76894 1.40965 0.65156<br>C 0.28768 1.53070 -0.19015<br>C 0.49014 0.54878 -1.26630<br>C -0.36405 -0.48388 -1.50052<br>H -0.94166 2.12566 1.45028<br>H 1.31025 0.73278 -1.95510<br>H -0.21682 -1.17291 -2.32625<br>O -2.31161 -1.60734 -0.78437<br>O -2.70860 0.20155 1.24049<br>C 1.26878 2.65060 -0.09454<br>H 1.25281 3.24275 -1.01738<br>H 1.05003 3.30839 0.74807<br>H 2.28510 2.25618 0.01256<br>S 2.34446 -1.08677 0.01844<br>C 1.38661 -1.49009 1.48172<br>H 2.10730 -1.70324 2.28085<br>H 0.78337 -0.63607 1.80716<br>H 0.76262 -2.37340 1.33540<br><br>ν_{imagin} = 79.6*i* cm^{-1}	C_1	-858.17906 (1.8)	-858.01526 (1.9)	-858.06842 (1.3)	-858.77883 (1.2)	-858.66819 (1.1)
6- addition, conf2	C 1.34072 -1.15096 0.43070<br>C 2.00936 -0.02467 -0.40010	C_1	-858.17867 (2.1)	-858.01491 (2.1)	-858.06972 (0.4)	-858.77612 (2.9)	-858.66717 (1.8)

	C 1.39490 1.29374 -0.37278<br>C 0.25680 1.53344 0.32234<br>C -0.39916 0.44394 1.06552<br>C 0.12065 -0.81225 1.15006<br>H 1.89268 2.08152 -0.93150<br>H -1.27105 0.70649 1.65937<br>H -0.35039 -1.58848 1.74519<br>O 1.85517 -2.25835 0.46728<br>O 3.02256 -0.28064 -1.03421<br>C -0.37872 2.88103 0.39095<br>H -0.40679 3.23075 1.42993<br>H 0.16115 3.61317 -0.21158<br>H -1.41710 2.82817 0.04550<br>S -2.04322 -0.31671 -0.95190<br>C -3.34966 -1.10699 0.00034<br>H -4.16263 -1.32739 -0.70173<br>H -3.01004 -2.05530 0.42324<br>H -3.73557 -0.45348 0.78514<br><br>ν_{imagin} = 109.3*i* cm^{-1}						
5-addition, conf1	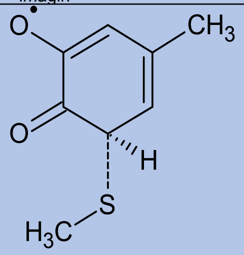<br>C -0.94636 -1.09688 0.62692<br>C -0.23162 -1.42365 -0.70588<br>C 1.14636 -0.97758 -0.84977<br>C 1.77794 -0.27239 0.12653<br>C 1.05673 0.12261 1.33451<br>C -0.24266 -0.19665 1.55261<br>H 1.66095 -1.25985 -1.76429<br>H 1.59478 0.71614 2.06941<br>H -0.75845 0.08687 2.46534<br>O -2.01040 -1.62713 0.89657<br>O -0.81966 -2.07820 -1.55579<br>C 3.21116 0.13562 0.01789<br>H 3.65083 -0.17870 -0.93004<br>H 3.31168 1.22280 0.11299	C_1	-858.17973<br>(1.4)	-858.01612<br>(1.4)	-858.06995<br>(0.3)	-858.77980<br>(0.6)	-858.67002<br>(0.0)

	H 3.78999 -0.30518 0.83810<br>S -1.53869 1.66438 -0.21738<br>C -0.15933 2.70231 -0.69613<br>H -0.42250 3.14700 -1.66419<br>H 0.00665 3.50849 0.02077<br>H 0.75232 2.11457 -0.83618<br><br>ν_{imagin} = 94.2i cm^{-1}						
5- addition, conf2	C 0.47317 0.83263 0.84635<br>C -0.22375 1.32725 -0.44088<br>C -1.47918 0.68254 -0.80866<br>C -2.05926 -0.26490 -0.02616<br>C -1.38813 -0.72955 1.18310<br>C -0.17997 -0.26290 1.57521<br>H -1.96107 1.03515 -1.71682<br>H -1.88268 -1.50050 1.76892<br>H 0.31170 -0.62437 2.47389<br>O 1.47411 1.40055 1.26443<br>O 0.25570 2.26350 -1.06778<br>C -3.37829 -0.88180 -0.36315<br>H -3.79078 -0.47587 -1.28851<br>H -3.27659 -1.96810 -0.46898<br>H -4.09612 -0.71049 0.44743<br>S 1.36492 -1.31908 -0.76426<br>C 3.04119 -0.93530 -0.28613<br>H 3.61860 -0.79047 -1.20803<br>H 3.07533 -0.01331 0.30549<br>H 3.48967 -1.76697 0.26345<br><br>ν_{imagin} = 42.0i cm^{-1}	C_1	-858.18195 (0.0)	-858.01828 (0.0)	-858.07042 (0.0)	-858.78069 (0.0)	-858.66916 (0.5)

Part 6: 4-Methylcatechol – methanethiol rearomatized adducts

Table S6.1. Reduced neutral form in vacuo.

Structure	Schematic drawing	Symmetry	*E*	H_{RRHO}	G_{RRHO}	$E_{M06-2X,large}$	$G_{RRHO,M06-2X,large}$
6-adduct, conf1	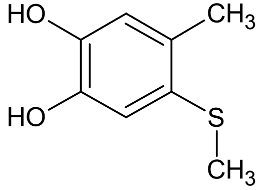<br>4-methyl-5-(methylsulfanyl)benzene-1,2-diol<br><br>C -1.64414 -0.84373 -0.02637<br>C -1.99798 0.50449 0.09856<br>C -1.00801 1.47571 0.03838<br>C 0.33937 1.14645 -0.14473<br>C 0.68378 -0.21296 -0.25294<br>C -0.31562 -1.19480 -0.19628<br>H -1.30988 2.51568 0.12638<br>H -0.03460 -2.24141 -0.28983<br>O -2.68715 -1.73013 0.03679<br>O -3.29224 0.86472 0.26863<br>C 1.36292 2.24436 -0.20936<br>H 0.90053 3.18860 -0.50922<br>H 1.83566 2.40942 0.76673<br>H 2.15837 2.00019 -0.91886<br>S 2.35517 -0.75706 -0.53909<br>C 3.08648 -0.56358 1.11184<br>H 4.11444 -0.92923 1.04310<br>H 3.10688 0.48345 1.42283<br>H 2.54483 -1.15938 1.85006<br>H -3.83191 0.06304 0.27117<br>H -2.37272 -2.62971 -0.09486	C_1	-858.86248 (4.7)	-858.68339 (4.5)	-858.73436 (4.2)	-859.46447 (3.9)	-859.33634 (3.5)
6-adduct, conf2	C -1.62753 -0.88850 -0.03908<br>C -1.99033 0.45533 0.09659<br>C -1.02452 1.45034 0.04476<br>C 0.32974 1.14901 -0.14097<br>C 0.68945 -0.20529 -0.26279<br>C -0.28897 -1.20568 -0.21676<br>H -1.32953 2.49139 0.14286	C_1	-858.86170 (5.2)	-858.68265 (5.0)	-858.73391 (4.5)	-859.46376 (4.4)	-859.33597 (3.7)

	H -0.00805 -2.24841 -0.32353<br>O -2.56332 -1.86991 0.00154<br>O -3.32803 0.68357 0.26921<br>C 1.33625 2.26302 -0.19511<br>H 2.08701 2.07826 -0.96826<br>H 0.84823 3.21985 -0.40077<br>H 1.87355 2.36822 0.75535<br>S 2.37623 -0.70817 -0.54649<br>C 3.06084 -0.60001 1.13197<br>H 4.09871 -0.93758 1.06913<br>H 3.04795 0.42561 1.50836<br>H 2.51485 -1.25331 1.81638<br>H -3.50280 1.62695 0.34019<br>H -3.42850 -1.45884 0.12649						
6-adduct, conf3	C -0.74846 -1.54288 0.00000<br>C 0.57553 -1.98732 0.00000<br>C 1.59407 -1.04673 0.00000<br>C 1.33760 0.32731 0.00000<br>C 0.00000 0.76052 0.00000<br>C -1.03391 -0.18547 0.00000<br>H 2.62040 -1.40454 0.00000<br>H -2.07451 0.12562 0.00000<br>O -1.69860 -2.53105 0.00000<br>O 0.86678 -3.31383 0.00000<br>C 2.50627 1.27701 0.00000<br>H 2.19900 2.32475 0.00000<br>H 3.13460 1.11959 0.88337<br>H 3.13460 1.11959 -0.88337<br>S -0.31641 2.51025 0.00000<br>C -2.11495 2.60881 0.00000<br>H -2.34682 3.67637 0.00000<br>H -2.55010 2.16057 -0.89781<br>H -2.55010 2.16057 0.89781<br>H 0.03497 -3.80467 0.00000<br>H -2.58176 -2.15033 0.00000	C_s	-858.85991 (6.3)	saddle point			
6-adduct, conf4	C 0.76806 -1.54242 0.00000<br>C -0.55101 -1.99804 0.00000<br>C -1.58209 -1.06790 0.00000<br>C -1.33589 0.30589 0.00000<br>C 0.00000 0.75037 0.00000<br>C 1.04376 -0.18125 0.00000<br>H -2.60404 -1.43650 0.00000<br>H 2.08114 0.14122 0.00000	C_s	-858.86225 (4.8)	-858.68320 (4.6)	-858.73367 (4.7)	-859.46499 (3.6)	-859.33641 (3.4)

	O 1.72710 -2.52251 0.00000<br>O -0.83048 -3.32733 0.00000<br>C -2.47785 1.28126 0.00000<br>H -3.43697 0.75768 0.00000<br>H -2.44806 1.93376 0.88117<br>H -2.44806 1.93376 -0.88117<br>S 0.26495 2.50389 0.00000<br>C 2.06044 2.65204 0.00000<br>H 2.26677 3.72475 0.00000<br>H 2.50597 2.21343 0.89751<br>H 2.50597 2.21343 -0.89751<br>H 0.00581 -3.81047 0.00000<br>H 2.60693 -2.13430 0.00000						
5-adduct, conf1	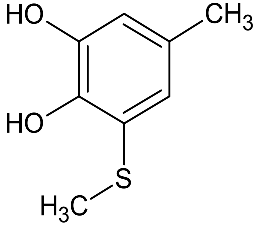<br>5-methyl-3-(methylsulfanyl)benzene-1,2-diol<br><br>C -0.07542 1.01281 -0.12681<br>C 1.29287 1.22710 0.09206<br>C 2.15745 0.14485 0.15075<br>C 1.68931 -1.16643 -0.01317<br>C 0.32903 -1.36904 -0.23459<br>C -0.55802 -0.28615 -0.28077<br>H 3.21303 0.34066 0.31936<br>H -0.06001 -2.37278 -0.38123<br>O -0.86161 2.12032 -0.17018<br>O 1.76640 2.48825 0.24475<br>C 2.65044 -2.32249 0.04203<br>H 3.42177 -2.23556 -0.73126<br>H 3.16343 -2.36768 1.00918<br>H 2.13392 -3.27444 -0.10720<br>S -2.29901 -0.49501 -0.57730<br>C -2.89905 -0.80109 1.11326<br>H -3.98421 -0.91380 1.04823<br>H -2.46589 -1.72168 1.50938<br>H -2.66244 0.03491 1.77472	C_1	-858.86994 (0.0)	-858.69054 (0.0)	-858.74112 (0.0)	-859.47073 (0.0)	-859.34191 (0.0)

	H 1.02496 3.10130 0.15181<br>H -1.75835 1.82347 -0.40935						
5-adduct, conf2	C -0.07653 1.01354 -0.12758<br>C 1.28794 1.23195 0.09125<br>C 2.15773 0.15021 0.15142<br>C 1.69603 -1.16023 -0.01098<br>C 0.33354 -1.36805 -0.23322<br>C -0.55468 -0.29000 -0.28066<br>H 3.21206 0.35058 0.32031<br>H -0.05253 -2.37347 -0.37855<br>O -0.86940 2.11679 -0.17314<br>O 1.76104 2.49359 0.24304<br>C 2.64973 -2.32361 0.04368<br>H 2.38360 -3.02013 0.84630<br>H 2.64197 -2.88898 -0.89467<br>H 3.67475 -1.98759 0.22113<br>S -2.29552 -0.50437 -0.57760<br>C -2.89636 -0.79445 1.11541<br>H -3.98171 -0.90582 1.05113<br>H -2.46483 -1.71222 1.51977<br>H -2.65862 0.04701 1.76952<br>H 1.01930 3.10608 0.14905<br>H -1.76307 1.81523 -0.41745	C_1	-858.86959 (0.2)	saddle point			
5-adduct, conf3	C -0.20927 0.90135 -0.14580<br>C 1.12587 1.29283 0.02082<br>C 2.14217 0.35589 0.11642<br>C 1.85052 -1.01299 0.05651<br>C 0.51968 -1.39779 -0.08401<br>C -0.51794 -0.45989 -0.18765<br>H 3.17110 0.69038 0.24245<br>H 0.26084 -2.45242 -0.11228<br>O -1.17793 1.84166 -0.26774<br>O 1.31896 2.64933 0.06185<br>C 2.95316 -2.03111 0.15615<br>H 3.64730 -1.95188 -0.68846<br>H 3.53850 -1.90014 1.07310<br>H 2.55058 -3.04734 0.15990<br>S -2.17089 -1.03183 -0.47132<br>C -3.07239 -0.29038 0.91786<br>H -4.10798 -0.62573 0.81996<br>H -2.67277 -0.64153 1.87200<br>H -3.03711 0.79867 0.86682<br>H 2.25811 2.85409 0.09908	C_1	-858.86243 (4.7)	-858.68310 (4.7)	-858.73438 (4.2)	-859.46404 (4.2)	-859.33599 (3.7)

	H -0.75339 2.70981 -0.24617						
5-adduct, conf4	C -0.30086 -1.11790 0.00138<br>C -1.66221 -0.80963 -0.00024<br>C -2.09995 0.50768 -0.00285<br>C -1.16962 1.55163 -0.00324<br>C 0.19050 1.24118 -0.00271<br>C 0.64108 -0.08545 -0.00011<br>H -3.16727 0.72295 -0.00453<br>H 0.90738 2.05487 -0.00453<br>O 0.12049 -2.40766 0.00269<br>O -2.49273 -1.89953 -0.00047<br>C -1.63486 2.98253 0.00386<br>H -2.17141 3.22354 0.92878<br>H -0.79115 3.67299 -0.07879<br>H -2.31505 3.18644 -0.83048<br>S 2.33519 -0.58326 -0.00017<br>C 3.20108 0.99846 -0.00022<br>H 4.26419 0.74647 0.00097<br>H 2.98435 1.58467 -0.89742<br>H 2.98251 1.58571 0.89585<br>H -3.41246 -1.61824 -0.00191<br>H -0.65718 -2.98058 0.00185	C_1	-858.86219 (4.9)	-858.68302 (4.7)	-858.73486 (3.9)	-859.46436 (4.0)	-859.33703 (3.1)
5-adduct, conf5	C 1.13806 0.18221 0.00000<br>C 1.01089 1.57505 0.00000<br>C -0.24710 2.16208 0.00000<br>C -1.39646 1.36773 0.00000<br>C -1.26362 -0.02142 0.00000<br>C 0.00000 -0.62525 0.00000<br>H -0.31239 3.24623 0.00000<br>H -2.15838 -0.63458 0.00000<br>O 2.42672 -0.28115 0.00000<br>O 2.12062 2.35328 0.00000<br>C -2.75786 2.00865 0.00000<br>H -2.89804 2.64306 0.88216<br>H -2.89804 2.64306 -0.88216<br>H -3.55233 1.25725 0.00000<br>S 0.28371 -2.37415 0.00000<br>C -1.37605 -3.07339 0.00000<br>H -1.23832 -4.15663 0.00000<br>H -1.92823 -2.78629 0.89793<br>H -1.92823 -2.78629 -0.89793<br>H 2.89456 1.77428 0.00000<br>H 2.45411 -1.24479 0.00000	C_s	-858.86364 (4.0)	saddle point			

5-adduct, conf6	C -1.13914 0.18757 0.00000<br>C -1.00907 1.57712 0.00000<br>C 0.25310 2.16218 0.00000<br>C 1.39861 1.36758 0.00000<br>C 1.26264 -0.02433 0.00000<br>C 0.00000 -0.62336 0.00000<br>H 0.32026 3.24588 0.00000<br>H 2.15637 -0.63977 0.00000<br>O -2.42815 -0.27559 0.00000<br>O -2.11494 2.36108 0.00000<br>C 2.76830 1.99169 0.00000<br>H 3.34377 1.69115 0.88270<br>H 3.34377 1.69115 -0.88270<br>H 2.70518 3.08295 0.00000<br>S -0.29054 -2.37111 0.00000<br>C 1.36667 -3.07592 0.00000<br>H 1.22568 -4.15873 0.00000<br>H 1.91972 -2.79033 -0.89790<br>H 1.91972 -2.79033 0.89790<br>H -2.89168 1.78587 0.00000<br>H -2.45603 -1.23919 0.00000	C_s	-858.86344 (4.1)	saddle point			
5-adduct, conf7	C 1.14798 0.15012 0.00000<br>C 1.02254 1.54027 0.00000<br>C -0.22532 2.14836 0.00000<br>C -1.38327 1.36441 0.00000<br>C -1.25554 -0.02467 0.00000<br>C 0.00000 -0.64681 0.00000<br>H -0.29766 3.23477 0.00000<br>H -2.15672 -0.62803 0.00000<br>O 2.37095 -0.43772 0.00000<br>O 2.21237 2.21949 0.00000<br>C -2.73985 2.01524 0.00000<br>H -2.87930 2.64946 0.88274<br>H -2.87930 2.64946 -0.88274<br>H -3.53744 1.26737 0.00000<br>S 0.26916 -2.39209 0.00000<br>C -1.41352 -3.04036 0.00000<br>H -1.30560 -4.12753 0.00000<br>H -1.96560 -2.74598 0.89667<br>H -1.96560 -2.74598 -0.89667<br>H 2.05521 3.16835 0.00000<br>H 3.04075 0.25819 0.00000	C_s	-858.86220 (4.9)	-858.68298 (4.7)	-858.73423 (4.3)	-859.46432 (4.0)	-859.33635 (3.5)

5-adduct, conf8	C -1.14847 0.15615 0.00000<br>C -1.02013 1.54308 0.00000<br>C 0.23228 2.14830 0.00000<br>C 1.38589 1.36347 0.00000<br>C 1.25495 -0.02830 0.00000<br>C 0.00000 -0.64509 0.00000<br>H 0.30754 3.23421 0.00000<br>H 2.15497 -0.63415 0.00000<br>O -2.37166 -0.43148 0.00000<br>O -2.20672 2.22856 0.00000<br>C 2.75067 1.99767 0.00000<br>H 3.32862 1.70132 0.88246<br>H 3.32862 1.70132 -0.88246<br>H 2.68199 3.08903 0.00000<br>S -0.27737 -2.38906 0.00000<br>C 1.40259 -3.04388 0.00000<br>H 1.29086 -4.13065 0.00000<br>H 1.95572 -2.75125 -0.89663<br>H 1.95572 -2.75125 0.89663<br>H -2.04459 3.17654 0.00000<br>H -3.04111 0.26476 0.00000	C_s	-858.86207 (4.9)	saddle point			

Table S6.2. Reduced neutral form in water.

Structure	Schematic drawing	Symmetry	G_{PCM}	$H_{PCM,RRHO}$	$G_{PCM,RRHO}$	$G_{SMD,M06\text{-}2X,large}$	$G_{SMD,RRHO,M06\text{-}2X,large}$
6-adduct, conf1	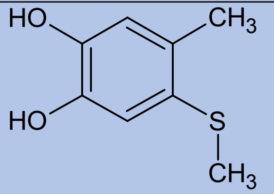<br>4-methyl-5-(methylsulfanyl)benzene-1,2-diol<br><br>C -1.61522 -0.86127 -0.06066<br>C -1.99858 0.47501 0.10810<br>C -1.03590 1.47632 0.07797<br>C 0.31986 1.18856 -0.11700<br>C 0.69344 -0.15803 -0.27719<br>C -0.27705 -1.16924 -0.25484<br>H -1.36125 2.50511 0.20733	C_1	-858.87255 (2.9)	-858.69384 (2.7)	-858.74526 (2.4)	-859.47705 (1.7)	-859.34975 (1.4)

	H 0.02436 -2.20498 -0.38887<br>O -2.63254 -1.77074 -0.02088<br>O -3.30316 0.79795 0.29725<br>C 1.31930 2.31105 -0.14794<br>H 0.81662 3.27673 -0.05315<br>H 2.04523 2.22989 0.66827<br>H 1.89082 2.30991 -1.08125<br>S 2.38956 -0.62932 -0.56066<br>C 3.01027 -0.78219 1.14078<br>H 4.05776 -1.08505 1.06714<br>H 2.95330 0.17117 1.67065<br>H 2.45396 -1.54674 1.68721<br>H -3.82615 -0.01635 0.29025<br>H -2.29872 -2.66956 -0.12328						
6-adduct, conf2	C -1.63751 -0.88427 -0.02758<br>C -1.99649 0.46393 0.09213<br>C -1.02147 1.45093 0.03279<br>C 0.33205 1.13790 -0.14736<br>C 0.68479 -0.21998 -0.25427<br>C -0.30138 -1.21447 -0.19653<br>H -1.32039 2.49339 0.12108<br>H -0.02657 -2.26116 -0.28343<br>O -2.58175 -1.86217 0.02189<br>O -3.32812 0.69856 0.25733<br>C 1.34130 2.24937 -0.20454<br>H 0.86143 3.19611 -0.46565<br>H 1.83730 2.39118 0.76311<br>H 2.12293 2.03750 -0.93939<br>S 2.36564 -0.74326 -0.53734<br>C 3.11004 -0.53817 1.10691<br>H 4.14187 -0.88935 1.02709<br>H 3.11679 0.50913 1.41525<br>H 2.58218 -1.14356 1.84670<br>H -3.50786 1.64374 0.32412<br>H -3.44696 -1.44736 0.14554	C_1	-858.87241 (2.9)	-858.69367 (2.8)	-858.74500 (2.6)	-859.47684 (1.8)	-859.34943 (1.6)
6-adduct, conf3	C -1.60414 -0.89725 -0.06201<br>C -1.99656 0.43636 0.10415<br>C -1.04772 1.45111 0.07502<br>C 0.31198 1.17796 -0.11912<br>C 0.69745 -0.16556 -0.27615<br>C -0.26099 -1.18669 -0.25208<br>H -1.37314 2.48157 0.20222<br>H 0.04009 -2.22176 -0.38210	C_1	-858.87242 (2.9)	-858.69374 (2.8)	-858.74501 (2.6)	-859.47700 (1.7)	-859.34958 (1.5)

	O -2.52280 -1.90030 -0.04063<br>O -3.33270 0.63009 0.28396<br>C 1.29838 2.31174 -0.15329<br>H 2.02589 2.24114 0.66244<br>H 1.86878 2.31499 -1.08727<br>H 0.78521 3.27219 -0.06056<br>S 2.39966 -0.61778 -0.55902<br>C 3.02351 -0.75129 1.14279<br>H 4.07434 -1.04257 1.07022<br>H 2.95619 0.20502 1.66611<br>H 2.47643 -1.51832 1.69498<br>H -3.53790 1.56769 0.37765<br>H -3.39800 -1.51206 0.09817						
6-adduct, conf4	C -0.74429 -1.54693 0.00000<br>C 0.58458 -1.98261 0.00000<br>C 1.60210 -1.04066 0.00000<br>C 1.34097 0.33426 0.00000<br>C 0.00000 0.75869 0.00000<br>C -1.03299 -0.18930 0.00000<br>H 2.63137 -1.39115 0.00000<br>H -2.07445 0.11558 0.00000<br>O -1.68540 -2.53643 0.00000<br>O 0.88087 -3.31183 0.00000<br>C 2.50713 1.28759 0.00000<br>H 2.19641 2.33436 0.00000<br>H 3.13746 1.13181 0.88211<br>H 3.13746 1.13181 -0.88211<br>S -0.33237 2.50818 0.00000<br>C -2.13340 2.60204 0.00000<br>H -2.36607 3.66913 0.00000<br>H -2.56381 2.15077 -0.89730<br>H -2.56381 2.15077 0.89730<br>H 0.05118 -3.80908 0.00000<br>H -2.57614 -2.16725 0.00000	C_s	-858.87145 (3.5)	saddle point			
6-adduct, conf5	C 0.76301 -1.54638 0.00000<br>C -0.56095 -1.99314 0.00000<br>C -1.59067 -1.06120 0.00000<br>C -1.33943 0.31354 0.00000<br>C 0.00000 0.74907 0.00000<br>C 1.04236 -0.18508 0.00000<br>H -2.61579 -1.42243 0.00000<br>H 2.08071 0.13104 0.00000<br>O 1.71298 -2.52795 0.00000	C_s	-858.87374 (2.1)	-858.69497 (2.0)	-858.74533 (2.4)	-859.47975 (0.0)	-859.35134 (0.4)

	O -0.84574 -3.32524 0.00000<br>C -2.47984 1.29145 0.00000<br>H -3.43995 0.77002 0.00000<br>H -2.45031 1.94438 0.88090<br>H -2.45031 1.94438 -0.88090<br>S 0.28278 2.50191 0.00000<br>C 2.08092 2.64392 0.00000<br>H 2.28878 3.71606 0.00000<br>H 2.52158 2.20192 0.89702<br>H 2.52158 2.20192 -0.89702<br>H -0.01151 -3.81475 0.00000<br>H 2.60034 -2.15084 0.00000						
5-adduct, conf1	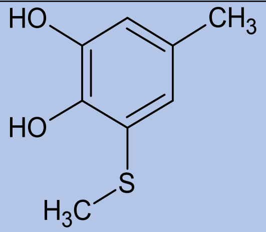<br>5-methyl-3-(methylsulfanyl)benzene-1,2-diol<br><br>C -0.04762 1.03155 -0.11602<br>C 1.32890 1.20112 0.09468<br>C 2.16373 0.09480 0.14275<br>C 1.65645 -1.20280 -0.02168<br>C 0.28869 -1.36196 -0.23732<br>C -0.56764 -0.25331 -0.27665<br>H 3.22633 0.25518 0.30672<br>H -0.12994 -2.35461 -0.37891<br>O -0.79806 2.16237 -0.14575<br>O 1.84433 2.44911 0.25363<br>C 2.58202 -2.38788 0.02808<br>H 3.35869 -2.31758 -0.74128<br>H 3.09140 -2.45288 0.99583<br>H 2.03657 -3.32212 -0.12828<br>S -2.31414 -0.41311 -0.57419<br>C -2.92602 -0.83852 1.08546<br>H -4.00999 -0.94373 0.99925<br>H -2.49746 -1.78615 1.41638<br>H -2.69583 -0.04809 1.80187<br>H 1.12424 3.09144 0.18306	C_1	-858.87710 (0.0)	-858.69820 (0.0)	-858.74909 (0.0)	-859.47830 (0.9)	-859.35029 (1.1)

	H -1.71885 1.89841 -0.32654						
5-adduct, conf2	C -0.04625 1.03338 -0.11620<br>C 1.32704 1.20411 0.09413<br>C 2.16449 0.09618 0.14288<br>C 1.66040 -1.19958 -0.02096<br>C 0.29010 -1.36051 -0.23772<br>C -0.56511 -0.25471 -0.27686<br>H 3.22647 0.25860 0.30710<br>H -0.12737 -2.35388 -0.37972<br>O -0.80110 2.16222 -0.14668<br>O 1.84503 2.45151 0.25182<br>C 2.57526 -2.39392 0.02966<br>H 2.30725 -3.06429 0.85350<br>H 2.51667 -2.97833 -0.89478<br>H 3.61586 -2.09020 0.16988<br>S -2.31208 -0.41587 -0.57444<br>C -2.92348 -0.83420 1.08679<br>H -4.00728 -0.94158 1.00099<br>H -2.49365 -1.77973 1.42213<br>H -2.69499 -0.04041 1.80004<br>H 1.12456 3.09427 0.19041<br>H -1.72035 1.89507 -0.32995	C_1	-858.87678 (0.2)	saddle point			
5-adduct, conf3	C -0.19758 0.91106 -0.15539<br>C 1.14233 1.28833 0.02412<br>C 2.14418 0.33437 0.12106<br>C 1.83480 -1.03125 0.05099<br>C 0.50040 -1.40113 -0.10559<br>C -0.52188 -0.44562 -0.21005<br>H 3.17578 0.65452 0.25364<br>H 0.23195 -2.45316 -0.14580<br>O -1.15772 1.86252 -0.27528<br>O 1.34739 2.63658 0.07594<br>C 2.92463 -2.06260 0.15765<br>H 3.65278 -1.95650 -0.65414<br>H 3.47495 -1.96413 1.09972<br>H 2.51487 -3.07482 0.11105<br>S -2.19114 -0.97942 -0.49370<br>C -3.05049 -0.33445 0.97035<br>H -4.09720 -0.63042 0.86770<br>H -2.64085 -0.77428 1.88197<br>H -2.98315 0.75368 1.00839<br>H 2.28464 2.84087 0.17380<br>H -0.73125 2.72994 -0.22116	C_1	-858.87327 (2.4)	-858.69432 (2.4)	-858.74540 (2.3)	-859.47686 (1.8)	-859.34899 (1.9)

5-adduct, conf4	C -0.30734 -1.11555 0.00101<br>C -1.67089 -0.80698 -0.00015<br>C -2.10207 0.51358 -0.00206<br>C -1.16676 1.55504 -0.00234<br>C 0.19429 1.24341 -0.00194<br>C 0.63689 -0.08647 -0.00007<br>H -3.16795 0.73167 -0.00340<br>H 0.91454 2.05420 -0.00320<br>O 0.11157 -2.41119 0.00210<br>O -2.49734 -1.89362 -0.00052<br>C -1.62875 2.98722 0.00264<br>H -2.29325 3.19363 -0.84327<br>H -2.18697 3.22088 0.91613<br>H -0.78213 3.67595 -0.05798<br>S 2.33505 -0.58547 -0.00015<br>C 3.21902 0.98760 -0.00015<br>H 4.27867 0.72322 0.00031<br>H 3.00381 1.57291 -0.89709<br>H 3.00307 1.57329 0.89636<br>H -3.42248 -1.62256 -0.00027<br>H -0.66807 -2.98429 0.00063	C_1	-858.87443 (1.7)	-858.69553 (1.7)	-858.74716 (1.2)	-859.47929 (0.3)	-859.35202 (0.0)
5-adduct, conf5	C -1.14708 -0.15355 0.00000<br>C -1.02504 -1.54620 0.00000<br>C 0.22497 -2.15186 0.00000<br>C 1.38340 -1.36617 0.00000<br>C 1.25813 0.02451 0.00000<br>C 0.00000 0.64293 0.00000<br>H 0.29626 -3.23753 0.00000<br>H 2.15814 0.62962 0.00000<br>O -2.37473 0.43544 0.00000<br>O -2.21259 -2.21948 0.00000<br>C 2.74050 -2.01716 0.00000<br>H 2.87561 -2.65371 0.88147<br>H 2.87561 -2.65371 -0.88147<br>H 3.53836 -1.27034 0.00000<br>S -0.26514 2.39309 0.00000<br>C 1.41310 3.05643 0.00000<br>H 1.29508 4.14217 0.00000<br>H 1.96365 2.76303 0.89675<br>H 1.96365 2.76303 -0.89675<br>H -2.06678 -3.17248 0.00000<br>H -3.04660 -0.26081 0.00000	C_s	-858.87445 (1.7)	-858.69548 (1.7)	-858.74686 (1.4)	-859.47919 (0.4)	-859.35159 (0.3)

5-adduct, conf6	C 1.14560 0.17328 0.00000<br>C 1.02334 1.56722 0.00000<br>C -0.23029 2.16468 0.00000<br>C -1.38629 1.37736 0.00000<br>C -1.26004 -0.01310 0.00000<br>C 0.00000 -0.62716 0.00000<br>H -0.29269 3.24945 0.00000<br>H -2.15764 -0.62189 0.00000<br>O 2.43144 -0.29272 0.00000<br>O 2.14037 2.34233 0.00000<br>C -2.74416 2.02611 0.00000<br>H -2.87927 2.66264 0.88127<br>H -2.87927 2.66264 -0.88127<br>H -3.54171 1.27860 0.00000<br>S 0.25958 -2.37992 0.00000<br>C -1.40946 -3.06289 0.00000<br>H -1.27781 -4.14663 0.00000<br>H -1.95757 -2.77136 0.89818<br>H -1.95757 -2.77136 -0.89818<br>H 2.91609 1.76375 0.00000<br>H 2.46752 -1.25715 0.00000	C_s	-858.87246 (2.9)	saddle point			
5-adduct, conf7	C -1.14665 0.17898 0.00000<br>C -1.02144 1.56959 0.00000<br>C 0.23652 2.16497 0.00000<br>C 1.38851 1.37705 0.00000<br>C 1.25903 -0.01607 0.00000<br>C 0.00000 -0.62514 0.00000<br>H 0.30073 3.24941 0.00000<br>H 2.15592 -0.62653 0.00000<br>O -2.43280 -0.28703 0.00000<br>O -2.13460 2.35066 0.00000<br>C 2.75472 2.00881 0.00000<br>H 3.33153 1.70778 0.88130<br>H 3.33153 1.70778 -0.88130<br>H 2.68592 3.09961 0.00000<br>S -0.26679 -2.37676 0.00000<br>C 1.39970 -3.06548 0.00000<br>H 1.26473 -4.14881 0.00000<br>H 1.94863 -2.77542 -0.89818<br>H 1.94863 -2.77542 0.89818<br>H -2.91321 1.77602 0.00000<br>H -2.46895 -1.25144 0.00000	C_s	-858.87231 (3.0)	saddle point			

5-adduct, conf8	C -1.14788 0.15946 0.00000<br>C -1.02329 1.54878 0.00000<br>C 0.23136 2.15190 0.00000<br>C 1.38554 1.36522 0.00000<br>C 1.25721 -0.02798 0.00000<br>C 0.00000 -0.64101 0.00000<br>H 0.30481 3.23719 0.00000<br>H 2.15648 -0.63482 0.00000<br>O -2.37560 -0.42988 0.00000<br>O -2.20743 2.22850 0.00000<br>C 2.75023 2.00017 0.00000<br>H 3.32782 1.70063 0.88132<br>H 3.32782 1.70063 -0.88132<br>H 2.67970 3.09093 0.00000<br>S -0.27254 -2.39006 0.00000<br>C 1.40343 -3.05866 0.00000<br>H 1.28254 -4.14405 0.00000<br>H 1.95468 -2.76651 -0.89674<br>H 1.95468 -2.76651 0.89674<br>H -2.05620 3.18059 0.00000<br>H -3.04720 0.26652 0.00000	C_s	-858.87429 (1.8)	saddle point			

Table S6.3. Reduced anionic form in water.

Structure	Schematic drawing	Symmetry	G_{PCM}	$H_{PCM,RRHO}$	$G_{PCM,RRHO}$	$G_{SMD,M06-2X,large}$	$G_{SMD,RRHO,M06-2X,large}$
6-adduct, 3-phenolate, conf1	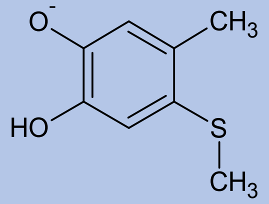<br>2-hydroxy-5-methyl-4-(methylsulfanyl)phenolate<br><br>C -1.63371 -0.86475 -0.06575<br>C -2.08154 0.48281 0.12618<br>C -1.07817 1.47632 0.09451<br>C 0.27967 1.18359 -0.10819<br>C 0.66895 -0.15630 -0.28228<br>C -0.30843 -1.17854 -0.26495	C_1	-858.40266 (0.9)	-858.23763 (0.7)	-858.28760 (1.2)	-859.00756 (0.8)	-858.89250 (0.6)

	H -1.38215 2.51326 0.23223<br>H -0.01003 -2.21454 -0.40830<br>O -2.63096 -1.79778 -0.03675<br>O -3.35143 0.66443 0.30500<br>C 1.27786 2.31246 -0.12535<br>H 0.77180 3.27374 0.00174<br>H 2.01855 2.21689 0.67695<br>H 1.83800 2.34417 -1.06603<br>S 2.36350 -0.61163 -0.56636<br>C 3.00415 -0.82699 1.12351<br>H 4.05135 -1.12972 1.03655<br>H 2.95062 0.10813 1.68653<br>H 2.44922 -1.60718 1.64994<br>H -3.41679 -1.22348 0.11998						
6-adduct, 3-phenolate, conf2	C -0.74638 -1.56496 0.00000<br>C 0.59286 -2.06591 0.00000<br>C 1.60187 -1.08668 0.00000<br>C 1.33217 0.29535 0.00000<br>C 0.00000 0.73089 0.00000<br>C -1.04494 -0.21819 0.00000<br>H 2.64111 -1.41540 0.00000<br>H -2.08740 0.08410 0.00000<br>O -1.70500 -2.53694 0.00000<br>O 0.74069 -3.35980 0.00000<br>C 2.50252 1.24795 0.00000<br>H 3.13609 1.09402 0.88133<br>H 3.13609 1.09402 -0.88133<br>H 2.19591 2.29705 0.00000<br>S -0.33145 2.49178 0.00000<br>C -2.13286 2.58971 0.00000<br>H -2.36766 3.65663 0.00000<br>H -2.56526 2.13591 -0.89532<br>H -2.56526 2.13591 0.89532<br>H -1.13730 -3.34565 0.00000	C_s	-858.39815 (3.7)	saddle point			
6-adduct, 3-phenolate, conf3	C 0.76862 -1.56356 0.00000<br>C -0.56364 -2.07760 0.00000<br>C -1.58684 -1.10948 0.00000<br>C -1.32989 0.27288 0.00000<br>C 0.00000 0.72140 0.00000<br>C 1.05507 -0.21243 0.00000<br>H -2.62136 -1.45104 0.00000<br>H 2.09405 0.10240 0.00000<br>O 1.73910 -2.52557 0.00000	C_s	-858.40016 (2.4)	saddle point			

	O -0.70105 -3.37298 0.00000<br>C -2.47807 1.24719 0.00000<br>H -2.45874 1.90292 0.88017<br>H -2.45874 1.90292 -0.88017<br>H -3.43537 0.71891 0.00000<br>S 0.27704 2.48585 0.00000<br>C 2.07512 2.63591 0.00000<br>H 2.28299 3.70822 0.00000<br>H 2.51851 2.19241 0.89504<br>H 2.51851 2.19241 -0.89504<br>H 1.18091 -3.34036 0.00000						
6-adduct, 4-phenolate, conf1	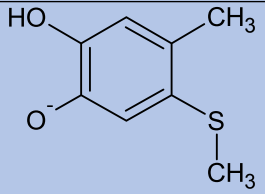<br>2-hydroxy-4-methyl-5-(methylsulfanyl)phenolate<br><br>C -1.66984 -0.93821 -0.06470<br>C -2.01119 0.44062 0.10886<br>C -1.07385 1.45472 0.08566<br>C 0.29398 1.17574 -0.11543<br>C 0.65829 -0.16897 -0.28106<br>C -0.30124 -1.20055 -0.26270<br>H -1.39988 2.48454 0.22156<br>H 0.02181 -2.22984 -0.40302<br>O -2.64442 -1.79879 -0.02518<br>O -3.34220 0.66125 0.29444<br>C 1.28786 2.30664 -0.13518<br>H 0.77275 3.26764 -0.04366<br>H 2.00955 2.24208 0.68830<br>H 1.87162 2.32385 -1.06201<br>S 2.36358 -0.62524 -0.56413<br>C 2.99423 -0.78068 1.13348<br>H 4.04190 -1.08479 1.06183<br>H 2.93751 0.17247 1.66474<br>H 2.43753 -1.54369 1.68255<br>H -3.68661 -0.26411 0.24808	C_1	-858.40095 (1.9)	-858.23595 (1.8)	-858.28629 (2.1)	-859.00609 (1.8)	-858.89142 (1.3)
6-adduct, 4-phenolate, conf2	C -0.83798 -1.59163 0.00000<br>C 0.53124 -2.00390 0.00000<br>C 1.57140 -1.10117 0.00000	C_s	-858.39902 (3.1)	saddle point			

	C 1.33420 0.29081 0.00000<br>C 0.00000 0.72098 0.00000<br>C -1.06422 -0.20056 0.00000<br>H 2.59785 -1.46561 0.00000<br>H -2.09591 0.13681 0.00000<br>O -1.74004 -2.52818 0.00000<br>O 0.70788 -3.35882 0.00000<br>C 2.51791 1.22420 0.00000<br>H 3.15173 1.06687 0.88144<br>H 3.15173 1.06687 -0.88144<br>H 2.22362 2.27687 0.00000<br>S -0.30291 2.48827 0.00000<br>C -2.10177 2.62748 0.00000<br>H -2.31056 3.69986 0.00000<br>H -2.54410 2.18353 -0.89510<br>H -2.54410 2.18353 0.89510<br>H -0.23117 -3.66240 0.00000						
6-adduct, 4-phenolate, conf3	C 0.86216 -1.58853 0.00000<br>C -0.50096 -2.01490 0.00000<br>C -1.55483 -1.12475 0.00000<br>C -1.33161 0.26730 0.00000<br>C 0.00000 0.71159 0.00000<br>C 1.07481 -0.19342 0.00000<br>H -2.57614 -1.50227 0.00000<br>H 2.10244 0.15707 0.00000<br>O 1.77602 -2.51395 0.00000<br>O -0.66351 -3.37289 0.00000<br>C -2.49416 1.22217 0.00000<br>H -2.49265 1.87903 0.88022<br>H -2.49265 1.87903 -0.88022<br>H -3.44152 0.67539 0.00000<br>S 0.24622 2.48178 0.00000<br>C 2.04022 2.67508 0.00000<br>H 2.22070 3.75245 0.00000<br>H 2.49388 2.24224 0.89485<br>H 2.49388 2.24224 -0.89485<br>H 0.27861 -3.66633 0.00000	C_s	-858.40100 (1.9)	-858.23592 (1.8)	-858.28558 (2.5)	-859.00800 (0.6)	-858.89258 (0.5)

5-adduct, 3-phenolate, conf1	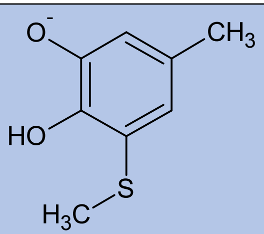<br>2-hydroxy-5-methyl-3-(methylsulfanyl)phenolate<br><br>C -0.20327 0.91392 -0.15646<br>C 1.13443 1.40440 0.03298<br>C 2.15198 0.43702 0.13043<br>C 1.87696 -0.94063 0.05455<br>C 0.56334 -1.37272 -0.11198<br>C -0.49277 -0.43814 -0.22020<br>H 3.17809 0.77683 0.26680<br>H 0.33471 -2.43445 -0.15663<br>O -1.13414 1.89974 -0.26218<br>O 1.26887 2.69588 0.07920<br>C 3.00305 -1.93715 0.16329<br>H 3.73336 -1.80772 -0.64458<br>H 3.55007 -1.82470 1.10688<br>H 2.63046 -2.96473 0.11343<br>S -2.15158 -1.01819 -0.50144<br>C -3.01806 -0.40657 0.97277<br>H -4.06645 -0.69735 0.86909<br>H -2.60777 -0.85987 1.87811<br>H -2.94694 0.68110 1.03038<br>H -0.55195 2.69609 -0.16891	C_1	-858.40297 (0.7)	-858.23773 (0.7)	-858.28762 (1.2)	-859.00697 (1.2)	-858.89162 (1.1)
5-adduct, 3-phenolate, conf2	C -0.06446 1.06751 -0.11440<br>C 1.33471 1.34614 0.10588<br>C 2.16655 0.19547 0.14720<br>C 1.70476 -1.11789 -0.01990<br>C 0.34745 -1.33666 -0.24026<br>C -0.53090 -0.23520 -0.27664<br>H 3.22953 0.36908 0.31475<br>H -0.04347 -2.34030 -0.38487<br>O -0.90904 2.14181 -0.15123<br>O 1.76706 2.54898 0.25251<br>C 2.67272 -2.27242 0.03889<br>H 3.46993 -2.16670 -0.70626	C_1	-858.39486 (5.8)	-858.22962 (5.7)	-858.27996 (6.0)	-859.00354 (3.4)	-858.88864 (3.0)

	H 3.16014 -2.33740 1.01906<br>H 2.16704 -3.22520 -0.14508<br>S -2.27383 -0.47731 -0.57664<br>C -2.89276 -0.84680 1.09385<br>H -3.97171 -0.99799 1.00847<br>H -2.43078 -1.75933 1.47581<br>H -2.69852 -0.01585 1.77496<br>H -1.79339 1.78343 -0.34862						
5-adduct, 3-phenolate, conf3	C -0.06675 1.06839 -0.11652<br>C 1.32767 1.35110 0.10519<br>C 2.16478 0.20081 0.14958<br>C 1.71035 -1.11255 -0.01615<br>C 0.35124 -1.33629 -0.23819<br>C -0.52756 -0.23868 -0.27716<br>H 3.22625 0.37984 0.31922<br>H -0.03754 -2.34122 -0.38088<br>O -0.91828 2.13891 -0.15641<br>O 1.76131 2.55383 0.25222<br>C 2.67686 -2.26979 0.03814<br>H 2.40598 -2.98432 0.82414<br>H 2.69386 -2.82574 -0.90669<br>H 3.69565 -1.92506 0.23864<br>S -2.27060 -0.48673 -0.57665<br>C -2.88849 -0.84286 1.09702<br>H -3.96714 -0.99713 1.01285<br>H -2.42440 -1.75098 1.48698<br>H -2.69629 -0.00553 1.77097<br>H -1.79956 1.77512 -0.35682	C_1	-858.39439 (6.0)	saddle point			
5-adduct, 3-phenolate, conf4	C 1.15570 0.14320 0.00000<br>C 1.14441 1.57410 0.00000<br>C -0.12269 2.19230 0.00000<br>C -1.30617 1.43673 0.00000<br>C -1.24897 0.04155 0.00000<br>C 0.00000 -0.61726 0.00000<br>H -0.17280 3.28043 0.00000<br>H -2.17227 -0.52815 0.00000<br>O 2.40663 -0.40617 0.00000<br>O 2.30522 2.15880 0.00000<br>C -2.64202 2.13721 0.00000<br>H -2.75862 2.78009 0.88052<br>H -2.75862 2.78009 -0.88052<br>H -3.46829 1.41994 0.00000<br>S 0.20344 -2.38498 0.00000	C_s	-858.40267 (0.9)	-858.23738 (0.9)	-858.28761 (1.2)	-859.00837 (0.3)	-858.89332 (0.1)

	C -1.49455 -2.99928 0.00000<br>H -1.41482 -4.08860 0.00000<br>H -2.03736 -2.68648 0.89503<br>H -2.03736 -2.68648 -0.89503<br>H 2.95588 0.41646 0.00000						
5-adduct, 3-phenolate, conf5	C -1.15665 0.14842 0.00000<br>C -1.14227 1.57563 0.00000<br>C 0.12939 2.19128 0.00000<br>C 1.30962 1.43556 0.00000<br>C 1.24896 0.03782 0.00000<br>C 0.00000 -0.61496 0.00000<br>H 0.18108 3.27905 0.00000<br>H 2.17089 -0.53465 0.00000<br>O -2.40827 -0.40254 0.00000<br>O -2.29919 2.16858 0.00000<br>C 2.65258 2.12446 0.00000<br>H 3.24608 1.85277 0.88082<br>H 3.24608 1.85277 -0.88082<br>H 2.53806 3.21238 0.00000<br>S -0.21105 -2.38170 0.00000<br>C 1.48435 -3.00266 0.00000<br>H 1.40065 -4.09169 0.00000<br>H 2.02824 -2.69164 -0.89502<br>H 2.02824 -2.69164 0.89502<br>H -2.95873 0.41828 0.00000	C_s	-858.40222 (1.1)	saddle point			
5-adduct, 3-phenolate, conf6	C 1.19403 0.12930 0.00000<br>C 1.21590 1.56820 0.00000<br>C -0.06440 2.18782 0.00000<br>C -1.26622 1.47551 0.00000<br>C -1.23972 0.07958 0.00000<br>C 0.00000 -0.58768 0.00000<br>H -0.08241 3.27743 0.00000<br>H -2.17046 -0.47722 0.00000<br>O 2.42784 -0.48568 0.00000<br>O 2.32280 2.22466 0.00000<br>C -2.58372 2.20945 0.00000<br>H -2.68357 2.85500 0.88054<br>H -2.68357 2.85500 -0.88054<br>H -3.42819 1.51352 0.00000<br>S 0.14202 -2.36711 0.00000<br>C -1.56502 -2.95008 0.00000<br>H -1.50233 -4.04020 0.00000<br>H -2.09701 -2.62461 0.89643	C_s	-858.38879 (9.6)	saddle point			

	H -2.09701 -2.62461 -0.89643<br>H 2.32197 -1.44486 0.00000						
5-adduct, 3-phenolate, conf7	C -1.19520 0.13424 0.00000<br>C -1.21345 1.56961 0.00000<br>C 0.07115 2.18701 0.00000<br>C 1.26984 1.47429 0.00000<br>C 1.23989 0.07567 0.00000<br>C 0.00000 -0.58559 0.00000<br>H 0.09085 3.27629 0.00000<br>H 2.16912 -0.48410 0.00000<br>O -2.43031 -0.48059 0.00000<br>O -2.31606 2.23393 0.00000<br>C 2.59351 2.19778 0.00000<br>H 3.19373 1.94053 0.88073<br>H 3.19373 1.94053 -0.88073<br>H 2.45081 3.28238 0.00000<br>S -0.14945 -2.36438 0.00000<br>C 1.55502 -2.95422 0.00000<br>H 1.48819 -4.04409 0.00000<br>H 2.08816 -2.63058 -0.89643<br>H 2.08816 -2.63058 0.89643<br>H -2.32512 -1.43976 0.00000	C_s	-858.38857 (9.7)	saddle point			
5-adduct, 4-phenolate, conf1	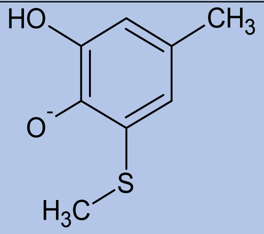<br>2-hydroxy-4-methyl-6-(methylsulfanyl)phenolate<br><br>C -0.24632 0.97110 -0.16432<br>C 1.13478 1.31847 0.02683<br>C 2.14732 0.38857 0.12578<br>C 1.84987 -0.99086 0.05365<br>C 0.52094 -1.36449 -0.11116<br>C -0.51940 -0.41668 -0.22280<br>H 3.17406 0.72498 0.26112<br>H 0.26403 -2.42133 -0.15658<br>O -1.08736 1.95157 -0.25680<br>O 1.35244 2.66331 0.08055	C_1	-858.40402 (0.0)	-858.23879 (0.0)	-858.28958 (0.0)	-859.00744 (0.9)	-858.89299 (0.3)

	C 2.94894 -2.01428 0.16547<br>H 3.69623 -1.89899 -0.62964<br>H 3.48612 -1.93403 1.11872<br>H 2.54789 -3.03011 0.09651<br>S -2.17829 -0.98200 -0.50666<br>C -3.03714 -0.37304 0.97364<br>H -4.10246 -0.58020 0.84304<br>H -2.67777 -0.88467 1.86989<br>H -2.88025 0.70375 1.06383<br>H 0.43033 3.00067 -0.03272						
5-adduct, 4-phenolate, conf2	C 0.84755 0.46000 0.00000<br>C 0.17390 1.72925 0.00000<br>C -1.19718 1.88154 0.00000<br>C -2.01922 0.73566 0.00000<br>C -1.40365 -0.51144 0.00000<br>C 0.00000 -0.66898 0.00000<br>H -1.63191 2.87953 0.00000<br>H -2.02807 -1.40483 0.00000<br>O 2.14830 0.50959 0.00000<br>O 1.04080 2.78036 0.00000<br>C -3.51899 0.87084 0.00000<br>H -3.87828 1.41780 0.88061<br>H -3.87828 1.41780 -0.88061<br>H -4.00383 -0.11030 0.00000<br>S 0.53338 -2.37497 0.00000<br>C 2.34425 -2.40772 0.00000<br>H 2.59607 -3.47223 0.00000<br>H 2.75764 -1.93333 -0.88849<br>H 2.75764 -1.93333 0.88849<br>H 1.90218 2.28376 0.00000	C_s	-858.39603 (5.0)	saddle point			
5-adduct, 4-phenolate, conf3	C -0.84662 0.46534 0.00000<br>C -0.17069 1.72902 0.00000<br>C 1.20344 1.87656 0.00000<br>C 2.02171 0.73145 0.00000<br>C 1.40142 -0.51611 0.00000<br>C 0.00000 -0.66799 0.00000<br>H 1.64101 2.87310 0.00000<br>H 2.02255 -1.41211 0.00000<br>O -2.14767 0.51362 0.00000<br>O -1.03185 2.78660 0.00000<br>C 3.52462 0.85270 0.00000<br>H 3.97385 0.37859 0.88103<br>H 3.97385 0.37859 -0.88103	C_s	-858.39535 (5.4)	saddle point			

	H 3.83289 1.90276 0.00000<br>S -0.54152 -2.37133 0.00000<br>C -2.35251 -2.39894 0.00000<br>H -2.60657 -3.46295 0.00000<br>H -2.76470 -1.92362 0.88850<br>H -2.76470 -1.92362 -0.88850<br>H -1.89598 2.29657 0.00000						
5-adduct, 4-phenolate, conf4	C 1.21811 0.08902 0.00000<br>C 1.10429 1.51058 0.00000<br>C -0.10773 2.17592 0.00000<br>C -1.30779 1.43928 0.00000<br>C -1.23494 0.04486 0.00000<br>C 0.00000 -0.62700 0.00000<br>H -0.12582 3.26433 0.00000<br>H -2.16218 -0.52178 0.00000<br>O 2.41256 -0.42717 0.00000<br>O 2.30706 2.15792 0.00000<br>C -2.63740 2.14788 0.00000<br>H -2.75713 2.79114 0.88059<br>H -2.75713 2.79114 -0.88059<br>H -3.46540 1.43237 0.00000<br>S 0.18994 -2.39535 0.00000<br>C -1.51595 -2.98938 0.00000<br>H -1.45522 -4.08001 0.00000<br>H -2.05714 -2.66874 0.89400<br>H -2.05714 -2.66874 -0.89400<br>H 2.92959 1.39285 0.00000	C_s	-858.40388 (0.1)	-858.23852 (0.2)	-858.28843 (0.7)	-859.00889 (0.0)	-858.89344 (0.0)
5-adduct, 4-phenolate, conf5	C -1.21912 0.09485 0.00000<br>C -1.10123 1.51242 0.00000<br>C 0.11553 2.17445 0.00000<br>C 1.31185 1.43703 0.00000<br>C 1.23518 0.04020 0.00000<br>C 0.00000 -0.62541 0.00000<br>H 0.13673 3.26265 0.00000<br>H 2.16067 -0.52978 0.00000<br>O -2.41308 -0.42316 0.00000<br>O -2.30087 2.16828 0.00000<br>C 2.64889 2.13404 0.00000<br>H 3.24768 1.87243 0.88092<br>H 3.24768 1.87243 -0.88092<br>H 2.52413 3.22121 0.00000<br>S -0.19884 -2.39227 0.00000<br>C 1.50429 -2.99383 0.00000	C_s	-858.40341 (0.4)	saddle point			

	H 1.43899 -4.08419 0.00000<br>H 2.04673 -2.67528 -0.89402<br>H 2.04673 -2.67528 0.89402<br>H -2.92872 1.40856 0.00000						

Table S6.4. One-electron oxidized neutral form in vacuo.

Structure	Schematic drawing	Symmetry	E	H_{RRHO}	G_{RRHO}	$E_{M06\text{-}2X,large}$	$G_{RRHO,M06\text{-}2X,large}$
6-adduct, 3-phenoxyl, conf1	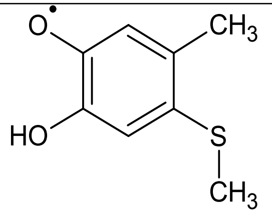<br>[2-hydroxy-5-methyl-4-(methylsulfanyl)phenyl]oxidanyl<br><br>C -0.83265 -1.54090 0.00000<br>C 0.52453 -2.07333 0.00000<br>C 1.58832 -1.11528 0.00000<br>C 1.35841 0.23965 0.00000<br>C 0.00000 0.71366 0.00000<br>C -1.07942 -0.18412 0.00000<br>H 2.60403 -1.50181 0.00000<br>H -2.10814 0.15485 0.00000<br>O -1.80663 -2.44690 0.00000<br>O 0.65835 -3.32079 0.00000<br>C 2.53526 1.18123 0.00000<br>H 3.15948 1.01224 0.88318<br>H 3.15948 1.01224 -0.88318<br>H 2.24669 2.23383 0.00000<br>S -0.23652 2.44838 0.00000<br>C -2.02768 2.64595 0.00000<br>H -2.19848 3.72465 0.00000<br>H -2.48040 2.21832 -0.89778<br>H -2.48040 2.21832 0.89778<br>H -1.33225 -3.30638 0.00000	C_s	-858.23947<br>(1.8)	saddle point			
6-adduct, 3-phenoxyl, conf2	C 0.84938 -1.53991 0.00000<br>C -0.50338 -2.08283 0.00000<br>C -1.57856 -1.13460 0.00000	C_s	-858.24227<br>(0.0)	-858.07562<br>(0.1)	-858.12479<br>(1.1)	-858.83804<br>(0.0)	-858.72055<br>(0.5)

	C -1.35633 0.22013 0.00000<br>C 0.00000 0.70485 0.00000<br>C 1.08742 -0.18019 0.00000<br>H -2.59010 -1.53036 0.00000<br>H 2.11328 0.16816 0.00000<br>O 1.83051 -2.43831 0.00000<br>O -0.62724 -3.33127 0.00000<br>C -2.50657 1.18542 0.00000<br>H -2.48533 1.83630 0.88190<br>H -2.48533 1.83630 -0.88190<br>H -3.45677 0.64715 0.00000<br>S 0.19266 2.44250 0.00000<br>C 1.97879 2.68207 0.00000<br>H 2.12728 3.76401 0.00000<br>H 2.44031 2.26355 0.89759<br>H 2.44031 2.26355 -0.89759<br>H 1.36311 -3.30157 0.00000						
6-adduct, 4-phenoxyl, conf1	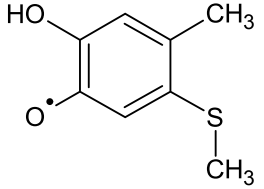<br>[2-hydroxy-4-methyl-5-(methylsulfanyl)phenyl]oxidanyl<br><br>C -1.66226 -0.95710 -0.06013<br>C -2.04325 0.43912 0.11356<br>C -1.10086 1.45402 0.08093<br>C 0.24975 1.15733 -0.11757<br>C 0.65963 -0.21265 -0.26920<br>C -0.26567 -1.22970 -0.25390<br>H -1.42497 2.48344 0.20142<br>H 0.03554 -2.26392 -0.38634<br>O -2.57241 -1.81983 -0.02351<br>O -3.33692 0.66959 0.29560<br>C 1.24530 2.27257 -0.18615<br>H 1.80480 2.23897 -1.12647<br>H 0.75067 3.24273 -0.10196<br>H 1.98861 2.19805 0.61548<br>S 2.37242 -0.62716 -0.55422<br>C 2.99683 -0.65979 1.15153<br>H 4.05308 -0.93361 1.08908	C_1	-858.23482 (4.7)	-858.06852 (4.6)	-858.11919 (4.6)	-858.83023 (4.9)	-858.71460 (4.3)

	H 2.91619 0.31702 1.63469<br>H 2.46877 -1.41094 1.74293<br>H -3.75353 -0.21804 0.26742						
6-adduct, 4-phenoxyl, conf2	C -0.95501 -1.52580 0.00000<br>C 0.40620 -2.05425 0.00000<br>C 1.50451 -1.21209 0.00000<br>C 1.33522 0.17192 0.00000<br>C 0.00000 0.72348 0.00000<br>C -1.10135 -0.10228 0.00000<br>H 2.50431 -1.63741 0.00000<br>H -2.11692 0.27435 0.00000<br>O -1.89387 -2.35646 0.00000<br>O 0.50949 -3.37713 0.00000<br>C 2.56242 1.03660 0.00000<br>H 3.17705 0.82505 0.88202<br>H 3.17705 0.82505 -0.88202<br>H 2.33944 2.10443 0.00000<br>S -0.12119 2.48432 0.00000<br>C -1.89884 2.76926 0.00000<br>H -2.02087 3.85460 0.00000<br>H -2.37140 2.36095 -0.89702<br>H -2.37140 2.36095 0.89702<br>H -0.42209 -3.68941 0.00000	C_s	-858.23761 (3.0)	saddle point			
6-adduct, 4-phenoxyl, conf3	C 0.96809 -1.52567 0.00000<br>C -0.38997 -2.06122 0.00000<br>C -1.49793 -1.22622 0.00000<br>C -1.33394 0.15523 0.00000<br>C 0.00000 0.71401 0.00000<br>C 1.10790 -0.10111 0.00000<br>H -2.49385 -1.65906 0.00000<br>H 2.12021 0.28458 0.00000<br>O 1.91067 -2.35282 0.00000<br>O -0.48571 -3.38413 0.00000<br>C -2.53271 1.05191 0.00000<br>H -2.54096 1.70592 0.88030<br>H -2.54096 1.70592 -0.88030<br>H -3.45525 0.46771 0.00000<br>S 0.08501 2.47459 0.00000<br>C 1.85692 2.79540 0.00000<br>H 1.95897 3.88271 0.00000<br>H 2.33684 2.39558 0.89691<br>H 2.33684 2.39558 -0.89691<br>H 0.44815 -3.69071 0.00000	C_s	-858.23999 (1.5)	-858.07349 (1.5)	-858.12275 (2.3)	-858.83526 (1.7)	-858.71801 (2.1)

5-adduct, 3-phenoxyl, conf1	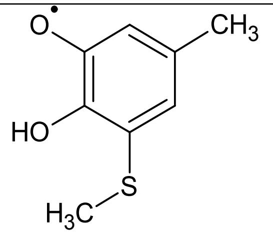<br>[2-hydroxy-5-methyl-3-(methylsulfanyl)phenyl]oxidanyl<br><br>C -0.22097 0.92122 -0.14375<br>C 1.14891 1.41118 0.02489<br>C 2.20158 0.44098 0.11982<br>C 1.91979 -0.90055 0.06232<br>C 0.56356 -1.32813 -0.07377<br>C -0.51021 -0.44616 -0.18163<br>H 3.21618 0.81066 0.23400<br>H 0.35179 -2.39432 -0.09381<br>O -1.14507 1.86321 -0.25797<br>O 1.29948 2.65539 0.05505<br>C 2.99719 -1.94234 0.15513<br>H 3.02009 -2.56407 -0.74699<br>H 3.98199 -1.48574 0.27777<br>H 2.82384 -2.61205 1.00492<br>S -2.14243 -1.07237 -0.46515<br>C -3.07653 -0.31060 0.89144<br>H -4.09920 -0.68362 0.79670<br>H -2.67523 -0.61507 1.86054<br>H -3.08007 0.77656 0.80309<br>H -0.63576 2.70309 -0.19728	C_1	-858.23541 (4.3)	-858.06892 (4.3)	-858.11914 (4.6)	-858.82987 (5.1)	-858.71360 (4.9)
5-adduct, 3-phenoxyl, conf2	C -0.10523 1.07427 -0.12935<br>C 1.31261 1.35977 0.11133<br>C 2.19792 0.21785 0.15789<br>C 1.75900 -1.07252 -0.01149<br>C 0.37358 -1.29530 -0.23459<br>C -0.54555 -0.24757 -0.27860<br>H 3.24701 0.44068 0.33230<br>H 0.01188 -2.31020 -0.38059<br>O -0.92813 2.11276 -0.19228<br>O 1.71456 2.52849 0.26408<br>C 2.69702 -2.24606 0.02900<br>H 2.67728 -2.79946 -0.91653	C_1	-858.22667 (9.8)	-858.06057 (9.6)	-858.11122 (9.6)	-858.82106 (10.7)	-858.70562 (9.9)

	H 3.72514 -1.92355 0.20945<br>H 2.41849 -2.94821 0.82273<br>S -2.27840 -0.52873 -0.57402<br>C -2.86931 -0.82654 1.12155<br>H -3.95130 -0.96512 1.05600<br>H -2.41686 -1.73201 1.53067<br>H -2.65529 0.02404 1.77188<br>H -1.81371 1.76023 -0.41033						
5-adduct, 3-phenoxyl, conf3	C 0.10825 -1.07434 -0.12838<br>C -1.31118 -1.35774 0.11156<br>C -2.19471 -0.21727 0.15656<br>C -1.75489 1.07619 -0.01406<br>C -0.37311 1.29491 -0.23586<br>C 0.54809 0.24469 -0.27837<br>H -3.24421 -0.44005 0.33086<br>H -0.00902 2.30842 -0.38336<br>O 0.92858 -2.11503 -0.18879<br>O -1.71408 -2.52690 0.26515<br>C -2.70887 2.23985 0.02867<br>H -3.47787 2.14690 -0.74514<br>H -3.22184 2.29378 0.99452<br>H -2.18988 3.18910 -0.12826<br>S 2.28007 0.52732 -0.57410<br>C 2.87044 0.83634 1.11972<br>H 3.95177 0.97939 1.05318<br>H 2.41416 1.74182 1.52453<br>H 2.66021 -0.01187 1.77433<br>H 1.81539 -1.76492 -0.40504	C_1	-858.22592 (10.3)	saddle point			
5-adduct, 3-phenoxyl, conf4	C 1.17840 0.11414 0.00000<br>C 1.16135 1.57570 0.00000<br>C -0.11417 2.22627 0.00000<br>C -1.26810 1.48232 0.00000<br>C -1.20106 0.05515 0.00000<br>C 0.00000 -0.64586 0.00000<br>H -0.12914 3.31200 0.00000<br>H -2.13940 -0.48962 0.00000<br>O 2.37675 -0.44777 0.00000<br>O 2.27954 2.14491 0.00000<br>C -2.61723 2.14979 0.00000<br>H -2.73940 2.78700 0.88200<br>H -2.73940 2.78700 -0.88200<br>H -3.43000 1.41890 0.00000<br>S 0.18162 -2.39945 0.00000	C_s	-858.23395 (5.3)	saddle point			

	C -1.53255 -2.95747 0.00000<br>H -1.48376 -4.04865 0.00000<br>H -2.06669 -2.63381 0.89750<br>H -2.06669 -2.63381 -0.89750<br>H 2.99826 0.31484 0.00000						
5-adduct, 3-phenoxyl, conf5	C -1.17779 0.12158 0.00000<br>C -1.15814 1.58157 0.00000<br>C 0.12135 2.22978 0.00000<br>C 1.27036 1.48305 0.00000<br>C 1.20151 0.05259 0.00000<br>C 0.00000 -0.64333 0.00000<br>H 0.13955 3.31507 0.00000<br>H 2.13987 -0.49301 0.00000<br>O -2.37724 -0.43873 0.00000<br>O -2.27384 2.15411 0.00000<br>C 2.62776 2.12547 0.00000<br>H 3.20503 1.82523 0.88199<br>H 3.20503 1.82523 -0.88199<br>H 2.55127 3.21506 0.00000<br>S -0.19059 -2.39589 0.00000<br>C 1.52083 -2.96190 0.00000<br>H 1.46750 -4.05284 0.00000<br>H 2.05628 -2.64047 -0.89748<br>H 2.05628 -2.64047 0.89748<br>H -2.99802 0.32458 0.00000	C_s	-858.23507 (4.6)	-858.06859 (4.5)	-858.11840 (5.1)	-858.83001 (5.0)	-858.71333 (5.1)
5-adduct, 3-phenoxyl, conf6	C 1.21364 0.08839 0.00000<br>C 1.23134 1.55182 0.00000<br>C -0.04803 2.21939 0.00000<br>C -1.23232 1.52571 0.00000<br>C -1.19604 0.10612 0.00000<br>C 0.00000 -0.61203 0.00000<br>H -0.02010 3.30542 0.00000<br>H -2.14121 -0.42743 0.00000<br>O 2.41198 -0.50132 0.00000<br>O 2.31258 2.17278 0.00000<br>C -2.55857 2.23815 0.00000<br>H -2.66136 2.87840 0.88218<br>H -2.66136 2.87840 -0.88218<br>H -3.39464 1.53350 0.00000<br>S 0.11126 -2.38149 0.00000<br>C -1.60820 -2.91733 0.00000<br>H -1.57290 -4.00870 0.00000<br>H -2.13127 -2.58226 0.89890	C_s	-858.21893 (14.7)	saddle point			

	H -2.13127 -2.58226 -0.89890<br>H 2.32663 -1.46422 0.00000						
5-adduct, 3-phenoxyl, conf7	C -1.21322 0.09680 0.00000<br>C -1.22690 1.55971 0.00000<br>C 0.05728 2.22289 0.00000<br>C 1.23534 1.52501 0.00000<br>C 1.19648 0.10117 0.00000<br>C 0.00000 -0.61028 0.00000<br>H 0.03489 3.30867 0.00000<br>H 2.14114 -0.43403 0.00000<br>O -2.41267 -0.48987 0.00000<br>O -2.30506 2.18392 0.00000<br>C 2.57102 2.21329 0.00000<br>H 3.15893 1.93666 0.88261<br>H 3.15893 1.93666 -0.88261<br>H 2.45437 3.29946 0.00000<br>S -0.12210 -2.37880 0.00000<br>C 1.59435 -2.92355 0.00000<br>H 1.55387 -4.01472 0.00000<br>H 2.11889 -2.59070 -0.89888<br>H 2.11889 -2.59070 0.89888<br>H -2.33042 -1.45308 0.00000	C_s	-858.21988 (14.1)	saddle point			
5-adduct, 4-phenoxyl, conf1	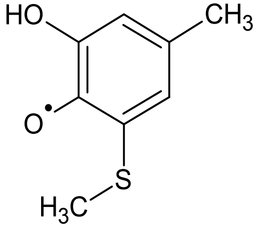<br>[2-hydroxy-4-methyl-6-(methylsulfanyl)phenyl]oxidanyl<br><br>C -0.26735 0.91704 -0.08445<br>C 1.12620 1.35773 0.00274<br>C 2.17443 0.47006 0.05713<br>C 1.90551 -0.91178 0.04475<br>C 0.58010 -1.37589 -0.01143<br>C -0.50838 -0.51283 -0.06886<br>H 3.19400 0.84042 0.10834<br>H 0.39996 -2.44763 0.00454<br>O -1.14158 1.81100 -0.17733<br>O 1.29767 2.67844 0.00717	C_1	-858.24017 (1.4)	-858.07363 (1.4)	-858.12499 (0.9)	-858.83465 (2.1)	-858.71947 (1.2)

	C 3.04427 -1.88451 0.11079<br>H 3.72106 -1.74839 -0.74070<br>H 3.63815 -1.72987 1.01891<br>H 2.69385 -2.91916 0.10449<br>S -2.09464 -1.22150 -0.25551<br>C -3.26219 -0.04409 0.47452<br>H -4.21258 -0.58262 0.50925<br>H -2.96207 0.21932 1.49030<br>H -3.35455 0.85665 -0.12880<br>H 0.39210 3.04550 -0.06813						
5-adduct, 4-phenoxyl, conf2	C -0.26057 0.92496 -0.09836<br>C 1.13325 1.35570 0.00436<br>C 2.18025 0.45906 0.06828<br>C 1.90323 -0.91429 0.05181<br>C 0.56748 -1.37008 -0.01573<br>C -0.51114 -0.50490 -0.08467<br>H 3.20043 0.82511 0.12907<br>H 0.38120 -2.44123 -0.00092<br>O -1.12687 1.82498 -0.20201<br>O 1.31630 2.67316 0.01098<br>C 3.01513 -1.91714 0.12622<br>H 2.89970 -2.56454 1.00315<br>H 3.01235 -2.56905 -0.75496<br>H 3.99183 -1.43193 0.18791<br>S -2.10801 -1.19040 -0.30327<br>C -3.23694 -0.06651 0.56213<br>H -4.20334 -0.57660 0.55165<br>H -2.92079 0.07616 1.59739<br>H -3.30677 0.89471 0.05689<br>H 0.41404 3.04781 -0.07398	C_1	-858.24007 (1.4)	saddle point			
5-adduct, 4-phenoxyl, conf3	C 0.82581 0.46996 0.00000<br>C 0.13233 1.75815 0.00000<br>C -1.23674 1.85522 0.00000<br>C -2.01002 0.67567 0.00000<br>C -1.38729 -0.57829 0.00000<br>C 0.00000 -0.72131 0.00000<br>H -1.70888 2.83308 0.00000<br>H -2.00903 -1.47019 0.00000<br>O 2.08117 0.49975 0.00000<br>O 0.93293 2.82487 0.00000<br>C -3.50557 0.77765 0.00000<br>H -3.85867 1.32634 0.88073<br>H -3.85867 1.32634 -0.88073	C_s	-858.23996 (1.5)	saddle point			

	H -3.97899 -0.20688 0.00000<br>S 0.60893 -2.35531 0.00000<br>C 2.41516 -2.28285 0.00000<br>H 2.72005 -3.33272 0.00000<br>H 2.79832 -1.78367 -0.88907<br>H 2.79832 -1.78367 0.88907<br>H 1.83980 2.45407 0.00000						
5-adduct, 4-phenoxyl, conf4	C -0.82463 0.47494 0.00000<br>C -0.12978 1.76025 0.00000<br>C 1.24341 1.85709 0.00000<br>C 2.01109 0.68044 0.00000<br>C 1.38284 -0.57680 0.00000<br>C 0.00000 -0.71896 0.00000<br>H 1.71521 2.83461 0.00000<br>H 2.00494 -1.46911 0.00000<br>O -2.08010 0.50770 0.00000<br>O -0.92603 2.82881 0.00000<br>C 3.50939 0.75272 0.00000<br>H 3.92562 0.25108 0.88104<br>H 3.92562 0.25108 -0.88104<br>H 3.86176 1.78660 0.00000<br>S -0.61499 -2.35355 0.00000<br>C -2.42128 -2.27528 0.00000<br>H -2.72866 -3.32448 0.00000<br>H -2.80377 -1.77587 0.88920<br>H -2.80377 -1.77587 -0.88920<br>H -1.83426 2.46033 0.00000	C_s	-858.23962 (1.7)	saddle point			
5-adduct, 4-phenoxyl, conf5	C 1.21203 0.13226 0.00000<br>C 1.06027 1.58233 0.00000<br>C -0.17853 2.18216 0.00000<br>C -1.33308 1.37836 0.00000<br>C -1.23179 -0.02718 0.00000<br>C 0.00000 -0.66518 0.00000<br>H -0.25410 3.26548 0.00000<br>H -2.15036 -0.60447 0.00000<br>O 2.37091 -0.34158 0.00000<br>O 2.19992 2.27198 0.00000<br>C -2.68262 2.03251 0.00000<br>H -2.80599 2.67308 0.88083<br>H -2.80599 2.67308 -0.88083<br>H -3.49021 1.29651 0.00000<br>S 0.28191 -2.38322 0.00000<br>C -1.39559 -3.04500 0.00000	C_s	-858.24232 (0.0)	-858.07582 (0.0)	-858.12648 (0.0)	-858.83724 (0.5)	-858.72140 (0.0)

	H -1.28612 -4.13159 0.00000<br>H -1.94652 -2.74854 0.89668<br>H -1.94652 -2.74854 -0.89668<br>H 2.90445 1.59169 0.00000						
5-adduct, 4-phenoxyl, conf6	C -1.21321 0.13567 0.00000<br>C -1.06147 1.58368 0.00000<br>C 0.18051 2.18675 0.00000<br>C 1.33030 1.38547 0.00000<br>C 1.22776 -0.02535 0.00000<br>C 0.00000 -0.66252 0.00000<br>H 0.25411 3.26977 0.00000<br>H 2.14767 -0.60160 0.00000<br>O -2.37241 -0.33800 0.00000<br>O -2.19890 2.27519 0.00000<br>C 2.69182 2.01473 0.00000<br>H 3.26624 1.70632 0.88129<br>H 3.26624 1.70632 -0.88129<br>H 2.63076 3.10533 0.00000<br>S -0.28468 -2.38196 0.00000<br>C 1.39303 -3.04280 0.00000<br>H 1.28444 -4.12950 0.00000<br>H 1.94407 -2.74615 -0.89665<br>H 1.94407 -2.74615 0.89665<br>H -2.90470 1.59577 0.00000	C_s	-858.24223 (0.1)	saddle point			

Table S6.5. One-electron oxidized neutral form in water.

Structure	Schematic drawing	Symmetry	G_{PCM}	$H_{PCM,RRHO}$	$G_{PCM,RRHO}$	$G_{SMD,M06-2X,large}$	$G_{SMD,RRHO,M06-2X,large}$
6-adduct, 3-phenoxyl, conf1	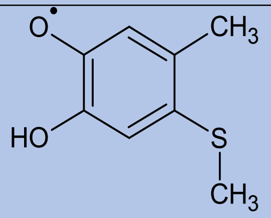<br>[2-hydroxy-5-methyl-4-(methylsulfanyl)phenyl]oxidanyl<br><br>C -0.83181 -1.53927 0.00000<br>C 0.52407 -2.07118 0.00000<br>C 1.58756 -1.11576 0.00000	C_s	-858.24972 (1.9)	saddle point			

	C 1.36041 0.23991 0.00000<br>C 0.00000 0.71566 0.00000<br>C -1.07957 -0.18430 0.00000<br>H 2.60530 -1.49738 0.00000<br>H -2.10706 0.15884 0.00000<br>O -1.81377 -2.44333 0.00000<br>O 0.66273 -3.32412 0.00000<br>C 2.53874 1.17775 0.00000<br>H 3.16239 1.00432 0.88240<br>H 3.16239 1.00432 -0.88240<br>H 2.25357 2.23085 0.00000<br>S -0.23748 2.44356 0.00000<br>C -2.02733 2.65366 0.00000<br>H -2.18654 3.73376 0.00000<br>H -2.47938 2.22924 -0.89883<br>H -2.47938 2.22924 0.89883<br>H -1.35566 -3.30939 0.00000						
6-adduct, 3-phenoxyl, conf2	C 0.84674 -1.53902 0.00000<br>C -0.50507 -2.08023 0.00000<br>C -1.57918 -1.13339 0.00000<br>C -1.35861 0.22192 0.00000<br>C 0.00000 0.70722 0.00000<br>C 1.08666 -0.18137 0.00000<br>H -2.59302 -1.52394 0.00000<br>H 2.11152 0.17026 0.00000<br>O 1.83525 -2.43604 0.00000<br>O -0.63534 -3.33393 0.00000<br>C -2.51049 1.18384 0.00000<br>H -2.48908 1.83436 0.88183<br>H -2.48908 1.83436 -0.88183<br>H -3.45949 0.64415 0.00000<br>S 0.19729 2.43760 0.00000<br>C 1.98225 2.68663 0.00000<br>H 2.12060 3.76954 0.00000<br>H 2.44247 2.27071 0.89866<br>H 2.44247 2.27071 -0.89866<br>H 1.38398 -3.30559 0.00000	C_s	-858.25248 (0.2)	-858.08611 (0.3)	-858.13528 (1.1)	-858.85014 (0.0)	-858.73295 (0.1)
6-adduct, 4-phenoxyl, conf1	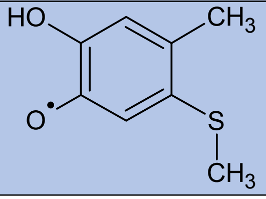	C_s	-858.24734 (3.4)	saddle point			

	[2-hydroxy-4-methyl-5-(methylsulfanyl)phenyl]oxidanyl<br><br>C -0.95584 -1.52310 0.00000<br>C 0.40534 -2.05070 0.00000<br>C 1.50533 -1.21093 0.00000<br>C 1.33817 0.17298 0.00000<br>C 0.00000 0.72338 0.00000<br>C -1.10292 -0.10339 0.00000<br>H 2.50563 -1.63464 0.00000<br>H -2.11544 0.28217 0.00000<br>O -1.89908 -2.35774 0.00000<br>O 0.51644 -3.37569 0.00000<br>C 2.56506 1.03523 0.00000<br>H 3.17889 0.81874 0.88093<br>H 3.17889 0.81874 -0.88093<br>H 2.34515 2.10331 0.00000<br>S -0.12434 2.47973 0.00000<br>C -1.90096 2.77467 0.00000<br>H -2.01333 3.86068 0.00000<br>H -2.37332 2.36940 -0.89782<br>H -2.37332 2.36940 0.89782<br>H -0.40772 -3.70491 0.00000						
6-adduct, 4-phenoxyl, conf2	C 0.96827 -1.52341 0.00000<br>C -0.38972 -2.05777 0.00000<br>C -1.49915 -1.22448 0.00000<br>C -1.33733 0.15667 0.00000<br>C 0.00000 0.71446 0.00000<br>C 1.10882 -0.10228 0.00000<br>H -2.49524 -1.65669 0.00000<br>H 2.11856 0.29097 0.00000<br>O 1.91541 -2.35401 0.00000<br>O -0.49449 -3.38257 0.00000<br>C -2.53688 1.05028 0.00000<br>H -2.54554 1.70351 0.88061<br>H -2.54554 1.70351 -0.88061<br>H -3.45776 0.46448 0.00000<br>S 0.09007 2.47033 0.00000<br>C 1.86105 2.79940 0.00000<br>H 1.95442 3.88713 0.00000<br>H 2.34040 2.40232 0.89772<br>H 2.34040 2.40232 -0.89772<br>H 0.43145 -3.70743 0.00000	C_s	-858.24959 (2.0)	-858.08338 (2.0)	-858.13266 (2.8)	-858.84649 (2.3)	-858.72956 (2.2)

5-adduct, 3-phenoxyl, conf1	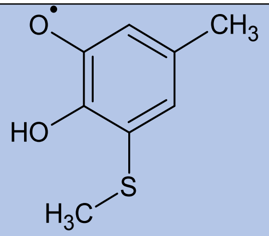<br>[2-hydroxy-5-methyl-3-(methylsulfanyl)phenyl]oxidanyl<br><br>C -0.20846 0.93476 -0.15242<br>C 1.16655 1.40258 0.03323<br>C 2.20181 0.41586 0.13083<br>C 1.90366 -0.92156 0.05789<br>C 0.54160 -1.32951 -0.10078<br>C -0.51432 -0.42867 -0.20792<br>H 3.22230 0.76407 0.26092<br>H 0.31921 -2.39262 -0.13789<br>O -1.12881 1.88067 -0.26608<br>O 1.34001 2.64697 0.07777<br>C 2.96239 -1.97898 0.15499<br>H 2.97613 -2.59925 -0.74787<br>H 3.95307 -1.53879 0.28604<br>H 2.76666 -2.64740 1.00059<br>S -2.16485 -1.01042 -0.49480<br>C -3.04313 -0.36434 0.95755<br>H -4.07798 -0.69972 0.85885<br>H -2.61924 -0.77338 1.87650<br>H -3.01793 0.72590 0.97652<br>H -0.63476 2.72591 -0.19073	C_1	-858.24407 (5.5)	-858.07783 (5.5)	-858.12809 (5.7)	-858.83895 (7.0)	-858.72298 (6.3)
5-adduct, 3-phenoxyl, conf2	C -0.06832 1.08953 -0.10859<br>C 1.35943 1.32729 0.11507<br>C 2.21016 0.16300 0.15068<br>C 1.72610 -1.10964 -0.01990<br>C 0.32904 -1.28710 -0.24097<br>C -0.55637 -0.21411 -0.27303<br>H 3.26866 0.34315 0.31767<br>H -0.05962 -2.29109 -0.38977<br>O -0.85531 2.15792 -0.14530<br>O 1.79550 2.49125 0.26670<br>C 2.61914 -2.31538 0.01211<br>H 3.65927 -2.03361 0.18975	C_1	-858.24045 (7.7)	-858.07436 (7.6)	-858.12480 (7.7)	-858.83584 (9.0)	-858.72019 (8.1)

	H 2.31045 -3.00851 0.80214<br>H 2.56792 -2.86304 -0.93521<br>S -2.29770 -0.42636 -0.57238<br>C -2.89272 -0.88828 1.08383<br>H -3.97344 -1.01910 0.99417<br>H -2.44120 -1.82991 1.40050<br>H -2.68323 -0.10171 1.81038<br>H -1.76601 1.84038 -0.31809						
5-adduct, 3-phenoxyl, conf3	C 1.17904 0.11577 0.00000<br>C 1.16251 1.57704 0.00000<br>C -0.11114 2.22759 0.00000<br>C -1.26730 1.48581 0.00000<br>C -1.20175 0.05676 0.00000<br>C 0.00000 -0.64209 0.00000<br>H -0.13134 3.31345 0.00000<br>H -2.14010 -0.48741 0.00000<br>O 2.37514 -0.45728 0.00000<br>O 2.28298 2.15090 0.00000<br>C -2.61452 2.15353 0.00000<br>H -2.73206 2.79209 0.88141<br>H -2.73206 2.79209 -0.88141<br>H -3.42651 1.42300 0.00000<br>S 0.17649 -2.39792 0.00000<br>C -1.53523 -2.96739 0.00000<br>H -1.47659 -4.05768 0.00000<br>H -2.06676 -2.64423 0.89794<br>H -2.06676 -2.64423 -0.89794<br>H 3.01373 0.28862 0.00000	C_s	-858.24425 (5.3)	saddle point			
5-adduct, 3-phenoxyl, conf4	C -1.17890 0.12256 0.00000<br>C -1.16079 1.58227 0.00000<br>C 0.11636 2.23144 0.00000<br>C 1.26821 1.48745 0.00000<br>C 1.20167 0.05518 0.00000<br>C 0.00000 -0.63928 0.00000<br>H 0.13854 3.31700 0.00000<br>H 2.14043 -0.48891 0.00000<br>O -2.37567 -0.44979 0.00000<br>O -2.27911 2.15897 0.00000<br>C 2.62356 2.12992 0.00000<br>H 3.19897 1.82355 0.88064<br>H 3.19897 1.82355 -0.88064<br>H 2.54737 3.21917 0.00000<br>S -0.18337 -2.39444 0.00000	C_s	-858.24550 (4.6)	-858.07923 (4.6)	-858.12904 (5.1)	-858.84135 (5.5)	-858.72488 (5.1)

	C 1.52646 -2.96929 0.00000<br>H 1.46496 -4.05938 0.00000<br>H 2.05877 -2.64750 -0.89793<br>H 2.05877 -2.64750 0.89793<br>H -3.01417 0.29617 0.00000						
5-adduct, 3-phenoxyl, conf5	C 1.21195 0.09027 0.00000<br>C 1.22662 1.55361 0.00000<br>C -0.04792 2.22348 0.00000<br>C -1.22982 1.52729 0.00000<br>C -1.19273 0.10364 0.00000<br>C 0.00000 -0.61667 0.00000<br>H -0.02852 3.30982 0.00000<br>H -2.13863 -0.42799 0.00000<br>O 2.41476 -0.49233 0.00000<br>O 2.31658 2.17292 0.00000<br>C -2.55675 2.23464 0.00000<br>H -2.65750 2.87573 0.88169<br>H -2.65750 2.87573 -0.88169<br>H -3.38934 1.52719 0.00000<br>S 0.10812 -2.38387 0.00000<br>C -1.61338 -2.92018 0.00000<br>H -1.57223 -4.01112 0.00000<br>H -2.13389 -2.58527 0.89952<br>H -2.13389 -2.58527 -0.89952<br>H 2.34311 -1.45814 0.00000	C_s	-858.23422 (11.6)	saddle point			
5-adduct, 3-phenoxyl, conf6	C -1.21160 0.09869 0.00000<br>C -1.22274 1.56138 0.00000<br>C 0.05602 2.22733 0.00000<br>C 1.23229 1.52691 0.00000<br>C 1.19301 0.09944 0.00000<br>C 0.00000 -0.61457 0.00000<br>H 0.04139 3.31348 0.00000<br>H 2.13861 -0.43328 0.00000<br>O -2.41529 -0.48106 0.00000<br>O -2.31009 2.18385 0.00000<br>C 2.56846 2.20912 0.00000<br>H 3.15353 1.92233 0.88094<br>H 3.15353 1.92233 -0.88094<br>H 2.45856 3.29579 0.00000<br>S -0.11824 -2.38089 0.00000<br>C 1.60058 -2.92517 0.00000<br>H 1.55478 -4.01591 0.00000<br>H 2.12235 -2.59223 -0.89954	C_s	-858.23539 (10.9)	saddle point			

	H 2.12235 -2.59223 0.89954<br>H -2.34642 -1.44714 0.00000						
5-adduct, 4-phenoxyl, conf1	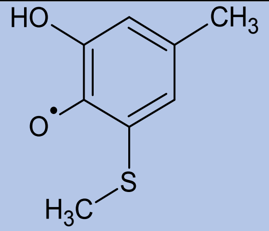<br>[2-hydroxy-4-methyl-6-(methylsulfanyl)phenyl]oxidanyl<br><br>C -0.24052 0.94072 -0.13564<br>C 1.16151 1.33319 0.01573<br>C 2.17905 0.40838 0.09923<br>C 1.86814 -0.96026 0.05779<br>C 0.52003 -1.37761 -0.05090<br>C -0.52853 -0.48061 -0.13515<br>H 3.20829 0.74162 0.19490<br>H 0.30508 -2.44265 -0.05468<br>O -1.08393 1.85947 -0.27161<br>O 1.38730 2.64664 0.04165<br>C 2.96435 -1.97511 0.15231<br>H 3.68038 -1.84656 -0.66735<br>H 3.52595 -1.85341 1.08539<br>H 2.57604 -2.99480 0.11359<br>S -2.15773 -1.09066 -0.39193<br>C -3.17700 -0.12314 0.75741<br>H -4.18613 -0.53066 0.66446<br>H -2.82855 -0.26111 1.78277<br>H -3.17406 0.93225 0.49010<br>H 0.50758 3.06367 -0.06342	C_1	-858.24736 (3.4)	saddle point			
5-adduct, 4-phenoxyl, conf2	C -0.23441 0.94841 -0.14249<br>C 1.16701 1.33216 0.01764<br>C 2.18361 0.39922 0.10664<br>C 1.86662 -0.96156 0.06071<br>C 0.51045 -1.37180 -0.05707<br>C -0.52934 -0.47225 -0.14827<br>H 3.21282 0.72921 0.20955<br>H 0.29146 -2.43636 -0.06448<br>O -1.07302 1.87201 -0.27700<br>O 1.40310 2.64199 0.04718	C_1	-858.24758 (3.3)	-858.08137 (3.2)	-858.13235 (3.0)	-858.84247 (4.8)	-858.72724 (3.6)

	C 2.93603 -2.00365 0.15516<br>H 2.75570 -2.66690 1.00877<br>H 2.93915 -2.63539 -0.74058<br>H 3.92595 -1.55701 0.26681<br>S -2.16748 -1.06212 -0.41704<br>C -3.15256 -0.14745 0.80382<br>H -4.17440 -0.51724 0.69348<br>H -2.80139 -0.36240 1.81490<br>H -3.12156 0.92306 0.60576<br>H 0.52677 3.06654 -0.05975						
5-adduct, 4-phenoxyl, conf3	C 0.82996 0.47173 0.00000<br>C 0.13776 1.76003 0.00000<br>C -1.22985 1.86154 0.00000<br>C -2.00848 0.68347 0.00000<br>C -1.38691 -0.57192 0.00000<br>C 0.00000 -0.71764 0.00000<br>H -1.70341 2.83889 0.00000<br>H -2.01198 -1.46140 0.00000<br>O 2.08689 0.49769 0.00000<br>O 0.93961 2.83129 0.00000<br>C -3.50226 0.78931 0.00000<br>H -3.85123 1.34193 0.87968<br>H -3.85123 1.34193 -0.87968<br>H -3.97777 -0.19370 0.00000<br>S 0.59878 -2.35757 0.00000<br>C 2.40528 -2.31441 0.00000<br>H 2.69158 -3.36894 0.00000<br>H 2.79451 -1.82604 -0.89219<br>H 2.79451 -1.82604 0.89219<br>H 1.84954 2.46974 0.00000	C_s	-858.24649 (3.9)	saddle point			
5-adduct, 4-phenoxyl, conf4	C -0.82854 0.47712 0.00000<br>C -0.13462 1.76258 0.00000<br>C 1.23734 1.86338 0.00000<br>C 2.00978 0.68819 0.00000<br>C 1.38205 -0.57068 0.00000<br>C 0.00000 -0.71493 0.00000<br>H 1.71038 2.84053 0.00000<br>H 2.00775 -1.46028 0.00000<br>O -2.08553 0.50625 0.00000<br>O -0.93112 2.83584 0.00000<br>C 3.50635 0.76341 0.00000<br>H 3.92082 0.25878 0.87990<br>H 3.92082 0.25878 -0.87990	C_s	-858.24618 (4.1)	saddle point			

	H 3.85699 1.79742 0.00000<br>S -0.60589 -2.35595 0.00000<br>C -2.41269 -2.30591 0.00000<br>H -2.70225 -3.35964 0.00000<br>H -2.80112 -1.81702 0.89223<br>H -2.80112 -1.81702 -0.89223<br>H -1.84285 2.47798 0.00000						
5-adduct, 4-phenoxyl, conf5	C 1.21431 0.13162 0.00000<br>C 1.06494 1.58074 0.00000<br>C -0.17146 2.18194 0.00000<br>C -1.33041 1.38080 0.00000<br>C -1.23103 -0.02555 0.00000<br>C 0.00000 -0.66263 0.00000<br>H -0.24933 3.26513 0.00000<br>H -2.14923 -0.60269 0.00000<br>O 2.37495 -0.34984 0.00000<br>O 2.20527 2.27792 0.00000<br>C -2.67620 2.03783 0.00000<br>H -2.79281 2.68076 0.87979<br>H -2.79281 2.68076 -0.87979<br>H -3.48520 1.30451 0.00000<br>S 0.26948 -2.38412 0.00000<br>C -1.40532 -3.05300 0.00000<br>H -1.28849 -4.13833 0.00000<br>H -1.95262 -2.75543 0.89733<br>H -1.95262 -2.75543 -0.89733<br>H 2.92071 1.61147 0.00000	C_s	-858.25280 (0.0)	saddle point			
5-adduct, 4-phenoxyl, conf6	C -1.21581 0.13522 0.00000<br>C -1.06641 1.58262 0.00000<br>C 0.17358 2.18707 0.00000<br>C 1.32720 1.38849 0.00000<br>C 1.22637 -0.02375 0.00000<br>C 0.00000 -0.65953 0.00000<br>H 0.24828 3.27020 0.00000<br>H 2.14624 -0.59936 0.00000<br>O -2.37650 -0.34702 0.00000<br>O -2.20354 2.28201 0.00000<br>C 2.68569 2.01879 0.00000<br>H 3.25902 1.70464 0.87975<br>H 3.25902 1.70464 -0.87975<br>H 2.62465 3.10894 0.00000<br>S -0.27285 -2.38286 0.00000<br>C 1.40282 -3.04939 0.00000	C_s	-858.25277 (0.0)	-858.08653 (0.0)	-858.13710 (0.0)	-858.84871 (0.9)	-858.73304 (0.0)

	H 1.28813 -4.13518 0.00000<br>H 1.95023 -2.75108 -0.89717<br>H 1.95023 -2.75108 0.89717<br>H -2.92061 1.61689 0.00000						

Table S6.6. One-electron oxidized anionic form in water.

**Structure**	**Schematic drawing**	**Symmetry**	G_{PCM}	$H_{PCM,RRHO}$	$G_{PCM,RRHO}$	$G_{SMD,M06\text{-}2X,large}$	$G_{SMD,RRHO,M06\text{-}2X,large}$
6-adduct, conf1	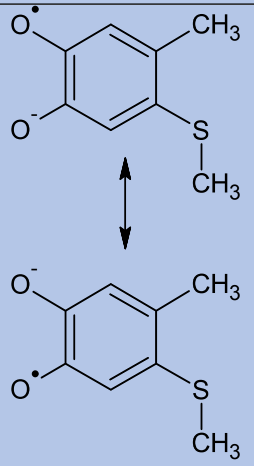<br>[5-methyl-4-(methylsulfanyl)-2-oxidophenyl]oxidanyl<br><br>C -1.72663 -0.98129 -0.04752<br>C -2.15723 0.43528 0.13261<br>C -1.13082 1.43937 0.07718<br>C 0.20616 1.17087 -0.12276<br>C 0.61230 -0.20072 -0.27119<br>C -0.32354 -1.21376 -0.24371<br>H -1.45822 2.47105 0.19627<br>H -0.00800 -2.24717 -0.36716<br>O -2.56134 -1.93029 -0.02235<br>O -3.37055 0.72732 0.32009<br>C 1.20272 2.29200 -0.19279<br>H 1.72915 2.29723 -1.15347<br>H 0.70426 3.25659 -0.06743<br>H 1.97145 2.20542 0.58330<br>S 2.32183 -0.63018 -0.55285	C_1	-857.78002 (2.1)	-857.62727 (1.9)	-857.67782 (1.5)	-858.38357 (3.3)	-858.28138 (2.7)

	C 2.97937 -0.65422 1.14222<br>H 4.03235 -0.93785 1.06816<br>H 2.91031 0.32960 1.61193<br>H 2.45053 -1.39338 1.74788						
6-adduct, conf2	C -0.94304 -1.61144 0.00000<br>C 0.44260 -2.15889 0.00000<br>C 1.51371 -1.20667 0.00000<br>C 1.34265 0.16128 0.00000<br>C 0.00000 0.67633 0.00000<br>C -1.08076 -0.18476 0.00000<br>H 2.52427 -1.61387 0.00000<br>H -2.09957 0.18961 0.00000<br>O -1.94918 -2.37854 0.00000<br>O 0.65370 -3.40645 0.00000<br>C 2.55626 1.05572 0.00000<br>H 3.17822 0.86625 0.88167<br>H 3.17822 0.86625 -0.88167<br>H 2.30652 2.11894 0.00000<br>S -0.18275 2.44056 0.00000<br>C -1.96834 2.68578 0.00000<br>H -2.11452 3.76819 0.00000<br>H -2.43194 2.26573 -0.89575<br>H -2.43194 2.26573 0.89575	C_s	-857.78060 (1.7)	saddle point			
6-adduct, conf3	C 0.96019 -1.60982 0.00000<br>C -0.42098 -2.16685 0.00000<br>C -1.50318 -1.22426 0.00000<br>C -1.33979 0.14314 0.00000<br>C 0.00000 0.66825 0.00000<br>C 1.08851 -0.18140 0.00000<br>H -2.50940 -1.64065 0.00000<br>H 2.10415 0.20203 0.00000<br>O 1.97213 -2.36992 0.00000<br>O -0.62385 -3.41594 0.00000<br>C -2.52950 1.06027 0.00000<br>H -2.53862 1.71457 0.88057<br>H -2.53862 1.71457 -0.88057<br>H -3.45785 0.48385 0.00000<br>S 0.14148 2.43352 0.00000<br>C 1.92119 2.71963 0.00000<br>H 2.04528 3.80465 0.00000<br>H 2.39325 2.30892 0.89570<br>H 2.39325 2.30892 -0.89570	C_s	-857.78331 (0.0)	-857.63036 (0.0)	-857.68014 (0.0)	-858.38881 (0.0)	-858.28565 (0.0)

5-adduct, conf1	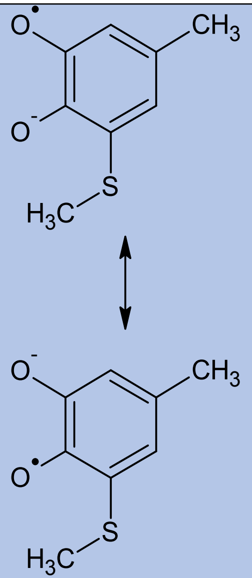<br>[5-methyl-3-(methylsulfanyl)-2-oxidophenyl]oxidanyl<br><br>C -0.23616 1.03643 -0.16596<br>C 1.19515 1.43254 0.02660<br>C 2.17586 0.38889 0.11721<br>C 1.85300 -0.94867 0.05522<br>C 0.48619 -1.32336 -0.09140<br>C -0.52062 -0.38444 -0.19772<br>H 3.21217 0.69894 0.24214<br>H 0.23091 -2.38063 -0.11732<br>O -1.12899 1.91450 -0.29278<br>O 1.50998 2.65378 0.08890<br>C 2.89987 -2.02217 0.15573<br>H 2.91105 -2.65066 -0.74278<br>H 3.89765 -1.59392 0.28214<br>H 2.70511 -2.68748 1.00522<br>S -2.18271 -0.94023 -0.48571<br>C -3.06976 -0.26081 0.94561<br>H -4.12364 -0.52038 0.81790<br>H -2.70151 -0.70297 1.87435<br>H -2.95734 0.82407 0.96901	C_1	-857.78218 (0.7)	-857.62919 (0.7)	-857.67913 (0.6)	-858.38469 (2.6)	-858.28164 (2.5)
5-adduct, conf2	C 0.90393 0.47964 0.00000<br>C 0.28359 1.84648 0.00000	C_s	-857.77485 (5.3)	saddle point			

	C -1.14116 1.94061 0.00000<br>C -1.95648 0.82496 0.00000<br>C -1.37205 -0.46732 0.00000<br>C 0.00000 -0.65414 0.00000<br>H -1.57350 2.94016 0.00000<br>H -2.03096 -1.33409 0.00000<br>O 2.15728 0.36106 0.00000<br>O 1.03313 2.86512 0.00000<br>C -3.45545 0.96328 0.00000<br>H -3.80262 1.51581 0.88047<br>H -3.80262 1.51581 -0.88047<br>H -3.94991 -0.01204 0.00000<br>S 0.50911 -2.35818 0.00000<br>C 2.31672 -2.45958 0.00000<br>H 2.51655 -3.53513 0.00000<br>H 2.74973 -2.00639 -0.89008<br>H 2.74973 -2.00639 0.89008						
5-adduct, conf3	C -0.90409 0.48490 0.00000<br>C -0.28285 1.84750 0.00000<br>C 1.14573 1.94049 0.00000<br>C 1.95808 0.82656 0.00000<br>C 1.37043 -0.46762 0.00000<br>C 0.00000 -0.65109 0.00000<br>H 1.57821 2.93971 0.00000<br>H 2.02909 -1.33520 0.00000<br>O -2.15794 0.36499 0.00000<br>O -1.02668 2.86989 0.00000<br>C 3.45642 0.94392 0.00000<br>H 3.89268 0.45611 0.88007<br>H 3.89268 0.45611 -0.88007<br>H 3.77440 1.99000 0.00000<br>S -0.51254 -2.35474 0.00000<br>C -2.32049 -2.45429 0.00000<br>H -2.52114 -3.53005 0.00000<br>H -2.75391 -2.00100 0.89006<br>H -2.75391 -2.00100 -0.89006	C_s	-857.77565<br>(4.8)	saddle point			
5-adduct, conf4	C 0.92965 0.46706 0.00000<br>C 0.34112 1.84966 0.00000<br>C -1.08037 1.98364 0.00000<br>C -1.92134 0.88888 0.00000<br>C -1.36644 -0.41625 0.00000<br>C 0.00000 -0.64585 0.00000<br>H -1.48590 2.99409 0.00000	C_s	-857.77224<br>(6.9)	saddle point			

	H -2.04827 -1.26540 0.00000<br>O 2.18419 0.33591 0.00000<br>O 1.11700 2.84741 0.00000<br>C -3.41680 1.06033 0.00000<br>H -3.75139 1.62035 0.88062<br>H -3.75139 1.62035 -0.88062<br>H -3.93254 0.09608 0.00000<br>S 0.41409 -2.38055 0.00000<br>C 2.21976 -2.60278 0.00000<br>H 2.69356 -1.62225 0.00000<br>H 2.50373 -3.16458 0.89210<br>H 2.50373 -3.16458 -0.89210						
5-adduct, conf5	C -0.92911 0.47206 0.00000<br>C -0.33887 1.85059 0.00000<br>C 1.08596 1.98341 0.00000<br>C 1.92334 0.88950 0.00000<br>C 1.36496 -0.41715 0.00000<br>C 0.00000 -0.64296 0.00000<br>H 1.49228 2.99322 0.00000<br>H 2.04617 -1.26724 0.00000<br>O -2.18455 0.34170 0.00000<br>O -1.10926 2.85240 0.00000<br>C 3.41879 1.03950 0.00000<br>H 3.86511 0.56147 0.88015<br>H 3.86511 0.56147 -0.88015<br>H 3.71321 2.09230 0.00000<br>S -0.41935 -2.37762 0.00000<br>C -2.22559 -2.59573 0.00000<br>H -2.69657 -1.61377 0.00000<br>H -2.51105 -3.15678 -0.89210<br>H -2.51105 -3.15678 0.89210	C_s	-857.77306 (6.4)	saddle point			
5-adduct, conf6	C 1.28069 0.05522 0.00000<br>C 1.27206 1.54646 0.00000<br>C -0.00282 2.19776 0.00000<br>C -1.19593 1.50384 0.00000<br>C -1.18760 0.07963 0.00000<br>C 0.00000 -0.62180 0.00000<br>H -0.00150 3.28675 0.00000<br>H -2.14177 -0.43882 0.00000<br>O 2.36056 -0.59876 0.00000<br>O 2.36361 2.18569 0.00000<br>C -2.51289 2.23433 0.00000<br>H -2.60903 2.87942 0.88071	C_s	-857.78413 (-0.5)	saddle point			

	H -2.60903 2.87942 -0.88071<br>H -3.35776 1.54011 0.00000<br>S 0.14221 -2.38341 0.00000<br>C -1.58204 -2.91850 0.00000<br>H -1.55690 -4.01055 0.00000<br>H -2.11075 -2.57940 0.89476<br>H -2.11075 -2.57940 -0.89476						
5-adduct, conf7	C -1.28194 0.05839 0.00000<br>C -1.27264 1.54693 0.00000<br>C 0.00469 2.19999 0.00000<br>C 1.19546 1.50713 0.00000<br>C 1.18690 0.08031 0.00000<br>C 0.00000 -0.61880 0.00000<br>H 0.00282 3.28864 0.00000<br>H 2.14149 -0.43808 0.00000<br>O -2.36176 -0.59716 0.00000<br>O -2.36173 2.19015 0.00000<br>C 2.51873 2.22016 0.00000<br>H 3.11478 1.95030 0.88011<br>H 3.11478 1.95030 -0.88011<br>H 2.38628 3.30525 0.00000<br>S -0.14500 -2.38081 0.00000<br>C 1.57872 -2.91748 0.00000<br>H 1.55271 -4.00952 0.00000<br>H 2.10779 -2.57886 -0.89475<br>H 2.10779 -2.57886 0.89475	C_s	-857.78510 (-1.1)	saddle point			

Table S6.7. Two-electron oxidized neutral form in vacuo.

Structure	Schematic drawing	Symmetry	E	H_{RRHO}	G_{RRHO}	$E_{M06\text{-}2X,large}$	$G_{RRHO,M06\text{-}2X,large}$
6-adduct, conf1	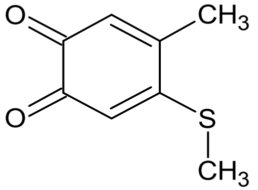<br>4-methyl-5-(methylsulfanyl)cyclohexa-3,5-diene-1,2-dione<br><br>C -1.07499 -1.54827 0.00000<br>C 0.32371 -2.19720 0.00000	C_s	-857.61290 (2.1)	saddle point			

	C 1.47304 -1.29089 0.00000<br>C 1.35456 0.05499 0.00000<br>C 0.00000 0.66811 0.00000<br>C -1.12637 -0.09352 0.00000<br>H 2.45267 -1.76290 0.00000<br>H -2.12217 0.33408 0.00000<br>O -2.07478 -2.24426 0.00000<br>O 0.44573 -3.40657 0.00000<br>C 2.59738 0.90592 0.00000<br>H 3.20472 0.68386 0.88277<br>H 3.20472 0.68386 -0.88277<br>H 2.39537 1.97761 0.00000<br>S -0.02121 2.41823 0.00000<br>C -1.77822 2.80873 0.00000<br>H -1.83647 3.89914 0.00000<br>H -2.27086 2.42600 -0.89728<br>H -2.27086 2.42600 0.89728						
6-adduct, conf2	C 1.08523 -1.54747 0.00000<br>C -0.31179 -2.20342 0.00000<br>C -1.46884 -1.30422 0.00000<br>C -1.35186 0.04115 0.00000<br>C 0.00000 0.66091 0.00000<br>C 1.13152 -0.09215 0.00000<br>H -2.44542 -1.78097 0.00000<br>H 2.12475 0.34183 0.00000<br>O 2.08694 -2.24041 0.00000<br>O -0.42653 -3.41308 0.00000<br>C -2.56700 0.91834 0.00000<br>H -2.58864 1.56909 0.88136<br>H -2.58864 1.56909 -0.88136<br>H -3.47420 0.31132 0.00000<br>S -0.00833 2.41043 0.00000<br>C 1.74222 2.83009 0.00000<br>H 1.78377 3.92122 0.00000<br>H 2.24079 2.45501 0.89722<br>H 2.24079 2.45501 -0.89722	C_s	-857.61629 (0.0)	-857.46154 (0.0)	-857.51061 (0.0)	-858.21566 (0.0)	-858.10998 (0.0)

5-adduct, conf1	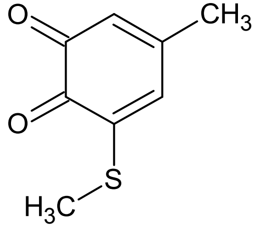<br>5-methyl-3-(methylsulfanyl)cyclohexa-3,5-diene-1,2-dione<br><br>C -0.26006 1.00590 -0.16729<br>C 1.21834 1.46034 0.02571<br>C 2.24486 0.42884 0.06634<br>C 1.91109 -0.88382 0.04845<br>C 0.51230 -1.30586 -0.00005<br>C -0.53951 -0.45383 -0.09260<br>H 3.27688 0.76119 0.13231<br>H 0.31756 -2.37601 0.03520<br>O -1.10903 1.84035 -0.38900<br>O 1.45032 2.64916 0.13204<br>C 2.93846 -1.97023 0.11015<br>H 2.86372 -2.62062 -0.76958<br>H 3.95129 -1.56578 0.15817<br>H 2.77453 -2.60549 0.98879<br>S -2.15849 -1.08964 -0.33700<br>C -3.20511 -0.03557 0.70591<br>H -4.20575 -0.46861 0.63588<br>H -2.87738 -0.07115 1.74691<br>H -3.21752 0.98996 0.34024	C_1	-857.61185 (2.8)	-857.45721 (2.7)	-857.50722 (2.1)	-858.21067 (3.1)	-858.10603 (2.5)
5-adduct, conf2	C 0.90706 0.46959 0.00000<br>C 0.24787 1.88641 0.00000<br>C -1.19759 1.96300 0.00000<br>C -1.95298 0.83375 0.00000<br>C -1.34240 -0.48515 0.00000<br>C 0.00000 -0.71172 0.00000<br>H -1.64185 2.95436 0.00000<br>H -2.01889 -1.33749 0.00000<br>O 2.11531 0.39055 0.00000<br>O 0.98667 2.85401 0.00000<br>C -3.45446 0.91056 0.00000<br>H -3.81276 1.45251 0.88129<br>H -3.81276 1.45251 -0.88129	C_s	-857.60776 (5.4)	saddle point			

	H -3.91646 -0.07921 0.00000<br>S 0.55136 -2.37608 0.00000<br>C 2.36115 -2.38437 0.00000<br>H 2.60763 -3.44965 0.00000<br>H 2.77276 -1.91232 -0.89098<br>H 2.77276 -1.91232 0.89098						
5-adduct, conf3	C -0.90495 0.47543 0.00000<br>C -0.24384 1.89098 0.00000<br>C 1.20409 1.96682 0.00000<br>C 1.95429 0.83657 0.00000<br>C 1.34133 -0.48400 0.00000<br>C 0.00000 -0.70849 0.00000<br>H 1.64970 2.95697 0.00000<br>H 2.01931 -1.33589 0.00000<br>O -2.11325 0.39932 0.00000<br>O -0.97977 2.85976 0.00000<br>C 3.45072 0.87979 0.00000<br>H 3.85083 0.36289 0.88039<br>H 3.85083 0.36289 -0.88039<br>H 3.82433 1.90545 0.00000<br>S -0.55600 -2.37272 0.00000<br>C -2.36593 -2.37619 0.00000<br>H -2.61630 -3.44052 0.00000<br>H -2.77634 -1.90319 0.89094<br>H -2.77634 -1.90319 -0.89094	C_s	-857.61000 (3.9)	saddle point			
5-adduct, conf4	C 0.93729 0.45966 0.00000<br>C 0.31388 1.89661 0.00000<br>C -1.12756 2.01816 0.00000<br>C -1.91184 0.90967 0.00000<br>C -1.33469 -0.42390 0.00000<br>C 0.00000 -0.69864 0.00000<br>H -1.54234 3.02205 0.00000<br>H -2.03652 -1.25573 0.00000<br>O 2.14547 0.36811 0.00000<br>O 1.08210 2.84048 0.00000<br>C -3.41072 1.02378 0.00000<br>H -3.75525 1.57455 0.88125<br>H -3.75525 1.57455 -0.88125<br>H -3.89724 0.04592 0.00000<br>S 0.44146 -2.39908 0.00000<br>C 2.25029 -2.57865 0.00000<br>H 2.73659 -1.60694 0.00000<br>H 2.53304 -3.13895 0.89292	C_s	-857.60403 (7.7)	saddle point			

	H 2.53304 -3.13895 -0.89292						
5-adduct, conf5	C -0.93499 0.46510 0.00000<br>C -0.30975 1.90176 0.00000<br>C 1.13395 2.02261 0.00000<br>C 1.91267 0.91272 0.00000<br>C 1.33369 -0.42262 0.00000<br>C 0.00000 -0.69529 0.00000<br>H 1.55117 3.02495 0.00000<br>H 2.03713 -1.25390 0.00000<br>O -2.14328 0.37645 0.00000<br>O -1.07569 2.84652 0.00000<br>C 3.40750 0.99277 0.00000<br>H 3.81986 0.48603 0.88054<br>H 3.81986 0.48603 -0.88054<br>H 3.75515 2.02752 0.00000<br>S -0.44607 -2.39591 0.00000<br>C -2.25555 -2.57072 0.00000<br>H -2.73920 -1.59770 0.00000<br>H -2.54012 -3.13003 -0.89294<br>H -2.54012 -3.13003 0.89294	C_s	-857.60629 (6.3)	saddle point			
5-adduct, conf6	C 1.29569 0.06823 0.00000<br>C 1.24248 1.62111 0.00000<br>C -0.06868 2.24313 0.00000<br>C -1.20310 1.49650 0.00000<br>C -1.15945 0.03863 0.00000<br>C 0.00000 -0.66802 0.00000<br>H -0.09744 3.32916 0.00000<br>H -2.11606 -0.47520 0.00000<br>O 2.36020 -0.50762 0.00000<br>O 2.29173 2.23787 0.00000<br>C -2.55586 2.15352 0.00000<br>H -2.67557 2.79211 0.88121<br>H -2.67557 2.79211 -0.88121<br>H -3.36767 1.42251 0.00000<br>S 0.19443 -2.39974 0.00000<br>C -1.51968 -2.95539 0.00000<br>H -1.48190 -4.04671 0.00000<br>H -2.05024 -2.62320 0.89690<br>H -2.05024 -2.62320 -0.89690	C_s	-857.61233 (2.5)	saddle point			
5-adduct, conf7	C -1.29558 0.07244 0.00000<br>C -1.24322 1.62550 0.00000<br>C 0.06920 2.24983 0.00000<br>C 1.20059 1.50196 0.00000	C_s	-857.61462 (1.1)	-857.45998 (1.0)	-857.50920 (0.9)	-858.21368 (1.2)	-858.10827 (1.1)

	C 1.15841 0.04170 0.00000<br>C 0.00000 -0.66436 0.00000<br>H 0.09857 3.33537 0.00000<br>H 2.11693 -0.46988 0.00000<br>O -2.36026 -0.50286 0.00000<br>O -2.29164 2.24229 0.00000<br>C 2.56038 2.12855 0.00000<br>H 3.13124 1.81050 0.88070<br>H 3.13124 1.81050 -0.88070<br>H 2.50261 3.21862 0.00000<br>S -0.19657 -2.39692 0.00000<br>C 1.51748 -2.95287 0.00000<br>H 1.47953 -4.04419 0.00000<br>H 2.04829 -2.62105 -0.89691<br>H 2.04829 -2.62105 0.89691						

Table S6.8. Two-electron oxidized neutral form in water.

Structure	Schematic drawing	Symmetry	G_{PCM}	$H_{PCM,RRHO}$	$G_{PCM,RRHO}$	$G_{SMD,M06-2X,large}$	$G_{SMD,RRHO,M06-2X,large}$
6-adduct, conf1	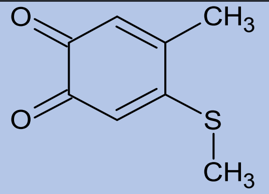<br>4-methyl-5-(methylsulfanyl)cyclohexa-3,5-diene-1,2-dione<br><br>C -1.07552 -1.53283 0.00000<br>C 0.32269 -2.18354 0.00000<br>C 1.47339 -1.29409 0.00000<br>C 1.35644 0.05355 0.00000<br>C 0.00000 0.66920 0.00000<br>C -1.13282 -0.09236 0.00000<br>H 2.45378 -1.76335 0.00000<br>H -2.12394 0.34560 0.00000<br>O -2.07032 -2.25119 0.00000<br>O 0.42531 -3.40121 0.00000<br>C 2.59939 0.89795 0.00000<br>H 3.20464 0.66808 0.88196<br>H 3.20464 0.66808 -0.88196	C_s	-857.62923 (2.0)	saddle point			

	H 2.40408 1.97019 0.00000<br>S -0.01797 2.40756 0.00000<br>C -1.76993 2.81822 0.00000<br>H -1.81018 3.90887 0.00000<br>H -2.26360 2.44205 -0.89877<br>H -2.26360 2.44205 0.89877						
6-adduct, conf2	C 1.08409 -1.53313 0.00000<br>C -0.31290 -2.18971 0.00000<br>C -1.47081 -1.30580 0.00000<br>C -1.35449 0.04104 0.00000<br>C 0.00000 0.66219 0.00000<br>C 1.13709 -0.09229 0.00000<br>H -2.44813 -1.78005 0.00000<br>H 2.12603 0.35095 0.00000<br>O 2.08029 -2.24928 0.00000<br>O -0.41003 -3.40710 0.00000<br>C -2.57036 0.91287 0.00000<br>H -2.59164 1.56264 0.88172<br>H -2.59164 1.56264 -0.88172<br>H -3.47533 0.30354 0.00000<br>S -0.00776 2.39983 0.00000<br>C 1.73822 2.83669 0.00000<br>H 1.76331 3.92777 0.00000<br>H 2.23723 2.46755 0.89868<br>H 2.23723 2.46755 -0.89868	C_s	-857.63241 (0.0)	-857.47783 (0.0)	-857.52701 (0.0)	-858.23252 (0.0)	-858.12712 (0.0)
5-adduct, conf1	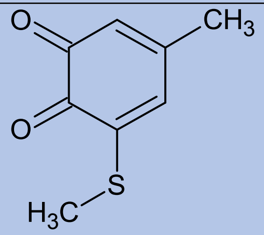<br>5-methyl-3-(methylsulfanyl)cyclohexa-3,5-diene-1,2-dione<br><br>C -0.24250 1.00981 -0.19189<br>C 1.23658 1.44170 0.03913<br>C 2.24511 0.40399 0.09449<br>C 1.89326 -0.90520 0.05085<br>C 0.48610 -1.30708 -0.03358<br>C -0.54832 -0.43774 -0.13147<br>H 3.28132 0.71424 0.19156	C_1	-857.62509 (4.6)	-857.47058 (4.5)	-857.52045 (4.1)	-858.22393 (5.4)	-858.11929 (4.9)

	H 0.27948 -2.37463 -0.01773<br>O -1.06956 1.86500 -0.43642<br>O 1.47372 2.63471 0.15506<br>C 2.89889 -2.00570 0.12078<br>H 2.82341 -2.64434 -0.76722<br>H 3.91684 -1.61992 0.19408<br>H 2.69905 -2.64833 0.98631<br>S -2.18963 -1.02192 -0.38915<br>C -3.15304 -0.08557 0.83288<br>H -4.17438 -0.46361 0.75056<br>H -2.78183 -0.27642 1.84133<br>H -3.13969 0.98096 0.61125						
5-adduct, conf2	C 0.90044 0.46946 0.00000<br>C 0.24005 1.88706 0.00000<br>C -1.19448 1.96804 0.00000<br>C -1.95090 0.83467 0.00000<br>C -1.34224 -0.48517 0.00000<br>C 0.00000 -0.71237 0.00000<br>H -1.64705 2.95545 0.00000<br>H -2.02201 -1.33401 0.00000<br>O 2.11153 0.40146 0.00000<br>O 0.99380 2.85270 0.00000<br>C -3.44860 0.91246 0.00000<br>H -3.80198 1.45925 0.88028<br>H -3.80198 1.45925 -0.88028<br>H -3.91123 -0.07606 0.00000<br>S 0.55099 -2.37756 0.00000<br>C 2.35973 -2.39642 0.00000<br>H 2.59995 -3.46255 0.00000<br>H 2.77094 -1.93000 -0.89405<br>H 2.77094 -1.93000 0.89405	C_s	-857.61998 (7.8)	saddle point			
5-adduct, conf3	C -0.89842 0.47461 0.00000<br>C -0.23654 1.89186 0.00000<br>C 1.20068 1.97282 0.00000<br>C 1.95199 0.83888 0.00000<br>C 1.34105 -0.48299 0.00000<br>C 0.00000 -0.70818 0.00000<br>H 1.65417 2.95931 0.00000<br>H 2.02251 -1.33120 0.00000<br>O -2.10972 0.40971 0.00000<br>O -0.98760 2.85821 0.00000<br>C 3.44507 0.88295 0.00000<br>H 3.84013 0.36031 0.87901	C_s	-857.62221 (6.4)	saddle point			

	H 3.84013 0.36031 -0.87901<br>H 3.81979 1.90767 0.00000<br>S -0.55503 -2.37423 0.00000<br>C -2.36411 -2.38888 0.00000<br>H -2.60666 -3.45452 0.00000<br>H -2.77469 -1.92198 0.89405<br>H -2.77469 -1.92198 -0.89405						
5-adduct, conf4	C 0.92940 0.46039 0.00000<br>C 0.30329 1.89773 0.00000<br>C -1.12765 2.02122 0.00000<br>C -1.91181 0.90802 0.00000<br>C -1.33495 -0.42599 0.00000<br>C 0.00000 -0.69910 0.00000<br>H -1.55203 3.02093 0.00000<br>H -2.03890 -1.25516 0.00000<br>O 2.14047 0.37885 0.00000<br>O 1.08458 2.84030 0.00000<br>C -3.40701 1.02135 0.00000<br>H -3.74706 1.57647 0.88027<br>H -3.74706 1.57647 -0.88027<br>H -3.89314 0.04425 0.00000<br>S 0.44601 -2.39880 0.00000<br>C 2.25413 -2.58670 0.00000<br>H 2.74601 -1.61893 0.00000<br>H 2.53167 -3.14902 0.89273<br>H 2.53167 -3.14902 -0.89273	C_s	-857.61586 (10.4)	saddle point			
5-adduct, conf5	C -0.92755 0.46547 0.00000<br>C -0.30021 1.90276 0.00000<br>C 1.13345 2.02665 0.00000<br>C 1.91256 0.91282 0.00000<br>C 1.33369 -0.42337 0.00000<br>C 0.00000 -0.69482 0.00000<br>H 1.55860 3.02556 0.00000<br>H 2.03955 -1.25166 0.00000<br>O -2.13888 0.38672 0.00000<br>O -1.07912 2.84589 0.00000<br>C 3.40401 0.99139 0.00000<br>H 3.81094 0.47813 0.87906<br>H 3.81094 0.47813 -0.87906<br>H 3.75470 2.02454 0.00000<br>S -0.44954 -2.39579 0.00000<br>C -2.25840 -2.57944 0.00000<br>H -2.74757 -1.61026 0.00000	C_s	-857.61811 (9.0)	saddle point			

	H -2.53800 -3.14068 -0.89274<br>H -2.53800 -3.14068 0.89274						
5-adduct, conf6	C 1.28763 0.06749 0.00000<br>C 1.23806 1.62344 0.00000<br>C -0.05815 2.24830 0.00000<br>C -1.19635 1.49962 0.00000<br>C -1.15728 0.04172 0.00000<br>C 0.00000 -0.66861 0.00000<br>H -0.09439 3.33385 0.00000<br>H -2.11531 -0.46777 0.00000<br>O 2.36277 -0.49793 0.00000<br>O 2.30455 2.22560 0.00000<br>C -2.54352 2.15864 0.00000<br>H -2.65424 2.80011 0.88033<br>H -2.65424 2.80011 -0.88033<br>H -3.35667 1.43083 0.00000<br>S 0.18129 -2.40261 0.00000<br>C -1.53115 -2.96401 0.00000<br>H -1.48479 -4.05449 0.00000<br>H -2.05749 -2.63098 0.89782<br>H -2.05749 -2.63098 -0.89782	C_s	-857.62806 (2.7)	saddle point			
5-adduct, conf7	C -1.28806 0.06997 0.00000<br>C -1.24147 1.62622 0.00000<br>C 0.05546 2.25578 0.00000<br>C 1.19138 1.50756 0.00000<br>C 1.15512 0.04686 0.00000<br>C 0.00000 -0.66432 0.00000<br>H 0.08965 3.34105 0.00000<br>H 2.11569 -0.45901 0.00000<br>O -2.36287 -0.49618 0.00000<br>O -2.30756 2.22701 0.00000<br>C 2.54585 2.13647 0.00000<br>H 3.11470 1.81123 0.87908<br>H 3.11470 1.81123 -0.87908<br>H 2.48695 3.22591 0.00000<br>S -0.18000 -2.40021 0.00000<br>C 1.53381 -2.95801 0.00000<br>H 1.48988 -4.04862 0.00000<br>H 2.05966 -2.62414 -0.89780<br>H 2.05966 -2.62414 0.89780	C_s	-857.63037 (1.3)	-857.47583 (1.3)	-857.52553 (0.9)	-858.22940 (2.0)	-858.12455 (1.6)

Part 7: 4-Methylcatechol – thioacetic acid adducts

Table S7.1. Reduced neutral form in vacuo.

Structure	Schematic drawing	Symmetry	*E*	H_{RRHO}	G_{RRHO}	$E_{M06-2X,large}$	$G_{RRHO,M06-2X,large}$
6-adduct, conf1	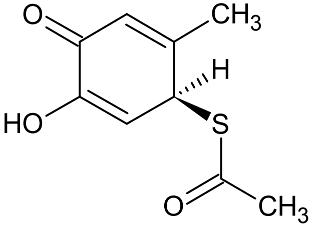 *S*-[(1*S*)-5-hydroxy-2-methyl-4-oxocyclohexa-2,5-dien-1-yl] ethanethioate<br><br>C 2.15586 -0.97864 0.18816<br>C 2.68116 0.36822 -0.12845<br>C 1.73872 1.47639 -0.14703<br>C 0.41948 1.30510 0.07841<br>C -0.13192 -0.06119 0.37288<br>C 0.84959 -1.17759 0.43044<br>H 2.15615 2.46104 -0.33926<br>H -0.70511 -0.00039 1.31461<br>H 0.47685 -2.16902 0.66932<br>O 3.08473 -1.94466 0.22194<br>O 3.88886 0.48415 -0.34095<br>C -0.53682 2.45461 0.08806<br>H -1.11859 2.46882 1.01661<br>H -0.00851 3.40496 -0.01277<br>H -1.25537 2.36940 -0.73529<br>S -1.46028 -0.50745 -0.83217<br>C -2.90451 -0.31105 0.19979<br>C -4.18980 -0.65840 -0.50161<br>H -4.85032 0.21290 -0.46739<br>H -4.03779 -0.96397 -1.53873<br>H -4.67615 -1.46782 0.05094<br>O -2.85276 0.05953 1.35119<br>H 3.92609 -1.49360 0.01545	C_1	-972.06879 (0.0)	-971.87831 (0.0)	-971.93430 (0.0)	-972.77947 (0.0)	-972.64499 (0.0)
6-adduct, conf2	C -2.11849 -0.98573 -0.21786<br>C -2.66623 0.34559 0.12705<br>C -1.74731 1.47533 0.13674	C_1	-972.05681 (7.5)	-971.86641 (7.5)	-971.92153 (8.0)	-972.76924 (6.4)	-972.63396 (6.9)

	C -0.43214 1.33668 -0.12535<br>C 0.15268 -0.02083 -0.41043<br>C -0.81316 -1.15220 -0.48720<br>H -2.18282 2.44822 0.34748<br>H 0.71352 0.04413 -1.35083<br>H -0.42269 -2.13556 -0.73112<br>O -3.02438 -1.97223 -0.24481<br>O -3.87000 0.43472 0.36575<br>C 0.49114 2.51268 -0.16299<br>H 0.95225 2.61377 -1.15357<br>H -0.04305 3.43925 0.05672<br>H 1.30250 2.39743 0.56417<br>S 1.41964 -0.43983 0.88946<br>C 3.03451 -0.42280 0.10825<br>C 3.18397 -0.16239 -1.36787<br>H 2.63443 -0.90064 -1.96032<br>H 2.82057 0.83457 -1.63520<br>H 4.24646 -0.23091 -1.60661<br>O 3.97277 -0.64790 0.83505<br>H -3.87238 -1.54776 -0.01218						
6-adduct, conf3	C 1.87071 -1.13605 0.11134<br>C 2.65081 0.09890 -0.12630<br>C 1.95429 1.37270 -0.01210<br>C 0.63655 1.45314 0.26223<br>C -0.18608 0.20279 0.43100<br>C 0.55816 -1.08535 0.39138<br>H 2.56168 2.26558 -0.13239<br>H -0.73039 0.29911 1.37910<br>H 0.00231 -2.00498 0.54963<br>O 2.57939 -2.26970 0.02728<br>O 3.84876 -0.01085 -0.38483<br>C -0.05434 2.76824 0.43958<br>H -0.51666 2.83128 1.43238<br>H 0.64907 3.59692 0.33639<br>H -0.85847 2.89477 -0.29318<br>S -1.50025 0.21500 -0.89081<br>C -2.98614 -0.45625 -0.14444<br>C -3.00363 -0.85803 1.30709<br>H -2.19995 -1.55900 1.54857<br>H -2.89712 0.02102 1.95170<br>H -3.96822 -1.32687 1.50769<br>O -3.94932 -0.54829 -0.86718<br>H 3.48922 -1.98770 -0.18765	C_1	-972.05712 (7.3)	-971.86669 (7.3)	-971.92186 (7.8)	-972.76930 (6.4)	-972.63405 (6.9)

6-adduct, conf4	C -1.76879 -0.84215 -0.53909<br>C -2.19857 0.13726 0.48378<br>C -1.39878 1.34007 0.65195<br>C -0.26400 1.55374 -0.04226<br>C 0.18015 0.57051 -1.09142<br>C -0.67001 -0.63653 -1.28170<br>H -1.75559 2.05795 1.38540<br>H 0.22387 1.12681 -2.04214<br>H -0.37364 -1.35070 -2.04402<br>O -2.57128 -1.90894 -0.65886<br>O -3.22316 -0.10532 1.12251<br>C 0.55868 2.78782 0.15205<br>H 0.77723 3.27585 -0.80500<br>H 0.04251 3.50160 0.79746<br>H 1.52492 2.54412 0.60779<br>S 1.96421 0.09565 -0.93769<br>C 1.99316 -0.75880 0.63740<br>C 3.32479 -1.39953 0.92960<br>H 3.24901 -2.46642 0.69355<br>H 4.13731 -0.96943 0.33941<br>H 3.53708 -1.30048 1.99656<br>O 1.03741 -0.82244 1.36963<br>H -3.27368 -1.77055 0.00598	C_1	-972.06305 (3.6)	-971.87274 (3.5)	-971.92818 (3.8)	-972.77582 (2.3)	-972.64095 (2.5)
6-adduct, conf5	C -1.79336 -0.79367 -0.57596<br>C -2.23323 0.18634 0.44175<br>C -1.39835 1.35886 0.65973<br>C -0.23986 1.55723 -0.00460<br>C 0.21926 0.56707 -1.03890<br>C -0.65031 -0.62044 -1.26071<br>H -1.76351 2.08073 1.38560<br>H 0.27763 1.12766 -1.98742<br>H -0.33756 -1.34269 -2.00915<br>O -2.61484 -1.84126 -0.73391<br>O -3.28505 -0.03301 1.04306<br>C 0.59140 2.78188 0.21227<br>H 0.80935 3.28036 -0.73941<br>H 0.07956 3.49015 0.86699<br>H 1.56038 2.52777 0.65548<br>S 2.01740 0.11494 -0.90778<br>C 2.28817 -0.88212 0.57020<br>C 1.14952 -1.23824 1.47806<br>H 0.61407 -0.34517 1.81223<br>H 0.43319 -1.87471 0.95013	C_1	-972.05858 (6.4)	-971.86840 (6.2)	-971.92322 (7.0)	-972.77201 (4.7)	-972.63664 (5.2)

	H 1.55755 -1.77351 2.33709<br>O 3.43007 -1.22882 0.74980<br>H -3.34988 -1.68636 -0.10973						
5-adduct, conf1	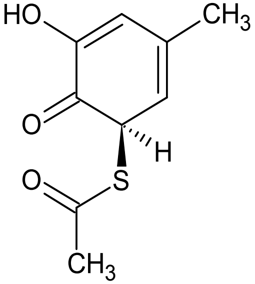<br>*S*-[(1*R*)-5-hydroxy-3-methyl-6-oxocyclohexa-2,4-dien-1-yl] ethanethioate<br><br>C -0.35383 -1.16705 -0.55852<br>C -1.60324 -1.18485 0.21596<br>C -2.29605 -0.04991 0.46158<br>C -1.84113 1.24676 -0.01219<br>C -0.72101 1.35204 -0.75431<br>C 0.07014 0.15627 -1.17292<br>H -3.20505 -0.10672 1.05507<br>H -0.37894 2.31767 -1.11605<br>H -0.06985 0.00870 -2.25694<br>O 0.24547 -2.21873 -0.74398<br>O -1.96666 -2.39266 0.67219<br>C -2.66292 2.44439 0.36481<br>H -3.68947 2.34799 -0.00764<br>H -2.72366 2.54530 1.45472<br>H -2.23687 3.36547 -0.04021<br>S 1.87502 0.41097 -1.05964<br>C 2.06382 0.27722 0.70721<br>C 3.48237 0.44055 1.18303<br>H 3.85935 -0.54516 1.47459<br>H 4.13947 0.85955 0.41805<br>H 3.48336 1.07928 2.06972<br>O 1.12974 0.04473 1.43848<br>H -1.27605 -3.00677 0.36152	C_1	-972.06061<br>(5.1)	-971.87049<br>(4.9)	-971.92636<br>(5.0)	-972.77263<br>(4.3)	-972.63839<br>(4.1)
5-adduct, conf2	C 0.45351 1.20706 -0.48823<br>C 1.75302 1.09493 0.19254<br>C 2.34694 -0.10699 0.38473<br>C 1.75325 -1.34684 -0.08975<br>C 0.58236 -1.32808 -0.76050	C_1	-972.05350<br>(9.6)	-971.86352<br>(9.3)	-971.91787<br>(10.3)	-972.76619<br>(8.3)	-972.63056<br>(9.1)

	C -0.11856 -0.05439 -1.10580<br>H 3.28978 -0.14572 0.92488<br>H 0.13326 -2.24584 -1.12965<br>H 0.03884 0.11175 -2.18708<br>O -0.07476 2.30908 -0.56783<br>O 2.25224 2.25001 0.65475<br>C 2.49109 -2.62144 0.19842<br>H 3.48602 -2.60688 -0.26137<br>H 2.63752 -2.75508 1.27653<br>H 1.95088 -3.49057 -0.18391<br>S -1.94710 -0.16199 -1.06428<br>C -2.41929 -0.30020 0.66481<br>C -1.38555 -0.13375 1.74162<br>H -0.48602 -0.72228 1.53987<br>H -1.10343 0.92239 1.80903<br>H -1.83108 -0.44260 2.68888<br>O -3.59218 -0.48825 0.88009<br>H 1.61470 2.93824 0.38803						
5-adduct, conf3	C 0.53723 1.12145 -0.07173<br>C 2.00360 1.17947 -0.00541<br>C 2.74942 0.04990 0.01241<br>C 2.14999 -1.27350 -0.06599<br>C 0.81174 -1.41178 -0.15808<br>C -0.10587 -0.23954 -0.22658<br>H 3.83019 0.13545 0.09559<br>H 0.35571 -2.39536 -0.22661<br>H -0.60271 -0.21429 -1.21832<br>O -0.09864 2.16841 -0.02865<br>O 2.51996 2.41400 0.09011<br>C 3.07789 -2.45255 -0.04520<br>H 3.77746 -2.41465 -0.88835<br>H 3.67906 -2.46019 0.87155<br>H 2.52732 -3.39463 -0.10041<br>S -1.53195 -0.41613 0.89463<br>C -2.85883 -0.22869 -0.29013<br>C -4.22160 -0.21126 0.34415<br>H -4.92038 -0.75004 -0.29961<br>H -4.22138 -0.64025 1.34865<br>H -4.54961 0.83159 0.40965<br>O -2.66886 -0.11475 -1.47880<br>H 1.75443 3.01796 0.07172	C_1	-972.06307 (3.6)	-971.87291 (3.4)	-971.92871 (3.5)	-972.77158 (5.0)	-972.63722 (4.9)
5-adduct, conf4	C -0.34945 1.07062 0.28276<br>C -1.70938 1.30655 -0.21644	C_1	-972.05557 (8.3)	-971.86529 (8.2)	-971.92022 (8.8)	-972.76675 (8.0)	-972.63140 (8.5)

	C -2.61305 0.30061 -0.26629<br>C -2.28667 -1.05331 0.15997<br>C -1.04142 -1.35698 0.57776<br>C 0.06221 -0.35246 0.58886<br>H -3.61849 0.51352 -0.62077<br>H -0.78578 -2.36720 0.88455<br>H 0.57874 -0.35683 1.55509<br>O 0.40688 2.02928 0.41440<br>O -1.98153 2.57027 -0.57246<br>C -3.38854 -2.06998 0.11944<br>H -4.23110 -1.75878 0.74781<br>H -3.77301 -2.18456 -0.90077<br>H -3.04416 -3.04697 0.46615<br>S 1.28619 -0.91373 -0.68354<br>C 2.91533 -0.34224 -0.16152<br>C 3.09533 0.35240 1.15623<br>H 2.46602 1.24530 1.20880<br>H 2.82114 -0.31468 1.98110<br>H 4.14893 0.62028 1.25070<br>O 3.81460 -0.59186 -0.92693<br>H -1.16720 3.07688 -0.40066						

Table S7.2. Reduced neutral form in water.

Structure	Schematic drawing	Symmetry	G_{PCM}	$H_{PCM,RRHO}$	$G_{PCM,RRHO}$	$G_{SMD,M06\text{-}2X,large}$	$G_{SMD,RRHO,M06\text{-}2X,large}$
6-adduct, conf1	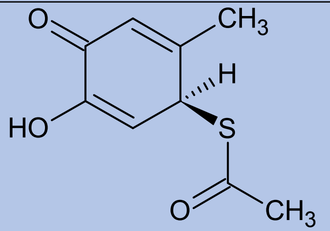<br>*S*-[(1*S*)-5-hydroxy-2-methyl-4-oxocyclohexa-2,5-dien-1-yl] ethanethioate<br><br>C 2.12974 -0.99272 0.17737<br>C 2.68063 0.34331 -0.12478<br>C 1.76685 1.46980 -0.13000<br>C 0.44231 1.32543 0.09591<br>C -0.13751 -0.03211 0.36912	C_1	-972.08027 (0.0)	-971.89041 (0.0)	-971.94696 (0.0)	-972.79217 (0.1)	-972.65885 (0.0)

	C 0.82026 -1.16856 0.41774<br>H 2.20069 2.44949 -0.31041<br>H -0.71057 0.02151 1.31148<br>H 0.42393 -2.15304 0.64816<br>O 3.03836 -1.98649 0.20066<br>O 3.89627 0.43516 -0.33906<br>C -0.48431 2.49653 0.12638<br>H -1.02615 2.53303 1.07827<br>H 0.06112 3.43301 -0.00370<br>H -1.23809 2.41924 -0.66519<br>S -1.46534 -0.43380 -0.84771<br>C -2.90892 -0.33918 0.19993<br>C -4.18779 -0.63933 -0.52531<br>H -4.81784 0.25494 -0.49640<br>H -4.02621 -0.93398 -1.56359<br>H -4.71117 -1.43856 0.00708<br>O -2.85094 -0.05672 1.37848<br>H 3.89274 -1.55944 -0.00107						
6-adduct, conf2	C -1.99599 -1.06456 -0.17267<br>C -2.66262 0.22022 0.12247<br>C -1.86149 1.43004 0.06875<br>C -0.53819 1.40752 -0.19882<br>C 0.16988 0.09837 -0.42098<br>C -0.68329 -1.12074 -0.44792<br>H -2.38423 2.36905 0.22810<br>H 0.73609 0.17896 -1.35616<br>H -0.20258 -2.06996 -0.66508<br>O -2.80465 -2.14046 -0.14931<br>O -3.87263 0.20266 0.37626<br>C 0.26525 2.66089 -0.31632<br>H 0.71535 2.73298 -1.31344<br>H -0.35634 3.54267 -0.15087<br>H 1.08905 2.66926 0.40543<br>S 1.44912 -0.10856 0.91647<br>C 3.01971 -0.44376 0.12884<br>C 3.14595 -0.50418 -1.36369<br>H 2.48979 -1.27003 -1.78735<br>H 2.88980 0.45717 -1.81919<br>H 4.18219 -0.74643 -1.60380<br>O 3.95107 -0.61642 0.88918<br>H -3.69059 -1.79583 0.07184	C_1	-972.07276 (4.7)	-971.88277 (4.8)	-971.93806 (5.6)	-972.78731 (3.1)	-972.65261 (3.9)
6-adduct, conf3	C 1.90065 -1.11503 0.14027<br>C 2.64716 0.12973 -0.13424	C_1	-972.07272 (4.7)	-971.88277 (4.8)	-971.93835 (5.4)	-972.78763 (2.9)	-972.65326 (3.5)

	C 1.92908 1.38820 -0.03920<br>C 0.61031 1.44650 0.24490<br>C -0.18123 0.18130 0.43940<br>C 0.58988 -1.09093 0.42925<br>H 2.51247 2.29433 -0.17731<br>H -0.73727 0.28098 1.37894<br>H 0.05239 -2.01337 0.62883<br>O 2.63610 -2.24064 0.08173<br>O 3.84996 0.03857 -0.40550<br>C -0.10555 2.74623 0.41488<br>H -0.53135 2.81516 1.42292<br>H 0.57091 3.58932 0.26425<br>H -0.94071 2.83290 -0.28828<br>S -1.47516 0.11753 -0.89958<br>C -2.99243 -0.45431 -0.14575<br>C -3.06465 -0.74007 1.32395<br>H -2.30435 -1.46309 1.63186<br>H -2.91939 0.17796 1.90255<br>H -4.05485 -1.14279 1.54169<br>O -3.93416 -0.57945 -0.90252<br>H 3.54032 -1.94954 -0.14252						
6-adduct, conf4	C -1.79518 -0.85136 -0.50684<br>C -2.22712 0.16322 0.47371<br>C -1.41595 1.35462 0.62276<br>C -0.26565 1.53603 -0.06163<br>C 0.17132 0.52429 -1.08376<br>C -0.67896 -0.68791 -1.23330<br>H -1.76887 2.10107 1.32930<br>H 0.18853 1.05344 -2.05076<br>H -0.37762 -1.42846 -1.96827<br>O -2.60743 -1.92166 -0.60735<br>O -3.26860 -0.05100 1.10910<br>C 0.56708 2.76390 0.11371<br>H 0.81667 3.21231 -0.85417<br>H 0.04479 3.50440 0.72240<br>H 1.51726 2.52133 0.60294<br>S 1.95611 0.06905 -0.95394<br>C 2.03851 -0.74101 0.63432<br>C 3.39713 -1.31019 0.93193<br>H 3.34549 -2.39761 0.81225<br>H 4.17248 -0.92111 0.26909<br>H 3.65140 -1.09319 1.97227<br>O 1.08769 -0.82848 1.37977	C_1	-972.07668 (2.3)	-971.88692 (2.2)	-971.94298 (2.5)	-972.79231 (0.0)	-972.65861 (0.2)

	H -3.32827 -1.75741 0.03039						
6-adduct, conf5	C -1.80516 -0.81717 -0.54336<br>C -2.24641 0.20052 0.43214<br>C -1.40633 1.36838 0.62286<br>C -0.23934 1.53884 -0.03720<br>C 0.21001 0.51942 -1.04591<br>C -0.65555 -0.67778 -1.22359<br>H -1.76482 2.11671 1.32449<br>H 0.24924 1.05073 -2.01185<br>H -0.34250 -1.42397 -1.94788<br>O -2.63578 -1.86662 -0.68130<br>O -3.31386 0.01053 1.02716<br>C 0.59845 2.76130 0.15255<br>H 0.85022 3.21428 -0.81270<br>H 0.07568 3.49941 0.76342<br>H 1.54800 2.51486 0.64041<br>S 2.00722 0.07330 -0.92986<br>C 2.30753 -0.84348 0.58068<br>C 1.20512 -1.11443 1.55349<br>H 0.77912 -0.17788 1.92511<br>H 0.40070 -1.67673 1.07113<br>H 1.61195 -1.68907 2.38697<br>O 3.45510 -1.20900 0.73644<br>H -3.37685 -1.69403 -0.06970	C_1	-972.07259 (4.8)	-971.88293 (4.7)	-971.93832 (5.4)	-972.78865 (2.3)	-972.65438 (2.8)
6-adduct, conf6	C 1.80927 -0.82222 0.53543<br>C 2.24826 0.20641 -0.42965<br>C 1.40423 1.37302 -0.61023<br>C 0.23597 1.53328 0.05008<br>C -0.20890 0.50487 1.05155<br>C 0.65922 -0.69236 1.21685<br>H 1.75978 2.12816 -1.30603<br>H -0.24561 1.02844 2.02187<br>H 0.34829 -1.44602 1.93424<br>O 2.64259 -1.87071 0.66363<br>O 3.31668 0.02547 -1.02571<br>C -0.60809 2.75233 -0.13309<br>H -0.86698 3.19622 0.83445<br>H -0.08726 3.49824 -0.73611<br>H -1.55400 2.50395 -0.62711<br>S -2.00514 0.05484 0.93855<br>C -2.30883 -0.83463 -0.58746<br>C -1.21228 -1.06678 -1.57672<br>H -0.81656 -0.11430 -1.94163	C_1	-972.07259 (4.8)	-971.88296 (4.7)	-971.93893 (5.0)	-972.78871 (2.3)	-972.65505 (2.4)

	H -0.38666 -1.61305 -1.11218<br>H -1.61478 -1.64115 -2.41246<br>O -3.45308 -1.21114 -0.74122<br>H 3.38338 -1.69052 0.05391						
5-adduct, conf1	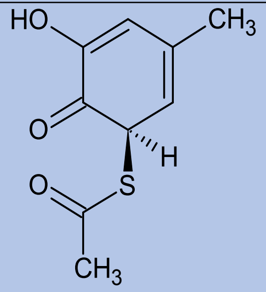<br>*S*-[(1*R*)-5-hydroxy-3-methyl-6-oxocyclohexa-2,4-dien-1-yl] ethanethioate<br><br>C -0.40248 -1.18394 -0.52417<br>C -1.65827 -1.14042 0.22994<br>C -2.30644 0.02568 0.45623<br>C -1.80125 1.29868 -0.03116<br>C -0.66818 1.34421 -0.76090<br>C 0.07203 0.10920 -1.15630<br>H -3.22473 0.01477 1.03857<br>H -0.28831 2.28656 -1.14531<br>H -0.08779 -0.04487 -2.23726<br>O 0.16599 -2.26292 -0.67700<br>O -2.08261 -2.32655 0.70789<br>C -2.58686 2.53094 0.30923<br>H -3.60566 2.46516 -0.08935<br>H -2.67501 2.64856 1.39532<br>H -2.11516 3.42830 -0.09764<br>S 1.88555 0.29401 -1.07003<br>C 2.10393 0.24095 0.69578<br>C 3.51523 0.48746 1.14398<br>H 3.77163 -0.23632 1.92141<br>H 4.23455 0.42689 0.32527<br>H 3.56365 1.49010 1.58183<br>O 1.17713 0.03021 1.45004<br>H -1.43225 -2.98585 0.40443	C_1	-972.07299 (4.6)	-971.88343 (4.4)	-971.93863 (5.2)	-972.78751 (3.0)	-972.65315 (3.6)
5-adduct, conf2	C 0.45464 1.21737 -0.47115<br>C 1.75135 1.09862 0.19964<br>C 2.34317 -0.10673 0.38104<br>C 1.75433 -1.34435 -0.10344	C_1	-972.06719 (8.2)	-971.87771 (8.0)	-971.93223 (9.2)	-972.78232 (6.3)	-972.64735 (7.2)

	C 0.58147 -1.31961 -0.77067<br>C -0.11240 -0.03938 -1.10074<br>H 3.28582 -0.15325 0.92097<br>H 0.13543 -2.23165 -1.15648<br>H 0.04859 0.13380 -2.18048<br>O -0.08688 2.31820 -0.53873<br>O 2.26415 2.24766 0.67694<br>C 2.49777 -2.61825 0.16888<br>H 3.49219 -2.59132 -0.29065<br>H 2.64536 -2.75957 1.24544<br>H 1.95988 -3.48415 -0.22315<br>S -1.94046 -0.13319 -1.05960<br>C -2.42028 -0.31307 0.65557<br>C -1.39434 -0.23198 1.74276<br>H -0.51301 -0.83808 1.51587<br>H -1.07767 0.80991 1.86228<br>H -1.84938 -0.56999 2.67517<br>O -3.60862 -0.46473 0.85475<br>H 1.63652 2.95044 0.42948						
5-adduct, conf3	C 0.55043 1.12555 -0.07676<br>C 2.01033 1.16755 0.01021<br>C 2.74517 0.02990 0.02652<br>C 2.13952 -1.28869 -0.07492<br>C 0.80050 -1.41120 -0.18338<br>C -0.10274 -0.22765 -0.24288<br>H 3.82595 0.10247 0.12290<br>H 0.33399 -2.38804 -0.27320<br>H -0.59580 -0.19049 -1.23477<br>O -0.08320 2.17955 -0.04049<br>O 2.55014 2.39659 0.12241<br>C 3.05807 -2.47462 -0.06057<br>H 3.76753 -2.42779 -0.89451<br>H 3.64928 -2.49701 0.86200<br>H 2.49984 -3.41054 -0.13532<br>S -1.52730 -0.40870 0.88187<br>C -2.86933 -0.20079 -0.27727<br>C -4.22470 -0.27266 0.36159<br>H -4.84269 -0.97535 -0.20359<br>H -4.18235 -0.57569 1.40918<br>H -4.68798 0.71679 0.29342<br>O -2.68919 -0.01635 -1.46291<br>H 1.80346 3.02204 0.09671	C_1	-972.07425 (3.8)	-971.88463 (3.6)	-971.94078 (3.9)	-972.78413 (5.1)	-972.65066 (5.1)

5-adduct, conf4	C -0.38619 1.06856 0.14517<br>C -1.80592 1.28803 -0.13073<br>C -2.68308 0.25681 -0.14066<br>C -2.27848 -1.11103 0.15044<br>C -0.98788 -1.39537 0.41731<br>C 0.07276 -0.34500 0.43819<br>H -3.72753 0.45501 -0.36910<br>H -0.67429 -2.40922 0.64855<br>H 0.53187 -0.32833 1.43554<br>O 0.38310 2.02833 0.13053<br>O -2.15766 2.55945 -0.39863<br>C -3.34911 -2.16100 0.15030<br>H -4.12420 -1.92597 0.88839<br>H -3.84221 -2.21051 -0.82722<br>H -2.93954 -3.14686 0.38097<br>S 1.39434 -0.84631 -0.74275<br>C 2.96026 -0.29908 -0.06495<br>C 3.02806 0.35815 1.27818<br>H 2.38584 1.24242 1.30527<br>H 2.69784 -0.33332 2.06060<br>H 4.06466 0.64052 1.46791<br>O 3.92937 -0.52424 -0.76165<br>H -1.34279 3.08851 -0.32832	C_1	-972.06782 (7.8)	-971.87812 (7.7)	-971.93326 (8.6)	-972.78105 (7.1)	-972.64649 (7.8)
5-adduct, conf5	C 0.80646 -1.19436 0.25502<br>C 2.22328 -0.90850 0.03095<br>C 2.66906 0.36290 -0.10239<br>C 1.78150 1.51292 -0.00981<br>C 0.46150 1.33496 0.19955<br>C -0.14890 -0.01743 0.34810<br>H 3.72812 0.53388 -0.27991<br>H -0.20761 2.18759 0.27475<br>H -0.61197 -0.11323 1.33849<br>O 0.44016 -2.36122 0.37459<br>O 3.02817 -1.98496 -0.04837<br>C 2.39903 2.87240 -0.15041<br>H 3.15984 3.03533 0.62133<br>H 2.89999 2.97198 -1.12008<br>H 1.64826 3.66134 -0.06717<br>S -1.49915 -0.28669 -0.87990<br>C -3.03458 0.12052 -0.05885<br>C -3.05343 0.54068 1.37987<br>H -2.73563 -0.28471 2.02549<br>H -2.39209 1.39237 1.56072	C_1	-972.06582 (9.1)	-971.87608 (9.0)	-971.93254 (9.0)	-972.77937 (8.1)	-972.64609 (8.0)

	H -4.07705 0.81604 1.63743<br>O -4.03163 0.02174 -0.74509<br>H 2.45728 -2.76263 0.08608						

Table S7.3. Reduced zwitterionic form in water.

Structure	Schematic drawing	Symmetry	G_{PCM}	$H_{PCM,RRHO}$	$G_{PCM,RRHO}$	$G_{SMD,M06\text{-}2X,large}$	$G_{SMD,RRHO,M06\text{-}2X,large}$
6-adduct, several starting structures	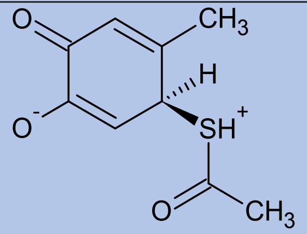<br>(3*S*)-3-(acetylsulfonio)-4-methyl-6-oxocyclohexa-1,4-dien-1-olate	C_1	dissociates to neutral molecules				
5-adduct, several starting structures	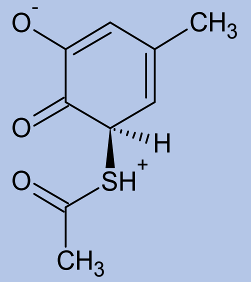<br>(5*R*)-5-(acetylsulfonio)-3-methyl-6-oxocyclohexa-1,3-dien-1-olate	C_1	most starting structures dissociate to neutral molecules				
5-adduct, conf1	C 0.29989 -1.26922 0.42385<br>C 1.62479 -1.43087 -0.26849<br>C 2.30195 -0.23044 -0.49107<br>C 1.92057 1.06025 0.02198<br>C 0.81094 1.24265 0.78181<br>C 0.01879 0.05154 1.15421<br>H 3.19928 -0.28268 -1.10533<br>H 0.58109 2.19331 1.25061<br>H 0.18987 -0.18152 2.21947<br>O -0.55883 -2.14913 0.42435<br>O 1.94625 -2.58008 -0.68755<br>C 2.83615 2.21388 -0.27801<br>H 3.83475 2.03344 0.13571	C_1	-971.98634 (1.0)	-971.80125 (0.7)	-971.85802 (0.1)	-972.70186 (1.6)	-972.57354 (0.6)

	H 2.95715 2.33493 -1.36054<br>H 2.45463 3.15091 0.13468<br>S -1.82041 0.30434 1.14603<br>C -2.08618 0.72421 -0.72496<br>C -2.90349 -0.28334 -1.44142<br>H -2.39195 -1.25179 -1.42209<br>H -3.86702 -0.40488 -0.93571<br>H -3.05374 0.05075 -2.46862<br>O -1.60120 1.73781 -1.09884<br>H -2.00760 -1.05272 1.04410						
5-adduct, conf2	C 0.34762 1.32060 -0.36567<br>C 1.67456 1.36383 0.35151<br>C 2.29258 0.11962 0.49734<br>C 1.85939 -1.10540 -0.11842<br>C 0.74187 -1.18339 -0.89044<br>C -0.00785 0.05991 -1.16681<br>H 3.18758 0.08477 1.11584<br>H 0.49046 -2.07930 -1.44899<br>H 0.12990 0.34699 -2.22291<br>O -0.45152 2.24940 -0.32737<br>O 2.04880 2.46559 0.84195<br>C 2.72264 -2.31846 0.08600<br>H 3.73185 -2.14438 -0.30348<br>H 2.82708 -2.53667 1.15498<br>H 2.30699 -3.19944 -0.40881<br>S -1.86197 -0.13032 -1.11428<br>C -2.23459 -0.46951 0.76606<br>C -1.72895 -1.78126 1.21533<br>H -2.25668 -2.57040 0.66853<br>H -0.66367 -1.87116 0.96280<br>H -1.89859 -1.88168 2.28784<br>O -2.85147 0.37022 1.33017<br>H -2.03333 1.21901 -0.98472	C_1	-971.98699 (0.6)	-971.80194 (0.3)	-971.85822 (0.0)	-972.70079 (2.2)	-972.57203 (1.6)
5-adduct, conf3	C -0.84944 -1.30509 -0.31729<br>C -2.09603 -0.70020 0.29487<br>C -2.12061 0.69732 0.31918<br>C -1.17479 1.55455 -0.34087<br>C -0.09428 1.07292 -1.01431<br>C 0.07909 -0.39176 -1.13217<br>H -2.94420 1.16680 0.85379<br>H 0.55424 1.72315 -1.59331<br>H -0.02703 -0.73304 -2.17425<br>O -0.55517 -2.48136 -0.17433	C_1	-971.98539 (1.6)	-971.79969 (1.7)	-971.85535 (1.8)	-972.70390 (0.3)	-972.57386 (0.4)

	O -2.93691 -1.48622 0.80968<br>C -1.43335 3.03363 -0.28720<br>H -2.39551 3.27285 -0.75374<br>H -1.49053 3.37426 0.75285<br>H -0.65020 3.60050 -0.79610<br>S 1.81298 -0.94278 -0.72926<br>C 2.20392 0.31295 0.72786<br>C 1.33276 0.09390 1.90317<br>H 0.27882 0.23159 1.63408<br>H 1.46453 -0.93166 2.26453<br>H 1.61354 0.80747 2.67892<br>O 3.12333 1.03742 0.55611<br>H 2.45502 -0.19538 -1.64964						
5-adduct, conf4	C -0.20915 0.92231 0.00955<br>C -1.56739 1.56971 0.04562<br>C -2.62425 0.66092 -0.03272<br>C -2.50028 -0.77343 0.02581<br>C -1.30282 -1.40051 0.16836<br>C -0.09690 -0.56016 0.33758<br>H -3.62385 1.07617 -0.14847<br>H -1.22770 -2.47096 0.32921<br>H 0.28202 -0.61381 1.37297<br>O 0.81557 1.52849 -0.29442<br>O -1.63369 2.83257 0.01765<br>C -3.77302 -1.57133 -0.02410<br>H -4.42921 -1.30601 0.81249<br>H -4.32631 -1.35125 -0.94428<br>H -3.57926 -2.64595 0.01452<br>S 1.33645 -1.11517 -0.69011<br>C 2.77155 -0.24326 0.34876<br>C 3.80985 0.31915 -0.53953<br>H 4.29305 -0.49900 -1.08457<br>H 3.33983 0.98257 -1.26975<br>H 4.54295 0.85630 0.06309<br>O 2.66915 -0.28529 1.52493<br>H 1.49163 -2.33180 -0.12454	C_1	-971.98793 (0.0)	-971.80234 (0.0)	-971.85810 (0.1)	-972.70434 (0.0)	-972.57451 (0.0)
5-adduct, conf5	C -0.48293 1.07152 -0.04410<br>C -1.95543 1.40454 -0.01209<br>C -2.78857 0.28434 -0.01174<br>C -2.34639 -1.07536 0.14317<br>C -1.03703 -1.41515 0.29767<br>C -0.03879 -0.32686 0.37320<br>H -3.85621 0.45942 -0.13062	C_1	-971.98435 (2.2)	-971.79862 (2.3)	-971.85497 (2.0)	-972.70074 (2.3)	-972.57135 (2.0)

	H -0.73485 -2.42616 0.55228<br>H 0.37404 -0.23430 1.38916<br>O 0.36883 1.86514 -0.42438<br>O -2.29548 2.61680 -0.11460<br>C -3.40866 -2.13750 0.19248<br>H -4.09256 -1.96028 1.03009<br>H -4.01291 -2.11493 -0.72159<br>H -2.97960 -3.13654 0.29968<br>S 1.41925 -0.69070 -0.73445<br>C 3.12843 -0.26349 0.11598<br>C 3.13145 0.97600 0.91730<br>H 2.59038 1.76818 0.39375<br>H 2.61075 0.78810 1.86358<br>H 4.16603 1.25394 1.12291<br>O 3.98613 -1.03829 -0.13878<br>H 1.53864 -2.00370 -0.45732						

Table S7.4. Reduced anionic form in water.

Structure	Schematic drawing	Symmetry	G_{PCM}	$H_{PCM,RRHO}$	$G_{PCM,RRHO}$	$G_{SMD,M06\text{-}2X,large}$	$G_{SMD,RRHO,M06\text{-}2X,large}$
6-adduct, conf1	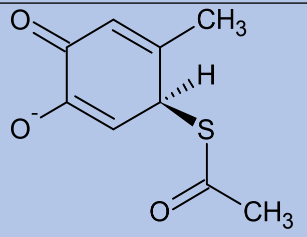<br>(3*S*)-3-(acetylsulfanyl)-4-methyl-6-oxocyclohexa-1,4-dien-1-olate<br><br>C 2.25775 -1.06866 0.22380<br>C 2.71847 0.31478 -0.18422<br>C 1.75833 1.42202 -0.16660<br>C 0.45060 1.27888 0.12643<br>C -0.07773 -0.08291 0.45939<br>C 0.91564 -1.18037 0.53175<br>H 2.16731 2.40369 -0.40088<br>H -0.67815 -0.00595 1.37966<br>H 0.53482 -2.15464 0.83354<br>O 3.12662 -2.00627 0.26853	C_1	-971.59843 (0.0)	-971.42228 (0.0)	-971.47887 (0.0)	-972.31922 (0.0)	-972.19966 (0.0)

	O 3.89149 0.52837 -0.51159<br>C -0.48938 2.44179 0.16877<br>H -0.94913 2.53096 1.15992<br>H 0.02976 3.37519 -0.06020<br>H -1.30843 2.31582 -0.54867<br>S -1.39712 -0.53672 -0.78371<br>C -2.88885 -0.29191 0.14593<br>C -4.13837 -0.63753 -0.61666<br>H -4.72571 0.27674 -0.74800<br>H -3.93049 -1.07388 -1.59526<br>H -4.73197 -1.33627 -0.02014<br>O -2.90472 0.11281 1.29405						
6-adduct, conf2	C -2.23266 -1.05623 -0.27947<br>C -2.69155 0.30808 0.19577<br>C -1.74102 1.42545 0.18181<br>C -0.44256 1.30176 -0.15399<br>C 0.09419 -0.05327 -0.50977<br>C -0.89771 -1.14659 -0.62404<br>H -2.15176 2.39699 0.45170<br>H 0.69638 0.03333 -1.42190<br>H -0.51593 -2.10758 -0.96386<br>O -3.09612 -1.99584 -0.34237<br>O -3.85258 0.49924 0.57141<br>C 0.48245 2.47598 -0.20532<br>H 0.85982 2.62610 -1.22438<br>H -0.02526 3.39117 0.10718<br>H 1.35322 2.32631 0.44337<br>S 1.34484 -0.53683 0.82088<br>C 2.99333 -0.40635 0.16540<br>C 3.22964 0.01187 -1.25712<br>H 2.73745 -0.67466 -1.95289<br>H 2.84361 1.01787 -1.44403<br>H 4.30504 -0.00033 -1.44139<br>O 3.90061 -0.70142 0.92501	C_1	-971.59217 (3.9)	-971.41595 (4.0)	-971.47097 (5.0)	-972.31489 (2.7)	-972.19370 (3.7)
6-adduct, conf3	C 1.88176 -1.28291 0.14814<br>C 2.65500 -0.02641 -0.19779<br>C 2.00167 1.27790 -0.04306<br>C 0.70935 1.43289 0.30353<br>C -0.13944 0.21275 0.51453<br>C 0.56300 -1.08706 0.51364<br>H 2.63870 2.14622 -0.20350<br>H -0.72547 0.36476 1.42910<br>H -0.02992 -1.96613 0.76089	C_1	-971.59248 (3.7)	-971.41623 (3.8)	-971.47130 (4.8)	-972.31442 (3.0)	-972.19323 (4.0)

	O 2.49470 -2.40234 0.09566<br>O 3.82800 -0.08014 -0.58089<br>C 0.09541 2.77954 0.52440<br>H -0.25120 2.87447 1.56080<br>H 0.81602 3.57650 0.32777<br>H -0.78005 2.93181 -0.11625<br>S -1.44527 0.29521 -0.85713<br>C -2.93223 -0.42144 -0.20113<br>C -3.01060 -0.83550 1.24020<br>H -2.20686 -1.52776 1.50359<br>H -2.92968 0.03916 1.89370<br>H -3.97587 -1.31621 1.40616<br>O -3.86956 -0.53561 -0.97264						
6-adduct, conf4	C -1.89521 -1.00750 -0.47056<br>C -2.29137 0.09846 0.48315<br>C -1.47331 1.31048 0.55281<br>C -0.32849 1.49007 -0.13380<br>C 0.09389 0.44199 -1.11788<br>C -0.75312 -0.77143 -1.20917<br>H -1.83438 2.08280 1.23048<br>H 0.13694 0.94907 -2.09719<br>H -0.44516 -1.51738 -1.93993<br>O -2.63981 -2.04754 -0.53966<br>O -3.29942 0.00198 1.19499<br>C 0.49075 2.73522 -0.00130<br>H 0.73209 3.15929 -0.98264<br>H -0.04022 3.48884 0.58500<br>H 1.44623 2.52565 0.49393<br>S 1.90873 0.02112 -0.97513<br>C 2.07699 -0.64444 0.66242<br>C 3.44888 -1.20343 0.93367<br>H 3.37939 -2.29659 0.93201<br>H 4.18502 -0.89862 0.18721<br>H 3.77225 -0.88495 1.92796<br>O 1.17850 -0.65975 1.47833	C_1	-971.59352 (3.1)	-971.41756 (3.0)	-971.47435 (2.8)	-972.31768 (1.0)	-972.19851 (0.7)
6-adduct, conf5	C -1.87491 -0.95475 -0.55020<br>C -2.28149 0.12281 0.43395<br>C -1.44166 1.31605 0.57375<br>C -0.28419 1.51072 -0.08855<br>C 0.16013 0.48126 -1.08235<br>C -0.69359 -0.72144 -1.22931<br>H -1.81128 2.07025 1.26685<br>H 0.23874 1.00878 -2.04849	C_1	-971.59252 (3.7)	-971.41661 (3.6)	-971.47098 (5.0)	-972.31530 (2.5)	-972.19375 (3.7)

	H -0.36873 -1.45244 -1.96734<br>O -2.63554 -1.97358 -0.68369<br>O -3.31130 0.02040 1.10993<br>C 0.53015 2.75445 0.08477<br>H 0.76586 3.20839 -0.88463<br>H -0.00504 3.48711 0.69298<br>H 1.48911 2.53720 0.56918<br>S 1.98814 0.06663 -0.92645<br>C 2.28098 -0.80153 0.60279<br>C 1.17471 -0.97622 1.59296<br>H 0.97467 -0.01868 2.08560<br>H 0.25360 -1.28876 1.09233<br>H 1.48205 -1.70957 2.34073<br>O 3.41559 -1.20887 0.77991						
5-adduct, conf1	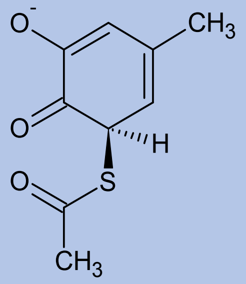<br>(5*R*)-5-(acetylsulfanyl)-3-methyl-6-oxocyclohexa-1,3-dien-1-olate<br><br>C -0.42402 -1.23298 -0.49283<br>C -1.73189 -1.26050 0.27239<br>C -2.35940 -0.03145 0.45091<br>C -1.85550 1.23352 -0.02383<br>C -0.70548 1.32332 -0.73303<br>C 0.03263 0.08881 -1.12094<br>H -3.28997 -0.02714 1.01802<br>H -0.34219 2.27316 -1.11553<br>H -0.12461 -0.08616 -2.19981<br>O 0.22592 -2.25467 -0.67887<br>O -2.13342 -2.38407 0.71854<br>C -2.66545 2.46171 0.29229<br>H -3.67070 2.38820 -0.13993<br>H -2.79521 2.57671 1.37515<br>H -2.19178 3.36804 -0.09503<br>S 1.85183 0.28740 -1.06422<br>C 2.14745 0.24738 0.68414	C_1	-971.59506 (2.1)	-971.41931 (1.9)	-971.47439 (2.8)	-972.31610 (2.0)	-972.19544 (2.7)

	C 3.58020 0.50395 1.06388<br>H 3.89626 -0.25491 1.78451<br>H 4.25340 0.50199 0.20466<br>H 3.63519 1.48117 1.55490<br>O 1.26863 0.03599 1.49566						
5-adduct, conf2	C 0.45331 1.26540 -0.38765<br>C 1.84496 1.21592 0.21095<br>C 2.39358 -0.05681 0.35660<br>C 1.78463 -1.28151 -0.09680<br>C 0.59069 -1.28913 -0.73849<br>C -0.08800 -0.00077 -1.05886<br>H 3.35812 -0.12446 0.85897<br>H 0.15208 -2.20440 -1.12503<br>H 0.10180 0.21785 -2.12668<br>O -0.20335 2.29926 -0.38503<br>O 2.36246 2.30913 0.60273<br>C 2.53334 -2.56173 0.15514<br>H 3.51458 -2.54128 -0.33360<br>H 2.71647 -2.70197 1.22710<br>H 1.98304 -3.43041 -0.21627<br>S -1.91874 -0.10566 -1.07373<br>C -2.42755 -0.32305 0.61970<br>C -1.41055 -0.26685 1.71791<br>H -0.51266 -0.83761 1.46092<br>H -1.11756 0.77627 1.87904<br>H -1.86174 -0.65511 2.63276<br>O -3.61920 -0.48552 0.81372	C_1	-971.59243 (3.8)	-971.41683 (3.4)	-971.47126 (4.8)	-972.31245 (4.2)	-972.19128 (5.3)
5-adduct, conf3	C 0.55760 1.16783 -0.02651<br>C 2.06495 1.29904 0.00516<br>C 2.77327 0.10039 0.01197<br>C 2.18452 -1.21446 -0.07180<br>C 0.84521 -1.39124 -0.16715<br>C -0.06425 -0.21480 -0.23221<br>H 3.85849 0.16559 0.08811<br>H 0.40492 -2.37951 -0.26595<br>H -0.52907 -0.14828 -1.23388<br>O -0.16851 2.15057 0.07640<br>O 2.56012 2.46978 0.07987<br>C 3.12215 -2.39085 -0.06971<br>H 3.81599 -2.33900 -0.91729<br>H 3.73492 -2.39925 0.83972<br>H 2.58033 -3.33886 -0.12736<br>S -1.51726 -0.44347 0.85175	C_1	-971.59729 (0.7)	-971.42149 (0.5)	-971.47714 (1.1)	-972.31380 (3.4)	-972.19365 (3.8)

	C -2.85556 -0.19130 -0.28784<br>C -4.21381 -0.30634 0.34515<br>H -4.81635 -1.00786 -0.23843<br>H -4.16928 -0.63440 1.38524<br>H -4.69863 0.67400 0.29926<br>O -2.69030 0.04884 -1.46873						
5-adduct, conf4	C -0.33563 1.11840 0.43031<br>C -1.59918 1.41755 -0.35349<br>C -2.51427 0.37019 -0.40827<br>C -2.29627 -0.94024 0.15788<br>C -1.11148 -1.29553 0.71563<br>C 0.02216 -0.33539 0.72303<br>H -3.47291 0.56480 -0.88785<br>H -0.93854 -2.29194 1.11189<br>H 0.60440 -0.37755 1.64758<br>O 0.40623 2.01428 0.81698<br>O -1.74841 2.58908 -0.82394<br>C -3.44406 -1.91004 0.10640<br>H -4.31718 -1.50883 0.63455<br>H -3.75590 -2.08624 -0.93006<br>H -3.18155 -2.87193 0.55490<br>S 1.16113 -0.87960 -0.65554<br>C 2.83208 -0.38649 -0.25826<br>C 3.13955 0.26288 1.05532<br>H 2.49585 1.13184 1.21698<br>H 2.95625 -0.44498 1.87152<br>H 4.19192 0.55175 1.06206<br>O 3.67746 -0.65352 -1.09356	C_1	-971.59282 (3.5)	-971.41685 (3.4)	-971.47181 (4.4)	-972.31096 (5.2)	-972.18995 (6.1)
5-adduct, conf5	C -0.56352 1.17434 0.10861<br>C -2.06348 1.29331 -0.04827<br>C -2.76349 0.09028 -0.07274<br>C -2.17371 -1.21872 0.07685<br>C -0.83947 -1.38672 0.23738<br>C 0.06604 -0.20825 0.29779<br>H -3.84285 0.14595 -0.21250<br>H -0.39999 -2.37116 0.37254<br>H 0.57034 -0.15322 1.27895<br>O 0.15695 2.16660 0.10559<br>O -2.55791 2.45936 -0.18116<br>C -3.10586 -2.39931 0.06377<br>H -3.83829 -2.32793 0.87678<br>H -3.67598 -2.43518 -0.87234<br>H -2.56371 -3.34271 0.17205	C_1	-971.59723 (0.7)	-971.42141 (0.5)	-971.47745 (0.9)	-972.31388 (3.3)	-972.19410 (3.5)

	S 1.48075 -0.41510 -0.84696<br>C 2.86328 -0.20019 0.24718<br>C 4.19424 -0.30908 -0.44276<br>H 4.80594 -1.04506 0.08648<br>H 4.10430 -0.59187 -1.49315<br>H 4.69778 0.66032 -0.37393<br>O 2.74675 0.01011 1.43926						
5-adduct, conf6	C 0.90950 -1.22554 0.48998<br>C 2.24369 -0.93920 -0.16126<br>C 2.53560 0.41195 -0.33248<br>C 1.65945 1.50088 0.02825<br>C 0.40196 1.28671 0.48923<br>C -0.12120 -0.09555 0.60203<br>H 3.51584 0.66202 -0.73717<br>H -0.25771 2.11448 0.73405<br>H -0.67216 -0.27585 1.52967<br>O 0.61857 -2.33824 0.91119<br>O 2.99775 -1.92313 -0.44596<br>C 2.19635 2.89601 -0.13393<br>H 3.10457 3.03739 0.46376<br>H 2.47305 3.08533 -1.17811<br>H 1.46377 3.64899 0.16890<br>S -1.32604 -0.48236 -0.77591<br>C -2.92072 0.08981 -0.22938<br>C -3.10686 0.63370 1.15644<br>H -2.91658 -0.14619 1.90087<br>H -2.42550 1.46432 1.35679<br>H -4.13814 0.97612 1.25347<br>O -3.83096 -0.01381 -1.03160	C_1	-971.59065 (4.9)	-971.41462 (4.8)	-971.47007 (5.5)	-972.30978 (5.9)	-972.18919 (6.6)
5-adduct, conf7	C -0.39738 1.11564 0.12470<br>C -1.85611 1.40735 -0.15162<br>C -2.70155 0.30165 -0.14688<br>C -2.29523 -1.05202 0.14381<br>C -1.00730 -1.37210 0.41487<br>C 0.04479 -0.31825 0.43643<br>H -3.75295 0.47844 -0.37133<br>H -0.71003 -2.38808 0.65893<br>H 0.50692 -0.27587 1.43185<br>O 0.44411 2.00766 0.10740<br>O -2.18619 2.61145 -0.39581<br>C -3.36964 -2.10441 0.15309<br>H -4.14034 -1.86829 0.89641<br>H -3.87359 -2.15311 -0.81962	C_1	-971.59251 (3.7)	-971.41664 (3.5)	-971.47124 (4.8)	-972.31100 (5.2)	-972.18973 (6.2)

	H -2.96410 -3.09404 0.38003<br>S 1.37881 -0.83540 -0.72809<br>C 2.94497 -0.29032 -0.07107<br>C 3.02358 0.35614 1.27835<br>H 2.38400 1.24203 1.31008<br>H 2.68622 -0.33873 2.05485<br>H 4.06260 0.62917 1.46986<br>O 3.92001 -0.51002 -0.76805						
5-adduct, conf8	C 0.87999 -1.25975 0.13327<br>C 2.36306 -0.95304 0.15293<br>C 2.70222 0.38811 0.00069<br>C 1.75855 1.47557 -0.09197<br>C 0.42241 1.27245 -0.00593<br>C -0.11644 -0.09555 0.23053<br>H 3.76326 0.62881 -0.05861<br>H -0.27918 2.10077 -0.05011<br>H -0.46796 -0.18300 1.27089<br>O 0.47142 -2.41336 0.09049<br>O 3.16859 -1.93369 0.23962<br>C 2.31866 2.85961 -0.27469<br>H 2.98306 3.12415 0.55649<br>H 2.91927 2.91833 -1.19028<br>H 1.52690 3.61132 -0.33437<br>S -1.57399 -0.48330 -0.82367<br>C -3.00109 0.14189 0.03054<br>C -2.87580 0.72733 1.40776<br>H -2.54601 -0.03644 2.11988<br>H -2.15274 1.54684 1.43467<br>H -3.85669 1.09602 1.71144<br>O -4.06693 0.04534 -0.55261	C_1	-971.59006 (5.3)	-971.41429 (5.0)	-971.46950 (5.9)	-972.30920 (6.3)	-972.18864 (6.9)

Table S7.5. One-electron oxidized neutral form in vacuo.

Structure	Schematic drawing	Symmetry	*E*	H_{RRHO}	G_{RRHO}	$E_{M06\text{-}2X,large}$	$G_{RRHO,M06\text{-}2X,large}$
6-adduct, conf1	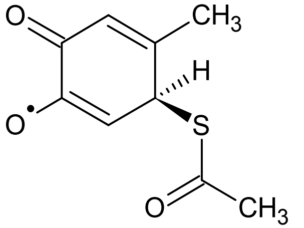<br>[(3*S*)-3-(acetylsulfanyl)-4-methyl-6-oxocyclohexa-1,4-dien-1-yl]oxidanyl<br><br>C 2.16274 -1.12444 0.20978<br>C 2.76128 0.22985 -0.17948<br>C 1.86886 1.39373 -0.12102<br>C 0.55016 1.32886 0.15084<br>C -0.12317 0.01182 0.42041<br>C 0.77157 -1.16214 0.51346<br>H 2.34655 2.35291 -0.30654<br>H -0.74770 0.09006 1.32854<br>H 0.34599 -2.11996 0.80144<br>O 2.87286 -2.13545 0.26933<br>O 3.93058 0.32133 -0.49851<br>C -0.30724 2.55175 0.22718<br>H -0.80372 2.61428 1.20290<br>H 0.28297 3.45759 0.07595<br>H -1.09827 2.52253 -0.53065<br>S -1.40684 -0.35445 -0.86266<br>C -2.88070 -0.30777 0.15743<br>C -4.13959 -0.61650 -0.60410<br>H -4.86163 0.18438 -0.42256<br>H -3.96990 -0.72830 -1.67677<br>H -4.56178 -1.54431 -0.20561<br>O -2.85427 -0.05951 1.34027	C_1	-971.41230 (4.7)	-971.23570 (5.0)	-971.29330 (5.1)	-972.11756 (4.9)	-971.99856 (4.7)
6-adduct, conf2	C -1.88440 -1.26256 -0.18924<br>C -2.68141 -0.02092 0.22004<br>C -2.01511 1.27919 0.06323<br>C -0.72720 1.43347 -0.30131<br>C 0.16088 0.23955 -0.53149	C_1	-971.40058 (12.1)	-971.22407 (12.3)	-971.28075 (13.0)	-972.10782 (11.0)	-971.98799 (11.3)

	C -0.53145 -1.06570 -0.59353<br>H -2.64393 2.14687 0.24837<br>H 0.76529 0.40898 -1.42993<br>H 0.02459 -1.94796 -0.90099<br>O -2.41297 -2.37928 -0.19511<br>O -3.82265 -0.12176 0.62333<br>C -0.10619 2.78017 -0.49613<br>H 0.21913 2.90414 -1.53698<br>H -0.81242 3.57894 -0.26202<br>H 0.78069 2.90059 0.13549<br>S 1.37034 0.16091 0.89240<br>C 2.94248 -0.40007 0.21714<br>C 3.08750 -0.68436 -1.25333<br>H 2.33223 -1.39007 -1.61041<br>H 2.99736 0.23957 -1.83461<br>H 4.08155 -1.10501 -1.41275<br>O 3.84306 -0.51186 1.01093						
6-adduct, conf3	C 1.78908 -0.88628 0.65415<br>C 2.24817 0.03628 -0.47765<br>C 1.46680 1.25938 -0.70086<br>C 0.34008 1.57824 -0.03103<br>C -0.20422 0.67418 1.04175<br>C 0.61085 -0.51626 1.36421<br>H 1.86451 1.92934 -1.45981<br>H -0.31264 1.27372 1.96120<br>H 0.29364 -1.15603 2.18407<br>O 2.42747 -1.90834 0.93389<br>O 3.22484 -0.24232 -1.14516<br>C -0.39383 2.85713 -0.28229<br>H -0.49176 3.43511 0.64498<br>H 0.12786 3.47137 -1.01859<br>H -1.41148 2.66574 -0.63892<br>S -2.00114 0.22076 0.83116<br>C -2.20589 -0.89845 -0.57559<br>C -1.02835 -1.38647 -1.36353<br>H -0.37604 -0.56349 -1.66587<br>H -0.44017 -2.08724 -0.76202<br>H -1.40619 -1.90659 -2.24547<br>O -3.34801 -1.21192 -0.79788	C_1	-971.40148 (11.5)	-971.22513 (11.6)	-971.28111 (12.8)	-972.11050 (9.3)	-971.99013 (10.0)
6-adduct, conf4	C -1.75324 -0.87754 -0.65455<br>C -2.14624 -0.03532 0.56130<br>C -1.40478 1.21463 0.77693<br>C -0.33480 1.59841 0.05611	C_1	-971.40784 (7.5)	-971.23134 (7.7)	-971.28804 (8.4)	-972.11644 (5.6)	-971.99664 (5.9)

	C 0.19157 0.74155 -1.06484<br>C -0.62115 -0.44707 -1.40888<br>H -1.78487 1.84462 1.57784<br>H 0.29671 1.36883 -1.96443<br>H -0.33435 -1.04307 -2.27158<br>O -2.40800 -1.87366 -0.97320<br>O -3.06066 -0.37819 1.28371<br>C 0.35690 2.90416 0.29390<br>H 0.33420 3.52525 -0.61060<br>H -0.12129 3.45968 1.10305<br>H 1.41183 2.75466 0.54834<br>S 1.96907 0.25512 -0.81889<br>C 1.83411 -0.91492 0.53576<br>C 3.16966 -1.41921 1.01167<br>H 3.08881 -2.49208 1.20237<br>H 3.97387 -1.22183 0.29954<br>H 3.40570 -0.92318 1.95902<br>O 0.77766 -1.24103 1.01628						
6-adduct, conf5	C 1.74369 -0.88662 0.65756<br>C 2.14799 -0.04624 -0.55590<br>C 1.41659 1.20920 -0.77353<br>C 0.34635 1.60008 -0.05697<br>C -0.19056 0.74648 1.06160<br>C 0.61233 -0.44800 1.40828<br>H 1.80430 1.83690 -1.57256<br>H -0.29477 1.37413 1.96104<br>H 0.31825 -1.04203 2.26990<br>O 2.38936 -1.88820 0.97772<br>O 3.06295 -0.39530 -1.27461<br>C -0.33531 2.91060 -0.29708<br>H -0.31219 3.53130 0.60766<br>H 0.14998 3.46305 -1.10411<br>H -1.39011 2.76831 -0.55616<br>S -1.97046 0.27315 0.80770<br>C -1.83881 -0.90277 -0.54104<br>C -3.17298 -1.43095 -0.99537<br>H -3.14531 -2.52326 -0.94754<br>H -4.00571 -1.05592 -0.39685<br>H -3.31799 -1.14512 -2.04160<br>O -0.78417 -1.22756 -1.02666	C_1	-971.40782 (7.6)	saddle point			

5-adduct, conf1	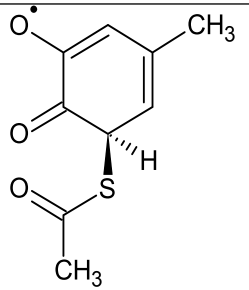<br>[(5*R*)-5-(acetylsulfanyl)-3-methyl-6-oxocyclohexa-1,3-dien-1-yl]oxidanyl<br><br>C -0.28743 -1.26911 -0.59356<br>C -1.53878 -1.32022 0.29928<br>C -2.25090 -0.09055 0.52768<br>C -1.87398 1.14642 -0.02487<br>C -0.77180 1.23903 -0.84727<br>C 0.07681 0.07719 -1.23313<br>H -3.11975 -0.15963 1.17782<br>H -0.49654 2.20165 -1.27121<br>H -0.00434 -0.08677 -2.31897<br>O 0.34558 -2.26662 -0.83135<br>O -1.88451 -2.39242 0.78175<br>C -2.69311 2.36727 0.29457<br>H -3.74067 2.21872 0.01296<br>H -2.67039 2.57415 1.36982<br>H -2.32105 3.25093 -0.22926<br>S 1.86012 0.45169 -1.04007<br>C 1.96595 0.31513 0.73443<br>C 3.34278 0.55237 1.28970<br>H 3.77353 -0.41963 1.55234<br>H 4.00455 1.05166 0.57896<br>H 3.25632 1.14282 2.20492<br>O 1.00632 0.02077 1.40994	C_1	-971.41866 (0.8)	-971.24227 (0.8)	-971.29934 (1.3)	-972.12531 (0.0)	-972.00599 (0.0)
5-adduct, conf2	C 0.44071 1.26452 -0.30213<br>C 1.91749 1.22081 0.13485<br>C 2.50935 -0.07984 0.30453<br>C 1.85714 -1.27959 -0.03435<br>C 0.61393 -1.25021 -0.63563<br>C -0.08124 0.01564 -1.01707<br>H 3.50453 -0.09861 0.74272<br>H 0.12068 -2.17851 -0.91171<br>H 0.17365 0.21315 -2.07595	C_1	-971.40937 (6.6)	-971.23334 (6.4)	-971.29002 (7.2)	-972.11577 (6.0)	-971.99641 (6.0)

	O -0.23071 2.24298 -0.10021<br>O 2.50315 2.26974 0.37914<br>C 2.54228 -2.59001 0.24193<br>H 3.48629 -2.65491 -0.30919<br>H 2.77973 -2.68824 1.30621<br>H 1.92005 -3.43917 -0.04944<br>S -1.89810 -0.12851 -1.12823<br>C -2.52440 -0.29629 0.55202<br>C -1.60048 -0.16451 1.72896<br>H -0.67449 -0.73128 1.59681<br>H -1.34285 0.89080 1.86430<br>H -2.13067 -0.52144 2.61358<br>O -3.71194 -0.48507 0.65053						
5-adduct, conf3	C 0.52095 1.19541 0.08122<br>C 2.04879 1.30946 -0.06645<br>C 2.80352 0.08508 -0.07561<br>C 2.21772 -1.19378 -0.05450<br>C 0.84469 -1.33330 -0.06881<br>C -0.09608 -0.18428 -0.14493<br>H 3.88567 0.19298 -0.08511<br>H 0.40371 -2.32660 -0.06690<br>H -0.53500 -0.13649 -1.16498<br>O -0.14697 2.16537 0.33559<br>O 2.56638 2.42018 -0.12145<br>C 3.10944 -2.40525 -0.03925<br>H 3.74619 -2.42881 -0.92987<br>H 3.77051 -2.38884 0.83349<br>H 2.53035 -3.33108 -0.01087<br>S -1.57041 -0.44712 0.88745<br>C -2.83438 -0.22423 -0.36102<br>C -4.22985 -0.24899 0.19415<br>H -4.89387 -0.72421 -0.53095<br>H -4.28642 -0.76103 1.15736<br>H -4.55574 0.78757 0.33230<br>O -2.57536 -0.05184 -1.52945	C_1	-971.41987 (0.0)	-971.24361 (0.0)	-971.30149 (0.0)	-972.12248 (1.8)	-972.00410 (1.2)
5-adduct, conf4	C -0.27214 1.14855 0.50113<br>C -1.46304 1.41770 -0.43034<br>C -2.46178 0.37969 -0.49469<br>C -2.33360 -0.86030 0.16203<br>C -1.15869 -1.18582 0.81241<br>C 0.04091 -0.30672 0.78753<br>H -3.36278 0.61456 -1.05627<br>H -1.05529 -2.15081 1.30114	C_1	-971.41377 (3.8)	-971.23726 (4.0)	-971.29401 (4.7)	-972.11939 (3.7)	-971.99964 (4.0)

	H 0.61850 -0.38438 1.71247<br>O 0.37256 2.05427 0.97694<br>O -1.55881 2.49145 -1.01156<br>C -3.48408 -1.82738 0.12842<br>H -4.38710 -1.37349 0.54914<br>H -3.71334 -2.11417 -0.90342<br>H -3.26293 -2.73677 0.69166<br>S 1.08636 -0.94279 -0.61278<br>C 2.79712 -0.44438 -0.28511<br>C 3.14220 0.28435 0.97833<br>H 2.52580 1.17914 1.10495<br>H 2.97430 -0.36587 1.84439<br>H 4.19937 0.55099 0.93321<br>O 3.59129 -0.76680 -1.13176						
5-adduct, conf5	C 0.86655 -1.30256 -0.02882<br>C 2.33279 -0.95483 0.28880<br>C 2.71780 0.42392 0.14217<br>C 1.80158 1.45354 -0.14182<br>C 0.44695 1.18879 -0.18304<br>C -0.13676 -0.15362 0.12004<br>H 3.78103 0.63724 0.22277<br>H -0.24916 1.98972 -0.41814<br>H -0.39926 -0.19111 1.19028<br>O 0.54966 -2.41994 -0.34143<br>O 3.11187 -1.85192 0.58844<br>C 2.31652 2.84598 -0.38284<br>H 2.87185 3.20918 0.48826<br>H 3.00220 2.86244 -1.23622<br>H 1.50381 3.54715 -0.58529<br>S -1.65927 -0.50654 -0.82966<br>C -2.99902 0.18152 0.14316<br>C -2.74647 0.72218 1.52715<br>H -2.39602 -0.07191 2.19531<br>H -2.00084 1.52277 1.52395<br>H -3.69270 1.11112 1.90633<br>O -4.09555 0.15418 -0.36220	C_1	-971.40655 (8.4)	-971.23047 (8.2)	-971.28827 (8.3)	-972.11190 (8.4)	-971.99362 (7.8)

Table S7.6. One-electron oxidized neutral form in water.

Structure	Schematic drawing	Symmetry	G_{PCM}	$H_{PCM,RRHO}$	$G_{PCM,RRHO}$	$G_{SMD,M06-2X,large}$	$G_{SMD,RRHO,M06-2X,large}$
6-adduct, conf1	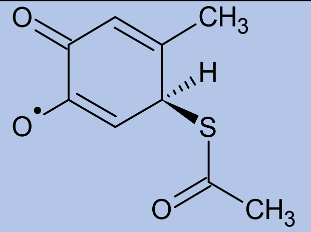 [(3*S*)-3-(acetylsulfanyl)-4-methyl-6-oxocyclohexa-1,4-dien-1-yl]oxidanyl<br><br>C 2.10685 -1.14515 0.20899<br>C 2.74119 0.18701 -0.19015<br>C 1.90228 1.37613 -0.12368<br>C 0.58341 1.35377 0.17032<br>C -0.12939 0.05893 0.43781<br>C 0.72311 -1.14279 0.53022<br>H 2.40336 2.32166 -0.31524<br>H -0.75691 0.14812 1.34206<br>H 0.26627 -2.08085 0.83385<br>O 2.79998 -2.17430 0.26203<br>O 3.91808 0.22328 -0.52390<br>C -0.22476 2.60419 0.27100<br>H -0.64321 2.70088 1.27997<br>H 0.38055 3.48625 0.05629<br>H -1.07205 2.58072 -0.42285<br>S -1.39807 -0.27041 -0.86564<br>C -2.87870 -0.32745 0.14662<br>C -4.12039 -0.61733 -0.64176<br>H -4.85929 0.15892 -0.42463<br>H -3.93875 -0.66370 -1.71671<br>H -4.52548 -1.57551 -0.30179<br>O -2.85644 -0.15321 1.34477	C_1	-971.43129 (4.3)	-971.25485 (4.3)	-971.31256 (4.7)	-972.13892 (5.5)	-972.02020 (4.8)
6-adduct, conf2	C -1.96153 -1.20451 -0.23731<br>C -2.68447 0.06578 0.21343<br>C -1.95875 1.32592 0.10746<br>C -0.66085 1.41821 -0.25546<br>C 0.15411 0.18454 -0.52995<br>C -0.60388 -1.07834 -0.63880	C_1	-971.42396 (8.9)	-971.24734 (9.0)	-971.30375 (10.2)	-972.13506 (7.9)	-972.01484 (8.2)

	H -2.53219 2.22363 0.32470<br>H 0.77376 0.34535 -1.41792<br>H -0.09166 -1.96804 -0.99465<br>O -2.56833 -2.28645 -0.27109<br>O -3.83829 -0.00215 0.61320<br>C 0.03298 2.72969 -0.41126<br>H 0.32497 2.87324 -1.45892<br>H -0.61095 3.55817 -0.11239<br>H 0.95233 2.76162 0.18289<br>S 1.33711 -0.04760 0.89578<br>C 2.95870 -0.40931 0.20710<br>C 3.17461 -0.45787 -1.27319<br>H 2.52541 -1.20043 -1.74607<br>H 2.97004 0.51677 -1.72698<br>H 4.21697 -0.72363 -1.45419<br>O 3.82811 -0.59512 1.03026						
6-adduct, conf3	C 1.74214 -0.85687 0.66172<br>C 2.12769 -0.02373 -0.55982<br>C 1.40086 1.21859 -0.78598<br>C 0.32840 1.60503 -0.06266<br>C -0.19486 0.75312 1.06215<br>C 0.61523 -0.43067 1.41615<br>H 1.77363 1.84705 -1.59108<br>H -0.30132 1.38218 1.95954<br>H 0.32899 -1.01350 2.28751<br>O 2.40860 -1.85638 0.96845<br>O 3.03921 -0.39867 -1.28513<br>C -0.36129 2.90633 -0.30910<br>H -0.33786 3.52576 0.59551<br>H 0.11495 3.45659 -1.12215<br>H -1.41630 2.75202 -0.55903<br>S -1.96257 0.25443 0.80859<br>C -1.82104 -0.93385 -0.52017<br>C -3.15195 -1.41417 -1.02012<br>H -3.08847 -2.48624 -1.22016<br>H -3.96375 -1.20940 -0.31948<br>H -3.36640 -0.90053 -1.96347<br>O -0.75449 -1.29088 -0.97052	C_1	-971.42925 (5.5)	-971.25278 (5.6)	-971.30942 (6.6)	-972.14109 (4.1)	-972.02125 (4.2)
6-adduct, conf4	C -1.82197 -0.92853 -0.58947<br>C -2.29611 0.08050 0.45541<br>C -1.49431 1.28339 0.64026<br>C -0.34152 1.53758 -0.01832<br>C 0.18903 0.57577 -1.04633	C_1	-971.42210 (10.0)	-971.24589 (9.9)	-971.30224 (11.2)	-972.13588 (7.4)	-972.01602 (7.5)

	C -0.61300 -0.64273 -1.28029<br>H -1.88425 2.00548 1.35328<br>H 0.24338 1.12128 -2.00419<br>H -0.27924 -1.34473 -2.03976<br>O -2.48864 -1.95050 -0.81798<br>O -3.32002 -0.13641 1.08816<br>C 0.42253 2.80171 0.19749<br>H 0.64661 3.28558 -0.76001<br>H -0.13938 3.49641 0.82360<br>H 1.38523 2.59760 0.67889<br>S 1.99442 0.16942 -0.88226<br>C 2.26419 -0.82595 0.58943<br>C 1.13354 -1.22599 1.48222<br>H 0.51914 -0.36646 1.76105<br>H 0.49656 -1.95528 0.97111<br>H 1.55045 -1.68808 2.37846<br>O 3.42070 -1.13770 0.77621						
6-adduct, conf5	C 2.02657 -1.16646 0.26086<br>C 2.69728 0.12428 -0.21106<br>C 1.91782 1.35361 -0.13267<br>C 0.61614 1.39873 0.22598<br>C -0.14747 0.13803 0.52489<br>C 0.66319 -1.09043 0.65526<br>H 2.45280 2.27035 -0.36768<br>H -0.76915 0.28382 1.41418<br>H 0.18659 -1.99469 1.02353<br>O 2.67732 -2.22177 0.31512<br>O 3.85507 0.09789 -0.60447<br>C -0.13029 2.68438 0.34916<br>H -0.44697 2.83385 1.38872<br>H 0.48642 3.53228 0.04729<br>H -1.03910 2.67174 -0.26197<br>S -1.32787 -0.18049 -0.88565<br>C -2.97355 -0.41482 -0.19814<br>C -3.22548 -0.30671 1.27330<br>H -2.62697 -1.02983 1.83450<br>H -2.98529 0.69692 1.63762<br>H -4.28382 -0.50505 1.44817<br>O -3.83162 -0.65051 -1.02059	C_1	-971.42392 (8.9)	-971.24736 (9.0)	-971.30370 (10.2)	-972.13473 (8.1)	-972.01451 (8.4)
6-adduct, conf6	C 2.09616 -1.15132 0.21245<br>C 2.73940 0.17486 -0.19249<br>C 1.90985 1.37061 -0.12661<br>C 0.59160 1.35902 0.17082	C_1	-971.43129 (4.3)	-971.25485 (4.3)	-971.31223 (4.9)	-972.13893 (5.5)	-972.01987 (5.0)

	C -0.13057 0.07002 0.44142<br>C 0.71335 -1.13744 0.53740<br>H 2.41777 2.31181 -0.32148<br>H -0.75929 0.16580 1.34390<br>H 0.25025 -2.07091 0.84563<br>O 2.78181 -2.18538 0.26714<br>O 3.91550 0.20121 -0.52991<br>C -0.20670 2.61572 0.27169<br>H -0.61728 2.71940 1.28324<br>H 0.40353 3.49248 0.04939<br>H -1.05908 2.59559 -0.41599<br>S -1.39752 -0.25288 -0.86562<br>C -2.87973 -0.32152 0.14484<br>C -4.11392 -0.63722 -0.64508<br>H -4.92261 0.01795 -0.31251<br>H -3.96418 -0.53213 -1.72101<br>H -4.40010 -1.67158 -0.42699<br>O -2.86046 -0.14566 1.34266						
5-adduct, conf1	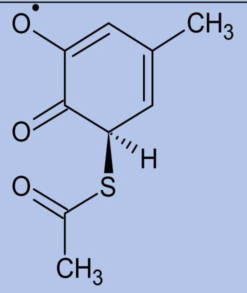<br>[(5*R*)-5-(acetylsulfanyl)-3-methyl-6-oxocyclohexa-1,3-dien-1-yl]oxidanyl<br><br>C -0.30453 -1.25770 -0.60012<br>C -1.54408 -1.29377 0.30393<br>C -2.24732 -0.06814 0.53024<br>C -1.86080 1.16651 -0.02001<br>C -0.75401 1.24750 -0.84075<br>C 0.07611 0.07614 -1.23304<br>H -3.11991 -0.12486 1.17646<br>H -0.47143 2.20449 -1.27055<br>H -0.01465 -0.07355 -2.32016<br>O 0.30262 -2.27646 -0.84563<br>O -1.88016 -2.37147 0.80271<br>C -2.67348 2.39164 0.29038<br>H -3.71038 2.25764 -0.03398	C_1	-971.43809 (0.0)	-971.26167 (0.0)	-971.31831 (1.1)	-972.14770 (0.0)	-972.02792 (0.0)

	H -2.69151 2.57683 1.36929<br>H -2.26909 3.27673 -0.20470<br>S 1.86336 0.42796 -1.03979<br>C 1.97858 0.26287 0.72867<br>C 3.31677 0.62573 1.29858<br>H 3.57711 -0.09301 2.07891<br>H 4.10121 0.65884 0.54032<br>H 3.23280 1.61621 1.75869<br>O 1.03113 -0.10849 1.39232						
5-adduct, conf2	C 0.45047 1.28021 -0.29393<br>C 1.90931 1.21032 0.18072<br>C 2.49668 -0.08788 0.31005<br>C 1.85007 -1.27670 -0.07335<br>C 0.60551 -1.22973 -0.67212<br>C -0.08341 0.04833 -1.01368<br>H 3.48899 -0.12814 0.75254<br>H 0.11769 -2.14812 -0.98589<br>H 0.16173 0.25770 -2.07279<br>O -0.20084 2.28397 -0.12155<br>O 2.48397 2.25907 0.48429<br>C 2.53853 -2.59162 0.15616<br>H 3.48434 -2.62777 -0.39392<br>H 2.77330 -2.72200 1.21723<br>H 1.91960 -3.43045 -0.16775<br>S -1.90356 -0.06251 -1.10919<br>C -2.51535 -0.32185 0.55546<br>C -1.57786 -0.34891 1.72226<br>H -0.73729 -1.02721 1.55259<br>H -1.17826 0.65498 1.89770<br>H -2.13492 -0.66888 2.60410<br>O -3.71838 -0.44967 0.65147	C_1	-971.42987 (5.2)	-971.25386 (4.9)	-971.31081 (5.8)	-972.14016 (4.7)	-972.02109 (4.3)
5-adduct, conf3	C 0.54682 1.20423 -0.01129<br>C 2.07883 1.27653 -0.01188<br>C 2.80191 0.04314 -0.00558<br>C 2.19119 -1.22249 -0.06488<br>C 0.81657 -1.32856 -0.14788<br>C -0.09699 -0.15890 -0.20657<br>H 3.88510 0.11914 0.05236<br>H 0.35446 -2.30964 -0.21117<br>H -0.57118 -0.11749 -1.20924<br>O -0.11043 2.21347 0.11692<br>O 2.62053 2.38616 0.01783<br>C 3.05472 -2.45146 -0.05590	C_1	-971.43796 (0.1)	-971.26170 (0.0)	-971.32001 (0.0)	-972.14295 (3.0)	-972.02500 (1.8)

	H 3.73051 -2.45188 -0.91722<br>H 3.67592 -2.47725 0.84511<br>H 2.45465 -3.36289 -0.08833<br>S -1.53785 -0.38977 0.88625<br>C -2.85585 -0.20591 -0.30838<br>C -4.22480 -0.31187 0.29365<br>H -4.82129 -1.00213 -0.30843<br>H -4.20428 -0.64615 1.33231<br>H -4.69623 0.67504 0.24525<br>O -2.64466 -0.00696 -1.48580						
5-adduct, conf4	C -0.29880 1.14841 0.48128<br>C -1.53172 1.40360 -0.39358<br>C -2.49894 0.34806 -0.45709<br>C -2.31932 -0.90558 0.16122<br>C -1.11762 -1.21535 0.77092<br>C 0.05166 -0.29736 0.75460<br>H -3.42471 0.56598 -0.98382<br>H -0.97319 -2.18584 1.23637<br>H 0.63483 -0.37825 1.67495<br>O 0.35457 2.07165 0.91814<br>O -1.66907 2.50106 -0.93861<br>C -3.44484 -1.89845 0.13772<br>H -4.32897 -1.48839 0.63620<br>H -3.73012 -2.12699 -0.89426<br>H -3.16803 -2.83046 0.63399<br>S 1.11723 -0.87025 -0.65816<br>C 2.81890 -0.42182 -0.26955<br>C 3.15598 0.22930 1.03285<br>H 2.54504 1.12093 1.19784<br>H 2.96879 -0.46615 1.85835<br>H 4.21482 0.49182 1.02072<br>O 3.62624 -0.71316 -1.12455	C_1	-971.43197 (3.8)	-971.25540 (4.0)	-971.31167 (5.2)	-972.13996 (4.9)	-972.01965 (5.2)

Part 8: 4-Methylcatechol – azide adducts

Table S8.1. Reduced anionic form in water.

Structura	Schematic drawing	Symmetry	G_{PCM}	$H_{PCM,RRHO}$	$G_{PCM,RRHO}$	$G_{SMD,M06\text{-}2X,large}$	$G_{SMD,RRHO,M06\text{-}2X,large}$
6-adduct, conf1	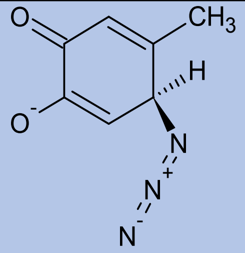<br>(3*S*)-3-azido-4-methyl-6-oxocyclohexa-1,4-dien-1-olate<br><br>C -1.57385 0.64735 0.45558<br>C -1.62004 -0.62520 -0.37551<br>C -0.44938 -1.51220 -0.40013<br>C 0.70131 -1.23961 0.23968<br>C 0.83595 0.04736 1.01351<br>C -0.37750 0.89438 1.09955<br>H -0.56456 -2.43188 -0.97179<br>H 1.21286 -0.20004 2.01433<br>H -0.29592 1.78481 1.72123<br>O -2.63057 1.36086 0.50752<br>O -2.62751 -0.92909 -1.01779<br>C 1.88053 -2.15748 0.21395<br>H 2.14030 -2.47810 1.23011<br>H 1.68014 -3.04494 -0.39054<br>H 2.76268 -1.64727 -0.18883<br>N 2.03437 0.80798 0.44061<br>N 1.81613 1.34436 -0.63713<br>N 1.68478 1.88132 -1.63379	C_1	-584.47984 (0.0)	-584.34141 (0.0)	-584.39084 (0.0)	-585.06601 (0.0)	-584.97700 (0.0)
6-adduct, conf2	C 1.34721 -1.19823 0.14105<br>C 2.06259 0.11069 -0.14280<br>C 1.30768 1.36882 -0.09122<br>C -0.01181 1.43609 0.15633<br>C -0.77336 0.17653 0.46117<br>C 0.00177 -1.08854 0.42876<br>H 1.88344 2.27327 -0.28102	C_1	-584.47857 (0.8)	-584.34026 (0.7)	-584.39024 (0.4)	-585.06392 (1.3)	-584.97559 (0.9)

	H -1.22842 0.32229 1.45708<br>H -0.54768 -1.99677 0.67509<br>O 2.03413 -2.27406 0.11039<br>O 3.26560 0.13338 -0.41545<br>C -0.77236 2.72176 0.17046<br>H -1.26525 2.87228 1.13837<br>H -0.11592 3.57300 -0.02357<br>H -1.56492 2.70597 -0.58620<br>N -1.93864 0.16057 -0.51033<br>N -2.79002 -0.68536 -0.28409<br>N -3.63267 -1.44484 -0.16012						
5-adduct, conf1	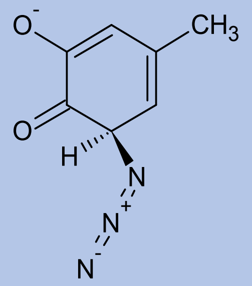<br>(5*R*)-5-azido-3-methyl-6-oxocyclohexa-1,3-dien-1-olate<br><br>C 1.31145 -0.37099 -0.51127<br>C 0.76224 -1.39500 0.46475<br>C -0.62534 -1.48054 0.49833<br>C -1.52095 -0.65357 -0.28248<br>C -1.07867 0.36812 -1.05689<br>C 0.36910 0.69950 -1.09174<br>H -1.06735 -2.24435 1.13684<br>H -1.76192 0.98886 -1.63019<br>H 0.72357 0.91310 -2.10412<br>O 2.49280 -0.34310 -0.82269<br>O 1.59166 -2.10432 1.11642<br>C -2.98575 -0.98423 -0.20749<br>H -3.58704 -0.30204 -0.81421<br>H -3.16981 -2.00888 -0.55185<br>H -3.34087 -0.93187 0.82851<br>N 0.70532 1.97189 -0.34543<br>N 0.07152 2.16267 0.68610<br>N -0.47180 2.44612 1.64587	C_1	-584.47573 (2.6)	-584.33748 (2.5)	-584.38790 (1.8)	-585.05952 (4.1)	-584.97170 (3.3)
5-adduct, conf2	C -0.31453 1.08496 -0.26244<br>C 1.12264 1.43541 0.06577	C_1	-584.47755 (1.4)	-584.33949 (1.2)	-584.38887 (1.2)	-585.06087 (3.2)	-584.97220 (3.0)

	C 1.96375 0.33655 0.22845<br>C 1.57911 -1.04014 0.01627<br>C 0.35549 -1.37405 -0.45973<br>C -0.58032 -0.28750 -0.88149<br>H 2.97485 0.53255 0.58411<br>H 0.07764 -2.39932 -0.68676<br>H -0.40111 -0.09649 -1.95540<br>O -1.23499 1.86877 -0.07304<br>O 1.41044 2.65555 0.27854<br>C 2.60475 -2.09663 0.32324<br>H 3.49567 -1.96679 -0.30277<br>H 2.93767 -2.02597 1.36551<br>H 2.21078 -3.10245 0.15434<br>N -2.00318 -0.66905 -0.83223<br>N -2.50217 -0.72560 0.28572<br>N -3.07815 -0.81787 1.26315						
5-adduct, conf3	C 0.35043 0.90746 -0.03956<br>C -1.04299 1.48470 0.07665<br>C -2.06734 0.54134 0.05379<br>C -1.87729 -0.89106 0.00555<br>C -0.64548 -1.44993 0.05583<br>C 0.54234 -0.57797 0.27534<br>H -3.08949 0.91821 0.03497<br>H -0.49295 -2.52515 0.06518<br>H 0.73087 -0.55172 1.36971<br>O 1.32429 1.59374 -0.32079<br>O -1.17078 2.75058 0.08221<br>C -3.11216 -1.74460 -0.08714<br>H -2.86448 -2.80817 -0.14093<br>H -3.76049 -1.58628 0.78300<br>H -3.70261 -1.48182 -0.97293<br>N 1.72334 -1.13717 -0.39559<br>N 2.81866 -0.72807 -0.02327<br>N 3.89599 -0.46896 0.23699	C_1	-584.47879 (0.7)	-584.34085 (0.4)	-584.38959 (0.8)	-585.06172 (2.7)	-584.97252 (2.8)
5-adduct, conf4	C 0.33764 -1.28065 -0.01565<br>C 1.79952 -0.89967 0.11333<br>C 2.05864 0.46766 0.05466<br>C 1.04910 1.49714 -0.02957<br>C -0.27247 1.20572 0.02108<br>C -0.70324 -0.20243 0.26766<br>H 3.10300 0.77756 0.03899<br>H -1.03310 1.98237 0.01501<br>H -0.85428 -0.32389 1.36089	C_1	-584.47629 (2.2)	-584.33837 (1.9)	-584.38797 (1.8)	-585.05966 (4.0)	-584.97134 (3.6)

	O -0.00925 -2.42186 -0.28449<br>O 2.65831 -1.83624 0.15994<br>C 1.52414 2.91840 -0.15774<br>H 0.68790 3.61912 -0.22895<br>H 2.13877 3.20248 0.70474<br>H 2.15311 3.03925 -1.04769<br>N -1.96072 -0.57727 -0.41205<br>N -2.96104 0.01322 -0.02790<br>N -3.95652 0.49704 0.24435						

Part 9: 4-Methylcatechol – methylamine adducts

Table S9.1. Reduced neutral form in vacuo.

Structure	Schematic drawing	Symmetry	E	H_{RRHO}	G_{RRHO}	$E_{M06\text{-}2X,large}$	$G_{RRHO,M06\text{-}2X,large}$
6-adduct, conf1	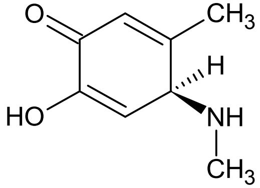<br>(4*S*)-2-hydroxy-5-methyl-4-(methylamino)cyclohexa-2,5-dien-1-one<br><br>C -1.54674 -0.82160 -0.09617<br>C -1.77251 0.62937 0.07443<br>C -0.60803 1.50406 0.04589<br>C 0.64370 1.03529 -0.13232<br>C 0.90998 -0.44438 -0.34378<br>C -0.31105 -1.31268 -0.27123<br>H -0.80379 2.56467 0.18344<br>H 1.29672 -0.52524 -1.37633<br>H -0.14407 -2.38147 -0.36484<br>O -2.66132 -1.57094 -0.03765<br>O -2.92640 1.02731 0.23938<br>C 1.81612 1.96656 -0.14260<br>H 2.41750 1.84130 -1.05010<br>H 1.48910 3.00748 -0.08546<br>H 2.47921 1.77227 0.70831<br>C 3.33087 -0.93126 0.08857<br>H 3.93961 -1.54291 0.76127<br>H 3.43026 -1.36014 -0.91498<br>H 3.76813 0.07957 0.06512<br>N 1.94790 -1.02033 0.50570<br>H 1.80708 -0.78174 1.47950<br>H -3.38741 -0.93452 0.10359	C_1	-516.08500 (3.0)	-515.89028 (2.9)	-515.93833 (3.1)	-516.58934 (3.4)	-516.44267 (3.3)
6-adduct, conf2	C -1.33810 -0.90564 -0.11952<br>C -1.78601 0.49016 0.09133<br>C -0.77636 1.53822 0.06616<br>C 0.53055 1.27161 -0.12915	C_1	-516.08974 (0.0)	-515.89486 (0.0)	-515.94327 (0.0)	-516.59361 (0.7)	-516.44715 (0.5)

	C 1.02079 -0.14050 -0.36301<br>C -0.04605 -1.19483 -0.33801<br>H -1.13343 2.55374 0.21650<br>H 1.45781 -0.12875 -1.37796<br>H 0.25610 -2.22699 -0.49457<br>O -2.32274 -1.81783 -0.06651<br>O -2.98643 0.69199 0.27989<br>C 1.56569 2.34779 -0.15405<br>H 2.06591 2.37693 -1.13006<br>H 1.12660 3.32895 0.03976<br>H 2.34544 2.13661 0.58436<br>C 2.99110 -1.52958 0.12637<br>H 3.79077 -1.65560 0.86164<br>H 2.47663 -2.49824 0.01716<br>H 3.46134 -1.28688 -0.83283<br>N 2.14484 -0.42330 0.53721<br>H 1.77837 -0.59119 1.46985<br>H -3.13562 -1.30220 0.09788						
6-adduct, conf3	C -1.44652 -0.67164 -0.25388<br>C -1.53348 0.73576 0.20024<br>C -0.31979 1.53853 0.16533<br>C 0.86090 1.03857 -0.25144<br>C 0.98111 -0.38286 -0.75300<br>C -0.28613 -1.18623 -0.68768<br>H -0.41491 2.56857 0.49920<br>H 1.24584 -0.28024 -1.82055<br>H -0.23962 -2.22164 -1.01926<br>O -2.60471 -1.34823 -0.18734<br>O -2.62687 1.15385 0.58381<br>C 2.10797 1.86077 -0.27299<br>H 2.51812 1.91336 -1.28863<br>H 1.92062 2.87665 0.08204<br>H 2.88425 1.39295 0.34116<br>C 1.96916 -1.36404 1.27202<br>H 2.87272 -1.86615 1.62818<br>H 1.85234 -0.44295 1.85363<br>H 1.10068 -2.00561 1.48610<br>N 2.14081 -1.03213 -0.13451<br>H 2.38001 -1.86309 -0.66381<br>H -3.25240 -0.70501 0.16006	C_1	-516.08959 (0.1)	-515.89474 (0.1)	-515.94285 (0.3)	-516.59471 (0.0)	-516.44797 (0.0)
6-adduct, conf4	C -1.45919 -0.67326 -0.24555<br>C -1.54033 0.73619 0.19955<br>C -0.32068 1.53285 0.16471	C_1	-516.08862 (0.7)	-515.89378 (0.7)	-515.94145 (1.1)	-516.59411 (0.4)	-516.44695 (0.6)

	C 0.85213 1.02120 -0.25694<br>C 0.96308 -0.40824 -0.75480<br>C -0.30065 -1.20477 -0.66436<br>H -0.41053 2.56349 0.49962<br>H 1.20291 -0.31789 -1.82994<br>H -0.24155 -2.24529 -0.97107<br>O -2.62074 -1.34478 -0.17550<br>O -2.63058 1.16531 0.57928<br>C 2.10391 1.84061 -0.28376<br>H 2.57380 1.81267 -1.27450<br>H 1.90195 2.88336 -0.02883<br>H 2.84215 1.45511 0.43078<br>C 2.02581 -1.32040 1.26625<br>H 2.87666 -1.92810 1.58568<br>H 2.04636 -0.37047 1.82693<br>H 1.11182 -1.85159 1.54846<br>N 2.06753 -1.17423 -0.17895<br>H 2.95696 -0.79366 -0.48020<br>H -3.26715 -0.69737 0.16496						
6-adduct, conf5	C 1.32281 -0.90524 0.09006<br>C 1.78499 0.48756 -0.08971<br>C 0.78079 1.54028 -0.05615<br>C -0.52830 1.27650 0.11750<br>C -1.02289 -0.12731 0.36950<br>C 0.02682 -1.19060 0.28224<br>H 1.14343 2.55604 -0.19083<br>H -1.35293 -0.11557 1.43754<br>H -0.27577 -2.22298 0.42703<br>O 2.30078 -1.82496 0.04672<br>O 2.98895 0.68736 -0.25517<br>C -1.54850 2.37299 0.11468<br>H -2.23545 2.28673 0.96511<br>H -1.06991 3.35390 0.15887<br>H -2.15143 2.34427 -0.80222<br>C -2.87108 -1.65116 -0.16195<br>H -3.80648 -1.68242 -0.72662<br>H -2.28823 -2.52720 -0.45927<br>H -3.11035 -1.74586 0.91267<br>N -2.16313 -0.42766 -0.49933<br>H -2.81710 0.34530 -0.45487<br>H 3.12044 -1.31586 -0.10153	C_1	-516.08447 (3.3)	-515.88968 (3.3)	-515.93769 (3.5)	-516.58942 (3.3)	-516.44265 (3.3)

5-adduct, conf1	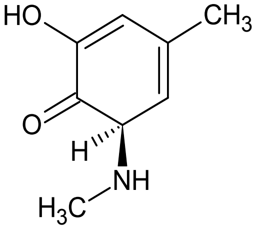<br>(6*R*)-2-hydroxy-4-methyl-6-(methylamino)cyclohexa-2,4-dien-1-one<br><br>C -0.78643 0.82342 -0.28371<br>C 0.47709 1.48519 0.05918<br>C 1.62003 0.76697 0.16857<br>C 1.66062 -0.67098 -0.06711<br>C 0.54673 -1.33166 -0.44094<br>C -0.74271 -0.63115 -0.73240<br>H 2.53176 1.27535 0.47387<br>H 0.55735 -2.40381 -0.62091<br>H -0.76455 -0.52620 -1.83597<br>O -1.83024 1.47042 -0.25075<br>O 0.39048 2.79716 0.33566<br>C 2.97786 -1.36162 0.13636<br>H 2.89322 -2.43744 -0.03501<br>H 3.73777 -0.96570 -0.54781<br>H 3.35041 -1.20474 1.15542<br>C -2.06089 -1.59371 1.07035<br>H -2.95152 -2.19958 1.25723<br>H -1.19697 -2.14161 1.45975<br>H -2.15708 -0.65380 1.63679<br>N -1.90778 -1.41126 -0.36699<br>H -2.73170 -0.94873 -0.73722<br>H -0.55001 3.02572 0.22173	C_1	-516.08680 (1.8)	-515.89208 (1.7)	-515.94015 (2.0)	-516.59049 (2.6)	-516.44383 (2.6)
5-adduct, conf2	C 0.74060 -0.87140 -0.26284<br>C -0.58028 -1.45412 0.03682<br>C -1.68324 -0.67780 0.14743<br>C -1.63090 0.76486 -0.04608<br>C -0.47121 1.35483 -0.39645<br>C 0.78008 0.57951 -0.71450<br>H -2.62692 -1.14059 0.42641<br>H -0.42245 2.43232 -0.54570<br>H 0.76449 0.45017 -1.81613<br>O 1.72404 -1.59717 -0.19286	C_1	-516.08053 (5.8)	-515.88614 (5.5)	-515.93458 (5.5)	-516.58456 (6.4)	-516.43860 (5.9)

	O -0.57191 -2.77344 0.28186<br>C -2.90007 1.53574 0.17297<br>H -2.74477 2.60816 0.03151<br>H -3.68134 1.20891 -0.52353<br>H -3.28640 1.37576 1.18637<br>C 2.24753 1.44438 1.02694<br>H 3.18361 1.99199 1.16393<br>H 1.43537 2.00947 1.51483<br>H 2.35435 0.48351 1.53977<br>N 2.04219 1.20907 -0.39225<br>H 2.13544 2.06971 -0.91848<br>H 0.36126 -3.04401 0.18904						
5-adduct, conf3	C 0.60118 0.84725 0.04423<br>C -0.76741 1.39160 0.03009<br>C -1.84359 0.57206 0.00831<br>C -1.70346 -0.87704 0.02278<br>C -0.48240 -1.44045 0.09928<br>C 0.77351 -0.64377 0.26840<br>H -2.83818 1.00671 -0.05464<br>H -0.37770 -2.52396 0.11978<br>H 0.97962 -0.67809 1.36896<br>O 1.53830 1.63096 -0.06439<br>O -0.84528 2.72806 -0.05362<br>C -2.96191 -1.69041 -0.05820<br>H -2.74603 -2.76161 -0.06369<br>H -3.61968 -1.47828 0.79295<br>H -3.52519 -1.44971 -0.96731<br>C 3.20011 -0.96613 0.10342<br>H 3.94982 -1.48238 -0.50233<br>H 3.43039 0.10046 0.09325<br>H 3.28056 -1.32900 1.14438<br>N 1.88789 -1.18551 -0.48330<br>H 1.73086 -2.17387 -0.63986<br>H 0.07996 3.03763 -0.07429	C_1	-516.08163 (5.1)	-515.88732 (4.7)	-515.93577 (4.7)	-516.58488 (6.2)	-516.43902 (5.6)
5-adduct, conf4	C 0.59488 0.83878 0.01646<br>C -0.76967 1.38738 0.06337<br>C -1.84634 0.56679 0.05394<br>C -1.71126 -0.88334 0.02146<br>C -0.48942 -1.45073 0.06244<br>C 0.76804 -0.65559 0.24909<br>H -2.84092 1.00554 0.02081<br>H -0.35948 -2.52910 0.03301<br>H 0.95776 -0.68998 1.34246	C_1	-516.08380 (3.7)	-515.88933 (3.5)	-515.93794 (3.3)	-516.58676 (5.0)	-516.44089 (4.4)

	O 1.53119 1.61287 -0.16468<br>O -0.85339 2.72676 -0.00379<br>C -2.97294 -1.69076 -0.07545<br>H -2.75685 -2.76075 -0.12316<br>H -3.61999 -1.51109 0.79135<br>H -3.54819 -1.41693 -0.96774<br>C 3.21463 -0.96180 0.17594<br>H 3.97196 -1.55034 -0.35046<br>H 3.48885 0.09940 0.12689<br>H 3.23201 -1.27726 1.22568<br>N 1.90925 -1.27733 -0.38711<br>H 1.89226 -1.08994 -1.38369<br>H 0.06799 3.04033 -0.06111						
5-adduct, conf5	C -0.05365 -1.21453 0.08834<br>C 1.39551 -0.95279 0.02544<br>C 1.88025 0.30931 -0.01140<br>C 1.00174 1.47136 0.02551<br>C -0.33077 1.31195 0.14915<br>C -0.99048 -0.03037 0.29719<br>H 2.95341 0.46006 -0.10151<br>H -0.98422 2.18097 0.17656<br>H -1.25491 -0.13523 1.36737<br>O -0.43456 -2.37546 0.02070<br>O 2.16330 -2.04942 -0.06642<br>C 1.64779 2.82130 -0.09089<br>H 0.90484 3.62245 -0.07132<br>H 2.35306 2.98909 0.73175<br>H 2.21801 2.90492 -1.02364<br>C -3.35968 0.53674 0.06111<br>H -4.22977 0.32898 -0.56792<br>H -3.59613 0.18976 1.07327<br>H -3.22581 1.63166 0.10144<br>N -2.22603 -0.21126 -0.44413<br>H -2.06866 -0.01347 -1.42644<br>H 1.53824 -2.79904 -0.05155	C_1	-516.08000 (6.1)	-515.88559 (5.8)	-515.93492 (5.2)	-516.58308 (7.3)	-516.43800 (6.3)
5-adduct, conf5	C -0.16593 -1.16728 0.01610<br>C 1.29414 -1.06827 0.07456<br>C 1.90223 0.14195 0.05941<br>C 1.14904 1.38683 -0.00319<br>C -0.19842 1.36356 0.02076<br>C -0.96385 0.09533 0.23224<br>H 2.98894 0.18163 0.04678<br>H -0.77038 2.28531 -0.04026	C_1	-516.08539 (2.7)	-515.89104 (2.4)	-515.93909 (2.6)	-516.58803 (4.2)	-516.44174 (3.9)

	H -1.10201 0.04742 1.34654<br>O -0.69507 -2.26618 -0.13486<br>O 1.95735 -2.23653 0.04286<br>C 1.93465 2.66047 -0.12195<br>H 1.27538 3.52870 -0.19663<br>H 2.58737 2.80157 0.74774<br>H 2.57904 2.64296 -1.00860<br>C -3.29297 0.79606 0.15679<br>H -4.22852 0.60452 -0.37511<br>H -3.44871 0.57456 1.22843<br>H -3.08409 1.86704 0.06458<br>N -2.23280 0.01799 -0.45200<br>H -2.49173 -0.96284 -0.51017<br>H 1.27270 -2.92708 -0.01167						

Table S9.2. Reduced neutral form in water.

Structure	Schematic drawing	Symmetry	G_{PCM}	$H_{PCM,RRHO}$	$G_{PCM,RRHO}$	$G_{SMD,M06\text{-}2X,large}$	$G_{SMD,RRHO,M06\text{-}2X,large}$
6-adduct, conf1	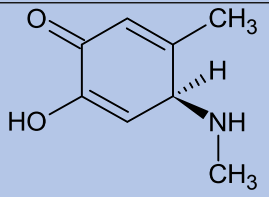<br>(4*S*)-2-hydroxy-5-methyl-4-(methylamino)cyclohexa-2,5-dien-1-one<br><br>C -1.54116 -0.82151 -0.09325<br>C -1.77004 0.62655 0.07046<br>C -0.61454 1.50319 0.04201<br>C 0.64286 1.03779 -0.12756<br>C 0.91391 -0.43987 -0.32701<br>C -0.30440 -1.31116 -0.25902<br>H -0.80804 2.56507 0.17167<br>H 1.29792 -0.52433 -1.35942<br>H -0.13550 -2.37986 -0.35607<br>O -2.65792 -1.58041 -0.04027<br>O -2.93378 1.01956 0.22909<br>C 1.80892 1.97338 -0.14158<br>H 2.39202 1.86108 -1.06214<br>H 1.47871 3.01132 -0.06218	C_1	-516.09633 (2.3)	-515.90197 (2.3)	-515.95011 (2.5)	-516.60502 (3.7)	-516.45880 (3.4)

	H 2.48718 1.76592 0.69374<br>C 3.32600 -0.95088 0.05959<br>H 3.96166 -1.48468 0.77163<br>H 3.39282 -1.47426 -0.90010<br>H 3.74423 0.05717 -0.07583<br>N 1.95372 -1.00238 0.53546<br>H 1.86730 -0.64886 1.48178<br>H -3.39003 -0.95004 0.09613						
6-adduct, conf2	C -1.33336 -0.90841 -0.10143<br>C -1.79022 0.48450 0.07252<br>C -0.79088 1.53541 0.04582<br>C 0.52407 1.27341 -0.11773<br>C 1.02154 -0.13483 -0.34453<br>C -0.03759 -1.19561 -0.29560<br>H -1.15067 2.55290 0.17563<br>H 1.42332 -0.12414 -1.37412<br>H 0.27691 -2.22798 -0.42423<br>O -2.31477 -1.83431 -0.04026<br>O -3.00149 0.68368 0.23892<br>C 1.54708 2.35949 -0.12372<br>H 2.14490 2.32128 -1.04158<br>H 1.08183 3.34423 -0.04439<br>H 2.24855 2.22063 0.70520<br>C 3.00323 -1.52323 0.08209<br>H 3.82503 -1.64917 0.79203<br>H 2.48233 -2.48960 -0.00407<br>H 3.43707 -1.28440 -0.89463<br>N 2.17103 -0.41327 0.52109<br>H 1.83667 -0.58258 1.46576<br>H -3.13646 -1.32754 0.10300	C_1	-516.09963 (0.3)	-515.90528 (0.2)	-515.95403 (0.0)	-516.60676 (2.6)	-516.46116 (1.9)
6-adduct, conf3	C -1.44233 -0.68042 -0.24068<br>C -1.54331 0.72779 0.19265<br>C -0.34166 1.53959 0.15693<br>C 0.84917 1.04490 -0.24556<br>C 0.97959 -0.37362 -0.74550<br>C -0.27644 -1.19212 -0.66173<br>H -0.44209 2.57238 0.48070<br>H 1.21067 -0.26384 -1.82010<br>H -0.21256 -2.23103 -0.97857<br>O -2.59831 -1.37461 -0.17027<br>O -2.64887 1.14277 0.56677<br>C 2.08542 1.88103 -0.25656<br>H 2.54939 1.87141 -1.24920	C_1	-516.10005 (0.0)	-515.90567 (0.0)	-515.95370 (0.2)	-516.60932 (1.0)	-516.46297 (0.7)

	H 1.86827 2.91368 0.02453<br>H 2.82963 1.46920 0.43342<br>C 2.01709 -1.35618 1.24957<br>H 2.92501 -1.86621 1.58146<br>H 1.91561 -0.43925 1.84043<br>H 1.15340 -2.00001 1.47560<br>N 2.15730 -1.01800 -0.16068<br>H 2.37241 -1.85265 -0.69576<br>H -3.25857 -0.73887 0.16548						
6-adduct, conf4	C -1.45862 -0.67407 -0.23917<br>C -1.54087 0.73436 0.19785<br>C -0.32825 1.53082 0.16272<br>C 0.84996 1.01909 -0.25350<br>C 0.96080 -0.40919 -0.75001<br>C -0.30009 -1.20789 -0.65290<br>H -0.41405 2.56390 0.49077<br>H 1.18455 -0.31228 -1.82755<br>H -0.24177 -2.24990 -0.95617<br>O -2.62364 -1.35278 -0.17045<br>O -2.64033 1.16262 0.57390<br>C 2.09634 1.84226 -0.28495<br>H 2.56004 1.81078 -1.27764<br>H 1.89118 2.88342 -0.02797<br>H 2.83959 1.45683 0.42376<br>C 2.04858 -1.30763 1.26132<br>H 2.91936 -1.88682 1.57871<br>H 2.05457 -0.35045 1.80731<br>H 1.15102 -1.85779 1.56056<br>N 2.07441 -1.17439 -0.18873<br>H 2.95493 -0.77310 -0.49228<br>H -3.27575 -0.70911 0.16582	C_1	-516.10005 (0.0)	-515.90559 (0.0)	-515.95333 (0.4)	-516.61086 (0.0)	-516.46414 (0.0)
6-adduct, conf5	C 1.32524 -0.90519 0.08138<br>C 1.78731 0.48710 -0.07873<br>C 0.78852 1.53749 -0.04695<br>C -0.52607 1.27365 0.11031<br>C -1.02045 -0.13099 0.34834<br>C 0.02791 -1.19444 0.25647<br>H 1.14744 2.55659 -0.16529<br>H -1.34760 -0.12317 1.41516<br>H -0.28121 -2.22762 0.38338<br>O 2.30547 -1.83176 0.03533<br>O 2.99989 0.68571 -0.23175<br>C -1.54238 2.37072 0.11026	C_1	-516.09698 (1.9)	-515.90246 (2.0)	-515.95046 (2.2)	-516.60786 (1.9)	-516.46134 (1.8)

	H -2.24444 2.26595 0.94486<br>H -1.06268 3.34922 0.17788<br>H -2.13143 2.35785 -0.81576<br>C -2.90366 -1.62752 -0.12231<br>H -3.83564 -1.66409 -0.69146<br>H -2.34015 -2.53332 -0.36207<br>H -3.15093 -1.65016 0.95212<br>N -2.16304 -0.43586 -0.51945<br>H -2.80385 0.35005 -0.48806<br>H 3.13027 -1.32677 -0.09591						
5-adduct, conf1	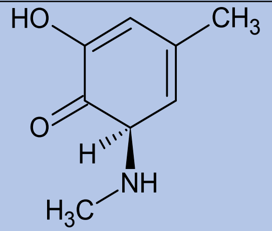<br>(6*R*)-2-hydroxy-4-methyl-6-(methylamino)cyclohexa-2,4-dien-1-one<br><br>C -0.78281 0.82708 -0.27867<br>C 0.48335 1.47982 0.05549<br>C 1.62343 0.75665 0.16743<br>C 1.66062 -0.68133 -0.06417<br>C 0.54093 -1.33426 -0.43609<br>C -0.74341 -0.62577 -0.72836<br>H 2.53911 1.26018 0.46953<br>H 0.54849 -2.40646 -0.61628<br>H -0.75578 -0.51247 -1.83130<br>O -1.82694 1.47964 -0.24185<br>O 0.41281 2.79855 0.33052<br>C 2.97485 -1.37744 0.13839<br>H 2.88295 -2.45323 -0.02832<br>H 3.73388 -0.98626 -0.54898<br>H 3.35065 -1.21578 1.15517<br>C -2.08093 -1.58409 1.06448<br>H -2.98908 -2.16406 1.24746<br>H -1.23299 -2.15246 1.45935<br>H -2.15460 -0.64146 1.62876<br>N -1.91547 -1.40349 -0.37395<br>H -2.73547 -0.93970 -0.75200<br>H -0.52194 3.04654 0.21381	C_1	-516.09517 (3.1)	-515.90099 (2.9)	-515.94920 (3.0)	-516.60434 (4.1)	-516.45838 (3.6)

5-adduct, conf2	C 0.73852 -0.89303 -0.26394<br>C -0.58054 -1.45378 0.05001<br>C -1.67396 -0.66276 0.15935<br>C -1.61404 0.77592 -0.05631<br>C -0.44745 1.34843 -0.41463<br>C 0.79547 0.55573 -0.71224<br>H -2.62258 -1.11096 0.44629<br>H -0.38571 2.42118 -0.58732<br>H 0.78770 0.43059 -1.81401<br>O 1.71842 -1.63479 -0.21096<br>O -0.59745 -2.77708 0.30873<br>C -2.87912 1.55805 0.14489<br>H -2.71457 2.62638 -0.01391<br>H -3.65907 1.22306 -0.54873<br>H -3.27045 1.41523 1.15871<br>C 2.19660 1.50030 1.03956<br>H 3.14124 2.02696 1.19665<br>H 1.37971 2.12457 1.43477<br>H 2.23051 0.57513 1.62397<br>N 2.06258 1.17824 -0.37550<br>H 2.17015 2.01857 -0.93340<br>H 0.32444 -3.07662 0.20315	C_1	-516.09243 (4.8)	-515.89832 (4.6)	-515.94666 (4.6)	-516.60188 (5.6)	-516.45611 (5.0)
5-adduct, conf3	C 0.58443 0.86379 -0.01608<br>C -0.78710 1.38070 0.06438<br>C -1.84931 0.54083 0.05539<br>C -1.68814 -0.90463 0.01379<br>C -0.45250 -1.44394 0.03685<br>C 0.78251 -0.61855 0.21379<br>H -2.85405 0.95690 0.04312<br>H -0.31972 -2.52391 0.02706<br>H 0.94681 -0.63130 1.32182<br>O 1.50585 1.65843 -0.20069<br>O -0.90475 2.72260 0.01739<br>C -2.93382 -1.73829 -0.05618<br>H -2.69694 -2.80341 -0.11029<br>H -3.56429 -1.56997 0.82447<br>H -3.53333 -1.47230 -0.93424<br>C 3.20729 -0.94874 0.21521<br>H 4.00207 -1.45127 -0.34219<br>H 3.43996 0.11707 0.25691<br>H 3.20112 -1.34304 1.24533<br>N 1.94100 -1.14852 -0.47980<br>H 1.79659 -2.14068 -0.63272	C_1	-516.09177 (5.2)	-515.89785 (4.9)	-515.94626 (4.9)	-516.60043 (6.5)	-516.45492 (5.8)

	H 0.00582 3.06636 -0.03719						
5-adduct, conf4	C 0.58964 0.84518 0.00009<br>C -0.77502 1.38055 0.07107<br>C -1.84728 0.55359 0.06252<br>C -1.70689 -0.89495 0.01747<br>C -0.47877 -1.45123 0.04131<br>C 0.77253 -0.64657 0.22573<br>H -2.84610 0.98397 0.04376<br>H -0.34572 -2.52932 0.01116<br>H 0.95324 -0.67878 1.32076<br>O 1.52163 1.62616 -0.19388<br>O -0.87747 2.72469 0.01709<br>C -2.96487 -1.70907 -0.06865<br>H -2.74239 -2.77718 -0.12622<br>H -3.60087 -1.53703 0.80743<br>H -3.55327 -1.43047 -0.95045<br>C 3.21462 -0.97071 0.21909<br>H 3.99409 -1.50971 -0.32693<br>H 3.47995 0.09370 0.25069<br>H 3.20418 -1.35544 1.24491<br>N 1.92768 -1.25189 -0.40636<br>H 1.94956 -1.00219 -1.38984<br>H 0.03660 3.05803 -0.03822	C_1	-516.09203<br>(5.0)	-515.89811<br>(4.7)	-515.94724<br>(4.3)	-516.59975<br>(7.0)	-516.45496<br>(5.8)
5-adduct, conf5	C 0.69806 0.73291 0.33409<br>C -0.52600 1.41826 -0.10057<br>C -1.69107 0.74338 -0.21421<br>C -1.78160 -0.69099 0.05101<br>C -0.68050 -1.39291 0.37872<br>C 0.68423 -0.78233 0.48465<br>H -2.59091 1.27871 -0.50829<br>H -0.73428 -2.46549 0.54722<br>H 1.09102 -0.98686 1.48756<br>O 1.70865 1.40999 0.53152<br>O -0.40227 2.74051 -0.33407<br>C -3.13816 -1.32095 -0.06886<br>H -3.54735 -1.17609 -1.07548<br>H -3.09796 -2.39322 0.13648<br>H -3.84509 -0.86117 0.63161<br>C 2.99573 -1.16879 -0.36009<br>H 3.52576 -1.79991 -1.07945<br>H 3.27525 -0.12199 -0.53911<br>H 3.33534 -1.44086 0.64499<br>N 1.57222 -1.45032 -0.47300	C_1	-516.09156<br>(5.3)	-515.89725<br>(5.3)	-515.94597<br>(5.1)	-516.59948<br>(7.1)	-516.45389<br>(6.4)

	H 1.24346 -1.27637 -1.41830<br>H 0.52409 2.95998 -0.12417						
5-adduct, conf5	C -0.05293 -1.21458 0.07042<br>C 1.39018 -0.95361 0.03661<br>C 1.87503 0.30950 0.00201<br>C 1.00133 1.47443 0.01997<br>C -0.33288 1.31111 0.12356<br>C -0.98633 -0.03190 0.27504<br>H 2.94961 0.46129 -0.07121<br>H -0.99130 2.17623 0.13907<br>H -1.23255 -0.12975 1.35086<br>O -0.43906 -2.37932 -0.01038<br>O 2.17336 -2.04865 -0.04128<br>C 1.65022 2.82318 -0.09209<br>H 0.90619 3.62322 -0.08976<br>H 2.34148 2.99349 0.74147<br>H 2.23733 2.90004 -1.01454<br>C -3.35898 0.53126 0.10100<br>H -4.23733 0.36091 -0.52709<br>H -3.58400 0.15218 1.10363<br>H -3.20401 1.61917 0.17638<br>N -2.23504 -0.21047 -0.45166<br>H -2.09131 0.05279 -1.42218<br>H 1.56295 -2.80890 -0.03081	C_1	-516.09186 (5.1)	-515.89774 (5.0)	-515.94656 (4.7)	-516.60013 (6.7)	-516.45484 (5.8)
5-adduct, conf6	C -0.14102 -1.17776 0.02043<br>C 1.31295 -1.04394 0.07260<br>C 1.89418 0.17982 0.04517<br>C 1.11568 1.40866 -0.00897<br>C -0.23167 1.35268 0.02384<br>C -0.96474 0.06631 0.23410<br>H 2.97956 0.24665 0.02450<br>H -0.82340 2.26318 -0.01255<br>H -1.10189 0.01947 1.34694<br>O -0.64814 -2.29092 -0.13140<br>O 2.01296 -2.19628 0.04060<br>C 1.87269 2.70024 -0.11621<br>H 1.19355 3.55306 -0.18725<br>H 2.51865 2.84707 0.75715<br>H 2.52262 2.70102 -0.99863<br>C -3.29615 0.76612 0.14441<br>H -4.23832 0.54292 -0.36221<br>H -3.42861 0.58652 1.22456<br>H -3.09260 1.83169 0.00253	C_1	-516.09435 (3.6)	-515.90037 (3.3)	-515.94851 (3.5)	-516.60233 (5.4)	-516.45649 (4.8)

	N -2.23846 -0.03909 -0.44810<br>H -2.50550 -1.01987 -0.43206<br>H 1.35508 -2.91322 0.00781						

Table S9.3. Reduced zwitterionic form in water.

Structura	Schematic drawing	Symmetry	G_{PCM}	$H_{PCM,RRHO}$	$G_{PCM,RRHO}$	$G_{SMD,M06-2X,large}$	$G_{SMD,RRHO,M06-2X,large}$
6-adduct, conf1	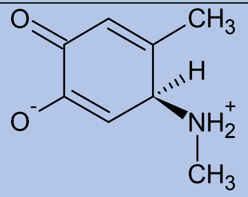<br>(3*S*)-4-methyl-3-(methylammonio)-6-oxocyclohexa-1,4-dien-1-olate<br><br>C -1.68751 -0.74848 -0.27603<br>C -1.64899 0.64950 0.34264<br>C -0.43798 1.46932 0.16633<br>C 0.71292 0.98722 -0.33145<br>C 0.80838 -0.49427 -0.62292<br>C -0.47809 -1.21238 -0.76468<br>H -0.53985 2.52464 0.41167<br>H 1.47208 -0.66268 -1.47925<br>H -0.43623 -2.20597 -1.20785<br>O -2.80418 -1.34764 -0.30735<br>O -2.62232 1.11461 0.92863<br>C 1.85839 1.87864 -0.69471<br>H 1.96800 1.89254 -1.78680<br>H 1.68089 2.90165 -0.35658<br>H 2.81917 1.54346 -0.29669<br>C 3.01209 -0.88499 0.78371<br>H 3.41073 -1.63332 1.46850<br>H 3.54327 -0.93321 -0.16667<br>H 3.10728 0.10182 1.23062<br>N 1.57706 -1.18869 0.54758<br>H 1.47661 -2.18883 0.36316<br>H 1.03533 -1.02242 1.39937	C_1	-516.07109 (4.8)	-515.87486 (5.2)	-515.92365 (4.9)	-516.59012 (4.5)	-516.44268 (3.9)
6-adduct, conf2	C -1.65051 -0.89194 -0.16858<br>C -1.81581 0.58111 0.17972<br>C -0.65880 1.48100 0.05140	C_1	-516.07177 (4.4)	saddle point			

	C 0.58483 1.06436 -0.23705<br>C 0.82787 -0.40725 -0.47380<br>C -0.36146 -1.28774 -0.47544<br>H -0.86330 2.53794 0.21125<br>H 1.41077 -0.52218 -1.39841<br>H -0.19406 -2.32042 -0.77994<br>O -2.68979 -1.61968 -0.16626<br>O -2.89832 1.03229 0.54562<br>C 1.73142 2.02149 -0.35078<br>H 2.43642 1.92433 0.48403<br>H 2.29665 1.85729 -1.27479<br>H 1.36993 3.05197 -0.34303<br>C 3.26476 -0.93189 0.28131<br>H 3.81354 -1.35519 1.12227<br>H 3.40858 -1.54675 -0.60660<br>H 3.60367 0.08355 0.09062<br>N 1.81715 -0.92688 0.61672<br>H 1.51855 -1.88439 0.81574<br>H 1.65038 -0.41377 1.48631						
6-adduct, conf3	C 1.65472 -0.88556 0.17270<br>C 1.80921 0.58608 -0.18653<br>C 0.64891 1.48059 -0.05263<br>C -0.59154 1.05916 0.24289<br>C -0.82623 -0.41373 0.48246<br>C 0.36948 -1.28580 0.48929<br>H 0.84814 2.53829 -0.21399<br>H -1.41161 -0.53076 1.40507<br>H 0.20897 -2.31798 0.79899<br>O 2.69803 -1.60731 0.17016<br>O 2.88615 1.04090 -0.56441<br>C -1.73931 2.01452 0.36275<br>H -2.31808 1.83132 1.27442<br>H -1.37610 3.04433 0.38336<br>H -2.43101 1.93803 -0.48508<br>C -3.25988 -0.92217 -0.30607<br>H -3.79470 -1.37023 -1.14317<br>H -3.43053 -1.50231 0.60025<br>H -3.59170 0.10271 -0.16069<br>N -1.80539 -0.94664 -0.60987<br>H -1.51927 -1.91415 -0.77606<br>H -1.61198 -0.46006 -1.48914	C_1	-516.07176 (4.4)	-515.87571 (4.6)	-515.92534 (3.8)	-516.59137 (3.7)	-516.44494 (2.5)
6-adduct, conf4	C -1.58140 -0.71157 -0.27780<br>C -1.57031 0.72607 0.22619	C_1	-516.07598 (1.7)	-515.87960 (2.2)	-515.92759 (2.4)	-516.59730 (0.0)	-516.44891 (0.0)

	C -0.33083 1.51373 0.13147<br>C 0.83389 1.01778 -0.31544<br>C 0.88195 -0.40388 -0.80860<br>C -0.37285 -1.18308 -0.75683<br>H -0.40392 2.55006 0.45584<br>H 1.29924 -0.38915 -1.82675<br>H -0.34003 -2.18291 -1.18791<br>O -2.68181 -1.34375 -0.24154<br>O -2.58064 1.24365 0.69832<br>C 2.08701 1.83393 -0.38493<br>H 2.53792 1.78355 -1.38238<br>H 1.88117 2.87987 -0.14915<br>H 2.84660 1.48849 0.32941<br>C 1.77029 -1.23729 1.44290<br>H 2.53723 -1.88276 1.86898<br>H 1.83723 -0.23843 1.87182<br>H 0.77794 -1.65393 1.61216<br>N 1.98491 -1.15113 -0.02301<br>H 2.89825 -0.73324 -0.21873<br>H 2.02721 -2.09702 -0.40821						
6-adduct, conf5	C -1.40433 -1.00209 -0.20678<br>C -1.81565 0.40851 0.19704<br>C -0.82942 1.49630 0.10295<br>C 0.46147 1.30179 -0.20944<br>C 0.94897 -0.09854 -0.49739<br>C -0.07069 -1.16790 -0.53914<br>H -1.20767 2.49703 0.30250<br>H 1.54843 -0.07307 -1.41871<br>H 0.24570 -2.14172 -0.90470<br>O -2.30636 -1.89336 -0.23363<br>O -2.95896 0.65646 0.57317<br>C 1.44396 2.42824 -0.30803<br>H 1.97132 2.40535 -1.26810<br>H 0.93714 3.39055 -0.21218<br>H 2.20963 2.39477 0.47848<br>C 2.80440 -1.64648 0.33138<br>H 3.53139 -1.75817 1.13516<br>H 2.13465 -2.50375 0.31031<br>H 3.31489 -1.53631 -0.62513<br>N 2.00684 -0.42122 0.58523<br>H 1.50828 -0.51826 1.47389<br>H 2.64870 0.36830 0.69190	C_1	-516.07511 (2.3)	-515.87883 (2.7)	-515.92752 (2.4)	-516.59503 (1.4)	-516.44744 (0.9)

6-adduct, conf6	C 1.40307 -1.00726 0.19335<br>C 1.82311 0.40653 -0.18686<br>C 0.83960 1.49572 -0.08806<br>C -0.45531 1.30258 0.20873<br>C -0.94672 -0.09606 0.49602<br>C 0.06568 -1.17302 0.50980<br>H 1.22128 2.49696 -0.27861<br>H -1.52147 -0.07069 1.43371<br>H -0.25482 -2.15010 0.86283<br>O 2.30102 -1.90307 0.21527<br>O 2.97025 0.65510 -0.55142<br>C -1.43893 2.43097 0.28231<br>H -2.06336 2.35819 1.17936<br>H -0.92005 3.39163 0.29758<br>H -2.11361 2.45371 -0.58427<br>C -2.79729 -1.65487 -0.32358<br>H -3.56677 -1.73835 -1.09036<br>H -2.11250 -2.49795 -0.38648<br>H -3.25452 -1.60540 0.66453<br>N -2.03743 -0.40133 -0.55602<br>H -1.57845 -0.44676 -1.46978<br>H -2.70311 0.37437 -0.59733	C_1	-516.07509 (2.3)	-515.87892 (2.6)	-515.92823 (2.0)	-516.59523 (1.3)	-516.44837 (0.3)
5-adduct, conf1	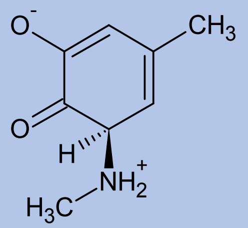<br>(5*R*)-3-methyl-5-(methylammonio)-6-oxocyclohexa-1,3-dien-1-olate<br><br>C -0.62052 1.01025 -0.23678<br>C 0.74922 1.55297 0.06011<br>C 1.73384 0.57267 0.18781<br>C 1.54906 -0.83966 -0.04908<br>C 0.36213 -1.36027 -0.44858<br>C -0.74746 -0.42405 -0.75509<br>H 2.71992 0.90336 0.51033<br>H 0.25310 -2.40644 -0.72009<br>H -0.81297 -0.29364 -1.84895<br>O -1.65208 1.66034 -0.09630	C_1	-516.07825 (0.3)	-515.88238 (0.5)	-515.93041 (0.6)	-516.59304 (2.7)	-516.44520 (2.3)

	O 0.87311 2.79742 0.26819<br>C 2.75065 -1.72372 0.13440<br>H 2.51060 -2.77523 -0.04176<br>H 3.55323 -1.43219 -0.55287<br>H 3.15079 -1.62393 1.14987<br>C -2.20341 -1.37309 1.06756<br>H -3.23375 -1.67074 1.25802<br>H -1.52676 -2.21415 1.20575<br>H -1.92250 -0.55078 1.72422<br>N -2.10670 -0.90722 -0.34086<br>H -2.42591 -1.64338 -0.97239<br>H -2.72814 -0.09504 -0.46337						
5-adduct, conf2	C 0.18333 1.18462 0.02004<br>C -1.31497 1.26602 0.10971<br>C -1.95229 0.02772 0.02428<br>C -1.28973 -1.25385 -0.02437<br>C 0.05870 -1.37957 0.06327<br>C 0.86176 -0.15538 0.31139<br>H -3.03906 0.03520 -0.04281<br>H 0.54256 -2.34983 0.12671<br>H 1.10837 -0.08790 1.38557<br>O 0.89902 2.13960 -0.26638<br>O -1.85697 2.41110 0.13693<br>C -2.16520 -2.46927 -0.14809<br>H -1.57672 -3.38848 -0.20327<br>H -2.84500 -2.54433 0.70839<br>H -2.79116 -2.40298 -1.04528<br>C 3.27942 -0.88155 0.24369<br>H 4.17056 -0.79071 -0.37613<br>H 3.46099 -0.45175 1.22804<br>H 2.99443 -1.92878 0.33548<br>N 2.17617 -0.13115 -0.40417<br>H 2.40775 0.87295 -0.49736<br>H 2.03155 -0.48331 -1.35405	C_1	-516.07876 (0.0)	-515.88312 (0.0)	-515.93139 (0.0)	-516.59173 (3.5)	-516.44436 (2.9)
5-adduct, conf3	C 0.65562 0.83016 0.39420<br>C -0.57279 1.53468 -0.14565<br>C -1.70183 0.73281 -0.28252<br>C -1.75795 -0.66997 0.04869<br>C -0.66887 -1.37285 0.45219<br>C 0.65247 -0.70494 0.55663<br>H -2.61473 1.20763 -0.63760<br>H -0.73489 -2.42241 0.72678<br>H 1.16196 -0.92823 1.50081	C_1	-516.07073 (5.0)	-515.87465 (5.3)	-515.92322 (5.1)	-516.58722 (6.3)	-516.43971 (5.8)

	O 1.67429 1.43659 0.68796<br>O -0.47391 2.77140 -0.40115<br>C -3.09904 -1.33874 -0.05772<br>H -3.49381 -1.24495 -1.07593<br>H -3.04617 -2.39985 0.19791<br>H -3.82363 -0.85559 0.60788<br>C 3.02691 -0.99262 -0.43450<br>H 3.52196 -1.51369 -1.25360<br>H 3.18045 0.08048 -0.51109<br>H 3.39549 -1.35675 0.52376<br>N 1.57054 -1.28947 -0.52009<br>H 1.20801 -1.01192 -1.43634<br>H 1.44135 -2.30353 -0.48449						

Table S9.4. Reduced anionic form in water.

Structure	Schematic drawing	Symmetry	G_{PCM}	$H_{PCM,RRHO}$	$G_{PCM,RRHO}$	$G_{SMD,M06-2X,large}$	$G_{SMD,RRHO,M06-2X,large}$
6-adduct, conf1	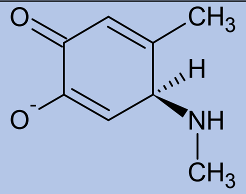(3*S*)-4-methyl-3-(methylamino)-6-oxocyclohexa-1,4-dien-1-olate<br><br>C -1.65236 -0.90724 -0.08674<br>C -1.83786 0.58249 0.07334<br>C -0.66069 1.45466 0.02858<br>C 0.60422 1.01637 -0.13101<br>C 0.87396 -0.45812 -0.32429<br>C -0.34671 -1.32114 -0.23134<br>H -0.86541 2.51881 0.14296<br>H 1.26946 -0.53561 -1.35566<br>H -0.15386 -2.38959 -0.32376<br>O -2.69442 -1.65989 -0.04957<br>O -2.95883 1.08023 0.24642<br>C 1.75417 1.97622 -0.14661<br>H 2.35531 1.86498 -1.05604<br>H 1.40053 3.00894 -0.08968<br>H 2.42596 1.80202 0.70217	C_1	-515.60945 (4.4)	-515.42894 (4.6)	-515.47709 (4.5)	-516.12776 (4.0)	-515.99541 (3.6)

	C 3.31255 -0.88569 0.05923<br>H 3.96895 -1.42707 0.74784<br>H 3.39845 -1.36553 -0.92229<br>H 3.70205 0.13893 -0.03656<br>N 1.94278 -1.00734 0.52710<br>H 1.84141 -0.67251 1.47953						
6-adduct, conf2	C -1.59500 -0.93914 -0.07390<br>C -1.85308 0.54721 0.05635<br>C -0.71874 1.47395 0.02534<br>C 0.56437 1.08455 -0.10429<br>C 0.89909 -0.37210 -0.29925<br>C -0.27144 -1.29724 -0.21469<br>H -0.97057 2.52828 0.13629<br>H 1.30016 -0.42659 -1.33458<br>H -0.04029 -2.35673 -0.33884<br>O -2.60156 -1.73418 -0.03855<br>O -3.00169 0.98885 0.19159<br>C 1.69580 2.06066 -0.08682<br>H 2.26074 2.02757 -1.02649<br>H 1.34012 3.08256 0.06646<br>H 2.39768 1.80549 0.71568<br>C 3.28330 -0.96818 -0.01479<br>H 3.98200 -1.39256 0.71224<br>H 3.25852 -1.62978 -0.89807<br>H 3.68804 -0.00306 -0.33530<br>N 1.98357 -0.79219 0.61527<br>H 1.69876 -1.66543 1.04365	C_1	-515.61158 (3.0)	-515.43114 (3.2)	-515.47956 (2.9)	-516.12918 (3.1)	-515.99716 (2.5)
6-adduct, conf3	C -1.55537 -0.75787 -0.23099<br>C -1.60205 0.69220 0.19841<br>C -0.38055 1.50051 0.12892<br>C 0.81530 1.02749 -0.27274<br>C 0.93816 -0.39337 -0.74880<br>C -0.31964 -1.20069 -0.65052<br>H -0.48999 2.53991 0.43722<br>H 1.20117 -0.29630 -1.81920<br>H -0.23289 -2.23965 -0.97437<br>O -2.63847 -1.44709 -0.17077<br>O -2.65005 1.21028 0.60820<br>C 2.04238 1.88176 -0.29980<br>H 2.49993 1.87747 -1.29601<br>H 1.81266 2.91398 -0.02333<br>H 2.80072 1.48992 0.38741<br>C 1.99497 -1.29168 1.28081	C_1	-515.61387 (1.6)	-515.43330 (1.9)	-515.48128 (1.9)	-516.13270 (0.9)	-516.00011 (0.7)

	H 2.87534 -1.84181 1.62525<br>H 1.95840 -0.34482 1.83142<br>H 1.09421 -1.86756 1.54423<br>N 2.12863 -1.02610 -0.14493<br>H 2.28898 -1.90400 -0.62933						
6-adduct, conf4	C -1.56957 -0.75217 -0.23020<br>C -1.60436 0.69752 0.20172<br>C -0.37466 1.49430 0.13704<br>C 0.81189 1.00764 -0.27388<br>C 0.92246 -0.42032 -0.75220<br>C -0.33786 -1.21439 -0.63954<br>H -0.47303 2.53334 0.45126<br>H 1.17733 -0.32812 -1.82537<br>H -0.25465 -2.25666 -0.94784<br>O -2.65962 -1.43097 -0.17220<br>O -2.64835 1.22428 0.60996<br>C 2.04794 1.85077 -0.31121<br>H 2.51614 1.82207 -1.30240<br>H 1.82648 2.89162 -0.06281<br>H 2.79826 1.48587 0.40217<br>C 2.02268 -1.27414 1.27772<br>H 2.89612 -1.83989 1.61455<br>H 2.01358 -0.30832 1.81070<br>H 1.12440 -1.82452 1.57369<br>N 2.05717 -1.16146 -0.17280<br>H 2.92772 -0.72688 -0.46314	C_1	-515.61385 (1.6)	-515.43318 (2.0)	-515.48088 (2.1)	-516.13412 (0.0)	-516.00116 (0.0)
6-adduct, conf5	C 1.39381 -1.02284 0.10217<br>C 1.84103 0.40535 -0.10748<br>C 0.84562 1.47860 -0.05034<br>C -0.47190 1.27184 0.13460<br>C -0.99137 -0.11835 0.37314<br>C 0.04046 -1.19642 0.30131<br>H 1.23526 2.48785 -0.17968<br>H -1.36976 -0.09152 1.42385<br>H -0.30820 -2.21147 0.48476<br>O 2.28611 -1.94566 0.08064<br>O 3.02838 0.68468 -0.31968<br>C -1.45182 2.40501 0.14957<br>H -2.12564 2.34066 1.01185<br>H -0.93709 3.36829 0.18382<br>H -2.07889 2.40082 -0.75167<br>C -2.87085 -1.59067 -0.19691<br>H -3.80051 -1.60710 -0.77245	C_1	-515.61149 (3.1)	-515.43076 (3.5)	-515.47855 (3.6)	-516.13161 (1.6)	-515.99868 (1.6)

	H -2.29510 -2.47787 -0.47383<br>H -3.12569 -1.67056 0.87451<br>N -2.13404 -0.37803 -0.52630<br>H -2.78186 0.39987 -0.44113						
6-adduct, conf6	C -1.39832 -1.03011 -0.11231<br>C -1.84729 0.39864 0.09577<br>C -0.85316 1.47441 0.04592<br>C 0.46613 1.27241 -0.13723<br>C 0.99314 -0.11973 -0.36327<br>C -0.04554 -1.19749 -0.32266<br>H -1.24666 2.48188 0.17785<br>H 1.43092 -0.08722 -1.37979<br>H 0.31149 -2.21331 -0.49103<br>O -2.28760 -1.95595 -0.07378<br>O -3.03554 0.67585 0.30696<br>C 1.44561 2.40208 -0.15850<br>H 2.04351 2.38280 -1.07737<br>H 0.93875 3.36805 -0.09009<br>H 2.15457 2.30977 0.67108<br>C 2.97892 -1.48309 0.13543<br>H 3.80598 -1.58212 0.84501<br>H 2.45265 -2.44995 0.09396<br>H 3.40957 -1.29157 -0.85380<br>N 2.15129 -0.35197 0.52547<br>H 1.78832 -0.51630 1.46161	C_1	-515.61340 (1.9)	-515.43282 (2.2)	-515.48143 (1.8)	-516.13041 (2.3)	-515.99844 (1.7)
5-adduct, conf1	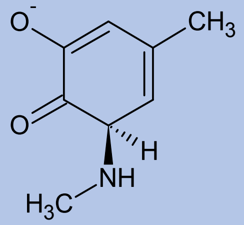<br>(5*R*)-3-methyl-5-(methylamino)-6-oxocyclohexa-1,3-dien-1-olate<br><br>C -0.69018 0.96118 -0.21346<br>C 0.67269 1.55257 0.06142<br>C 1.70431 0.62147 0.15773<br>C 1.56483 -0.80334 -0.04895<br>C 0.38472 -1.35249 -0.41957<br>C -0.79145 -0.48340 -0.71807<br>H 2.68862 0.99889 0.43582	C_1	-515.61606 (0.2)	-515.43575 (0.4)	-515.48366 (0.4)	-516.13243 (1.1)	-516.00003 (0.7)

	H 0.28366 -2.41779 -0.61418<br>H -0.76855 -0.31236 -1.81517<br>O -1.71700 1.62844 -0.10205<br>O 0.76644 2.80881 0.26611<br>C 2.79351 -1.64943 0.14864<br>H 2.58103 -2.71063 -0.00903<br>H 3.58958 -1.35300 -0.54542<br>H 3.19692 -1.52610 1.16111<br>C -2.26196 -1.40245 0.98815<br>H -3.25523 -1.83620 1.13577<br>H -1.51991 -2.13483 1.32055<br>H -2.17553 -0.51362 1.63328<br>N -2.06776 -1.12398 -0.43080<br>H -2.80057 -0.48917 -0.73488						
5-adduct, conf2	C -0.64033 1.00426 -0.19298<br>C 0.77003 1.51567 0.03236<br>C 1.75417 0.53568 0.13445<br>C 1.52902 -0.88264 -0.02483<br>C 0.31025 -1.36246 -0.36465<br>C -0.81734 -0.42887 -0.69474<br>H 2.76251 0.86772 0.38315<br>H 0.14154 -2.42589 -0.52657<br>H -0.74567 -0.25189 -1.78918<br>O -1.60765 1.74223 -0.03613<br>O 0.93601 2.76937 0.20092<br>C 2.70381 -1.79889 0.18677<br>H 2.42191 -2.84886 0.06651<br>H 3.50770 -1.57676 -0.52585<br>H 3.12814 -1.66933 1.18988<br>C -2.42638 -1.23580 0.94483<br>H -3.40688 -1.71241 1.03284<br>H -1.67495 -1.89653 1.40757<br>H -2.45541 -0.30273 1.51586<br>N -2.16156 -0.93877 -0.45659<br>H -2.27426 -1.78639 -1.00366	C_1	-515.61228 (2.6)	-515.43214 (2.6)	-515.48015 (2.6)	-516.12952 (2.9)	-515.99739 (2.4)
5-adduct, conf3	C 0.69520 0.78743 0.41593<br>C -0.49324 1.51540 -0.18559<br>C -1.65466 0.75796 -0.27141<br>C -1.74228 -0.64842 0.06615<br>C -0.65607 -1.36686 0.43374<br>C 0.70074 -0.74165 0.48599<br>H -2.56079 1.25758 -0.61394<br>H -0.73128 -2.42882 0.66138	C_1	-515.61138 (3.1)	-515.43102 (3.3)	-515.47943 (3.0)	-516.12672 (4.6)	-515.99477 (4.0)

	H 1.20881 -1.00611 1.43301<br>O 1.67522 1.40403 0.82158<br>O -0.35691 2.74568 -0.49149<br>C -3.10281 -1.28502 -0.01774<br>H -3.06912 -2.34702 0.24116<br>H -3.80924 -0.78761 0.65807<br>H -3.51604 -1.18966 -1.02930<br>C 2.94638 -1.14919 -0.46023<br>H 3.44385 -1.60522 -1.32110<br>H 3.28328 -0.11227 -0.38353<br>H 3.27214 -1.67517 0.45404<br>N 1.50693 -1.19648 -0.66559<br>H 1.22382 -2.14586 -0.88244						
5-adduct, conf4	C 0.74311 0.69125 0.48209<br>C -0.35023 1.50710 -0.18431<br>C -1.56654 0.84720 -0.30319<br>C -1.78485 -0.54031 0.06218<br>C -0.77672 -1.34258 0.47535<br>C 0.62792 -0.83451 0.53769<br>H -2.41478 1.41172 -0.69057<br>H -0.94387 -2.39401 0.69812<br>H 1.12462 -1.14611 1.46795<br>O 1.75075 1.23155 0.93129<br>O -0.09750 2.71539 -0.50569<br>C -3.19253 -1.05761 -0.05702<br>H -3.26123 -2.11274 0.22243<br>H -3.87482 -0.48591 0.58412<br>H -3.56185 -0.94822 -1.08411<br>C 2.83469 -1.15971 -0.58519<br>H 3.27906 -1.69992 -1.42680<br>H 3.10476 -0.09679 -0.67220<br>H 3.28645 -1.54051 0.33753<br>N 1.40706 -1.43538 -0.56525<br>H 0.97715 -1.18037 -1.45016	C_1	-515.61214 (2.7)	-515.43166 (2.9)	-515.47975 (2.8)	-516.12779 (4.0)	-515.99540 (3.6)
5-adduct, conf5	C 0.56595 0.91248 -0.09863<br>C -0.82441 1.48846 0.10378<br>C -1.85519 0.55145 0.09191<br>C -1.66669 -0.87661 -0.00674<br>C -0.42848 -1.42509 -0.00456<br>C 0.77706 -0.56612 0.20357<br>H -2.87639 0.93148 0.12296<br>H -0.28571 -2.50428 -0.01625<br>H 0.90682 -0.51273 1.31715	C_1	-515.61283 (2.2)	-515.43279 (2.2)	-515.48080 (2.2)	-516.12846 (3.6)	-515.99642 (3.0)

	O 1.50364 1.62592 -0.44110<br>O -0.95692 2.75556 0.15128<br>C -2.89988 -1.73500 -0.08540<br>H -3.52166 -1.45204 -0.94327<br>H -2.64795 -2.79517 -0.17882<br>H -3.52142 -1.60854 0.80952<br>C 3.20871 -0.83767 0.31237<br>H 4.03611 -1.36782 -0.16833<br>H 3.42318 0.23244 0.28103<br>H 3.16686 -1.14928 1.37112<br>N 1.97543 -1.11162 -0.41214<br>H 1.85597 -2.11601 -0.48940						
5-adduct, conf6	C 0.56592 0.87606 -0.14351<br>C -0.79130 1.48689 0.14655<br>C -1.84062 0.57071 0.14439<br>C -1.68449 -0.85966 -0.00917<br>C -0.45847 -1.43466 -0.03760<br>C 0.76457 -0.60052 0.19527<br>H -2.85283 0.97002 0.21165<br>H -0.32308 -2.51170 -0.09482<br>H 0.88009 -0.54880 1.29998<br>O 1.48221 1.54194 -0.61566<br>O -0.88817 2.75553 0.23927<br>C -2.93871 -1.68460 -0.11088<br>H -3.55430 -1.36306 -0.95994<br>H -2.71217 -2.74748 -0.23437<br>H -3.55735 -1.56873 0.78761<br>C 3.18735 -0.85455 0.42725<br>H 4.05657 -1.29845 -0.06764<br>H 3.36348 0.22576 0.53155<br>H 3.12051 -1.29160 1.43010<br>N 1.98553 -1.19309 -0.32541<br>H 2.10257 -0.89210 -1.28880	C_1	-515.61309 (2.1)	-515.43306 (2.0)	-515.48175 (1.6)	-516.12828 (3.7)	-515.99694 (2.6)
5-adduct, conf7	C 0.04508 -1.25862 0.04585<br>C -1.45294 -1.07660 -0.12755<br>C -1.89936 0.24080 -0.08498<br>C -1.03912 1.39555 0.03351<br>C 0.30920 1.27488 0.00131<br>C 0.95157 -0.05963 -0.24108<br>H -2.97701 0.40444 -0.09994<br>H 0.95063 2.15156 0.04393<br>H 1.05422 -0.15574 -1.34471<br>O 0.50833 -2.35071 0.35277	C_1	-515.61161 (3.0)	-515.43155 (3.0)	-515.48078 (2.2)	-516.12707 (4.4)	-515.99623 (3.1)

	O -2.18264 -2.12033 -0.19275<br>C -1.70337 2.73816 0.17805<br>H -0.96903 3.54276 0.27631<br>H -2.33633 2.95681 -0.69088<br>H -2.35850 2.76061 1.05753<br>C 3.29250 0.64235 -0.25590<br>H 4.26099 0.42337 0.20366<br>H 3.38404 0.45371 -1.33166<br>H 3.09295 1.71768 -0.12056<br>N 2.28863 -0.24783 0.30279<br>H 2.25080 -0.13347 1.31136						
5-adduct, conf8	C -0.18247 -1.20833 -0.03207<br>C 1.32076 -1.21420 0.10615<br>C 1.91648 0.04429 0.06889<br>C 1.20323 1.29794 -0.03094<br>C -0.14962 1.34170 -0.01100<br>C -0.93271 0.09053 0.21732<br>H 3.00608 0.07771 0.07728<br>H -0.68732 2.28552 -0.05226<br>H -1.04828 0.01928 1.33255<br>O -0.81520 -2.23438 -0.27855<br>O 1.92324 -2.33753 0.15255<br>C 2.02678 2.55206 -0.14671<br>H 1.39585 3.44001 -0.24520<br>H 2.66641 2.68341 0.73477<br>H 2.69401 2.50559 -1.01587<br>C -3.23384 0.88542 0.22742<br>H -4.20567 0.72063 -0.24643<br>H -3.33528 0.68138 1.30815<br>H -2.98845 1.94603 0.11167<br>N -2.23418 0.05638 -0.42405<br>H -2.53412 -0.91553 -0.42271	C_1	-515.61638 (0.0)	-515.43632 (0.0)	-515.48426 (0.0)	-516.13067 (2.2)	-515.99855 (1.6)

Table S9.5. One-electron oxidized neutral form in vacuo.

Structure	Schematic drawing	Symmetry	*E*	H_{RRHO}	G_{RRHO}	$E_{M06\text{-}2X,large}$	$G_{RRHO,M06\text{-}2X,large}$
6-adduct, conf1	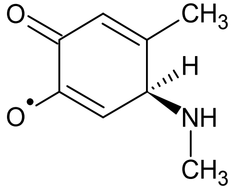<br>[(3*S*)-4-methyl-3-(methylamino)-6-oxocyclohexa-1,4-dien-1-yl]oxidanyl<br><br>C -1.62165 -0.90986 -0.11701<br>C -1.86044 0.58095 0.13036<br>C -0.68984 1.46451 0.05808<br>C 0.56978 1.04889 -0.18339<br>C 0.90557 -0.41492 -0.38459<br>C -0.28268 -1.32150 -0.35618<br>H -0.90452 2.52109 0.20511<br>H 1.36539 -0.50785 -1.38395<br>H -0.09567 -2.38357 -0.49095<br>O -2.56803 -1.70885 -0.10575<br>O -2.97519 1.00153 0.37239<br>C 1.70593 2.02204 -0.25569<br>H 2.25309 1.91777 -1.20026<br>H 1.34864 3.05096 -0.17472<br>H 2.42864 1.84988 0.55018<br>C 3.28493 -0.92350 0.20620<br>H 3.85336 -1.43745 0.98619<br>H 3.43131 -1.48432 -0.72314<br>H 3.72318 0.07823 0.06945<br>N 1.88177 -0.94352 0.56253<br>H 1.70025 -0.62115 1.50455	C_1	-515.43008 (9.7)	-515.24920 (9.9)	-515.29908 (9.9)	-515.92907 (9.1)	-515.79807 (9.0)
6-adduct, conf2	C -1.39689 -1.02235 -0.14435<br>C -1.87774 0.40447 0.13500<br>C -0.87081 1.47114 0.08127<br>C 0.43882 1.27076 -0.16265<br>C 1.00453 -0.11211 -0.40184<br>C -0.01377 -1.19957 -0.42808<br>H -1.25500 2.47474 0.25067<br>H 1.52386 -0.08544 -1.37459<br>H 0.32081 -2.21783 -0.61515	C_1	-515.43423 (7.1)	-515.25323 (7.4)	-515.30316 (7.4)	-515.93261 (6.9)	-515.80153 (6.8)

	O -2.19489 -1.96871 -0.13461<br>O -3.04666 0.62547 0.38590<br>C 1.42156 2.39513 -0.21760<br>H 1.86158 2.46863 -1.22060<br>H 0.95092 3.35087 0.02197<br>H 2.24681 2.20965 0.47685<br>C 3.02872 -1.39412 0.21385<br>H 3.73689 -1.52412 1.03636<br>H 2.61510 -2.38408 -0.03621<br>H 3.59077 -1.03240 -0.65378<br>N 2.04565 -0.39586 0.59496<br>H 1.61449 -0.62325 1.48589						
6-adduct, conf3	C -1.53311 -0.74028 -0.25832<br>C -1.62007 0.71090 0.22209<br>C -0.39177 1.51072 0.15267<br>C 0.79976 1.04670 -0.27253<br>C 0.98110 -0.37428 -0.76228<br>C -0.26860 -1.19430 -0.72652<br>H -0.49795 2.54399 0.47603<br>H 1.29005 -0.29514 -1.81959<br>H -0.21158 -2.22990 -1.05951<br>O -2.53037 -1.47386 -0.23714<br>O -2.66958 1.16246 0.63799<br>C 2.01907 1.90944 -0.31165<br>H 2.41541 1.96821 -1.33307<br>H 1.80319 2.92105 0.03817<br>H 2.81599 1.47239 0.29849<br>C 1.88765 -1.33399 1.32400<br>H 2.80330 -1.77074 1.73097<br>H 1.68356 -0.41542 1.88406<br>H 1.05734 -2.03259 1.50917<br>N 2.11175 -1.00772 -0.07748<br>H 2.41374 -1.82600 -0.59390	C_1	-515.43307 (7.9)	-515.25215 (8.1)	-515.30196 (8.1)	-515.93339 (6.4)	-515.80228 (6.4)
6-adduct, conf4	C -1.55242 -0.76823 -0.22169<br>C -1.65546 0.69673 0.21237<br>C -0.42732 1.49940 0.14964<br>C 0.76363 1.02949 -0.26791<br>C 0.94875 -0.39985 -0.74913<br>C -0.27732 -1.24073 -0.63696<br>H -0.53936 2.53602 0.46116<br>H 1.17140 -0.33018 -1.83076<br>H -0.18383 -2.29317 -0.89454<br>O -2.54768 -1.50462 -0.18725	C_1	-515.43246 (8.2)	-515.25157 (8.4)	-515.30072 (8.9)	-515.93325 (6.5)	-515.80152 (6.9)

	O -2.71389 1.15715 0.59381<br>C 1.97928 1.90066 -0.31873<br>H 2.43153 1.88546 -1.31857<br>H 1.73829 2.93533 -0.06616<br>H 2.74647 1.55073 0.38308<br>C 2.06238 -1.23158 1.28022<br>H 2.94665 -1.78836 1.60032<br>H 2.04298 -0.26883 1.81716<br>H 1.18356 -1.80490 1.59228<br>N 2.07872 -1.11820 -0.16937<br>H 2.95471 -0.73029 -0.49778						
6-adduct, conf5	C 1.39223 -1.04655 0.05423<br>C 1.91638 0.38435 -0.06410<br>C 0.91652 1.45771 -0.04414<br>C -0.40817 1.26320 0.09497<br>C -0.99719 -0.10910 0.33408<br>C -0.01176 -1.22005 0.19642<br>H 1.31887 2.46283 -0.14931<br>H -1.29863 -0.10661 1.41194<br>H -0.37004 -2.24517 0.23341<br>O 2.16958 -2.00941 0.00814<br>O 3.10647 0.60513 -0.17636<br>C -1.36421 2.41687 0.09625<br>H -2.07238 2.34958 0.93131<br>H -0.83063 3.36651 0.17382<br>H -1.94936 2.44827 -0.83184<br>C -3.00957 -1.44911 -0.06689<br>H -3.93017 -1.44544 -0.65557<br>H -2.52138 -2.41116 -0.24711<br>H -3.27799 -1.39750 1.00323<br>N -2.16572 -0.35202 -0.51026<br>H -2.72194 0.49305 -0.55727	C_1	-515.42824 (10.9)	-515.24748 (11.0)	-515.29754 (10.9)	-515.92824 (9.6)	-515.79753 (9.4)
5-adduct, conf1	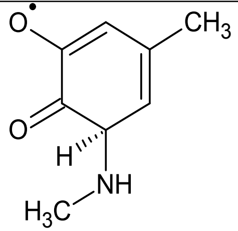<br>[(5*R*)-3-methyl-5-(methylamino)-6-oxocyclohexa-1,3-dien-1-yl]oxidanyl	C_1	-515.44548 (0.1)	-515.26459 (0.3)	-515.31436 (0.4)	-515.94357 (0.0)	-515.81245 (0.0)

	C -0.67175 1.00816 -0.15502<br>C 0.76173 1.52667 0.03846<br>C 1.79153 0.52610 0.13987<br>C 1.55745 -0.85315 -0.02432<br>C 0.30772 -1.30911 -0.39196<br>C -0.83086 -0.40532 -0.73499<br>H 2.78441 0.88684 0.39900<br>H 0.12122 -2.37403 -0.51034<br>H -0.75256 -0.22918 -1.82691<br>O -1.62953 1.69683 0.10287<br>O 0.97025 2.73167 0.14579<br>C 2.69400 -1.81582 0.18870<br>H 3.49634 -1.63343 -0.53437<br>H 3.12429 -1.69654 1.18839<br>H 2.36803 -2.85261 0.07767<br>C -2.40506 -1.29636 0.90356<br>H -3.38809 -1.76928 0.97242<br>H -1.67246 -2.00645 1.30128<br>H -2.40496 -0.40117 1.54257<br>N -2.11310 -1.02908 -0.49970<br>H -2.83876 -0.43557 -0.88692						
5-adduct, conf2	C -1.15550 0.47122 -0.56171<br>C -0.30022 1.47413 0.22945<br>C 1.10736 1.18724 0.33366<br>C 1.70348 0.01628 -0.17887<br>C 0.92242 -0.96404 -0.75494<br>C -0.57677 -0.92584 -0.81548<br>H 1.71545 1.94492 0.82286<br>H 1.40061 -1.86366 -1.14061<br>H -0.91288 -1.20162 -1.82279<br>O -2.24648 0.78218 -0.97079<br>O -0.82026 2.47307 0.71747<br>C 3.19728 -0.13815 -0.08261<br>H 3.53166 -1.08824 -0.50591<br>H 3.70611 0.67128 -0.61668<br>H 3.52626 -0.09446 0.96111<br>C -0.93718 -1.70038 1.51568<br>H -1.43667 -2.50070 2.06755<br>H 0.13079 -1.70779 1.78587<br>H -1.37024 -0.75549 1.86114<br>N -1.21639 -1.87777 0.09993<br>H -1.00759 -2.82464 -0.19648	C_1	-515.43668 (5.6)	-515.25571 (5.9)	-515.30598 (5.6)	-515.93547 (5.1)	-515.80477 (4.8)

5-adduct, conf3	C 0.58374 1.04960 0.07920<br>C -0.89671 1.46904 0.02339<br>C -1.85983 0.40169 -0.07824<br>C -1.50509 -0.95870 -0.01154<br>C -0.20690 -1.31380 0.29906<br>C 0.84901 -0.32083 0.71015<br>H -2.88766 0.69765 -0.27579<br>H 0.07476 -2.36515 0.32443<br>H 0.67651 -0.11836 1.78581<br>O 1.44968 1.77285 -0.34054<br>O -1.19426 2.65868 0.00136<br>C -2.55639 -2.00543 -0.26245<br>H -3.35337 -1.94005 0.48617<br>H -3.02037 -1.86519 -1.24412<br>H -2.13740 -3.01376 -0.22298<br>C 2.64357 -1.09655 -0.76662<br>H 3.67625 -1.45215 -0.72679<br>H 2.02829 -1.87736 -1.24604<br>H 2.62665 -0.20545 -1.39886<br>N 2.21983 -0.74051 0.57658<br>H 2.42571 -1.47888 1.23790	C_1	-515.43947 (3.8)	-515.25891 (3.8)	-515.30862 (4.0)	-515.93768 (3.7)	-515.80683 (3.5)
5-adduct, conf4	C 0.79336 0.71248 0.56699<br>C -0.26213 1.48046 -0.24449<br>C -1.53928 0.82236 -0.39367<br>C -1.78941 -0.48736 0.06682<br>C -0.75744 -1.25872 0.56089<br>C 0.66740 -0.80571 0.54754<br>H -2.33550 1.41067 -0.84388<br>H -0.96148 -2.26453 0.92253<br>H 1.20494 -1.21002 1.42182<br>O 1.69254 1.28456 1.13505<br>O -0.03259 2.60986 -0.65760<br>C -3.19241 -1.02555 -0.00045<br>H -3.24894 -2.05152 0.37085<br>H -3.87457 -0.40845 0.59361<br>H -3.56032 -1.01502 -1.03197<br>C 2.75340 -1.31235 -0.64399<br>H 3.13523 -1.59277 -1.62905<br>H 3.18998 -0.34545 -0.37812<br>H 3.09791 -2.05330 0.09673<br>N 1.30772 -1.19276 -0.71533<br>H 0.87818 -2.02936 -1.09261	C_1	-515.44068 (3.1)	-515.25982 (3.3)	-515.31003 (3.1)	-515.93851 (3.2)	-515.80786 (2.9)

5-adduct, conf5	C 0.78539 0.71183 0.51218<br>C -0.33503 1.49923 -0.18782<br>C -1.60467 0.82554 -0.30203<br>C -1.80711 -0.52251 0.06647<br>C -0.74673 -1.29995 0.48218<br>C 0.66316 -0.80534 0.53212<br>H -2.43066 1.41817 -0.68895<br>H -0.90141 -2.35057 0.71485<br>H 1.16012 -1.16320 1.44373<br>O 1.75441 1.28024 0.96452<br>O -0.14566 2.65020 -0.56748<br>C -3.19470 -1.09588 -0.02769<br>H -3.21156 -2.15442 0.24149<br>H -3.87968 -0.56234 0.63983<br>H -3.58826 -0.99532 -1.04468<br>C 2.83622 -1.20549 -0.58377<br>H 3.25294 -1.68727 -1.47240<br>H 3.17166 -0.15865 -0.55446<br>H 3.24388 -1.71212 0.29697<br>N 1.39223 -1.37987 -0.60330<br>H 0.98819 -1.06325 -1.47941	C_1	-515.44268 (1.8)	-515.26159 (2.2)	-515.31171 (2.0)	-515.94007 (2.2)	-515.80910 (2.1)
5-adduct, conf6	C 0.61409 0.87348 -0.17927<br>C -0.76005 1.49452 0.13398<br>C -1.87617 0.58401 0.13676<br>C -1.72435 -0.80731 -0.00841<br>C -0.46180 -1.36609 -0.00531<br>C 0.78041 -0.58010 0.26676<br>H -2.86611 1.03111 0.18947<br>H -0.34785 -2.43886 -0.15072<br>H 0.83489 -0.47396 1.38202<br>O 1.48347 1.51791 -0.70727<br>O -0.86506 2.70652 0.28931<br>C -2.94790 -1.67059 -0.15047<br>H -3.54560 -1.36025 -1.01362<br>H -2.68547 -2.72355 -0.27774<br>H -3.58618 -1.58500 0.73548<br>C 3.21243 -0.78380 0.33023<br>H 4.02496 -1.39068 -0.07710<br>H 3.40664 0.25762 0.06858<br>H 3.22452 -0.87807 1.43095<br>N 1.95853 -1.21153 -0.27071<br>H 1.86330 -2.21778 -0.19423	C_1	-515.44019 (3.4)	-515.25977 (3.3)	-515.30955 (3.4)	-515.93789 (3.6)	-515.80725 (3.3)

5-adduct, conf7	C 0.57843 0.88586 -0.19964<br>C -0.78891 1.48560 0.16170<br>C -1.88280 0.54842 0.16788<br>C -1.70294 -0.83776 -0.00800<br>C -0.42991 -1.37591 -0.02498<br>C 0.80014 -0.56461 0.25047<br>H -2.88176 0.97147 0.24552<br>H -0.26980 -2.43962 -0.18128<br>H 0.88593 -0.49789 1.35269<br>O 1.40097 1.50799 -0.82551<br>O -0.90673 2.69135 0.35420<br>C -2.91186 -1.72174 -0.14945<br>H -3.52704 -1.40890 -0.99950<br>H -2.62954 -2.76668 -0.29696<br>H -3.54037 -1.66314 0.74574<br>C 3.21641 -0.80596 0.45740<br>H 4.06733 -1.30806 -0.01049<br>H 3.41278 0.27564 0.46449<br>H 3.15801 -1.16025 1.49251<br>N 1.99994 -1.18721 -0.24844<br>H 2.09960 -0.97020 -1.23550	C_1	-515.44521 (0.2)	-515.26443 (0.4)	-515.31439 (0.3)	-515.94252 (0.7)	-515.81169 (0.5)
5-adduct, conf8	C 0.34128 -1.22995 -0.44138<br>C -1.00960 -1.32168 0.28421<br>C -1.77769 -0.10108 0.33419<br>C -1.30004 1.13881 -0.13872<br>C 0.00270 1.26454 -0.58587<br>C 0.99775 0.14916 -0.50035<br>H -2.78453 -0.18319 0.73757<br>H 0.35900 2.23542 -0.92363<br>H 1.65548 0.13609 -1.37650<br>O 0.84525 -2.20228 -0.94699<br>O -1.40785 -2.38997 0.73509<br>C -2.22932 2.32228 -0.12876<br>H -1.73271 3.22770 -0.48545<br>H -3.09970 2.13676 -0.76702<br>H -2.60421 2.51391 0.88236<br>C 3.02726 1.12714 0.50444<br>H 3.58055 1.16660 1.44672<br>H 3.68470 0.67566 -0.24497<br>H 2.80740 2.16177 0.19322<br>N 1.85882 0.28325 0.68380<br>H 1.30894 0.58926 1.47979	C_1	-515.43763 (5.0)	-515.25663 (5.3)	-515.30818 (4.2)	-515.93460 (5.6)	-515.80515 (4.6)

5-adduct, conf9	C -0.27543 -1.19395 -0.12512<br>C 1.22709 -1.28286 0.17142<br>C 1.94826 -0.03684 0.15169<br>C 1.32726 1.21233 -0.04355<br>C -0.04998 1.29754 -0.07522<br>C -0.94297 0.13104 0.21733<br>H 3.03085 -0.10957 0.22615<br>H -0.53856 2.24588 -0.28358<br>H -1.01382 0.06710 1.33280<br>O -0.89585 -2.12792 -0.57333<br>O 1.75314 -2.37724 0.34814<br>C 2.18299 2.43776 -0.21364<br>H 2.87321 2.31849 -1.05509<br>H 1.57666 3.32879 -0.39257<br>H 2.78854 2.61379 0.68203<br>C -3.22369 0.95393 0.39717<br>H -4.18653 0.93342 -0.11966<br>H -3.36355 0.54149 1.41243<br>H -2.93557 2.00596 0.50256<br>N -2.24149 0.23950 -0.39281<br>H -2.56029 -0.69441 -0.63432	C_1	-515.44560<br>(0.0)	-515.26504<br>(0.0)	-515.31494<br>(0.0)	-515.94242<br>(0.7)	-515.81176<br>(0.4)
5-adduct, conf10	C -0.08863 -1.25227 -0.14031<br>C 1.40143 -1.12520 0.20786<br>C 1.92699 0.21746 0.16415<br>C 1.11060 1.34181 -0.06324<br>C -0.26554 1.20478 -0.06023<br>C -0.95320 -0.08291 0.32110<br>H 3.00785 0.31789 0.23148<br>H -0.89398 2.06620 -0.27213<br>H -0.91221 -0.13732 1.42925<br>O -0.51694 -2.18531 -0.77103<br>O 2.07716 -2.12484 0.42329<br>C 1.75362 2.68384 -0.28560<br>H 2.31990 2.99199 0.60006<br>H 2.45633 2.64681 -1.12427<br>H 1.00964 3.45594 -0.49579<br>C -3.26078 0.75670 0.31108<br>H -4.26669 0.43939 0.02298<br>H -3.25232 0.86149 1.40241<br>H -3.08906 1.75721 -0.12277<br>N -2.31711 -0.26325 -0.08090<br>H -2.38834 -0.54078 -1.05183	C_1	-515.44062<br>(3.1)	-515.26023<br>(3.0)	-515.31059<br>(2.7)	-515.93769<br>(3.7)	-515.80766<br>(3.0)

Table S9.6. One-electron oxidized neutral form in water.

Structure	Schematic drawing	Symmetry	G_{PCM}	$H_{PCM,RRHO}$	$G_{PCM,RRHO}$	$G_{SMD,M06\text{-}2X,large}$	$G_{SMD,RRHO,M06\text{-}2X,large}$
6-adduct, conf1	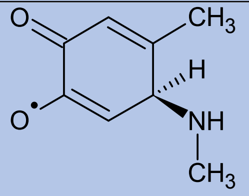<br>[(3*S*)-4-methyl-3-(methylamino)-6-oxocyclohexa-1,4-dien-1-yl]oxidanyl<br><br>C -1.59827 -0.90622 -0.13163<br>C -1.83441 0.57734 0.14773<br>C -0.68703 1.46988 0.06176<br>C 0.57425 1.06115 -0.20516<br>C 0.91123 -0.39773 -0.40961<br>C -0.26929 -1.30983 -0.40995<br>H -0.89927 2.52485 0.22058<br>H 1.40314 -0.48824 -1.39110<br>H -0.07911 -2.36360 -0.59469<br>O -2.55659 -1.69971 -0.12158<br>O -2.96061 0.97239 0.42331<br>C 1.70357 2.03546 -0.28801<br>H 2.22138 1.94464 -1.24963<br>H 1.35040 3.06201 -0.17337<br>H 2.44787 1.84031 0.49234<br>C 3.24896 -0.97346 0.25778<br>H 3.78754 -1.43621 1.08837<br>H 3.39550 -1.60156 -0.62640<br>H 3.70464 0.00822 0.05844<br>N 1.83660 -0.92525 0.58843<br>H 1.65529 -0.53460 1.50522	C_1	-515.44956 (7.1)	-515.26852 (7.3)	-515.31836 (7.2)	-515.95376 (7.5)	-515.82257 (7.6)
6-adduct, conf2	C -1.65212 -0.67772 -0.28363<br>C -1.56691 0.70082 0.38301<br>C -0.34913 1.46524 0.14784<br>C 0.77397 0.92220 -0.36924<br>C 0.88064 -0.57594 -0.59241<br>C -0.45325 -1.20534 -0.83736<br>H -0.40168 2.52855 0.37059<br>H 1.52741 -0.74277 -1.47124	C_1	-515.44244 (11.6)	-515.26128 (11.9)	-515.31097 (11.9)	-515.94945 (10.2)	-515.81798 (10.5)

	H -0.50256 -2.16877 -1.33958<br>O -2.73936 -1.27051 -0.34338<br>O -2.52688 1.13680 1.00294<br>C 1.93478 1.76706 -0.77699<br>H 2.00701 1.76052 -1.87256<br>H 1.81576 2.80099 -0.44709<br>H 2.88713 1.37824 -0.40639<br>C 2.77791 -0.89558 0.98300<br>H 3.12182 -1.60847 1.73632<br>H 3.49148 -0.90899 0.14354<br>H 2.79336 0.09723 1.44074<br>N 1.41690 -1.25925 0.60475<br>H 1.39644 -2.25665 0.41073						
6-adduct, conf3	C -1.38974 -1.01282 -0.15466<br>C -1.85768 0.41153 0.14367<br>C -0.86431 1.47534 0.08365<br>C 0.44610 1.26787 -0.16831<br>C 1.00166 -0.11746 -0.40680<br>C -0.01515 -1.19945 -0.44552<br>H -1.23949 2.48160 0.25617<br>H 1.53867 -0.09230 -1.36765<br>H 0.32427 -2.21392 -0.64066<br>O -2.21336 -1.94534 -0.15985<br>O -3.03378 0.62025 0.41434<br>C 1.42832 2.38813 -0.23121<br>H 2.26484 2.19410 0.44708<br>H 1.84997 2.46035 -1.24157<br>H 0.96692 3.34397 0.02337<br>C 3.03552 -1.37867 0.22723<br>H 3.69881 -1.54453 1.07948<br>H 2.65550 -2.35737 -0.10236<br>H 3.62731 -0.95073 -0.58802<br>N 2.00454 -0.43130 0.61806<br>H 1.55025 -0.70813 1.48354	C_1	-515.45277 (5.1)	-515.27176 (5.3)	-515.32169 (5.1)	-515.95567 (6.3)	-515.82459 (6.3)
6-adduct, conf4	C -1.51966 -0.77353 -0.22857<br>C -1.64430 0.68523 0.20842<br>C -0.44662 1.50966 0.14537<br>C 0.76240 1.06064 -0.25853<br>C 0.97129 -0.35423 -0.75064<br>C -0.24437 -1.21660 -0.66509<br>H -0.57099 2.54498 0.45420<br>H 1.20932 -0.25904 -1.82513<br>H -0.14789 -2.26037 -0.95823	C_1	-515.45150 (5.9)	-515.27052 (6.1)	-515.31996 (6.2)	-515.95761 (5.1)	-515.82607 (5.4)

	O -2.51389 -1.51946 -0.19588<br>O -2.72508 1.11237 0.59531<br>C 1.96051 1.94826 -0.27861<br>H 2.42790 1.93832 -1.27004<br>H 1.70032 2.97507 -0.01493<br>H 2.71777 1.57736 0.42007<br>C 2.00753 -1.30466 1.27147<br>H 2.95164 -1.72570 1.62546<br>H 1.80643 -0.40135 1.85712<br>H 1.20716 -2.03033 1.48052<br>N 2.15488 -0.95290 -0.13586<br>H 2.44522 -1.76052 -0.67631						
6-adduct, conf5	C -1.54532 -0.77368 -0.21469<br>C -1.65290 0.69063 0.21010<br>C -0.44309 1.49812 0.14925<br>C 0.75488 1.02915 -0.26286<br>C 0.94433 -0.39967 -0.74166<br>C -0.27099 -1.24877 -0.61801<br>H -0.55401 2.53695 0.45224<br>H 1.13867 -0.32251 -1.82806<br>H -0.17144 -2.30279 -0.86582<br>O -2.55033 -1.50492 -0.18034<br>O -2.73015 1.13508 0.58606<br>C 1.96125 1.90647 -0.31651<br>H 2.40984 1.88424 -1.31686<br>H 1.71444 2.93937 -0.06453<br>H 2.73159 1.55823 0.38197<br>C 2.09883 -1.21462 1.26830<br>H 3.00316 -1.74482 1.57574<br>H 2.07019 -0.24806 1.79561<br>H 1.23841 -1.80497 1.59972<br>N 2.08971 -1.10882 -0.18492<br>H 2.95311 -0.70103 -0.52482	C_1	-515.45151 (5.9)	-515.27059 (6.0)	-515.31959 (6.5)	-515.95935 (4.0)	-515.82743 (4.5)
6-adduct, conf6	C 1.38125 -1.04360 0.05180<br>C 1.90790 0.38446 -0.05911<br>C 0.92657 1.45793 -0.03969<br>C -0.40392 1.26528 0.09059<br>C -0.99576 -0.10579 0.32195<br>C -0.02063 -1.22007 0.17768<br>H 1.32601 2.46467 -0.13612<br>H -1.27559 -0.10909 1.40396<br>H -0.38713 -2.24236 0.20637<br>O 2.17025 -2.00342 0.00790	C_1	-515.44785 (8.2)	-515.26693 (8.3)	-515.31651 (8.4)	-515.95570 (6.3)	-515.82437 (6.5)

	O 3.11102 0.58404 -0.16860<br>C -1.35258 2.41972 0.08894<br>H -2.06108 2.35208 0.92245<br>H -0.81685 3.36802 0.15936<br>H -1.93962 2.44109 -0.83787<br>C -3.00528 -1.45524 -0.05681<br>H -3.94949 -1.43104 -0.60539<br>H -2.52425 -2.41086 -0.28289<br>H -3.22702 -1.42719 1.02241<br>N -2.18112 -0.33800 -0.50503<br>H -2.75258 0.49948 -0.48341						
5-adduct, conf1	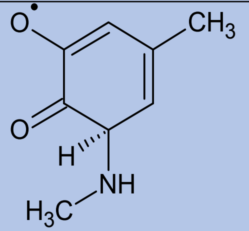<br>[(5*R*)-3-methyl-5-(methylamino)-6-oxocyclohexa-1,3-dien-1-yl]oxidanyl<br><br>C -0.67542 0.99793 -0.16689<br>C 0.75042 1.52052 0.04200<br>C 1.78523 0.53760 0.14280<br>C 1.56646 -0.84278 -0.02530<br>C 0.31750 -1.30890 -0.38865<br>C -0.82936 -0.41637 -0.72904<br>H 2.77673 0.90128 0.40243<br>H 0.14430 -2.37558 -0.50648<br>H -0.74854 -0.24316 -1.82139<br>O -1.63309 1.70307 0.06740<br>O 0.93819 2.73590 0.16727<br>C 2.71167 -1.79368 0.17891<br>H 3.49636 -1.61222 -0.56321<br>H 3.16057 -1.65342 1.16723<br>H 2.38936 -2.83298 0.08829<br>C -2.39748 -1.30939 0.91602<br>H -3.38364 -1.77409 0.99049<br>H -1.66575 -2.01888 1.31535<br>H -2.38984 -0.41159 1.55128<br>N -2.10783 -1.04733 -0.49017<br>H -2.83978 -0.46939 -0.88923	C_1	-515.46094 (0.0)	-515.28010 (0.0)	-515.32989 (0.0)	-515.96572 (0.0)	-515.83466 (0.0)

5-adduct, conf2	C -0.60426 1.04978 -0.12501<br>C 0.86572 1.47382 0.00675<br>C 1.83581 0.42661 0.11209<br>C 1.51129 -0.93756 -0.00199<br>C 0.22158 -1.31116 -0.33033<br>C -0.85777 -0.33487 -0.70712<br>H 2.85679 0.72795 0.33477<br>H -0.03114 -2.36641 -0.40810<br>H -0.71905 -0.15379 -1.79203<br>O -1.48709 1.81177 0.19878<br>O 1.13885 2.67664 0.08151<br>C 2.58303 -1.96791 0.21530<br>H 2.17748 -2.98090 0.17459<br>H 3.36003 -1.87875 -0.55139<br>H 3.06690 -1.82530 1.18656<br>C -2.55275 -1.17576 0.83776<br>H -3.58046 -1.54636 0.85525<br>H -1.89753 -1.96086 1.24731<br>H -2.49990 -0.30681 1.49978<br>N -2.22065 -0.77505 -0.52311<br>H -2.41860 -1.52838 -1.17201	C_1	-515.45848 (1.5)	-515.27766 (1.6)	-515.32725 (1.7)	-515.96339 (1.5)	-515.83215 (1.6)
5-adduct, conf3	C 0.78140 0.72976 0.54751<br>C -0.30336 1.48046 -0.23623<br>C -1.56241 0.80680 -0.37401<br>C -1.78385 -0.51317 0.07275<br>C -0.73224 -1.26866 0.55336<br>C 0.68056 -0.78361 0.54792<br>H -2.37775 1.37733 -0.81236<br>H -0.90561 -2.28798 0.88886<br>H 1.22131 -1.18114 1.42084<br>O 1.69438 1.32949 1.07491<br>O -0.08928 2.62650 -0.63878<br>C -3.17262 -1.08024 0.00006<br>H -3.20348 -2.11161 0.35683<br>H -3.86434 -0.48317 0.60310<br>H -3.54071 -1.05946 -1.03095<br>C 2.77638 -1.32795 -0.61212<br>H 3.16756 -1.62664 -1.58766<br>H 3.24463 -0.37800 -0.33764<br>H 3.06987 -2.08185 0.13489<br>N 1.33553 -1.15610 -0.71629<br>H 0.89584 -1.98301 -1.10629	C_1	-515.45722 (2.3)	-515.27625 (2.5)	-515.32648 (2.1)	-515.95949 (3.9)	-515.82875 (3.7)

5-adduct, conf4	C 0.77591 0.72027 0.48885<br>C -0.36494 1.49513 -0.18395<br>C -1.61698 0.80923 -0.30305<br>C -1.80323 -0.54132 0.06398<br>C -0.73165 -1.30421 0.48162<br>C 0.67139 -0.79303 0.53417<br>H -2.45498 1.38631 -0.68694<br>H -0.87399 -2.35387 0.72396<br>H 1.16179 -1.13536 1.45470<br>O 1.75299 1.31107 0.90753<br>O -0.18968 2.66731 -0.53070<br>C -3.18143 -1.13112 -0.03091<br>H -3.18558 -2.18629 0.24944<br>H -3.87422 -0.59548 0.62638<br>H -3.56833 -1.04194 -1.05125<br>C 2.85785 -1.20321 -0.55993<br>H 3.28107 -1.69803 -1.43780<br>H 3.19357 -0.15578 -0.55321<br>H 3.25682 -1.69240 0.33435<br>N 1.41350 -1.37193 -0.59185<br>H 1.02137 -1.06120 -1.47595	C_1	-515.45845 (1.6)	-515.27729 (1.8)	-515.32710 (1.8)	-515.96159 (2.6)	-515.83024 (2.8)
5-adduct, conf5	C 0.59945 0.88873 -0.16451<br>C -0.78494 1.48263 0.13198<br>C -1.88170 0.56265 0.13179<br>C -1.71270 -0.82665 -0.00986<br>C -0.44113 -1.36933 -0.02170<br>C 0.79267 -0.56635 0.23393<br>H -2.88044 0.98865 0.18980<br>H -0.31472 -2.44252 -0.14577<br>H 0.86140 -0.49290 1.34886<br>O 1.46650 1.57177 -0.66080<br>O -0.90306 2.70437 0.26966<br>C -2.92467 -1.70657 -0.12549<br>H -3.55531 -1.38898 -0.96176<br>H -2.64817 -2.75195 -0.27686<br>H -3.53202 -1.64067 0.78332<br>C 3.21463 -0.80742 0.36354<br>H 4.04117 -1.37399 -0.07210<br>H 3.42448 0.25570 0.22920<br>H 3.17729 -1.02126 1.44457<br>N 1.98195 -1.15980 -0.33132<br>H 1.87555 -2.16867 -0.32901	C_1	-515.45802 (1.8)	-515.27743 (1.7)	-515.32719 (1.7)	-515.96233 (2.1)	-515.83150 (2.0)

5-adduct, conf6	C 0.58374 0.88685 -0.16580<br>C -0.79662 1.47711 0.14717<br>C -1.88597 0.54878 0.14729<br>C -1.70876 -0.83920 -0.00913<br>C -0.43480 -1.37775 -0.02329<br>C 0.79919 -0.57061 0.23987<br>H -2.88752 0.96757 0.21131<br>H -0.28410 -2.44489 -0.16137<br>H 0.89109 -0.52112 1.34318<br>O 1.43135 1.55255 -0.71765<br>O -0.91592 2.69687 0.30284<br>C -2.91754 -1.72251 -0.13403<br>H -3.54630 -1.40305 -0.97118<br>H -2.63611 -2.76594 -0.28959<br>H -3.52918 -1.66358 0.77246<br>C 3.22197 -0.82818 0.42363<br>H 4.06254 -1.33706 -0.05513<br>H 3.43027 0.25063 0.43315<br>H 3.16709 -1.18034 1.45910<br>N 1.99401 -1.19317 -0.27331<br>H 2.08336 -0.99233 -1.26461	C_1	-515.45997 (0.6)	-515.27927 (0.6)	-515.32940 (0.3)	-515.96311 (1.6)	-515.83254 (1.3)
5-adduct, conf7	C 0.11989 -1.25828 0.11679<br>C -1.38086 -1.13584 -0.18232<br>C -1.93354 0.18363 -0.13500<br>C -1.14567 1.33402 0.05517<br>C 0.23361 1.23144 0.05407<br>C 0.96871 -0.04490 -0.24413<br>H -3.01661 0.26436 -0.19067<br>H 0.83929 2.11779 0.22492<br>H 1.03070 -0.11363 -1.34936<br>O 0.56325 -2.27410 0.60129<br>O -2.04409 -2.16114 -0.36807<br>C -1.82137 2.66410 0.23249<br>H -1.09763 3.45766 0.42896<br>H -2.38447 2.92904 -0.66870<br>H -2.53424 2.62943 1.06227<br>C 3.27072 0.74524 -0.37323<br>H 4.25064 0.60124 0.08855<br>H 3.35666 0.48207 -1.43284<br>H 3.02186 1.81663 -0.31000<br>N 2.31363 -0.14339 0.26640<br>H 2.31623 -0.01535 1.27325	C_1	-515.45853 (1.5)	-515.27774 (1.5)	-515.32769 (1.4)	-515.96190 (2.4)	-515.83106 (2.3)

5-adduct, conf8	C -0.23818 -1.21289 -0.09564<br>C 1.27162 -1.24129 0.15361<br>C 1.94646 0.02056 0.12331<br>C 1.27984 1.24861 -0.04660<br>C -0.10159 1.28407 -0.06694<br>C -0.94969 0.08580 0.21987<br>H 3.03207 -0.00705 0.17932<br>H -0.61918 2.22410 -0.23729<br>H -1.02010 0.03330 1.33597<br>O -0.82784 -2.19584 -0.49166<br>O 1.83958 -2.32877 0.29896<br>C 2.08670 2.50690 -0.19606<br>H 1.44750 3.36992 -0.39324<br>H 2.65731 2.70481 0.71758<br>H 2.80651 2.41210 -1.01497<br>C -3.22193 0.94039 0.34491<br>H -4.19576 0.86319 -0.14436<br>H -3.33202 0.62943 1.39686<br>H -2.93207 1.99587 0.33479<br>N -2.25519 0.14144 -0.39346<br>H -2.59132 -0.81171 -0.49771	C_1	-515.46084 (0.1)	-515.28017 (0.0)	-515.32989 (0.0)	-515.96400 (1.1)	-515.83304 (1.0)
5-adduct, conf9	C -0.27907 -1.27712 0.42297<br>C 1.08348 -1.25780 -0.27578<br>C 1.75380 0.00488 -0.33821<br>C 1.18888 1.20558 0.13793<br>C -0.11182 1.23015 0.60004<br>C -1.01932 0.04047 0.58832<br>H 2.76151 0.00346 -0.74682<br>H -0.52086 2.16260 0.97943<br>H -1.56819 0.00607 1.54625<br>O -0.77512 -2.32484 0.77864<br>O 1.55956 -2.31331 -0.70410<br>C 2.02600 2.45288 0.12559<br>H 2.35680 2.68130 -0.89285<br>H 1.47145 3.31182 0.50866<br>H 2.92496 2.31968 0.73612<br>C -2.75733 1.27640 -0.63707<br>H -3.50359 1.13737 -1.42284<br>H -3.27969 1.50662 0.30519<br>H -2.14319 2.13921 -0.91001<br>N -1.93431 0.07443 -0.56503<br>H -2.54234 -0.73665 -0.48704	C_1	-515.45547 (3.4)	-515.27427 (3.7)	-515.32564 (2.7)	-515.95939 (4.0)	-515.82955 (3.2)

Part 10: 4-Methylcatechol – thiourea adducts

Table S10.1. Reduced neutral form in vacuo.

Structure	Schematic drawing	Symmetry	*E*	H_{RRHO}	G_{RRHO}	$E_{M06-2X,large}$	$G_{RRHO,M06-2X,large}$
6-adduct, conf1	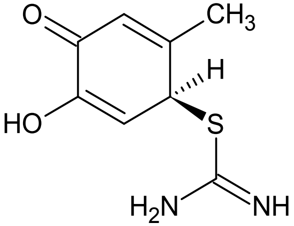<br>(1*S*)-5-hydroxy-2-methyl-4-oxocyclohexa-2,5-dien-1-yl carbamimidothioate<br><br>C 1.92642 0.60522 -0.46331<br>C 2.08099 -0.50129 0.51165<br>C 1.03575 -1.50824 0.56440<br>C -0.06552 -1.45120 -0.21653<br>C -0.24819 -0.32337 -1.19295<br>C 0.84107 0.69120 -1.25267<br>H 1.18663 -2.32434 1.26590<br>H -0.35534 -0.77716 -2.19137<br>H 0.74050 1.49903 -1.97217<br>O 2.93719 1.48559 -0.46789<br>O 3.09240 -0.50880 1.21509<br>C -1.13079 -2.49844 -0.15193<br>H -1.41254 -2.83691 -1.15512<br>H -0.79324 -3.35964 0.42884<br>H -2.03965 -2.09392 0.30933<br>S -1.93570 0.44371 -1.03688<br>C -2.02094 0.84397 0.71045<br>N -3.05501 0.41812 1.32530<br>H -3.09816 0.79142 2.27586<br>N -1.01504 1.63739 1.18847<br>H -0.20716 1.82000 0.61198<br>H -0.87496 1.68109 2.18650<br>H 3.56602 1.15115 0.20173	C_1	-968.22803 (4.8)	-968.03631 (4.7)	-968.09083 (5.0)	-968.94006 (2.9)	-968.80286 (3.1)
6-adduct, conf2	C 1.90048 0.71295 -0.47853<br>C 2.18778 -0.37182 0.49110<br>C 1.22791 -1.45632 0.59750	C_1	-968.23319 (1.5)	-968.04108 (1.7)	-968.09541 (2.2)	-968.94451 (0.1)	-968.80674 (0.7)

	C 0.08740 -1.48591 -0.12377<br>C -0.21100 -0.40231 -1.12216<br>C 0.78296 0.70294 -1.22599<br>H 1.47088 -2.24866 1.30000<br>H -0.27704 -0.89763 -2.10554<br>H 0.59058 1.49029 -1.94920<br>O 2.83466 1.67187 -0.53013<br>O 3.23031 -0.29695 1.14217<br>C -0.89318 -2.60724 0.00761<br>H -1.21417 -2.97344 -0.97368<br>H -0.46070 -3.43748 0.57007<br>H -1.79704 -2.27021 0.52718<br>S -1.95521 0.23062 -1.02249<br>C -2.18494 0.83641 0.65639<br>N -3.33465 0.84686 1.20627<br>H -4.03422 0.36818 0.64562<br>N -1.07086 1.28334 1.30416<br>H -0.31366 1.66927 0.75986<br>H -1.26296 1.73175 2.18944<br>H 3.51605 1.39498 0.11392						
6-adduct, conf3	C 1.73906 0.93446 -0.47451<br>C 2.26819 -0.14558 0.38930<br>C 1.50039 -1.37204 0.48735<br>C 0.30918 -1.52751 -0.12552<br>C -0.19900 -0.47319 -1.07234<br>C 0.59625 0.78228 -1.16111<br>H 1.90677 -2.14730 1.13079<br>H -0.20822 -0.94912 -2.06859<br>H 0.23476 1.56614 -1.81976<br>O 2.50506 2.03501 -0.51556<br>O 3.33608 0.05704 0.97184<br>C -0.51243 -2.76128 0.05426<br>H -0.87443 -3.15215 -0.90315<br>H 0.06131 -3.54077 0.56038<br>H -1.39523 -2.52808 0.66053<br>S -2.00352 -0.11401 -0.91892<br>C -2.09458 0.64782 0.69519<br>N -3.16892 1.50347 0.76851<br>H -3.44775 1.98782 -0.07115<br>H -3.29476 2.01655 1.62835<br>N -1.28066 0.30346 1.60955<br>H -1.41185 0.84584 2.46355<br>H 3.26129 1.83050 0.06931	C_1	-968.22947 (3.9)	-968.03776 (3.8)	-968.09231 (4.1)	-968.94117 (2.2)	-968.80401 (2.4)

6-adduct, conf4	C 1.92269 0.78656 -0.41573<br>C 2.25637 -0.35522 0.46747<br>C 1.30491 -1.45234 0.54283<br>C 0.14807 -1.45954 -0.15198<br>C -0.17426 -0.33809 -1.10314<br>C 0.78234 0.80684 -1.12802<br>H 1.57196 -2.27587 1.19935<br>H -0.18149 -0.79311 -2.10883<br>H 0.54874 1.64591 -1.77648<br>O 2.83568 1.76612 -0.42846<br>O 3.31974 -0.31563 1.08610<br>C -0.81332 -2.60091 -0.05723<br>H -1.14374 -2.92785 -1.04922<br>H -0.36004 -3.44822 0.46170<br>H -1.71422 -2.30191 0.48985<br>S -1.93058 0.22418 -1.03670<br>C -2.10958 0.78548 0.67004<br>N -3.44092 0.81887 1.00299<br>H -4.04836 0.10893 0.62467<br>H -3.61903 1.09282 1.95920<br>N -1.20995 1.17618 1.47601<br>H -0.29251 1.23873 1.04499<br>H 3.53737 1.46775 0.18243	C_1	-968.23253 (2.0)	-968.04039 (2.2)	-968.09446 (2.8)	-968.94372 (0.6)	-968.80564 (1.4)
6-adduct, conf5	C 1.70069 -0.92249 0.54240<br>C 2.23863 0.07256 -0.41046<br>C 1.49687 1.31490 -0.58620<br>C 0.34235 1.56870 0.06610<br>C -0.20701 0.56922 1.05085<br>C 0.56761 -0.68949 1.22506<br>H 1.92774 2.03840 -1.27333<br>H -0.24951 1.09096 2.02165<br>H 0.18688 -1.42126 1.93095<br>O 2.43711 -2.03528 0.66378<br>O 3.28878 -0.18831 -0.99648<br>C -0.39687 2.85692 -0.11012<br>H -0.57427 3.34158 0.85710<br>H 0.16348 3.54497 -0.74641<br>H -1.38228 2.68432 -0.55674<br>S -2.01269 0.23074 0.83726<br>C -2.07401 -0.73638 -0.68813<br>N -1.29763 -0.69280 -1.69160<br>H -0.57350 0.01333 -1.61177<br>N -3.20537 -1.51422 -0.68502	C_1	-968.23263 (1.9)	-968.04048 (2.1)	-968.09508 (2.4)	-968.94365 (0.7)	-968.80610 (1.1)

	H -3.48706 -1.92879 0.19003<br>H -3.29202 -2.10784 -1.49867<br>H 3.18797 -1.91327 0.05189						
6-adduct, conf6	C -1.64317 0.87670 0.61417<br>C -2.18209 -0.05931 -0.39463<br>C -1.45619 -1.30325 -0.62426<br>C -0.31579 -1.60830 0.03449<br>C 0.26332 -0.64460 1.03373<br>C -0.50963 0.60181 1.28253<br>H -1.89831 -1.99281 -1.33889<br>H 0.35442 -1.19709 1.98321<br>H -0.11599 1.29881 2.01617<br>O -2.36965 1.99039 0.78943<br>O -3.21735 0.24895 -0.98634<br>C 0.38818 -2.91168 -0.16664<br>H 0.46893 -3.45484 0.78268<br>H -0.14541 -3.54245 -0.88049<br>H 1.41141 -2.75143 -0.52294<br>S 2.06476 -0.26925 0.76561<br>C 2.11382 0.89883 -0.58959<br>N 1.24207 0.70003 -1.62887<br>H 0.46352 0.06792 -1.51976<br>H 1.07299 1.48756 -2.23689<br>N 3.05453 1.75342 -0.50171<br>H 3.14645 2.29864 -1.35991<br>H -3.12509 1.90364 0.17768	C_1	-968.22629 (5.9)	-968.03458 (5.8)	-968.08910 (6.1)	-968.93809 (4.2)	-968.80090 (4.4)
6-adduct, conf7	C -1.58333 -0.86178 -0.66240<br>C -2.12194 0.01429 0.40050<br>C -1.44565 1.28313 0.64602<br>C -0.33123 1.64406 -0.02340<br>C 0.27866 0.71472 -1.03541<br>C -0.47557 -0.52685 -1.34423<br>H -1.90873 1.94216 1.37615<br>H 0.42591 1.29062 -1.96232<br>H -0.08084 -1.18012 -2.11631<br>O -2.28862 -1.98102 -0.87590<br>O -3.12881 -0.35186 1.00651<br>C 0.31361 2.98019 0.16861<br>H 0.27450 3.55869 -0.76275<br>H -0.19108 3.55553 0.94773<br>H 1.37212 2.87492 0.43019<br>S 2.06771 0.32835 -0.65982<br>C 2.04668 -0.95081 0.60986	C_1	-968.23161 (2.5)	-968.03947 (2.8)	-968.09389 (3.1)	-968.94286 (1.2)	-968.80513 (1.7)

	N 1.10067 -0.82494 1.58506<br>H 0.76474 0.09183 1.83219<br>H 1.18861 -1.49225 2.33882<br>N 2.88746 -1.90718 0.60844<br>H 3.42760 -1.93834 -0.25148<br>H -3.02110 -1.95055 -0.23179						
6-adduct, conf8	C -1.90807 -0.68485 -0.47790<br>C -2.16338 0.39776 0.50298<br>C -1.18295 1.46326 0.60547<br>C -0.05033 1.47734 -0.13005<br>C 0.22238 0.39007 -1.13199<br>C -0.79608 -0.69367 -1.23381<br>H -1.40361 2.25602 1.31493<br>H 0.28959 0.88601 -2.11492<br>H -0.62575 -1.48050 -1.96334<br>O -2.86241 -1.62493 -0.52516<br>O -3.19930 0.33558 1.16698<br>C 0.95166 2.58048 -0.00638<br>H 1.23752 2.96740 -0.99084<br>H 0.55314 3.40197 0.59286<br>H 1.87309 2.21674 0.46222<br>S 1.95877 -0.25795 -1.03752<br>C 2.15039 -0.83455 0.64570<br>N 1.03901 -1.35909 1.24554<br>H 0.27812 -1.69844 0.67503<br>H 1.15586 -1.79824 2.14633<br>N 3.32686 -0.69660 1.11854<br>H 3.42392 -1.15803 2.02410<br>H -3.53140 -1.33598 0.12675	C_1	-968.22813 (4.7)	-968.03649 (4.6)	-968.09118 (4.8)	-968.94012 (2.9)	-968.80317 (2.9)
6-adduct, conf9	C 2.14578 -0.98118 0.19049<br>C 2.68234 0.36807 -0.09337<br>C 1.74229 1.47608 -0.13456<br>C 0.41498 1.30306 0.04197<br>C -0.14325 -0.06094 0.32968<br>C 0.83246 -1.18094 0.39010<br>H 2.16513 2.46165 -0.30981<br>H -0.72279 0.00712 1.27047<br>H 0.45055 -2.17392 0.60668<br>O 3.07369 -1.94927 0.23944<br>O 3.89784 0.48339 -0.26395<br>C -0.54344 2.44963 0.01389<br>H -1.18845 2.43396 0.89921<br>H -0.01328 3.40335 -0.03236	C_1	-968.23564 (0.0)	-968.04386 (0.0)	-968.09872 (0.1)	-968.94474 (0.0)	-968.80782 (0.0)

	H -1.20579 2.37573 -0.85635<br>S -1.48337 -0.49756 -0.86345<br>C -2.91985 -0.32030 0.18450<br>N -2.80675 0.11646 1.37687<br>H -3.69518 0.14975 1.87313<br>N -4.06685 -0.65567 -0.48963<br>H -3.99446 -1.24349 -1.30640<br>H -4.90648 -0.77933 0.05522<br>H 3.91971 -1.49314 0.06461						
6-adduct, conf10	C 1.60025 -1.21292 0.24748<br>C 2.54774 -0.14482 -0.13791<br>C 2.06132 1.22850 -0.13186<br>C 0.78519 1.54001 0.17172<br>C -0.20511 0.45516 0.49079<br>C 0.32535 -0.92977 0.56222<br>H 2.79304 1.99898 -0.35932<br>H -0.70971 0.70889 1.43143<br>H -0.36164 -1.71932 0.85275<br>O 2.12538 -2.44607 0.26359<br>O 3.70287 -0.46308 -0.42067<br>C 0.30976 2.95839 0.22504<br>H -0.12852 3.18348 1.20474<br>H 1.13317 3.65361 0.04831<br>H -0.47162 3.14562 -0.51986<br>S -1.53979 0.55583 -0.81934<br>C -2.79023 -0.54513 -0.13597<br>N -3.34163 -1.48078 -0.79730<br>H -2.89120 -1.61488 -1.69865<br>N -3.11062 -0.30821 1.18194<br>H -3.16928 0.66017 1.46168<br>H -3.88114 -0.87972 1.50564<br>H 3.05765 -2.33048 -0.00210	C_1	-968.23099 (2.9)	-968.03848 (3.4)	-968.09418 (2.9)	-968.94133 (2.1)	-968.80452 (2.1)
6-adduct, conf11	C 1.64420 -1.22650 0.17230<br>C 2.57820 -0.13785 -0.18815<br>C 2.08202 1.23040 -0.12594<br>C 0.80855 1.52257 0.20503<br>C -0.16762 0.41875 0.51550<br>C 0.37243 -0.96706 0.51721<br>H 2.80377 2.01424 -0.33889<br>H -0.61379 0.65464 1.49320<br>H -0.30435 -1.77328 0.78471<br>O 2.17597 -2.45607 0.12596<br>O 3.73024 -0.43737 -0.50144	C_1	-968.23231 (2.1)	-968.04012 (2.3)	-968.09526 (2.3)	-968.94235 (1.5)	-968.80530 (1.6)

	C 0.32280 2.93425 0.31101<br>H -0.12186 3.11972 1.29587<br>H 1.14249 3.64036 0.16272<br>H -0.45807 3.14351 -0.42837<br>S -1.55629 0.57058 -0.70343<br>C -2.81478 -0.46375 0.07166<br>N -3.69663 -0.90181 -0.87969<br>H -3.33741 -1.11914 -1.79628<br>H -4.41978 -1.51314 -0.52712<br>N -2.98220 -0.73392 1.30391<br>H -2.34609 -0.23988 1.92120<br>H 3.10311 -2.32345 -0.14969						
6-adduct, conf12	C 2.14890 -0.97829 0.18975<br>C 2.68215 0.37216 -0.09366<br>C 1.73906 1.47773 -0.13542<br>C 0.41217 1.30135 0.04049<br>C -0.14260 -0.06380 0.32834<br>C 0.83595 -1.18140 0.38883<br>H 2.15962 2.46443 -0.30999<br>H -0.72203 0.00345 1.26958<br>H 0.45607 -2.17522 0.60505<br>O 3.07908 -1.94421 0.23908<br>O 3.89731 0.49123 -0.26394<br>C -0.54852 2.44614 0.01095<br>H -1.20135 2.42367 0.89028<br>H -0.01997 3.40120 -0.02579<br>H -1.20235 2.37564 -0.86609<br>S -1.48370 -0.50855 -0.86044<br>C -2.91980 -0.31986 0.18579<br>N -4.05742 -0.74643 -0.44832<br>H -4.03605 -0.92466 -1.44043<br>H -4.94607 -0.45269 -0.07442<br>N -2.80511 0.12676 1.37472<br>H -3.69020 0.14463 1.87770<br>H 3.92423 -1.48636 0.06500	C_1	-968.23541 (0.1)	-968.04372 (0.1)	-968.09885 (0.0)	-968.94443 (0.2)	-968.80787 (0.0)
6-adduct, conf13	C 2.22409 -0.88385 0.12628<br>C 2.61112 0.52864 -0.09696<br>C 1.57040 1.53989 0.00298<br>C 0.28287 1.22427 0.25373<br>C -0.13532 -0.20924 0.42526<br>C 0.95119 -1.22468 0.38650<br>H 1.88685 2.57230 -0.11790<br>H -0.69780 -0.28674 1.36589	C_1	-968.23215 (2.2)	-968.03963 (2.7)	-968.09428 (2.9)	-968.94254 (1.4)	-968.80467 (2.0)

	H 0.68197 -2.26454 0.54573<br>O 3.23919 -1.75577 0.06062<br>O 3.79453 0.77348 -0.33214<br>C -0.77412 2.27192 0.39559<br>H -1.31287 2.15794 1.34331<br>H -0.34211 3.27399 0.35648<br>H -1.52018 2.18518 -0.40216<br>S -1.38399 -0.68600 -0.87996<br>C -2.96760 -0.33365 -0.10230<br>N -3.07391 -0.70991 1.21914<br>H -2.67055 -1.60474 1.45923<br>H -4.01036 -0.57662 1.58107<br>N -3.93831 0.21750 -0.71377<br>H -3.65904 0.55777 -1.63048<br>H 4.02784 -1.21316 -0.13375						
6-adduct, conf14	C 1.59076 -1.23131 0.20730<br>C 2.54884 -0.16694 -0.15872<br>C 2.07748 1.21100 -0.12213<br>C 0.80712 1.53089 0.19536<br>C -0.19308 0.45264 0.51154<br>C 0.32134 -0.94101 0.53622<br>H 2.81484 1.97779 -0.34384<br>H -0.64850 0.69629 1.48027<br>H -0.37319 -1.72978 0.80860<br>O 2.10014 -2.47146 0.18949<br>O 3.69920 -0.49173 -0.45399<br>C 0.34633 2.95349 0.26708<br>H -0.11547 3.16548 1.23873<br>H 1.18184 3.64206 0.12404<br>H -0.41332 3.16486 -0.49392<br>S -1.54791 0.61503 -0.76486<br>C -2.79093 -0.51045 -0.15389<br>N -2.97128 -0.51253 1.21833<br>H -2.89277 0.38036 1.68414<br>H -3.74687 -1.06913 1.55203<br>N -3.39075 -1.21170 -1.03005<br>H -4.17813 -1.72224 -0.62632<br>H 3.03057 -2.36104 -0.08447	C_1	-968.22327 (7.8)	-968.03131 (7.9)	-968.08668 (7.6)	-968.93486 (6.2)	-968.79827 (6.0)
6-adduct, conf15	C 2.24442 -0.88361 0.15535<br>C 2.62678 0.51367 -0.15199<br>C 1.58713 1.53048 -0.09169<br>C 0.30417 1.23345 0.19879<br>C -0.11038 -0.18896 0.46194	C_1	-968.23211 (2.2)	-968.04013 (2.3)	-968.09539 (2.2)	-968.94214 (1.6)	-968.80542 (1.5)

	C 0.97646 -1.20829 0.45553<br>H 1.90235 2.55330 -0.27934<br>H -0.62057 -0.21294 1.43605<br>H 0.70614 -2.23609 0.67782<br>O 3.25770 -1.75942 0.11957<br>O 3.80503 0.74654 -0.42106<br>C -0.74833 2.29119 0.29102<br>H -1.27050 2.24638 1.25338<br>H -0.31497 3.28640 0.17237<br>H -1.50849 2.15019 -0.48565<br>S -1.39016 -0.74894 -0.75270<br>C -2.94208 -0.36492 0.09427<br>N -3.19656 -0.35125 1.34156<br>H -2.42785 -0.67894 1.91817<br>N -3.92727 -0.16483 -0.83069<br>H -3.69101 0.23953 -1.72234<br>H -4.84043 0.04317 -0.45334<br>H 4.04383 -1.23062 -0.11719						
6-adduct, conf16	C 2.21672 -0.88859 0.11369<br>C 2.61340 0.52764 -0.06599<br>C 1.57536 1.54007 0.03856<br>C 0.28269 1.22419 0.26293<br>C -0.14432 -0.21156 0.39678<br>C 0.93859 -1.23044 0.34404<br>H 1.89617 2.57348 -0.05993<br>H -0.71021 -0.31190 1.33355<br>H 0.66324 -2.27336 0.46956<br>O 3.23001 -1.76268 0.04017<br>O 3.80256 0.77155 -0.27371<br>C -0.77502 2.27156 0.40055<br>H -1.29750 2.17106 1.35964<br>H -0.34445 3.27355 0.34447<br>H -1.53280 2.16919 -0.38493<br>S -1.38780 -0.62936 -0.93124<br>C -2.94658 -0.32432 -0.10632<br>N -3.06396 -0.91080 1.14043<br>H -2.62809 -1.81506 1.25451<br>H -3.96914 -0.83463 1.58454<br>N -3.79604 0.39839 -0.72126<br>H -4.70029 0.39855 -0.24467<br>H 4.02239 -1.21654 -0.12829	C_1	-968.22580 (6.2)	-968.03377 (6.3)	-968.08818 (6.7)	-968.93693 (4.9)	-968.79931 (5.4)

5-adduct, conf1	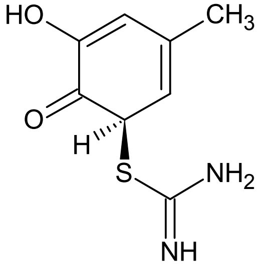<br>(1*R*)-5-hydroxy-3-methyl-6-oxocyclohexa-2,4-dien-1-yl carbamimidothioate<br><br>C 0.53856 1.17674 -0.45972<br>C 1.84325 0.96814 0.17775<br>C 2.33442 -0.27765 0.37831<br>C 1.62148 -1.46812 -0.05418<br>C 0.45433 -1.35424 -0.71943<br>C -0.12441 -0.02889 -1.09185<br>H 3.27785 -0.38914 0.90752<br>H -0.09038 -2.23220 -1.05401<br>H 0.08094 0.12029 -2.16848<br>O 0.07679 2.31578 -0.49434<br>O 2.44863 2.08308 0.61991<br>C 2.23212 -2.79904 0.27403<br>H 3.22285 -2.89891 -0.18461<br>H 2.36517 -2.91195 1.35627<br>H 1.60669 -3.62175 -0.08000<br>S -1.95608 -0.01113 -1.09400<br>C -2.33451 -0.20405 0.64451<br>N -3.21824 -1.07174 0.94236<br>H -3.47636 -1.01089 1.92933<br>N -1.62119 0.62672 1.47946<br>H -1.38027 1.54078 1.11761<br>H -1.88515 0.61177 2.45489<br>H 1.85726 2.81698 0.37166	C_1	-968.22166 (8.8)	-968.02997 (8.7)	-968.08405 (9.3)	-968.93207 (8.0)	-968.79446 (8.4)
5-adduct, conf2	C 0.54055 1.18208 -0.48647<br>C 1.82283 0.95462 0.19035<br>C 2.29724 -0.29675 0.39230<br>C 1.58743 -1.47624 -0.07551<br>C 0.43784 -1.34734 -0.76752<br>C -0.13248 -0.01567 -1.12659<br>H 3.22522 -0.42274 0.94476<br>H -0.09743 -2.21959 -1.13125	C_1	-968.22785 (4.9)	-968.03568 (5.1)	-968.08975 (5.7)	-968.93774 (4.4)	-968.79964 (5.2)

	H 0.04161 0.14260 -2.20612<br>O 0.10164 2.32785 -0.54658<br>O 2.42760 2.06041 0.65335<br>C 2.18415 -2.81462 0.24655<br>H 3.18931 -2.90814 -0.18082<br>H 2.28081 -2.94690 1.33041<br>H 1.56930 -3.62948 -0.14251<br>S -1.96606 0.02727 -1.06453<br>C -2.28893 -0.19381 0.69583<br>N -3.12828 -1.03363 1.15459<br>H -3.46884 -1.65410 0.42539<br>N -1.56399 0.63393 1.51303<br>H -1.38614 1.56952 1.17365<br>H -1.82975 0.56802 2.48703<br>H 1.85304 2.80268 0.39080						
5-adduct, conf3	C -0.41062 -1.19059 -0.51116<br>C -1.67586 -1.10214 0.23810<br>C -2.30972 0.07710 0.42105<br>C -1.75443 1.32757 -0.07009<br>C -0.60422 1.34126 -0.77200<br>C 0.11293 0.08643 -1.14969<br>H -3.24284 0.09468 0.97863<br>H -0.18514 2.27359 -1.14035<br>H -0.03041 -0.07814 -2.23153<br>O 0.09523 -2.29083 -0.68604<br>O -2.12171 -2.27316 0.71744<br>C -2.50832 2.58627 0.24614<br>H -3.52421 2.54818 -0.16462<br>H -2.60499 2.72060 1.33008<br>H -2.00691 3.46650 -0.16381<br>S 1.92724 0.21221 -1.05087<br>C 2.11626 0.23615 0.71877<br>N 3.41571 0.48866 1.09081<br>H 4.00828 1.00084 0.45552<br>H 3.58892 0.66074 2.06918<br>N 1.11505 -0.03363 1.45647<br>H 1.33056 0.00693 2.45134<br>H -1.46882 -2.93484 0.42066	C_1	-968.22592 (6.1)	-968.03445 (5.9)	-968.08838 (6.6)	-968.93708 (4.8)	-968.79954 (5.2)
5-adduct, conf4	C -0.21624 -1.16039 -0.60805<br>C -1.45911 -1.30874 0.17272<br>C -2.26145 -0.25077 0.43172<br>C -1.93672 1.08896 -0.03422<br>C -0.81836 1.31403 -0.75739	C_1	-968.22776 (5.0)	-968.03579 (5.1)	-968.08951 (5.9)	-968.93836 (4.0)	-968.80012 (4.9)

	C 0.10717 0.21368 -1.16596<br>H -3.16381 -0.40286 1.01839<br>H -0.57935 2.31033 -1.11971<br>H 0.02905 0.09112 -2.25899<br>O 0.46571 -2.15364 -0.81547<br>O -1.70116 -2.54954 0.61081<br>C -2.89574 2.18948 0.31514<br>H -3.88536 1.98852 -0.11137<br>H -3.02655 2.26809 1.40079<br>H -2.55163 3.15593 -0.06062<br>S 1.88811 0.63702 -0.99654<br>C 2.21653 0.40994 0.75235<br>N 1.12561 0.47598 1.57788<br>H 0.39279 1.13248 1.34384<br>H 1.36531 0.41953 2.55877<br>N 3.38603 0.17869 1.19867<br>H 4.05452 0.00238 0.45339<br>H -0.95903 -3.09227 0.28371						
5-adduct, conf5	C 0.35885 1.17070 -0.52647<br>C 1.63770 1.16247 0.20192<br>C 2.32831 0.01907 0.40794<br>C 1.82570 -1.26978 -0.03700<br>C 0.66976 -1.35523 -0.72399<br>C -0.10195 -0.14366 -1.13535<br>H 3.26780 0.06162 0.95347<br>H 0.28519 -2.31559 -1.05566<br>H 0.04519 0.00097 -2.21992<br>O -0.21012 2.23928 -0.70892<br>O 2.03473 2.36558 0.64577<br>C 2.63488 -2.48415 0.31378<br>H 3.64642 -2.41611 -0.10375<br>H 2.74105 -2.58015 1.40086<br>H 2.16938 -3.39730 -0.06520<br>S -1.90749 -0.35497 -1.04746<br>C -2.12270 -0.26886 0.71599<br>N -1.11421 -0.04092 1.45774<br>H -1.34945 -0.00943 2.44850<br>N -3.42530 -0.51242 1.09184<br>H -4.14890 -0.39598 0.39800<br>H -3.68728 -0.20522 2.01658<br>H 1.34678 2.98783 0.34364	C_1	-968.22620 (5.9)	-968.03470 (5.7)	-968.08883 (6.3)	-968.93739 (4.6)	-968.80002 (4.9)
5-adduct, conf6	C 0.27031 1.18865 -0.53007<br>C 1.54313 1.29160 0.20136	C_1	-968.22872 (4.3)	-968.03676 (4.5)	-968.09099 (4.9)	-968.93845 (3.9)	-968.80072 (4.5)

	C 2.31672 0.20378 0.42668<br>C 1.94159 -1.12058 -0.04302<br>C 0.80270 -1.30005 -0.74543<br>C -0.08179 -0.15884 -1.13750<br>H 3.23446 0.32114 0.99735<br>H 0.51501 -2.28424 -1.10468<br>H 0.04935 0.00078 -2.22200<br>O -0.41350 2.19465 -0.66482<br>O 1.83666 2.51673 0.65649<br>C 2.86105 -2.25747 0.29397<br>H 3.85813 -2.08880 -0.12909<br>H 2.98389 -2.35180 1.37915<br>H 2.48151 -3.20659 -0.09153<br>S -1.86765 -0.52581 -1.04778<br>C -2.12072 -0.47175 0.73885<br>N -1.28214 -0.72610 1.65943<br>H -0.39997 -1.08455 1.30169<br>N -3.43187 -0.17702 1.00949<br>H -3.88533 0.50297 0.41812<br>H -3.63664 -0.10296 1.99658<br>H 1.09678 3.08581 0.37396						
5-adduct, conf7	C 0.27832 1.19620 -0.56991<br>C 1.52622 1.25355 0.21768<br>C 2.27078 0.14723 0.44550<br>C 1.87552 -1.15858 -0.06143<br>C 0.74648 -1.30098 -0.78984<br>C -0.12193 -0.14190 -1.16432<br>H 3.18000 0.23352 1.03498<br>H 0.45390 -2.27170 -1.18126<br>H -0.02710 0.00796 -2.25296<br>O -0.34204 2.23376 -0.74987<br>O 1.83220 2.46644 0.69200<br>C 2.77537 -2.31879 0.25071<br>H 3.77258 -2.15790 -0.17512<br>H 2.90631 -2.43733 1.33272<br>H 2.37874 -3.25357 -0.15238<br>S -1.92313 -0.47329 -1.03583<br>C -2.24173 -0.39865 0.71793<br>N -1.14293 -0.47720 1.54300<br>H -0.36916 -1.05985 1.24562<br>H -1.34030 -0.51369 2.53303<br>N -3.46667 -0.23775 1.02432<br>H -3.61718 -0.29736 2.03122	C_1	-968.22142 (8.9)	-968.02993 (8.7)	-968.08362 (9.6)	-968.93287 (7.4)	-968.79507 (8.0)

	H 1.12399 3.05708 0.37129						
5-adduct, conf8	C -0.68835 -1.27847 -0.39771<br>C -1.88196 -0.85657 0.35038<br>C -2.24277 0.44742 0.41899<br>C -1.48733 1.49807 -0.24709<br>C -0.38129 1.19785 -0.95680<br>C 0.09878 -0.20450 -1.13328<br>H -3.12414 0.71699 0.99578<br>H 0.19462 1.97476 -1.45139<br>H -0.02005 -0.47498 -2.19596<br>O -0.40325 -2.46817 -0.43472<br>O -2.54786 -1.84444 0.96526<br>C -1.98435 2.90530 -0.09618<br>H -3.00780 3.00249 -0.47652<br>H -2.00189 3.19921 0.95971<br>H -1.34956 3.61185 -0.63583<br>S 1.90587 -0.40064 -0.95007<br>C 2.19748 0.23669 0.71030<br>N 1.37735 0.40293 1.66605<br>H 0.43129 0.10010 1.45993<br>N 3.54193 0.46577 0.87719<br>H 4.06498 0.81307 0.08829<br>H 3.77607 0.87507 1.77068<br>H -2.06485 -2.66312 0.74775	C_1	-968.22646 (5.8)	-968.03467 (5.8)	-968.08931 (6.0)	-968.93691 (4.9)	-968.79976 (5.1)
5-adduct, conf9	C -0.17211 0.94177 0.51951<br>C -1.42967 1.41625 -0.08274<br>C -2.46022 0.56500 -0.28690<br>C -2.36375 -0.84993 0.04853<br>C -1.21097 -1.36995 0.51821<br>C 0.02372 -0.54803 0.65941<br>H -3.39326 0.94906 -0.69132<br>H -1.13251 -2.42793 0.75292<br>H 0.55957 -0.74255 1.59337<br>O 0.67363 1.76272 0.85376<br>O -1.48394 2.73160 -0.33122<br>C -3.59705 -1.68231 -0.13996<br>H -4.42837 -1.29061 0.45775<br>H -3.91892 -1.66502 -1.18786<br>H -3.42662 -2.72231 0.14822<br>S 1.13200 -1.06794 -0.74241<br>C 2.69673 -0.32623 -0.26883<br>N 3.25712 0.43184 -1.12324<br>H 4.19303 0.70561 -0.81590	C_1	-968.21889 (10.5)	-968.02710 (10.5)	-968.08226 (10.4)	-968.92975 (9.4)	-968.79313 (9.3)

	N 3.12688 -0.67549 0.99243<br>H 2.90100 -1.60364 1.31653<br>H 4.03341 -0.33364 1.27749<br>H -0.62490 3.08960 -0.04063						
5-adduct, conf10	C -0.32093 1.02970 0.34891<br>C -1.71325 1.34194 -0.00743<br>C -2.63210 0.35915 -0.14540<br>C -2.29330 -1.04433 0.05120<br>C -1.02842 -1.41155 0.34056<br>C 0.08191 -0.42490 0.45216<br>H -3.65554 0.62396 -0.39915<br>H -0.77160 -2.45838 0.47764<br>H 0.63337 -0.54025 1.39450<br>O 0.46220 1.95342 0.53671<br>O -1.98317 2.64598 -0.16255<br>C -3.40740 -2.04112 -0.07258<br>H -4.20192 -1.83199 0.65323<br>H -3.86209 -1.99345 -1.06903<br>H -3.05209 -3.06099 0.09249<br>S 1.32471 -0.79636 -0.87292<br>C 2.86877 -0.25940 -0.10676<br>N 3.70429 0.51263 -0.67183<br>H 3.31695 0.92617 -1.51561<br>N 3.08800 -0.77799 1.14904<br>H 2.84424 -1.74903 1.28036<br>H 3.98988 -0.51903 1.52840<br>H -1.14647 3.11012 0.02617	C_1	-968.22596 (6.1)	-968.03369 (6.4)	-968.08981 (5.7)	-968.93459 (6.4)	-968.79844 (5.9)
5-adduct, conf11	C -0.45439 1.09310 0.01476<br>C -1.91540 1.24448 0.03913<br>C -2.73237 0.16461 0.04219<br>C -2.21513 -1.19408 0.06492<br>C -0.88450 -1.41529 0.09008<br>C 0.10165 -0.30259 0.16062<br>H -3.80825 0.31979 0.01222<br>H -0.48565 -2.42517 0.12048<br>H 0.58363 -0.31326 1.16720<br>O 0.24063 2.09949 -0.08087<br>O -2.35593 2.51135 -0.02272<br>C -3.21367 -2.31465 0.06316<br>H -3.86576 -2.26041 0.94288<br>H -3.86051 -2.26139 -0.82046<br>H -2.71875 -3.28879 0.06544<br>S 1.54475 -0.58173 -0.90874	C_1	-968.22955 (3.8)	-968.03812 (3.6)	-968.09276 (3.8)	-968.93622 (5.3)	-968.79942 (5.3)

	C 2.84226 -0.26634 0.28245<br>N 4.05678 -0.13110 -0.33946<br>H 4.06470 0.23834 -1.27858<br>H 4.84015 0.14341 0.23370<br>N 2.57954 -0.26937 1.52768<br>H 3.38244 -0.04501 2.11110<br>H -1.55050 3.06124 -0.04692						
5-adduct, conf12	C -0.32202 1.02215 0.14845<br>C -1.75036 1.35395 0.06953<br>C -2.68898 0.38085 0.00463<br>C -2.34209 -1.03239 0.04135<br>C -1.05470 -1.41815 0.14959<br>C 0.06230 -0.43784 0.27949<br>H -3.73406 0.66469 -0.09235<br>H -0.78739 -2.47067 0.18172<br>H 0.48555 -0.52551 1.29367<br>O 0.49943 1.93225 0.13014<br>O -2.03092 2.66390 0.00194<br>C -3.46909 -2.01908 -0.04367<br>H -4.17096 -1.88350 0.78743<br>H -4.03777 -1.88164 -0.97069<br>H -3.10215 -3.04773 -0.01717<br>S 1.43990 -0.85792 -0.85352<br>C 2.85328 -0.36279 0.17913<br>N 3.71769 0.42318 -0.52487<br>H 3.31179 1.08423 -1.16947<br>H 4.51391 0.74852 0.00647<br>N 3.09921 -0.75140 1.36552<br>H 2.45619 -1.47253 1.68035<br>H -1.16999 3.11909 0.04438	C_1	-968.22704 (5.4)	-968.03502 (5.5)	-968.09036 (5.3)	-968.93510 (6.1)	-968.79841 (5.9)
5-adduct, conf13	C -0.38734 1.06649 0.19487<br>C -1.78767 1.26225 -0.17666<br>C -2.65254 0.22001 -0.18304<br>C -2.24479 -1.12877 0.17956<br>C -0.96158 -1.38496 0.50503<br>C 0.09411 -0.33284 0.48943<br>H -3.68841 0.40150 -0.45926<br>H -0.64010 -2.38889 0.76666<br>H 0.62021 -0.30327 1.45417<br>O 0.34988 2.05565 0.24896<br>O -2.13890 2.51895 -0.49262<br>C -3.30169 -2.19329 0.17871<br>H -4.11137 -1.94314 0.87401	C_1	-968.22895 (4.2)	-968.03707 (4.3)	-968.09060 (5.2)	-968.93726 (4.7)	-968.79891 (5.6)

	H -3.75085 -2.29473 -0.81614<br>H -2.89008 -3.16324 0.46701<br>S 1.37569 -0.86228 -0.73758<br>C 2.91810 -0.29511 -0.02607<br>N 3.93356 -0.96501 -0.40840<br>H 4.80450 -0.53801 -0.09227<br>N 2.83847 0.75435 0.85114<br>H 2.10576 1.44579 0.69384<br>H 3.72216 1.11867 1.17793<br>H -1.33455 3.05693 -0.38558						
5-adduct, conf14	C -0.44762 1.08537 0.20616<br>C -1.85877 1.22337 -0.16900<br>C -2.67901 0.14733 -0.18353<br>C -2.21203 -1.18572 0.17270<br>C -0.91923 -1.39458 0.49186<br>C 0.09395 -0.29962 0.48434<br>H -3.72201 0.28463 -0.45793<br>H -0.56075 -2.38713 0.74897<br>H 0.61972 -0.25105 1.44878<br>O 0.24452 2.09819 0.28004<br>O -2.25652 2.46720 -0.47456<br>C -3.22610 -2.29096 0.17313<br>H -4.04150 -2.07383 0.87288<br>H -3.67638 -2.40588 -0.81980<br>H -2.77638 -3.24531 0.45682<br>S 1.39434 -0.74039 -0.74494<br>C 2.88648 -0.10956 0.07202<br>N 2.99825 0.89535 0.83607<br>H 2.15641 1.46938 0.86381<br>N 3.96646 -0.85811 -0.32474<br>H 3.84103 -1.85205 -0.43717<br>H 4.84138 -0.56511 0.08736<br>H -1.47404 3.03502 -0.35401	C_1	-968.23084 (3.0)	-968.03857 (3.3)	-968.09258 (3.9)	-968.93894 (3.6)	-968.80068 (4.5)
5-adduct, conf15	C -0.40147 1.07008 0.18594<br>C -1.80844 1.25086 -0.17205<br>C -2.66149 0.19903 -0.17452<br>C -2.23527 -1.14617 0.18097<br>C -0.94686 -1.39044 0.49540<br>C 0.09464 -0.32529 0.48059<br>H -3.70148 0.36899 -0.44225<br>H -0.61442 -2.39109 0.75587<br>H 0.62618 -0.28765 1.44222<br>O 0.32804 2.06344 0.22905	C_1	-968.23433 (0.8)	-968.04207 (1.1)	-968.09568 (2.0)	-968.94198 (1.7)	-968.80333 (2.9)

	O -2.17509 2.50375 -0.48297<br>C -3.28079 -2.22178 0.18679<br>H -4.08604 -1.98193 0.89075<br>H -3.73888 -2.32442 -0.80386<br>H -2.85672 -3.18839 0.46822<br>S 1.38954 -0.81873 -0.74817<br>C 2.94043 -0.27317 -0.01019<br>N 2.85269 0.76884 0.86500<br>H 2.14630 1.47830 0.68610<br>H 3.75406 1.09641 1.18517<br>N 4.04359 -0.84686 -0.29249<br>H 3.91185 -1.69105 -0.84297<br>H -1.37556 3.05037 -0.38241						
5-adduct, conf16	C 1.12713 -1.16736 0.34406<br>C 2.33952 -0.50352 -0.14716<br>C 2.39325 0.84250 -0.27819<br>C 1.26243 1.69682 0.05145<br>C 0.10084 1.16122 0.47791<br>C -0.09654 -0.30972 0.61450<br>H 3.31511 1.29943 -0.62969<br>H -0.74775 1.79368 0.72312<br>H -0.43290 -0.58132 1.62420<br>O 1.13930 -2.38076 0.51470<br>O 3.36897 -1.31995 -0.41725<br>C 1.45108 3.17696 -0.10540<br>H 2.27973 3.53414 0.51702<br>H 1.69640 3.43042 -1.14354<br>H 0.54977 3.72598 0.17685<br>S -1.38329 -0.92555 -0.55746<br>C -2.82950 -0.03367 0.04381<br>N -3.09381 0.34969 1.22894<br>H -2.41288 0.03770 1.91404<br>N -3.73081 0.12386 -0.97393<br>H -3.37900 0.28148 -1.90503<br>H -4.57404 0.61779 -0.71832<br>H 3.05502 -2.21905 -0.20969	C_1	-968.22598 (6.1)	-968.03395 (6.2)	-968.08908 (6.1)	-968.93522 (6.0)	-968.79833 (6.0)
5-adduct, conf17	C 1.14143 -1.16377 0.33947<br>C 2.31973 -0.43920 -0.14802<br>C 2.31969 0.91201 -0.23099<br>C 1.16286 1.70988 0.14686<br>C 0.02700 1.11184 0.56066<br>C -0.11678 -0.36738 0.61831<br>H 3.21913 1.41681 -0.57510	C_1	-968.22568 (6.2)	-968.03328 (6.6)	-968.08886 (6.3)	-968.93526 (6.0)	-968.79844 (5.9)

	H -0.84234 1.69793 0.84567<br>H -0.50333 -0.69805 1.58818<br>O 1.20507 -2.37978 0.48629<br>O 3.37865 -1.20457 -0.45352<br>C 1.29736 3.20142 0.05964<br>H 2.11860 3.55901 0.69161<br>H 1.52299 3.51200 -0.96735<br>H 0.37917 3.70324 0.37351<br>S -1.32459 -0.95277 -0.67430<br>C -2.80533 -0.04938 -0.19550<br>N -3.11639 -0.12009 1.14308<br>H -2.94653 -1.00628 1.59655<br>H -4.01655 0.29000 1.35850<br>N -3.52690 0.60576 -1.01335<br>H -3.08395 0.67348 -1.92569<br>H 3.10375 -2.12134 -0.27008						

Table S10.2. Reduced neutral form in water.

Structure	Schematic drawing	Symmetry	G_{PCM}	$H_{PCM,RRHO}$	$G_{PCM,RRHO}$	$G_{SMD,M06\text{-}2X,large}$	$G_{SMD,RRHO,M06\text{-}2X,large}$
6-adduct, conf1	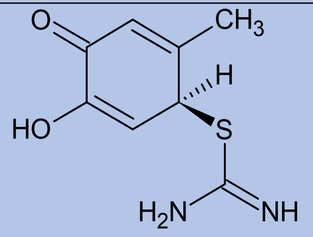<br><br>(1*S*)-5-hydroxy-2-methyl-4-oxocyclohexa-2,5-dien-1-yl carbamimidothioate<br><br>C 1.76913 0.93112 -0.46551<br>C 2.30132 -0.14526 0.39175<br>C 1.52735 -1.36153 0.51330<br>C 0.32823 -1.51900 -0.09006<br>C -0.18204 -0.46810 -1.03636<br>C 0.60956 0.78915 -1.12539<br>H 1.93774 -2.14110 1.14955<br>H -0.16199 -0.94359 -2.03299<br>H 0.23789 1.57452 -1.77683<br>O 2.54085 2.03496 -0.52842	C_1	-968.24657 (3.0)	-968.05537 (3.0)	-968.11007 (3.3)	-968.96424 (1.9)	-968.82773 (1.4)

	O 3.38584 0.05056 0.95993<br>C -0.47898 -2.76210 0.08483<br>H -0.79310 -3.17149 -0.88149<br>H 0.08797 -3.52283 0.62502<br>H -1.39335 -2.54131 0.64715<br>S -1.98671 -0.13636 -0.92566<br>C -2.15596 0.67219 0.65983<br>N -1.26454 0.52876 1.56204<br>H -1.49971 1.07485 2.39197<br>N -3.35851 1.32129 0.74251<br>H -3.77126 1.68868 -0.10260<br>H -3.53104 1.87126 1.57245<br>H 3.31036 1.83937 0.04020						
6-adduct, conf2	C 1.68750 0.92404 -0.54646<br>C 2.23660 -0.06068 0.40638<br>C 1.51169 -1.30591 0.59170<br>C 0.35540 -1.57527 -0.05560<br>C -0.20813 -0.58336 -1.03759<br>C 0.55102 0.68282 -1.21973<br>H 1.95002 -2.02728 1.27598<br>H -0.24835 -1.10385 -2.00855<br>H 0.15998 1.40494 -1.93025<br>O 2.41454 2.04899 -0.68046<br>O 3.29098 0.21743 0.98963<br>C -0.36089 -2.87408 0.11952<br>H -0.52394 -3.35780 -0.85022<br>H 0.20825 -3.55106 0.75895<br>H -1.35058 -2.71843 0.56259<br>S -2.01328 -0.25371 -0.81963<br>C -2.08208 0.74555 0.67918<br>N -1.28326 0.72756 1.67522<br>H -0.56201 0.01862 1.58519<br>N -3.21647 1.50484 0.67423<br>H -3.56268 1.85472 -0.20655<br>H -3.32875 2.12405 1.46538<br>H 3.17781 1.93852 -0.08236	C_1	-968.24931 (1.3)	-968.05773 (1.6)	-968.11230 (1.9)	-968.96682 (0.3)	-968.82981 (0.1)
6-adduct, conf3	C 1.91670 0.77331 -0.42851<br>C 2.24266 -0.34761 0.47882<br>C 1.29815 -1.44413 0.56805<br>C 0.14397 -1.46896 -0.13535<br>C -0.17732 -0.36035 -1.09947<br>C 0.77990 0.78181 -1.14578<br>H 1.56261 -2.26181 1.23286	C_1	-968.24940 (1.2)	-968.05782 (1.5)	-968.11202 (2.1)	-968.96731 (0.0)	-968.82993 (0.0)

	H -0.18847 -0.82556 -2.09977<br>H 0.55142 1.60338 -1.81817<br>O 2.83264 1.75706 -0.46327<br>O 3.30526 -0.29172 1.10915<br>C -0.80526 -2.61818 -0.03835<br>H -1.09943 -2.96928 -1.03321<br>H -0.35629 -3.44705 0.51213<br>H -1.72628 -2.31973 0.47457<br>S -1.93254 0.20240 -1.02827<br>C -2.10524 0.80718 0.66038<br>N -3.42453 0.83607 1.01014<br>H -4.04320 0.13198 0.63639<br>H -3.61433 1.12217 1.96101<br>N -1.18725 1.23652 1.43735<br>H -0.28379 1.27953 0.97430<br>H 3.53623 1.47874 0.15394						
6-adduct, conf4	C -1.63759 0.86682 0.62292<br>C -2.16147 -0.04561 -0.41181<br>C -1.44792 -1.28999 -0.64834<br>C -0.31789 -1.61273 0.02002<br>C 0.25461 -0.66455 1.03632<br>C -0.51824 0.57594 1.30514<br>H -1.88397 -1.97113 -1.37421<br>H 0.35296 -1.22890 1.97711<br>H -0.13979 1.24830 2.06932<br>O -2.36690 1.98194 0.81754<br>O -3.19257 0.28168 -1.01107<br>C 0.36811 -2.92507 -0.17558<br>H 0.40614 -3.47634 0.77133<br>H -0.15407 -3.53594 -0.91395<br>H 1.40495 -2.78401 -0.49886<br>S 2.05183 -0.27278 0.76546<br>C 2.11330 0.89777 -0.58671<br>N 1.17389 0.78758 -1.56140<br>H 0.67083 -0.07641 -1.69542<br>H 1.27262 1.37019 -2.38083<br>N 3.07711 1.73929 -0.50367<br>H 3.15485 2.28813 -1.36112<br>H -3.11245 1.91813 0.19116	C_1	-968.24568 (3.6)	-968.05438 (3.7)	-968.10959 (3.6)	-968.96357 (2.3)	-968.82747 (1.5)
6-adduct, conf5	C -1.60622 -0.87703 -0.63082<br>C -2.14856 0.03382 0.39679<br>C -1.45992 1.29318 0.62420<br>C -0.33059 1.62647 -0.03889	C_1	-968.24833 (1.9)	-968.05671 (2.2)	-968.11140 (2.5)	-968.96531 (1.3)	-968.82838 (1.0)

	C 0.26578 0.67643 -1.03868<br>C -0.48866 -0.57370 -1.31025<br>H -1.91222 1.97413 1.34020<br>H 0.38723 1.23192 -1.98147<br>H -0.09677 -1.24394 -2.06946<br>O -2.31928 -2.00281 -0.82285<br>O -3.17568 -0.30807 0.99479<br>C 0.33327 2.95176 0.14548<br>H 0.36016 3.49640 -0.80573<br>H -0.19767 3.55958 0.88013<br>H 1.37278 2.83073 0.46843<br>S 2.05989 0.29582 -0.70852<br>C 2.08105 -0.91304 0.62275<br>N 1.15962 -0.76443 1.60847<br>H 0.77185 0.14520 1.80368<br>H 1.26437 -1.38113 2.40257<br>N 2.95083 -1.85352 0.63902<br>H 3.46927 -1.89422 -0.23459<br>H -3.06762 -1.94642 -0.19897						
6-adduct, conf6	C -1.77382 -0.92527 -0.46692<br>C -2.30055 0.15022 0.39463<br>C -1.52249 1.36382 0.51784<br>C -0.32392 1.51896 -0.08706<br>C 0.18107 0.46874 -1.03685<br>C -0.61500 -0.78539 -1.12843<br>H -1.92945 2.14309 1.15665<br>H 0.16292 0.94709 -2.03213<br>H -0.24693 -1.57027 -1.78248<br>O -2.54906 -2.02654 -0.53133<br>O -3.38421 -0.04376 0.96503<br>C 0.48777 2.75891 0.08942<br>H 0.80027 3.17025 -0.87662<br>H -0.07535 3.51975 0.63344<br>H 1.40314 2.53388 0.64843<br>S 1.98408 0.12687 -0.92912<br>C 2.15688 -0.67624 0.65743<br>N 1.24950 -0.55613 1.54792<br>H 1.48789 -1.09591 2.38095<br>N 3.32088 -1.39170 0.72918<br>H 4.01330 -1.29601 0.00111<br>H 3.68925 -1.59646 1.64712<br>H -3.31651 -1.83059 0.03987	C_1	-968.24631 (3.2)	-968.05524 (3.1)	-968.11027 (3.2)	-968.96390 (2.1)	-968.82786 (1.3)

6-adduct, conf7	C -1.88796 -0.74504 -0.47402<br>C -2.21423 0.33615 0.48252<br>C -1.27732 1.43386 0.61014<br>C -0.12389 1.48899 -0.09314<br>C 0.20659 0.41586 -1.09236<br>C -0.75400 -0.71886 -1.19596<br>H -1.54432 2.22445 1.30591<br>H 0.23731 0.91565 -2.07534<br>H -0.53009 -1.50499 -1.91115<br>O -2.80437 -1.72509 -0.55112<br>O -3.27410 0.24684 1.11364<br>C 0.82280 2.63561 0.04990<br>H 1.11846 3.02523 -0.92997<br>H 0.37128 3.44183 0.63115<br>H 1.74324 2.31821 0.55243<br>S 1.96954 -0.14672 -1.02412<br>C 2.22535 -0.81573 0.61454<br>N 1.13189 -1.24544 1.29836<br>H 0.30024 -1.51053 0.78900<br>H 1.28939 -1.74321 2.16382<br>N 3.45153 -0.81130 0.98702<br>H 3.56747 -1.30630 1.87189<br>H -3.50578 -1.47462 0.08068	C_1	-968.24622 (3.2)	-968.05491 (3.3)	-968.10919 (3.8)	-968.96468 (1.6)	-968.82766 (1.4)
6-adduct, conf8	C 1.89292 -0.73536 0.47642<br>C 2.20759 0.34625 -0.48311<br>C 1.26581 1.44092 -0.60411<br>C 0.11581 1.49017 0.10470<br>C -0.20592 0.41155 1.09997<br>C 0.76327 -0.71445 1.20476<br>H 1.52748 2.23485 -1.29809<br>H -0.25535 0.90595 2.08456<br>H 0.54753 -1.50054 1.92247<br>O 2.81565 -1.70984 0.55041<br>O 3.26372 0.26201 -1.12106<br>C -0.83514 2.63455 -0.02778<br>H -1.12366 3.02098 0.95553<br>H -0.39030 3.44362 -0.61020<br>H -1.75867 2.31675 -0.52428<br>S -1.96225 -0.17940 1.01006<br>C -2.21419 -0.83239 -0.64318<br>N -1.11619 -1.26006 -1.31818<br>H -0.31345 -1.58451 -0.79878<br>H -1.30310 -1.74068 -2.18793	C_1	-968.24870 (1.7)	-968.05720 (1.9)	-968.11174 (2.2)	-968.96627 (0.7)	-968.82931 (0.4)

	N -3.39078 -0.88607 -1.14725<br>H -4.07390 -0.41157 -0.56192<br>H 3.51245 -1.45630 -0.08514						
6-adduct, conf9	C 1.78605 -1.16018 0.22490<br>C 2.62816 0.00776 -0.09914<br>C 2.00210 1.31781 -0.10245<br>C 0.68420 1.48912 0.13909<br>C -0.19652 0.30240 0.40926<br>C 0.47542 -1.02141 0.48138<br>H 2.65300 2.16829 -0.28571<br>H -0.73806 0.48741 1.34514<br>H -0.13692 -1.88439 0.72717<br>O 2.43680 -2.33941 0.24635<br>O 3.82808 -0.18550 -0.33072<br>C 0.06024 2.84660 0.17516<br>H -0.50568 2.98664 1.10306<br>H 0.81931 3.62791 0.10448<br>H -0.65046 2.97335 -0.64883<br>S -1.51170 0.24748 -0.91328<br>C -2.89695 -0.51391 -0.07410<br>N -3.53679 -1.39163 -0.75098<br>H -4.39535 -1.66352 -0.26892<br>N -3.13426 -0.08857 1.20401<br>H -2.93893 0.87712 1.42792<br>H -3.96266 -0.45355 1.65562<br>H 3.36497 -2.12743 0.03167	C_1	-968.24442 (4.3)	-968.05268 (4.7)	-968.10791 (4.6)	-968.96099 (4.0)	-968.82448 (3.4)
6-adduct, conf10	C 1.61683 -1.21095 0.23497<br>C 2.55358 -0.13628 -0.14887<br>C 2.06376 1.23054 -0.13203<br>C 0.78516 1.53516 0.17805<br>C -0.19600 0.44344 0.50055<br>C 0.34219 -0.93939 0.55794<br>H 2.78631 2.01028 -0.35673<br>H -0.68423 0.69465 1.44955<br>H -0.33871 -1.73336 0.85131<br>O 2.14656 -2.44854 0.24000<br>O 3.71281 -0.45163 -0.44327<br>C 0.30249 2.94853 0.23774<br>H -0.18207 3.14994 1.19958<br>H 1.12901 3.64933 0.10658<br>H -0.44576 3.14439 -0.53850<br>S -1.53460 0.53513 -0.80102<br>C -2.81936 -0.52569 -0.12348	C_1	-968.24759 (2.4)	-968.05571 (2.8)	-968.11167 (2.3)	-968.96401 (2.1)	-968.82810 (1.1)

	N -3.46629 -1.35455 -0.85105<br>H -3.05665 -1.42741 -1.77892<br>N -3.05882 -0.38809 1.21323<br>H -2.97522 0.53309 1.61842<br>H -3.84846 -0.92148 1.55413<br>H 3.07819 -2.33393 -0.02739						
6-adduct, conf11	C 2.13961 -0.97965 0.15976<br>C 2.67837 0.37189 -0.08386<br>C 1.74637 1.48203 -0.09394<br>C 0.41581 1.30937 0.07600<br>C -0.14761 -0.05980 0.31410<br>C 0.82523 -1.18231 0.34818<br>H 2.16866 2.47289 -0.23788<br>H -0.73061 -0.03112 1.25731<br>H 0.43766 -2.17938 0.53566<br>O 3.06405 -1.96066 0.18444<br>O 3.90064 0.48887 -0.25021<br>C -0.53098 2.46426 0.08715<br>H -1.13721 2.45432 0.99967<br>H 0.00465 3.41340 0.02482<br>H -1.22921 2.39972 -0.75489<br>S -1.48508 -0.46102 -0.88644<br>C -2.92076 -0.35526 0.18560<br>N -4.06885 -0.60818 -0.50192<br>H -4.07991 -0.52052 -1.50738<br>H -4.94715 -0.41627 -0.04242<br>N -2.77760 -0.12629 1.43439<br>H -3.67096 -0.09187 1.92305<br>H 3.91671 -1.51236 0.02600	C_1	-968.25134 (0.0)	-968.06018 (0.0)	-968.11532 (0.0)	-968.96452 (1.7)	-968.82850 (0.9)
6-adduct, conf12	C 1.65886 -1.21940 0.16351<br>C 2.58370 -0.12280 -0.18543<br>C 2.08045 1.23756 -0.11875<br>C 0.80149 1.52021 0.20790<br>C -0.16525 0.40836 0.51645<br>C 0.38404 -0.97341 0.50444<br>H 2.79282 2.03176 -0.32420<br>H -0.60352 0.63564 1.49856<br>H -0.28662 -1.78649 0.76681<br>O 2.19729 -2.45191 0.11211<br>O 3.74364 -0.41604 -0.49779<br>C 0.30705 2.92686 0.31266<br>H -0.18760 3.09272 1.27600<br>H 1.13020 3.63669 0.21264	C_1	-968.24848 (1.8)	-968.05682 (2.1)	-968.11176 (2.2)	-968.96524 (1.3)	-968.82852 (0.9)

	H -0.43493 3.14421 -0.46382<br>S -1.55511 0.54893 -0.70044<br>C -2.82697 -0.46970 0.07195<br>N -3.72857 -0.87008 -0.86904<br>H -3.40980 -1.03722 -1.81153<br>H -4.46483 -1.47876 -0.53899<br>N -2.97664 -0.74810 1.30966<br>H -2.31109 -0.27675 1.91401<br>H 3.12588 -2.31981 -0.15772						
6-adduct, conf13	C 2.12761 -0.99068 0.16093<br>C 2.68193 0.35509 -0.07969<br>C 1.76203 1.47518 -0.09358<br>C 0.42887 1.31666 0.07012<br>C -0.14980 -0.04617 0.30758<br>C 0.81037 -1.17929 0.34391<br>H 2.19539 2.46151 -0.23557<br>H -0.73407 -0.00967 1.25002<br>H 0.41142 -2.17222 0.52950<br>O 3.04140 -1.98158 0.18895<br>O 3.90612 0.45880 -0.24083<br>C -0.50599 2.48132 0.07659<br>H -1.11931 2.47549 0.98442<br>H 0.03990 3.42499 0.02039<br>H -1.19846 2.42549 -0.77078<br>S -1.49194 -0.42762 -0.89379<br>C -2.92137 -0.35526 0.18921<br>N -2.77533 -0.10056 1.43282<br>H -3.66403 -0.09563 1.93092<br>N -4.07796 -0.55414 -0.50155<br>H -4.04954 -0.96382 -1.42361<br>H -4.91882 -0.73786 0.02632<br>H 3.89952 -1.54231 0.03469	C_1	-968.25132 (0.0)	-968.06022 (0.0)	-968.11529 (0.0)	-968.96442 (1.8)	-968.82839 (1.0)
6-adduct, conf14	C 2.21956 -0.90127 0.14908<br>C 2.62846 0.48647 -0.15291<br>C 1.61783 1.52535 -0.07600<br>C 0.32900 1.25583 0.22417<br>C -0.11639 -0.15988 0.46823<br>C 0.94749 -1.20183 0.45415<br>H 1.95081 2.54419 -0.25391<br>H -0.63367 -0.18949 1.43720<br>H 0.65411 -2.22253 0.68038<br>O 3.21456 -1.80649 0.10453<br>O 3.81503 0.69415 -0.43214	C_1	-968.24844 (1.8)	-968.05692 (2.1)	-968.11192 (2.1)	-968.96500 (1.5)	-968.82848 (0.9)

	C -0.69320 2.33696 0.34963<br>H -1.14902 2.32269 1.34626<br>H -0.24789 3.31948 0.18357<br>H -1.50309 2.19570 -0.37462<br>S -1.38860 -0.66387 -0.77927<br>C -2.95572 -0.38505 0.08100<br>N -3.20270 -0.48326 1.33001<br>H -2.41357 -0.82306 1.87147<br>N -3.93764 -0.14737 -0.83121<br>H -3.70984 0.33453 -1.68746<br>H -4.86254 0.01542 -0.45786<br>H 4.01572 -1.30138 -0.13146						
6-adduct, conf15	C 2.21305 -0.88823 0.13463<br>C 2.60907 0.51511 -0.10757<br>C 1.58410 1.53677 -0.00980<br>C 0.29412 1.23652 0.25774<br>C -0.13685 -0.19134 0.43636<br>C 0.93987 -1.21685 0.40574<br>H 1.90490 2.56630 -0.14307<br>H -0.69459 -0.26499 1.37922<br>H 0.65813 -2.24961 0.58830<br>O 3.22200 -1.77760 0.07498<br>O 3.79847 0.74661 -0.35748<br>C -0.74894 2.29481 0.40556<br>H -1.22726 2.22658 1.38957<br>H -0.31802 3.29148 0.29466<br>H -1.54039 2.17026 -0.34216<br>S -1.37206 -0.65105 -0.88259<br>C -2.96232 -0.34111 -0.11847<br>N -3.08021 -0.71159 1.19203<br>H -2.57070 -1.52618 1.50443<br>H -3.99608 -0.61503 1.61047<br>N -3.84010 0.20285 -0.87422<br>H -4.75634 0.20489 -0.42239<br>H 4.01919 -1.25177 -0.12734	C_1	-968.24529 (3.8)	-968.05365 (4.1)	-968.10837 (4.4)	-968.96247 (3.0)	-968.82554 (2.8)
6-adduct, conf16	C 2.20984 -0.89154 0.13054<br>C 2.60875 0.51102 -0.11072<br>C 1.58704 1.53566 -0.00600<br>C 0.29695 1.23906 0.26410<br>C -0.13637 -0.18796 0.44304<br>C 0.93682 -1.21666 0.40708<br>H 1.91099 2.56459 -0.13626<br>H -0.69484 -0.26216 1.38548	C_1	-968.24817 (2.0)	-968.05632 (2.4)	-968.11113 (2.6)	-968.96432 (1.9)	-968.82728 (1.7)

	H 0.65261 -2.24857 0.59042<br>O 3.21554 -1.78382 0.06518<br>O 3.79743 0.74005 -0.36541<br>C -0.74172 2.30100 0.41706<br>H -1.24087 2.21449 1.38895<br>H -0.30185 3.29662 0.33613<br>H -1.51813 2.20124 -0.34986<br>S -1.37877 -0.64949 -0.87081<br>C -2.97874 -0.34730 -0.10375<br>N -3.08740 -0.69995 1.21033<br>H -2.59783 -1.52687 1.52119<br>H -4.01861 -0.60048 1.59394<br>N -3.95416 0.15815 -0.75822<br>H -3.65316 0.49652 -1.66897<br>H 4.01382 -1.26049 -0.13926						
6-adduct, conf17	C 1.58778 -1.23061 0.20630<br>C 2.54352 -0.17154 -0.17210<br>C 2.08419 1.20503 -0.12711<br>C 0.81685 1.53277 0.20417<br>C -0.18468 0.45881 0.52797<br>C 0.32373 -0.93699 0.55081<br>H 2.82016 1.97239 -0.35091<br>H -0.63634 0.71109 1.49518<br>H -0.37053 -1.72034 0.84057<br>O 2.08885 -2.48009 0.18223<br>O 3.69160 -0.50753 -0.48718<br>C 0.36602 2.95552 0.28769<br>H -0.10977 3.15373 1.25449<br>H 1.20735 3.63960 0.16240<br>H -0.38103 3.18008 -0.48215<br>S -1.53634 0.61268 -0.75023<br>C -2.80486 -0.49462 -0.15050<br>N -2.95765 -0.57218 1.20531<br>H -2.77209 0.25144 1.75923<br>H -3.74001 -1.11953 1.53934<br>N -3.44906 -1.12458 -1.05970<br>H -4.24725 -1.62149 -0.66086<br>H 3.01896 -2.38182 -0.09661	C_1	-968.24464 (4.2)	-968.05303 (4.5)	-968.10820 (4.5)	-968.96248 (3.0)	-968.82603 (2.4)
6-adduct, conf18	C 1.58186 -1.22396 0.22711<br>C 2.53607 -0.16922 -0.16825<br>C 2.07669 1.20783 -0.13925<br>C 0.81046 1.53954 0.19276<br>C -0.18957 0.46869 0.52897	C_1	-968.24760 (2.4)	-968.05578 (2.8)	-968.11189 (2.2)	-968.96421 (1.9)	-968.82851 (0.9)

	C 0.31864 -0.92600 0.57098<br>H 2.81220 1.97225 -0.37428<br>H -0.65371 0.73003 1.48749<br>H -0.37486 -1.70517 0.87378<br>O 2.08390 -2.47296 0.22001<br>O 3.68308 -0.50912 -0.48244<br>C 0.36011 2.96313 0.26340<br>H -0.10462 3.17298 1.23313<br>H 1.19987 3.64569 0.12051<br>H -0.39547 3.17847 -0.50067<br>S -1.54175 0.60353 -0.75502<br>C -2.80387 -0.51758 -0.13870<br>N -3.00192 -0.51234 1.21151<br>H -2.89995 0.36212 1.70559<br>H -3.78352 -1.07547 1.52166<br>N -3.47370 -1.27360 -0.92307<br>H -3.09419 -1.25544 -1.86637<br>H 3.01335 -2.37828 -0.06231						
5-adduct, conf1	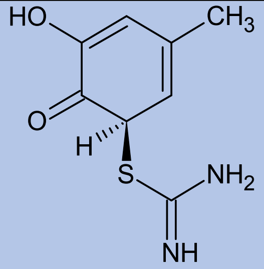<br><br>(1*R*)-5-hydroxy-3-methyl-6-oxocyclohexa-2,4-dien-1-yl carbamimidothioate<br><br>C 0.57282 1.19361 -0.46288<br>C 1.84932 0.93243 0.20318<br>C 2.29685 -0.33209 0.39152<br>C 1.56710 -1.49379 -0.08909<br>C 0.41593 -1.33195 -0.77311<br>C -0.12276 0.01721 -1.11405<br>H 3.22343 -0.48564 0.93961<br>H -0.13124 -2.18723 -1.15876<br>H 0.06642 0.17863 -2.19102<br>O 0.14508 2.34776 -0.50121<br>O 2.48710 2.01738 0.68356<br>C 2.14693 -2.84615 0.20351<br>H 3.14267 -2.94657 -0.24308	C_1	-968.23928 (7.6)	-968.04790 (7.7)	-968.10192 (8.4)	-968.95605 (7.1)	-968.81869 (7.0)

	H 2.26340 -2.99425 1.28306<br>H 1.51165 -3.64386 -0.18781<br>S -1.95339 0.09794 -1.07395<br>C -2.32062 -0.19634 0.65436<br>N -3.26339 -1.02676 0.89563<br>H -3.50221 -1.02187 1.88908<br>N -1.55153 0.50208 1.54378<br>H -1.24210 1.42542 1.27128<br>H -1.80020 0.41808 2.52108<br>H 1.93601 2.78423 0.44449						
5-adduct, conf2	C 0.34175 1.21627 -0.47674<br>C 1.63034 1.23253 0.22263<br>C 2.34207 0.09668 0.41835<br>C 1.88883 -1.19699 -0.06671<br>C 0.73068 -1.29519 -0.75418<br>C -0.08327 -0.09349 -1.11475<br>H 3.27539 0.14917 0.97351<br>H 0.38499 -2.25113 -1.13690<br>H 0.07428 0.08442 -2.19361<br>O -0.30121 2.25857 -0.56500<br>O 2.01100 2.43008 0.70294<br>C 2.74884 -2.38893 0.23136<br>H 3.74343 -2.26789 -0.21241<br>H 2.89132 -2.50519 1.31159<br>H 2.30529 -3.30684 -0.16048<br>S -1.88604 -0.35906 -1.06418<br>C -2.17791 -0.44130 0.71239<br>N -1.33210 -0.71866 1.62845<br>H -0.42980 -1.00234 1.25221<br>N -3.49918 -0.23476 0.98073<br>H -4.02512 0.39151 0.38970<br>H -3.74670 -0.22050 1.96059<br>H 1.31628 3.06109 0.44082	C_1	-968.24428 (4.4)	-968.05304 (4.5)	-968.10747 (4.9)	-968.96007 (4.5)	-968.82326 (4.2)
5-adduct, conf3	C -0.45628 -1.19747 -0.47814<br>C -1.73175 -1.06739 0.23544<br>C -2.32791 0.13396 0.41026<br>C -1.73171 1.36792 -0.07469<br>C -0.56923 1.33684 -0.75701<br>C 0.10519 0.05514 -1.12115<br>H -3.26966 0.18371 0.95184<br>H -0.11759 2.24999 -1.13451<br>H -0.06525 -0.10735 -2.20040<br>O 0.03361 -2.31583 -0.61873	C_1	-968.24345 (5.0)	-968.05239 (4.9)	-968.10639 (5.6)	-968.95996 (4.6)	-968.82290 (4.4)

	O -2.23461 -2.22116 0.71828<br>C -2.45662 2.64877 0.21932<br>H -3.46165 2.63638 -0.21791<br>H -2.57974 2.78857 1.29949<br>H -1.91856 3.51147 -0.18044<br>S 1.92171 0.12875 -1.06055<br>C 2.17040 0.21727 0.70591<br>N 3.46915 0.50825 1.01785<br>H 4.02484 1.03254 0.35774<br>H 3.68311 0.68939 1.98822<br>N 1.20189 -0.05781 1.49131<br>H 1.47011 0.04606 2.47008<br>H -1.61465 -2.91811 0.43447						
5-adduct, conf4	C -0.27459 -1.19897 -0.54124<br>C -1.54147 -1.26569 0.19698<br>C -2.28837 -0.15891 0.41912<br>C -1.89242 1.15299 -0.06945<br>C -0.75409 1.29878 -0.78133<br>C 0.10905 0.13603 -1.14940<br>H -3.20646 -0.24758 0.99452<br>H -0.45892 2.26840 -1.17215<br>H 0.00202 -0.02159 -2.23634<br>O 0.38895 -2.22305 -0.67576<br>O -1.86766 -2.48155 0.66970<br>C -2.79612 2.30813 0.24378<br>H -3.79136 2.14525 -0.18492<br>H -2.92742 2.41673 1.32623<br>H -2.39773 3.24439 -0.15310<br>S 1.90946 0.46949 -1.03032<br>C 2.25321 0.41535 0.72841<br>N 1.18733 0.56907 1.56133<br>H 0.42124 1.16074 1.26504<br>H 1.42305 0.62957 2.54317<br>N 3.44070 0.21676 1.16421<br>H 4.08546 -0.01364 0.41190<br>H -1.15895 -3.08436 0.37938	C_1	-968.24393 (4.6)	-968.05260 (4.8)	-968.10657 (5.5)	-968.95985 (4.7)	-968.82249 (4.7)
5-adduct, conf5	C 0.42706 1.18946 -0.48768<br>C 1.70825 1.10153 0.22178<br>C 2.33688 -0.08145 0.40826<br>C 1.77097 -1.33636 -0.05798<br>C 0.60534 -1.34388 -0.73572<br>C -0.10103 -0.08443 -1.11674<br>H 3.28175 -0.09960 0.94641	C_1	-968.24334 (5.0)	-968.05227 (5.0)	-968.10627 (5.7)	-968.95994 (4.6)	-968.82287 (4.4)

	H 0.17566 -2.27323 -1.09902<br>H 0.06926 0.06932 -2.19741<br>O -0.09387 2.29259 -0.63723<br>O 2.18202 2.27379 0.68957<br>C 2.52824 -2.59476 0.25108<br>H 3.53231 -2.56289 -0.18736<br>H 2.65558 -2.71799 1.33280<br>H 2.01152 -3.47556 -0.13723<br>S -1.91474 -0.20482 -1.06165<br>C -2.17246 -0.23519 0.70442<br>N -1.19271 0.01292 1.48538<br>H -1.46935 -0.04698 2.46535<br>N -3.45607 -0.57068 1.03330<br>H -4.17409 -0.54465 0.32423<br>H -3.77245 -0.33816 1.96388<br>H 1.54243 2.95064 0.40077						
5-adduct, conf6	C 0.29259 1.20774 -0.52898<br>C 1.55934 1.24891 0.21206<br>C 2.28891 0.12912 0.42688<br>C 1.87313 -1.17271 -0.07211<br>C 0.73384 -1.29477 -0.78729<br>C -0.11066 -0.11582 -1.14969<br>H 3.20720 0.19917 1.00452<br>H 0.42395 -2.25688 -1.18531<br>H 0.01156 0.04995 -2.23398<br>O -0.35389 2.24379 -0.65493<br>O 1.90305 2.45576 0.69502<br>C 2.75717 -2.34472 0.23457<br>H 3.75566 -2.19504 -0.19134<br>H 2.88470 -2.46326 1.31641<br>H 2.34417 -3.27134 -0.16989<br>S -1.91578 -0.41975 -1.05507<br>C -2.26301 -0.40638 0.69709<br>N -1.20353 -0.57657 1.53799<br>H -0.41632 -1.13191 1.22379<br>H -1.41417 -0.66063 2.52342<br>N -3.49278 -0.19821 0.98787<br>H -3.65581 -0.30690 1.98918<br>H 1.20441 3.07150 0.40718	C_1	-968.24149 (6.2)	-968.05033 (6.2)	-968.10417 (7.0)	-968.95823 (5.7)	-968.82091 (5.7)
5-adduct, conf7	C -0.58306 -1.19527 -0.47468<br>C -1.84995 -0.91094 0.20104<br>C -2.27629 0.36093 0.38701<br>C -1.53121 1.50911 -0.10353	C_1	-968.24218 (5.7)	-968.05062 (6.0)	-968.10491 (6.5)	-968.95803 (5.8)	-968.82076 (5.8)

	C -0.38598 1.32732 -0.79230<br>C 0.13389 -0.03066 -1.12520<br>H -3.19741 0.53164 0.93916<br>H 0.17168 2.17275 -1.18468<br>H -0.03680 -0.19377 -2.20440<br>O -0.17930 -2.35716 -0.52205<br>O -2.50262 -1.98425 0.68726<br>C -2.09002 2.87135 0.18363<br>H -3.08700 2.98299 -0.25759<br>H -2.19792 3.02790 1.26286<br>H -1.44596 3.65745 -0.21673<br>S 1.96332 -0.14301 -1.04441<br>C 2.29220 0.17768 0.69766<br>N 1.51610 -0.52664 1.57185<br>H 1.24905 -1.46557 1.31062<br>H 1.76665 -0.41265 2.54584<br>N 3.19297 0.99716 1.08587<br>H 3.56583 1.52860 0.30332<br>H -1.96687 -2.76081 0.44465						
5-adduct, conf8	C -0.27545 1.00813 0.39900<br>C -1.61762 1.37265 -0.06403<br>C -2.57715 0.43219 -0.22243<br>C -2.33292 -0.97802 0.05403<br>C -1.10730 -1.39538 0.43122<br>C 0.04653 -0.46004 0.55613<br>H -3.56843 0.73588 -0.55016<br>H -0.91207 -2.44377 0.63819<br>H 0.55775 -0.59079 1.51618<br>O 0.54334 1.89857 0.62044<br>O -1.82001 2.68488 -0.28618<br>C -3.49545 -1.91505 -0.08338<br>H -4.31099 -1.62359 0.58820<br>H -3.89591 -1.88729 -1.10321<br>H -3.20807 -2.94325 0.14737<br>S 1.25348 -0.92622 -0.77251<br>C 2.82137 -0.29737 -0.17067<br>N 3.54348 0.30653 -1.03788<br>H 4.47739 0.48836 -0.66492<br>N 3.09799 -0.54306 1.14277<br>H 2.72214 -1.37845 1.56710<br>H 4.01579 -0.28126 1.47657<br>H -0.98217 3.12923 -0.06277	C_1	-968.23884 (7.8)	-968.04741 (8.0)	-968.10288 (7.8)	-968.95551 (7.4)	-968.81954 (6.5)

5-adduct, conf9	C -0.26331 0.99819 0.41872<br>C -1.59889 1.37638 -0.05140<br>C -2.56480 0.44437 -0.22152<br>C -2.33363 -0.96933 0.04781<br>C -1.11340 -1.39908 0.42935<br>C 0.04656 -0.47367 0.56336<br>H -3.55169 0.75804 -0.55304<br>H -0.92770 -2.45006 0.63165<br>H 0.56575 -0.61970 1.51694<br>O 0.56113 1.87957 0.65464<br>O -1.79030 2.69158 -0.26560<br>C -3.50289 -1.89605 -0.10143<br>H -4.32051 -1.60065 0.56585<br>H -3.89613 -1.86053 -1.12383<br>H -3.22563 -2.92758 0.12674<br>S 1.25017 -0.93618 -0.77229<br>C 2.82405 -0.29359 -0.16726<br>N 3.61435 0.37738 -0.91542<br>H 3.16487 0.62574 -1.79284<br>N 3.12327 -0.60006 1.12608<br>H 2.79758 -1.48005 1.49762<br>H 4.05012 -0.32906 1.42727<br>H -0.95158 3.12901 -0.03240	C_1	-968.24189 (5.9)	-968.05015 (6.3)	-968.10515 (6.4)	-968.95727 (6.3)	-968.82052 (5.9)
5-adduct, conf10	C -0.53559 1.12528 -0.00127<br>C -1.99791 1.17091 0.04544<br>C -2.73776 0.03582 0.05584<br>C -2.13114 -1.28455 0.06546<br>C -0.78686 -1.40775 0.06989<br>C 0.11656 -0.22685 0.12835<br>H -3.82250 0.11410 0.04540<br>H -0.31586 -2.38636 0.09138<br>H 0.60705 -0.20088 1.13352<br>O 0.09028 2.18199 -0.09437<br>O -2.53979 2.40431 -0.00027<br>C -3.04960 -2.47125 0.07415<br>H -3.69142 -2.46153 0.96250<br>H -3.71152 -2.45846 -0.79929<br>H -2.48769 -3.40803 0.06657<br>S 1.57505 -0.40255 -0.94016<br>C 2.86830 -0.21635 0.29089<br>N 4.10499 -0.25905 -0.27320<br>H 4.21396 -0.06902 -1.25843<br>H 4.90149 -0.03002 0.30335	C_1	-968.24555 (3.6)	-968.05481 (3.4)	-968.10952 (3.6)	-968.95640 (6.8)	-968.82037 (6.0)

	N 2.55809 -0.12747 1.52723<br>H 3.37155 -0.01865 2.12981<br>H -1.78737 3.02348 -0.02581						
5-adduct, conf11	C -0.37358 1.05069 0.18250<br>C -1.79722 1.32464 -0.02064<br>C -2.69927 0.31810 -0.09657<br>C -2.31936 -1.07942 0.05190<br>C -1.03046 -1.41489 0.26083<br>C 0.05017 -0.39137 0.37810<br>H -3.74417 0.55850 -0.27759<br>H -0.73499 -2.45254 0.38597<br>H 0.45509 -0.44042 1.40042<br>O 0.42047 1.98844 0.20189<br>O -2.12338 2.62207 -0.17292<br>C -3.41313 -2.10246 -0.02817<br>H -4.16329 -1.93134 0.75218<br>H -3.93259 -2.03929 -0.99107<br>H -3.02007 -3.11500 0.08680<br>S 1.42462 -0.82949 -0.74747<br>C 2.89025 -0.29117 0.16788<br>N 3.88029 0.02653 -0.71114<br>H 3.63251 0.43748 -1.59833<br>H 4.75203 0.33668 -0.30450<br>N 3.08018 -0.29420 1.42990<br>H 2.30946 -0.70008 1.95222<br>H -1.29232 3.12274 -0.08479	C_1	-968.24213 (5.8)	-968.05089 (5.9)	-968.10658 (5.5)	-968.95685 (6.6)	-968.82130 (5.4)
5-adduct, conf12	C -0.45664 1.08277 0.15541<br>C -1.88202 1.22008 -0.13380<br>C -2.69762 0.13928 -0.13701<br>C -2.21503 -1.20074 0.16514<br>C -0.90917 -1.40781 0.42940<br>C 0.09019 -0.29990 0.43406<br>H -3.75195 0.27521 -0.36538<br>H -0.53526 -2.40053 0.66219<br>H 0.57858 -0.25135 1.41902<br>O 0.25184 2.09123 0.16962<br>O -2.30839 2.46769 -0.40730<br>C -3.22450 -2.30958 0.17818<br>H -4.00991 -2.11179 0.91638<br>H -3.71653 -2.39663 -0.79722<br>H -2.75924 -3.26835 0.41731<br>S 1.43226 -0.73147 -0.74857<br>C 2.90131 -0.10310 0.10037	C_1	-968.24527 (3.8)	-968.05374 (4.1)	-968.10814 (4.5)	-968.95849 (5.5)	-968.82135 (5.4)

	N 2.97331 0.91131 0.86934<br>H 2.10652 1.44867 0.86897<br>N 3.99322 -0.83383 -0.26689<br>H 3.88368 -1.82056 -0.44607<br>H 4.86650 -0.55895 0.16146<br>H -1.53089 3.04818 -0.32583						
5-adduct, conf13	C -0.39671 1.06387 0.16494<br>C -1.80689 1.26046 -0.15177<br>C -2.66889 0.21545 -0.15511<br>C -2.25146 -1.13953 0.17235<br>C -0.95982 -1.39798 0.46156<br>C 0.08752 -0.33666 0.45809<br>H -3.71250 0.39536 -0.40184<br>H -0.63270 -2.40246 0.71357<br>H 0.58617 -0.30697 1.43794<br>O 0.35408 2.04513 0.18242<br>O -2.17682 2.52160 -0.44553<br>C -3.30773 -2.20392 0.18321<br>H -4.09562 -1.96323 0.90574<br>H -3.78710 -2.28348 -0.79910<br>H -2.88728 -3.17811 0.44213<br>S 1.39870 -0.84971 -0.73908<br>C 2.93273 -0.28509 -0.00642<br>N 2.85616 0.77358 0.84113<br>H 2.10292 1.44612 0.70590<br>H 3.72903 1.14064 1.19534<br>N 3.95651 -0.97119 -0.36144<br>H 4.81702 -0.53439 -0.02981<br>H -1.37842 3.07160 -0.35863	C_1	-968.24442 (4.3)	-968.05308 (4.5)	-968.10699 (5.2)	-968.95729 (6.3)	-968.81985 (6.3)
5-adduct, conf14	C -0.40338 1.06666 0.16056<br>C -1.81551 1.25317 -0.15449<br>C -2.67039 0.20232 -0.15539<br>C -2.24348 -1.14945 0.17320<br>C -0.95002 -1.39945 0.46220<br>C 0.08830 -0.33007 0.46038<br>H -3.71532 0.37466 -0.40181<br>H -0.61630 -2.40119 0.71634<br>H 0.58728 -0.29405 1.43974<br>O 0.34212 2.05151 0.17201<br>O -2.19387 2.51112 -0.45036<br>C -3.29280 -2.22056 0.18627<br>H -4.08085 -1.98433 0.91012<br>H -3.77348 -2.30342 -0.79511	C_1	-968.24690 (2.8)	-968.05536 (3.1)	-968.10931 (3.8)	-968.95896 (5.2)	-968.82137 (5.4)

	H -2.86588 -3.19197 0.44499<br>S 1.40945 -0.82869 -0.73417<br>C 2.94808 -0.26259 0.00669<br>N 2.85791 0.79163 0.85480<br>H 2.11637 1.47132 0.70199<br>H 3.74184 1.14611 1.19433<br>N 4.05538 -0.84715 -0.26893<br>H 3.91015 -1.69706 -0.80819<br>H -1.39873 3.06638 -0.36649						
5-adduct, conf15	C -0.54438 1.12707 0.00010<br>C -2.00717 1.16154 0.04268<br>C -2.73819 0.02084 0.05398<br>C -2.12127 -1.29479 0.06829<br>C -0.77611 -1.40770 0.07581<br>C 0.11830 -0.21987 0.13179<br>H -3.82348 0.09059 0.04079<br>H -0.29770 -2.38265 0.10043<br>H 0.61165 -0.18747 1.13543<br>O 0.07343 2.18851 -0.09234<br>O -2.55812 2.39070 -0.00692<br>C -3.03069 -2.48839 0.07784<br>H -3.67517 -2.48096 0.96428<br>H -3.69014 -2.48306 -0.79755<br>H -2.46169 -3.42091 0.07453<br>S 1.57557 -0.38247 -0.94066<br>C 2.87107 -0.21078 0.28949<br>N 2.56187 -0.09946 1.52455<br>H 3.37745 -0.00479 2.12677<br>N 4.10539 -0.18783 -0.27905<br>H 4.22602 -0.46294 -1.24234<br>H 4.91137 -0.31377 0.31541<br>H -1.81018 3.01534 -0.03157	C_1	-968.24546 (3.7)	-968.05480 (3.4)	-968.10980 (3.5)	-968.95633 (6.9)	-968.82068 (5.8)
5-adduct, conf16	C 1.08062 -1.18131 0.31940<br>C 2.34198 -0.57601 -0.10343<br>C 2.46183 0.76542 -0.24509<br>C 1.35911 1.67739 0.01838<br>C 0.15881 1.19699 0.40174<br>C -0.10164 -0.26095 0.56676<br>H 3.41728 1.17802 -0.56016<br>H -0.66895 1.86965 0.60715<br>H -0.43455 -0.48923 1.58902<br>O 1.01476 -2.40048 0.46364<br>O 3.35342 -1.43544 -0.32923	C_1	-968.24149 (6.2)	-968.05008 (6.4)	-968.10558 (6.1)	-968.95710 (6.4)	-968.82118 (5.5)

	C 1.62209 3.14400 -0.15274<br>H 2.43371 3.47306 0.50593<br>H 1.93399 3.36459 -1.18005<br>H 0.73267 3.73653 0.07276<br>S -1.42497 -0.84442 -0.57529<br>C -2.86990 0.01783 0.07205<br>N -3.07463 0.43941 1.26019<br>H -2.33021 0.20119 1.90760<br>N -3.82959 0.09230 -0.89365<br>H -3.54685 0.18158 -1.85796<br>H -4.67272 0.58611 -0.63526<br>H 3.00189 -2.32547 -0.14778						
5-adduct, conf17	C 1.13093 -1.16059 0.34741<br>C 2.32835 -0.47362 -0.13189<br>C 2.35727 0.87496 -0.25460<br>C 1.21453 1.71012 0.08101<br>C 0.06376 1.14637 0.50311<br>C -0.10452 -0.32742 0.62345<br>H 3.26953 1.35272 -0.60387<br>H -0.79443 1.75819 0.76582<br>H -0.45302 -0.60884 1.62389<br>O 1.15319 -2.38207 0.49441<br>O 3.38225 -1.26395 -0.41141<br>C 1.38083 3.19437 -0.05706<br>H 2.20378 3.55464 0.57046<br>H 1.62585 3.46231 -1.09129<br>H 0.46987 3.72451 0.22959<br>S -1.34726 -0.95228 -0.61069<br>C -2.83404 -0.05800 -0.17912<br>N -3.04784 0.15995 1.15196<br>H -2.69924 -0.51653 1.81533<br>H -3.93794 0.56641 1.40810<br>N -3.56222 0.29556 -1.17060<br>H -4.45978 0.66181 -0.84898<br>H 3.09581 -2.17618 -0.22560	C_1	-968.23788 (8.4)	-968.04641 (8.7)	-968.10161 (8.6)	-968.95436 (8.1)	-968.81809 (7.4)
5-adduct, conf18	C 1.12621 -1.16569 0.33664<br>C 2.32682 -0.47460 -0.12973<br>C 2.35706 0.87499 -0.23940<br>C 1.21302 1.70788 0.09856<br>C 0.05951 1.14078 0.50821<br>C -0.10910 -0.33339 0.61521<br>H 3.27133 1.35558 -0.57938<br>H -0.79952 1.75056 0.77336	C_1	-968.24076 (6.6)	-968.04903 (7.0)	-968.10431 (6.9)	-968.95604 (7.1)	-968.81960 (6.5)

	H -0.46632 -0.62137 1.61041<br>O 1.14721 -2.38837 0.47222<br>O 3.38138 -1.26283 -0.41213<br>C 1.38201 3.19329 -0.02149<br>H 2.20020 3.54525 0.61690<br>H 1.63610 3.47250 -1.05051<br>H 0.46938 3.72128 0.26373<br>S -1.35759 -0.94683 -0.62060<br>C -2.84381 -0.04198 -0.16742<br>N -3.07891 0.08523 1.16991<br>H -2.79703 -0.66770 1.78026<br>H -3.97205 0.50004 1.40271<br>N -3.63327 0.44193 -1.04930<br>H -3.22849 0.38165 -1.98016<br>H 3.09397 -2.17666 -0.23604						
5-adduct, conf19	C 0.54054 -1.12670 -0.00373<br>C 2.00300 -1.16483 0.04623<br>C 2.73714 -0.02603 0.05790<br>C 2.12378 1.29113 0.06571<br>C 0.77878 1.40726 0.06690<br>C -0.11845 0.22186 0.12397<br>H 3.82228 -0.09889 0.04995<br>H 0.30254 2.38338 0.08672<br>H -0.61181 0.19323 1.12872<br>O -0.07939 -2.18699 -0.09747<br>O 2.55105 -2.39561 0.00216<br>C 3.03596 2.48266 0.07586<br>H 3.67639 2.47649 0.96523<br>H 3.69936 2.47335 -0.79653<br>H 2.46910 3.41646 0.06720<br>S -1.57777 0.38765 -0.94424<br>C -2.86841 0.21418 0.29294<br>N -2.55043 0.11887 1.52844<br>H -3.36108 0.02014 2.13637<br>N -4.10106 0.21478 -0.26488<br>H -4.23772 0.29119 -1.26019<br>H -4.92302 0.18852 0.31804<br>H 1.80143 -3.01818 -0.02480	C_1	-968.24537 (3.7)	saddle point			

Table10.3. Reduced zwitterionic form in water.

Structural[a]	Schematic drawing	Symmetry	G_{PCM}	$H_{PCM,RRHO}$	$G_{PCM,RRHO}$	$G_{SMD,M06-2X,large}$	$G_{SMD,RRHO,M06-2X,large}$
6-adduct, conf1	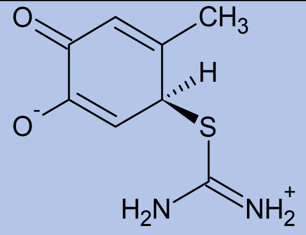 (3*S*)-3-{[amino(iminio)methyl]sulfanyl}-4-methyl-6-oxocyclohexa-1,4-dien-1-olate<br><br>C 1.93926 -0.85299 0.54304<br>C 2.26787 0.18340 -0.52500<br>C 1.38603 1.34278 -0.68302<br>C 0.25481 1.53642 0.02285<br>C -0.12637 0.53496 1.07434<br>C 0.73623 -0.66241 1.21427<br>H 1.70557 2.07893 -1.41830<br>H -0.19934 1.08130 2.02855<br>H 0.48041 -1.35121 2.01713<br>O 2.75324 -1.80732 0.72191<br>O 3.26573 0.06663 -1.23607<br>C -0.59135 2.75742 -0.15840<br>H -0.83175 3.21571 0.80746<br>H -0.07596 3.49474 -0.77738<br>H -1.54557 2.51318 -0.63809<br>S -1.97306 0.10858 0.94903<br>C -2.07980 -0.86588 -0.49781<br>N -3.26810 -0.94119 -1.07698<br>H -4.02909 -0.35381 -0.76998<br>H -3.44754 -1.58921 -1.83175<br>N -1.03440 -1.51655 -0.95461<br>H -0.17095 -1.49514 -0.39321<br>H -1.07124 -2.04433 -1.81614	C_1	-968.23389 (1.0)	-968.04291 (0.9)	-968.09730 (0.6)	-968.95463 (0.0)	-968.81804 (0.0)
6-adduct, conf2	C 2.39276 -0.90985 0.21957<br>C 2.61043 0.49947 -0.31353<br>C 1.54843 1.49793 -0.14004	C_1	-968.22672 (5.5)	-968.03521 (5.8)	-968.09116 (4.4)	-968.95245 (1.4)	-968.81689 (0.7)

	C 0.32823 1.20733 0.34834<br>C 0.01898 -0.20987 0.73201<br>C 1.13227 -1.16800 0.73353<br>H 1.80014 2.51561 -0.43231<br>H -0.55033 -0.23549 1.66670<br>H 0.92616 -2.15725 1.13575<br>O 3.37250 -1.71615 0.18366<br>O 3.66710 0.81556 -0.86033<br>C -0.72537 2.25235 0.53417<br>H -1.10044 2.25258 1.56382<br>H -0.33061 3.24511 0.30838<br>H -1.58656 2.08065 -0.12198<br>S -1.21135 -0.89227 -0.59601<br>C -2.82534 -0.35900 -0.18040<br>N -3.22519 -0.23274 1.07279<br>H -2.66865 -0.58480 1.83576<br>H -4.14242 0.12753 1.30059<br>N -3.65944 -0.12819 -1.17991<br>H -3.32099 -0.10007 -2.13023<br>H -4.65125 0.00160 -1.02901						
5-adduct, conf1	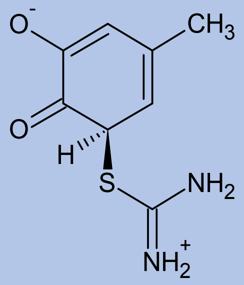<br>(5*R*)-5-{[amino(iminio)methyl]sulfanyl}-3-methyl-6-oxocyclohexa-1,3-dien-1-olate<br><br>C 0.41232 1.30664 -0.33326<br>C 1.80348 1.29579 0.28034<br>C 2.39887 0.03842 0.40056<br>C 1.84777 -1.18365 -0.11153<br>C 0.66007 -1.22583 -0.77914<br>C -0.04891 0.05135 -1.08808<br>H 3.35081 -0.01259 0.92633<br>H 0.30226 -2.13541 -1.25259<br>H 0.14171 0.31335 -2.14434	C_1	-968.23173 (2.4)	-968.04095 (2.2)	-968.09520 (1.9)	-968.95044 (2.6)	-968.81391 (2.6)

	O -0.30593 2.29070 -0.26392<br>O 2.26260 2.39222 0.71033<br>C 2.64488 -2.44185 0.09404<br>H 3.61699 -2.36394 -0.40599<br>H 2.84691 -2.60319 1.15894<br>H 2.12503 -3.31953 -0.29797<br>S -1.87965 -0.09999 -1.11249<br>C -2.21888 -0.42949 0.56879<br>N -1.28164 -0.87844 1.37671<br>H -0.39897 -1.21239 0.97174<br>H -1.45968 -1.02527 2.36131<br>N -3.46065 -0.22089 0.97445<br>H -4.12777 0.24452 0.37715<br>H -3.77786 -0.52202 1.88555						
5-adduct, conf2	C -0.64642 -1.42652 -0.43497<br>C -1.65398 -1.03394 0.63493<br>C -2.01676 0.31581 0.64026<br>C -1.58109 1.29536 -0.33211<br>C -0.60651 1.02734 -1.23636<br>C 0.01367 -0.31671 -1.27332<br>H -2.75003 0.62891 1.38147<br>H -0.26997 1.76599 -1.95716<br>H 0.05454 -0.70861 -2.29876<br>O -0.33782 -2.58802 -0.63842<br>O -2.06221 -1.93545 1.42024<br>C -2.24390 2.64264 -0.28841<br>H -3.32135 2.54882 -0.46639<br>H -2.12541 3.09848 0.70164<br>H -1.82669 3.32270 -1.03517<br>S 1.85062 -0.31101 -0.94593<br>C 2.06607 0.42656 0.62264<br>N 1.07787 0.72296 1.43861<br>H 0.09517 0.55832 1.21110<br>H 1.26706 1.15554 2.33330<br>N 3.32617 0.66383 0.96332<br>H 4.08696 0.45071 0.33594<br>H 3.56504 1.05233 1.86477	C_1	-968.22975<br>(3.6)	-968.03894<br>(3.4)	-968.09344<br>(3.0)	-968.94947<br>(3.2)	-968.81315<br>(3.1)
5-adduct, conf3	C -0.41904 1.11359 0.26368<br>C -1.80361 1.39138 -0.26249<br>C -2.65594 0.29020 -0.28684<br>C -2.30822 -1.03637 0.15792<br>C -1.06112 -1.35098 0.58878<br>C 0.00290 -0.31602 0.60784	C_1	-968.23549<br>(0.0)	-968.04439<br>(0.0)	-968.09821<br>(0.0)	-968.95176<br>(1.8)	-968.81448<br>(2.2)

	H -3.66968 0.45361 -0.64883<br>H -0.80452 -2.34772 0.93377<br>H 0.52575 -0.28739 1.57239<br>O 0.39727 2.02459 0.41416<br>O -2.07280 2.57565 -0.62874<br>C -3.39789 -2.07111 0.14380<br>H -4.23460 -1.76140 0.78058<br>H -3.79911 -2.19307 -0.86908<br>H -3.03846 -3.04262 0.49221<br>S 1.26733 -0.89871 -0.62626<br>C 2.78681 -0.23439 -0.05618<br>N 3.87857 -0.89238 -0.41705<br>H 3.81832 -1.79632 -0.86109<br>H 4.79965 -0.50323 -0.27120<br>N 2.83171 0.85159 0.68208<br>H 1.98606 1.44404 0.74733<br>H 3.70828 1.17922 1.06658						
5-adduct, conf4	C 1.19604 -1.25324 0.49917<br>C 2.02024 -0.64558 -0.63644<br>C 2.01283 0.75479 -0.68381<br>C 1.26469 1.56677 0.21396<br>C 0.32903 1.04389 1.08279<br>C 0.10371 -0.39350 1.12293<br>H 2.67122 1.23921 -1.40198<br>H -0.18764 1.67410 1.80020<br>H -0.18268 -0.78374 2.10003<br>O 1.36749 -2.39633 0.88682<br>O 2.68480 -1.42494 -1.36573<br>C 1.51856 3.04799 0.19418<br>H 2.56610 3.26014 0.43470<br>H 1.33471 3.45555 -0.80659<br>H 0.88438 3.58019 0.90737<br>S -1.35906 -1.15533 -0.00013<br>C -2.49277 0.13289 -0.30318<br>N -3.76094 -0.22380 -0.45305<br>H -4.04837 -1.17505 -0.27860<br>H -4.46152 0.42939 -0.77560<br>N -2.10389 1.38189 -0.45281<br>H -1.13404 1.61016 -0.22230<br>H -2.77578 2.13484 -0.52059	C_1	-968.22578<br>(6.1)	-968.03495<br>(5.9)	-968.09165<br>(4.1)	-968.94140<br>(8.3)	-968.80727<br>(6.8)
5-adduct, conf5	C 1.22327 -1.18193 0.49148<br>C 2.32858 -0.56224 -0.33723<br>C 2.26502 0.82567 -0.45718	C_1	-968.22446<br>(6.9)	-968.03297<br>(7.2)	-968.08812<br>(6.3)	-968.94656<br>(5.1)	-968.81021<br>(4.9)

	C 1.24000 1.65308 0.11943<br>C 0.15456 1.13197 0.75509<br>C -0.01283 -0.32880 0.82864<br>H 3.07726 1.31729 -0.99034<br>H -0.61398 1.77064 1.17864<br>H -0.40297 -0.70289 1.77984<br>O 1.25646 -2.34399 0.86686<br>O 3.22845 -1.32796 -0.79488<br>C 1.41040 3.14144 -0.00241<br>H 2.35507 3.46383 0.45021<br>H 1.44776 3.43781 -1.05739<br>H 0.59254 3.68390 0.47866<br>S -1.19321 -0.97351 -0.51304<br>C -2.65856 -0.08654 -0.18214<br>N -3.01662 0.26296 1.03867<br>H -2.52989 -0.08918 1.84812<br>H -3.84617 0.81647 1.20643<br>N -3.42784 0.18383 -1.22182<br>H -3.08824 0.03774 -2.16113<br>H -4.37060 0.53270 -1.11234						

[a] Some starting structures dissociate to neutral molecules.

Table S10.4. Reduced anionic form in water.

Structure	Schematic drawing	Symmetry	G_{PCM}	$H_{PCM,RRHO}$	$G_{PCM,RRHO}$	$G_{SMD,M06\text{-}2X,large}$	$G_{SMD,RRHO,M06\text{-}2X,large}$
6-adduct, conf1	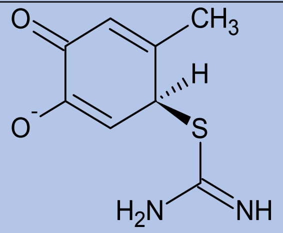<br>(3*S*)-3-(carbamimidoylsulfanyl)-4-methyl-6-oxocyclohexa-1,4-dien-1-olate<br><br>C 1.95270 0.81598 -0.50467<br>C 2.22544 -0.27930 0.50943<br>C 1.27873 -1.38948 0.62864<br>C 0.14220 -1.49771 -0.08959<br>C -0.18346 -0.44564 -1.10768	C_1	-967.76790 (3.0)	-967.59040 (2.7)	-967.64415 (2.6)	-968.49092 (1.6)	-968.36717 (2.2)

	C 0.76634 0.69301 -1.20928<br>H 1.54997 -2.16250 1.34586<br>H -0.25418 -0.96694 -2.07748<br>H 0.53619 1.44299 -1.96419<br>O 2.80499 1.75748 -0.61977<br>O 3.23598 -0.25249 1.21874<br>C -0.77331 -2.67234 0.05971<br>H -1.02689 -3.09978 -0.91688<br>H -0.31094 -3.44901 0.67332<br>H -1.71922 -2.37741 0.52733<br>S -1.97942 0.08632 -0.99289<br>C -2.13801 0.85667 0.61251<br>N -3.29297 0.73917 1.16614<br>H -3.33923 1.30889 2.01283<br>N -1.06383 1.55445 1.05438<br>H -0.19078 1.50709 0.53156<br>H -1.02807 1.83313 2.02402						
6-adduct, conf2	C 1.79724 1.04040 -0.54865<br>C 2.28369 -0.00581 0.43019<br>C 1.53011 -1.25734 0.57289<br>C 0.38458 -1.53208 -0.08432<br>C -0.14035 -0.52958 -1.07115<br>C 0.63845 0.72118 -1.22961<br>H 1.95664 -1.98487 1.26180<br>H -0.20733 -1.06189 -2.03537<br>H 0.25704 1.43165 -1.96067<br>O 2.48165 2.11281 -0.68157<br>O 3.30632 0.16643 1.10291<br>C -0.34062 -2.82909 0.08939<br>H -0.52627 -3.30667 -0.87954<br>H 0.23526 -3.51639 0.71308<br>H -1.32055 -2.67326 0.55470<br>S -1.97141 -0.21356 -0.85812<br>C -2.09237 0.70806 0.67564<br>N -1.24931 0.75912 1.63499<br>H -0.43992 0.17084 1.46001<br>N -3.31188 1.32952 0.76172<br>H -3.73652 1.65975 -0.09237<br>H -3.41554 1.94886 1.55436	C_1	-967.76835 (2.7)	-967.59048 (2.7)	-967.64573 (1.6)	-968.49332 (0.1)	-968.37070 (0.0)
6-adduct, conf3	C 1.70865 1.00809 -0.63795<br>C 2.20731 0.03709 0.40964<br>C 1.49040 -1.22961 0.61054<br>C 0.36750 -1.57687 -0.05304	C_1	-967.76516 (4.7)	-967.58755 (4.5)	-967.64239 (3.7)	-968.49031 (2.0)	-968.36754 (2.0)

	C -0.20024 -0.62456 -1.06326<br>C 0.55969 0.62358 -1.30288<br>H 1.93600 -1.91094 1.33369<br>H -0.31510 -1.19367 -2.00042<br>H 0.16306 1.28528 -2.07068<br>O 2.37375 2.08224 -0.83437<br>O 3.21082 0.27965 1.08839<br>C -0.29852 -2.90102 0.14665<br>H -0.36268 -3.44593 -0.80270<br>H 0.25405 -3.51424 0.86176<br>H -1.32556 -2.77645 0.50711<br>S -2.03089 -0.29082 -0.77528<br>C -2.11268 0.84813 0.59554<br>N -1.22889 0.66758 1.61557<br>H -0.41864 0.07958 1.47823<br>H -1.10364 1.43303 2.26303<br>N -3.08462 1.68704 0.54832<br>H -3.14796 2.20988 1.42361						
6-adduct, conf4	C -1.97089 -0.87577 -0.46868<br>C -2.27011 0.24812 0.50229<br>C -1.34471 1.38012 0.58921<br>C -0.20064 1.48164 -0.11683<br>C 0.14095 0.40733 -1.10621<br>C -0.78563 -0.75312 -1.17179<br>H -1.63603 2.17179 1.27773<br>H 0.18944 0.90766 -2.08878<br>H -0.53119 -1.52821 -1.89258<br>O -2.80543 -1.83874 -0.55726<br>O -3.28243 0.22835 1.21083<br>C 0.70005 2.67032 0.00634<br>H 0.96601 3.06771 -0.97965<br>H 0.22011 3.46215 0.58617<br>H 1.64107 2.40238 0.49993<br>S 1.93532 -0.09939 -0.99924<br>C 2.05402 -0.84220 0.63322<br>N 3.35791 -0.87436 1.05289<br>H 3.96392 -0.11617 0.77593<br>H 3.49315 -1.20773 1.99806<br>N 1.10726 -1.35846 1.31765<br>H 0.23674 -1.35510 0.78335	C_1	-967.76994 (1.7)	-967.59199 (1.7)	-967.64616 (1.4)	-968.49354 (0.0)	-968.36976 (0.6)
6-adduct, conf5	C 1.78903 -1.10243 0.48093<br>C 2.32626 -0.00194 -0.40835<br>C 1.60562 1.26909 -0.48502	C_1	-967.76250 (6.4)	-967.58500 (6.1)	-967.64026 (5.1)	-968.48869 (3.0)	-968.36644 (2.7)

	C 0.43314 1.51140 0.13360<br>C -0.12100 0.47554 1.06288<br>C 0.61815 -0.80628 1.14972<br>H 2.06085 2.03011 -1.11741<br>H -0.14987 0.95957 2.05568<br>H 0.21011 -1.55021 1.83186<br>O 2.44901 -2.19763 0.55901<br>O 3.36663 -0.15686 -1.06147<br>C -0.28719 2.81334 -0.01161<br>H -0.55037 3.23472 0.96540<br>H 0.32377 3.53958 -0.55307<br>H -1.22671 2.67126 -0.55775<br>S -1.96248 0.22539 0.88721<br>C -2.15696 -0.59495 -0.68866<br>N -1.44189 -0.27246 -1.69887<br>H -1.66589 -0.86998 -2.49676<br>N -3.20751 -1.47385 -0.65075<br>H -3.42556 -1.92699 0.22404<br>H -3.39820 -2.01677 -1.48150						
6-adduct, conf6	C 1.68468 -1.01510 0.64758<br>C 2.19145 -0.04806 -0.40090<br>C 1.49268 1.23016 -0.59333<br>C 0.37487 1.58670 0.07346<br>C -0.20897 0.63094 1.07065<br>C 0.53973 -0.62061 1.31411<br>H 1.94825 1.91125 -1.31043<br>H -0.35101 1.19134 2.00854<br>H 0.13678 -1.27927 2.08117<br>O 2.34182 -2.09343 0.84364<br>O 3.18759 -0.30362 -1.08490<br>C -0.27162 2.92268 -0.11042<br>H -0.31177 3.46312 0.84289<br>H 0.28065 3.53064 -0.83023<br>H -1.30571 2.81724 -0.45635<br>S -2.03981 0.30229 0.72592<br>C -2.08171 -0.86040 -0.63832<br>N -1.22436 -0.63341 -1.66776<br>H -0.43340 -0.02063 -1.53538<br>H -1.09365 -1.39822 -2.31476<br>N -2.96065 -1.79515 -0.69167<br>H -3.50018 -1.83368 0.16891	C_1	-967.76784 (3.0)	-967.58999 (3.0)	-967.64463 (2.3)	-968.49205 (0.9)	-968.36883 (1.2)
6-adduct, conf7	C -1.98610 -0.46354 -0.59398<br>C -1.90701 0.36254 0.67606	C_1	-967.76001 (7.9)	-967.58240 (7.8)	-967.63751 (6.8)	-968.48630 (4.5)	-968.36380 (4.3)

	C -0.84705 1.36909 0.80410<br>C 0.14402 1.52081 -0.09633<br>C 0.22669 0.56677 -1.24678<br>C -0.94444 -0.28540 -1.48423<br>H -0.91944 2.02748 1.66847<br>H 0.52752 1.11141 -2.15199<br>H -0.93741 -0.88210 -2.39459<br>O -2.99379 -1.23374 -0.75036<br>O -2.73827 0.23057 1.58103<br>C 1.14094 2.63298 -0.01221<br>H 0.95471 3.35929 -0.81376<br>H 1.07431 3.15756 0.94382<br>H 2.16424 2.26929 -0.15177<br>S 1.86281 -0.43563 -1.03945<br>C 1.62374 -1.19878 0.56978<br>N 0.85415 -2.21766 0.67908<br>H 0.80559 -2.52251 1.65460<br>N 2.38530 -0.62999 1.55163<br>H 2.76493 0.29182 1.40664<br>H 2.23606 -0.90992 2.51096						
6-adduct, conf8	C 1.95654 -0.80084 0.50325<br>C 2.21516 0.29832 -0.51060<br>C 1.25954 1.40208 -0.62216<br>C 0.12686 1.50003 0.10330<br>C -0.18620 0.44031 1.11682<br>C 0.77546 -0.68605 1.21815<br>H 1.52194 2.17979 -1.33751<br>H -0.27891 0.95309 2.08874<br>H 0.55708 -1.43725 1.97524<br>O 2.81678 -1.73544 0.61207<br>O 3.22234 0.28138 -1.22454<br>C -0.79603 2.67044 -0.03312<br>H -1.04105 3.09375 0.94750<br>H -0.34378 3.45129 -0.64891<br>H -1.74536 2.37311 -0.49203<br>S -1.97721 -0.12439 0.97764<br>C -2.11385 -0.88730 -0.64321<br>N -1.04290 -1.60394 -1.05379<br>H -0.17558 -1.54774 -0.52580<br>H -1.00806 -1.87611 -2.02523<br>N -3.20213 -0.82216 -1.32423<br>H -3.87341 -0.20627 -0.87286	C_1	-967.77038 (1.4)	-967.59268 (1.3)	-967.64670 (1.0)	-968.49270 (0.5)	-968.36902 (1.1)

6-adduct, conf9	C 1.60186 -1.37907 0.25418<br>C 2.54399 -0.29306 -0.22085<br>C 2.11110 1.10651 -0.15018<br>C 0.87770 1.49252 0.23033<br>C -0.13466 0.44414 0.58603<br>C 0.35043 -0.94742 0.65341<br>H 2.86528 1.84733 -0.41131<br>H -0.62248 0.75002 1.52020<br>H -0.36367 -1.69024 1.00321<br>O 2.02760 -2.58392 0.26922<br>O 3.67371 -0.56298 -0.64341<br>C 0.48896 2.93419 0.34024<br>H 0.15077 3.16404 1.35773<br>H 1.33159 3.58636 0.09968<br>H -0.34315 3.18063 -0.32905<br>S -1.47919 0.65531 -0.73964<br>C -2.76889 -0.44862 -0.19790<br>N -3.39186 -1.08239 -1.12417<br>H -4.19800 -1.57838 -0.73963<br>N -2.98419 -0.52538 1.14930<br>H -2.75688 0.27188 1.72427<br>H -3.79745 -1.04220 1.45531	C_1	-967.76342 (5.8)	-967.58559 (5.8)	-967.64153 (4.3)	-968.48827 (3.3)	-968.36639 (2.7)
6-adduct, conf10	C 1.59330 -1.36912 0.28948<br>C 2.53008 -0.29278 -0.22020<br>C 2.10048 1.10873 -0.16711<br>C 0.87118 1.50123 0.22036<br>C -0.13925 0.45811 0.59389<br>C 0.34483 -0.92934 0.69052<br>H 2.85332 1.84437 -0.44598<br>H -0.64777 0.77835 1.51117<br>H -0.36657 -1.66434 1.06192<br>O 2.02135 -2.57177 0.32973<br>O 3.65231 -0.57277 -0.65506<br>C 0.48531 2.94426 0.32005<br>H 0.18041 3.18807 1.34492<br>H 1.31926 3.59332 0.04361<br>H -0.36849 3.18029 -0.32504<br>S -1.48162 0.64266 -0.75104<br>C -2.76315 -0.47447 -0.19105<br>N -3.39850 -1.24670 -0.99429<br>H -2.97553 -1.22884 -1.91884<br>N -3.03774 -0.45532 1.14584<br>H -2.91266 0.41012 1.64857	C_1	-967.76683 (3.6)	-967.58874 (3.8)	-967.64469 (2.3)	-968.49026 (2.1)	-968.36813 (1.6)

	H -3.84836 -0.99320 1.42253						
6-adduct, conf11	C 2.26802 -1.05551 0.21853<br>C 2.71843 0.33940 -0.15676<br>C 1.74382 1.43318 -0.13817<br>C 0.43122 1.26743 0.12176<br>C -0.08654 -0.10465 0.42715<br>C 0.92046 -1.19084 0.48790<br>H 2.14424 2.42411 -0.34756<br>H -0.69635 -0.04948 1.34416<br>H 0.54665 -2.17665 0.76016<br>O 3.14919 -1.98344 0.26831<br>O 3.89501 0.57326 -0.45950<br>C -0.52202 2.41934 0.15936<br>H -0.99774 2.49376 1.14382<br>H -0.01015 3.36022 -0.05572<br>H -1.32957 2.28578 -0.56942<br>S -1.40614 -0.55495 -0.81655<br>C -2.90904 -0.31790 0.11972<br>N -4.02011 -0.48242 -0.66321<br>H -3.92591 -0.95000 -1.55329<br>H -4.89772 -0.64972 -0.19129<br>N -2.87426 -0.00385 1.36048<br>H -3.81440 0.08384 1.74570	C_1	-967.76787 (3.0)	-967.59031 (2.8)	-967.64574 (1.6)	-968.49100 (1.6)	-968.36888 (1.1)
6-adduct, conf12	C 1.69421 -1.36464 0.16541<br>C 2.60358 -0.22088 -0.23145<br>C 2.10683 1.15524 -0.12685<br>C 0.85272 1.47758 0.24332<br>C -0.11472 0.37571 0.56848<br>C 0.42433 -1.00028 0.57220<br>H 2.83221 1.93564 -0.35116<br>H -0.58617 0.63702 1.52760<br>H -0.26196 -1.78868 0.87481<br>O 2.16072 -2.55320 0.11651<br>O 3.75885 -0.42338 -0.62028<br>C 0.40288 2.89859 0.38583<br>H 0.01429 3.08154 1.39461<br>H 1.22950 3.58862 0.20229<br>H -0.40899 3.13658 -0.31057<br>S -1.51045 0.58240 -0.67712<br>C -2.80294 -0.43581 0.03705<br>N -3.75137 -0.73109 -0.90436<br>H -3.45732 -0.83281 -1.86424<br>H -4.46925 -1.37570 -0.60261	C_1	-967.76784 (3.0)	-967.58998 (3.0)	-967.64587 (1.6)	-968.49168 (1.2)	-968.36971 (0.6)

	N -2.94286 -0.80742 1.25378<br>H -2.22339 -0.43140 1.86338						
6-adduct, conf13	C 1.87069 -1.29019 0.10410<br>C 2.65972 -0.04003 -0.22457<br>C 2.02678 1.27051 -0.04226<br>C 0.73955 1.44111 0.31499<br>C -0.12473 0.23053 0.52608<br>C 0.55690 -1.08200 0.47970<br>H 2.67551 2.13178 -0.19340<br>H -0.67061 0.38230 1.46931<br>H -0.04793 -1.95946 0.70286<br>O 2.46502 -2.41809 0.02182<br>O 3.82849 -0.10352 -0.61914<br>C 0.15195 2.79576 0.55907<br>H -0.20263 2.87730 1.59381<br>H 0.89240 3.57984 0.38564<br>H -0.71387 2.98075 -0.08573<br>S -1.48870 0.36147 -0.76174<br>C -2.84999 -0.49141 0.03183<br>N -2.85934 -1.12525 1.14444<br>H -1.94629 -1.16703 1.58496<br>N -3.96863 -0.44823 -0.75848<br>H -4.08877 0.34011 -1.37721<br>H -4.81605 -0.76756 -0.30917	C_1	-967.76761 (3.2)	-967.58971 (3.2)	-967.64505 (2.1)	-968.49136 (1.4)	-968.36880 (1.2)
6-adduct, conf14	C 1.94699 -1.24469 0.09096<br>C 2.67322 0.05553 -0.18458<br>C 1.96433 1.32299 0.01879<br>C 0.66019 1.41022 0.34568<br>C -0.14579 0.15110 0.48404<br>C 0.61421 -1.11764 0.43334<br>H 2.56821 2.22297 -0.08562<br>H -0.74533 0.23773 1.40064<br>H 0.06216 -2.03925 0.61577<br>O 2.60569 -2.33582 -0.00114<br>O 3.85318 0.06544 -0.55024<br>C -0.00632 2.72163 0.61996<br>H -0.37745 2.75167 1.65176<br>H 0.68914 3.55221 0.47996<br>H -0.87323 2.87327 -0.03185<br>S -1.46476 0.25135 -0.87065<br>C -2.93829 -0.41524 -0.12680<br>N -2.79379 -1.15388 1.01556<br>H -1.94305 -1.67886 1.15361	C_1	-967.76307 (6.0)	-967.58526 (6.0)	-967.64052 (4.9)	-968.48726 (3.9)	-968.36470 (3.8)

	H -3.62355 -1.62076 1.35625<br>N -4.03210 -0.11019 -0.72539<br>H -4.82184 -0.61238 -0.31848						
6-adduct, conf15	C 1.97314 -1.22836 0.08493<br>C 2.67515 0.08705 -0.18628<br>C 1.94322 1.34079 0.02223<br>C 0.63909 1.40177 0.35512<br>C -0.13993 0.12652 0.49273<br>C 0.64037 -1.12683 0.43706<br>H 2.52929 2.25236 -0.08272<br>H -0.75172 0.19487 1.40220<br>H 0.10422 -2.05634 0.62576<br>O 2.65100 -2.30658 -0.01472<br>O 3.85455 0.12056 -0.55182<br>C -0.05306 2.69808 0.63661<br>H -0.41486 2.71877 1.67197<br>H 0.62234 3.54405 0.49086<br>H -0.92924 2.83074 -0.00689<br>S -1.46413 0.20343 -0.87017<br>C -2.95130 -0.43251 -0.11511<br>N -2.80690 -1.18153 1.01899<br>H -1.98803 -1.76386 1.11037<br>H -3.66290 -1.61549 1.33917<br>N -4.11174 -0.18475 -0.60447<br>H -4.05712 0.49323 -1.36100	C_1	-967.76630 (4.0)	-967.58831 (4.1)	-967.64388 (2.8)	-968.48907 (2.8)	-968.36665 (2.5)
6-adduct, conf16	C 2.35315 -0.95920 0.19556<br>C 2.65982 0.46233 -0.23091<br>C 1.60140 1.47416 -0.15496<br>C 0.33320 1.21320 0.21730<br>C -0.04855 -0.19203 0.57699<br>C 1.04983 -1.19129 0.58944<br>H 1.90112 2.48754 -0.41757<br>H -0.56548 -0.17569 1.54706<br>H 0.77857 -2.19410 0.91413<br>O 3.30281 -1.81465 0.18052<br>O 3.78620 0.78729 -0.62101<br>C -0.70250 2.28895 0.30407<br>H -1.12774 2.34348 1.31288<br>H -0.27453 3.26254 0.05483<br>H -1.53594 2.09486 -0.38080<br>S -1.32782 -0.82411 -0.63180<br>C -2.92227 -0.31580 0.03457<br>N -3.23803 -0.06725 1.24977	C_1	-967.76764 (3.1)	-967.58990 (3.0)	-967.64571 (1.7)	-968.49245 (0.7)	-968.37052 (0.1)

	H -2.46699 -0.21622 1.89357<br>N -3.86803 -0.32826 -0.95167<br>H -3.58884 -0.09667 -1.89296<br>H -4.78915 -0.01450 -0.67757						
6-adduct, conf17	C 2.35099 -0.94243 0.15994<br>C 2.63571 0.50806 -0.17741<br>C 1.56053 1.49489 -0.04445<br>C 0.29439 1.18393 0.29742<br>C -0.06784 -0.24835 0.54871<br>C 1.04772 -1.22354 0.52156<br>H 1.84339 2.52920 -0.23427<br>H -0.62967 -0.30286 1.48978<br>H 0.79333 -2.25096 0.77612<br>O 3.31854 -1.77595 0.10693<br>O 3.75973 0.87212 -0.54017<br>C -0.76661 2.22701 0.45183<br>H -1.21063 2.18478 1.45317<br>H -0.35856 3.22791 0.29382<br>H -1.58273 2.07067 -0.26310<br>S -1.29713 -0.80265 -0.76992<br>C -2.91689 -0.30977 -0.19211<br>N -3.15067 -0.46566 1.14634<br>H -2.66437 -1.20361 1.63434<br>H -4.09601 -0.30823 1.46916<br>N -3.72530 0.14174 -1.08027<br>H -4.66310 0.25477 -0.68989	C_1	-967.76401 (5.4)	-967.58618 (5.4)	-967.64107 (4.6)	-968.48958 (2.5)	-968.36664 (2.5)
6-adduct, conf18	C 2.35000 -0.93883 0.16430<br>C 2.62482 0.50898 -0.19369<br>C 1.55011 1.49495 -0.04913<br>C 0.29014 1.18329 0.31378<br>C -0.06420 -0.24881 0.57617<br>C 1.05329 -1.21973 0.55041<br>H 1.82815 2.52877 -0.24827<br>H -0.63171 -0.30447 1.51340<br>H 0.80625 -2.24468 0.82126<br>O 3.31936 -1.76906 0.10692<br>O 3.74050 0.87170 -0.58162<br>C -0.76912 2.22572 0.48234<br>H -1.19809 2.18495 1.49014<br>H -0.36410 3.22644 0.31588<br>H -1.59637 2.06901 -0.21938<br>S -1.29454 -0.81927 -0.74298<br>C -2.92725 -0.31120 -0.19332	C_1	-967.76727 (3.4)	-967.58917 (3.5)	-967.64422 (2.6)	-968.49159 (1.2)	-968.36853 (1.4)

	N -3.17616 -0.43545 1.14356<br>H -2.72465 -1.18672 1.64367<br>H -4.13669 -0.26799 1.41348<br>N -3.81988 0.12612 -1.00391<br>H -3.42350 0.28110 -1.92767						
6-adduct, conf19	C -2.28114 -1.04307 -0.20842<br>C -2.71349 0.35985 0.15853<br>C -1.72650 1.44223 0.12499<br>C -0.41685 1.25899 -0.13796<br>C 0.08443 -0.12203 -0.43007<br>C -0.93571 -1.19681 -0.47840<br>H -2.11490 2.43974 0.32570<br>H 0.69183 -0.08465 -1.34937<br>H -0.57383 -2.18913 -0.74293<br>O -3.17350 -1.96072 -0.25008<br>O -3.88536 0.60985 0.46674<br>C 0.54888 2.39997 -0.18964<br>H 1.03087 2.45375 -1.17238<br>H 0.04592 3.34954 0.00752<br>H 1.35089 2.27062 0.54608<br>S 1.40024 -0.57981 0.81468<br>C 2.90576 -0.32594 -0.11536<br>N 2.87370 -0.07635 -1.37057<br>H 3.81416 0.03623 -1.74829<br>N 4.01137 -0.52052 0.66723<br>H 3.92808 -0.39817 1.66595<br>H 4.90633 -0.25491 0.28092	C_1	-967.76790 (3.0)	-967.59025 (2.8)	-967.64507 (2.1)	-968.49101 (1.6)	-968.36819 (1.6)
6-adduct, conf20	C 2.33437 -0.98119 0.23330<br>C 2.67766 0.41659 -0.24057<br>C 1.64130 1.45357 -0.21352<br>C 0.36495 1.23601 0.15840<br>C -0.05046 -0.14513 0.57239<br>C 1.02387 -1.16887 0.62699<br>H 1.96501 2.44869 -0.51450<br>H -0.56774 -0.08254 1.54096<br>H 0.72741 -2.15217 0.98697<br>O 3.26344 -1.85837 0.25445<br>O 3.81500 0.70154 -0.62950<br>C -0.64663 2.33719 0.19295<br>H -1.05557 2.46231 1.20246<br>H -0.20125 3.28493 -0.11773<br>H -1.49313 2.12043 -0.46842<br>S -1.34235 -0.79053 -0.61389	C_1	-967.76710 (3.5)	saddle point			

	C -2.93739 -0.29223 0.05287<br>N -3.91728 -0.44390 -0.88864<br>H -3.73002 -0.99000 -1.71563<br>H -4.86232 -0.49733 -0.53479<br>N -3.22126 0.15025 1.22056<br>H -2.40865 0.22867 1.82303						
5-adduct, conf1	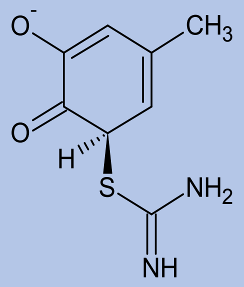<br>(5*R*)-5-(carbamimidoylsulfanyl)-3-methyl-6-oxocyclohexa-1,3-dien-1-olate<br><br>C 0.56825 1.21827 -0.33304<br>C 1.99698 1.09414 0.14962<br>C 2.44429 -0.21225 0.33676<br>C 1.70384 -1.40067 -0.00917<br>C 0.49393 -1.33113 -0.61405<br>C -0.06372 -0.00206 -0.99701<br>H 3.42210 -0.33919 0.80073<br>H -0.04217 -2.21952 -0.93398<br>H 0.20920 0.17393 -2.05722<br>O -0.04571 2.27720 -0.22809<br>O 2.62377 2.15997 0.45003<br>C 2.33971 -2.72828 0.30138<br>H 3.29534 -2.83559 -0.22558<br>H 2.55746 -2.81662 1.37242<br>H 1.69358 -3.56166 0.01228<br>S -1.89166 0.02329 -1.12601<br>C -2.44898 -0.21189 0.55580<br>N -3.46606 -0.97958 0.70861<br>H -3.80062 -0.94150 1.67319<br>N -1.74846 0.45928 1.51419<br>H -1.27720 1.31323 1.24322<br>H -2.11966 0.44226 2.45437	C_1	-967.76369 (5.6)	-967.58635 (5.3)	-967.63985 (5.3)	-968.48479 (5.5)	-968.36095 (6.1)
5-adduct, conf2	C -0.38281 -1.27908 -0.37956<br>C -1.75711 -1.31214 0.26264<br>C -2.38879 -0.07774 0.40350	C_1	-967.76937 (2.1)	-967.59189 (1.8)	-967.64605 (1.4)	-968.48998 (2.2)	-968.36667 (2.5)

	C -1.87213 1.17717 -0.08035<br>C -0.69470 1.25555 -0.75002<br>C 0.06302 0.01184 -1.07871<br>H -3.34315 -0.06543 0.92888<br>H -0.32787 2.19379 -1.15621<br>H -0.11928 -0.22496 -2.14312<br>O 0.33175 -2.27215 -0.39309<br>O -2.18730 -2.43141 0.68404<br>C -2.69547 2.41015 0.17370<br>H -3.68182 2.32240 -0.29701<br>H -2.86809 2.54995 1.24739<br>H -2.20783 3.30757 -0.21643<br>S 1.87721 0.23008 -1.07878<br>C 2.19981 0.43652 0.67618<br>N 1.36038 0.75923 1.58418<br>H 0.44866 0.98806 1.18580<br>N 3.53253 0.27459 0.94450<br>H 4.05208 -0.39228 0.39297<br>H 3.77518 0.29776 1.92567						
5-adduct, conf3	C -0.37094 -1.31148 -0.44024<br>C -1.67674 -1.29118 0.33576<br>C -2.30586 -0.05229 0.44097<br>C -1.81635 1.17152 -0.13763<br>C -0.66085 1.22019 -0.85093<br>C 0.10269 -0.02887 -1.13796<br>H -3.22895 -0.00895 1.01757<br>H -0.31853 2.13909 -1.31869<br>H -0.01698 -0.27006 -2.20813<br>O 0.27591 -2.34359 -0.55269<br>O -2.06772 -2.38139 0.85573<br>C -2.64134 2.41219 0.06986<br>H -3.63547 2.29249 -0.37670<br>H -2.79513 2.60554 1.13793<br>H -2.16836 3.29111 -0.37611<br>S 1.92803 0.17966 -1.05698<br>C 2.26485 0.42902 0.67704<br>N 1.19594 0.68779 1.47829<br>H 0.37045 1.11127 1.05861<br>H 1.39111 0.95408 2.43363<br>N 3.50073 0.30782 1.00595<br>H 3.63832 0.54675 1.98832	C_1	-967.76716 (3.4)	-967.58988 (3.1)	-967.64349 (3.1)	-968.48807 (3.4)	-968.36440 (4.0)
5-adduct, conf4	C 0.36557 1.30939 -0.44754<br>C 1.67240 1.29942 0.32657	C_1	-967.76966 (1.9)	-967.59222 (1.6)	-967.64602 (1.5)	-968.48978 (2.4)	-968.36614 (2.9)

	C 2.30823 0.06439 0.43533<br>C 1.82512 -1.16412 -0.13876<br>C 0.66902 -1.22252 -0.85021<br>C -0.10196 0.02077 -1.13800<br>H 3.23239 0.02817 1.01068<br>H 0.33238 -2.14447 -1.31597<br>H 0.00375 0.25800 -2.21036<br>O -0.28807 2.33665 -0.56417<br>O 2.05796 2.39383 0.84173<br>C 2.65924 -2.39847 0.06972<br>H 3.65173 -2.27255 -0.37878<br>H 2.81627 -2.58834 1.13793<br>H 2.19179 -3.28171 -0.37351<br>S -1.92723 -0.20091 -1.03618<br>C -2.26037 -0.43904 0.70610<br>N -1.18632 -0.68590 1.50046<br>H -0.37133 -1.12967 1.08472<br>H -1.40529 -0.94732 2.45212<br>N -3.45440 -0.35875 1.17288<br>H -4.11369 -0.05796 0.45888						
5-adduct, conf5	C -0.52145 -1.26002 -0.43232<br>C -1.84789 -1.15972 0.29692<br>C -2.38983 0.11697 0.40698<br>C -1.77021 1.32511 -0.08025<br>C -0.58955 1.30428 -0.74204<br>C 0.05665 0.00504 -1.07803<br>H -3.33859 0.20952 0.93545<br>H -0.13376 2.21167 -1.12865<br>H -0.11141 -0.18646 -2.15378<br>O 0.03275 -2.33992 -0.59552<br>O -2.34560 -2.23304 0.77084<br>C -2.49211 2.62130 0.17223<br>H -3.48326 2.61427 -0.29733<br>H -2.65308 2.77766 1.24581<br>H -1.93437 3.47672 -0.21924<br>S 1.88205 0.05373 -1.05335<br>C 2.23938 0.22083 0.68563<br>N 1.35410 -0.04647 1.56767<br>H 1.71725 0.11787 2.50734<br>N 3.55450 0.55790 0.88684<br>H 4.02706 1.09380 0.17326<br>H 3.83004 0.78611 1.83187	C_1	-967.76370 (5.6)	-967.58648 (5.2)	-967.64107 (4.6)	-968.48771 (3.7)	-968.36508 (3.5)

5-adduct, conf6	C -0.40265 -1.28036 -0.36520<br>C -1.78371 -1.29344 0.26321<br>C -2.39978 -0.05032 0.39550<br>C -1.86143 1.19650 -0.08582<br>C -0.67832 1.25714 -0.74705<br>C 0.06219 0.00137 -1.06946<br>H -3.35856 -0.02408 0.91228<br>H -0.29410 2.18940 -1.15075<br>H -0.12529 -0.23827 -2.13256<br>O 0.30085 -2.28127 -0.36548<br>O -2.23282 -2.40571 0.68336<br>C -2.66896 2.44129 0.16115<br>H -3.65299 2.36695 -0.31671<br>H -2.84751 2.58444 1.23342<br>H -2.16593 3.33141 -0.22612<br>S 1.87878 0.18944 -1.07675<br>C 2.21759 0.42208 0.67244<br>N 1.38010 0.73778 1.58602<br>H 0.45690 0.92930 1.19698<br>N 3.53289 0.18650 0.95720<br>H 4.19796 0.09752 0.20479<br>H 3.88437 0.59247 1.81240	C_1	-967.76862 (2.5)	-967.59133 (2.2)	-967.64609 (1.4)	-968.48946 (2.6)	-968.36693 (2.4)
5-adduct, conf7	C -0.56993 -1.21764 -0.34449<br>C -1.99747 -1.08817 0.14111<br>C -2.43984 0.21933 0.33105<br>C -1.69512 1.40555 -0.01324<br>C -0.48464 1.33359 -0.61685<br>C 0.06927 0.00380 -1.00058<br>H -3.41733 0.34918 0.79479<br>H 0.05425 2.22097 -0.93487<br>H -0.18974 -0.16743 -2.06471<br>O 0.03731 -2.28076 -0.24922<br>O -2.62774 -2.15247 0.43897<br>C -2.32730 2.73461 0.29852<br>H -3.28207 2.84535 -0.22926<br>H -2.54597 2.82165 1.36947<br>H -1.67849 3.56662 0.01155<br>S 1.89980 -0.03476 -1.10249<br>C 2.44037 0.19730 0.59371<br>N 1.72430 -0.46976 1.53998<br>H 1.27970 -1.33747 1.27113<br>H 2.10856 -0.43580 2.47472<br>N 3.45397 0.92478 0.89076	C_1	-967.76660 (3.8)	-967.58904 (3.6)	-967.64266 (3.6)	-968.48684 (4.2)	-968.36290 (4.9)

	H 3.77775 1.44356 0.07838						
5-adduct, conf8	C -0.19707 0.96404 0.64105<br>C -1.37108 1.51647 -0.14875<br>C -2.40926 0.61212 -0.35452<br>C -2.36958 -0.77739 0.03243<br>C -1.25612 -1.34873 0.56067<br>C -0.01964 -0.54438 0.72347<br>H -3.31702 0.98495 -0.82752<br>H -1.22016 -2.40369 0.81724<br>H 0.54330 -0.78621 1.62886<br>O 0.60922 1.70186 1.19479<br>O -1.34974 2.74421 -0.47779<br>C -3.62137 -1.58428 -0.17566<br>H -4.45990 -1.15424 0.38495<br>H -3.91375 -1.58003 -1.23258<br>H -3.49124 -2.62267 0.14090<br>S 1.06531 -0.97944 -0.73990<br>C 2.68430 -0.32678 -0.32053<br>N 3.26659 0.39075 -1.21004<br>H 4.22920 0.59468 -0.93194<br>N 3.15655 -0.68272 0.90718<br>H 2.79746 -1.51410 1.35042<br>H 4.10823 -0.43882 1.14304	C_1	-967.76330 (5.9)	-967.58568 (5.7)	-967.64062 (4.9)	-968.48570 (4.9)	-968.36302 (4.8)
5-adduct, conf9	C -0.17840 0.94278 0.67099<br>C -1.33572 1.51862 -0.12611<br>C -2.38535 0.63130 -0.34878<br>C -2.36930 -0.76100 0.02722<br>C -1.26870 -1.35286 0.56159<br>C -0.02263 -0.56829 0.73731<br>H -3.28297 1.02153 -0.82700<br>H -1.24994 -2.41034 0.80931<br>H 0.54416 -0.83144 1.63435<br>O 0.63146 1.66320 1.24204<br>O -1.29286 2.74793 -0.44577<br>C -3.63013 -1.54831 -0.20017<br>H -4.46864 -1.11007 0.35409<br>H -3.91039 -1.53217 -1.26022<br>H -3.51857 -2.59068 0.11024<br>S 1.05669 -1.00176 -0.73526<br>C 2.67761 -0.31532 -0.32976<br>N 3.30248 0.49753 -1.09810<br>H 2.70485 0.79136 -1.86589<br>N 3.20580 -0.74828 0.84734	C_1	-967.76669 (3.7)	-967.58880 (3.7)	-967.64365 (3.0)	-968.48773 (3.6)	-968.36469 (3.8)

	H 2.92130 -1.64591 1.20669<br>H 4.16212 -0.47953 1.03405						
5-adduct, conf10	C -0.51040 1.15018 -0.05492<br>C -2.00991 1.33784 0.04575<br>C -2.76220 0.16612 0.06133<br>C -2.21807 -1.17023 0.06741<br>C -0.88213 -1.39320 0.07765<br>C 0.06659 -0.24798 0.14680<br>H -3.84683 0.27338 0.05928<br>H -0.47104 -2.39767 0.12476<br>H 0.50870 -0.19292 1.16392<br>O 0.23546 2.10500 -0.24591<br>O -2.46746 2.52689 0.02965<br>C -3.19488 -2.31446 0.08486<br>H -3.83032 -2.27302 0.97777<br>H -3.86547 -2.26940 -0.78174<br>H -2.68377 -3.28113 0.07318<br>S 1.54751 -0.53879 -0.87522<br>C 2.85631 -0.21872 0.30067<br>N 4.08232 -0.24979 -0.30184<br>H 4.14467 -0.05469 -1.29043<br>H 4.87993 0.04806 0.24149<br>N 2.59605 -0.07204 1.54477<br>H 3.43949 0.10842 2.08768	C_1	-967.76702 (3.5)	-967.58995 (3.0)	-967.64492 (2.2)	-968.48579 (4.9)	-968.36369 (4.4)
5-adduct, conf11	C -0.40730 1.12881 0.35410<br>C -1.73084 1.36866 -0.34172<br>C -2.58649 0.27221 -0.36570<br>C -2.26679 -1.03289 0.16525<br>C -1.04267 -1.32578 0.66876<br>C 0.03186 -0.29927 0.66664<br>H -3.57674 0.41726 -0.79582<br>H -0.79814 -2.31587 1.04253<br>H 0.59778 -0.28227 1.60578<br>O 0.32864 2.06159 0.66518<br>O -1.96853 2.53503 -0.78977<br>C -3.35693 -2.06801 0.13903<br>H -4.23083 -1.72782 0.70724<br>H -3.69808 -2.24481 -0.88808<br>H -3.02148 -3.02007 0.55885<br>S 1.25852 -0.81647 -0.63017<br>C 2.83165 -0.18286 -0.01576<br>N 3.87418 -0.91527 -0.51477<br>H 3.70882 -1.58389 -1.25084	C_1	-967.76970 (1.8)	-967.59189 (1.8)	-967.64621 (1.3)	-968.48846 (3.2)	-968.36496 (3.6)

	H 4.77237 -0.45395 -0.53168<br>N 3.01812 0.78164 0.80021<br>H 2.14812 1.27223 1.00975						
5-adduct, conf12	C -0.35278 1.10621 0.32794<br>C -1.68592 1.40423 -0.31639<br>C -2.58029 0.33867 -0.33430<br>C -2.29602 -0.98572 0.16722<br>C -1.07411 -1.32435 0.64539<br>C 0.03354 -0.33282 0.64767<br>H -3.57320 0.52510 -0.74179<br>H -0.85589 -2.32660 1.00267<br>H 0.58629 -0.33588 1.59453<br>O 0.44078 2.01244 0.58752<br>O -1.89311 2.58410 -0.74446<br>C -3.41943 -1.98469 0.14128<br>H -4.27119 -1.62875 0.73296<br>H -3.78502 -2.13010 -0.88228<br>H -3.10685 -2.95512 0.53596<br>S 1.24383 -0.91584 -0.64104<br>C 2.85942 -0.34261 -0.12489<br>N 3.83184 -0.97011 -0.68459<br>H 4.72624 -0.54970 -0.43089<br>N 2.90531 0.64185 0.80922<br>H 2.11350 1.28523 0.87896<br>H 3.81697 1.02117 1.02602	C_1	-967.76995 (1.7)	-967.59227 (1.6)	-967.64585 (1.6)	-968.48784 (3.6)	-968.36374 (4.4)
5-adduct, conf13	C -0.41029 1.12830 0.35015<br>C -1.74132 1.36594 -0.33108<br>C -2.59295 0.26628 -0.35217<br>C -2.26288 -1.04082 0.16769<br>C -1.03319 -1.33078 0.65870<br>C 0.03651 -0.29899 0.65512<br>H -3.58756 0.40954 -0.77278<br>H -0.78077 -2.32209 1.02404<br>H 0.60477 -0.28205 1.59290<br>O 0.32651 2.06315 0.65413<br>O -1.98757 2.53320 -0.77253<br>C -3.34845 -2.08084 0.14394<br>H -4.21817 -1.74957 0.72374<br>H -3.69917 -2.25099 -0.88108<br>H -3.00427 -3.03468 0.55255<br>S 1.26234 -0.80255 -0.64746<br>C 2.83134 -0.17833 -0.01543<br>N 3.01821 0.79065 0.79363	C_1	-967.77047 (1.4)	-967.59250 (1.4)	-967.64627 (1.3)	-968.48903 (2.8)	-968.36484 (3.7)

	H 2.15166 1.29710 0.98296<br>N 3.87067 -0.86494 -0.58742<br>H 3.74396 -1.84696 -0.78354<br>H 4.78582 -0.61484 -0.23736						
5-adduct, conf14	C -0.35676 1.11047 0.32810<br>C -1.68763 1.39911 -0.32583<br>C -2.57790 0.32984 -0.34256<br>C -2.29175 -0.98995 0.16868<br>C -1.07143 -1.32170 0.65689<br>C 0.03071 -0.32575 0.65985<br>H -3.56909 0.51008 -0.75685<br>H -0.85251 -2.32025 1.02386<br>H 0.58547 -0.32276 1.60532<br>O 0.43373 2.01946 0.58501<br>O -1.89713 2.57529 -0.76205<br>C -3.41169 -1.99273 0.14335<br>H -4.26866 -1.63454 0.72602<br>H -3.76954 -2.14750 -0.88159<br>H -3.09891 -2.95893 0.54805<br>S 1.25112 -0.90282 -0.62681<br>C 2.87308 -0.32541 -0.11690<br>N 2.91380 0.64862 0.82544<br>H 2.13041 1.30047 0.88513<br>H 3.83568 1.01612 1.01856<br>N 3.93482 -0.85084 -0.61640<br>H 3.71627 -1.64325 -1.21577	C_1	-967.77264 (0.0)	-967.59476 (0.0)	-967.64836 (0.0)	-968.48971 (2.4)	-968.36542 (3.3)
5-adduct, conf15	C 0.52340 -1.15309 -0.06560<br>C 2.02417 -1.32520 0.04513<br>C 2.76399 -0.14555 0.06544<br>C 2.20549 1.18486 0.07171<br>C 0.86717 1.39343 0.07830<br>C -0.06894 0.23765 0.14176<br>H 3.84976 -0.24109 0.06814<br>H 0.44504 2.39323 0.12666<br>H -0.51096 0.17105 1.15858<br>O -0.21065 -2.11467 -0.26767<br>O 2.49384 -2.50949 0.03120<br>C 3.16975 2.33963 0.09437<br>H 3.80358 2.30291 0.98860<br>H 3.84270 2.30406 -0.77084<br>H 2.64812 3.30067 0.08385<br>S -1.55373 0.51459 -0.87803<br>C -2.85829 0.20703 0.30421	C_1	-967.76690 (3.6)	-967.58983 (3.1)	-967.64448 (2.4)	-968.48557 (5.0)	-968.36315 (4.7)

	N -2.58967 0.01936 1.54142<br>H -3.43346 -0.14588 2.08878<br>N -4.08577 0.18174 -0.29469<br>H -4.18458 0.53374 -1.23559<br>H -4.89829 0.30083 0.29305						
5-adduct, conf16	C 1.10943 -1.21658 0.44871<br>C 2.33219 -0.69109 -0.26719<br>C 2.39457 0.69536 -0.38599<br>C 1.37953 1.61274 0.07156<br>C 0.20867 1.18163 0.60357<br>C -0.07072 -0.27040 0.70628<br>H 3.29279 1.11582 -0.83712<br>H -0.56161 1.87826 0.92069<br>H -0.48461 -0.56606 1.67784<br>O 1.02324 -2.38284 0.81403<br>O 3.21235 -1.52775 -0.64633<br>C 1.66605 3.08198 -0.07388<br>H 2.57629 3.35896 0.47104<br>H 1.83662 3.34174 -1.12565<br>H 0.84088 3.69344 0.30110<br>S -1.31390 -0.82218 -0.56471<br>C -2.79527 0.03655 -0.02061<br>N -3.07105 0.47412 1.14910<br>H -2.34675 0.25871 1.82727<br>N -3.70709 0.09538 -1.03738<br>H -3.36728 0.17364 -1.98418<br>H -4.54837 0.61751 -0.83412	C_1	-967.76649 (3.9)	-967.58873 (3.8)	-967.64380 (2.9)	-968.48755 (3.8)	-968.36487 (3.7)
5-adduct, conf17	C 1.16132 -1.20262 0.42094<br>C 2.36409 -0.57735 -0.24648<br>C 2.33022 0.81106 -0.34980<br>C 1.24969 1.64933 0.10757<br>C 0.11090 1.12991 0.63019<br>C -0.06998 -0.33825 0.71408<br>H 3.20195 1.29841 -0.78554<br>H -0.70592 1.76380 0.96160<br>H -0.45044 -0.66960 1.68661<br>O 1.13080 -2.39349 0.71320<br>O 3.31214 -1.34257 -0.61395<br>C 1.43342 3.13663 -0.02000<br>H 2.32333 3.46998 0.52698<br>H 1.58345 3.41979 -1.06890<br>H 0.56820 3.68473 0.36262<br>S -1.27725 -0.95073 -0.57899	C_1	-967.76238 (6.4)	-967.58468 (6.3)	-967.63933 (5.7)	-968.48444 (5.7)	-968.36139 (5.8)

	C -2.76874 -0.03291 -0.23971<br>N -3.02212 0.27513 1.06499<br>H -2.62680 -0.29938 1.79351<br>H -3.92954 0.66470 1.28035<br>N -3.48536 0.24950 -1.26598<br>H -4.38492 0.64049 -0.98126						
5-adduct, conf18	C 1.15984 -1.20806 0.41468<br>C 2.35460 -0.56895 -0.25527<br>C 2.31474 0.82096 -0.33659<br>C 1.23511 1.64750 0.14373<br>C 0.10042 1.11470 0.66288<br>C -0.07521 -0.35375 0.71612<br>H 3.18176 1.31887 -0.76973<br>H -0.71747 1.73851 1.01099<br>H -0.47473 -0.70537 1.67269<br>O 1.14014 -2.40035 0.70092<br>O 3.30215 -1.32536 -0.64098<br>C 1.41473 3.13738 0.04517<br>H 2.30658 3.46170 0.59438<br>H 1.55918 3.44177 -0.99856<br>H 0.55014 3.67563 0.44281<br>S -1.27405 -0.93986 -0.60367<br>C -2.76753 -0.01723 -0.23946<br>N -3.07186 0.14226 1.07999<br>H -2.77918 -0.56825 1.73322<br>H -3.98309 0.54066 1.26373<br>N -3.51983 0.45022 -1.16601<br>H -3.07528 0.35717 -2.07577	C_1	-967.76554 (4.5)	-967.58764 (4.5)	-967.64273 (3.5)	-968.48645 (4.5)	-968.36364 (4.4)
5-adduct, conf19	C -1.17808 -1.19441 -0.46147<br>C -2.30507 -0.55124 0.31553<br>C -2.25347 0.83995 0.37653<br>C -1.19681 1.65137 -0.17484<br>C -0.08950 1.10200 -0.73665<br>C 0.07158 -0.36757 -0.76701<br>H -3.08921 1.34934 0.85528<br>H 0.72011 1.71329 -1.12375<br>H 0.48882 -0.74436 -1.70535<br>O -1.22397 -2.36398 -0.82572<br>O -3.22196 -1.30038 0.78041<br>C -1.36329 3.14395 -0.09703<br>H -2.27759 3.46301 -0.61137<br>H -1.45793 3.47004 0.94580<br>H -0.51511 3.66861 -0.54504	C_1	-967.76552 (4.5)	-967.58760 (4.5)	-967.64277 (3.5)	-968.48626 (4.6)	-968.36351 (4.5)

	S 1.22728 -0.95190 0.60098<br>C 2.73330 -0.03411 0.27583<br>N 3.09187 0.08833 -1.03372<br>H 2.82664 -0.64196 -1.67666<br>H 4.00849 0.48516 -1.19212<br>N 3.44431 0.46173 1.21983<br>H 2.96150 0.39216 2.11197						
5-adduct, conf20	C 1.06514 -1.22261 0.46601<br>C 2.31914 -0.76322 -0.24073<br>C 2.44086 0.61615 -0.39048<br>C 1.45935 1.58459 0.03317<br>C 0.26450 1.21489 0.55800<br>C -0.07834 -0.22199 0.68767<br>H 3.36156 0.98857 -0.83844<br>H -0.47822 1.95273 0.84681<br>H -0.51473 -0.48156 1.66016<br>O 0.92603 -2.37641 0.85285<br>O 3.16855 -1.64413 -0.58686<br>C 1.80966 3.03689 -0.13952<br>H 2.72325 3.28699 0.41277<br>H 2.00595 3.26562 -1.19398<br>H 1.00609 3.69076 0.20985<br>S -1.33247 -0.74443 -0.58176<br>C -2.81788 0.08555 -0.00924<br>N -3.03496 0.63489 1.12589<br>H -2.23883 0.57554 1.75173<br>N -3.77514 0.09263 -0.98580<br>H -3.68622 -0.53506 -1.77040<br>H -4.71994 0.25745 -0.66804	C_1	-967.76603 (4.1)	-967.58837 (4.0)	-967.64387 (2.8)	-968.48714 (4.0)	-968.36498 (3.6)
5-adduct, conf21	C 0.82105 -1.21043 0.55048<br>C 2.15340 -1.05645 -0.15088<br>C 2.55087 0.26107 -0.36474<br>C 1.77460 1.42491 -0.00628<br>C 0.50793 1.32170 0.46787<br>C -0.12965 -0.01103 0.59583<br>H 3.53929 0.42191 -0.79399<br>H -0.06781 2.21093 0.71300<br>H -0.73253 -0.12175 1.50284<br>O 0.47639 -2.26913 1.05960<br>O 2.81530 -2.10753 -0.42535<br>C 2.42665 2.76754 -0.18723<br>H 3.34779 2.83778 0.40336<br>H 2.71125 2.92176 -1.23508	C_1	-967.76109 (7.3)	-967.58339 (7.1)	-967.63844 (6.2)	-968.48261 (6.9)	-968.35996 (6.7)

	H 1.76264 3.58320 0.11160<br>S -1.32953 -0.36734 -0.80408<br>C -2.91597 0.13325 -0.16557<br>N -2.92783 1.00463 0.88752<br>H -2.17796 1.67427 0.97260<br>H -3.83181 1.36295 1.16502<br>N -3.92198 -0.40422 -0.75216<br>H -4.79645 0.01334 -0.43235						
5-adduct, conf22	C 0.86996 -1.19825 0.64328<br>C 2.08958 -1.01755 -0.23970<br>C 2.42566 0.31238 -0.49100<br>C 1.67960 1.44921 -0.01072<br>C 0.47080 1.30661 0.59459<br>C -0.12284 -0.04178 0.72411<br>H 3.35123 0.50048 -1.03408<br>H -0.07831 2.17126 0.95775<br>H -0.73889 -0.17799 1.61682<br>O 0.65230 -2.23728 1.25093<br>O 2.73412 -2.05089 -0.60000<br>C 2.28991 2.80865 -0.20878<br>H 3.26900 2.87319 0.28028<br>H 2.45592 3.00613 -1.27470<br>H 1.65122 3.60088 0.19101<br>S -1.27344 -0.48096 -0.71812<br>C -2.87284 0.12794 -0.20025<br>N -2.88379 1.14842 0.70544<br>H -2.13414 1.82269 0.66865<br>H -3.79837 1.54564 0.87629<br>N -3.95871 -0.37847 -0.65715<br>H -3.77540 -1.21457 -1.20685	C_1	-967.76435 (5.2)	-967.58654 (5.2)	-967.64234 (3.8)	-968.48417 (5.9)	-968.36216 (5.4)

Table S10.5. One-electron oxidized neutral form in vacuo.

Structure	Schematic drawing	Symmetry	*E*	H_{RRHO}	G_{RRHO}	$E_{M06\text{-}2X,large}$	$G_{RRHO,M06\text{-}2X,large}$
6-adduct, conf1	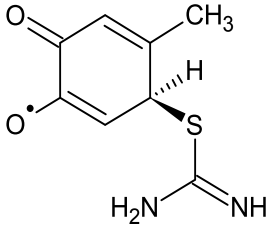<br>[(3*S*)-3-(carbamimidoylsulfanyl)-4-methyl-6-oxocyclohexa-1,4-dien-1-yl]oxidanyl<br><br>C 1.90986 0.43206 -0.59443<br>C 1.88291 -0.53936 0.59040<br>C 0.78637 -1.51343 0.63008<br>C -0.26354 -1.51180 -0.21643<br>C -0.38132 -0.46278 -1.28327<br>C 0.81410 0.37114 -1.50666<br>H 0.85731 -2.26113 1.41673<br>H -0.70800 -0.92248 -2.22593<br>H 0.84233 1.02387 -2.37571<br>O 2.85237 1.21551 -0.74958<br>O 2.75319 -0.50140 1.43915<br>C -1.37784 -2.50212 -0.11100<br>H -1.59318 -2.95838 -1.08443<br>H -1.13289 -3.29331 0.60033<br>H -2.29326 -2.00218 0.22636<br>S -1.84869 0.66410 -0.94522<br>C -1.55190 1.05365 0.78723<br>N -2.36025 0.55222 1.63418<br>H -2.16169 0.88375 2.58068<br>N -0.44362 1.82829 1.00534<br>H -0.11666 2.43324 0.26841<br>H -0.24421 2.13057 1.94838	C_1	-967.57313<br>(11.7)	-967.39499<br>(11.4)	-967.45030<br>(11.4)	-968.28148<br>(7.9)	-968.15865<br>(7.6)
6-adduct, conf2	C 1.88812 0.41506 -0.63579<br>C 1.88966 -0.41970 0.64954<br>C 0.82890 -1.42573 0.79301<br>C -0.20680 -1.56020 -0.05873<br>C -0.37567 -0.61655 -1.21350	C_1	-967.57738<br>(9.0)	-967.39876<br>(9.0)	-967.45372<br>(9.2)	-968.28486<br>(5.8)	-968.16120<br>(6.0)

	C 0.79224 0.22566 -1.53244<br>H 0.93241 -2.09064 1.64718<br>H -0.70067 -1.16773 -2.10682<br>H 0.79635 0.78669 -2.46375<br>O 2.80817 1.19985 -0.88242<br>O 2.76234 -0.26972 1.48150<br>C -1.23304 -2.63771 0.09169<br>H -1.18341 -3.33065 -0.75801<br>H -1.07669 -3.20893 1.00873<br>H -2.24558 -2.22071 0.10340<br>S -1.88026 0.48777 -0.96455<br>C -1.57956 1.16558 0.68757<br>N -2.37549 0.99451 1.66488<br>H -3.12321 0.34751 1.43228<br>N -0.41321 1.86926 0.81296<br>H -0.09947 2.41447 0.02473<br>H -0.28195 2.29978 1.71946						
6-adduct, conf3	C 1.79565 0.75389 -0.69787<br>C 2.08301 -0.01830 0.59259<br>C 1.30697 -1.24466 0.82509<br>C 0.23987 -1.61360 0.09337<br>C -0.23896 -0.76477 -1.05315<br>C 0.65356 0.34628 -1.45423<br>H 1.64896 -1.86005 1.65429<br>H -0.40376 -1.41660 -1.92583<br>H 0.41310 0.91060 -2.35120<br>O 2.53493 1.66802 -1.06392<br>O 2.94957 0.35244 1.35994<br>C -0.50381 -2.88609 0.35319<br>H -0.48022 -3.53644 -0.53059<br>H -0.06736 -3.43230 1.19190<br>H -1.55851 -2.68882 0.57365<br>S -1.98053 -0.15747 -0.82130<br>C -1.79023 1.01957 0.51089<br>N -3.01509 1.43795 0.97124<br>H -3.81148 0.82908 0.86012<br>H -3.02480 2.01600 1.79799<br>N -0.63262 1.40635 0.86396<br>H -0.64593 2.07017 1.63675	C_1	-967.57568 (10.1)	-967.39775 (9.6)	-967.45352 (9.4)	-968.28324 (6.8)	-968.16108 (6.1)
6-adduct, conf4	C -1.87959 -0.54264 -0.59097<br>C -1.96466 0.35098 0.65008<br>C -0.95655 1.41302 0.77431<br>C 0.07741 1.58282 -0.07350	C_1	-967.57451 (10.8)	-967.39619 (10.6)	-967.45200 (10.3)	-968.28122 (8.1)	-968.15871 (7.6)

	C 0.32657 0.60970 -1.19071<br>C -0.76672 -0.34475 -1.46376<br>H -1.11688 2.10713 1.59617<br>H 0.57435 1.15812 -2.11149<br>H -0.71133 -0.96089 -2.35811<br>O -2.75597 -1.37873 -0.83046<br>O -2.86012 0.20761 1.45761<br>C 1.01327 2.74571 0.03512<br>H 0.89509 3.40727 -0.83274<br>H 0.81677 3.33083 0.93588<br>H 2.05848 2.41919 0.04236<br>S 1.93394 -0.31669 -0.94309<br>C 1.55841 -1.21725 0.59570<br>N 2.42996 -0.88278 1.58978<br>H 2.86292 0.02503 1.58480<br>H 2.26828 -1.32467 2.48368<br>N 0.66827 -2.10939 0.75611<br>H 0.20154 -2.36634 -0.10721						
6-adduct, conf5	C 1.76462 -0.80477 0.69921<br>C 2.10891 -0.00788 -0.56180<br>C 1.36547 1.23862 -0.78980<br>C 0.28632 1.61597 -0.08068<br>C -0.23309 0.76327 1.04607<br>C 0.62004 -0.37981 1.44159<br>H 1.73883 1.85962 -1.60095<br>H -0.38861 1.40722 1.92648<br>H 0.34727 -0.95356 2.32332<br>O 2.46314 -1.75406 1.05715<br>O 2.99155 -0.38029 -1.30979<br>C -0.43260 2.90101 -0.34692<br>H -0.44923 3.53214 0.55076<br>H 0.04916 3.46067 -1.15123<br>H -1.47602 2.71692 -0.62516<br>S -1.98901 0.21641 0.78334<br>C -1.81788 -0.98430 -0.52937<br>N -0.66448 -1.33311 -0.93255<br>H -0.69124 -2.01373 -1.69024<br>N -3.04578 -1.38282 -1.00181<br>H -3.86747 -1.19435 -0.44761<br>H -3.07499 -2.24394 -1.52691	C_1	-967.57568 (10.1)	-967.39775 (9.6)	-967.45402 (9.1)	-968.28328 (6.8)	-968.16163 (5.7)
6-adduct, conf6	C 1.61204 -1.06995 0.63612<br>C 2.22479 -0.14691 -0.41948<br>C 1.59463 1.16927 -0.60333	C_1	-967.57581 (10.0)	-967.39754 (9.8)	-967.45344 (9.4)	-968.28185 (7.7)	-968.15948 (7.1)

	C 0.48711 1.58389 0.04506<br>C -0.20385 0.69429 1.04586<br>C 0.46002 -0.59752 1.32854<br>H 2.09859 1.82668 -1.30837<br>H -0.29884 1.25644 1.98916<br>H 0.04055 -1.23621 2.10152<br>O 2.12289 -2.16639 0.89071<br>O 3.20501 -0.48757 -1.05038<br>C -0.08177 2.95315 -0.14950<br>H -0.08835 3.50408 0.79937<br>H 0.50131 3.52393 -0.87468<br>H -1.12137 2.90185 -0.49104<br>S -2.00788 0.41277 0.69964<br>C -1.96700 -0.72851 -0.71094<br>N -1.16574 -0.73869 -1.69518<br>H -0.51377 0.03841 -1.69556<br>N -3.03986 -1.57467 -0.64018<br>H -3.34227 -1.89135 0.26766<br>H -3.08952 -2.24918 -1.39144						
6-adduct, conf7	C 1.54146 -0.97558 0.75702<br>C 2.11732 -0.17043 -0.41218<br>C 1.54070 1.16082 -0.65805<br>C 0.45593 1.64346 -0.01748<br>C -0.27130 0.81188 1.00315<br>C 0.40019 -0.43627 1.41928<br>H 2.06133 1.76352 -1.39944<br>H -0.45120 1.43369 1.89393<br>H -0.02087 -1.00276 2.24573<br>O 2.06933 -2.03538 1.10818<br>O 3.02574 -0.61402 -1.08718<br>C -0.06714 3.02375 -0.25763<br>H 0.01689 3.62399 0.65725<br>H 0.48847 3.52945 -1.04981<br>H -1.12894 3.00020 -0.52597<br>S -2.05012 0.45775 0.55194<br>C -1.98418 -0.92549 -0.58328<br>N -0.88741 -0.99375 -1.40432<br>H -0.43730 -0.13755 -1.69393<br>H -0.86993 -1.74089 -2.08394<br>N -2.97568 -1.71758 -0.49622<br>H -2.97330 -2.41210 -1.24414	C_1	-967.57143 (12.7)	-967.39360 (12.3)	-967.44888 (12.3)	-968.27894 (9.5)	-968.15638 (9.0)
6-adduct, conf8	C 1.51508 -0.99383 0.75791<br>C 2.10697 -0.19073 -0.40459	C_1	-967.57666 (9.5)	-967.39837 (9.3)	-967.45354 (9.4)	-968.28352 (6.6)	-968.16040 (6.5)

	C 1.55598 1.15340 -0.63845<br>C 0.47670 1.64844 0.00017<br>C -0.27025 0.81985 1.00828<br>C 0.37912 -0.44069 1.42014<br>H 2.09181 1.75461 -1.37008<br>H -0.46138 1.43603 1.90010<br>H -0.05025 -1.00483 2.24390<br>O 2.02723 -2.05994 1.11015<br>O 3.01211 -0.64279 -1.07739<br>C -0.02097 3.04036 -0.22812<br>H 0.05809 3.62774 0.69549<br>H 0.55471 3.54721 -1.00507<br>H -1.07841 3.03851 -0.51470<br>S -2.04481 0.48606 0.50728<br>C -1.97307 -0.92758 -0.60961<br>N -0.88928 -0.98103 -1.44238<br>H -0.52258 -0.11536 -1.80922<br>H -0.91864 -1.75266 -2.09640<br>N -2.87690 -1.82164 -0.61710<br>H -3.52527 -1.72277 0.15935						
6-adduct, conf9	C 2.14147 -1.13379 0.21233<br>C 2.76078 0.21959 -0.14671<br>C 1.87606 1.38922 -0.10468<br>C 0.54930 1.33256 0.13047<br>C -0.13678 0.02114 0.38722<br>C 0.74294 -1.16176 0.47845<br>H 2.36560 2.34589 -0.27182<br>H -0.77679 0.10004 1.28908<br>H 0.29960 -2.11816 0.74295<br>O 2.84262 -2.15146 0.27861<br>O 3.94011 0.30465 -0.43146<br>C -0.30141 2.56082 0.18922<br>H -0.82802 2.61613 1.14910<br>H 0.29941 3.46349 0.06164<br>H -1.07072 2.53868 -0.59054<br>S -1.42352 -0.32010 -0.89690<br>C -2.89242 -0.32464 0.12984<br>N -2.81097 -0.04240 1.36897<br>H -3.70643 -0.07855 1.85134<br>N -4.00897 -0.60406 -0.61067<br>H -3.90530 -1.05436 -1.50722<br>H -4.86303 -0.81076 -0.11623	C_1	-967.58018 (7.3)	-967.40225 (6.8)	-967.45888 (6.0)	-968.28365 (6.5)	-968.16235 (5.3)

6-adduct, conf10	C 1.82725 -1.25069 0.34281<br>C 2.65951 -0.06992 -0.16660<br>C 2.02111 1.25389 -0.14910<br>C 0.72841 1.46667 0.16661<br>C -0.18580 0.31839 0.48584<br>C 0.47164 -0.98684 0.69552<br>H 2.67453 2.08681 -0.39809<br>H -0.83893 0.56687 1.33107<br>H -0.12004 -1.81421 1.07910<br>O 2.33370 -2.37116 0.46538<br>O 3.80537 -0.23145 -0.53698<br>C 0.13306 2.83825 0.21493<br>H -0.22956 3.06198 1.22629<br>H 0.86576 3.59912 -0.06095<br>H -0.72392 2.91799 -0.46321<br>S -1.36888 0.09213 -0.95592<br>C -2.86002 -0.48943 -0.12138<br>N -3.50569 -1.52322 -0.47960<br>H -3.01023 -2.05005 -1.19406<br>N -3.24998 0.27401 0.95658<br>H -3.18046 1.27530 0.84279<br>H -4.12888 -0.03450 1.35394	C_1	-967.57553 (10.2)	-967.39684 (10.2)	-967.45387 (9.1)	-968.28009 (8.8)	-968.15844 (7.7)
6-adduct, conf11	C 1.65762 -1.34039 0.31720<br>C 2.59978 -0.26348 -0.22951<br>C 2.12529 1.12700 -0.18189<br>C 0.88341 1.49189 0.19060<br>C -0.14496 0.45601 0.55776<br>C 0.36292 -0.92008 0.73970<br>H 2.86110 1.87736 -0.46154<br>H -0.69566 0.79514 1.44409<br>H -0.30472 -1.67075 1.15544<br>O 2.02994 -2.51517 0.40836<br>O 3.69943 -0.55826 -0.65355<br>C 0.45433 2.92295 0.26487<br>H 0.19117 3.18886 1.29652<br>H 1.24981 3.59219 -0.06831<br>H -0.43587 3.10173 -0.34797<br>S -1.38822 0.39319 -0.82473<br>C -2.81457 -0.34237 0.01864<br>N -3.54951 -1.06513 -0.87611<br>H -3.07050 -1.54834 -1.61963<br>H -4.36865 -1.51507 -0.49231<br>N -3.18611 -0.19301 1.22563	C_1	-967.57639 (9.6)	-967.39808 (9.4)	-967.45481 (8.6)	-968.28103 (8.2)	-968.15945 (7.1)

	H -2.64373 0.49318 1.74018						
6-adduct, conf12	C 2.03592 -1.18295 0.12377<br>C 2.71558 0.14728 -0.21351<br>C 1.92687 1.36958 -0.00811<br>C 0.62526 1.38405 0.33976<br>C -0.14609 0.10241 0.50713<br>C 0.66512 -1.13570 0.51050<br>H 2.47293 2.30071 -0.14022<br>H -0.76603 0.16941 1.41253<br>H 0.18461 -2.08046 0.75305<br>O 2.66939 -2.24328 0.08193<br>O 3.86582 0.17455 -0.60349<br>C -0.12175 2.65506 0.58956<br>H -0.47458 2.69318 1.62783<br>H 0.50922 3.52730 0.40864<br>H -1.00664 2.72229 -0.05232<br>S -1.38057 -0.04876 -0.87376<br>C -2.89284 -0.45936 0.02895<br>N -3.01967 -1.00682 1.17053<br>H -2.15009 -1.33830 1.57444<br>N -3.97954 -0.16547 -0.74487<br>H -3.94400 0.63152 -1.36025<br>H -4.87191 -0.37187 -0.31909	C_1	-967.57572 (10.1)	-967.39748 (9.8)	-967.45379 (9.2)	-968.28007 (8.8)	-968.15814 (7.9)
6-adduct, conf13	C 2.16279 -1.11923 0.20969<br>C 2.76212 0.24421 -0.14451<br>C 1.85828 1.39920 -0.10658<br>C 0.53186 1.32116 0.12418<br>C -0.13391 -0.00050 0.38129<br>C 0.76361 -1.17039 0.46913<br>H 2.33290 2.36361 -0.27230<br>H -0.77032 0.07004 1.28702<br>H 0.33406 -2.13462 0.72789<br>O 2.87986 -2.12572 0.27697<br>O 3.94139 0.34898 -0.42294<br>C -0.33772 2.53644 0.17843<br>H -0.87787 2.57892 1.13128<br>H 0.25130 3.44839 0.06216<br>H -1.09596 2.50653 -0.61194<br>S -1.42429 -0.36556 -0.89178<br>C -2.89296 -0.32091 0.13447<br>N -4.00611 -0.66502 -0.57957<br>H -3.96596 -0.71959 -1.58516<br>H -4.91205 -0.48285 -0.17789	C_1	-967.57993 (7.4)	-967.40212 (6.9)	-967.45914 (5.8)	-968.28336 (6.7)	-968.16256 (5.1)

	N -2.80321 -0.02860 1.37114<br>H -3.69681 -0.05208 1.85759						
6-adduct, conf14	C 2.19180 -1.06827 0.11578<br>C 2.71200 0.34847 -0.14495<br>C 1.76561 1.45207 0.05761<br>C 0.45931 1.28901 0.34866<br>C -0.14662 -0.08207 0.45644<br>C 0.81100 -1.20680 0.44074<br>H 2.19133 2.44889 -0.03030<br>H -0.79234 -0.13955 1.34394<br>H 0.44617 -2.21116 0.64021<br>O 2.95354 -2.04063 0.06708<br>O 3.86637 0.53232 -0.47722<br>C -0.45532 2.44762 0.58565<br>H -0.88614 2.39833 1.59336<br>H 0.07300 3.39721 0.48138<br>H -1.29358 2.43561 -0.11963<br>S -1.34951 -0.35971 -0.95575<br>C -2.95248 -0.34380 -0.13367<br>N -3.00440 -1.02363 1.06262<br>H -2.53091 -1.91588 1.09050<br>H -3.93919 -1.04719 1.45200<br>N -3.96881 0.25390 -0.60841<br>H -3.73459 0.82640 -1.41535	C_1	-967.57565 (10.1)	-967.39708 (10.1)	-967.45397 (9.1)	-968.27996 (8.8)	-968.15829 (7.8)
6-adduct, conf15	C 1.95041 -1.20916 0.31499<br>C 2.70575 0.04012 -0.14588<br>C 1.96527 1.30968 -0.13654<br>C 0.65239 1.41936 0.14626<br>C -0.17945 0.20478 0.44414<br>C 0.56840 -1.05702 0.62528<br>H 2.55857 2.19241 -0.36396<br>H -0.82818 0.39337 1.30920<br>H 0.02738 -1.93841 0.95953<br>O 2.53807 -2.29020 0.43625<br>O 3.87368 -0.02450 -0.47520<br>C -0.04416 2.74333 0.18109<br>H -0.47582 2.92465 1.17397<br>H 0.64453 3.55999 -0.04409<br>H -0.86411 2.77217 -0.54562<br>S -1.37747 -0.09335 -0.96718<br>C -2.89275 -0.47614 -0.09371<br>N -3.20391 0.39394 0.93697<br>H -3.00237 1.37077 0.77465	C_1	-967.56821 (14.8)	-967.39006 (14.5)	-967.44746 (13.2)	-968.27363 (12.8)	-968.15289 (11.2)

	H -4.09309 0.23504 1.39182<br>N -3.52922 -1.50451 -0.48629<br>H -4.44460 -1.57444 -0.03752						
6-adduct, conf16	C 2.23479 -1.03236 0.11845<br>C 2.70587 0.40594 -0.11814<br>C 1.71318 1.46985 0.06739<br>C 0.40977 1.25511 0.33854<br>C -0.14388 -0.13920 0.43761<br>C 0.85519 -1.22717 0.41829<br>H 2.09881 2.48304 -0.01790<br>H -0.77838 -0.22808 1.33075<br>H 0.52567 -2.24693 0.60053<br>O 3.03297 -1.97548 0.07196<br>O 3.86077 0.63508 -0.42035<br>C -0.55816 2.37454 0.55037<br>H -0.99633 2.31715 1.55493<br>H -0.07000 3.34504 0.44261<br>H -1.38797 2.31599 -0.16328<br>S -1.33903 -0.44150 -0.97328<br>C -2.92200 -0.31387 -0.14236<br>N -3.03767 -1.09109 0.99401<br>H -2.58558 -1.99428 0.97113<br>H -3.94641 -1.09967 1.43766<br>N -3.77203 0.48257 -0.65315<br>H -4.68591 0.39758 -0.20372	C_1	-967.56945 (14.0)	-967.39132 (13.7)	-967.44793 (12.9)	-968.27490 (12.0)	-968.15338 (10.9)
5-adduct, conf1	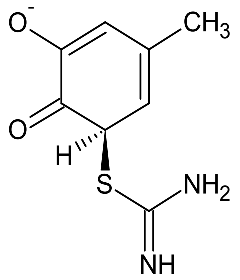<br>(5*R*)-5-(carbamimidoylsulfanyl)-3-methyl-6-oxocyclohexa-1,3-dien-1-olate<br><br>C 0.37799 1.34541 -0.63879<br>C 1.51017 1.18669 0.39137<br>C 2.09493 -0.11881 0.53741<br>C 1.65344 -1.25405 -0.16697<br>C 0.59533 -1.16352 -1.04864<br>C -0.16617 0.08575 -1.30601	C_1	-967.58047 (7.1)	-967.40233 (6.8)	-967.45855 (6.2)	-968.28640 (4.8)	-968.16448 (3.9)

	H 2.91806 -0.19659 1.24386<br>H 0.26093 -2.05000 -1.58105<br>H -0.19129 0.30118 -2.38369<br>O -0.05640 2.44007 -0.90985<br>O 1.87091 2.16395 1.03942<br>C 2.35268 -2.56733 0.05361<br>H 3.41983 -2.48338 -0.17684<br>H 2.26821 -2.87830 1.10027<br>H 1.92853 -3.35812 -0.56908<br>S -1.96550 -0.19302 -0.97478<br>C -1.96330 -0.42498 0.80326<br>N -2.48268 -1.49690 1.25259<br>H -2.55240 -1.47321 2.27238<br>N -1.37176 0.60636 1.49941<br>H -1.47090 1.54258 1.12983<br>H -1.39828 0.55090 2.50861						
5-adduct, conf2	C -0.40080 -1.34718 -0.62603<br>C -1.55039 -1.15305 0.37846<br>C -2.09298 0.17192 0.51886<br>C -1.61030 1.29066 -0.18535<br>C -0.54913 1.16487 -1.05847<br>C 0.17916 -0.10650 -1.30260<br>H -2.92032 0.27734 1.21652<br>H -0.19255 2.03721 -1.60026<br>H 0.20581 -0.33231 -2.37804<br>O 0.01731 -2.45223 -0.87672<br>O -1.96143 -2.11995 1.01033<br>C -2.27042 2.62533 0.02709<br>H -3.34522 2.56487 -0.17208<br>H -2.14812 2.95268 1.06508<br>H -1.84436 3.39341 -0.62239<br>S 1.98425 0.10922 -0.95008<br>C 1.95784 0.38835 0.83406<br>N 2.47893 1.39949 1.40326<br>H 2.79600 2.08440 0.72273<br>N 1.30709 -0.59129 1.54120<br>H 1.42074 -1.54602 1.22878<br>H 1.35299 -0.46544 2.54464	C_1	-967.58653 (3.3)	-967.40792 (3.3)	-967.46388 (2.9)	-968.29181 (1.4)	-968.16917 (1.0)
5-adduct, conf3	C -0.23472 -1.22467 -0.66404<br>C -1.46470 -1.35466 0.24967<br>C -2.25146 -0.17148 0.49217<br>C -1.91846 1.10150 0.00062	C_1	-967.58636 (3.4)	-967.40847 (2.9)	-967.46414 (2.7)	-968.29166 (1.5)	-968.16944 (0.8)

	C -0.79462 1.27970 -0.77758<br>C 0.10151 0.17166 -1.21056<br>H -3.13176 -0.30742 1.11606<br>H -0.54364 2.27355 -1.14010<br>H 0.04609 0.07220 -2.30604<br>O 0.38014 -2.19832 -1.02100<br>O -1.74768 -2.44883 0.72292<br>C -2.80067 2.27111 0.34496<br>H -3.83168 2.09006 0.02369<br>H -2.82036 2.43338 1.42801<br>H -2.45275 3.19190 -0.12939<br>S 1.86755 0.58702 -0.98009<br>C 1.99164 0.29322 0.76955<br>N 3.24015 0.58103 1.26384<br>H 4.01688 0.60359 0.62020<br>H 3.46272 0.20699 2.17413<br>N 0.96127 -0.10832 1.40201<br>H 1.13302 -0.28644 2.38984						
5-adduct, conf4	C -1.03912 -1.24170 -0.52446<br>C -1.77324 -0.47649 0.58772<br>C -1.68172 0.95930 0.55046<br>C -0.90140 1.66572 -0.38648<br>C -0.10855 0.99024 -1.29246<br>C 0.02857 -0.48557 -1.30545<br>H -2.26743 1.49580 1.29279<br>H 0.49264 1.54686 -2.00679<br>H 0.01595 -0.88362 -2.32757<br>O -1.31187 -2.38982 -0.77819<br>O -2.42265 -1.09550 1.42344<br>C -0.93644 3.16872 -0.37267<br>H -1.96296 3.53685 -0.46792<br>H -0.53862 3.54942 0.57418<br>H -0.34266 3.59323 -1.18547<br>S 1.73039 -0.99743 -0.75915<br>C 1.83994 -0.14173 0.84673<br>N 2.65401 0.95277 0.79508<br>H 3.24573 1.08943 -0.00591<br>H 2.95516 1.33219 1.68096<br>N 1.24839 -0.48329 1.91851<br>H 0.70733 -1.33593 1.81479	C_1	-967.58247 (5.8)	-967.40409 (5.7)	-967.46189 (4.1)	-968.28760 (4.0)	-968.16702 (2.3)
5-adduct, conf5	C 0.10214 1.17202 -0.75890<br>C 1.29030 1.47409 0.17359<br>C 2.19245 0.39193 0.48531	C_1	-967.57666 (9.5)	-967.39882 (9.0)	-967.45443 (8.8)	-968.28302 (6.9)	-968.16079 (6.3)

	C 2.01688 -0.92754 0.02621<br>C 0.92775 -1.25426 -0.75789<br>C -0.10397 -0.27855 -1.20691<br>H 3.04374 0.64748 1.11216<br>H 0.80593 -2.27696 -1.10706<br>H -0.10071 -0.24344 -2.30698<br>O -0.60505 2.06224 -1.15663<br>O 1.44527 2.61000 0.60441<br>C 3.03884 -1.97088 0.39062<br>H 4.01323 -1.72069 -0.04223<br>H 3.17293 -2.02927 1.47580<br>H 2.75117 -2.96032 0.02770<br>S -1.82050 -0.88002 -0.89759<br>C -2.12345 -0.39749 0.79198<br>N -0.99993 -0.21528 1.57533<br>H -0.25028 -0.88541 1.46759<br>H -1.17895 0.04689 2.53542<br>N -3.34616 -0.22438 1.09095<br>H -3.47388 -0.05426 2.08872						
5-adduct, conf6	C 0.05670 1.14064 -0.76031<br>C 1.26073 1.50309 0.12825<br>C 2.19343 0.45374 0.45792<br>C 2.05153 -0.88028 0.03123<br>C 0.96788 -1.25617 -0.73681<br>C -0.09534 -0.32060 -1.19626<br>H 3.04302 0.74795 1.06979<br>H 0.87458 -2.28912 -1.06384<br>H -0.09922 -0.29761 -2.29664<br>O -0.70866 1.99199 -1.13542<br>O 1.39821 2.65775 0.51221<br>C 3.10515 -1.88519 0.41250<br>H 4.07316 -1.60986 -0.01931<br>H 3.23555 -1.92443 1.49904<br>H 2.85086 -2.88829 0.06225<br>S -1.78911 -0.97175 -0.85367<br>C -2.11169 -0.39578 0.81475<br>N -1.00013 -0.25406 1.61253<br>H -0.30691 -0.98792 1.56629<br>H -1.23370 0.04352 2.55193<br>N -3.27663 -0.10397 1.22895<br>H -3.96102 -0.09463 0.47698	C_1	-967.58331 (5.3)	-967.40504 (5.1)	-967.46066 (4.9)	-968.28870 (3.4)	-968.16606 (2.9)
5-adduct, conf7	C -0.75329 -1.35780 -0.53572<br>C -1.72772 -0.81924 0.52638	C_1	-967.58258 (5.8)	-967.40430 (5.5)	-967.46174 (4.2)	-968.28732 (4.2)	-968.16648 (2.7)

	C -1.95097 0.60123 0.55291<br>C -1.29358 1.51014 -0.29940<br>C -0.33925 1.06906 -1.19235<br>C 0.10015 -0.34545 -1.29584<br>H -2.67850 0.95651 1.27852<br>H 0.15438 1.77992 -1.85051<br>H 0.10322 -0.67671 -2.34305<br>O -0.68321 -2.53996 -0.76797<br>O -2.28317 -1.60567 1.28584<br>C -1.64997 2.96906 -0.21785<br>H -2.71932 3.11975 -0.39794<br>H -1.42924 3.36230 0.78025<br>H -1.09389 3.56145 -0.94829<br>S 1.89860 -0.49639 -0.89269<br>C 1.91281 0.08287 0.83151<br>N 1.27132 -0.40968 1.81274<br>H 0.79511 -1.27776 1.58938<br>N 2.80296 1.10203 0.99611<br>H 3.02132 1.70394 0.22053<br>H 2.89116 1.47219 1.93131						
5-adduct, conf8	C -0.16723 1.00020 0.68662<br>C -1.34511 1.50764 -0.15956<br>C -2.40960 0.56051 -0.39350<br>C -2.36377 -0.78049 0.03675<br>C -1.21113 -1.29137 0.60176<br>C 0.04248 -0.50187 0.70640<br>H -3.29605 0.94898 -0.88931<br>H -1.17645 -2.33031 0.91941<br>H 0.63639 -0.76660 1.58526<br>O 0.54248 1.75678 1.30401<br>O -1.38752 2.67383 -0.52997<br>C -3.58099 -1.64555 -0.14001<br>H -4.44210 -1.21852 0.38425<br>H -3.84858 -1.71983 -1.19941<br>H -3.41563 -2.65645 0.23977<br>S 1.04480 -0.91470 -0.80710<br>C 2.67686 -0.33135 -0.32932<br>N 3.24844 0.47046 -1.13194<br>H 4.21161 0.66132 -0.84857<br>N 3.12399 -0.82702 0.87573<br>H 2.85848 -1.77071 1.11430<br>H 4.05978 -0.56798 1.15430	C_1	-967.57693 (9.3)	-967.39894 (8.9)	-967.45599 (7.8)	-968.28125 (8.0)	-968.16031 (6.6)

5-adduct, conf9	C -0.20954 1.01370 0.69300<br>C -1.41388 1.48890 -0.13430<br>C -2.44990 0.50955 -0.36009<br>C -2.34342 -0.83725 0.03914<br>C -1.15910 -1.31572 0.56797<br>C 0.06070 -0.47801 0.67500<br>H -3.35984 0.87337 -0.83151<br>H -1.07612 -2.36018 0.85700<br>H 0.69098 -0.74296 1.52879<br>O 0.47749 1.78671 1.31547<br>O -1.49167 2.65360 -0.50522<br>C -3.52857 -1.74619 -0.13449<br>H -4.39413 -1.36683 0.41828<br>H -3.81644 -1.80550 -1.18951<br>H -3.31513 -2.75840 0.21650<br>S 1.07372 -0.79964 -0.85719<br>C 2.72750 -0.30810 -0.29795<br>N 3.42723 0.58602 -0.86450<br>H 2.89775 1.09649 -1.56559<br>N 3.16643 -0.99723 0.80509<br>H 2.93275 -1.97745 0.85430<br>H 4.11572 -0.76963 1.07111	C_1	-967.58432 (4.7)	-967.40575 (4.6)	-967.46260 (3.7)	-968.28730 (4.2)	-968.16558 (3.2)
5-adduct, conf10	C -0.42692 1.15318 -0.14764<br>C -1.93265 1.38160 0.07786<br>C -2.77396 0.21555 0.11117<br>C -2.28320 -1.10102 0.06107<br>C -0.92277 -1.33786 0.03116<br>C 0.09260 -0.25789 0.10170<br>H -3.84455 0.40110 0.15939<br>H -0.55110 -2.35882 0.00919<br>H 0.51999 -0.22508 1.13417<br>O 0.29191 2.06552 -0.47008<br>O -2.36354 2.52764 0.16324<br>C -3.25809 -2.24695 0.06691<br>H -3.85891 -2.24056 0.98257<br>H -3.95092 -2.17203 -0.77772<br>H -2.74659 -3.21023 0.00372<br>S 1.57326 -0.64941 -0.87291<br>C 2.81394 -0.25181 0.35443<br>N 4.05775 -0.16442 -0.21238<br>H 4.11550 0.13481 -1.17454<br>H 4.81369 0.14756 0.37832<br>N 2.48459 -0.16014 1.58059	C_1	-967.58746 (2.7)	-967.40988 (2.0)	-967.46646 (1.2)	-968.28817 (3.7)	-968.16716 (2.3)

	H 3.25357 0.11183 2.18879						
5-adduct, conf11	C -0.12814 0.93291 0.73253<br>C -1.26786 1.51145 -0.12008<br>C -2.38556 0.63106 -0.36026<br>C -2.40103 -0.72315 0.02841<br>C -1.26957 -1.30545 0.56806<br>C 0.01488 -0.57391 0.70627<br>H -3.25311 1.07545 -0.84234<br>H -1.27866 -2.35574 0.84718<br>H 0.59977 -0.90892 1.56774<br>O 0.61867 1.64582 1.36017<br>O -1.22659 2.67555 -0.49989<br>C -3.65649 -1.52585 -0.17107<br>H -4.49676 -1.07384 0.36582<br>H -3.92713 -1.55838 -1.23182<br>H -3.53824 -2.55326 0.18055<br>S 1.01723 -0.92592 -0.82248<br>C 2.67217 -0.46904 -0.18990<br>N 3.18356 0.61383 -0.83851<br>H 2.54254 1.30775 -1.18927<br>H 4.06215 0.95482 -0.47287<br>N 3.32450 -1.11934 0.68528<br>H 2.85025 -1.97365 0.96118	C_1	-967.58498<br>(4.2)	-967.40655<br>(4.1)	-967.46429<br>(2.6)	-968.28837<br>(3.6)	-968.16768<br>(1.9)
5-adduct, conf12	C -0.36983 1.17376 0.41597<br>C -1.66778 1.35874 -0.38314<br>C -2.56667 0.23274 -0.40979<br>C -2.27353 -1.01208 0.18237<br>C -1.03071 -1.24534 0.73698<br>C 0.07343 -0.25170 0.68990<br>H -3.52646 0.39839 -0.89332<br>H -0.80236 -2.21357 1.17436<br>H 0.67739 -0.26793 1.60426<br>O 0.26954 2.12538 0.80277<br>O -1.91590 2.43701 -0.90906<br>C -3.32896 -2.08287 0.18705<br>H -4.23230 -1.73659 0.69958<br>H -3.61546 -2.34432 -0.83718<br>H -2.97987 -2.99064 0.68427<br>S 1.20249 -0.76874 -0.67995<br>C 2.80716 -0.21252 -0.03163<br>N 3.04452 0.73621 0.77203<br>H 2.23900 1.32131 0.98658<br>N 3.80374 -0.95017 -0.61943	C_1	-967.58856<br>(2.0)	-967.41003<br>(1.9)	-967.46565<br>(1.8)	-968.29134<br>(1.7)	-968.16843<br>(1.5)

	H 3.64202 -1.93352 -0.77298<br>H 4.73275 -0.69908 -0.31043						
5-adduct, conf13	C -0.31129 1.14923 0.37788<br>C -1.63870 1.39209 -0.35238<br>C -2.57081 0.29636 -0.37194<br>C -2.30012 -0.96912 0.18540<br>C -1.05506 -1.24545 0.71406<br>C 0.07555 -0.28449 0.67104<br>H -3.53732 0.50008 -0.82661<br>H -0.84392 -2.22851 1.12603<br>H 0.66955 -0.32233 1.59105<br>O 0.40428 2.08023 0.68721<br>O -1.87150 2.49265 -0.84032<br>C -3.38283 -2.01216 0.18592<br>H -4.27078 -1.65168 0.71533<br>H -3.68789 -2.24927 -0.83886<br>H -3.05080 -2.93676 0.66329<br>S 1.19755 -0.86176 -0.69161<br>C 2.83498 -0.37824 -0.13987<br>N 2.90637 0.59819 0.81590<br>H 2.17875 1.30992 0.85388<br>H 3.83613 0.91919 1.04550<br>N 3.76296 -1.04688 -0.70019<br>H 4.68773 -0.68401 -0.46964	C_1	-967.58700 (3.0)	-967.40870 (2.8)	-967.46386 (2.9)	-968.29003 (2.5)	-968.16690 (2.4)
5-adduct, conf14	C -0.33395 1.15802 0.35978<br>C -1.68006 1.37954 -0.34325<br>C -2.59263 0.26693 -0.35383<br>C -2.29001 -0.99678 0.19046<br>C -1.03243 -1.25369 0.69960<br>C 0.07785 -0.27073 0.65350<br>H -3.57011 0.45595 -0.79107<br>H -0.79965 -2.23429 1.10588<br>H 0.67948 -0.29367 1.56929<br>O 0.37676 2.09743 0.64898<br>O -1.94152 2.47724 -0.82265<br>C -3.35355 -2.05937 0.20106<br>H -4.23889 -1.71855 0.74753<br>H -3.67161 -2.29478 -0.82017<br>H -2.99745 -2.98108 0.66653<br>S 1.22314 -0.81705 -0.70540<br>C 2.86811 -0.35391 -0.12243<br>N 2.92018 0.61620 0.83262<br>H 2.21780 1.35064 0.82979	C_1	-967.59174 (0.0)	-967.41313 (0.0)	-967.46844 (0.0)	-968.29405 (0.0)	-968.17075 (0.0)

	H 3.86349 0.89944 1.06129<br>N 3.90759 -0.93090 -0.57946<br>H 3.69042 -1.72537 -1.17472						
5-adduct, conf15	C 1.18505 -1.24528 0.47364<br>C 2.30156 -0.58490 -0.34641<br>C 2.28734 0.85329 -0.40824<br>C 1.26611 1.64487 0.14779<br>C 0.15179 1.05478 0.71439<br>C -0.07174 -0.41407 0.72654<br>H 3.14114 1.31898 -0.89485<br>H -0.63851 1.67568 1.12834<br>H -0.49467 -0.76800 1.67272<br>O 1.29834 -2.36404 0.90786<br>O 3.16973 -1.27960 -0.86350<br>C 1.39341 3.14254 0.09657<br>H 2.31818 3.47188 0.58118<br>H 1.42803 3.49041 -0.94152<br>H 0.55350 3.63491 0.59186<br>S -1.25462 -0.89398 -0.61493<br>C -2.74587 -0.04410 -0.04607<br>N -3.10921 0.20275 1.14809<br>H -2.50766 -0.21897 1.84845<br>N -3.54169 0.25444 -1.11474<br>H -3.11120 0.48486 -1.99583<br>H -4.40906 0.71984 -0.88904	C_1	-967.58346 (5.2)	-967.40517 (5.0)	-967.46236 (3.8)	-968.28692 (4.5)	-968.16582 (3.1)
5-adduct, conf16	C 1.19413 -1.24744 0.45053<br>C 2.30095 -0.51952 -0.32488<br>C 2.23422 0.91884 -0.33846<br>C 1.17739 1.65287 0.22953<br>C 0.07876 1.00030 0.75791<br>C -0.09332 -0.47188 0.70293<br>H 3.07764 1.43193 -0.79451<br>H -0.73881 1.57128 1.19059<br>H -0.54824 -0.86965 1.61352<br>O 1.33784 -2.37806 0.84590<br>O 3.20426 -1.16367 -0.84731<br>C 1.25015 3.15482 0.23567<br>H 2.16039 3.49987 0.73653<br>H 1.27475 3.54192 -0.78873<br>H 0.39008 3.59696 0.74351<br>S -1.21199 -0.92369 -0.72220<br>C -2.71104 -0.02706 -0.27735<br>N -3.10044 -0.17080 1.03445	C_1	-967.58363 (5.1)	-967.40495 (5.1)	-967.46252 (3.7)	-968.28749 (4.1)	-968.16638 (2.7)

	H -2.97362 -1.08639 1.44184<br>H -4.00713 0.24037 1.21924<br>N -3.36965 0.68815 -1.09627<br>H -2.87668 0.79964 -1.97805						

Table S10.6. One-electron oxidized neutral form in water.

Structure	Schematic drawing	Symmetry	G_{PCM}	$H_{PCM,RRHO}$	$G_{PCM,RRHO}$	$G_{SMD,M06-2X,large}$	$G_{SMD,RRHO,M06-2X,large}$
6-adduct, conf1	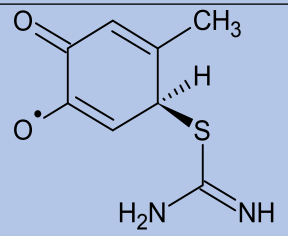<br>[(3*S*)-3-(carbamimidoylsulfanyl)-4-methyl-6-oxocyclohexa-1,4-dien-1-yl]oxidanyl<br><br>C 1.87871 0.42904 -0.62924<br>C 1.88880 -0.42960 0.63644<br>C 0.84110 -1.43326 0.77663<br>C -0.19939 -1.55868 -0.07694<br>C -0.37721 -0.59645 -1.21404<br>C 0.76956 0.28086 -1.50829<br>H 0.94718 -2.11631 1.61579<br>H -0.67526 -1.13681 -2.12233<br>H 0.75334 0.86922 -2.42176<br>O 2.82476 1.19382 -0.86861<br>O 2.78125 -0.28393 1.46075<br>C -1.21632 -2.64148 0.06481<br>H -1.21052 -3.27882 -0.82773<br>H -1.01544 -3.26234 0.93943<br>H -2.22378 -2.22151 0.14950<br>S -1.90430 0.45954 -0.95115<br>C -1.58711 1.13743 0.68675<br>N -2.37025 0.76339 1.62462<br>H -2.17100 1.27410 2.48782<br>N -0.50916 1.97286 0.75748<br>H -0.26729 2.50665 -0.06452<br>H -0.34950 2.45329 1.63386	C_1	-967.59715 (8.6)	-967.41884 (8.8)	-967.47432 (9.6)	-968.31284 (5.3)	-968.19001 (5.5)

6-adduct, conf2	C 1.87935 0.41295 -0.63255<br>C 1.87727 -0.43112 0.64339<br>C 0.82442 -1.42950 0.78764<br>C -0.21098 -1.55864 -0.07117<br>C -0.38345 -0.59894 -1.21103<br>C 0.77167 0.26412 -1.51415<br>H 0.92600 -2.10808 1.63095<br>H -0.69563 -1.13685 -2.11591<br>H 0.76178 0.84371 -2.43324<br>O 2.83252 1.16632 -0.87753<br>O 2.76660 -0.28261 1.47013<br>C -1.22130 -2.64937 0.06008<br>H -1.14557 -3.32739 -0.79909<br>H -1.06687 -3.22717 0.97279<br>H -2.24007 -2.24897 0.05796<br>S -1.89654 0.47931 -0.94223<br>C -1.56406 1.17081 0.69755<br>N -2.29780 0.92339 1.71261<br>H -3.00061 0.22160 1.49692<br>N -0.45804 1.96484 0.76687<br>H -0.21773 2.51415 -0.04489<br>H -0.31643 2.43090 1.65449	C_1	-967.60016 (6.7)	-967.42160 (7.1)	-967.47663 (8.2)	-968.31463 (4.1)	-968.19110 (4.8)
6-adduct, conf3	C 1.80158 0.76126 -0.66686<br>C 2.10576 -0.07062 0.57818<br>C 1.31129 -1.27201 0.79962<br>C 0.22589 -1.60047 0.06785<br>C -0.23574 -0.72510 -1.06425<br>C 0.65175 0.40123 -1.42222<br>H 1.63957 -1.91492 1.61292<br>H -0.37222 -1.35598 -1.95665<br>H 0.40686 0.99283 -2.29999<br>O 2.54885 1.69575 -0.98775<br>O 3.01663 0.26388 1.32485<br>C -0.54259 -2.85647 0.31743<br>H -0.57485 -3.47089 -0.59028<br>H -0.09128 -3.44125 1.12068<br>H -1.58105 -2.63313 0.58398<br>S -1.97309 -0.12490 -0.85211<br>C -1.80960 1.01039 0.52294<br>N -3.03304 1.34214 1.02731<br>H -3.80156 0.69486 0.93042<br>H -3.05850 1.92066 1.85502<br>N -0.66086 1.45381 0.86013	C_1	-967.60104 (6.1)	-967.42306 (6.2)	-967.47893 (6.7)	-968.31433 (4.3)	-968.19221 (4.1)

	H -0.71415 2.06813 1.67265						
6-adduct, conf4	C -1.86008 -0.46093 -0.61684<br>C -1.89432 0.39246 0.65245<br>C -0.87608 1.42589 0.79303<br>C 0.15406 1.58851 -0.06726<br>C 0.36904 0.62482 -1.19751<br>C -0.75305 -0.28381 -1.49449<br>H -1.00457 2.10792 1.62983<br>H 0.65600 1.16797 -2.10805<br>H -0.72035 -0.87189 -2.40789<br>O -2.78462 -1.25007 -0.86039<br>O -2.78400 0.22088 1.47427<br>C 1.11295 2.72666 0.04849<br>H 0.98203 3.40335 -0.80521<br>H 0.95012 3.29334 0.96676<br>H 2.15050 2.37946 0.02060<br>S 1.91999 -0.38546 -0.92197<br>C 1.45907 -1.27261 0.59857<br>N 2.23720 -0.92480 1.64670<br>H 2.78906 -0.08378 1.63114<br>H 2.08337 -1.38374 2.53294<br>N 0.54907 -2.16855 0.68325<br>H 0.16941 -2.40436 -0.22803	C_1	-967.59930 (7.2)	-967.42101 (7.5)	-967.47694 (8.0)	-968.31395 (4.6)	-968.19158 (4.5)
6-adduct, conf5	C 1.55046 -1.07074 0.66332<br>C 2.19055 -0.18028 -0.40167<br>C 1.61664 1.14565 -0.60524<br>C 0.51559 1.59986 0.03426<br>C -0.20997 0.73899 1.03270<br>C 0.41564 -0.56054 1.34944<br>H 2.14036 1.78363 -1.31276<br>H -0.31926 1.31291 1.96595<br>H -0.02260 -1.16623 2.13788<br>O 2.03719 -2.18339 0.91994<br>O 3.16088 -0.58206 -1.02787<br>C -0.00470 2.98246 -0.17353<br>H 0.01791 3.53769 0.77197<br>H 0.58917 3.52311 -0.91199<br>H -1.04932 2.96124 -0.50202<br>S -2.00523 0.46513 0.64318<br>C -1.94116 -0.74188 -0.70646<br>N -1.12114 -0.77116 -1.68534<br>H -0.50667 0.03648 -1.70350<br>N -2.98515 -1.60494 -0.60300	C_1	-967.59957 (7.0)	-967.42138 (7.2)	-967.47717 (7.8)	-968.31334 (5.0)	-968.19094 (4.9)

	H -3.34787 -1.84140 0.30758<br>H -3.05688 -2.31438 -1.31925						
6-adduct, conf6	C 1.53930 -1.00043 0.72316<br>C 2.14312 -0.15718 -0.40074<br>C 1.55919 1.15786 -0.64653<br>C 0.46444 1.62732 -0.00771<br>C -0.25265 0.78839 1.01303<br>C 0.40089 -0.47745 1.39420<br>H 2.07188 1.77385 -1.38122<br>H -0.40367 1.39543 1.91880<br>H -0.02009 -1.05120 2.21526<br>O 2.05864 -2.08277 1.03643<br>O 3.09805 -0.58266 -1.03520<br>C -0.06468 3.00175 -0.24722<br>H -0.00524 3.59010 0.67660<br>H 0.50095 3.51579 -1.02586<br>H -1.12085 2.97019 -0.53496<br>S -2.03948 0.44904 0.59948<br>C -2.01386 -0.88729 -0.59184<br>N -0.95904 -0.92977 -1.45654<br>H -0.53167 -0.06030 -1.74370<br>H -1.00038 -1.63141 -2.18443<br>N -3.00462 -1.68980 -0.49762<br>H -3.02153 -2.34448 -1.28102	C_1	-967.59724 (8.5)	-967.41923 (8.6)	-967.47471 (9.4)	-968.31209 (5.7)	-968.18957 (5.8)
6-adduct, conf7	C 1.49839 -1.01565 0.73087<br>C 2.12703 -0.17640 -0.38280<br>C 1.57652 1.15471 -0.61699<br>C 0.48359 1.63802 0.01379<br>C -0.26296 0.80164 1.01415<br>C 0.36649 -0.47437 1.39936<br>H 2.11032 1.76856 -1.33832<br>H -0.43721 1.40334 1.91867<br>H -0.07053 -1.04422 2.21471<br>O 1.99319 -2.11019 1.03985<br>O 3.07538 -0.61859 -1.01577<br>C -0.01830 3.02427 -0.21635<br>H 0.03180 3.59951 0.71618<br>H 0.57000 3.53943 -0.97722<br>H -1.06895 3.01349 -0.52568<br>S -2.04092 0.47516 0.53769<br>C -1.98162 -0.89659 -0.62652<br>N -0.93436 -0.91001 -1.49902<br>H -0.58085 -0.02744 -1.83965	C_1	-967.59992 (6.8)	-967.42158 (7.1)	-967.47673 (8.1)	-968.31369 (4.7)	-968.19050 (5.2)

	H -0.98054 -1.64228 -2.19656<br>N -2.88103 -1.80380 -0.62176<br>H -3.49514 -1.71986 0.18459						
6-adduct, conf8	C 1.79990 -1.25350 0.35150<br>C 2.63944 -0.09269 -0.18334<br>C 2.03469 1.23452 -0.17907<br>C 0.74981 1.47427 0.16293<br>C -0.17416 0.34490 0.51766<br>C 0.46150 -0.96819 0.73222<br>H 2.69089 2.05569 -0.45638<br>H -0.80806 0.61830 1.36730<br>H -0.13601 -1.77379 1.15030<br>O 2.30409 -2.38212 0.46946<br>O 3.78414 -0.29511 -0.56497<br>C 0.18127 2.85316 0.20714<br>H -0.11373 3.10162 1.23388<br>H 0.90497 3.59402 -0.13639<br>H -0.72091 2.92547 -0.40955<br>S -1.33634 0.07623 -0.92581<br>C -2.85706 -0.46482 -0.14136<br>N -3.44636 -1.43830 -0.72341<br>H -4.37016 -1.59815 -0.31788<br>N -3.22015 0.20502 0.99232<br>H -3.00777 1.19075 1.05646<br>H -4.11036 -0.04875 1.40066	*C*$_1$	-967.59678 (8.8)	-967.41835 (9.1)	-967.47399 (9.8)	-968.30924 (7.5)	-968.18644 (7.7)
6-adduct, conf9	C 1.80858 -1.24452 0.35857<br>C 2.64023 -0.08109 -0.18340<br>C 2.02669 1.24192 -0.18583<br>C 0.74015 1.47493 0.15511<br>C -0.17613 0.34137 0.51585<br>C 0.46791 -0.96631 0.73786<br>H 2.67735 2.06612 -0.46708<br>H -0.81447 0.61427 1.36268<br>H -0.12403 -1.77340 1.16093<br>O 2.31981 -2.36896 0.48280<br>O 3.78583 -0.27841 -0.56469<br>C 0.16291 2.85038 0.19306<br>H -0.14012 3.09935 1.21730<br>H 0.88430 3.59474 -0.14774<br>H -0.73570 2.91624 -0.42958<br>S -1.34597 0.06530 -0.92200<br>C -2.87403 -0.46991 -0.12443<br>N -3.56735 -1.43519 -0.59129	*C*$_1$	-967.59965 (7.0)	-967.42103 (7.4)	-967.47698 (8.0)	-968.31085 (6.5)	-968.18818 (6.6)

	H -3.07425 -1.92579 -1.33308<br>N -3.23086 0.22032 0.99621<br>H -3.04622 1.21286 1.02490<br>H -4.12690 -0.04663 1.38371						
6-adduct, conf10	C 2.15176 -1.11216 0.18879<br>C 2.74665 0.25635 -0.13898<br>C 1.85414 1.40548 -0.09067<br>C 0.52494 1.32057 0.14257<br>C -0.13688 -0.00504 0.37804<br>C 0.75737 -1.17417 0.44932<br>H 2.32109 2.37493 -0.24604<br>H -0.77707 0.04245 1.28537<br>H 0.32625 -2.13973 0.69968<br>O 2.88428 -2.11553 0.23542<br>O 3.93770 0.35335 -0.40723<br>C -0.33876 2.53554 0.21749<br>H -0.82637 2.59181 1.19767<br>H 0.24253 3.44541 0.05915<br>H -1.13719 2.49241 -0.53097<br>S -1.41516 -0.36398 -0.89937<br>C -2.88986 -0.33865 0.13219<br>N -4.00885 -0.56623 -0.60347<br>H -3.99329 -0.44245 -1.60485<br>H -4.90656 -0.43612 -0.16032<br>N -2.77928 -0.17294 1.39308<br>H -3.68188 -0.16092 1.86497	C_1	-967.60340 (4.6)	-967.42570 (4.5)	-967.48240 (4.6)	-968.31233 (5.6)	-968.19133 (4.7)
6-adduct, conf11	C 1.85663 -1.24421 0.34257<br>C 2.66380 -0.07013 -0.21370<br>C 2.03247 1.24460 -0.21004<br>C 0.74838 1.46177 0.14796<br>C -0.14734 0.31648 0.53066<br>C 0.51802 -0.98521 0.74114<br>H 2.66734 2.07632 -0.50478<br>H -0.74548 0.59109 1.40696<br>H -0.05674 -1.79901 1.17464<br>O 2.38509 -2.36131 0.46018<br>O 3.80608 -0.25243 -0.61131<br>C 0.15093 2.82836 0.18752<br>H -0.12532 3.08490 1.21756<br>H 0.85055 3.57955 -0.18232<br>H -0.76676 2.87272 -0.40842<br>S -1.35580 0.03617 -0.85246<br>C -2.89640 -0.33130 0.03404	C_1	-967.59989 (6.9)	-967.42159 (7.1)	-967.47787 (7.4)	-968.31158 (6.1)	-968.18956 (5.8)

	N -3.66364 -1.15904 -0.72280<br>H -3.21986 -1.83607 -1.32426<br>H -4.55839 -1.42112 -0.33288<br>N -3.30016 0.15609 1.14119<br>H -2.69421 0.88132 1.51228						
6-adduct, conf12	C 2.07916 -1.14189 0.14241<br>C 2.69596 0.20827 -0.22589<br>C 1.87664 1.39751 -0.02752<br>C 0.57892 1.36346 0.34601<br>C -0.13674 0.05451 0.53678<br>C 0.71870 -1.15051 0.55198<br>H 2.37680 2.35013 -0.18258<br>H -0.75658 0.10641 1.44168<br>H 0.27917 -2.10214 0.83768<br>O 2.76938 -2.17267 0.10158<br>O 3.84632 0.25786 -0.63860<br>C -0.21006 2.60423 0.59785<br>H -0.50985 2.64698 1.65213<br>H 0.36932 3.49866 0.36364<br>H -1.13016 2.61142 0.00409<br>S -1.34417 -0.17379 -0.85557<br>C -2.90973 -0.44568 0.01377<br>N -3.09467 -0.93904 1.17567<br>H -2.25027 -1.29114 1.61481<br>N -3.94174 -0.12446 -0.81229<br>H -3.83084 0.62863 -1.47436<br>H -4.86875 -0.24474 -0.42785	C_1	-967.59974 (6.9)	-967.42156 (7.1)	-967.47800 (7.3)	-968.31183 (5.9)	-968.19009 (5.4)
6-adduct, conf13	C 2.16286 -1.07555 0.13460<br>C 2.69232 0.32678 -0.16908<br>C 1.77943 1.44502 0.02584<br>C 0.47542 1.30308 0.35211<br>C -0.14632 -0.05730 0.48671<br>C 0.79183 -1.19638 0.48817<br>H 2.21065 2.43608 -0.09103<br>H -0.78988 -0.08966 1.37583<br>H 0.41439 -2.18585 0.73147<br>O 2.92831 -2.05219 0.09168<br>O 3.85205 0.47206 -0.53168<br>C -0.41368 2.47558 0.59855<br>H -0.76947 2.46312 1.63612<br>H 0.10898 3.41668 0.42011<br>H -1.30080 2.43638 -0.04291<br>S -1.32549 -0.33242 -0.93788	C_1	-967.59672 (8.8)	-967.41837 (9.1)	-967.47399 (9.8)	-968.30926 (7.5)	-968.18654 (7.7)

	C -2.94102 -0.34749 -0.15845<br>N -3.01897 -0.96062 1.05974<br>H -2.44867 -1.77857 1.22293<br>H -3.93841 -1.01407 1.47854<br>N -3.85952 0.24087 -0.82358<br>H -4.77732 0.07144 -0.40871						
6-adduct, conf14	C 2.15747 -1.07724 0.12936<br>C 2.68709 0.32489 -0.17503<br>C 1.77821 1.44470 0.03143<br>C 0.47612 1.30477 0.36563<br>C -0.14643 -0.05516 0.49990<br>C 0.78887 -1.19604 0.49322<br>H 2.21110 2.43517 -0.08415<br>H -0.79268 -0.09086 1.38675<br>H 0.41057 -2.18489 0.73774<br>O 2.92095 -2.05486 0.07914<br>O 3.84361 0.46920 -0.54763<br>C -0.40825 2.47875 0.62153<br>H -0.76984 2.45670 1.65681<br>H 0.12021 3.41891 0.45577<br>H -1.29205 2.45223 -0.02513<br>S -1.32619 -0.33186 -0.92703<br>C -2.95309 -0.35814 -0.15063<br>N -3.02759 -0.98236 1.05934<br>H -2.47248 -1.81374 1.20357<br>H -3.96200 -1.03577 1.44482<br>N -3.96322 0.18531 -0.71161<br>H -3.69463 0.73759 -1.52235	C_1	-967.59962 (7.0)	-967.42104 (7.4)	-967.47672 (8.1)	-968.31093 (6.5)	-968.18803 (6.7)
6-adduct, conf15	C 2.12786 -1.12935 0.19475<br>C 2.74732 0.22668 -0.13962<br>C 1.87601 1.39226 -0.09517<br>C 0.54572 1.33271 0.13974<br>C -0.13995 0.02028 0.38056<br>C 0.73327 -1.16429 0.45886<br>H 2.36040 2.35242 -0.25486<br>H -0.78316 0.08310 1.28441<br>H 0.28503 -2.12021 0.71611<br>O 2.84192 -2.14576 0.24465<br>O 3.93966 0.30066 -0.40942<br>C -0.29578 2.56337 0.21140<br>H -0.77470 2.63537 1.19490<br>H 0.30047 3.46160 0.04258<br>H -1.10059 2.52896 -0.53057	C_1	-967.60339 (4.7)	-967.42569 (4.5)	-967.48266 (4.4)	-968.31225 (5.6)	-968.19152 (4.5)

	S -1.41792 -0.31673 -0.90344<br>C -2.89030 -0.33953 0.13155<br>N -2.78477 -0.14318 1.38845<br>H -3.68425 -0.17278 1.86530<br>N -4.01164 -0.53314 -0.61017<br>H -3.94446 -0.87842 -1.55600<br>H -4.87471 -0.74020 -0.12914						
6-adduct, conf16	C -2.19823 -1.04875 -0.14124<br>C -2.68838 0.36621 0.16946<br>C -1.74392 1.45905 -0.01678<br>C -0.44422 1.28176 -0.34311<br>C 0.13851 -0.09461 -0.48939<br>C -0.83106 -1.20712 -0.49509<br>H -2.14636 2.46128 0.10740<br>H 0.77438 -0.13775 -1.38356<br>H -0.48066 -2.20570 -0.74118<br>O -2.99090 -2.00356 -0.10223<br>O -3.84459 0.54152 0.53015<br>C 0.47910 2.42966 -0.57911<br>H 0.84310 2.41043 -1.61372<br>H -0.01827 3.38449 -0.40150<br>H 1.35957 2.36398 0.06942<br>S 1.31605 -0.42211 0.92407<br>C 2.93818 -0.33870 0.16256<br>N 3.05001 -0.87717 -1.08776<br>H 2.51123 -1.70381 -1.30544<br>H 3.97434 -0.87212 -1.49921<br>N 3.82900 0.23996 0.87260<br>H 4.75679 0.12482 0.46146	C_1	-967.59672 (8.8)	-967.41847 (9.1)	-967.47428 (9.7)	-968.30920 (7.6)	-968.18676 (7.5)
5-adduct, conf1	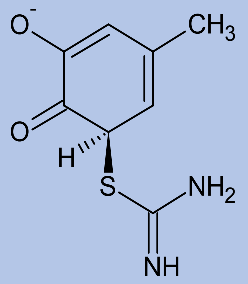<br>(5*R*)-5-(carbamimidoylsulfanyl)-3-methyl-6-oxocyclohexa-1,3-dien-1-olate<br><br>C 0.49740 1.34663 -0.55255<br>C 1.68127 1.05339 0.38047	C_1	-967.60318 (4.8)	-967.42494 (5.0)	-967.48131 (5.2)	-968.31633 (3.1)	-968.19446 (2.7)

	C 2.11974 -0.30334 0.49371<br>C 1.53149 -1.37481 -0.20393<br>C 0.45821 -1.15424 -1.04396<br>C -0.16445 0.17589 -1.26366<br>H 2.96056 -0.48622 1.15838<br>H 0.02387 -1.98492 -1.59305<br>H -0.13843 0.41963 -2.33621<br>O 0.12561 2.48789 -0.72274<br>O 2.20094 1.99222 0.99066<br>C 2.09805 -2.75536 -0.03194<br>H 3.15309 -2.77727 -0.32305<br>H 2.04402 -3.06335 1.01731<br>H 1.55787 -3.48825 -0.63442<br>S -1.98598 0.08821 -0.97022<br>C -2.03355 -0.33915 0.77353<br>N -2.69100 -1.38502 1.09655<br>H -2.75980 -1.46893 2.11306<br>N -1.32322 0.51051 1.58139<br>H -1.33817 1.49374 1.34566<br>H -1.36930 0.33096 2.57679						
5-adduct, conf2	C -0.50664 -1.34210 -0.55114<br>C -1.70743 -1.03695 0.35591<br>C -2.12446 0.32616 0.47386<br>C -1.50736 1.39321 -0.20517<br>C -0.42611 1.16141 -1.03179<br>C 0.17562 -0.17778 -1.25514<br>H -2.97304 0.51754 1.12611<br>H 0.03006 1.98901 -1.56774<br>H 0.15105 -0.41645 -2.32894<br>O -0.13924 -2.48626 -0.70929<br>O -2.25736 -1.97207 0.94441<br>C -2.05302 2.78158 -0.02924<br>H -3.10355 2.82339 -0.33411<br>H -2.00805 3.07973 1.02327<br>H -1.49269 3.51033 -0.61815<br>S 1.99596 -0.12662 -0.94565<br>C 2.02538 0.31365 0.80650<br>N 2.64500 1.33203 1.26069<br>H 2.99871 1.91213 0.50421<br>N 1.30779 -0.53135 1.60749<br>H 1.33979 -1.51728 1.38753<br>H 1.36963 -0.32576 2.59731	C_1	-967.60623 (2.9)	-967.42773 (3.2)	-967.48442 (3.3)	-968.31819 (1.9)	-968.19637 (1.5)

5-adduct, conf3	C 0.27710 1.24386 -0.62623<br>C 1.50300 1.28659 0.29622<br>C 2.23085 0.06928 0.50875<br>C 1.84825 -1.16985 -0.03054<br>C 0.72300 -1.26730 -0.82441<br>C -0.11786 -0.10579 -1.22313<br>H 3.11342 0.13921 1.14011<br>H 0.43839 -2.23114 -1.23771<br>H -0.04653 0.02128 -2.31455<br>O -0.26898 2.27150 -0.96802<br>O 1.83174 2.36362 0.79946<br>C 2.67932 -2.38628 0.26864<br>H 3.71099 -2.24061 -0.06737<br>H 2.71245 -2.57415 1.34688<br>H 2.27960 -3.27546 -0.22329<br>S -1.90104 -0.44393 -1.00646<br>C -1.98223 -0.25914 0.76654<br>N -3.18085 -0.64024 1.28421<br>H -3.75966 -1.29428 0.77915<br>H -3.27971 -0.65817 2.28888<br>N -0.97898 0.26043 1.36577<br>H -1.10426 0.32576 2.37483	C_1	-967.61080 (0.0)	-967.43290 (0.0)	-967.48794 (1.1)	-968.32124 (0.0)	-968.19838 (0.2)
5-adduct, conf4	C 0.65785 1.37009 -0.54936<br>C 1.68903 0.89274 0.48200<br>C 1.96484 -0.50946 0.53722<br>C 1.33448 -1.46378 -0.28519<br>C 0.35248 -1.07758 -1.17605<br>C -0.13868 0.31636 -1.30341<br>H 2.72217 -0.82587 1.25031<br>H -0.11918 -1.81700 -1.81729<br>H -0.15262 0.62324 -2.35775<br>O 0.49909 2.55411 -0.75263<br>O 2.24254 1.72998 1.20080<br>C 1.75556 -2.90175 -0.18430<br>H 2.82024 -3.00737 -0.41607<br>H 1.60661 -3.27292 0.83486<br>H 1.18778 -3.53476 -0.86891<br>S -1.94031 0.39591 -0.88785<br>C -1.87920 -0.13987 0.85273<br>N -2.49870 -1.32500 1.04468<br>H -2.67026 -1.94592 0.27146<br>H -2.52761 -1.70486 1.98015<br>N -1.37081 0.53823 1.81137	C_1	-967.60477 (3.8)	-967.42650 (4.0)	-967.48356 (3.8)	-968.31722 (2.5)	-968.19601 (1.7)

	H -1.08694 1.46517 1.50937						
5-adduct, conf5	C 0.27737 1.24429 -0.62840<br>C 1.50208 1.28723 0.29574<br>C 2.22959 0.07003 0.51006<br>C 1.84759 -1.16934 -0.02931<br>C 0.72374 -1.26677 -0.82504<br>C -0.11739 -0.10565 -1.22428<br>H 3.11112 0.14013 1.14281<br>H 0.43994 -2.23061 -1.23889<br>H -0.04777 0.02078 -2.31584<br>O -0.26733 2.27191 -0.97244<br>O 1.83032 2.36456 0.79859<br>C 2.67763 -2.38596 0.27194<br>H 3.70926 -2.24219 -0.06502<br>H 2.71114 -2.57154 1.35055<br>H 2.27660 -3.27577 -0.21780<br>S -1.89955 -0.44873 -1.00529<br>C -1.98332 -0.25615 0.76652<br>N -0.97925 0.26548 1.36373<br>H -1.10572 0.33716 2.37221<br>N -3.14222 -0.71937 1.30398<br>H -3.91199 -0.98719 0.70987<br>H -3.38709 -0.43174 2.24010	C_1	-967.61066 (0.1)	-967.43287 (0.0)	-967.48834 (0.8)	-968.32107 (0.1)	-968.19876 (0.0)
5-adduct, conf6	C 0.10552 1.16908 -0.68325<br>C 1.36162 1.47033 0.14729<br>C 2.24715 0.38501 0.44475<br>C 2.03058 -0.93945 0.01894<br>C 0.91522 -1.25482 -0.73217<br>C -0.09548 -0.25794 -1.17982<br>H 3.12498 0.62616 1.03914<br>H 0.76357 -2.27687 -1.06825<br>H -0.04423 -0.19328 -2.27848<br>O -0.66689 2.05954 -0.95871<br>O 1.55414 2.62758 0.52880<br>C 3.03294 -1.99678 0.38469<br>H 4.01595 -1.74954 -0.02885<br>H 3.14426 -2.06266 1.47167<br>H 2.73685 -2.97728 0.00687<br>S -1.82782 -0.83248 -0.94034<br>C -2.15557 -0.40279 0.76121<br>N -1.06643 -0.32965 1.58904<br>H -0.33166 -1.01348 1.46130<br>H -1.26492 -0.13162 2.56187	C_1	-967.60406 (4.2)	-967.42602 (4.3)	-967.48137 (5.2)	-968.31715 (2.6)	-968.19446 (2.7)

	N -3.38069 -0.15580 1.02777<br>H -3.51971 -0.03638 2.03193						
5-adduct, conf7	C 0.07717 1.14661 -0.70562<br>C 1.32493 1.48666 0.12210<br>C 2.23555 0.42587 0.43172<br>C 2.05033 -0.90767 0.02058<br>C 0.94192 -1.25850 -0.72494<br>C -0.09521 -0.29244 -1.17769<br>H 3.10770 0.69455 1.02262<br>H 0.81496 -2.28751 -1.04981<br>H -0.06547 -0.24680 -2.27782<br>O -0.71258 2.01617 -0.99841<br>O 1.49008 2.65254 0.48961<br>C 3.07830 -1.93640 0.39664<br>H 4.05765 -1.66385 -0.00948<br>H 3.18240 -1.99635 1.48476<br>H 2.81176 -2.92564 0.01968<br>S -1.81085 -0.90392 -0.89244<br>C -2.13496 -0.39918 0.79764<br>N -1.03978 -0.30679 1.61156<br>H -0.32822 -1.01998 1.52367<br>H -1.26172 -0.06739 2.57031<br>N -3.31139 -0.10582 1.19904<br>H -3.97543 -0.07541 0.42879	C_1	-967.60678 (2.5)	-967.42852 (2.7)	-967.48376 (3.7)	-968.31884 (1.5)	-968.19583 (1.8)
5-adduct, conf8	C -0.18255 0.99594 0.67047<br>C -1.38347 1.49541 -0.14190<br>C -2.43864 0.55091 -0.36738<br>C -2.37139 -0.79841 0.03418<br>C -1.20456 -1.30537 0.57593<br>C 0.03396 -0.49754 0.70217<br>H -3.33927 0.93130 -0.84312<br>H -1.14702 -2.34841 0.87307<br>H 0.62052 -0.77262 1.58198<br>O 0.55483 1.77418 1.23751<br>O -1.42729 2.67465 -0.50000<br>C -3.57762 -1.67378 -0.14567<br>H -4.42899 -1.27552 0.41542<br>H -3.87051 -1.70731 -1.20007<br>H -3.38651 -2.69411 0.19211<br>S 1.05590 -0.86225 -0.81206<br>C 2.70112 -0.33858 -0.30644<br>N 3.31040 0.43384 -1.12252<br>H 4.28141 0.57210 -0.83494	C_1	-967.60406 (4.2)	-967.42593 (4.4)	-967.48292 (4.2)	-968.31503 (3.9)	-968.19389 (3.1)

	N 3.12405 -0.82407 0.89474<br>H 2.77785 -1.71999 1.20589<br>H 4.07560 -0.61992 1.16889						
5-adduct, conf9	C -0.17210 0.98279 0.68419<br>C -1.36900 1.49662 -0.12484<br>C -2.43011 0.56081 -0.35825<br>C -2.37092 -0.79220 0.03122<br>C -1.20704 -1.31133 0.56866<br>C 0.03559 -0.51211 0.70231<br>H -3.32834 0.95076 -0.83075<br>H -1.15602 -2.35720 0.85688<br>H 0.62584 -0.80061 1.57552<br>O 0.56980 1.75075 1.25898<br>O -1.40403 2.67888 -0.47396<br>C -3.58183 -1.65916 -0.15716<br>H -4.43396 -1.25617 0.39932<br>H -3.86844 -1.68911 -1.21343<br>H -3.39947 -2.68128 0.17998<br>S 1.05687 -0.87305 -0.81518<br>C 2.70774 -0.33125 -0.30329<br>N 3.39833 0.48864 -0.99691<br>H 2.85089 0.88301 -1.75714<br>N 3.15074 -0.87052 0.86454<br>H 2.84939 -1.80132 1.11184<br>H 4.10651 -0.64828 1.11001	C_1	-967.60704 (2.4)	-967.42859 (2.7)	-967.48539 (2.7)	-968.31670 (2.9)	-968.19504 (2.3)
5-adduct, conf10	C -0.51914 1.19319 -0.10372<br>C -2.03932 1.29244 0.07983<br>C -2.78422 0.07252 0.10237<br>C -2.19364 -1.20176 0.05703<br>C -0.81719 -1.32717 0.02258<br>C 0.11001 -0.17225 0.08163<br>H -3.86670 0.16747 0.14563<br>H -0.36611 -2.31508 0.00305<br>H 0.57352 -0.13186 1.10350<br>O 0.12819 2.18881 -0.34531<br>O -2.55791 2.41232 0.14909<br>C -3.07390 -2.41920 0.06765<br>H -3.67177 -2.45307 0.98425<br>H -3.77202 -2.39656 -0.77525<br>H -2.48740 -3.33801 0.00583<br>S 1.59182 -0.42496 -0.93475<br>C 2.84103 -0.21917 0.33959<br>N 4.09702 -0.29947 -0.17018	C_1	-967.61005 (0.5)	-967.43273 (0.1)	-967.48917 (0.3)	-968.31592 (3.3)	-968.19505 (2.3)

	H 4.25188 -0.15425 -1.15676<br>H 4.87452 -0.07195 0.43203<br>N 2.47834 -0.08149 1.55700<br>H 3.26343 0.03862 2.19398						
5-adduct, conf11	C -0.20795 1.01835 0.67802<br>C -1.34773 1.48625 -0.23556<br>C -2.40662 0.54574 -0.46233<br>C -2.38246 -0.77907 0.01885<br>C -1.25022 -1.27348 0.63883<br>C -0.00294 -0.47534 0.77822<br>H -3.27543 0.91127 -1.00416<br>H -1.22228 -2.29913 0.99456<br>H 0.52914 -0.72471 1.70015<br>O 0.48492 1.81588 1.27188<br>O -1.34801 2.64167 -0.66519<br>C -3.59553 -1.64394 -0.16491<br>H -4.46189 -1.20149 0.33704<br>H -3.84473 -1.73067 -1.22743<br>H -3.43741 -2.64692 0.23587<br>S 1.06191 -0.93145 -0.66449<br>C 2.71382 -0.41589 -0.11189<br>N 3.44815 -0.02140 -1.18627<br>H 2.98537 0.42388 -1.96396<br>H 4.39436 0.27849 -0.99525<br>N 3.20139 -0.49334 1.06317<br>H 2.57798 -0.94054 1.72784	C_1	-967.60701 (2.4)	-967.42879 (2.6)	-967.48531 (2.7)	-968.31716 (2.6)	-968.19546 (2.1)
5-adduct, conf12	C -0.52741 1.19594 -0.10248<br>C -2.04829 1.28357 0.08072<br>C -2.78402 0.05812 0.10329<br>C -2.18413 -1.21169 0.05633<br>C -0.80682 -1.32692 0.01859<br>C 0.11173 -0.16537 0.07827<br>H -3.86713 0.14501 0.14787<br>H -0.34854 -2.31148 -0.00341<br>H 0.57764 -0.12392 1.09960<br>O 0.11268 2.19720 -0.34019<br>O -2.57533 2.39949 0.15024<br>C -3.05544 -2.43553 0.06859<br>H -3.65164 -2.47331 0.98613<br>H -3.75499 -2.41842 -0.77324<br>H -2.46230 -3.35004 0.00640<br>S 1.59504 -0.40055 -0.93992<br>C 2.84184 -0.21793 0.34071	C_1	-967.60993 (0.6)	-967.43277 (0.1)	-967.48968 (0.0)	-968.31574 (3.5)	-968.19549 (2.1)

	N 2.47423 -0.07460 1.55649<br>H 3.25630 0.02770 2.20025<br>N 4.09785 -0.22777 -0.17061<br>H 4.26149 -0.49636 -1.12895<br>H 4.88037 -0.32831 0.45833						
5-adduct, conf13	C -0.37068 1.16420 0.40806<br>C -1.67407 1.35377 -0.37452<br>C -2.57156 0.23830 -0.40476<br>C -2.27936 -1.01258 0.17584<br>C -1.03402 -1.25254 0.72428<br>C 0.06726 -0.25534 0.69337<br>H -3.53479 0.40237 -0.88141<br>H -0.80387 -2.22118 1.15823<br>H 0.65476 -0.27991 1.61767<br>O 0.28237 2.12286 0.76784<br>O -1.91495 2.44834 -0.88941<br>C -3.33687 -2.07789 0.17787<br>H -4.22708 -1.73411 0.71413<br>H -3.64467 -2.31161 -0.84651<br>H -2.98074 -2.99570 0.64927<br>S 1.21026 -0.76917 -0.66740<br>C 2.81405 -0.21944 -0.02633<br>N 3.03931 0.72848 0.79407<br>H 2.20842 1.27219 1.01977<br>N 3.80924 -0.93148 -0.62837<br>H 3.64907 -1.90173 -0.85515<br>H 4.74716 -0.70314 -0.32791	C_1	-967.60941 (0.9)	-967.43106 (1.2)	-967.48698 (1.7)	-968.31807 (2.0)	-968.19564 (2.0)
5-adduct, conf14	C -0.33346 1.14553 0.35086<br>C -1.68639 1.37932 -0.32703<br>C -2.59942 0.27802 -0.34429<br>C -2.29882 -0.99407 0.18229<br>C -1.03740 -1.26271 0.67713<br>C 0.07495 -0.28045 0.64225<br>H -3.58198 0.46939 -0.76842<br>H -0.80580 -2.24534 1.07760<br>H 0.65535 -0.31219 1.57116<br>O 0.38870 2.08612 0.62323<br>O -1.94231 2.49575 -0.78637<br>C -3.36789 -2.04782 0.19293<br>H -4.23712 -1.70790 0.76477<br>H -3.70991 -2.25396 -0.82654<br>H -3.00731 -2.98037 0.63092<br>S 1.22741 -0.83416 -0.70435	C_1	-967.60809 (1.7)	-967.42990 (1.9)	-967.48537 (2.7)	-968.31655 (2.9)	-968.19383 (3.1)

	C 2.85739 -0.36254 -0.12180<br>N 2.92733 0.62253 0.80942<br>H 2.18826 1.31856 0.86460<br>H 3.85066 0.93532 1.07684<br>N 3.79711 -1.04908 -0.65611<br>H 4.71328 -0.67782 -0.40354						
5-adduct, conf15	C -0.34294 1.15149 0.34127<br>C -1.70203 1.37230 -0.32882<br>C -2.60554 0.26295 -0.34102<br>C -2.29198 -1.00592 0.18533<br>C -1.02696 -1.26296 0.67768<br>C 0.07524 -0.27023 0.64218<br>H -3.59113 0.44531 -0.76205<br>H -0.78610 -2.24289 1.07929<br>H 0.65649 -0.29157 1.57086<br>O 0.37692 2.09715 0.60027<br>O -1.97007 2.48563 -0.78851<br>C -3.35133 -2.06939 0.19973<br>H -4.22320 -1.73574 0.77121<br>H -3.69216 -2.28154 -0.81891<br>H -2.98201 -2.99733 0.64021<br>S 1.24184 -0.81521 -0.69871<br>C 2.87736 -0.34322 -0.10926<br>N 2.93187 0.63423 0.82869<br>H 2.20527 1.34317 0.85712<br>H 3.86419 0.93490 1.07942<br>N 3.91806 -0.93079 -0.56852<br>H 3.68399 -1.72742 -1.15568	C_1	-967.61040 (0.3)	-967.43206 (0.5)	-967.48784 (1.2)	-968.31802 (2.0)	-968.19546 (2.1)
5-adduct, conf16	C 1.07496 -1.28021 0.37697<br>C 2.36337 -0.70136 -0.21552<br>C 2.46867 0.72253 -0.29586<br>C 1.44952 1.60656 0.10357<br>C 0.24002 1.11671 0.55850<br>C -0.09224 -0.32914 0.61319<br>H 3.40948 1.11662 -0.67247<br>H -0.53832 1.80665 0.87283<br>H -0.52079 -0.60944 1.58229<br>O 0.99362 -2.45738 0.64737<br>O 3.25517 -1.47751 -0.57174<br>C 1.69518 3.08575 0.02321<br>H 2.58101 3.36139 0.60390<br>H 1.87965 3.38566 -1.01369<br>H 0.84290 3.65494 0.39927	C_1	-967.60499 (3.6)	-967.42680 (3.8)	-967.48401 (3.6)	-968.31518 (3.8)	-968.19421 (2.9)

	S -1.35242 -0.73070 -0.67682<br>C -2.83590 0.03172 0.02707<br>N -3.12613 0.20327 1.25724<br>H -2.45115 -0.20485 1.89599<br>N -3.70527 0.33483 -0.97477<br>H -3.34360 0.60710 -1.87618<br>H -4.57241 0.77019 -0.69200						
5-adduct, conf17	C 1.13918 -1.25982 0.40549<br>C 2.34641 -0.58149 -0.24861<br>C 2.34431 0.84794 -0.30094<br>C 1.28187 1.64373 0.16447<br>C 0.12913 1.05315 0.64907<br>C -0.09606 -0.41219 0.66398<br>H 3.23706 1.31875 -0.70530<br>H -0.68416 1.67250 1.01723<br>H -0.53181 -0.74602 1.60982<br>O 1.17192 -2.43538 0.69568<br>O 3.27205 -1.28433 -0.66539<br>C 1.41735 3.13854 0.12244<br>H 2.30524 3.46129 0.67507<br>H 1.53506 3.48125 -0.91091<br>H 0.54313 3.63239 0.55094<br>S -1.28658 -0.87872 -0.69377<br>C -2.78384 0.00529 -0.21913<br>N -3.07392 0.00628 1.11311<br>H -2.84735 -0.81610 1.65355<br>H -3.96549 0.42440 1.34688<br>N -3.51958 0.58855 -1.08478<br>H -3.07369 0.61405 -1.99820	C_1	-967.60451 (4.0)	-967.42605 (4.3)	-967.48346 (3.9)	-968.31472 (4.1)	-968.19367 (3.2)
5-adduct, conf18	C 1.14317 -1.25364 0.40959<br>C 2.35724 -0.58312 -0.23935<br>C 2.35557 0.84516 -0.31190<br>C 1.28975 1.64743 0.13379<br>C 0.13537 1.06450 0.62356<br>C -0.09030 -0.40092 0.66387<br>H 3.25099 1.31008 -0.71722<br>H -0.67920 1.68997 0.97799<br>H -0.50848 -0.72048 1.62290<br>O 1.16834 -2.42928 0.70037<br>O 3.28715 -1.29152 -0.63735<br>C 1.42363 3.14156 0.06609<br>H 2.30737 3.47547 0.61876<br>H 1.54761 3.46607 -0.97238	C_1	-967.60164 (5.8)	-967.42339 (6.0)	-967.48027 (5.9)	-968.31318 (5.1)	-968.19182 (4.4)

	H 0.54596 3.64170 0.47998<br>S -1.28871 -0.89045 -0.67606<br>C -2.78067 -0.01030 -0.22138<br>N -3.03047 0.11129 1.11555<br>H -2.73515 -0.63210 1.73206<br>H -3.91649 0.52614 1.37278<br>N -3.46506 0.42749 -1.20854<br>H -4.37101 0.78129 -0.89676						

Part 11: 4-Methylcatechol – methanethiol adducts, optimizations using the ωB97X-D functional

Table S11.1. Reduced neutral form in vacuo.

Structure	Schematic drawing	Symmetry	*E*	H_{RRHO}	G_{RRHO}	$E_{\omega B97X\text{-}D,large}$	$G_{RRHO,\omega B97X\text{-}D,large}$
6-adduct, conf1	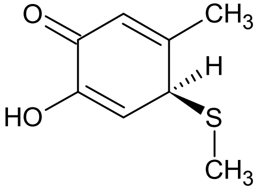<br>(4*S*)-2-hydroxy-5-methyl-4-(methylsulfanyl)cyclohexa-2,5-dien-1-one<br><br>C -1.55288 -0.64516 -0.42360<br>C -1.71546 0.64559 0.29260<br>C -0.58290 1.56870 0.29006<br>C 0.59123 1.26124 -0.29164<br>C 0.81163 -0.09495 -0.91152<br>C -0.39414 -0.96952 -1.01232<br>H -0.75064 2.53405 0.75825<br>H 1.24996 0.04600 -1.90784<br>H -0.28427 -1.91788 -1.52811<br>O -2.64737 -1.42729 -0.43758<br>O -2.79446 0.88668 0.82815<br>C 1.72814 2.23807 -0.34390<br>H 2.00414 2.44229 -1.38465<br>H 1.46075 3.18214 0.13452<br>H 2.61524 1.82625 0.14698<br>S 2.17265 -1.00282 -0.03206<br>C 1.43207 -1.18572 1.61110<br>H 2.13058 -1.78282 2.20039<br>H 1.29278 -0.21713 2.09675<br>H 0.47730 -1.71288 1.55231<br>H -3.32990 -0.93962 0.05517	C_1	-859.35840 (0.0)	-859.17840 (0.0)	-859.22765 (0.0)	-859.49982 (0.0)	-859.36907 (0.0)
6-adduct, conf2	C 1.45763 -0.98955 0.17792<br>C 2.04950 0.32655 -0.16564<br>C 1.18107 1.49837 -0.08761<br>C -0.12104 1.40813 0.23801<br>C -0.75690 0.07026 0.52470<br>C 0.16725 -1.10252 0.51678	C_1	-859.35308 (3.3)	-859.17322 (3.2)	-859.22321 (2.8)	-859.49434 (3.4)	-859.36447 (2.9)

	H 1.65090 2.45690 -0.28577<br>H -1.24496 0.14512 1.50672<br>H -0.23773 -2.07526 0.77571<br>O 2.31405 -2.02485 0.12883<br>O 3.23718 0.37338 -0.47534<br>C -0.99081 2.62739 0.34921<br>H -1.52496 2.63450 1.30537<br>H -0.39592 3.53980 0.27456<br>H -1.75145 2.63975 -0.43737<br>S -2.14630 -0.16815 -0.68341<br>C -3.00627 -1.55405 0.10568<br>H -3.94942 -1.68057 -0.42951<br>H -2.43887 -2.48404 0.03244<br>H -3.22885 -1.33251 1.15294<br>H 3.16967 -1.64904 -0.14273						
6-adduct, conf3	C 1.71275 -0.84932 0.19438<br>C 2.05031 0.55633 -0.14414<br>C 0.96983 1.53798 -0.10604<br>C -0.29840 1.19951 0.18842<br>C -0.67381 -0.23169 0.48385<br>C 0.45918 -1.20758 0.49988<br>H 1.24778 2.56553 -0.32008<br>H -1.17269 -0.25430 1.46267<br>H 0.22834 -2.23974 0.74137<br>O 2.75260 -1.70106 0.17912<br>O 3.21555 0.83009 -0.41878<br>C -1.38918 2.22877 0.24173<br>H -1.92294 2.18244 1.19747<br>H -0.98461 3.23532 0.12056<br>H -2.11861 2.05175 -0.55590<br>S -1.95090 -0.86295 -0.70744<br>C -3.48186 -0.56949 0.22197<br>H -4.29071 -0.99326 -0.37701<br>H -3.45915 -1.08312 1.18634<br>H -3.67984 0.49374 0.37165<br>H 3.52868 -1.17032 -0.07096	C_1	-859.35287 (3.5)	-859.17325 (3.2)	-859.22248 (3.2)	-859.49422 (3.5)	-859.36383 (3.3)

5-adduct, conf1	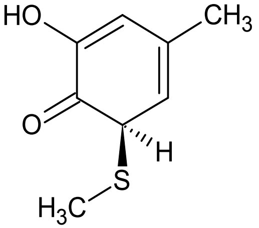<br>(6*R*)-2-hydroxy-4-methyl-6-(methylsulfanyl)cyclohexa-2,4-dien-1-one<br><br>C -0.51688 1.06957 -0.56699<br>C 0.68711 1.45800 0.19635<br>C 1.70899 0.59483 0.36350<br>C 1.68141 -0.75668 -0.19795<br>C 0.59220 -1.19988 -0.84987<br>C -0.65306 -0.38320 -0.97720<br>H 2.59139 0.92064 0.90741<br>H 0.55750 -2.20345 -1.26326<br>H -1.03523 -0.39580 -2.00374<br>O -1.36353 1.91812 -0.80795<br>O 0.69325 2.71899 0.66444<br>C 2.91886 -1.59458 -0.02355<br>H 3.78832 -1.10322 -0.47360<br>H 3.14224 -1.73966 1.03933<br>H 2.80208 -2.57714 -0.48613<br>S -2.03881 -1.16615 -0.03512<br>C -1.39762 -1.06706 1.65790<br>H -2.06951 -1.66653 2.27529<br>H -0.39184 -1.48921 1.72091<br>H -1.39816 -0.04029 2.03041<br>H -0.12949 3.13022 0.35010	C_1	-859.35109 (4.6)	-859.17150 (4.3)	-859.22087 (4.3)	-859.49190 (5.0)	-859.36169 (4.6)
5-adduct, conf2	C 0.38480 0.92655 0.47120<br>C -0.84403 1.42301 -0.18161<br>C -1.91188 0.61631 -0.33671<br>C -1.89140 -0.78099 0.09866<br>C -0.77213 -1.32189 0.61071<br>C 0.50988 -0.55813 0.70489<br>H -2.82066 1.01863 -0.77513<br>H -0.74506 -2.36264 0.91908<br>H 1.01884 -0.72453 1.65982<br>O 1.26559 1.72740 0.76646<br>O -0.83354 2.72283 -0.52388	C_1	-859.35165 (4.2)	-859.17236 (3.8)	-859.22290 (3.0)	-859.49244 (4.6)	-859.36368 (3.4)

	C -3.16996 -1.55932 -0.04670<br>H -3.98923 -1.06606 0.48763<br>H -3.46155 -1.62517 -1.10074<br>H -3.06517 -2.57391 0.34396<br>S 1.61518 -1.22447 -0.62759<br>C 3.21275 -0.51240 -0.15114<br>H 3.93211 -0.86448 -0.89390<br>H 3.18244 0.57803 -0.16185<br>H 3.51770 -0.86438 0.83716<br>H 0.02326 3.07543 -0.23096						
5-adduct, conf3	C 0.42672 -1.20386 0.41736<br>C 1.68675 -0.70247 -0.16488<br>C 1.90624 0.61988 -0.30848<br>C 0.91768 1.61397 0.10257<br>C -0.27755 1.22820 0.58283<br>C -0.68560 -0.20611 0.67282<br>H 2.85445 0.95708 -0.71760<br>H -1.00382 1.97050 0.89978<br>H -1.12125 -0.44138 1.65032<br>O 0.30990 -2.40083 0.63986<br>O 2.59043 -1.64508 -0.48270<br>C 1.30720 3.06043 -0.03336<br>H 2.22169 3.27344 0.53077<br>H 1.50818 3.30522 -1.08231<br>H 0.51793 3.72395 0.32736<br>S -1.98252 -0.62511 -0.59089<br>C -3.27579 0.55036 -0.11427<br>H -4.16113 0.27647 -0.69218<br>H -3.51527 0.45823 0.94849<br>H -3.00846 1.58138 -0.35288<br>H 2.19144 -2.49822 -0.24226	C_1	-859.34642 (7.5)	-859.16669 (7.3)	-859.21729 (6.5)	-859.48730 (7.9)	-859.35817 (6.8)

Table S11.2. Reduced neutral form in water.

Structure	Schematic drawing	Symmetry	G_{PCM}	$H_{PCM,RRHO}$	$G_{PCM,RRHO}$	$G_{SMD,\omega B97X\text{-}D,large}$	$G_{SMD,RRHO,\omega B97X\text{-}D,large}$
6-adduct, conf1	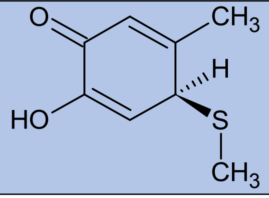	C_1	-859.36842 (0.0)	-859.18899 (0.0)	-859.23930 (0.0)	-859.51093 (0.0)	-859.38182 (0.0)

	(4*S*)-2-hydroxy-5-methyl-4-(methylsulfanyl)cyclohexa-2,5-dien-1-one<br><br>C -1.56551 -0.66694 -0.38646<br>C -1.72849 0.64841 0.27452<br>C -0.60127 1.56934 0.23947<br>C 0.57725 1.24099 -0.32636<br>C 0.79127 -0.12724 -0.91726<br>C -0.40925 -1.01230 -0.96669<br>H -0.76127 2.54902 0.67950<br>H 1.19828 -0.00742 -1.92893<br>H -0.30032 -1.97479 -1.45606<br>O -2.66085 -1.45826 -0.37038<br>O -2.81395 0.90997 0.80161<br>C 1.72251 2.20632 -0.37142<br>H 2.13543 2.27142 -1.38298<br>H 1.40906 3.20150 -0.05171<br>H 2.53228 1.86666 0.28259<br>S 2.17498 -1.00713 -0.04970<br>C 1.52805 -1.05793 1.64210<br>H 2.23739 -1.64631 2.22668<br>H 1.45651 -0.05589 2.07059<br>H 0.55198 -1.54688 1.67289<br>H -3.34795 -0.96071 0.10534						
6-adduct, conf2	C 1.45796 -0.98854 0.17465<br>C 2.05109 0.32538 -0.15792<br>C 1.18847 1.49564 -0.08292<br>C -0.11823 1.40726 0.23482<br>C -0.75494 0.07096 0.51733<br>C 0.16594 -1.10353 0.50598<br>H 1.65676 2.45644 -0.27345<br>H -1.24486 0.14085 1.49748<br>H -0.24562 -2.07458 0.76152<br>O 2.31312 -2.03352 0.12423<br>O 3.24671 0.37095 -0.46294<br>C -0.98279 2.62815 0.34462<br>H -1.53007 2.62582 1.29262<br>H -0.38368 3.53834 0.28477<br>H -1.73003 2.64935 -0.45466<br>S -2.13973 -0.18041 -0.69366<br>C -3.04133 -1.52198 0.12911<br>H -3.97504 -1.64962 -0.42147<br>H -2.48780 -2.46195 0.10248	C_1	-859.36437 (2.5)	-859.18486 (2.6)	-859.23486 (2.8)	-859.50695 (2.5)	-859.37745 (2.7)

	H -3.27477 -1.25220 1.16138<br>H 3.17506 -1.66550 -0.13656						
6-adduct, conf3	C 1.70711 -0.84207 0.20403<br>C 2.03733 0.55585 -0.15909<br>C 0.96401 1.53743 -0.11598<br>C -0.30328 1.20336 0.19958<br>C -0.67544 -0.22537 0.50139<br>C 0.45830 -1.19793 0.52929<br>H 1.23604 2.56515 -0.33681<br>H -1.18548 -0.24997 1.47284<br>H 0.23007 -2.22351 0.80084<br>O 2.74974 -1.70085 0.19461<br>O 3.20490 0.82454 -0.45651<br>C -1.38799 2.23538 0.27152<br>H -1.90640 2.18431 1.23441<br>H -0.98171 3.24011 0.14503<br>H -2.13235 2.06385 -0.51331<br>S -1.92981 -0.86456 -0.71012<br>C -3.48548 -0.58627 0.18372<br>H -4.27566 -0.99885 -0.44650<br>H -3.48151 -1.11428 1.13918<br>H -3.67917 0.47539 0.34223<br>H 3.52870 -1.18091 -0.06765	C_1	-859.36453 (2.4)	-859.18521 (2.4)	-859.23473 (2.9)	-859.50719 (2.3)	-859.37740 (2.8)
5-adduct, conf1	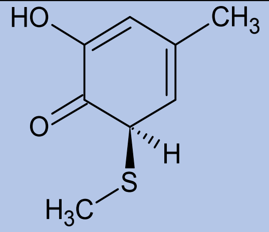<br>(6*R*)-2-hydroxy-4-methyl-6-(methylsulfanyl)cyclohexa-2,4-dien-1-one<br><br>C -0.50088 1.07603 -0.55725<br>C 0.71265 1.45221 0.18354<br>C 1.72219 0.57377 0.35213<br>C 1.67659 -0.78245 -0.19481<br>C 0.57800 -1.21142 -0.84046<br>C -0.65034 -0.37216 -0.97592<br>H 2.61186 0.88785 0.89131<br>H 0.52610 -2.21460 -1.25250<br>H -1.01725 -0.37839 -2.00820	C_1	-859.36108 (4.6)	-859.18174 (4.6)	-859.23120 (5.1)	-859.50302 (5.0)	-859.37313 (5.4)

	O -1.35667 1.92795 -0.77841<br>O 0.75219 2.72009 0.64772<br>C 2.90014 -1.63754 -0.01083<br>H 3.77876 -1.15932 -0.45638<br>H 3.11307 -1.77941 1.05416<br>H 2.76963 -2.61947 -0.47054<br>S -2.05695 -1.13505 -0.04754<br>C -1.43504 -1.05496 1.65511<br>H -2.12200 -1.64807 2.26118<br>H -0.43638 -1.49205 1.72282<br>H -1.42448 -0.02986 2.03060<br>H -0.07212 3.14897 0.36469						
5-adduct, conf2	C 0.38844 0.92486 0.48651<br>C -0.82697 1.42136 -0.18463<br>C -1.89740 0.61932 -0.34821<br>C -1.89373 -0.77611 0.09609<br>C -0.77954 -1.31949 0.61765<br>C 0.50497 -0.56068 0.71557<br>H -2.80076 1.02206 -0.79783<br>H -0.76088 -2.35566 0.94140<br>H 1.01724 -0.73998 1.66555<br>O 1.26956 1.72205 0.79936<br>O -0.81476 2.72372 -0.53888<br>C -3.17937 -1.54246 -0.04751<br>H -3.99297 -1.03794 0.48435<br>H -3.47195 -1.60645 -1.10109<br>H -3.08280 -2.55611 0.34705<br>S 1.60063 -1.22326 -0.62910<br>C 3.21241 -0.53087 -0.17161<br>H 3.92603 -0.92755 -0.89683<br>H 3.20773 0.55826 -0.22743<br>H 3.49958 -0.85396 0.83101<br>H 0.03743 3.08769 -0.24765	C_1	-859.35997 (5.3)	-859.18094 (5.0)	-859.23150 (4.9)	-859.50144 (6.0)	-859.37297 (5.6)
5-adduct, conf3	C 0.39239 -1.20898 0.39319<br>C 1.68314 -0.74710 -0.13481<br>C 1.94576 0.56860 -0.27590<br>C 0.97682 1.59846 0.09083<br>C -0.24284 1.24859 0.53719<br>C -0.68575 -0.17413 0.63937<br>H 2.91673 0.87647 -0.65443<br>H -0.96098 2.00920 0.82694<br>H -1.12360 -0.38109 1.62241<br>O 0.21705 -2.40912 0.58690	C_1	-859.35762 (6.8)	-859.17833 (6.7)	-859.22841 (6.8)	-859.49935 (7.3)	-859.37014 (7.3)

	O 2.57952 -1.71357 -0.42711<br>C 1.41288 3.03110 -0.04914<br>H 2.31694 3.22188 0.53856<br>H 1.65220 3.25740 -1.09392<br>H 0.63214 3.71899 0.28213<br>S -2.01208 -0.55521 -0.60499<br>C -3.31317 0.57660 -0.04492<br>H -4.20695 0.31504 -0.61463<br>H -3.51718 0.43150 1.01802<br>H -3.06126 1.61900 -0.24353<br>H 2.15725 -2.56237 -0.21484						

Table S11.3. Reduced zwitterionic form in water.

Structure	Schematic drawing	Symmetry	G_{PCM}	$H_{PCM,RRHO}$	$G_{PCM,RRHO}$	$G_{SMD,\omega B97X\text{-}D,large}$	$G_{SMD,RRHO,\omega B97X\text{-}D,large}$
6-adduct, several starting structures	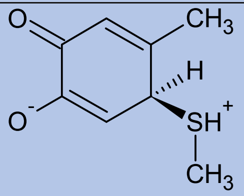<br><br>(3*S*)-4-methyl-3-(methylsulfonio)-6-oxocyclohexa-1,4-dien-1-olate	C_1	dissociates to neutral molecules				
5-adduct, several starting structures	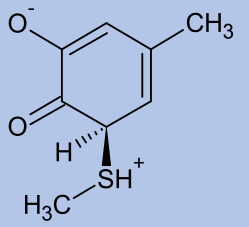<br><br>(5*R*)-3-methyl-5-(methylsulfonio)-6-oxocyclohexa-1,3-dien-1-olate	C_1	most starting structures dissociate to neutral molecules				
5-adduct, conf1	C -0.18907 1.18587 -0.35897<br>C 1.24452 1.42347 0.08443<br>C 1.99063 0.26743 0.27724<br>C 1.56377 -1.06258 -0.09121<br>C 0.35208 -1.32114 -0.64079<br>C -0.57898 -0.19960 -0.90480<br>H 2.98219 0.37928 0.71015<br>H 0.09028 -2.30785 -1.00903	C_1	-859.29408 (1.0)	-859.11804 (1.2)	-859.16906 (1.3)	-859.44512 (0.7)	-859.32010 (1.0)

	H -0.77055 -0.06752 -1.97843<br>O -1.05289 2.04262 -0.25734<br>O 1.58991 2.61458 0.33491<br>C 2.55860 -2.17441 0.11742<br>H 3.48557 -1.96819 -0.42754<br>H 2.81969 -2.25102 1.17845<br>H 2.16494 -3.13819 -0.21315<br>S -2.30169 -0.49094 -0.23778<br>C -1.96028 -0.76221 1.51812<br>H -2.82368 -1.27159 1.94361<br>H -1.05389 -1.36155 1.61623<br>H -1.84191 0.22005 1.97358<br>H -2.40933 -1.77691 -0.61855						
5-adduct, conf2	C -0.20591 1.18149 -0.33250<br>C 1.22611 1.43830 0.09298<br>C 1.99487 0.29375 0.26396<br>C 1.58797 -1.04749 -0.09319<br>C 0.37478 -1.32595 -0.62273<br>C -0.57563 -0.21088 -0.87916<br>H 2.99113 0.42345 0.68122<br>H 0.11419 -2.31853 -0.97502<br>H -0.73491 -0.07105 -1.95831<br>O -1.07797 2.03333 -0.24467<br>O 1.55889 2.63349 0.34695<br>C 2.60328 -2.14145 0.11151<br>H 3.51762 -1.92785 -0.45182<br>H 2.88374 -2.20348 1.16851<br>H 2.21853 -3.11436 -0.20265<br>S -2.26645 -0.65118 -0.28166<br>C -1.99989 -0.81184 1.50081<br>H -2.96337 -1.04574 1.95141<br>H -1.30395 -1.63990 1.63555<br>H -1.59258 0.11371 1.90471<br>H -2.74796 0.61263 -0.31534	C_1	-859.29569 (0.0)	-859.11999 (0.0)	-859.17106 (0.0)	-859.44626 (0.0)	-859.32164 (0.0)
5-adduct, conf3	C 0.13480 1.04465 -0.07485<br>C -1.31512 1.44895 0.12087<br>C -2.20884 0.38558 0.08621<br>C -1.83485 -1.01214 0.03256<br>C -0.54760 -1.42299 0.09685<br>C 0.50873 -0.39098 0.31945<br>H -3.26985 0.62558 0.07143<br>H -0.27500 -2.47079 0.15580<br>H 0.74224 -0.32371 1.39550	C_1	-859.29183 (2.4)	-859.11623 (2.4)	-859.16686 (2.6)	-859.44188 (2.7)	-859.31692 (3.0)

	O 0.97814 1.80593 -0.52072<br>O -1.58236 2.68664 0.16119<br>C -2.95503 -2.01508 -0.06556<br>H -3.62903 -1.92426 0.79287<br>H -3.55236 -1.83003 -0.96471<br>H -2.57755 -3.03953 -0.10434<br>S 2.11076 -0.86245 -0.45255<br>C 3.34214 -0.21111 0.70767<br>H 4.31324 -0.29879 0.22153<br>H 3.11090 0.82735 0.93835<br>H 3.31211 -0.83846 1.59742<br>H 2.14153 0.16990 -1.32591						
5-adduct, conf4	C 0.14260 1.05902 0.05612<br>C -1.32169 1.45970 0.05230<br>C -2.20629 0.39083 -0.03154<br>C -1.82861 -1.00295 0.03978<br>C -0.54939 -1.41080 0.22257<br>C 0.50246 -0.37985 0.43026<br>H -3.26109 0.62131 -0.16345<br>H -0.30000 -2.45330 0.39088<br>H 0.82578 -0.35189 1.48166<br>O 1.04105 1.83073 -0.24358<br>O -1.59503 2.69522 0.01197<br>C -2.94127 -2.01450 -0.05481<br>H -3.67719 -1.85102 0.73951<br>H -3.46831 -1.90767 -1.00885<br>H -2.56623 -3.03750 0.02306<br>S 2.03404 -0.77399 -0.53746<br>C 3.40882 -0.19036 0.48892<br>H 4.32334 -0.58857 0.05019<br>H 3.39372 0.89605 0.42912<br>H 3.28145 -0.53738 1.51325<br>H 2.09586 -2.08025 -0.22466	C_1	-859.29264 (1.9)	-859.11671 (2.1)	-859.16734 (2.3)	-859.44352 (1.7)	-859.31822 (2.1)
5-adduct, conf5	C 0.32642 -1.27039 0.44490<br>C 1.68069 -0.99242 -0.19134<br>C 1.99853 0.35389 -0.34253<br>C 1.17276 1.44873 0.09992<br>C -0.07024 1.27093 0.62118<br>C -0.62832 -0.09081 0.71542<br>H 2.96477 0.59192 -0.78141<br>H -0.65860 2.10109 0.99589<br>H -1.18148 -0.29161 1.63678<br>O -0.06345 -2.39580 0.70381	C_1	-859.29291 (1.7)	-859.11686 (2.0)	-859.16764 (2.1)	-859.44426 (1.2)	-859.31900 (1.7)

	O 2.39065 -1.98918 -0.50756<br>C 1.75669 2.83219 -0.01542<br>H 2.69646 2.90052 0.54215<br>H 1.98693 3.05618 -1.06252<br>H 1.06989 3.59438 0.35940<br>S -1.91640 -0.29556 -0.64922<br>C -3.22853 0.81886 -0.09567<br>H -4.10174 0.61319 -0.71431<br>H -3.44508 0.65124 0.95821<br>H -2.87708 1.83405 -0.27376<br>H -2.45734 -1.46805 -0.26167						

Table S11.4. Reduced anionic form in water.

Structure	Schematic drawing	Symmetry	G_{PCM}	$H_{PCM,RRHO}$	$G_{PCM,RRHO}$	$G_{SMD,\omega B97X\text{-}D,large}$	$G_{SMD,RRHO,\omega B97X\text{-}D,large}$
6-adduct, conf1	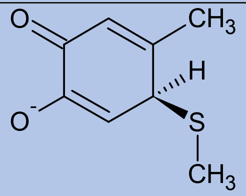<br>(3*S*)-4-methyl-3-(methylsulfanyl)-6-oxocyclohexa-1,4-dien-1-olate<br><br>C -1.65819 -0.71026 -0.44784<br>C -1.74050 0.60629 0.30809<br>C -0.60086 1.53425 0.24902<br>C 0.57059 1.23507 -0.33916<br>C 0.77266 -0.12985 -0.93414<br>C -0.44620 -0.97585 -1.04134<br>H -0.75983 2.50755 0.70879<br>H 1.25706 -0.01467 -1.91224<br>H -0.32851 -1.91756 -1.57416<br>O -2.70641 -1.44995 -0.48467<br>O -2.75033 0.91868 0.94498<br>C 1.69863 2.22109 -0.41695<br>H 1.97870 2.39567 -1.46190<br>H 1.42140 3.17627 0.03377<br>H 2.58720 1.83449 0.09222<br>S 2.13187 -1.01073 0.01841<br>C 1.36124 -1.12594 1.65273	C_1	-858.88276 (0.0)	-858.71716 (0.0)	-858.76722 (0.0)	-859.03085 (0.0)	-858.91531 (0.0)

	H 1.98522 -1.78422 2.25996<br>H 1.29726 -0.14641 2.13165<br>H 0.36123 -1.55815 1.56247						
6-adduct, conf2	C 1.49664 -1.10500 0.23562<br>C 2.05204 0.22820 -0.23385<br>C 1.21729 1.43176 -0.10513<br>C -0.06972 1.40600 0.28289<br>C -0.72622 0.08880 0.58762<br>C 0.17722 -1.09167 0.62725<br>H 1.70899 2.37471 -0.33524<br>H -1.26657 0.19107 1.53833<br>H -0.25989 -2.02515 0.97494<br>O 2.28648 -2.11555 0.24345<br>O 3.19373 0.32266 -0.69238<br>C -0.88182 2.66119 0.43119<br>H -1.35916 2.69538 1.41635<br>H -0.25641 3.54879 0.31447<br>H -1.68442 2.70344 -0.31237<br>S -2.10179 -0.09916 -0.67841<br>C -2.90488 -1.59776 -0.05043<br>H -3.82993 -1.72409 -0.61627<br>H -2.27708 -2.47894 -0.19315<br>H -3.15185 -1.48467 1.00808	C_1	-858.87911 (2.3)	-858.71346 (2.3)	-858.76377 (2.2)	-859.02699 (2.4)	-858.91165 (2.3)
6-adduct, conf3	C 1.49626 -1.11183 0.21986<br>C 2.06315 0.22669 -0.22006<br>C 1.22536 1.42900 -0.10031<br>C -0.06540 1.40368 0.27498<br>C -0.72382 0.08747 0.57917<br>C 0.17591 -1.09636 0.60856<br>H 1.71931 2.37222 -0.32441<br>H -1.25619 0.18994 1.53459<br>H -0.26607 -2.03229 0.94323<br>O 2.27822 -2.12873 0.21207<br>O 3.21597 0.32745 -0.64880<br>C -0.87779 2.65992 0.41349<br>H -1.66997 2.70428 -0.34101<br>H -1.36855 2.69438 1.39199<br>H -0.24913 3.54641 0.30594<br>S -2.11037 -0.09604 -0.67409<br>C -2.92312 -1.58148 -0.02747<br>H -3.85382 -1.70350 -0.58490<br>H -2.30566 -2.47039 -0.16735<br>H -3.16069 -1.45686 1.03193	C_1	-858.87911 (2.3)	-858.71339 (2.4)	-858.76408 (2.0)	-859.02704 (2.4)	-858.91201 (2.1)

6-adduct, conf4	C 1.81495 -0.93488 0.20092<br>C 2.09772 0.51209 -0.16536<br>C 1.00290 1.49011 -0.09446<br>C -0.26645 1.17264 0.21642<br>C -0.62894 -0.25545 0.51704<br>C 0.51012 -1.21857 0.52996<br>H 1.27807 2.52077 -0.30915<br>H -1.15670 -0.27291 1.48130<br>H 0.26214 -2.24220 0.80357<br>O 2.79504 -1.76322 0.19119<br>O 3.22608 0.88264 -0.50060<br>C -1.34151 2.21863 0.28614<br>H -1.88704 2.15660 1.23369<br>H -0.91823 3.22083 0.19228<br>H -2.06969 2.08053 -0.52061<br>S -1.90895 -0.87996 -0.69376<br>C -3.47496 -0.49174 0.14237<br>H -4.26818 -0.94466 -0.45607<br>H -3.49371 -0.93376 1.14129<br>H -3.65545 0.58190 0.21086	C_1	-858.87921 (2.2)	-858.71377 (2.1)	-858.76386 (2.1)	-859.02766 (2.0)	-858.91232 (1.9)
5-adduct, conf1	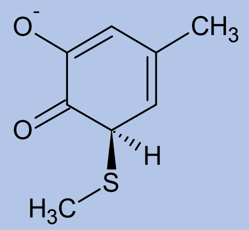<br>(5*R*)-3-methyl-5-(methylsulfanyl)-6-oxocyclohexa-1,3-dien-1-olate<br><br>C -0.59565 1.09204 -0.59690<br>C 0.51640 1.57047 0.32581<br>C 1.61145 0.72443 0.40489<br>C 1.70384 -0.56903 -0.24862<br>C 0.66397 -1.10271 -0.92641<br>C -0.63554 -0.38034 -0.99805<br>H 2.46588 1.05796 0.99203<br>H 0.72486 -2.08424 -1.38778<br>H -1.08173 -0.42972 -1.99663<br>O -1.46071 1.85588 -1.00667<br>O 0.37982 2.70415 0.89428<br>C 3.01619 -1.30080 -0.12646	C_1	-858.88119 (1.0)	-858.71575 (0.9)	-858.76516 (1.3)	-859.02672 (2.6)	-858.91068 (2.9)

	H 2.99146 -2.26398 -0.64281<br>H 3.83451 -0.70385 -0.54461<br>H 3.25828 -1.48026 0.92739<br>S -1.94760 -1.21188 0.03271<br>C -1.16713 -1.25669 1.66963<br>H -1.77597 -1.91889 2.28810<br>H -0.15710 -1.66478 1.58955<br>H -1.13265 -0.26661 2.12711						
5-adduct, conf2	C -0.60543 1.08214 -0.60268<br>C 0.49960 1.57239 0.32209<br>C 1.60282 0.73738 0.40441<br>C 1.70908 -0.55609 -0.24703<br>C 0.67526 -1.10104 -0.92517<br>C -0.63126 -0.39163 -0.99952<br>H 2.45268 1.07979 0.99303<br>H 0.74633 -2.08255 -1.38508<br>H -1.07757 -0.44927 -1.99756<br>O -1.47628 1.83744 -1.01592<br>O 0.35037 2.70533 0.88878<br>C 3.02829 -1.27486 -0.12158<br>H 3.01487 -2.23760 -0.63916<br>H 3.84175 -0.66906 -0.53648<br>H 3.26894 -1.45317 0.93278<br>S -1.93355 -1.23209 0.03663<br>C -1.15668 -1.24095 1.67608<br>H -1.74371 -1.91944 2.29782<br>H -0.13288 -1.61429 1.59933<br>H -1.15647 -0.24708 2.12672	C_1	-858.88119 (1.0)	-858.71562 (1.0)	-858.76473 (1.6)	-859.02670 (2.6)	-858.91025 (3.2)
5-adduct, conf3	C 0.41588 0.92458 0.56320<br>C -0.71929 1.52492 -0.25956<br>C -1.83337 0.70930 -0.39152<br>C -1.91030 -0.65299 0.09983<br>C -0.84608 -1.27839 0.65376<br>C 0.46955 -0.58254 0.74237<br>H -2.71051 1.12353 -0.88609<br>H -0.90061 -2.30919 0.99141<br>H 1.01275 -0.80825 1.66509<br>O 1.28879 1.62824 1.05846<br>O -0.58860 2.72061 -0.67794<br>C -3.23653 -1.35576 -0.04343<br>H -3.20506 -2.36592 0.37312<br>H -4.02964 -0.79581 0.46468<br>H -3.52122 -1.42648 -1.09942	C_1	-858.88116 (1.0)	-858.71598 (0.7)	-858.76591 (0.8)	-859.02638 (2.8)	-858.91113 (2.6)

	S 1.51961 -1.21805 -0.65920<br>C 3.15409 -0.53647 -0.26494<br>H 3.83396 -0.89334 -1.04152<br>H 3.14212 0.55479 -0.26913<br>H 3.49922 -0.89728 0.70663						
5-adduct, conf4	C 0.39349 -1.25814 0.48855<br>C 1.65407 -0.88189 -0.27550<br>C 1.89180 0.48064 -0.38249<br>C 1.01274 1.51105 0.13319<br>C -0.18890 1.21723 0.67904<br>C -0.66942 -0.18999 0.73310<br>H 2.81801 0.79449 -0.86149<br>H -0.84308 1.99624 1.05847<br>H -1.16794 -0.42377 1.67906<br>O 0.19726 -2.40320 0.87597<br>O 2.40343 -1.82468 -0.69194<br>C 1.49769 2.93514 0.03204<br>H 0.77367 3.63980 0.44965<br>H 2.44956 3.06077 0.56013<br>H 1.67655 3.20493 -1.01510<br>S -1.93180 -0.54972 -0.60250<br>C -3.15209 0.74572 -0.25676<br>H -3.98244 0.57962 -0.94608<br>H -3.52045 0.66881 0.76887<br>H -2.73694 1.73912 -0.43281	C_1	-858.87825 (2.8)	-858.71282 (2.7)	-858.76271 (2.8)	-859.02344 (4.6)	-858.90790 (4.6)

Table S11.5. Reduced cationic form in water.

Structure	Schematic drawing	Symmetry	G_{PCM}	$H_{PCM,RRHO}$	$G_{PCM,RRHO}$	$G_{SMD,\omega B97X\text{-}D,large}$	$G_{SMD,RRHO,\omega B97X\text{-}D,large}$
6-adduct, conf1	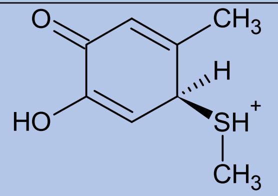<br>[(1*S*)-5-hydroxy-2-methyl-4-oxocyclohexa-2,5-dien-1-yl](methyl)sulfonium<br><br>C -1.56709 -0.67569 -0.40654<br>C -1.76890 0.62155 0.30046<br>C -0.66462 1.57866 0.29951	C_1	-859.76932 (0.0)	-859.57860 (0.0)	-859.62847 (0.0)	-859.92219 (0.4)	-859.78134 (0.2)

	C 0.52219 1.30854 -0.26952<br>C 0.75095 -0.03425 -0.92636<br>C -0.39843 -0.97402 -0.99044<br>H -0.85786 2.54124 0.76138<br>H 1.19201 0.11428 -1.91879<br>H -0.26861 -1.90683 -1.52910<br>O -2.63212 -1.48438 -0.43379<br>O -2.85677 0.83410 0.82517<br>C 1.63822 2.30871 -0.31428<br>H 1.97206 2.46148 -1.34515<br>H 1.31188 3.26426 0.09692<br>H 2.50923 1.97567 0.26063<br>S 2.19757 -0.83522 -0.01551<br>C 1.48530 -1.26615 1.58774<br>H 2.20936 -1.90830 2.08833<br>H 1.36552 -0.33536 2.14128<br>H 0.53339 -1.77758 1.45265<br>H -3.34698 -1.03175 0.04724<br>H 2.14420 -2.03542 -0.62164						
6-adduct, conf2	C -1.60263 -0.69440 -0.35585<br>C -1.77242 0.64104 0.28318<br>C -0.65032 1.57561 0.22012<br>C 0.52732 1.25191 -0.34029<br>C 0.72250 -0.11968 -0.93652<br>C -0.44661 -1.04684 -0.93339<br>H -0.82107 2.56457 0.63277<br>H 1.13369 -0.02760 -1.94871<br>H -0.33814 -2.00787 -1.42470<br>O -2.68303 -1.48328 -0.32935<br>O -2.84966 0.90331 0.80729<br>C 1.66516 2.22640 -0.42162<br>H 2.15569 2.18374 -1.39786<br>H 1.30740 3.24272 -0.25350<br>H 2.42486 2.02500 0.34299<br>S 2.09671 -1.05268 -0.06285<br>C 1.63938 -0.93162 1.68177<br>H 2.45034 -1.39020 2.24650<br>H 1.49493 0.10741 1.97395<br>H 0.72425 -1.51039 1.80503<br>H -3.38371 -0.99279 0.13487<br>H 3.07171 -0.12643 -0.11368	C_1	-859.76874 (0.4)	-859.57811 (0.3)	-859.62760 (0.5)	-859.92279 (0.0)	-859.78165 (0.0)
6-adduct, conf3	C 1.44853 -0.98266 0.22168<br>C 2.03206 0.31572 -0.22169	C_1	-859.76597 (2.1)	-859.57514 (2.2)	-859.62452 (2.5)	-859.92011 (1.7)	-859.77866 (1.9)

	C 1.19117 1.50673 -0.13266<br>C -0.09279 1.45278 0.25905<br>C -0.71523 0.12348 0.62091<br>C 0.17496 -1.07105 0.62890<br>H 1.66417 2.45256 -0.37550<br>H -1.25746 0.20982 1.56791<br>H -0.22088 -2.00984 0.99974<br>O 2.29016 -2.02143 0.21099<br>O 3.19691 0.32889 -0.60550<br>C -0.94727 2.68131 0.37626<br>H -1.52055 2.67375 1.30790<br>H -0.32844 3.57908 0.35452<br>H -1.65891 2.76084 -0.45377<br>S -2.05522 -0.20679 -0.65878<br>C -2.88309 -1.68405 -0.01580<br>H -3.84041 -1.74904 -0.53267<br>H -2.26455 -2.54102 -0.27609<br>H -3.02695 -1.59835 1.05991<br>H 3.14638 -1.69388 -0.11583<br>H -2.95554 0.71150 -0.25951						
6-adduct, conf4	C 1.43730 -0.97600 0.26374<br>C 2.00682 0.29950 -0.25924<br>C 1.17255 1.49711 -0.19097<br>C -0.09523 1.46368 0.25262<br>C -0.71931 0.14094 0.64829<br>C 0.17502 -1.04581 0.70873<br>H 1.64323 2.43248 -0.47495<br>H -1.27440 0.26470 1.58484<br>H -0.20864 -1.96678 1.13238<br>O 2.28049 -2.01435 0.28035<br>O 3.15860 0.29236 -0.68078<br>C -0.92292 2.70186 0.42216<br>H -1.16100 2.84277 1.48171<br>H -0.38249 3.57965 0.06696<br>H -1.87353 2.64147 -0.11624<br>S -2.12004 -0.13293 -0.58499<br>C -2.88043 -1.69592 -0.08273<br>H -3.65554 -1.90202 -0.82065<br>H -2.14478 -2.49652 -0.06093<br>H -3.33169 -1.53307 0.89523<br>H 3.12723 -1.70245 -0.08396<br>H -1.37330 -0.56970 -1.61669	C_1	-859.76695 (1.5)	-859.57660 (1.3)	-859.62704 (0.9)	-859.91967 (2.0)	-859.77975 (1.2)

6-adduct, conf5	C 1.44105 -0.97784 0.25543<br>C 2.01355 0.30317 -0.25002<br>C 1.17859 1.49977 -0.17460<br>C -0.09332 1.46124 0.25637<br>C -0.71631 0.13620 0.64541<br>C 0.17684 -1.05204 0.69407<br>H 1.64988 2.43771 -0.44885<br>H -1.26371 0.25566 1.58716<br>H -0.20812 -1.97717 1.10737<br>O 2.28349 -2.01698 0.26343<br>O 3.16735 0.30049 -0.66619<br>C -0.93147 2.69503 0.40714<br>H -1.28727 2.78496 1.43847<br>H -0.35438 3.58557 0.15631<br>H -1.81557 2.66946 -0.23786<br>S -2.12643 -0.12940 -0.57797<br>C -2.88573 -1.69312 -0.07632<br>H -3.66688 -1.89485 -0.80905<br>H -2.15138 -2.49505 -0.06350<br>H -3.32919 -1.53356 0.90576<br>H 3.13181 -1.70157 -0.09413<br>H -1.38827 -0.56329 -1.61701	C_1	-859.76694 (1.5)	saddle point			
6-adduct, conf6	C 1.74771 -0.68246 0.28663<br>C 1.82619 0.64193 -0.40052<br>C 0.67987 1.53975 -0.27878<br>C -0.44915 1.17458 0.35445<br>C -0.61004 -0.24597 0.83463<br>C 0.62493 -1.08352 0.89914<br>H 0.81541 2.55141 -0.64716<br>H -1.16537 -0.27920 1.77656<br>H 0.57067 -2.04111 1.40485<br>O 2.87301 -1.40394 0.26486<br>O 2.86777 0.93435 -0.97788<br>C -1.52098 2.16364 0.70374<br>H -1.30930 2.55407 1.70586<br>H -1.52895 3.00276 0.00655<br>H -2.51780 1.72482 0.74862<br>S -1.70722 -1.26158 -0.32072<br>C -3.21213 -0.34530 -0.73644<br>H -3.74751 -0.98309 -1.44090<br>H -3.79606 -0.24059 0.17694<br>H -2.97740 0.61493 -1.18995<br>H 3.53289 -0.89180 -0.23461	C_1	-859.76320 (3.8)	-859.57234 (3.9)	-859.62270 (3.6)	-859.91660 (3.9)	-859.77610 (3.5)

	H -1.00575 -1.04617 -1.44825						
6-adduct, conf7	C 1.74689 -0.81218 0.21565<br>C 2.02498 0.58732 -0.21590<br>C 0.93465 1.55399 -0.12854<br>C -0.30769 1.20896 0.24892<br>C -0.61347 -0.22386 0.62033<br>C 0.52540 -1.18565 0.62011<br>H 1.17858 2.58354 -0.36951<br>H -1.14511 -0.25740 1.57669<br>H 0.34650 -2.19403 0.97846<br>O 2.79975 -1.63578 0.20113<br>O 3.15926 0.86627 -0.58993<br>C -1.41377 2.21948 0.34206<br>H -2.04788 2.04380 1.21498<br>H -1.00052 3.22661 0.40937<br>H -2.04744 2.19352 -0.55152<br>S -1.85069 -0.90230 -0.63102<br>C -3.49997 -0.44764 -0.02637<br>H -4.19732 -1.11202 -0.53755<br>H -3.55835 -0.58086 1.05254<br>H -3.69429 0.58212 -0.31651<br>H 3.56210 -1.12157 -0.11778<br>H -1.83926 -2.17331 -0.18982	C_1	-859.76489 (2.8)	-859.57421 (2.8)	-859.62415 (2.7)	-859.91905 (2.3)	-859.77832 (2.1)
6-adduct, conf8	C 1.76312 -0.72165 0.24662<br>C 1.89461 0.64550 -0.33612<br>C 0.75765 1.55118 -0.20356<br>C -0.41021 1.16794 0.34216<br>C -0.59754 -0.26210 0.78103<br>C 0.61339 -1.13669 0.79538<br>H 0.91760 2.57912 -0.51344<br>H -1.11547 -0.29931 1.74451<br>H 0.52711 -2.12263 1.23888<br>O 2.87158 -1.46897 0.20483<br>O 2.96380 0.95730 -0.85053<br>C -1.51848 2.15585 0.58921<br>H -1.12581 3.00473 1.15450<br>H -1.91410 2.55074 -0.35103<br>H -2.34089 1.72790 1.16350<br>S -1.77204 -1.22333 -0.33752<br>C -3.30940 -0.30060 -0.58596<br>H -3.90574 -0.91919 -1.25756<br>H -3.80628 -0.22634 0.38036<br>H -3.12408 0.67528 -1.02655	C_1	-859.76321 (3.8)	-859.57219 (4.0)	-859.62173 (4.2)	-859.91641 (4.0)	-859.77493 (4.2)

	H 3.55892 -0.93647 -0.23264<br>H -1.16056 -0.96386 -1.50727						
6-adduct, conf9	C 1.74090 -0.82917 0.20636<br>C 2.04486 0.57488 -0.19161<br>C 0.96299 1.55229 -0.11539<br>C -0.28970 1.21533 0.23331<br>C -0.61580 -0.21654 0.59103<br>C 0.50796 -1.19495 0.58144<br>H 1.22059 2.58079 -0.34629<br>H -1.14339 -0.24964 1.55004<br>H 0.31152 -2.20739 0.91860<br>O 2.78303 -1.66676 0.19365<br>O 3.19057 0.84706 -0.53453<br>C -1.39379 2.22905 0.30417<br>H -0.98121 3.23810 0.33521<br>H -2.03745 2.17436 -0.58188<br>H -2.01836 2.07986 1.18929<br>S -1.88349 -0.84210 -0.65899<br>C -3.50764 -0.49506 0.07144<br>H -4.22343 -1.10034 -0.48513<br>H -3.51217 -0.75790 1.12780<br>H -3.72074 0.56070 -0.08053<br>H 3.55814 -1.15615 -0.09904<br>H -1.80522 -2.14621 -0.34164	C_1	-859.76493 (2.8)	-859.57446 (2.6)	-859.62477 (2.3)	-859.91911 (2.3)	-859.77895 (1.7)
6-adduct, conf10	C 1.76455 -0.76762 0.23438<br>C 1.96729 0.61799 -0.27407<br>C 0.85125 1.55185 -0.16042<br>C -0.35869 1.18510 0.29677<br>C -0.60316 -0.24905 0.69870<br>C 0.57740 -1.16525 0.70997<br>H 1.05406 2.58304 -0.43145<br>H -1.12539 -0.28556 1.66080<br>H 0.43993 -2.16622 1.10432<br>O 2.84734 -1.55356 0.20452<br>O 3.06904 0.91927 -0.72196<br>C -1.46783 2.18784 0.45338<br>H -2.19388 1.88469 1.21026<br>H -1.05399 3.15562 0.74227<br>H -1.99392 2.33731 -0.49544<br>S -1.81501 -1.13043 -0.44174<br>C -3.44680 -0.35814 -0.29286<br>H -4.10217 -0.94697 -0.93497<br>H -3.75679 -0.46508 0.74599	C_1	-859.76347 (3.7)	-859.57280 (3.6)	-859.62202 (4.0)	-859.91663 (3.9)	-859.77519 (4.1)

	H -3.43337 0.68185 -0.60448<br>H 3.57249 -1.02663 -0.17437<br>H -1.40189 -0.60716 -1.61062						
5-adduct, conf1	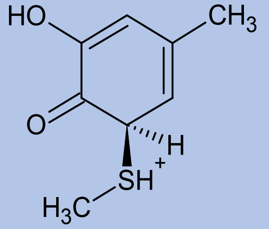<br>[(1*R*)-5-hydroxy-3-methyl-6-oxocyclohexa-2,4-dien-1-yl](methyl)sulfonium<br><br>C -0.22955 1.12080 -0.38813<br>C 1.14224 1.33790 0.07312<br>C 1.98101 0.29303 0.23901<br>C 1.61269 -1.08272 -0.09615<br>C 0.39760 -1.36695 -0.59519<br>C -0.58831 -0.27994 -0.87386<br>H 2.96987 0.47561 0.64908<br>H 0.12968 -2.37659 -0.88890<br>H -0.74151 -0.19406 -1.95950<br>O -1.05057 2.02823 -0.38538<br>O 1.47211 2.59866 0.40577<br>C 2.65517 -2.14058 0.12600<br>H 3.55129 -1.92349 -0.46386<br>H 2.95524 -2.16055 1.17865<br>H 2.28464 -3.12911 -0.15023<br>S -2.25802 -0.73852 -0.25783<br>C -2.02423 -0.75261 1.53749<br>H -2.99972 -0.95660 1.97771<br>H -1.33333 -1.56623 1.75714<br>H -1.63442 0.20442 1.88055<br>H 0.70165 3.16723 0.25259<br>H -2.80719 0.48693 -0.40474	C_1	-859.76311 (3.9)	-859.57339 (3.3)	-859.62397 (2.8)	-859.91460 (5.1)	-859.77546 (3.9)
5-adduct, conf2	C -0.19295 1.17088 -0.43583<br>C 1.15850 1.30472 0.12061<br>C 1.94449 0.22128 0.28708<br>C 1.53940 -1.11953 -0.13785<br>C 0.33210 -1.33331 -0.68964<br>C -0.62360 -0.21401 -0.91987<br>H 2.91919 0.34415 0.74956	C_1	-859.76157 (4.9)	-859.57137 (4.5)	-859.62140 (4.4)	-859.91350 (5.8)	-859.77333 (5.2)

	H 0.04433 -2.31660 -1.04733<br>H -0.86051 -0.11491 -1.98709<br>O -0.94715 2.13097 -0.48549<br>O 1.52051 2.53972 0.50831<br>C 2.54030 -2.22251 0.05068<br>H 3.46884 -1.99094 -0.48044<br>H 2.78931 -2.32782 1.11156<br>H 2.15563 -3.17620 -0.31449<br>S -2.29433 -0.50655 -0.16546<br>C -1.89712 -0.85866 1.56524<br>H -2.78159 -1.32430 1.99861<br>H -1.03329 -1.52038 1.62204<br>H -1.70584 0.09731 2.05064<br>H 0.78332 3.14338 0.32513<br>H -2.46381 -1.76748 -0.60587						
5-adduct, conf3	C 0.18442 1.02260 0.19600<br>C -1.22755 1.35694 -0.01717<br>C -2.15701 0.38368 -0.10547<br>C -1.83305 -1.03273 0.06741<br>C -0.57316 -1.42799 0.32066<br>C 0.52579 -0.43154 0.49871<br>H -3.18656 0.65512 -0.31861<br>H -0.33955 -2.47271 0.49860<br>H 0.90374 -0.46744 1.52922<br>O 1.04956 1.88494 0.13182<br>O -1.50909 2.65910 -0.19795<br>C -2.97223 -2.00570 -0.03264<br>H -3.72787 -1.78570 0.72811<br>H -3.45889 -1.91695 -1.00917<br>H -2.63284 -3.03445 0.09820<br>S 1.96708 -0.86320 -0.58227<br>C 3.41920 -0.23004 0.29900<br>H 4.28877 -0.64811 -0.20809<br>H 3.39411 0.85265 0.19523<br>H 3.38664 -0.53542 1.34316<br>H -0.68045 3.15996 -0.13616<br>H 2.05730 -2.15929 -0.23417	C_1	-859.76008 (5.8)	-859.56984 (5.5)	-859.62030 (5.1)	-859.91125 (7.2)	-859.77147 (6.4)
5-adduct, conf4	C 0.32987 0.96320 0.47475<br>C -0.91436 1.43466 -0.15019<br>C -1.94666 0.59002 -0.34820<br>C -1.90080 -0.81452 0.06264<br>C -0.78039 -1.34546 0.58398<br>C 0.46133 -0.53087 0.74361	C_1	-859.76009 (5.8)	-859.57004 (5.4)	-859.62043 (5.0)	-859.91108 (7.3)	-859.77142 (6.4)

	H -2.85855 0.96768 -0.80080<br>H -0.74010 -2.37908 0.91038<br>H 0.92399 -0.67226 1.72567<br>O 1.22949 1.74696 0.73900<br>O -0.95591 2.73690 -0.48031<br>C -3.16228 -1.61101 -0.10218<br>H -3.98783 -1.13539 0.43665<br>H -3.44518 -1.65262 -1.15910<br>H -3.04196 -2.63030 0.26831<br>S 1.71215 -1.28408 -0.41625<br>C 3.22962 -0.30498 -0.29445<br>H 3.88937 -0.69093 -1.07212<br>H 3.02387 0.75356 -0.43374<br>H 3.65256 -0.50226 0.68963<br>H -0.11473 3.14261 -0.21698<br>H 1.21741 -0.81292 -1.57710						
5-adduct, conf5	C 0.16314 1.02796 0.15424<br>C -1.26087 1.34311 0.00518<br>C -2.17575 0.35423 -0.06566<br>C -1.82062 -1.05938 0.06493<br>C -0.54614 -1.43585 0.26859<br>C 0.53225 -0.41836 0.45241<br>H -3.21658 0.61027 -0.23946<br>H -0.28848 -2.47910 0.41883<br>H 0.88953 -0.44656 1.49132<br>O 1.01742 1.89688 0.04692<br>O -1.57120 2.64224 -0.15032<br>C -2.94295 -2.05332 -0.02183<br>H -3.68334 -1.86107 0.76115<br>H -3.45561 -1.96059 -0.98451<br>H -2.57896 -3.07651 0.08480<br>S 2.01036 -0.79595 -0.59588<br>C 3.42158 -0.25275 0.40428<br>H 4.31700 -0.57608 -0.12619<br>H 3.36910 0.83378 0.44320<br>H 3.36792 -0.69722 1.39634<br>H -0.75005 3.15741 -0.11539<br>H 2.07014 -2.11605 -0.34160	C_1	-859.76011 (5.8)	-859.56989 (5.5)	-859.62018 (5.2)	-859.91139 (7.2)	-859.77146 (6.4)
5-adduct, conf6	C 0.39665 -1.21629 0.37660<br>C 1.71558 -0.79262 -0.11057<br>C 2.00256 0.51468 -0.27324<br>C 1.05740 1.57603 0.07562<br>C -0.18320 1.28516 0.50695	C_1	-859.75933 (6.3)	-859.56896 (6.0)	-859.61974 (5.5)	-859.91121 (7.3)	-859.77162 (6.3)

	C -0.64105 -0.12378 0.65053<br>H 2.97879 0.79668 -0.65592<br>H -0.86329 2.07027 0.81776<br>H -1.05729 -0.31505 1.64678<br>O 0.13252 -2.39973 0.51887<br>O 2.58601 -1.77652 -0.39485<br>C 1.55298 2.98782 -0.04673<br>H 2.44840 3.13233 0.56592<br>H 1.83189 3.19876 -1.08434<br>H 0.79445 3.70766 0.26461<br>S -2.07205 -0.54903 -0.46566<br>C -3.32731 0.68960 -0.06309<br>H -4.13838 0.53204 -0.77416<br>H -3.67060 0.47961 0.94896<br>H -2.92268 1.69491 -0.15463<br>H 2.15198 -2.62548 -0.21461<br>H -1.59036 -0.00094 -1.59841						
5-adduct, conf7	C 0.23718 -1.19561 0.24039<br>C 1.66442 -0.96915 0.00581<br>C 2.14891 0.28430 -0.11798<br>C 1.30999 1.47463 0.02360<br>C -0.00455 1.36447 0.28296<br>C -0.63802 0.02975 0.49308<br>H 3.20407 0.42045 -0.33529<br>H -0.61848 2.24573 0.43693<br>H -1.00716 -0.05914 1.52338<br>O -0.23386 -2.32499 0.24203<br>O 2.42761 -2.06694 -0.13822<br>C 1.99053 2.80597 -0.11480<br>H 2.77424 2.91286 0.64198<br>H 2.47112 2.88659 -1.09502<br>H 1.28218 3.62856 -0.00384<br>S -2.12267 -0.18339 -0.58814<br>C -3.40202 0.77650 0.25886<br>H -4.34363 0.52444 -0.22879<br>H -3.42695 0.52354 1.31723<br>H -3.17693 1.82990 0.09948<br>H 1.85886 -2.84763 -0.04981<br>H -2.44320 -1.42077 -0.15793	C_1	-859.76025 (5.7)	-859.57011 (5.3)	-859.62054 (5.0)	-859.91217 (6.7)	-859.77245 (5.8)
5-adduct, conf8	C 0.36255 -1.21663 0.30356<br>C 1.73283 -0.83195 -0.05511<br>C 2.06980 0.46581 -0.19722<br>C 1.12459 1.55556 0.04647	C_1	-859.75934 (6.3)	-859.56931 (5.8)	-859.62084 (4.8)	-859.91138 (7.2)	-859.77288 (5.5)

	C -0.15076 1.30372 0.39056<br>C -0.64199 -0.09196 0.56802<br>H 3.08351 0.71714 -0.49444<br>H -0.83229 2.11425 0.62335<br>H -1.01590 -0.24955 1.58823<br>O 0.04260 -2.39275 0.37866<br>O 2.59407 -1.84159 -0.27005<br>C 1.65933 2.95309 -0.07848<br>H 2.46401 3.11643 0.64574<br>H 2.08343 3.10874 -1.07557<br>H 0.88004 3.69780 0.09152<br>S -2.13209 -0.49733 -0.46115<br>C -3.35901 0.71891 0.07820<br>H -4.24836 0.53604 -0.52433<br>H -3.57124 0.51219 1.12641<br>H -2.99426 1.73293 -0.06695<br>H 2.12092 -2.67727 -0.13122<br>H -1.73381 0.08403 -1.60909						
5-adduct, conf9	C 0.21685 -1.19051 0.22594<br>C 1.65170 -0.99121 0.01687<br>C 2.16144 0.25281 -0.10149<br>C 1.34218 1.45890 0.01970<br>C 0.02199 1.37383 0.25875<br>C -0.63619 0.05157 0.47307<br>H 3.22244 0.36862 -0.30150<br>H -0.57815 2.26715 0.39638<br>H -0.99953 -0.02633 1.50665<br>O -0.27599 -2.31059 0.21646<br>O 2.39669 -2.10323 -0.11393<br>C 2.04838 2.77716 -0.11603<br>H 2.82330 2.87402 0.65104<br>H 2.54374 2.84444 -1.08987<br>H 1.35338 3.61276 -0.01875<br>S -2.13143 -0.13969 -0.59569<br>C -3.41216 0.77285 0.30045<br>H -4.35550 0.53496 -0.19097<br>H -3.42648 0.47338 1.34664<br>H -3.19595 1.83400 0.18581<br>H 1.81245 -2.87327 -0.03368<br>H -2.42749 -1.39651 -0.20449	C_1	-859.76024 (5.7)	-859.57039 (5.2)	-859.62591 (1.6)	-859.91201 (6.8)	-859.77768 (2.5)

Table S11.6. One-electron oxidized neutral form in vacuo.

Structure	Schematic drawing	Symmetry	E	H_{RRHO}	G_{RRHO}	$E_{\omega B97X\text{-}D,large}$	$G_{RRHO,\omega B97X\text{-}D,large}$
6-adduct, conf1	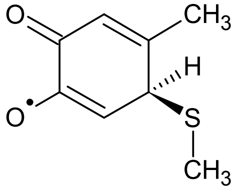<br>[(3*S*)-4-methyl-3-(methylsulfanyl)-6-oxocyclohexa-1,4-dien-1-yl]oxidanyl<br><br>C -1.61101 -0.64231 -0.51244<br>C -1.70773 0.63661 0.33775<br>C -0.56222 1.56287 0.28501<br>C 0.60618 1.27351 -0.31442<br>C 0.84466 -0.07817 -0.93513<br>C -0.36853 -0.89526 -1.16376<br>H -0.71935 2.52433 0.76652<br>H 1.40521 0.03379 -1.87085<br>H -0.29206 -1.78423 -1.78283<br>O -2.58338 -1.39493 -0.62839<br>O -2.70137 0.86767 0.99462<br>C 1.74249 2.25017 -0.37589<br>H 1.97672 2.48947 -1.41978<br>H 1.49916 3.17824 0.14430<br>H 2.64575 1.82005 0.06840<br>S 2.02996 -1.09060 0.08732<br>C 1.12204 -1.23340 1.65033<br>H 1.76562 -1.81451 2.31371<br>H 0.94313 -0.25435 2.09954<br>H 0.17905 -1.76933 1.52523	C_1	-858.69999 (5.2)	-858.53391 (5.5)	-858.58418 (6.2)	-858.83917 (5.0)	-858.72336 (6.1)
6-adduct, conf2	C 1.48160 -1.10508 0.27294<br>C 2.07289 0.21161 -0.25621<br>C 1.23898 1.42053 -0.12132<br>C -0.03949 1.40776 0.29186<br>C -0.74984 0.11047 0.59132<br>C 0.12843 -1.07407 0.72480<br>H 1.73500 2.35582 -0.36581<br>H -1.38158 0.23490 1.47901<br>H -0.28365 -1.99797 1.12018	C_1	-858.69522 (8.2)	-858.52941 (8.3)	-858.58117 (8.1)	-858.83440 (8.0)	-858.72035 (8.0)

	O 2.17020 -2.12687 0.32706<br>O 3.19005 0.24371 -0.72720<br>C -0.83951 2.66416 0.47391<br>H -1.07963 2.80401 1.53463<br>H -0.28752 3.54051 0.12986<br>H -1.78700 2.60638 -0.07052<br>S -1.92472 -0.23232 -0.81660<br>C -3.10970 -1.32265 0.01552<br>H -3.87653 -1.56492 -0.72281<br>H -2.64243 -2.25338 0.34342<br>H -3.58319 -0.81926 0.86192						
6-adduct, conf3	C 1.75294 -0.96829 0.22410<br>C 2.12872 0.46196 -0.19461<br>C 1.07711 1.48841 -0.08667<br>C -0.19883 1.22771 0.24498<br>C -0.67185 -0.17872 0.51563<br>C 0.39622 -1.20545 0.59289<br>H 1.39979 2.50469 -0.29583<br>H -1.24741 -0.19178 1.45085<br>H 0.12910 -2.21048 0.90568<br>O 2.60758 -1.85834 0.25924<br>O 3.25239 0.72072 -0.57109<br>C -1.23076 2.31174 0.35418<br>H -2.01668 2.16623 -0.39483<br>H -1.70650 2.29503 1.34156<br>H -0.78718 3.29698 0.20119<br>S -1.88767 -0.74046 -0.77997<br>C -3.44236 -0.64107 0.15145<br>H -4.23423 -0.94360 -0.53672<br>H -3.44016 -1.32548 1.00274<br>H -3.64091 0.37905 0.48797	C_1	-858.69407 (8.9)	-858.52838 (8.9)	-858.57990 (8.9)	-858.83320 (8.8)	-858.71902 (8.8)
5-adduct, conf1	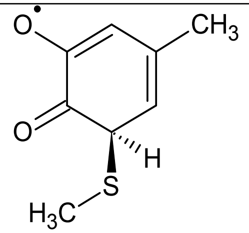<br>[(5*R*)-3-methyl-5-(methylsulfanyl)-6-oxocyclohexa-1,3-dien-1-yl]oxidanyl<br><br>C -0.69152 1.03903 -0.67104	C_1	-858.70648 (1.1)	-858.54060 (1.3)	-858.59142 (1.7)	-858.84533 (1.2)	-858.73027 (1.7)

	C 0.37651 1.57066 0.30514<br>C 1.58732 0.79214 0.41508<br>C 1.76647 -0.45647 -0.21500<br>C 0.73121 -1.03930 -0.91497<br>C -0.63120 -0.44510 -1.00520<br>H 2.38617 1.22667 1.01019<br>H 0.87178 -2.00834 -1.38536<br>H -1.06069 -0.57638 -2.00306<br>O -1.52332 1.77318 -1.14339<br>O 0.18721 2.61661 0.91331<br>C 3.10384 -1.14452 -0.09573<br>H 3.89995 -0.51339 -0.50184<br>H 3.34281 -1.34016 0.95428<br>H 3.11479 -2.09674 -0.63028<br>S -1.81162 -1.39693 0.05688<br>C -1.18380 -1.05525 1.72498<br>H -1.76044 -1.69695 2.39407<br>H -0.12683 -1.31440 1.81879<br>H -1.34576 -0.01492 2.01424						
5-adduct, conf2	C -0.70650 1.02917 -0.67561<br>C 0.34855 1.57697 0.30669<br>C 1.57564 0.81965 0.42031<br>C 1.77576 -0.42069 -0.20525<br>C 0.74976 -1.02268 -0.91498<br>C -0.62191 -0.45384 -1.00770<br>H 2.36230 1.27082 1.01830<br>H 0.91159 -1.98955 -1.38388<br>H -1.05093 -0.59481 -2.00441<br>O -1.54604 1.75092 -1.15328<br>O 0.13716 2.61613 0.91677<br>C 3.11394 -1.11301 -0.09734<br>H 3.56282 -1.24233 -1.08677<br>H 3.81061 -0.53867 0.51648<br>H 3.00382 -2.10595 0.34864<br>S -1.78588 -1.42371 0.05869<br>C -1.16463 -1.06655 1.72619<br>H -1.73267 -1.71409 2.39700<br>H -0.10398 -1.30913 1.82255<br>H -1.34213 -0.02761 2.01126	C_1	-858.70586 (1.5)	-858.53997 (1.7)	-858.59098 (1.9)	-858.84468 (1.6)	-858.72980 (2.0)
5-adduct, conf3	C -0.16742 1.19205 -0.15729<br>C 1.35782 1.36825 0.01157<br>C 2.13637 0.16142 0.14695<br>C 1.59073 -1.12665 -0.01228	C_1	-858.70337 (3.1)	-858.53803 (2.9)	-858.58937 (2.9)	-858.84171 (3.5)	-858.72770 (3.3)

	C 0.28267 -1.28495 -0.41654<br>C -0.62622 -0.14173 -0.76099<br>H 3.18303 0.29052 0.40908<br>H -0.12781 -2.28142 -0.55452<br>H -0.55844 0.01704 -1.85106<br>O -0.92107 2.07115 0.17022<br>O 1.83377 2.49350 0.08139<br>C 2.47321 -2.32595 0.23176<br>H 3.29778 -2.34556 -0.48773<br>H 2.91158 -2.28645 1.23321<br>H 1.91749 -3.26114 0.13613<br>S -2.38788 -0.51880 -0.50195<br>C -2.43343 -0.59260 1.30948<br>H -3.43497 -0.93147 1.58077<br>H -1.70493 -1.31121 1.69427<br>H -2.26167 0.39432 1.74221						
5-adduct, conf4	C 0.48000 0.93229 0.61158<br>C -0.63322 1.52073 -0.27668<br>C -1.82173 0.70964 -0.42277<br>C -1.94060 -0.59138 0.10629<br>C -0.85796 -1.20245 0.70302<br>C 0.49244 -0.57466 0.75450<br>H -2.65743 1.17024 -0.94254<br>H -0.95085 -2.20937 1.09926<br>H 1.03890 -0.85319 1.66017<br>O 1.31151 1.63772 1.13354<br>O -0.51332 2.63764 -0.75933<br>C -3.26314 -1.30651 -0.01197<br>H -4.06730 -0.71357 0.43341<br>H -3.51443 -1.46727 -1.06499<br>H -3.23882 -2.27946 0.48338<br>S 1.44956 -1.20696 -0.70268<br>C 3.11621 -0.64158 -0.26720<br>H 3.76417 -0.94127 -1.09345<br>H 3.15115 0.44428 -0.15836<br>H 3.46406 -1.11837 0.65161	C_1	-858.70823 (0.0)	-858.54261 (0.0)	-858.59405 (0.0)	-858.84721 (0.0)	-858.73303 (0.0)
5-adduct, conf5	C 0.40464 -1.30315 0.54166<br>C 1.60216 -0.87960 -0.33173<br>C 1.85035 0.54038 -0.42987<br>C 1.00992 1.50950 0.14788<br>C -0.17781 1.13651 0.74371<br>C -0.69154 -0.26247 0.74355<br>H 2.75995 0.83515 -0.94606	C_1	-858.70156 (4.2)	-858.53592 (4.2)	-858.58856 (3.4)	-858.84037 (4.3)	-858.72736 (3.6)

	H -0.80561 1.89596 1.20146<br>H -1.21772 -0.50279 1.67191<br>O 0.34536 -2.40033 1.03627<br>O 2.31195 -1.72898 -0.85192<br>C 1.42279 2.95933 0.09210<br>H 2.40680 3.10069 0.54873<br>H 1.48987 3.29744 -0.94678<br>H 0.70901 3.60127 0.61315<br>S -1.88811 -0.54249 -0.65185<br>C -3.12233 0.72772 -0.26688<br>H -3.93937 0.57734 -0.97545<br>H -3.51355 0.60335 0.74614<br>H -2.72718 1.73651 -0.40085						
5-adduct, conf6	C 0.23253 -1.28297 -0.04243<br>C 1.74221 -1.06228 0.18941<br>C 2.20903 0.29956 0.08797<br>C 1.33844 1.39570 -0.06476<br>C -0.02608 1.21698 0.01435<br>C -0.66673 -0.10259 0.32426<br>H 3.28598 0.44416 0.09269<br>H -0.68240 2.07367 -0.10536<br>H -0.78169 -0.17801 1.42054<br>O -0.18148 -2.32606 -0.47615<br>O 2.47555 -2.02195 0.38398<br>C 1.92553 2.76547 -0.29954<br>H 2.63165 3.02435 0.49510<br>H 2.47212 2.79099 -1.24721<br>H 1.15120 3.53484 -0.33247<br>S -2.32647 -0.32530 -0.40611<br>C -3.23220 0.91036 0.56439<br>H -4.28402 0.80182 0.29260<br>H -3.13240 0.72022 1.63644<br>H -2.92582 1.93534 0.34087	C_1	-858.69958 (5.4)	-858.53445 (5.1)	-858.58626 (4.9)	-858.83830 (5.6)	-858.72498 (5.0)

Table S11.7. One-electron oxidized neutral form in water.

Structure	Schematic drawing	Symmetry	G_{PCM}	$H_{PCM,RRHO}$	$G_{PCM,RRHO}$	$G_{SMD,\omega B97X\text{-}D,large}$	$G_{SMD,RRHO,\omega B97X\text{-}D,large}$
6-adduct, conf1	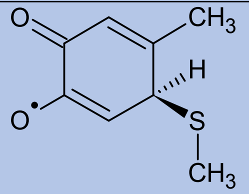<br>[(3*S*)-4-methyl-3-(methylsulfanyl)-6-oxocyclohexa-1,4-dien-1-yl]oxidanyl<br><br>C -1.60218 -0.67824 -0.48045<br>C -1.72218 0.62241 0.32696<br>C -0.59412 1.55180 0.28103<br>C 0.57851 1.27008 -0.32099<br>C 0.82910 -0.07792 -0.94077<br>C -0.36375 -0.92967 -1.12907<br>H -0.75385 2.51518 0.75677<br>H 1.36186 0.03776 -1.89101<br>H -0.27372 -1.82273 -1.73956<br>O -2.57377 -1.44541 -0.56890<br>O -2.74802 0.85702 0.94712<br>C 1.69789 2.26126 -0.38928<br>H 1.92898 2.48606 -1.43644<br>H 1.44012 3.18981 0.12163<br>H 2.60584 1.84709 0.05993<br>S 2.04877 -1.06724 0.06095<br>C 1.21891 -1.16397 1.67040<br>H 1.88952 -1.73917 2.31134<br>H 1.07605 -0.17328 2.10558<br>H 0.26618 -1.69223 1.60381	C_1	-858.71726 (3.9)	-858.55120 (4.0)	-858.60176 (4.3)	-858.85986 (2.7)	-858.74436 (3.1)
6-adduct, conf2	C 1.50097 -1.08042 0.28302<br>C 2.06361 0.24668 -0.24545<br>C 1.21519 1.43317 -0.13461<br>C -0.06526 1.39313 0.28236<br>C -0.74218 0.08411 0.59075<br>C 0.15313 -1.08284 0.73248<br>H 1.68527 2.37864 -0.38933<br>H -1.39612 0.19586 1.46110<br>H -0.24896 -2.00612 1.13820	C_1	-858.71475 (5.5)	-858.54864 (5.7)	-858.59940 (5.8)	-858.85675 (4.7)	-858.74140 (4.9)

	O 2.22779 -2.08477 0.32949<br>O 3.19486 0.28532 -0.70467<br>C -0.89044 2.62956 0.46023<br>H -1.08595 2.78569 1.52723<br>H -0.38147 3.51078 0.06736<br>H -1.86012 2.52487 -0.03532<br>S -1.86487 -0.34048 -0.83697<br>C -3.20282 -1.19620 0.03917<br>H -3.92523 -1.49578 -0.72202<br>H -2.83716 -2.08946 0.54789<br>H -3.68674 -0.52445 0.75011						
5-adduct, conf1	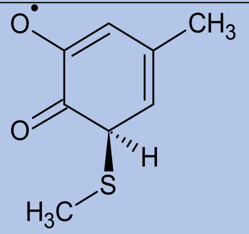<br>[(5*R*)-3-methyl-5-(methylsulfanyl)-6-oxocyclohexa-1,3-dien-1-yl]oxidanyl<br><br>C -0.66944 1.07416 -0.62396<br>C 0.45209 1.57286 0.30370<br>C 1.61962 0.74777 0.40764<br>C 1.74757 -0.50376 -0.22868<br>C 0.68672 -1.03915 -0.92899<br>C -0.65099 -0.39366 -1.00538<br>H 2.44330 1.14226 0.99615<br>H 0.78779 -2.00460 -1.41501<br>H -1.07875 -0.48979 -2.00788<br>O -1.52713 1.83490 -1.01502<br>O 0.31985 2.65394 0.88189<br>C 3.05533 -1.24323 -0.11904<br>H 3.87343 -0.63646 -0.51749<br>H 3.28296 -1.45556 0.92990<br>H 3.02643 -2.18855 -0.66366<br>S -1.86988 -1.32482 0.03417<br>C -1.17302 -1.15586 1.70215<br>H -1.78450 -1.79652 2.34011<br>H -0.14068 -1.50838 1.74091<br>H -1.24092 -0.13080 2.07070	C_1	-858.72291 (0.3)	-858.55703 (0.4)	-858.60784 (0.5)	-858.86419 (0.0)	-858.74912 (0.1)

5-adduct, conf2	C 0.46545 0.93848 0.60189<br>C -0.66565 1.50977 -0.27135<br>C -1.83450 0.68716 -0.41874<br>C -1.93308 -0.61700 0.10721<br>C -0.83791 -1.21176 0.70043<br>C 0.50008 -0.56096 0.75389<br>H -2.68212 1.13083 -0.93353<br>H -0.91137 -2.21808 1.10025<br>H 1.05534 -0.84082 1.65267<br>O 1.29517 1.66925 1.10159<br>O -0.55879 2.64355 -0.73957<br>C -3.24376 -1.35032 -0.00618<br>H -4.05062 -0.77023 0.45025<br>H -3.50055 -1.50248 -1.05885<br>H -3.19794 -2.32578 0.48111<br>S 1.46225 -1.16060 -0.71672<br>C 3.14073 -0.67122 -0.23865<br>H 3.79210 -0.97945 -1.05852<br>H 3.21444 0.41027 -0.10938<br>H 3.44549 -1.18197 0.67638	C_1	-858.72345 (0.0)	-858.55764 (0.0)	-858.60860 (0.0)	-858.86409 (0.1)	-858.74924 (0.0)
5-adduct, conf3	C 0.32711 -1.30250 0.49175<br>C 1.62219 -0.94471 -0.25560<br>C 1.94873 0.44689 -0.35838<br>C 1.11838 1.47394 0.12619<br>C -0.12216 1.17618 0.65492<br>C -0.69537 -0.19728 0.68983<br>H 2.90570 0.68914 -0.81204<br>H -0.74568 1.97758 1.03885<br>H -1.22439 -0.38625 1.62848<br>O 0.13599 -2.43129 0.88581<br>O 2.33254 -1.85305 -0.69137<br>C 1.60269 2.89839 0.04819<br>H 2.54565 3.01348 0.59023<br>H 1.78604 3.17818 -0.99356<br>H 0.87311 3.59329 0.46770<br>S -1.94131 -0.42042 -0.67458<br>C -3.20240 0.77262 -0.14711<br>H -4.05984 0.61014 -0.80268<br>H -3.50311 0.58342 0.88508<br>H -2.85973 1.80138 -0.26308	C_1	-858.71931 (2.6)	-858.55346 (2.6)	-858.60588 (1.7)	-858.85989 (2.7)	-858.74646 (1.7)
5-adduct, conf4	C 0.23863 -1.29314 0.08797<br>C 1.75763 -1.03709 0.09000<br>C 2.19600 0.32101 -0.02390	C_1	-858.71817 (3.3)	-858.55262 (3.2)	-858.60444 (2.6)	-858.85900 (3.3)	-858.74527 (2.5)

	C 1.31610 1.41917 -0.02567<br>C -0.03684 1.22632 0.15410<br>C -0.67027 -0.10766 0.38293<br>H 3.26557 0.48064 -0.12719<br>H -0.69407 2.08970 0.18007<br>H -0.89899 -0.19823 1.45815<br>O -0.18131 -2.40879 -0.11973<br>O 2.52155 -2.00314 0.13782<br>C 1.88245 2.80380 -0.20568<br>H 2.62193 3.01814 0.57123<br>H 2.38745 2.88548 -1.17266<br>H 1.10170 3.56492 -0.15934<br>S -2.26452 -0.30392 -0.50724<br>C -3.29704 0.85070 0.43654<br>H -4.30990 0.73601 0.04637<br>H -3.29502 0.59452 1.49798<br>H -2.98820 1.88831 0.29876						

Part 12: 4-Methylcatechol – methanethiol rearomatized adducts, optimizations using the ωB97X-D functional

Table S12.1. Reduced neutral form in vacuo.

Structure	Schematic drawing	Symmetry	E	H_{RRHO}	G_{RRHO}	$E_{\omega B97X\text{-}D,large}$	$G_{RRHO,\omega B97X\text{-}D,large}$
6-adduct, conf1	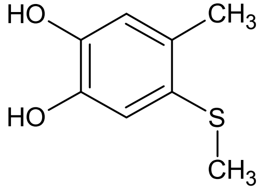<br>4-methyl-5-(methylsulfanyl)benzene-1,2-diol<br><br>C -1.63982 -0.84329 -0.03281<br>C -1.99328 0.50313 0.09981<br>C -1.00575 1.47564 0.03965<br>C 0.34065 1.14744 -0.15108<br>C 0.68466 -0.20956 -0.26868<br>C -0.31227 -1.19248 -0.21088<br>H -1.30776 2.51394 0.13687<br>H -0.03318 -2.23830 -0.31042<br>O -2.68097 -1.73441 0.03179<br>O -3.28777 0.86484 0.27881<br>C 1.36568 2.25101 -0.21062<br>H 0.89027 3.20654 -0.44487<br>H 1.87848 2.36822 0.75085<br>H 2.12832 2.04424 -0.96516<br>S 2.36429 -0.75217 -0.53993<br>C 3.05103 -0.57834 1.13629<br>H 4.07347 -0.96114 1.10129<br>H 3.07577 0.46692 1.45072<br>H 2.47205 -1.16677 1.85068<br>H -3.83271 0.06857 0.28056<br>H -2.36877 -2.63231 -0.10662	C_1	-859.37445 (3.9)	-859.19474 (3.6)	-859.24562 (3.3)	-859.51485 (4.1)	-859.38602 (3.4)
6-adduct, conf2	C -1.62017 -0.84961 -0.05766<br>C -1.99194 0.48738 0.11104<br>C -1.01878 1.47648 0.07334<br>C 0.33069 1.17167 -0.12915<br>C 0.69287 -0.17680 -0.28334<br>C -0.28816 -1.17526 -0.25061	C_1	-859.37443 (3.9)	-859.19477 (3.6)	-859.24569 (3.2)	-859.51477 (4.1)	-859.38604 (3.4)

	H -1.33643 2.50691 0.19898<br>H 0.00551 -2.21366 -0.38224<br>O -2.64892 -1.75650 -0.01293<br>O -3.29084 0.82476 0.30426<br>C 1.34459 2.28620 -0.18686<br>H 2.08065 2.20762 0.61950<br>H 1.90069 2.26290 -1.12807<br>H 0.85401 3.25830 -0.09731<br>S 2.38812 -0.67112 -0.55263<br>C 2.98290 -0.71980 1.16733<br>H 4.03535 -1.01037 1.13473<br>H 2.90050 0.26058 1.64144<br>H 2.42407 -1.45577 1.74878<br>H -3.82278 0.01977 0.28832<br>H -2.32548 -2.64598 -0.17731						
6-adduct, conf3	C -1.62170 -0.88735 -0.04650<br>C -1.98620 0.45403 0.09730<br>C -1.02274 1.45068 0.04633<br>C 0.33040 1.15110 -0.14696<br>C 0.69130 -0.20015 -0.27931<br>C -0.28447 -1.20198 -0.23294<br>H -1.32801 2.48974 0.15495<br>H -0.00517 -2.24379 -0.34640<br>O -2.55358 -1.87493 -0.00573<br>O -3.32444 0.68205 0.27953<br>C 1.33814 2.27115 -0.19137<br>H 0.84445 3.23178 -0.35983<br>H 1.89074 2.34729 0.75192<br>H 2.07301 2.11186 -0.98396<br>S 2.38678 -0.69880 -0.54803<br>C 3.02032 -0.62290 1.15610<br>H 4.06053 -0.95468 1.12452<br>H 2.98587 0.39508 1.54995<br>H 2.45028 -1.28942 1.80616<br>H -3.50431 1.62361 0.34287<br>H -3.42195 -1.47513 0.12196	C_1	-859.37372 (4.4)	-859.19394 (4.1)	-859.24495 (3.7)	-859.51412 (4.5)	-859.38535 (3.8)
6-adduct, conf4	C -1.60636 -0.89228 -0.06598<br>C -1.98568 0.44145 0.10486<br>C -1.03356 1.45064 0.07452<br>C 0.32193 1.16978 -0.12574<br>C 0.69738 -0.17416 -0.28628<br>C -0.26551 -1.18796 -0.26034<br>H -1.35128 2.48331 0.20524	C_1	-859.37376 (4.4)	-859.19398 (4.1)	-859.24421 (4.2)	-859.51411 (4.5)	-859.38456 (4.3)

	H 0.02562 -2.22379 -0.39741<br>O -2.52677 -1.89129 -0.04466<br>O -3.32610 0.65072 0.29341<br>C 1.31984 2.29907 -0.17146<br>H 2.07226 2.20739 0.61800<br>H 1.85892 2.30976 -1.12277<br>H 0.82015 3.26327 -0.04689<br>S 2.40383 -0.63487 -0.55535<br>C 2.97417 -0.73404 1.17064<br>H 4.03306 -1.00082 1.14376<br>H 2.86352 0.22667 1.67855<br>H 2.42306 -1.50337 1.71510<br>H -3.51705 1.58899 0.37144<br>H -3.39988 -1.50399 0.08913						
6-adduct, conf5	C -0.74762 -1.54372 0.00000<br>C 0.57564 -1.98734 0.00000<br>C 1.59335 -1.04770 0.00000<br>C 1.33578 0.32610 0.00000<br>C 0.00000 0.75935 0.00000<br>C -1.03285 -0.18695 0.00000<br>H 2.61923 -1.40490 0.00000<br>H -2.07354 0.12092 0.00000<br>O -1.70063 -2.53129 0.00000<br>O 0.87045 -3.31458 0.00000<br>C 2.51473 1.27107 0.00000<br>H 2.21888 2.32132 0.00000<br>H 3.13860 1.10470 0.88382<br>H 3.13860 1.10470 -0.88382<br>S -0.31875 2.51505 0.00000<br>C -2.12192 2.61457 0.00000<br>H -2.35292 3.68153 0.00000<br>H -2.55516 2.16554 -0.89732<br>H -2.55516 2.16554 0.89732<br>H 0.04403 -3.81179 0.00000<br>H -2.58375 -2.15372 0.00000	C_s	-859.37067 (6.3)	saddle point			
6-adduct, conf6	C 0.76352 -1.54440 0.00000<br>C -0.55549 -1.99693 0.00000<br>C -1.58440 -1.06617 0.00000<br>C -1.33520 0.30703 0.00000<br>C 0.00000 0.74926 0.00000<br>C 1.04107 -0.18435 0.00000<br>H -2.60574 -1.43452 0.00000<br>H 2.07897 0.13374 0.00000	C_s	-859.37316 (4.7)	-859.19336 (4.5)	-859.24403 (4.3)	-859.51409 (4.5)	-859.38496 (4.1)

	O 1.72375 -2.52540 0.00000<br>O -0.84067 -3.32653 0.00000<br>C -2.48366 1.28247 0.00000<br>H -3.44030 0.75527 0.00000<br>H -2.45549 1.93250 0.88198<br>H -2.45549 1.93250 -0.88198<br>S 0.27386 2.50831 0.00000<br>C 2.07439 2.65027 0.00000<br>H 2.28448 3.72154 0.00000<br>H 2.51604 2.20877 0.89708<br>H 2.51604 2.20877 -0.89708<br>H -0.01048 -3.81737 0.00000<br>H 2.60418 -2.14174 0.00000						
5-adduct, conf1	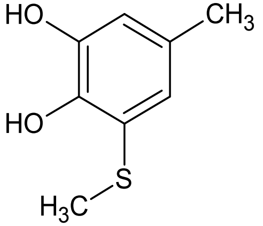<br>5-methyl-3-(methylsulfanyl)benzene-1,2-diol<br><br>C -0.09162 1.00319 -0.13879<br>C 1.27195 1.23739 0.08522<br>C 2.15233 0.16970 0.15074<br>C 1.70257 -1.14766 -0.01004<br>C 0.34745 -1.37032 -0.23480<br>C -0.55532 -0.30123 -0.29160<br>H 3.20381 0.37921 0.32363<br>H -0.02845 -2.37964 -0.37357<br>O -0.89073 2.10537 -0.18350<br>O 1.72993 2.50609 0.23708<br>C 2.68380 -2.29169 0.05672<br>H 3.45633 -2.19357 -0.71288<br>H 3.18879 -2.32164 1.02767<br>H 2.18346 -3.25205 -0.09116<br>S -2.29637 -0.55400 -0.57834<br>C -2.87322 -0.76136 1.13891<br>H -3.95591 -0.89811 1.09879<br>H -2.41396 -1.64410 1.58672<br>H -2.64120 0.12126 1.73775	C_1	-859.38070<br>(0.0)	-859.20051<br>(0.0)	-859.25029<br>(0.4)	-859.52132<br>(0.0)	-859.39090<br>(0.4)

	H 0.98686 3.11403 0.13861<br>H -1.77910 1.81890 -0.44886						
5-adduct, conf2	C -0.09279 1.00200 -0.14246<br>C 1.26883 1.23968 0.08581<br>C 2.15150 0.17362 0.15954<br>C 1.70531 -1.14474 0.00271<br>C 0.35035 -1.37130 -0.22312<br>C -0.55370 -0.30466 -0.28925<br>H 3.20158 0.38649 0.33647<br>H -0.02323 -2.38237 -0.35579<br>O -0.89472 2.10198 -0.19364<br>O 1.72410 2.50956 0.23618<br>C 2.68974 -2.28717 0.04666<br>H 2.18139 -3.24432 0.18893<br>H 3.25881 -2.34799 -0.88743<br>H 3.40840 -2.16122 0.86191<br>S -2.29377 -0.56235 -0.57827<br>C -2.87573 -0.75447 1.13886<br>H -3.95806 -0.89338 1.09638<br>H -2.41647 -1.63217 1.59644<br>H -2.64737 0.13414 1.73022<br>H 0.98026 3.11592 0.13398<br>H -1.78125 1.81234 -0.46165	C_1	-859.38064 (0.0)	-859.20051 (0.0)	-859.25087 (0.0)	-859.52125 (0.0)	-859.39148 (0.0)
5-adduct, conf3	C -0.20542 0.90546 -0.15538<br>C 1.13078 1.28839 0.01983<br>C 2.13920 0.34449 0.11698<br>C 1.83871 -1.02144 0.04852<br>C 0.50806 -1.39762 -0.10567<br>C -0.52150 -0.45203 -0.21014<br>H 3.16934 0.66962 0.25161<br>H 0.24477 -2.45022 -0.14502<br>O -1.17161 1.85162 -0.26903<br>O 1.33447 2.64437 0.07174<br>C 2.93787 -2.04793 0.16552<br>H 3.69744 -1.90661 -0.61055<br>H 3.44065 -1.98027 1.13604<br>H 2.54073 -3.06081 0.06376<br>S -2.18816 -1.00519 -0.48756<br>C -3.03494 -0.31310 0.96566<br>H -4.08168 -0.61393 0.88478<br>H -2.61362 -0.72069 1.88665<br>H -2.96852 0.77535 0.96444<br>H 2.27259 2.84603 0.11523	C_1	-859.37363 (4.4)	-859.19362 (4.3)	-859.24359 (4.6)	-859.51379 (4.7)	-859.38374 (4.9)

	H -0.75038 2.71941 -0.23964						
5-adduct, conf4	C -0.20584 0.90445 -0.15849<br>C 1.12857 1.28982 0.02075<br>C 2.13821 0.34739 0.12606<br>C 1.84019 -1.01869 0.06092<br>C 0.50981 -1.39762 -0.09343<br>C -0.52020 -0.45417 -0.20662<br>H 3.16701 0.67476 0.26495<br>H 0.24800 -2.45086 -0.12601<br>O -1.17338 1.84854 -0.27859<br>O 1.33037 2.64619 0.07162<br>C 2.94232 -2.04457 0.15218<br>H 2.53747 -3.03919 0.35469<br>H 3.50544 -2.10002 -0.78600<br>H 3.65102 -1.79922 0.94911<br>S -2.18546 -1.01130 -0.48425<br>C -3.03863 -0.30825 0.95998<br>H -4.08433 -0.61242 0.87808<br>H -2.61946 -0.70686 1.88585<br>H -2.97474 0.78027 0.94931<br>H 2.26816 2.84912 0.11619<br>H -0.75379 2.71716 -0.25047	C_1	-859.37360 (4.5)	-859.19365 (4.3)	-859.24457 (4.0)	-859.51375 (4.7)	-859.38472 (4.2)
5-adduct, conf5	C 1.13992 0.17783 0.00000<br>C 1.01774 1.57103 0.00000<br>C -0.23648 2.16331 0.00000<br>C -1.38799 1.37293 0.00000<br>C -1.26055 -0.01547 0.00000<br>C 0.00000 -0.62493 0.00000<br>H -0.29953 3.24697 0.00000<br>H -2.15849 -0.62309 0.00000<br>O 2.42978 -0.28802 0.00000<br>O 2.13044 2.34740 0.00000<br>C -2.74967 2.02303 0.00000<br>H -2.88287 2.65652 0.88280<br>H -2.88287 2.65652 -0.88280<br>H -3.54734 1.27566 0.00000<br>S 0.27136 -2.38183 0.00000<br>C -1.39901 -3.06776 0.00000<br>H -1.27124 -4.15146 0.00000<br>H -1.94686 -2.77420 0.89759<br>H -1.94686 -2.77420 -0.89759<br>H 2.90485 1.77140 0.00000<br>H 2.46390 -1.24968 0.00000	C_s	-859.37418 (4.1)	saddle point			

5-adduct, conf6	C -1.14141 0.18332 0.00000<br>C -1.01680 1.57305 0.00000<br>C 0.24132 2.16459 0.00000<br>C 1.38893 1.37435 0.00000<br>C 1.25878 -0.01707 0.00000<br>C 0.00000 -0.62275 0.00000<br>H 0.30398 3.24793 0.00000<br>H 2.15715 -0.62491 0.00000<br>O -2.43153 -0.28270 0.00000<br>O -2.12602 2.35466 0.00000<br>C 2.76143 2.00241 0.00000<br>H 3.33368 1.70083 0.88347<br>H 3.33368 1.70083 -0.88347<br>H 2.69620 3.09320 0.00000<br>S -0.27722 -2.37864 0.00000<br>C 1.39108 -3.06910 0.00000<br>H 1.26075 -4.15249 0.00000<br>H 1.93963 -2.77670 -0.89757<br>H 1.93963 -2.77670 0.89757<br>H -2.90295 1.78210 0.00000<br>H -2.46595 -1.24430 0.00000	C_s	-859.37400 (4.2)	saddle point			
5-adduct, conf7	C 1.14731 0.14986 0.00000<br>C 1.02428 1.53992 0.00000<br>C -0.22201 2.14920 0.00000<br>C -1.37978 1.36625 0.00000<br>C -1.25445 -0.02199 0.00000<br>C 0.00000 -0.64517 0.00000<br>H -0.29544 3.23487 0.00000<br>H -2.15708 -0.62229 0.00000<br>O 2.37113 -0.44000 0.00000<br>O 2.21538 2.21948 0.00000<br>C -2.73812 2.02329 0.00000<br>H -2.87183 2.65630 0.88338<br>H -2.87183 2.65630 -0.88338<br>H -3.53730 1.27783 0.00000<br>S 0.26659 -2.39705 0.00000<br>C -1.42238 -3.04235 0.00000<br>H -1.31772 -4.12909 0.00000<br>H -1.97172 -2.74488 0.89648<br>H -1.97172 -2.74488 -0.89648<br>H 2.06400 3.16789 0.00000<br>H 3.04398 0.25095 0.00000	C_s	-859.37290 (4.9)	saddle point			

5-adduct,<br>conf8	C -1.14823 0.15600 0.00000<br>C -1.02275 1.54267 0.00000<br>C 0.22780 2.15037 0.00000<br>C 1.38120 1.36692 0.00000<br>C 1.25302 -0.02425 0.00000<br>C 0.00000 -0.64310 0.00000<br>H 0.30186 3.23567 0.00000<br>H 2.15594 -0.62501 0.00000<br>O -2.37207 -0.43409 0.00000<br>O -2.21095 2.22798 0.00000<br>C 2.75035 2.00183 0.00000<br>H 3.32431 1.70301 0.88321<br>H 3.32431 1.70301 -0.88321<br>H 2.68214 3.09289 0.00000<br>S -0.27381 -2.39375 0.00000<br>C 1.41283 -3.04460 0.00000<br>H 1.30496 -4.13102 0.00000<br>H 1.96303 -2.74856 -0.89645<br>H 1.96303 -2.74856 0.89645<br>H -2.05501 3.17562 0.00000<br>H -3.04481 0.25697 0.00000	C_s	-859.37281<br>(5.0)	saddle point			

Table S12.2. Reduced neutral form in water.

Structure	Schematic drawing	Symmetry	G_{PCM}	$H_{PCM,RRHO}$	$G_{PCM,RRHO}$	$G_{SMD,\omega B97X\text{-}D,large}$	$G_{SMD,RRHO,\omega B97X\text{-}D,large}$
6-adduct,<br>conf1	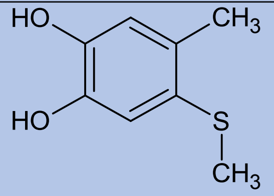<br>4-methyl-5-(methylsulfanyl)benzene-1,2-diol<br><br>C -1.61637 -0.85644 -0.06417<br>C -1.99223 0.48015 0.10804<br>C -1.02645 1.47703 0.07697<br>C 0.32651 1.18199 -0.12410<br>C 0.69263 -0.16446 -0.28881<br>C -0.28101 -1.17129 -0.26260<br>H -1.34634 2.50609 0.21135	C_1	-859.38466<br>(2.1)	saddle point			

	H 0.01342 -2.20822 -0.39638<br>O -2.63774 -1.76442 -0.02218<br>O -3.29625 0.81051 0.30373<br>C 1.33341 2.30415 -0.16084<br>H 0.84095 3.26762 -0.01149<br>H 2.09384 2.19081 0.61786<br>H 1.85831 2.33265 -1.12014<br>S 2.39336 -0.64517 -0.55737<br>C 2.98128 -0.76581 1.16249<br>H 4.03615 -1.04459 1.12086<br>H 2.88558 0.19357 1.67433<br>H 2.42517 -1.53346 1.70336<br>H -3.82628 0.00245 0.29849<br>H -2.30917 -2.66479 -0.11458						
6-adduct, conf2	C -1.61685 -0.85622 -0.06395<br>C -1.99210 0.48052 0.10798<br>C -1.02592 1.47704 0.07653<br>C 0.32687 1.18143 -0.12454<br>C 0.69244 -0.16527 -0.28884<br>C -0.28157 -1.17166 -0.26227<br>H -1.34543 2.50625 0.21068<br>H 0.01243 -2.20879 -0.39560<br>O -2.63848 -1.76385 -0.02166<br>O -3.29591 0.81166 0.30377<br>C 1.33429 2.30305 -0.16186<br>H 2.09482 2.18957 0.61672<br>H 1.85896 2.33091 -1.12131<br>H 0.84235 3.26681 -0.01269<br>S 2.39302 -0.64663 -0.55712<br>C 2.98156 -0.76329 1.16278<br>H 4.03663 -1.04141 1.12160<br>H 2.88531 0.19715 1.67256<br>H 2.42599 -1.53005 1.70549<br>H -3.82646 0.00396 0.29856<br>H -2.31019 -2.66432 -0.11405	C_1	-859.38466 (2.1)	-859.20526 (1.9)	-859.25662 (1.4)	-859.52721 (1.0)	-859.39917 (0.3)
6-adduct, conf3	C -1.63478 -0.88230 -0.03149<br>C -1.99266 0.46454 0.09256<br>C -1.01811 1.45101 0.03176<br>C 0.33381 1.13644 -0.15464<br>C 0.68510 -0.21963 -0.26738<br>C -0.30050 -1.21334 -0.20685<br>H -1.31526 2.49244 0.12688<br>H -0.02928 -2.26000 -0.29736	C_1	-859.38446 (2.2)	-859.20508 (2.0)	-859.25618 (1.7)	-859.52707 (1.0)	-859.39879 (0.5)

	O -2.57759 -1.86449 0.01997<br>O -3.32481 0.70274 0.26527<br>C 1.34584 2.25227 -0.20768<br>H 0.85953 3.20435 -0.43230<br>H 1.86082 2.36638 0.75247<br>H 2.10867 2.06246 -0.96668<br>S 2.37251 -0.74489 -0.53704<br>C 3.08171 -0.54066 1.12710<br>H 4.10695 -0.91348 1.08052<br>H 3.09857 0.50909 1.42428<br>H 2.51777 -1.12722 1.85426<br>H -3.50600 1.64669 0.32495<br>H -3.44520 -1.45833 0.14342						
6-adduct, conf4	C -1.60583 -0.89231 -0.06451<br>C -1.99135 0.44162 0.10441<br>C -1.03835 1.45156 0.07387<br>C 0.31831 1.17094 -0.12625<br>C 0.69663 -0.17259 -0.28688<br>C -0.26553 -1.18885 -0.25823<br>H -1.35813 2.48229 0.20552<br>H 0.02809 -2.22550 -0.38745<br>O -2.52725 -1.89578 -0.04108<br>O -3.32729 0.64499 0.29088<br>C 1.31215 2.30442 -0.16726<br>H 2.07587 2.20121 0.60952<br>H 1.83388 2.33712 -1.12817<br>H 0.80997 3.26300 -0.01839<br>S 2.40342 -0.63370 -0.55568<br>C 2.99581 -0.73306 1.16385<br>H 4.05484 -0.99563 1.12232<br>H 2.88630 0.22826 1.66930<br>H 2.45242 -1.50536 1.71098<br>H -3.52965 1.58245 0.37765<br>H -3.40304 -1.51257 0.09723	C_1	-859.38455 (2.1)	-859.20505 (2.0)	-859.25491 (2.5)	-859.52717 (1.0)	-859.39752 (1.3)
6-adduct, conf5	C -0.74291 -1.54765 0.00000<br>C 0.58523 -1.98219 0.00000<br>C 1.60177 -1.04113 0.00000<br>C 1.33919 0.33338 0.00000<br>C 0.00000 0.75747 0.00000<br>C -1.03185 -0.19090 0.00000<br>H 2.63067 -1.39068 0.00000<br>H -2.07328 0.11089 0.00000<br>O -1.68708 -2.53676 0.00000	C_s	-859.38228 (3.6)	saddle point			

	O 0.88548 -3.31234 0.00000<br>C 2.51536 1.28236 0.00000<br>H 2.21568 2.33161 0.00000<br>H 3.14116 1.11790 0.88262<br>H 3.14116 1.11790 -0.88262<br>S -0.33542 2.51268 0.00000<br>C -2.14130 2.60649 0.00000<br>H -2.37370 3.67279 0.00000<br>H -2.56949 2.15414 -0.89678<br>H -2.56949 2.15414 0.89678<br>H 0.06132 -3.81597 0.00000<br>H -2.57732 -2.16980 0.00000						
6-adduct, conf6	C 0.75821 -1.54818 0.00000<br>C -0.56562 -1.99171 0.00000<br>C -1.59315 -1.05920 0.00000<br>C -1.33870 0.31476 0.00000<br>C 0.00000 0.74785 0.00000<br>C 1.03971 -0.18819 0.00000<br>H -2.61769 -1.42001 0.00000<br>H 2.07846 0.12365 0.00000<br>O 1.70966 -2.53075 0.00000<br>O -0.85635 -3.32430 0.00000<br>C -2.48533 1.29312 0.00000<br>H -3.44306 0.76839 0.00000<br>H -2.45716 1.94339 0.88185<br>H -2.45716 1.94339 -0.88185<br>S 0.29182 2.50603 0.00000<br>C 2.09514 2.64140 0.00000<br>H 2.30710 3.71197 0.00000<br>H 2.53172 2.19637 0.89657<br>H 2.53172 2.19637 -0.89657<br>H -0.02833 -3.82151 0.00000<br>H 2.59712 -2.15723 0.00000	C_s	-859.38472 (2.0)	-859.20525 (1.9)	-859.25568 (2.0)	-859.52862 (0.1)	-859.39958 (0.0)
5-adduct, conf1	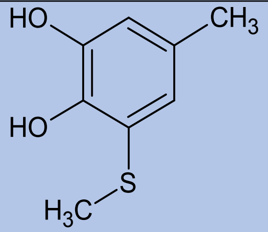<br>5-methyl-3-(methylsulfanyl)benzene-1,2-diol	C_1	-859.38797 (0.0)	-859.20824 (0.0)	-859.25851 (0.2)	-859.52873 (0.0)	-859.39928 (0.2)

	C -0.05450 1.02850 -0.12413<br>C 1.31998 1.20346 0.09170<br>C 2.15990 0.10268 0.14370<br>C 1.65737 -1.19602 -0.02142<br>C 0.29260 -1.36092 -0.24117<br>C -0.56888 -0.25651 -0.28736<br>H 3.22054 0.26631 0.31199<br>H -0.12285 -2.35474 -0.37965<br>O -0.80511 2.16287 -0.15355<br>O 1.83192 2.45475 0.25243<br>C 2.59221 -2.37899 0.03448<br>H 3.37342 -2.29881 -0.72807<br>H 3.09036 -2.44041 1.00735<br>H 2.05519 -3.31639 -0.12910<br>S -2.31827 -0.44308 -0.57723<br>C -2.90244 -0.81608 1.10871<br>H -3.98433 -0.94567 1.04475<br>H -2.44762 -1.74004 1.46846<br>H -2.67435 0.00570 1.78875<br>H 1.11466 3.09782 0.17901<br>H -1.72466 1.91782 -0.34600						
5-adduct, conf2	C -0.05558 1.02761 -0.12846<br>C 1.31695 1.20595 0.09229<br>C 2.15910 0.10645 0.15319<br>C 1.66010 -1.19325 -0.00846<br>C 0.29532 -1.36197 -0.22987<br>C -0.56732 -0.25988 -0.28584<br>H 3.21832 0.27326 0.32690<br>H -0.11796 -2.35764 -0.36206<br>O -0.80922 2.15989 -0.16427<br>O 1.82630 2.45847 0.25222<br>C 2.59775 -2.37494 0.02426<br>H 2.05374 -3.30884 0.18565<br>H 3.14240 -2.46571 -0.92175<br>H 3.34009 -2.26981 0.82070<br>S -2.31602 -0.45109 -0.57761<br>C -2.90380 -0.80984 1.11016<br>H -3.98551 -0.94017 1.04474<br>H -2.44960 -1.73061 1.47874<br>H -2.67744 0.01778 1.78365<br>H 1.10801 3.10010 0.17633<br>H -1.72756 1.91152 -0.35829	C_1	-859.38791 (0.0)	-859.20821 (0.0)	-859.25888 (0.0)	-859.52866 (0.0)	-859.39963 (0.0)

5-adduct, conf3	C -0.18951 0.91979 -0.16510<br>C 1.15307 1.28014 0.02547<br>C 2.14142 0.31405 0.12442<br>C 1.81575 -1.04656 0.04246<br>C 0.48023 -1.39970 -0.13070<br>C -0.52863 -0.43102 -0.23575<br>H 3.17546 0.61817 0.26868<br>H 0.20150 -2.44788 -0.18400<br>O -1.14101 1.88369 -0.27642<br>O 1.37605 2.62687 0.09097<br>C 2.89648 -2.09246 0.16093<br>H 3.66000 -1.96009 -0.61234<br>H 3.39929 -2.02948 1.13128<br>H 2.48228 -3.09836 0.05862<br>S -2.21326 -0.93712 -0.51254<br>C -3.00864 -0.36902 1.02331<br>H -4.06585 -0.62917 0.94329<br>H -2.57485 -0.87523 1.88716<br>H -2.90753 0.71184 1.12711<br>H 2.31269 2.82140 0.20040<br>H -0.71223 2.74705 -0.20620	C_1	-859.38465 (2.1)	-859.20499 (2.0)	-859.25529 (2.3)	-859.52646 (1.4)	-859.39710 (1.6)
5-adduct, conf4	C -0.18993 0.91887 -0.16753<br>C 1.15120 1.28139 0.02603<br>C 2.14059 0.31654 0.13171<br>C 1.81699 -1.04435 0.05271<br>C 0.48174 -1.39986 -0.12054<br>C -0.52754 -0.43291 -0.23296<br>H 3.17349 0.62268 0.27945<br>H 0.20421 -2.44865 -0.16811<br>O -1.14282 1.88102 -0.28346<br>O 1.37255 2.62847 0.09044<br>C 2.90058 -2.08942 0.15093<br>H 2.47478 -3.08454 0.30078<br>H 3.50178 -2.11613 -0.76429<br>H 3.57950 -1.87874 0.98248<br>S -2.21103 -0.94212 -0.51054<br>C -3.01167 -0.36521 1.01916<br>H -4.06855 -0.62595 0.93677<br>H -2.58101 -0.86652 1.88741<br>H -2.91096 0.71620 1.11715<br>H 2.30897 2.82414 0.19965<br>H -0.71539 2.74523 -0.21568	C_1	-859.38460 (2.1)	-859.20505 (2.0)	-859.25625 (1.6)	-859.52642 (1.5)	-859.39807 (1.0)

5-adduct, conf5	C -1.14655 -0.15291 0.00000<br>C -1.02711 -1.54533 0.00000<br>C 0.22107 -2.15238 0.00000<br>C 1.37940 -1.36764 0.00000<br>C 1.25687 0.02195 0.00000<br>C 0.00000 0.64144 0.00000<br>H 0.29331 -3.23722 0.00000<br>H 2.15845 0.62383 0.00000<br>O -2.37534 0.43847 0.00000<br>O -2.21617 -2.21915 0.00000<br>C 2.73742 -2.02598 0.00000<br>H 2.86638 -2.66109 0.88215<br>H 2.86638 -2.66109 -0.88215<br>H 3.53730 -1.28168 0.00000<br>S -0.26162 2.39813 0.00000<br>C 1.42372 3.05686 0.00000<br>H 1.31036 4.14229 0.00000<br>H 1.97104 2.75934 0.89648<br>H 1.97104 2.75934 -0.89648<br>H -2.07506 -3.17144 0.00000<br>H -3.05008 -0.25295 0.00000	C_s	-859.38535 (1.6)	saddle point			
5-adduct, conf6	C 1.14726 0.16903 0.00000<br>C 1.02995 1.56314 0.00000<br>C -0.21984 2.16563 0.00000<br>C -1.37785 1.38232 0.00000<br>C -1.25694 -0.00735 0.00000<br>C 0.00000 -0.62663 0.00000<br>H -0.28022 3.24988 0.00000<br>H -2.15759 -0.61063 0.00000<br>O 2.43435 -0.29992 0.00000<br>O 2.15029 2.33646 0.00000<br>C -2.73594 2.04025 0.00000<br>H -2.86397 2.67570 0.88197<br>H -2.86397 2.67570 -0.88197<br>H -3.53653 1.29671 0.00000<br>S 0.24751 -2.38735 0.00000<br>C -1.43236 -3.05678 0.00000<br>H -1.31101 -4.14087 0.00000<br>H -1.97593 -2.75848 0.89779<br>H -1.97593 -2.75848 -0.89779<br>H 2.92612 1.76040 0.00000<br>H 2.47605 -1.26242 0.00000	C_s	-859.38303 (3.1)	saddle point			

5-adduct, conf7	C -1.14868 0.17494 0.00000<br>C -1.02881 1.56553 0.00000<br>C 0.22507 2.16710 0.00000<br>C 1.37898 1.38350 0.00000<br>C 1.25516 -0.00909 0.00000<br>C 0.00000 -0.62430 0.00000<br>H 0.28509 3.25114 0.00000<br>H 2.15648 -0.61205 0.00000<br>O -2.43599 -0.29439 0.00000<br>O -2.14564 2.34435 0.00000<br>C 2.74799 2.01910 0.00000<br>H 3.32146 1.71714 0.88214<br>H 3.32146 1.71714 -0.88214<br>H 2.67693 3.10939 0.00000<br>S -0.25395 -2.38397 0.00000<br>C 1.42373 -3.05835 0.00000<br>H 1.29956 -4.14212 0.00000<br>H 1.96800 -2.76126 -0.89779<br>H 1.96800 -2.76126 0.89779<br>H -2.92410 1.77191 0.00000<br>H -2.47748 -1.25687 0.00000	C_s	-859.38290 (3.2)	saddle point			
5-adduct, conf8	C -1.14778 0.15876 0.00000<br>C -1.02641 1.54769 0.00000<br>C 0.22593 2.15386 0.00000<br>C 1.38019 1.36892 0.00000<br>C 1.25502 -0.02360 0.00000<br>C 0.00000 -0.63915 0.00000<br>H 0.29782 3.23848 0.00000<br>H 2.15732 -0.62510 0.00000<br>O -2.37653 -0.43326 0.00000<br>O -2.21260 2.22700 0.00000<br>C 2.74898 2.00506 0.00000<br>H 3.32260 1.70342 0.88216<br>H 3.32260 1.70342 -0.88216<br>H 2.67836 3.09543 0.00000<br>S -0.26780 -2.39490 0.00000<br>C 1.41570 -3.05795 0.00000<br>H 1.30018 -4.14310 0.00000<br>H 1.96349 -2.76129 -0.89647<br>H 1.96349 -2.76129 0.89647<br>H -2.06699 3.17856 0.00000<br>H -3.05088 0.25846 0.00000	C_s	-859.38522 (1.7)	saddle point			

Table S12.3. Reduced anionic form in water.

Structure	Schematic drawing	Symmetry	G_{PCM}	$H_{PCM,RRHO}$	$G_{PCM,RRHO}$	$G_{SMD,\omega B97X\text{-}D,large}$	$G_{SMD,RRHO,\omega B97X\text{-}D,large}$
6-adduct, 3-phenolate, conf1	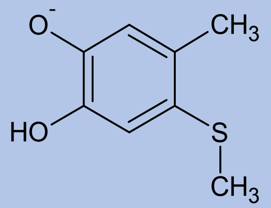<br>2-hydroxy-5-methyl-4-(methylsulfanyl)phenolate<br><br>C -1.63827 -0.85810 -0.06609<br>C -2.07788 0.49154 0.12604<br>C -1.06767 1.47717 0.09043<br>C 0.28748 1.17588 -0.11737<br>C 0.66700 -0.16407 -0.29400<br>C -0.31632 -1.17893 -0.26877<br>H -1.36533 2.51492 0.23050<br>H -0.02861 -2.21772 -0.40897<br>O -2.63056 -1.80156 -0.03420<br>O -3.34491 0.69278 0.31072<br>C 1.29460 2.30281 -0.14094<br>H 0.79929 3.26398 0.02012<br>H 2.05703 2.18490 0.63647<br>H 1.82277 2.34951 -1.09866<br>S 2.36529 -0.63414 -0.56328<br>C 2.97738 -0.79928 1.14552<br>H 4.03085 -1.08374 1.09125<br>H 2.89085 0.14945 1.67958<br>H 2.42140 -1.57389 1.67769<br>H -3.42707 -1.25311 0.12342	C_1	-858.91093 (0.4)	-858.74515 (0.2)	-858.79429 (0.2)	-859.05404 (0.0)	-858.93741 (0.0)
6-adduct, 3-phenolate, conf2	C -0.74044 -1.56865 0.00000<br>C 0.59898 -2.06751 0.00000<br>C 1.60342 -1.08451 0.00000<br>C 1.33072 0.29680 0.00000<br>C 0.00000 0.72986 0.00000<br>C -1.04032 -0.22369 0.00000<br>H 2.64276 -1.41098 0.00000<br>H -2.08428 0.07037 0.00000	C_s	-858.90552 (3.8)	saddle point			

	O -1.71420 -2.53088 0.00000<br>O 0.76332 -3.35973 0.00000<br>C 2.50939 1.24712 0.00000<br>H 3.13859 1.08535 0.88179<br>H 3.13859 1.08535 -0.88179<br>H 2.21198 2.29809 0.00000<br>S -0.33917 2.49535 0.00000<br>C -2.14561 2.58833 0.00000<br>H -2.38362 3.65366 0.00000<br>H -2.57397 2.13209 -0.89494<br>H -2.57397 2.13209 0.89494<br>H -1.17922 -3.35332 0.00000						
6-adduct, 3-phenolate, conf3	C 0.75949 -1.56876 0.00000<br>C -0.57369 -2.07828 0.00000<br>C -1.59107 -1.10479 0.00000<br>C -1.32950 0.27642 0.00000<br>C 0.00000 0.72014 0.00000<br>C 1.04889 -0.21961 0.00000<br>H -2.62541 -1.44512 0.00000<br>H 2.08990 0.08570 0.00000<br>O 1.74310 -2.52217 0.00000<br>O -0.72928 -3.37204 0.00000<br>C -2.48187 1.25326 0.00000<br>H -2.46170 1.90636 0.88107<br>H -2.46170 1.90636 -0.88107<br>H -3.43823 0.72434 0.00000<br>S 0.29009 2.48914 0.00000<br>C 2.09386 2.62822 0.00000<br>H 2.30837 3.69842 0.00000<br>H 2.53180 2.18077 0.89474<br>H 2.53180 2.18077 -0.89474<br>H 1.21652 -3.34971 0.00000	C_s	-858.90772 (2.4)	saddle point			
6-adduct, 4-phenolate, conf1	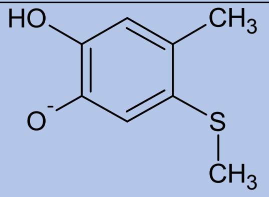<br>2-hydroxy-4-methyl-5-(methylsulfanyl)phenolate<br><br>C -1.67250 -0.93700 -0.06768<br>C -2.00680 0.44228 0.10846	C_1	-858.90924 (1.5)	-858.74338 (1.3)	-858.79233 (1.4)	-859.05267 (0.9)	-858.93576 (1.0)

	C -1.06538 1.45086 0.08338<br>C 0.30016 1.16690 -0.12306<br>C 0.65779 -0.17669 -0.29175<br>C -0.30579 -1.20350 -0.26838<br>H -1.38837 2.48036 0.22291<br>H 0.00991 -2.23489 -0.40572<br>O -2.64297 -1.80366 -0.02636<br>O -3.33644 0.68580 0.30228<br>C 1.29992 2.29887 -0.15351<br>H 0.79613 3.25653 0.00509<br>H 2.06600 2.19697 0.62313<br>H 1.82593 2.35157 -1.11256<br>S 2.36796 -0.64223 -0.55960<br>C 2.96687 -0.75352 1.15666<br>H 4.02300 -1.02950 1.11780<br>H 2.86788 0.20816 1.66453<br>H 2.41362 -1.51874 1.70476<br>H -3.71167 -0.22097 0.26159						
6-adduct, 4-phenolate, conf2	C -0.84029 -1.59297 0.00000<br>C 0.52873 -2.00487 0.00000<br>C 1.56721 -1.10252 0.00000<br>C 1.33145 0.28925 0.00000<br>C 0.00000 0.72026 0.00000<br>C -1.06351 -0.20236 0.00000<br>H 2.59222 -1.46904 0.00000<br>H -2.09535 0.13217 0.00000<br>O -1.75175 -2.52134 0.00000<br>O 0.72387 -3.36065 0.00000<br>C 2.52637 1.21680 0.00000<br>H 3.15518 1.04969 0.88190<br>H 3.15518 1.04969 -0.88190<br>H 2.24458 2.27220 0.00000<br>S -0.30446 2.49269 0.00000<br>C -2.10789 2.63432 0.00000<br>H -2.31557 3.70603 0.00000<br>H -2.54788 2.18955 -0.89474<br>H -2.54788 2.18955 0.89474<br>H -0.19846 -3.69439 0.00000	C_s	-858.90626 (3.3)	saddle point			
6-adduct, 4-phenolate, conf3	C 0.86058 -1.59136 0.00000<br>C -0.50277 -2.01492 0.00000<br>C -1.55375 -1.12328 0.00000<br>C -1.32993 0.26803 0.00000<br>C 0.00000 0.71083 0.00000	C_s	-858.90846 (2.0)	-858.74242 (1.9)	-858.79127 (2.0)	-859.05325 (0.5)	-858.93607 (0.8)

	C 1.07241 -0.19698 0.00000<br>H -2.57347 -1.50307 0.00000<br>H 2.10068 0.14936 0.00000<br>O 1.78244 -2.50999 0.00000<br>O -0.68688 -3.37337 0.00000<br>C -2.49917 1.22285 0.00000<br>H -2.49764 1.87712 0.88104<br>H -2.49764 1.87712 -0.88104<br>H -3.44446 0.67351 0.00000<br>S 0.25398 2.48574 0.00000<br>C 2.05328 2.67389 0.00000<br>H 2.23732 3.74981 0.00000<br>H 2.50271 2.23825 0.89457<br>H 2.50271 2.23825 -0.89457<br>H 0.23783 -3.69966 0.00000						
5-adduct, 3-phenolate, conf1	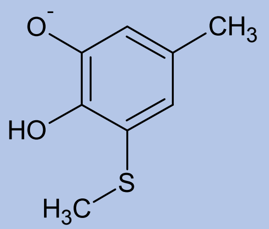<br>2-hydroxy-5-methyl-3-(methylsulfanyl)phenolate<br><br>C -0.19300 0.92674 -0.16406<br>C 1.15048 1.39719 0.03692<br>C 2.15062 0.41339 0.13429<br>C 1.85681 -0.95953 0.04529<br>C 0.54093 -1.37101 -0.13720<br>C -0.49933 -0.41901 -0.24438<br>H 3.18005 0.73633 0.28264<br>H 0.29823 -2.42840 -0.19636<br>O -1.12246 1.91926 -0.26151<br>O 1.31382 2.68535 0.09995<br>C 2.97207 -1.97327 0.16438<br>H 3.73195 -1.82243 -0.61057<br>H 3.47909 -1.89364 1.13240<br>H 2.59184 -2.99424 0.06689<br>S -2.17374 -0.96904 -0.52204<br>C -2.98037 -0.44768 1.02416<br>H -4.03463 -0.72149 0.94568	C_1	-858.91056 (0.6)	-858.74440 (0.7)	-858.79286 (1.1)	-859.05309 (0.6)	-858.93539 (1.3)

	H -2.53573 -0.96008 1.87945<br>H -2.89430 0.63270 1.14834<br>H -0.55690 2.71811 -0.15975						
5-adduct, 3-phenolate, conf2	C -0.07996 1.05935 -0.12777<br>C 1.31414 1.35462 0.10107<br>C 2.16112 0.21773 0.14862<br>C 1.71517 -1.10120 -0.01662<br>C 0.36362 -1.33690 -0.24081<br>C -0.52920 -0.24741 -0.28851<br>H 3.22051 0.40324 0.32201<br>H -0.01575 -2.34559 -0.37768<br>O -0.93579 2.12892 -0.16898<br>O 1.73265 2.56435 0.24994<br>C 2.70229 -2.24432 0.04885<br>H 3.47900 -2.14348 -0.71728<br>H 3.20928 -2.27597 1.01954<br>H 2.20612 -3.20751 -0.10140<br>S -2.27203 -0.52895 -0.57820<br>C -2.86559 -0.81383 1.12164<br>H -3.94380 -0.97600 1.06415<br>H -2.38777 -1.69936 1.54380<br>H -2.66318 0.05498 1.75023<br>H -1.81643 1.77850 -0.37868	C_1	-858.90275 (5.5)	-858.73675 (5.5)	-858.78625 (5.2)	-859.04986 (2.6)	-858.93336 (2.5)
5-adduct, 3-phenolate, conf3	C 1.15588 0.14318 0.00000<br>C 1.14729 1.57432 0.00000<br>C -0.11944 2.19143 0.00000<br>C -1.30376 1.43802 0.00000<br>C -1.24889 0.04462 0.00000<br>C 0.00000 -0.61399 0.00000<br>H -0.16895 3.27914 0.00000<br>H -2.17327 -0.52226 0.00000<br>O 2.40256 -0.42392 0.00000<br>O 2.30387 2.16820 0.00000<br>C -2.63995 2.14671 0.00000<br>H -2.74920 2.78840 0.88122<br>H -2.74920 2.78840 -0.88122<br>H -3.46892 1.43319 0.00000<br>S 0.20290 -2.38695 0.00000<br>C -1.50093 -2.99923 0.00000<br>H -1.42371 -4.08798 0.00000<br>H -2.04073 -2.68315 0.89482<br>H -2.04073 -2.68315 -0.89482<br>H 2.97567 0.37399 0.00000	C_s	-858.91004 (1.0)	saddle point			

5-adduct, 3-phenolate, conf4	C -1.15755 0.14829 0.00000<br>C -1.14668 1.57554 0.00000<br>C 0.12420 2.19172 0.00000<br>C 1.30535 1.43891 0.00000<br>C 1.24765 0.04265 0.00000<br>C 0.00000 -0.61123 0.00000<br>H 0.17239 3.27915 0.00000<br>H 2.17222 -0.52460 0.00000<br>O -2.40433 -0.42166 0.00000<br>O -2.29955 2.17717 0.00000<br>C 2.65172 2.13013 0.00000<br>H 3.24135 1.85584 0.88166<br>H 3.24135 1.85584 -0.88166<br>H 2.53710 3.21772 0.00000<br>S -0.20880 -2.38340 0.00000<br>C 1.49309 -3.00050 0.00000<br>H 1.41304 -4.08906 0.00000<br>H 2.03366 -2.68563 -0.89482<br>H 2.03366 -2.68563 0.89482<br>H -2.97973 0.37365 0.00000	C_s	-858.90964 (1.2)	saddle point			
5-adduct, 3-phenolate, conf5	C 1.19480 0.12512 0.00000<br>C 1.22018 1.56396 0.00000<br>C -0.05551 2.18852 0.00000<br>C -1.25878 1.47912 0.00000<br>C -1.23695 0.08506 0.00000<br>C 0.00000 -0.58773 0.00000<br>H -0.07180 3.27766 0.00000<br>H -2.17053 -0.46575 0.00000<br>O 2.43080 -0.49130 0.00000<br>O 2.32970 2.21928 0.00000<br>C -2.57521 2.22318 0.00000<br>H -2.66674 2.86755 0.88120<br>H -2.66674 2.86755 -0.88120<br>H -3.42332 1.53234 0.00000<br>S 0.13156 -2.37322 0.00000<br>C -1.58386 -2.94631 0.00000<br>H -1.52809 -4.03598 0.00000<br>H -2.11146 -2.61591 0.89619<br>H -2.11146 -2.61591 -0.89619<br>H 2.33312 -1.44953 0.00000	C_s	-858.89664 (9.4)	saddle point			
5-adduct, 3-phenolate, conf6	C -1.19670 0.12972 0.00000<br>C -1.21959 1.56479 0.00000<br>C 0.05983 2.18916 0.00000	C_s	-858.89643 (9.5)	saddle point			

	C 1.26012 1.48025 0.00000<br>C 1.23572 0.08322 0.00000<br>C 0.00000 -0.58526 0.00000<br>H 0.07471 3.27796 0.00000<br>H 2.16944 -0.46795 0.00000<br>O -2.43360 -0.48746 0.00000<br>O -2.32550 2.22678 0.00000<br>C 2.58623 2.20776 0.00000<br>H 3.18290 1.94844 0.88153<br>H 3.18290 1.94844 -0.88153<br>H 2.44250 3.29184 0.00000<br>S -0.13676 -2.37026 0.00000<br>C 1.57695 -2.94782 0.00000<br>H 1.51860 -4.03736 0.00000<br>H 2.10526 -2.61849 -0.89619<br>H 2.10526 -2.61849 0.89619<br>H -2.33594 -1.44559 0.00000						
5-adduct, 4-phenolate, conf1	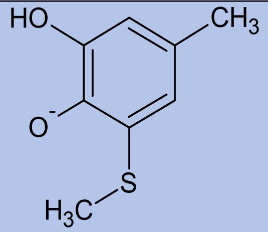<br>2-hydroxy-4-methyl-6-(methylsulfanyl)phenolate<br><br>C -0.22812 0.99043 -0.16887<br>C 1.16132 1.30222 0.02993<br>C 2.14776 0.34782 0.12887<br>C 1.81851 -1.02383 0.04468<br>C 0.48513 -1.36326 -0.13521<br>C -0.53154 -0.38906 -0.24622<br>H 3.18073 0.65773 0.27490<br>H 0.20418 -2.41282 -0.19387<br>O -1.05010 1.98863 -0.24918<br>O 1.42618 2.64084 0.10044<br>C 2.89723 -2.07393 0.16585<br>H 3.66321 -1.95816 -0.60992<br>H 3.40902 -2.01614 1.13358<br>H 2.47740 -3.07971 0.07066<br>S -2.20830 -0.91528 -0.52995	C_1	-858.91159 (0.0)	-858.74551 (0.0)	-858.79454 (0.0)	-859.05352 (0.3)	-858.93647 (0.6)

	C -3.01405 -0.40541 1.02079<br>H -4.08104 -0.61871 0.92390<br>H -2.60797 -0.96396 1.86681<br>H -2.86734 0.66515 1.17266<br>H 0.52852 3.02548 -0.00856						
5-adduct, 4-phenolate, conf2	C 0.84932 0.45948 0.00000<br>C 0.17666 1.72948 0.00000<br>C -1.19255 1.88191 0.00000<br>C -2.01638 0.73757 0.00000<br>C -1.40381 -0.50828 0.00000<br>C 0.00000 -0.66661 0.00000<br>H -1.62691 2.87929 0.00000<br>H -2.02901 -1.40057 0.00000<br>O 2.15095 0.49942 0.00000<br>O 1.03316 2.79315 0.00000<br>C -3.52000 0.88013 0.00000<br>H -3.87249 1.42829 0.88138<br>H -3.87249 1.42829 -0.88138<br>H -4.00951 -0.09821 0.00000<br>S 0.53061 -2.38024 0.00000<br>C 2.34581 -2.42091 0.00000<br>H 2.59329 -3.48572 0.00000<br>H 2.75827 -1.94664 -0.88808<br>H 2.75827 -1.94664 0.88808<br>H 1.90369 2.32855 0.00000	C_s	-858.90294 (5.4)	saddle point			
5-adduct, 4-phenolate, conf3	C -0.84845 0.46566 0.00000<br>C -0.17314 1.72936 0.00000<br>C 1.19937 1.87778 0.00000<br>C 2.01896 0.73428 0.00000<br>C 1.40100 -0.51207 0.00000<br>C 0.00000 -0.66520 0.00000<br>H 1.63461 2.87453 0.00000<br>H 2.02459 -1.40586 0.00000<br>O -2.15022 0.50427 0.00000<br>O -1.02387 2.80006 0.00000<br>C 3.52628 0.85542 0.00000<br>H 3.97107 0.38059 0.88169<br>H 3.97107 0.38059 -0.88169<br>H 3.83485 1.90505 0.00000<br>S -0.53919 -2.37615 0.00000<br>C -2.35456 -2.41024 0.00000<br>H -2.60528 -3.47427 0.00000<br>H -2.76533 -1.93468 0.88809	C_s	-858.90231 (5.8)	saddle point			

	H -2.76533 -1.93468 -0.88809<br>H -1.89731 2.34251 0.00000						
5-adduct, 4-phenolate, conf4	C 1.22103 0.08351 0.00000<br>C 1.11185 1.50516 0.00000<br>C -0.09700 2.17331 0.00000<br>C -1.29966 1.44290 0.00000<br>C -1.23227 0.05021 0.00000<br>C 0.00000 -0.62601 0.00000<br>H -0.11010 3.26127 0.00000<br>H -2.16233 -0.51057 0.00000<br>O 2.41170 -0.44409 0.00000<br>O 2.31164 2.16391 0.00000<br>C -2.62791 2.16307 0.00000<br>H -2.73867 2.80535 0.88129<br>H -2.73867 2.80535 -0.88129<br>H -3.46048 1.45346 0.00000<br>S 0.17861 -2.40191 0.00000<br>C -1.53571 -2.98631 0.00000<br>H -1.48207 -4.07662 0.00000<br>H -2.07256 -2.66065 0.89391<br>H -2.07256 -2.66065 -0.89391<br>H 2.95081 1.42004 0.00000	C_s	-858.91098 (0.4)	saddle point			
5-adduct, 4-phenolate, conf5	C -1.22253 0.08921 0.00000<br>C -1.11031 1.50676 0.00000<br>C 0.10277 2.17328 0.00000<br>C 1.30174 1.44280 0.00000<br>C 1.23132 0.04738 0.00000<br>C 0.00000 -0.62401 0.00000<br>H 0.11636 3.26105 0.00000<br>H 2.16118 -0.51435 0.00000<br>O -2.41258 -0.44075 0.00000<br>O -2.30747 2.17272 0.00000<br>C 2.64057 2.14554 0.00000<br>H 3.23611 1.88270 0.88175<br>H 3.23611 1.88270 -0.88175<br>H 2.51292 3.23206 0.00000<br>S -0.18565 -2.39865 0.00000<br>C 1.52658 -2.98873 0.00000<br>H 1.46942 -4.07886 0.00000<br>H 2.06436 -2.66461 -0.89393<br>H 2.06436 -2.66461 0.89393<br>H -2.95082 1.43313 0.00000	C_s	-858.91056 (0.6)	saddle point			

Table S12.4. One-electron oxidized neutral form in vacuo.

Structure	Schematic drawing	Symmetry	*E*	H_{RRHO}	G_{RRHO}	$E_{\omega B97X\text{-}D,large}$	$G_{RRHO,\omega B97X\text{-}D,large}$
6-adduct, 3-phenoxyl, conf1	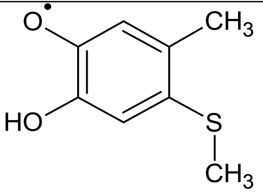 [2-hydroxy-5-methyl-4-(methylsulfanyl)phenyl]oxidanyl<br><br>C -0.83200 -1.54328 0.00000<br>C 0.52594 -2.07716 0.00000<br>C 1.59036 -1.11446 0.00000<br>C 1.35977 0.23726 0.00000<br>C 0.00000 0.71340 0.00000<br>C -1.07673 -0.18632 0.00000<br>H 2.60508 -1.50167 0.00000<br>H -2.10615 0.14777 0.00000<br>O -1.81476 -2.44192 0.00000<br>O 0.66747 -3.32087 0.00000<br>C 2.54589 1.17538 0.00000<br>H 3.16572 0.99875 0.88367<br>H 3.16572 0.99875 -0.88367<br>H 2.26727 2.22969 0.00000<br>S -0.23977 2.45393 0.00000<br>C -2.03623 2.64759 0.00000<br>H -2.20984 3.72501 0.00000<br>H -2.48607 2.21798 -0.89735<br>H -2.48607 2.21798 0.89735<br>H -1.36309 -3.30927 0.00000	C_s	-858.74451<br>(1.9)	saddle point			
6-adduct, 3-phenoxyl, conf2	C 0.84573 -1.54380 0.00000<br>C -0.50820 -2.08604 0.00000<br>C -1.58318 -1.13189 0.00000<br>C -1.35868 0.21940 0.00000<br>C 0.00000 0.70405 0.00000<br>C 1.08349 -0.18410 0.00000<br>H -2.59326 -1.52939 0.00000<br>H 2.11050 0.15810 0.00000<br>O 1.83488 -2.43569 0.00000	C_s	-858.74754<br>(0.0)	-858.58035<br>(0.1)	-858.62981<br>(0.4)	-858.88786<br>(0.0)	-858.77012<br>(0.3)

	O -0.64192 -3.33067 0.00000<br>C -2.51358 1.18644 0.00000<br>H -2.49218 1.83496 0.88260<br>H -2.49218 1.83496 -0.88260<br>H -3.46240 0.64673 0.00000<br>S 0.20017 2.44761 0.00000<br>C 1.99228 2.67879 0.00000<br>H 2.14656 3.75911 0.00000<br>H 2.44979 2.25700 0.89718<br>H 2.44979 2.25700 -0.89718<br>H 1.38979 -3.30635 0.00000						
6-adduct, 4-phenoxyl, conf1	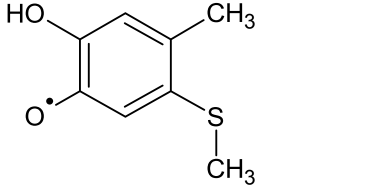<br>[2-hydroxy-4-methyl-5-(methylsulfanyl)phenyl]oxidanyl<br><br>C -1.65940 -0.95998 -0.06768<br>C -2.04050 0.43581 0.11500<br>C -1.09811 1.45085 0.08408<br>C 0.25057 1.15781 -0.12157<br>C 0.66202 -0.21125 -0.28396<br>C -0.25939 -1.22798 -0.26857<br>H -1.42478 2.47757 0.21327<br>H 0.04013 -2.26143 -0.40529<br>O -2.56130 -1.82812 -0.03316<br>O -3.33321 0.67497 0.30501<br>C 1.24651 2.27963 -0.18875<br>H 1.77065 2.27477 -1.14899<br>H 0.75354 3.24532 -0.06091<br>H 2.01428 2.18164 0.58524<br>S 2.38330 -0.62087 -0.55247<br>C 2.95786 -0.67306 1.17449<br>H 4.02414 -0.90703 1.14537<br>H 2.82176 0.29026 1.67090<br>H 2.43353 -1.45268 1.73012<br>H -3.76737 -0.20012 0.27682	C_1	-858.74220 (3.4)	-858.57503 (3.5)	-858.62434 (3.8)	-858.88152 (4.0)	-858.76366 (4.4)
6-adduct, 4-phenoxyl, conf2	C -0.95395 -1.52857 0.00000<br>C 0.40731 -2.05791 0.00000<br>C 1.50367 -1.21369 0.00000	C_s	-858.74293 (2.9)	saddle point			

	C 1.33417 0.16953 0.00000<br>C 0.00000 0.72416 0.00000<br>C -1.09835 -0.10069 0.00000<br>H 2.50257 -1.63902 0.00000<br>H -2.11403 0.27356 0.00000<br>O -1.89738 -2.35031 0.00000<br>O 0.52169 -3.38162 0.00000<br>C 2.57053 1.03025 0.00000<br>H 3.17995 0.81147 0.88272<br>H 3.17995 0.81147 -0.88272<br>H 2.35687 2.09901 0.00000<br>S -0.12658 2.49228 0.00000<br>C -1.91015 2.77078 0.00000<br>H -2.03729 3.85470 0.00000<br>H -2.37923 2.35986 -0.89672<br>H -2.37923 2.35986 0.89672<br>H -0.39822 -3.71522 0.00000						
6-adduct, 4-phenoxyl, conf3	C 0.96643 -1.52945 0.00000<br>C -0.39104 -2.06519 0.00000<br>C -1.49750 -1.22790 0.00000<br>C -1.33386 0.15238 0.00000<br>C 0.00000 0.71432 0.00000<br>C 1.10425 -0.09980 0.00000<br>H -2.49143 -1.66320 0.00000<br>H 2.11764 0.28125 0.00000<br>O 1.91426 -2.34666 0.00000<br>O -0.49931 -3.38896 0.00000<br>C -2.53943 1.04815 0.00000<br>H -2.54873 1.69973 0.88112<br>H -2.54873 1.69973 -0.88112<br>H -3.45967 0.46111 0.00000<br>S 0.09092 2.48259 0.00000<br>C 1.86898 2.79640 0.00000<br>H 1.97712 3.88240 0.00000<br>H 2.34503 2.39337 0.89661<br>H 2.34503 2.39337 -0.89661<br>H 0.42245 -3.71782 0.00000	C_s	-858.74553 (1.3)	-858.57836 (1.4)	-858.62732 (2.0)	-858.88557 (1.4)	-858.76736 (2.1)

5-adduct, 3-phenoxyl, conf1	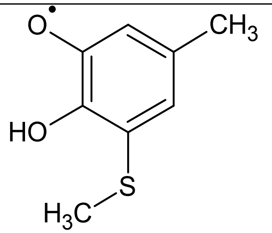<br>[2-hydroxy-5-methyl-3-(methylsulfanyl)phenyl]oxidanyl<br><br>C -0.21945 0.92761 -0.15528<br>C 1.15170 1.41239 0.02629<br>C 2.19946 0.43199 0.12780<br>C 1.91286 -0.90502 0.06153<br>C 0.55490 -1.32615 -0.09072<br>C -0.51113 -0.43859 -0.20285<br>H 3.21318 0.79806 0.25488<br>H 0.34083 -2.39112 -0.11977<br>O -1.14847 1.86733 -0.26685<br>O 1.31547 2.65202 0.06695<br>C 2.98622 -1.95701 0.15745<br>H 3.96741 -1.50771 0.32443<br>H 2.78151 -2.65026 0.97962<br>H 3.03360 -2.54665 -0.76415<br>S -2.15546 -1.05176 -0.47948<br>C -3.04286 -0.33313 0.93680<br>H -4.06329 -0.71776 0.88176<br>H -2.59046 -0.65216 1.87745<br>H -3.06198 0.75495 0.87101<br>H -0.65943 2.71353 -0.20042	C_1	-858.74186 (3.6)	-858.57476 (3.6)	-858.62519 (3.3)	-858.88107 (4.3)	-858.76440 (3.9)
5-adduct, 3-phenoxyl, conf2	C -0.12517 1.06561 -0.14129<br>C 1.29065 1.36987 0.10576<br>C 2.19127 0.23900 0.15672<br>C 1.76775 -1.05732 -0.01095<br>C 0.38891 -1.29520 -0.23596<br>C -0.54597 -0.25887 -0.29043<br>H 3.23694 0.47306 0.33459<br>H 0.03775 -2.31393 -0.37473<br>O -0.95593 2.10046 -0.20374<br>O 1.67664 2.54196 0.25885<br>C 2.73997 -2.21093 0.04149<br>H 3.50981 -2.10812 -0.72913	C_1	-858.73236 (9.5)	-858.56555 (9.4)	-858.61623 (8.9)	-858.87203 (9.9)	-858.75590 (9.2)

	H 3.24583 -2.25195 1.01076<br>H 2.23522 -3.16756 -0.11448<br>S -2.27683 -0.58118 -0.57451<br>C -2.84623 -0.79999 1.14443<br>H -3.92582 -0.95781 1.10331<br>H -2.37111 -1.67357 1.59352<br>H -2.63396 0.08799 1.74245<br>H -1.83817 1.75843 -0.43364						
5-adduct, 3-phenoxyl, conf3	C -0.12260 1.06507 -0.14361<br>C 1.29108 1.37305 0.10571<br>C 2.19530 0.24197 0.16072<br>C 1.77382 -1.05143 -0.00565<br>C 0.39145 -1.29505 -0.23234<br>C -0.54265 -0.26302 -0.29021<br>H 3.23971 0.47876 0.33990<br>H 0.04573 -2.31639 -0.36808<br>O -0.95662 2.09686 -0.21115<br>O 1.67439 2.54539 0.25687<br>C 2.72623 -2.21862 0.04025<br>H 2.43177 -2.93549 0.81345<br>H 2.73555 -2.75144 -0.91626<br>H 3.74528 -1.88864 0.25323<br>S -2.27386 -0.58664 -0.57391<br>C -2.84491 -0.78560 1.14692<br>H -3.92454 -0.94333 1.10654<br>H -2.37050 -1.65433 1.60603<br>H -2.63291 0.10898 1.73516<br>H -1.83697 1.75180 -0.44398	C_1	-858.73311 (9.1)	-858.56634 (8.9)	-858.61664 (8.7)	-858.87288 (9.4)	-858.75641 (8.9)
5-adduct, 3-phenoxyl, conf4	C 1.18085 0.11240 0.00000<br>C 1.16758 1.57462 0.00000<br>C -0.11160 2.22705 0.00000<br>C -1.26433 1.48774 0.00000<br>C -1.19865 0.05855 0.00000<br>C 0.00000 -0.64265 0.00000<br>H -0.12405 3.31228 0.00000<br>H -2.13821 -0.48293 0.00000<br>O 2.37551 -0.46182 0.00000<br>O 2.28005 2.14899 0.00000<br>C -2.61511 2.16164 0.00000<br>H -2.73230 2.79728 0.88272<br>H -2.73230 2.79728 -0.88272<br>H -3.42914 1.43278 0.00000<br>S 0.17798 -2.40359 0.00000	C_s	-858.73972 (4.9)	saddle point			

	C -1.54282 -2.95738 0.00000<br>H -1.49757 -4.04797 0.00000<br>H -2.07394 -2.63075 0.89730<br>H -2.07394 -2.63075 -0.89730<br>H 3.01376 0.28105 0.00000						
5-adduct, 3-phenoxyl, conf5	C -1.18061 0.12005 0.00000<br>C -1.16484 1.58106 0.00000<br>C 0.11781 2.23154 0.00000<br>C 1.26541 1.48896 0.00000<br>C 1.19840 0.05639 0.00000<br>C 0.00000 -0.64014 0.00000<br>H 0.13263 3.31632 0.00000<br>H 2.13914 -0.48406 0.00000<br>O -2.37603 -0.45226 0.00000<br>O -2.27535 2.15802 0.00000<br>C 2.62689 2.13295 0.00000<br>H 3.20041 1.83164 0.88303<br>H 3.20041 1.83164 -0.88303<br>H 2.54898 3.22212 0.00000<br>S -0.18633 -2.39991 0.00000<br>C 1.53220 -2.96020 0.00000<br>H 1.48320 -4.05061 0.00000<br>H 2.06436 -2.63514 -0.89727<br>H 2.06436 -2.63514 0.89727<br>H -3.01282 0.29205 0.00000	C_s	-858.74089 (4.2)	-858.57371 (4.3)	-858.62414 (4.0)	-858.88076 (4.5)	-858.76401 (4.2)
5-adduct, 3-phenoxyl, conf6	C 1.21545 0.08440 0.00000<br>C 1.23835 1.55020 0.00000<br>C -0.04271 2.22151 0.00000<br>C -1.22518 1.53059 0.00000<br>C -1.19236 0.10933 0.00000<br>C 0.00000 -0.61218 0.00000<br>H -0.01398 3.30695 0.00000<br>H -2.13961 -0.41914 0.00000<br>O 2.41477 -0.50535 0.00000<br>O 2.31786 2.16807 0.00000<br>C -2.55261 2.24977 0.00000<br>H -2.64996 2.88859 0.88278<br>H -2.64996 2.88859 -0.88278<br>H -3.39016 1.54747 0.00000<br>S 0.10228 -2.38832 0.00000<br>C -1.62540 -2.91394 0.00000<br>H -1.59790 -4.00471 0.00000<br>H -2.14394 -2.57364 0.89854	C_s	-858.72493 (14.2)	saddle point			

	H -2.14394 -2.57364 -0.89854<br>H 2.33862 -1.46720 0.00000						
5-adduct, 3-phenoxyl, conf7	C -1.21555 0.09289 0.00000<br>C -1.23477 1.55811 0.00000<br>C 0.05073 2.22620 0.00000<br>C 1.22691 1.53128 0.00000<br>C 1.19188 0.10550 0.00000<br>C 0.00000 -0.61016 0.00000<br>H 0.02539 3.31134 0.00000<br>H 2.13982 -0.42258 0.00000<br>O -2.41575 -0.49425 0.00000<br>O -2.31159 2.17870 0.00000<br>C 2.56655 2.22098 0.00000<br>H 3.15082 1.94234 0.88343<br>H 3.15082 1.94234 -0.88343<br>H 2.44959 3.30674 0.00000<br>S -0.11212 -2.38534 0.00000<br>C 1.61295 -2.91878 0.00000<br>H 1.58088 -4.00941 0.00000<br>H 2.13278 -2.58038 -0.89852<br>H 2.13278 -2.58038 0.89852<br>H -2.34227 -1.45634 0.00000	C_s	-858.72592<br>(13.6)	saddle point			
5-adduct, 4-phenoxyl, conf1	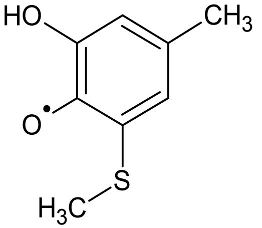<br>[2-hydroxy-4-methyl-6-(methylsulfanyl)phenyl]oxidanyl<br>C -0.25660 0.92833 -0.11508<br>C 1.14377 1.34596 0.00244<br>C 2.17583 0.43832 0.07292<br>C 1.88520 -0.93502 0.04904<br>C 0.54796 -1.37758 -0.03506<br>C -0.51977 -0.50038 -0.11030<br>H 3.19905 0.79234 0.14758<br>H 0.35021 -2.44553 -0.02643<br>O -1.11632 1.83058 -0.22186<br>O 1.34904 2.66200 0.01763<br>C 3.00657 -1.93123 0.15007	C_1	-858.74554<br>(1.3)	-858.57844<br>(1.3)	-858.62834<br>(1.3)	-858.88509<br>(1.7)	-858.76789<br>(1.7)

	H 3.77282 -1.73095 -0.60558<br>H 3.49174 -1.86449 1.13006<br>H 2.65052 -2.95478 0.01567<br>S -2.13322 -1.15965 -0.33876<br>C -3.20883 -0.07032 0.64351<br>H -4.17068 -0.58464 0.69419<br>H -2.81208 0.04571 1.65329<br>H -3.32007 0.90319 0.17147<br>H 0.46357 3.06452 -0.07139						
5-adduct, 4-phenoxyl, conf2	C -0.25274 0.93331 -0.12374<br>C 1.14555 1.34715 0.00577<br>C 2.17986 0.43556 0.08910<br>C 1.88729 -0.93109 0.06456<br>C 0.54302 -1.37125 -0.02727<br>C -0.51912 -0.49604 -0.11617<br>H 3.20148 0.79106 0.17286<br>H 0.34532 -2.43966 -0.01498<br>O -1.10941 1.83756 -0.23861<br>O 1.35623 2.66098 0.02051<br>C 2.98662 -1.95312 0.14101<br>H 2.77488 -2.69841 0.91407<br>H 3.07668 -2.48862 -0.81079<br>H 3.95091 -1.49049 0.36128<br>S -2.13800 -1.14551 -0.35635<br>C -3.19388 -0.08317 0.67643<br>H -4.16600 -0.57961 0.70369<br>H -2.79622 -0.02154 1.69077<br>H -3.28577 0.91236 0.24753<br>H 0.47255 3.06666 -0.07624	C_1	-858.74566 (1.2)	-858.57846 (1.3)	-858.62868 (1.1)	-858.88517 (1.7)	-858.76819 (1.5)
5-adduct, 4-phenoxyl, conf3	C -0.29942 -1.18483 -0.00188<br>C -1.71647 -0.83239 0.00204<br>C -2.13706 0.47754 0.00756<br>C -1.18098 1.50790 0.00785<br>C 0.19757 1.21284 0.00556<br>C 0.65934 -0.09330 0.00046<br>H -3.19870 0.70241 0.01128<br>H 0.89453 2.04331 0.00772<br>O 0.01178 -2.39562 -0.00585<br>O -2.56603 -1.85980 0.00121<br>C -1.64425 2.93882 -0.00897<br>H -2.11368 3.17649 -0.97011<br>H -2.39265 3.11578 0.76977<br>H -0.81716 3.63565 0.14452	C_1	-858.74751 (0.0)	-858.58052 (0.0)	-858.63013 (0.2)	-858.88767 (0.1)	-858.77029 (0.2)

	S 2.32831 -0.60918 -0.00220<br>C 3.21385 0.96715 0.00358<br>H 4.27502 0.71198 0.00127<br>H 2.99274 1.55575 -0.89011<br>H 2.99468 1.54772 0.90298<br>H -2.00910 -2.66132 -0.00222						
5-adduct, 4-phenoxyl, conf4	C -0.30868 -1.18451 -0.00283<br>C -1.72214 -0.82528 0.00243<br>C -2.13928 0.48991 0.01002<br>C -1.18101 1.51150 0.01130<br>C 0.20061 1.20759 0.00830<br>C 0.65618 -0.09671 0.00058<br>H -3.20015 0.71657 0.01518<br>H 0.89953 2.03701 0.01226<br>O -0.00353 -2.39708 -0.00778<br>O -2.57818 -1.84619 0.00149<br>C -1.60922 2.95316 -0.01213<br>H -1.01894 3.55192 0.68804<br>H -1.46092 3.37939 -1.01115<br>H -2.66515 3.06328 0.24428<br>S 2.32403 -0.62213 -0.00280<br>C 3.21535 0.95082 0.00437<br>H 4.27569 0.69215 0.00117<br>H 2.99591 1.54141 -0.88849<br>H 2.99893 1.53133 0.90455<br>H -2.02664 -2.65166 -0.00295	C_1	-858.74752 (0.0)	-858.58055 (0.0)	-858.63047 (0.0)	-858.88769 (0.1)	-858.77064 (0.0)
5-adduct, 4-phenoxyl, conf5	C 0.82818 0.47140 0.00000<br>C 0.13375 1.76151 0.00000<br>C -1.23433 1.85837 0.00000<br>C -2.00711 0.67932 0.00000<br>C -1.38529 -0.57549 0.00000<br>C 0.00000 -0.72092 0.00000<br>H -1.70676 2.83511 0.00000<br>H -2.00808 -1.46593 0.00000<br>O 2.08155 0.49778 0.00000<br>O 0.92735 2.83490 0.00000<br>C -3.50687 0.78507 0.00000<br>H -3.85493 1.33364 0.88153<br>H -3.85493 1.33364 -0.88153<br>H -3.98238 -0.19790 0.00000<br>S 0.60609 -2.36277 0.00000<br>C 2.41807 -2.29760 0.00000<br>H 2.71763 -3.34819 0.00000	C_s	-858.74493 (1.6)	saddle point			

	H 2.80174 -1.80146 -0.88931<br>H 2.80174 -1.80146 0.88931<br>H 1.83891 2.48550 0.00000						
5-adduct, 4-phenoxyl, conf6	C -0.82711 0.47707 0.00000<br>C -0.13145 1.76408 0.00000<br>C 1.24136 1.86161 0.00000<br>C 2.00791 0.68600 0.00000<br>C 1.38001 -0.57276 0.00000<br>C 0.00000 -0.71798 0.00000<br>H 1.71169 2.83887 0.00000<br>H 2.00506 -1.46236 0.00000<br>O -2.08065 0.50656 0.00000<br>O -0.92068 2.83909 0.00000<br>C 3.51047 0.75486 0.00000<br>H 3.92161 0.25237 0.88175<br>H 3.92161 0.25237 -0.88175<br>H 3.86567 1.78740 0.00000<br>S -0.61168 -2.36103 0.00000<br>C -2.42374 -2.28996 0.00000<br>H -2.72600 -3.33984 0.00000<br>H -2.80668 -1.79343 0.88942<br>H -2.80668 -1.79343 -0.88942<br>H -1.83347 2.49182 0.00000	C_s	-858.74469 (1.8)	saddle point			
5-adduct, 4-phenoxyl, conf7	C 1.21544 0.13035 0.00000<br>C 1.06518 1.58242 0.00000<br>C -0.17265 2.18232 0.00000<br>C -1.32740 1.38039 0.00000<br>C -1.22856 -0.02580 0.00000<br>C 0.00000 -0.66578 0.00000<br>H -0.24785 3.26494 0.00000<br>H -2.14884 -0.59918 0.00000<br>O 2.37028 -0.34854 0.00000<br>O 2.20167 2.27972 0.00000<br>C -2.67878 2.04070 0.00000<br>H -2.79662 2.67950 0.88163<br>H -2.79662 2.67950 -0.88163<br>H -3.48753 1.30672 0.00000<br>S 0.27629 -2.39076 0.00000<br>C -1.40933 -3.04514 0.00000<br>H -1.30496 -4.13150 0.00000<br>H -1.95716 -2.74504 0.89655<br>H -1.95716 -2.74504 -0.89655<br>H 2.91718 1.61599 0.00000	C_s	-858.74757 (0.0)	saddle point			

5-adduct, 4-phenoxyl, conf8	C -1.21697 0.13397 0.00000<br>C -1.06706 1.58378 0.00000<br>C 0.17399 2.18806 0.00000<br>C 1.32354 1.38913 0.00000<br>C 1.22361 -0.02294 0.00000<br>C 0.00000 -0.66270 0.00000<br>H 0.24503 3.27052 0.00000<br>H 2.14657 -0.59320 0.00000<br>O -2.37211 -0.34488 0.00000<br>O -2.20143 2.28255 0.00000<br>C 2.68930 2.01917 0.00000<br>H 3.25954 1.70887 0.88222<br>H 3.25954 1.70887 -0.88222<br>H 2.62814 3.10940 0.00000<br>S -0.27832 -2.38944 0.00000<br>C 1.40772 -3.04219 0.00000<br>H 1.30465 -4.12870 0.00000<br>H 1.95553 -2.74164 -0.89651<br>H 1.95553 -2.74164 0.89651<br>H -2.91800 1.61948 0.00000	C_s	-858.74756<br>(0.0)	saddle point			

Table S12.5. One-electron oxidized neutral form in water.

Structure	Schematic drawing	Symmetry	G_{PCM}	$H_{PCM,RRHO}$	$G_{PCM,RRHO}$	$G_{SMD,\omega B97X\text{-}D,large}$	$G_{SMD,RRHO,\omega B97X\text{-}D,large}$
6-adduct, 3-phenoxyl, conf1	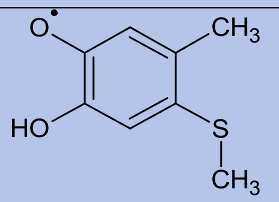<br>[2-hydroxy-5-methyl-4-(methylsulfanyl)phenyl]oxidanyl<br><br>C -0.82984 -1.54265 0.00000<br>C 0.52761 -2.07474 0.00000<br>C 1.59096 -1.11346 0.00000<br>C 1.36231 0.23847 0.00000<br>C 0.00000 0.71514 0.00000<br>C -1.07595 -0.18747 0.00000<br>H 2.60782 -1.49537 0.00000<br>H -2.10442 0.14992 0.00000	C_s	-858.75477<br>(2.2)	saddle point			

	O -1.82193 -2.43720 0.00000<br>O 0.67700 -3.32338 0.00000<br>C 2.54871 1.17439 0.00000<br>H 3.16806 0.99458 0.88299<br>H 3.16806 0.99458 -0.88299<br>H 2.27157 2.22873 0.00000<br>S -0.24300 2.44889 0.00000<br>C -2.03925 2.65130 0.00000<br>H -2.20409 3.72966 0.00000<br>H -2.48752 2.22371 -0.89824<br>H -2.48752 2.22371 0.89824<br>H -1.39180 -3.31311 0.00000						
6-adduct, 3-phenoxyl, conf2	C 0.84232 -1.54369 0.00000<br>C -0.51144 -2.08341 0.00000<br>C -1.58476 -1.12974 0.00000<br>C -1.36139 0.22167 0.00000<br>C 0.00000 0.70613 0.00000<br>C 1.08201 -0.18591 0.00000<br>H -2.59721 -1.52178 0.00000<br>H 2.10831 0.15868 0.00000<br>O 1.84005 -2.43190 0.00000<br>O -0.65393 -3.33281 0.00000<br>C -2.51706 1.18639 0.00000<br>H -2.49497 1.83431 0.88269<br>H -2.49497 1.83431 -0.88269<br>H -3.46511 0.64613 0.00000<br>S 0.20625 2.44263 0.00000<br>C 1.99814 2.68056 0.00000<br>H 2.14436 3.76158 0.00000<br>H 2.45378 2.26051 0.89809<br>H 2.45378 2.26051 -0.89809<br>H 1.41619 -3.31079 0.00000	C_s	-858.75776<br>(0.4)	-858.59089<br>(0.3)	-858.64027<br>(0.5)	-858.89971<br>(0.0)	-858.78222<br>(0.0)
6-adduct, 4-phenoxyl, conf1	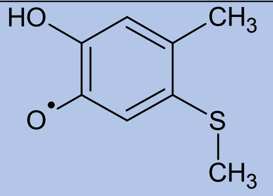<br>[2-hydroxy-4-methyl-5-(methylsulfanyl)phenyl]oxidanyl<br><br>C -1.70105 -0.94575 -0.01211<br>C -2.04601 0.46647 0.09271	C_1	-858.75111<br>(4.5)	-858.58439<br>(4.4)	-858.63449<br>(4.1)	-858.89103<br>(5.4)	-858.77441<br>(4.9)

	C -1.07553 1.45023 0.02216<br>C 0.26857 1.11359 -0.15341<br>C 0.64397 -0.27505 -0.23914<br>C -0.30786 -1.26189 -0.17495<br>H -1.36990 2.49220 0.09457<br>H -0.03549 -2.30964 -0.24229<br>O -2.62598 -1.79321 0.05341<br>O -3.33470 0.75834 0.25171<br>C 1.28573 2.21247 -0.23582<br>H 0.81653 3.15296 -0.52989<br>H 1.75602 2.37090 0.74157<br>H 2.07902 1.96741 -0.94560<br>S 2.33613 -0.77562 -0.52115<br>C 3.10452 -0.40294 1.08631<br>H 4.11290 -0.81895 1.04433<br>H 3.17237 0.67101 1.26286<br>H 2.55106 -0.88543 1.89319<br>H -3.80912 -0.09456 0.26438						
6-adduct, 4-phenoxyl, conf2	C -1.65795 -0.96225 -0.06543<br>C -2.04226 0.43197 0.11442<br>C -1.10242 1.44869 0.08267<br>C 0.24736 1.15994 -0.12224<br>C 0.66099 -0.21046 -0.28331<br>C -0.25810 -1.22792 -0.26469<br>H -1.42847 2.47556 0.21124<br>H 0.04533 -2.26141 -0.39352<br>O -2.55712 -1.83889 -0.03138<br>O -3.33623 0.67652 0.30410<br>C 1.23829 2.28392 -0.18735<br>H 2.01425 2.17640 0.57659<br>H 1.74720 2.29261 -1.15593<br>H 0.74464 3.24596 -0.04211<br>S 2.38202 -0.62068 -0.55241<br>C 2.97426 -0.66728 1.16975<br>H 4.03874 -0.90507 1.12686<br>H 2.84573 0.29973 1.65893<br>H 2.45105 -1.44332 1.73033<br>H -3.78485 -0.19020 0.28154	C_1	-858.75124 (4.5)	-858.58443 (4.4)	-858.63415 (4.3)	-858.89110 (5.4)	-858.77401 (5.2)
6-adduct, 4-phenoxyl, conf3	C -0.95422 -1.52618 0.00000<br>C 0.40644 -2.05529 0.00000<br>C 1.50424 -1.21323 0.00000<br>C 1.33705 0.16980 0.00000<br>C 0.00000 0.72402 0.00000	C_s	-858.75274 (3.5)	saddle point			

	C -1.09925 -0.10112 0.00000<br>H 2.50353 -1.63713 0.00000<br>H -2.11247 0.28055 0.00000<br>O -1.90332 -2.34981 0.00000<br>O 0.52936 -3.38090 0.00000<br>C 2.57312 1.02784 0.00000<br>H 3.18174 0.80451 0.88165<br>H 3.18174 0.80451 -0.88165<br>H 2.36254 2.09688 0.00000<br>S -0.12967 2.48887 0.00000<br>C -1.91295 2.77608 0.00000<br>H -2.03197 3.86054 0.00000<br>H -2.38147 2.36755 -0.89736<br>H -2.38147 2.36755 0.89736<br>H -0.38230 -3.73253 0.00000						
6-adduct, 4-phenoxyl, conf4	C 0.96440 -1.52758 0.00000<br>C -0.39352 -2.06171 0.00000<br>C -1.50047 -1.22513 0.00000<br>C -1.33737 0.15476 0.00000<br>C 0.00000 0.71462 0.00000<br>C 1.10437 -0.10136 0.00000<br>H -2.49513 -1.65841 0.00000<br>H 2.11519 0.28712 0.00000<br>O 1.91617 -2.34861 0.00000<br>O -0.51109 -3.38703 0.00000<br>C -2.54234 1.04931 0.00000<br>H -2.55068 1.69978 0.88160<br>H -2.55068 1.69978 -0.88160<br>H -3.46180 0.46222 0.00000<br>S 0.09887 2.47907 0.00000<br>C 1.87734 2.79605 0.00000<br>H 1.98025 3.88208 0.00000<br>H 2.35167 2.39416 0.89727<br>H 2.35167 2.39416 -0.89727<br>H 0.40245 -3.73468 0.00000	C_s	-858.75522 (2.0)	-858.58834 (1.9)	-858.63735 (2.3)	-858.89671 (1.9)	-858.77883 (2.1)
5-adduct, 3-phenoxyl, conf1	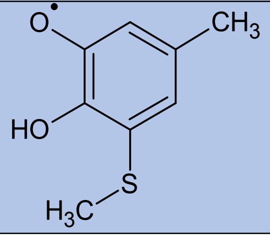	C_1	-858.75097 (4.6)	-858.58398 (4.7)	-858.63387 (4.5)	-858.89008 (6.0)	-858.77297 (5.8)

	[2-hydroxy-5-methyl-3-(methylsulfanyl)phenyl]oxidanyl<br><br>C -0.20069 0.94886 -0.16177<br>C 1.17925 1.39777 0.03817<br>C 2.20075 0.39217 0.13902<br>C 1.88647 -0.93761 0.05296<br>C 0.51969 -1.32625 -0.12507<br>C -0.52032 -0.41059 -0.23409<br>H 3.22325 0.72667 0.28303<br>H 0.28595 -2.38573 -0.17639<br>O -1.11677 1.90201 -0.26696<br>O 1.37632 2.63593 0.09779<br>C 2.93278 -2.01385 0.15264<br>H 3.92441 -1.58766 0.31591<br>H 2.70701 -2.69628 0.97793<br>H 2.95911 -2.60992 -0.76523<br>S -2.18745 -0.96154 -0.51877<br>C -2.99929 -0.41258 1.01591<br>H -4.04451 -0.71681 0.93685<br>H -2.54516 -0.89647 1.88168<br>H -2.94631 0.67204 1.11462<br>H -0.63280 2.74764 -0.18131						
5-adduct, 3-phenoxyl, conf2	C -0.07014 1.09217 -0.11428<br>C 1.35881 1.32766 0.11393<br>C 2.21008 0.15982 0.15199<br>C 1.72227 -1.10786 -0.01959<br>C 0.32451 -1.28207 -0.24502<br>C -0.55940 -0.20992 -0.28370<br>H 3.26754 0.33774 0.32278<br>H -0.06496 -2.28571 -0.39018<br>O -0.85045 2.16786 -0.14655<br>O 1.79645 2.48876 0.26664<br>C 2.61044 -2.32267 0.01252<br>H 3.64847 -2.05028 0.21316<br>H 2.28099 -3.02282 0.78645<br>H 2.57208 -2.85218 -0.94466<br>S -2.30385 -0.43829 -0.57485<br>C -2.87025 -0.87896 1.10112<br>H -3.94874 -1.03083 1.03231<br>H -2.39429 -1.80433 1.42778<br>H -2.66084 -0.07480 1.80726<br>H -1.76439 1.87384 -0.31974	C_1	-858.74739 (6.9)	-858.58064 (6.7)	-858.63066 (6.5)	-858.88770 (7.5)	-858.77097 (7.1)

5-adduct, 3-phenoxyl, conf3	C 1.18193 0.11262 0.00000<br>C 1.17006 1.57424 0.00000<br>C -0.10674 2.22808 0.00000<br>C -1.26232 1.49236 0.00000<br>C -1.19903 0.06140 0.00000<br>C 0.00000 -0.63865 0.00000<br>H -0.12260 3.31343 0.00000<br>H -2.13884 -0.47894 0.00000<br>O 2.37448 -0.47311 0.00000<br>O 2.28533 2.15250 0.00000<br>C -2.61061 2.16776 0.00000<br>H -2.72278 2.80456 0.88217<br>H -2.72278 2.80456 -0.88217<br>H -3.42435 1.43991 0.00000<br>S 0.17042 -2.40225 0.00000<br>C -1.54892 -2.96533 0.00000<br>H -1.49568 -4.05503 0.00000<br>H -2.07672 -2.63795 0.89758<br>H -2.07672 -2.63795 -0.89758<br>H 3.02901 0.25355 0.00000	C_s	-858.75041 (5.0)	saddle point			
5-adduct, 3-phenoxyl, conf4	C -1.18205 0.11990 0.00000<br>C -1.16831 1.58031 0.00000<br>C 0.11148 2.23291 0.00000<br>C 1.26239 1.49419 0.00000<br>C 1.19829 0.05990 0.00000<br>C 0.00000 -0.63599 0.00000<br>H 0.12880 3.31795 0.00000<br>H 2.13952 -0.47873 0.00000<br>O -2.37504 -0.46437 0.00000<br>O -2.28204 2.16069 0.00000<br>C 2.62129 2.13947 0.00000<br>H 3.19321 1.83314 0.88177<br>H 3.19321 1.83314 -0.88177<br>H 2.54230 3.22817 0.00000<br>S -0.17724 -2.39865 0.00000<br>C 1.54047 -2.96628 0.00000<br>H 1.48476 -4.05585 0.00000<br>H 2.06894 -2.63987 -0.89757<br>H 2.06894 -2.63987 0.89757<br>H -3.02846 0.26344 0.00000	C_s	-858.75170 (4.2)	-858.58477 (4.2)	-858.63502 (3.8)	-858.89211 (4.8)	-858.77544 (4.3)
5-adduct, 3-phenoxyl, conf5	C 1.21444 0.08500 0.00000<br>C 1.23483 1.55014 0.00000<br>C -0.04036 2.22498 0.00000	C_s	-858.74064 (11.1)	saddle point			

	C -1.22115 1.53299 0.00000<br>C -1.18831 0.10774 0.00000<br>C 0.00000 -0.61676 0.00000<br>H -0.01880 3.31069 0.00000<br>H -2.13658 -0.41812 0.00000<br>O 2.41835 -0.49811 0.00000<br>O 2.32399 2.16617 0.00000<br>C -2.54862 2.24849 0.00000<br>H -2.64325 2.88800 0.88243<br>H -2.64325 2.88800 -0.88243<br>H -3.38339 1.54426 0.00000<br>S 0.09625 -2.39126 0.00000<br>C -1.63497 -2.91361 0.00000<br>H -1.60419 -4.00402 0.00000<br>H -2.15032 -2.57214 0.89904<br>H -2.15032 -2.57214 -0.89904<br>H 2.35617 -1.46270 0.00000						
5-adduct, 3-phenoxyl, conf6	C -1.21459 0.09348 0.00000<br>C -1.23175 1.55793 0.00000<br>C 0.04723 2.22999 0.00000<br>C 1.22238 1.53394 0.00000<br>C 1.18771 0.10467 0.00000<br>C 0.00000 -0.61440 0.00000<br>H 0.02829 3.31547 0.00000<br>H 2.13685 -0.42030 0.00000<br>O -2.41914 -0.48715 0.00000<br>O -2.31871 2.17659 0.00000<br>C 2.56187 2.21909 0.00000<br>H 3.14349 1.93166 0.88196<br>H 3.14349 1.93166 -0.88196<br>H 2.44979 3.30518 0.00000<br>S -0.10540 -2.38805 0.00000<br>C 1.62351 -2.91734 0.00000<br>H 1.58862 -4.00762 0.00000<br>H 2.13995 -2.57755 -0.89906<br>H 2.13995 -2.57755 0.89906<br>H -2.35940 -1.45195 0.00000	C_s	-858.74185 (10.4)	saddle point			

5-adduct, 4-phenoxyl, conf1	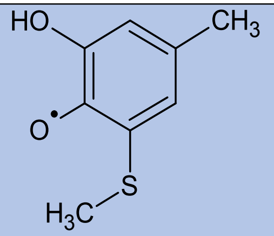 [2-hydroxy-4-methyl-6-(methylsulfanyl)phenyl]oxidanyl<br><br>C -0.22275 0.96097 -0.16024<br>C 1.18738 1.31550 0.01861<br>C 2.17833 0.36094 0.11299<br>C 1.83453 -0.99491 0.05335<br>C 0.47329 -1.37661 -0.08928<br>C -0.54396 -0.45490 -0.18737<br>H 3.21278 0.66605 0.23414<br>H 0.23270 -2.43515 -0.11172<br>O -1.04539 1.89684 -0.28608<br>O 1.45844 2.61992 0.06513<br>C 2.90332 -2.04259 0.17067<br>H 3.68025 -1.88537 -0.58415<br>H 3.38846 -1.98346 1.15060<br>H 2.49692 -3.04788 0.04692<br>S -2.20683 -0.99519 -0.46007<br>C -3.09926 -0.18369 0.90485<br>H -4.14454 -0.47978 0.80066<br>H -2.71786 -0.53302 1.86539<br>H -3.01341 0.89916 0.82694<br>H 0.60442 3.08011 -0.04159	C_1	-858.75363 (3.0)	-858.58667 (3.0)	-858.63661 (2.8)	-858.89270 (4.4)	-858.77568 (4.1)
5-adduct, 4-phenoxyl, conf2	C -0.21929 0.96589 -0.16335<br>C 1.18858 1.31747 0.02062<br>C 2.18222 0.35945 0.12180<br>C 1.83783 -0.99042 0.06067<br>C 0.47147 -1.37085 -0.08751<br>C -0.54202 -0.45077 -0.19160<br>H 3.21502 0.66695 0.24780<br>H 0.23256 -2.43023 -0.10944<br>O -1.04081 1.90335 -0.28898<br>O 1.46388 2.61969 0.06705<br>C 2.88302 -2.06153 0.16432<br>H 2.64916 -2.74912 0.98342	C_1	-858.75401 (2.7)	-858.58703 (2.7)	-858.63719 (2.4)	-858.89314 (4.1)	-858.77632 (3.7)

	H 2.91112 -2.65539 -0.75570<br>H 3.87488 -1.63928 0.33403<br>S -2.20799 -0.98463 -0.46759<br>C -3.08782 -0.19443 0.91768<br>H -4.13690 -0.47608 0.81160<br>H -2.70697 -0.56757 1.86950<br>H -2.99070 0.88907 0.86229<br>H 0.61116 3.08254 -0.04246						
5-adduct, 4-phenoxyl, conf3	C -0.30855 -1.18445 0.00156<br>C -1.72216 -0.82388 -0.00182<br>C -2.13445 0.48716 -0.00711<br>C -1.17258 1.51451 -0.00779<br>C 0.20530 1.21035 -0.00608<br>C 0.65566 -0.09793 -0.00096<br>H -3.19396 0.72205 -0.01003<br>H 0.90762 2.03576 -0.00851<br>O 0.00060 -2.40072 0.00541<br>O -2.58705 -1.84406 -0.00065<br>C -1.62707 2.94602 0.00817<br>H -2.37587 3.12375 -0.76957<br>H -2.09802 3.18095 0.96904<br>H -0.79567 3.63713 -0.14232<br>S 2.32655 -0.61541 0.00185<br>C 3.23064 0.95087 -0.00250<br>H 4.28751 0.68072 0.00065<br>H 3.01474 1.53001 -0.90232<br>H 3.01128 1.53712 0.89187<br>H -2.05162 -2.65856 0.00274	C_1	-858.75834<br>(0.0)	-858.59139<br>(0.0)	-858.64102<br>(0.0)	-858.89895<br>(0.5)	-858.78162<br>(0.4)
5-adduct, 4-phenoxyl, conf4	C 0.83016 0.47328 0.00000<br>C 0.13567 1.76283 0.00000<br>C -1.23101 1.86214 0.00000<br>C -2.00712 0.68328 0.00000<br>C -1.38463 -0.57248 0.00000<br>C 0.00000 -0.71791 0.00000<br>H -1.70694 2.83733 0.00000<br>H -2.00945 -1.46142 0.00000<br>O 2.08521 0.49727 0.00000<br>O 0.92957 2.84109 0.00000<br>C -3.50533 0.79064 0.00000<br>H -3.84964 1.34237 0.88074<br>H -3.84964 1.34237 -0.88074<br>H -3.98105 -0.19169 0.00000<br>S 0.60151 -2.36440 0.00000	C_s	-858.75171<br>(4.2)	saddle point			

	C 2.41376 -2.31797 0.00000<br>H 2.70165 -3.37126 0.00000<br>H 2.79985 -1.82867 -0.89198<br>H 2.79985 -1.82867 0.89198<br>H 1.84389 2.50033 0.00000						
5-adduct, 4-phenoxyl, conf5	C -0.82883 0.47943 0.00000<br>C -0.13254 1.76578 0.00000<br>C 1.23927 1.86511 0.00000<br>C 2.00834 0.68980 0.00000<br>C 1.37882 -0.57018 0.00000<br>C 0.00000 -0.71463 0.00000<br>H 1.71285 2.84099 0.00000<br>H 2.00622 -1.45794 0.00000<br>O -2.08398 0.50706 0.00000<br>O -0.92097 2.84574 0.00000<br>C 3.50930 0.75900 0.00000<br>H 3.91739 0.25308 0.88089<br>H 3.91739 0.25308 -0.88089<br>H 3.86409 1.79122 0.00000<br>S -0.60853 -2.36280 0.00000<br>C -2.42101 -2.30890 0.00000<br>H -2.71269 -3.36125 0.00000<br>H -2.80610 -1.81886 0.89200<br>H -2.80610 -1.81886 -0.89200<br>H -1.83699 2.50843 0.00000	C_s	-858.75151 (4.3)	saddle point			
5-adduct, 4-phenoxyl, conf6	C 1.21711 0.13040 0.00000<br>C 1.06932 1.58146 0.00000<br>C -0.16621 2.18273 0.00000<br>C -1.32467 1.38320 0.00000<br>C -1.22732 -0.02447 0.00000<br>C 0.00000 -0.66287 0.00000<br>H -0.24409 3.26515 0.00000<br>H -2.14737 -0.59738 0.00000<br>O 2.37414 -0.35549 0.00000<br>O 2.20701 2.28487 0.00000<br>C -2.67278 2.04533 0.00000<br>H -2.78427 2.68604 0.88073<br>H -2.78427 2.68604 -0.88073<br>H -3.48222 1.31331 0.00000<br>S 0.26466 -2.39205 0.00000<br>C -1.41879 -3.05348 0.00000<br>H -1.30761 -4.13859 0.00000<br>H -1.96285 -2.75184 0.89704	C_s	-858.75838 (0.0)	saddle point			

	H -1.96285 -2.75184 -0.89704<br>H 2.93193 1.63300 0.00000						
5-adduct, 4-phenoxyl, conf7	C -1.21892 0.13347 0.00000<br>C -1.07237 1.58270 0.00000<br>C 0.16622 2.18926 0.00000<br>C 1.31957 1.39340 0.00000<br>C 1.22172 -0.02057 0.00000<br>C 0.00000 -0.65931 0.00000<br>H 0.23842 3.27173 0.00000<br>H 2.14512 -0.58906 0.00000<br>O -2.37597 -0.35299 0.00000<br>O -2.20774 2.28678 0.00000<br>C 2.68231 2.02449 0.00000<br>H 3.25086 1.70869 0.88094<br>H 3.25086 1.70869 -0.88094<br>H 2.62038 3.11411 0.00000<br>S -0.26539 -2.39117 0.00000<br>C 1.41934 -3.04913 0.00000<br>H 1.31075 -4.13457 0.00000<br>H 1.96310 -2.74640 -0.89697<br>H 1.96310 -2.74640 0.89697<br>H -2.93386 1.63579 0.00000	C_s	-858.75845 (-0.1)	saddle point			

Table S12.6. One-electron oxidized anionic form in water.

Structure	Schematic drawing	Symmetry	G_{PCM}	$H_{PCM,RRHO}$	$G_{PCM,RRHO}$	$G_{SMD,\omega B97X\text{-}D,large}$	$G_{SMD,RRHO,\omega B97X\text{-}D,large}$
6-adduct, conf1	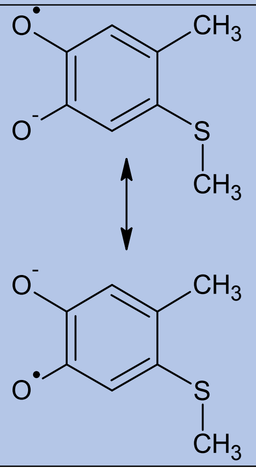	C_1	-858.28602 (2.2)	-858.13252 (2.1)	-858.18203 (2.8)	-858.42911 (2.6)	-858.32512 (3.1)

	[5-methyl-4-(methylsulfanyl)-2-oxidophenyl]oxidanyl<br><br>C -1.72302 -0.97977 -0.05552<br>C -2.15155 0.43537 0.13554<br>C -1.12477 1.44151 0.07862<br>C 0.20823 1.17209 -0.12858<br>C 0.61359 -0.20016 -0.28710<br>C -0.31914 -1.21212 -0.25858<br>H -1.45202 2.47143 0.20578<br>H -0.00564 -2.24498 -0.38597<br>O -2.55764 -1.92873 -0.03318<br>O -3.36298 0.72716 0.33327<br>C 1.20835 2.29675 -0.19693<br>H 0.71293 3.25912 -0.04876<br>H 1.98685 2.19581 0.56637<br>H 1.71615 2.31644 -1.16641<br>S 2.33106 -0.62787 -0.55080<br>C 2.93638 -0.66896 1.16705<br>H 3.99923 -0.91658 1.12727<br>H 2.81531 0.30321 1.64895<br>H 2.40697 -1.43403 1.73781						
6-adduct, conf2	C -0.94277 -1.61158 0.00000<br>C 0.44134 -2.16105 0.00000<br>C 1.51447 -1.20856 0.00000<br>C 1.34263 0.15666 0.00000<br>C 0.00000 0.67564 0.00000<br>C -1.07954 -0.18274 0.00000<br>H 2.52412 -1.61597 0.00000<br>H -2.09799 0.19025 0.00000<br>O -1.95067 -2.37571 0.00000<br>O 0.65181 -3.40823 0.00000<br>C 2.56616 1.04658 0.00000<br>H 3.18289 0.84871 0.88236<br>H 3.18289 0.84871 -0.88236<br>H 2.32738 2.11145 0.00000<br>S -0.18387 2.44663 0.00000<br>C -1.97446 2.69055 0.00000<br>H -2.12271 3.77186 0.00000<br>H -2.43537 2.26874 -0.89540<br>H -2.43537 2.26874 0.89540	C_s	-858.28498 (2.9)	saddle point			
6-adduct, conf3	C 0.95718 -1.61149 0.00000<br>C -0.42304 -2.16884 0.00000	C_s	-858.28792 (1.0)	-858.13435 (1.0)	-858.18363 (1.8)	-858.43320 (0.0)	-858.32891 (0.7)

	C -1.50643 -1.22478 0.00000<br>C -1.34081 0.13973 0.00000<br>C 0.00000 0.66704 0.00000<br>C 1.08617 -0.18101 0.00000<br>H -2.51127 -1.64237 0.00000<br>H 2.10211 0.19925 0.00000<br>O 1.97001 -2.36967 0.00000<br>O -0.62673 -3.41727 0.00000<br>C -2.53635 1.05760 0.00000<br>H -2.54527 1.70919 0.88152<br>H -2.54527 1.70919 -0.88152<br>H -3.46312 0.47951 0.00000<br>S 0.14654 2.43912 0.00000<br>C 1.93182 2.72093 0.00000<br>H 2.06005 3.80466 0.00000<br>H 2.40025 2.30752 0.89538<br>H 2.40025 2.30752 -0.89538						
5-adduct, conf1	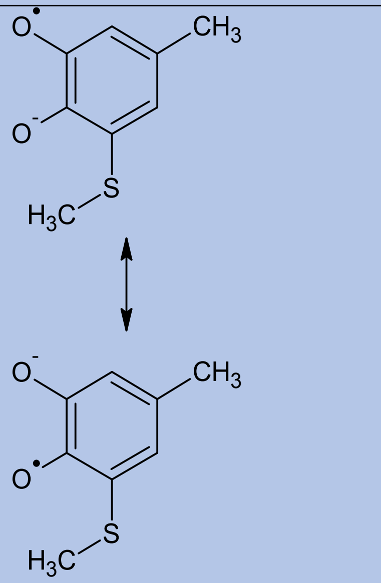<br><br>[5-methyl-3-(methylsulfanyl)-2-oxidophenyl]oxidanyl<br><br>C -0.22904 1.04591 -0.17634<br>C 1.20439 1.42668 0.02950<br>C 2.17568 0.37234 0.12486<br>C 1.84001 -0.95852 0.05266	C_1	-858.28723 (1.4)	-858.13373 (1.4)	-858.18378 (1.7)	-858.42950 (2.3)	-858.32605 (2.5)

	C 0.46967 -1.31969 -0.11081<br>C -0.52598 -0.37353 -0.22135<br>H 3.21278 0.67146 0.26240<br>H 0.20872 -2.37473 -0.14556<br>O -1.11216 1.93441 -0.29785<br>O 1.53194 2.64347 0.10297<br>C 2.87634 -2.04777 0.15612<br>H 3.87399 -1.63145 0.31580<br>H 2.65129 -2.72715 0.98545<br>H 2.90156 -2.65269 -0.75723<br>S -2.20119 -0.91099 -0.50031<br>C -3.03545 -0.28457 0.99056<br>H -4.09531 -0.52847 0.89223<br>H -2.63450 -0.76636 1.88447<br>H -2.91143 0.79698 1.05519						
5-adduct, conf2	C -0.26415 -1.26005 -0.00032<br>C -1.73219 -1.01299 0.00038<br>C -2.17482 0.35424 0.00023<br>C -1.30131 1.41498 -0.00014<br>C 0.10759 1.17749 -0.00026<br>C 0.60982 -0.10171 -0.00028<br>H -3.24922 0.52542 0.00047<br>H 0.76858 2.03859 -0.00034<br>O 0.20709 -2.43169 -0.00089<br>O -2.54090 -1.98483 0.00103<br>C -1.78615 2.84225 -0.00048<br>H -2.87771 2.89362 -0.00032<br>H -1.41828 3.38024 0.88025<br>H -1.41858 3.37965 -0.88170<br>S 2.33541 -0.51801 -0.00033<br>C 3.13158 1.10723 0.00112<br>H 4.20606 0.91542 0.00164<br>H 2.87595 1.67989 -0.89344<br>H 2.87488 1.67869 0.89615	C_1	-858.28954 (0.0)	-858.13591 (0.0)	-858.18643 (0.0)	-858.43316 (0.0)	-858.33005 (0.0)
5-adduct, conf3	C 0.90489 0.48169 0.00000<br>C 0.28361 1.84787 0.00000<br>C -1.14355 1.94201 0.00000<br>C -1.95534 0.82822 0.00000<br>C -1.36850 -0.46562 0.00000<br>C 0.00000 -0.65271 0.00000<br>H -1.57745 2.94008 0.00000<br>H -2.02808 -1.33121 0.00000<br>O 2.15827 0.36521 0.00000	C_s	-858.27913 (6.5)	saddle point			

	O 1.02957 2.86829 0.00000<br>C -3.45865 0.96712 0.00000<br>H -3.80214 1.51869 0.88133<br>H -3.80214 1.51869 -0.88133<br>H -3.95275 -0.00785 0.00000<br>S 0.50842 -2.36479 0.00000<br>C 2.32104 -2.46631 0.00000<br>H 2.52218 -3.54073 0.00000<br>H 2.75093 -2.01136 -0.88972<br>H 2.75093 -2.01136 0.88972						
5-adduct, conf4	C -0.90452 0.48717 0.00000<br>C -0.28187 1.84933 0.00000<br>C 1.14867 1.94308 0.00000<br>C 1.95690 0.83027 0.00000<br>C 1.36672 -0.46515 0.00000<br>C 0.00000 -0.64941 0.00000<br>H 1.58153 2.94120 0.00000<br>H 2.02738 -1.33043 0.00000<br>O -2.15878 0.37112 0.00000<br>O -1.02270 2.87338 0.00000<br>C 3.46000 0.94193 0.00000<br>H 3.89024 0.45290 0.88093<br>H 3.89024 0.45290 -0.88093<br>H 3.78094 1.98657 0.00000<br>S -0.51268 -2.36111 0.00000<br>C -2.32552 -2.46012 0.00000<br>H -2.52780 -3.53437 0.00000<br>H -2.75503 -2.00482 0.88971<br>H -2.75503 -2.00482 -0.88971	C_s	-858.28002 (6.0)	saddle point			
5-adduct, conf5	C 0.92862 0.47170 0.00000<br>C 0.33715 1.85255 0.00000<br>C -1.08718 1.98314 0.00000<br>C -1.92276 0.88868 0.00000<br>C -1.36343 -0.41734 0.00000<br>C 0.00000 -0.64325 0.00000<br>H -1.49662 2.99131 0.00000<br>H -2.04438 -1.26637 0.00000<br>O 2.18301 0.34244 0.00000<br>O 1.10749 2.85372 0.00000<br>C -3.42284 1.05875 0.00000<br>H -3.75444 1.61736 0.88151<br>H -3.75444 1.61736 -0.88151<br>H -3.93678 0.09405 0.00000	C_s	-858.27614 (8.4)	saddle point			

	S 0.42035 -2.38472 0.00000<br>C 2.23091 -2.60953 0.00000<br>H 2.71049 -1.63335 0.00000<br>H 2.51186 -3.17118 0.89221<br>H 2.51186 -3.17118 -0.89221						
5-adduct, conf6	C -0.92804 0.47731 0.00000<br>C -0.33497 1.85407 0.00000<br>C 1.09272 1.98397 0.00000<br>C 1.92450 0.89013 0.00000<br>C 1.36181 -0.41741 0.00000<br>C 0.00000 -0.64014 0.00000<br>H 1.50144 2.99206 0.00000<br>H 2.04379 -1.26612 0.00000<br>O -2.18333 0.34876 0.00000<br>O -1.10017 2.85907 0.00000<br>C 3.42487 1.03272 0.00000<br>H 3.86476 0.55259 0.88108<br>H 3.86476 0.55259 -0.88108<br>H 3.72370 2.08397 0.00000<br>S -0.42538 -2.38122 0.00000<br>C -2.23645 -2.60197 0.00000<br>H -2.71347 -1.62451 0.00000<br>H -2.51879 -3.16292 -0.89222<br>H -2.51879 -3.16292 0.89222	C_s	-858.27707 (7.8)	saddle point			
5-adduct, conf7	C 1.28411 0.05143 0.00000<br>C 1.27983 1.54275 0.00000<br>C 0.00540 2.19944 0.00000<br>C -1.18629 1.51003 0.00000<br>C -1.18186 0.08375 0.00000<br>C 0.00000 -0.62184 0.00000<br>H 0.00933 3.28782 0.00000<br>H -2.13813 -0.42908 0.00000<br>O 2.36350 -0.60308 0.00000<br>O 2.37167 2.18010 0.00000<br>C -2.50401 2.24814 0.00000<br>H -2.59380 2.89142 0.88154<br>H -2.59380 2.89142 -0.88154<br>H -3.35087 1.55697 0.00000<br>S 0.12903 -2.39129 0.00000<br>C -1.60346 -2.91488 0.00000<br>H -1.58628 -4.00632 0.00000<br>H -2.12730 -2.57039 0.89462<br>H -2.12730 -2.57039 -0.89462	C_s	-858.28844 (0.7)	saddle point			

5-adduct, conf8	C -1.28583 0.05431 0.00000<br>C -1.28190 1.54298 0.00000<br>C -0.00563 2.20295 0.00000<br>C 1.18394 1.51502 0.00000<br>C 1.18018 0.08615 0.00000<br>C 0.00000 -0.61853 0.00000<br>H -0.01263 3.29094 0.00000<br>H 2.13820 -0.42426 0.00000<br>O -2.36478 -0.60240 0.00000<br>O -2.37202 2.18315 0.00000<br>C 2.51097 2.22991 0.00000<br>H 3.10291 1.95784 0.88114<br>H 3.10291 1.95784 -0.88114<br>H 2.37823 3.31462 0.00000<br>S -0.12995 -2.38832 0.00000<br>C 1.60255 -2.91162 0.00000<br>H 1.58577 -4.00306 0.00000<br>H 2.12630 -2.56692 -0.89460<br>H 2.12630 -2.56692 0.89460	*C*s	-858.28950<br>(0.0)	saddle point			

Table S12.7. Two-electron oxidized neutral form in vacuo.

Structure	Schematic drawing	Symmetry	*E*	H_{RRHO}	G_{RRHO}	$E_{\omega B97X\text{-}D,large}$	$G_{RRHO,\omega B97X\text{-}D,large}$
6-adduct, conf1	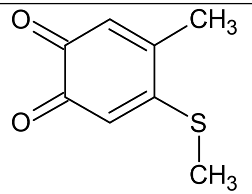<br>4-methyl-5-(methylsulfanyl)cyclohexa-3,5-diene-1,2-dione<br><br>C -1.08047 -1.54802 0.00000<br>C 0.31835 -2.20453 0.00000<br>C 1.47328 -1.29707 0.00000<br>C 1.35680 0.04532 0.00000<br>C 0.00000 0.66853 0.00000<br>C -1.12538 -0.08630 0.00000<br>H 2.45103 -1.77080 0.00000<br>H -2.12006 0.34159 0.00000<br>O -2.08156 -2.23640 0.00000	*C*s	-858.11824<br>(2.3)	saddle point			

	O 0.43514 -3.41190 0.00000<br>C 2.61003 0.89027 0.00000<br>H 3.21226 0.66099 0.88341<br>H 3.21226 0.66099 -0.88341<br>H 2.41852 1.96280 0.00000<br>S -0.01707 2.42617 0.00000<br>C -1.77830 2.81994 0.00000<br>H -1.83642 3.90958 0.00000<br>H -2.26946 2.43684 -0.89697<br>H -2.26946 2.43684 0.89697						
6-adduct, conf2	C 1.08794 -1.54893 0.00000<br>C -0.30998 -2.21033 0.00000<br>C -1.47176 -1.30863 0.00000<br>C -1.35506 0.03316 0.00000<br>C 0.00000 0.66078 0.00000<br>C 1.12959 -0.08673 0.00000<br>H -2.44612 -1.78772 0.00000<br>H 2.12215 0.34648 0.00000<br>O 2.09012 -2.23552 0.00000<br>O -0.42136 -3.41787 0.00000<br>C -2.57643 0.91029 0.00000<br>H -2.59843 1.55834 0.88224<br>H -2.59843 1.55834 -0.88224<br>H -3.48150 0.30081 0.00000<br>S -0.00852 2.41824 0.00000<br>C 1.74739 2.83613 0.00000<br>H 1.79181 3.92640 0.00000<br>H 2.24333 2.45913 0.89693<br>H 2.24333 2.45913 -0.89693	C_s	-858.12185<br>(0.0)	-857.96634<br>(0.0)	-858.01526<br>(0.0)	-858.26146<br>(0.0)	-858.15487<br>(0.0)
5-adduct, conf1	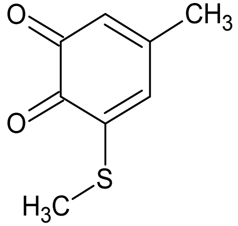<br>5-methyl-3-(methylsulfanyl)cyclohexa-3,5-diene-1,2-dione<br><br>C -0.25324 1.02003 -0.17773<br>C 1.23175 1.45569 0.02258<br>C 2.24745 0.40401 0.08344	C_1	-858.11807<br>(2.4)	-857.96293<br>(2.1)	-858.01319<br>(1.3)	-858.25697<br>(2.8)	-858.15209<br>(1.7)

	C 1.89959 -0.89900 0.05503<br>C 0.48849 -1.30472 -0.02367<br>C -0.54570 -0.44243 -0.12894<br>H 3.28112 0.72472 0.16727<br>H 0.28219 -2.37218 0.00318<br>O -1.09524 1.86526 -0.37367<br>O 1.48503 2.63815 0.11832<br>C 2.91140 -2.00479 0.12964<br>H 3.92662 -1.61440 0.21845<br>H 2.70824 -2.65101 0.99063<br>H 2.85552 -2.63372 -0.76585<br>S -2.18451 -1.04667 -0.38687<br>C -3.16231 -0.06937 0.79699<br>H -4.17540 -0.47236 0.74173<br>H -2.78233 -0.20229 1.81142<br>H -3.16666 0.98415 0.52192						
5-adduct, conf2	C 0.91307 0.47179 0.00000<br>C 0.25327 1.89092 0.00000<br>C -1.19976 1.96792 0.00000<br>C -1.95136 0.84361 0.00000<br>C -1.33607 -0.48245 0.00000<br>C 0.00000 -0.71049 0.00000<br>H -1.64343 2.95866 0.00000<br>H -2.01422 -1.33265 0.00000<br>O 2.11952 0.39423 0.00000<br>O 0.98666 2.85860 0.00000<br>C -3.45736 0.92008 0.00000<br>H -3.81314 1.46034 0.88218<br>H -3.81314 1.46034 -0.88218<br>H -3.91867 -0.06953 0.00000<br>S 0.54477 -2.38644 0.00000<br>C 2.36011 -2.40011 0.00000<br>H 2.60350 -3.46529 0.00000<br>H 2.77088 -1.92953 -0.89104<br>H 2.77088 -1.92953 0.89104	C_s	-858.11306 (5.5)	saddle point			
5-adduct, conf3	C -0.91059 0.47783 0.00000<br>C -0.24887 1.89636 0.00000<br>C 1.20624 1.97304 0.00000<br>C 1.95217 0.84701 0.00000<br>C 1.33516 -0.48035 0.00000<br>C 0.00000 -0.70674 0.00000<br>H 1.65081 2.96281 0.00000<br>H 2.01620 -1.32906 0.00000	C_s	-858.11545 (4.0)	saddle point			

	O -2.11714 0.40296 0.00000<br>O -0.98029 2.86484 0.00000<br>C 3.45293 0.88349 0.00000<br>H 3.84712 0.36611 0.88151<br>H 3.84712 0.36611 -0.88151<br>H 3.83063 1.90740 0.00000<br>S -0.54920 -2.38223 0.00000<br>C -2.36468 -2.39135 0.00000<br>H -2.61071 -3.45592 0.00000<br>H -2.77438 -1.91993 0.89106<br>H -2.77438 -1.91993 -0.89106						
5-adduct, conf4	C 0.94082 0.46368 0.00000<br>C 0.31458 1.90167 0.00000<br>C -1.13469 2.02013 0.00000<br>C -1.91299 0.91492 0.00000<br>C -1.32891 -0.42505 0.00000<br>C 0.00000 -0.69693 0.00000<br>H -1.55152 3.02235 0.00000<br>H -2.03063 -1.25600 0.00000<br>O 2.14722 0.37346 0.00000<br>O 1.07531 2.84760 0.00000<br>C -3.41664 1.02619 0.00000<br>H -3.75951 1.57465 0.88216<br>H -3.75951 1.57465 -0.88216<br>H -3.90040 0.04749 0.00000<br>S 0.44258 -2.40674 0.00000<br>C 2.25724 -2.58909 0.00000<br>H 2.74744 -1.62050 0.00000<br>H 2.53807 -3.14822 0.89316<br>H 2.53807 -3.14822 -0.89316	C_s	-858.10907 (8.0)	saddle point			
5-adduct, conf5	C -0.93854 0.46959 0.00000<br>C -0.31043 1.90747 0.00000<br>C 1.14084 2.02566 0.00000<br>C 1.91345 0.91858 0.00000<br>C 1.32797 -0.42282 0.00000<br>C 0.00000 -0.69331 0.00000<br>H 1.55889 3.02683 0.00000<br>H 2.03275 -1.25202 0.00000<br>O -2.14500 0.38199 0.00000<br>O -1.06946 2.85402 0.00000<br>C 3.41281 0.99016 0.00000<br>H 3.81906 0.48249 0.88157<br>H 3.81906 0.48249 -0.88157	C_s	-858.11147 (6.5)	saddle point			

	H 3.76547 2.02292 0.00000<br>S -0.44667 -2.40296 0.00000<br>C -2.26186 -2.58096 0.00000<br>H -2.74970 -1.61118 0.00000<br>H -2.54424 -3.13928 -0.89315<br>H -2.54424 -3.13928 0.89315						
5-adduct, conf6	C 1.30189 0.06514 0.00000<br>C 1.25086 1.62143 0.00000<br>C -0.06554 2.24753 0.00000<br>C -1.19570 1.50491 0.00000<br>C -1.15255 0.03935 0.00000<br>C 0.00000 -0.66868 0.00000<br>H -0.09276 3.33295 0.00000<br>H -2.11065 -0.46981 0.00000<br>O 2.36498 -0.50962 0.00000<br>O 2.29709 2.23753 0.00000<br>C -2.55198 2.16490 0.00000<br>H -2.66852 2.80121 0.88211<br>H -2.66852 2.80121 -0.88211<br>H -3.36292 1.43360 0.00000<br>S 0.18447 -2.40982 0.00000<br>C -1.53830 -2.95489 0.00000<br>H -1.50671 -4.04566 0.00000<br>H -2.06506 -2.61898 0.89691<br>H -2.06506 -2.61898 -0.89691	C_s	-858.11775 (2.6)	saddle point			
5-adduct, conf7	C -1.30202 0.06934 0.00000<br>C -1.25228 1.62602 0.00000<br>C 0.06489 2.25510 0.00000<br>C 1.19217 1.51101 0.00000<br>C 1.15112 0.04349 0.00000<br>C 0.00000 -0.66487 0.00000<br>H 0.09113 3.34003 0.00000<br>H 2.11218 -0.46153 0.00000<br>O -2.36509 -0.50521 0.00000<br>O -2.29825 2.24146 0.00000<br>C 2.55732 2.13610 0.00000<br>H 3.12348 1.81634 0.88191<br>H 3.12348 1.81634 -0.88191<br>H 2.50000 3.22588 0.00000<br>S -0.18559 -2.40660 0.00000<br>C 1.53737 -2.95107 0.00000<br>H 1.50629 -4.04186 0.00000<br>H 2.06408 -2.61506 -0.89690	C_s	-858.12016 (1.1)	-857.96499 (0.8)	-858.01445 (0.5)	-858.25974 (1.1)	-858.15404 (0.5)

	H 2.06408 -2.61506 0.89690						

Table S12.8. Two-electron oxidized neutral form in water.

Structure	Schematic drawing	Symmetry	G_{PCM}	$H_{PCM,RRHO}$	$G_{PCM,RRHO}$	$G_{SMD,\omega B97X-D,large}$	$G_{SMD,RRHO,\omega B97X-D,large}$
6-adduct, conf1	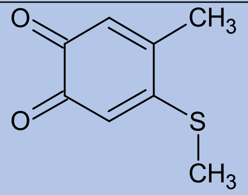<br>4-methyl-5-(methylsulfanyl)cyclohexa-3,5-diene-1,2-dione<br><br>C -1.08010 -1.53247 0.00000<br>C 0.31889 -2.19151 0.00000<br>C 1.47462 -1.30061 0.00000<br>C 1.35864 0.04330 0.00000<br>C 0.00000 0.66935 0.00000<br>C -1.13150 -0.08455 0.00000<br>H 2.45312 -1.77151 0.00000<br>H -2.12202 0.35225 0.00000<br>O -2.07574 -2.24240 0.00000<br>O 0.41545 -3.40676 0.00000<br>C 2.61172 0.88260 0.00000<br>H 3.21174 0.64643 0.88275<br>H 3.21174 0.64643 -0.88275<br>H 2.42577 1.95547 0.00000<br>S -0.01466 2.41681 0.00000<br>C -1.77237 2.82714 0.00000<br>H -1.81493 3.91691 0.00000<br>H -2.26401 2.44943 -0.89824<br>H -2.26401 2.44943 0.89824	C_s	-858.13411<br>(2.2)	saddle point			
6-adduct, conf2	C 1.08644 -1.53426 0.00000<br>C -0.31193 -2.19746 0.00000<br>C -1.47419 -1.31115 0.00000<br>C -1.35739 0.03193 0.00000<br>C 0.00000 0.66166 0.00000<br>C 1.13506 -0.08602 0.00000<br>H -2.44921 -1.78781 0.00000<br>H 2.12386 0.35502 0.00000	C_s	-858.13754<br>(0.0)	-857.98234<br>(0.0)	-858.03151<br>(0.0)	-858.27879<br>(0.0)	-858.17276 (0.0)

	O 2.08281 -2.24293 0.00000<br>O -0.40442 -3.41244 0.00000<br>C -2.57909 0.90454 0.00000<br>H -2.60023 1.55136 0.88277<br>H -2.60023 1.55136 -0.88277<br>H -3.48228 0.29336 0.00000<br>S -0.00831 2.40888 0.00000<br>C 1.74431 2.84175 0.00000<br>H 1.77384 3.93194 0.00000<br>H 2.24043 2.46989 0.89818<br>H 2.24043 2.46989 -0.89818						
5-adduct, conf1	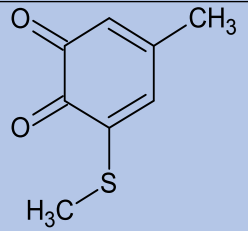<br>5-methyl-3-(methylsulfanyl)cyclohexa-3,5-diene-1,2-dione<br><br>C -0.23039 1.03146 -0.19863<br>C 1.25929 1.43150 0.03864<br>C 2.24784 0.36613 0.11376<br>C 1.87214 -0.92985 0.05484<br>C 0.45165 -1.30339 -0.06687<br>C -0.55840 -0.41600 -0.17685<br>H 3.28708 0.65610 0.23030<br>H 0.22609 -2.36646 -0.06654<br>O -1.04513 1.90644 -0.40325<br>O 1.52689 2.61590 0.14380<br>C 2.85369 -2.05742 0.13437<br>H 3.87517 -1.69325 0.25093<br>H 2.60989 -2.71139 0.97834<br>H 2.79546 -2.66950 -0.77215<br>S -2.22340 -0.95464 -0.44489<br>C -3.09695 -0.14351 0.93210<br>H -4.13927 -0.45677 0.85233<br>H -2.69155 -0.47736 1.88805<br>H -3.03578 0.94057 0.84437	C_1	-858.13194 (3.5)	-857.97692 (3.4)	-858.02703 (2.8)	-858.27093 (4.9)	-858.16601 (4.2)
5-adduct, conf2	C 0.90690 0.47109 0.00000<br>C 0.24623 1.89061 0.00000	C_s	-858.12547 (7.6)	saddle point			

	C -1.19652 1.97388 0.00000<br>C -1.94961 0.84693 0.00000<br>C -1.33535 -0.48092 0.00000<br>C 0.00000 -0.71001 0.00000<br>H -1.64587 2.96178 0.00000<br>H -2.01730 -1.32719 0.00000<br>O 2.11639 0.40454 0.00000<br>O 0.99404 2.85587 0.00000<br>C -3.45207 0.92388 0.00000<br>H -3.80301 1.46824 0.88143<br>H -3.80301 1.46824 -0.88143<br>H -3.91349 -0.06471 0.00000<br>S 0.54361 -2.38888 0.00000<br>C 2.35839 -2.41383 0.00000<br>H 2.59539 -3.47987 0.00000<br>H 2.76912 -1.94877 -0.89387<br>H 2.76912 -1.94877 0.89387						
5-adduct, conf3	C -0.90479 0.47694 0.00000<br>C -0.24254 1.89615 0.00000<br>C 1.20265 1.97962 0.00000<br>C 1.95036 0.85143 0.00000<br>C 1.33433 -0.47805 0.00000<br>C 0.00000 -0.70564 0.00000<br>H 1.65196 2.96705 0.00000<br>H 2.01942 -1.32254 0.00000<br>O -2.11443 0.41314 0.00000<br>O -0.98823 2.86202 0.00000<br>C 3.44782 0.88827 0.00000<br>H 3.83723 0.36588 0.88031<br>H 3.83723 0.36588 -0.88031<br>H 3.82645 1.91125 0.00000<br>S -0.54736 -2.38474 0.00000<br>C -2.36233 -2.40569 0.00000<br>H -2.60168 -3.47123 0.00000<br>H -2.77228 -1.93996 0.89387<br>H -2.77228 -1.93996 -0.89387	C_s	-858.12787 (6.1)	saddle point			
5-adduct, conf4	C 0.93341 0.46310 0.00000<br>C 0.30489 1.90199 0.00000<br>C -1.13422 2.02441 0.00000<br>C -1.91285 0.91584 0.00000<br>C -1.32858 -0.42530 0.00000<br>C 0.00000 -0.69654 0.00000<br>H -1.55850 3.02324 0.00000	C_s	-858.12110 (10.3)	saddle point			

	H -2.03300 -1.25311 0.00000<br>O 2.14300 0.38302 0.00000<br>O 1.07898 2.84567 0.00000<br>C -3.41311 1.02630 0.00000<br>H -3.75131 1.57848 0.88147<br>H -3.75131 1.57848 -0.88147<br>H -3.89624 0.04825 0.00000<br>S 0.44558 -2.40785 0.00000<br>C 2.26003 -2.59812 0.00000<br>H 2.75382 -1.63225 0.00000<br>H 2.53701 -3.15853 0.89296<br>H 2.53701 -3.15853 -0.89296						
5-adduct, conf5	C -0.93135 0.46907 0.00000<br>C -0.30213 1.90741 0.00000<br>C 1.13941 2.03055 0.00000<br>C 1.91316 0.92076 0.00000<br>C 1.32768 -0.42208 0.00000<br>C 0.00000 -0.69179 0.00000<br>H 1.56347 3.02904 0.00000<br>H 2.03525 -1.24801 0.00000<br>O -2.14100 0.39083 0.00000<br>O -1.07436 2.85155 0.00000<br>C 3.40937 0.99142 0.00000<br>H 3.81053 0.47812 0.88036<br>H 3.81053 0.47812 -0.88036<br>H 3.76409 2.02292 0.00000<br>S -0.44857 -2.40376 0.00000<br>C -2.26354 -2.59093 0.00000<br>H -2.75562 -1.62410 0.00000<br>H -2.54190 -3.15066 -0.89303<br>H -2.54190 -3.15066 0.89303	C_s	-858.12353 (8.8)	saddle point			
5-adduct, conf6	C 1.29367 0.06363 0.00000<br>C 1.24711 1.62223 0.00000<br>C -0.05434 2.25300 0.00000<br>C -1.18826 1.50978 0.00000<br>C -1.14964 0.04311 0.00000<br>C 0.00000 -0.66869 0.00000<br>H -0.08648 3.33792 0.00000<br>H -2.10954 -0.46114 0.00000<br>O 2.36672 -0.50213 0.00000<br>O 2.30981 2.22370 0.00000<br>C -2.53930 2.17164 0.00000<br>H -2.64735 2.81017 0.88143	C_s	-858.13366 (2.4)	saddle point			

	H -2.64735 2.81017 -0.88143<br>H -3.35129 1.44329 0.00000<br>S 0.17135 -2.41332 0.00000<br>C -1.55122 -2.96159 0.00000<br>H -1.51371 -4.05162 0.00000<br>H -2.07315 -2.62350 0.89750<br>H -2.07315 -2.62350 -0.89750						
5-adduct, conf7	C -1.29422 0.06664 0.00000<br>C -1.25053 1.62570 0.00000<br>C 0.05131 2.26119 0.00000<br>C 1.18294 1.51772 0.00000<br>C 1.14744 0.04884 0.00000<br>C 0.00000 -0.66431 0.00000<br>H 0.08035 3.34585 0.00000<br>H 2.11073 -0.45014 0.00000<br>O -2.36688 -0.49988 0.00000<br>O -2.31327 2.22553 0.00000<br>C 2.54318 2.14469 0.00000<br>H 3.10705 1.81847 0.88052<br>H 3.10705 1.81847 -0.88052<br>H 2.48450 3.23379 0.00000<br>S -0.16999 -2.41025 0.00000<br>C 1.55369 -2.95538 0.00000<br>H 1.51838 -4.04549 0.00000<br>H 2.07506 -2.61636 -0.89748<br>H 2.07506 -2.61636 0.89748	C_s	-858.13610 (0.9)	-857.98103 (0.8)	-858.03125 (0.2)	-858.27617 (1.6)	-858.17132 (0.9)

Part 13: 4-Methylcatechol – thioacetic acid adducts, optimizations using the ωB97X-D functional

Table S13.1. Reduced neutral form in vacuo.

Structure	Schematic drawing	Symmetry	E	H_{RRHO}	G_{RRHO}	$E_{\omega B97X\text{-}D,large}$	$G_{RRHO,\omega B97X\text{-}D,large}$
6-adduct, conf1	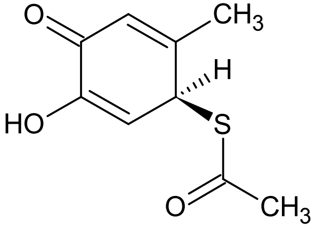<br>*S*-[(1*S*)-5-hydroxy-2-methyl-4-oxocyclohexa-2,5-dien-1-yl] ethanethioate<br><br>C 2.20461 -0.93766 0.20747<br>C 2.66660 0.42439 -0.16465<br>C 1.67281 1.49394 -0.18817<br>C 0.37212 1.27311 0.07575<br>C -0.12033 -0.11129 0.41535<br>C 0.91773 -1.18312 0.48566<br>H 2.04521 2.48855 -0.41375<br>H -0.66453 -0.04777 1.36902<br>H 0.59564 -2.18178 0.76188<br>O 3.17115 -1.86934 0.25254<br>O 3.85787 0.58955 -0.41286<br>C -0.63372 2.38552 0.08829<br>H -1.17868 2.39966 1.03750<br>H -0.14977 3.35259 -0.05954<br>H -1.37587 2.24552 -0.70488<br>S -1.43201 -0.65846 -0.76667<br>C -2.90527 -0.29911 0.18443<br>C -4.18476 -0.61980 -0.54828<br>H -4.56940 0.30651 -0.98663<br>H -4.03739 -1.34991 -1.34651<br>H -4.91812 -0.98806 0.17147<br>O -2.87514 0.17529 1.29651<br>H 3.99526 -1.40986 0.01364	C_1	-972.66357 (0.0)	-972.47204 (0.0)	-972.52600 (0.0)	-972.83596 (0.0)	-972.69839 (0.0)
6-adduct, conf2	C -2.16977 -0.93700 -0.24955<br>C -2.64857 0.40821 0.16178<br>C -1.67179 1.49547 0.18972	C_1	-972.65325 (6.5)	-972.46204 (6.3)	-972.51692 (5.7)	-972.82636 (6.0)	-972.69003 (5.3)

	C -0.37412 1.30242 -0.10351<br>C 0.14671 -0.07417 -0.44114<br>C -0.88245 -1.15422 -0.54778<br>H -2.05825 2.47869 0.43988<br>H 0.68957 -0.00802 -1.38985<br>H -0.54561 -2.14350 -0.83949<br>O -3.11980 -1.88349 -0.30519<br>O -3.83682 0.54988 0.43181<br>C 0.60532 2.43847 -0.12794<br>H 1.02449 2.56223 -1.13352<br>H 0.12606 3.37709 0.15518<br>H 1.43729 2.25341 0.55927<br>S 1.38659 -0.61829 0.83520<br>C 3.02273 -0.41284 0.12067<br>C 3.19902 0.00233 -1.32106<br>H 2.72426 -0.71604 -1.99606<br>H 2.76605 0.98901 -1.50535<br>H 4.26939 0.03563 -1.52587<br>O 3.95261 -0.65373 0.85150<br>H -3.94902 -1.44903 -0.03949						
6-adduct, conf3	C 1.81820 -0.80762 0.54393<br>C 2.23517 0.22857 -0.43460<br>C 1.36790 1.39219 -0.59980<br>C 0.20834 1.53047 0.06921<br>C -0.22265 0.49325 1.07663<br>C 0.67676 -0.69031 1.23524<br>H 1.70968 2.14677 -1.30191<br>H -0.26006 1.01728 2.04442<br>H 0.38285 -1.45259 1.94926<br>O 2.65970 -1.84819 0.65814<br>O 3.28401 0.06293 -1.05079<br>C -0.66945 2.73298 -0.11760<br>H -0.95163 3.16616 0.84751<br>H -0.16224 3.49670 -0.70998<br>H -1.60241 2.46017 -0.62141<br>S -2.00884 0.00879 0.95887<br>C -2.27643 -0.84437 -0.60934<br>C -1.15005 -1.02824 -1.58974<br>H -0.78275 -0.05833 -1.93689<br>H -0.31291 -1.55272 -1.12258<br>H -1.52868 -1.60257 -2.43570<br>O -3.40248 -1.23158 -0.80010<br>H 3.39297 -1.66826 0.04371	C_1	-972.65659 (4.4)	-972.46549 (4.1)	-972.52039 (3.5)	-972.82939 (4.1)	-972.69319 (3.3)

6-adduct, conf4	C -1.75227 -0.84516 -0.55106<br>C -2.19150 0.12577 0.48351<br>C -1.40191 1.34140 0.65322<br>C -0.27241 1.56595 -0.03858<br>C 0.18800 0.58949 -1.09416<br>C -0.66087 -0.62425 -1.29277<br>H -1.76488 2.05112 1.39012<br>H 0.22776 1.14844 -2.04079<br>H -0.35931 -1.33287 -2.05694<br>O -2.54192 -1.92380 -0.68069<br>O -3.20633 -0.12750 1.12678<br>C 0.54652 2.80649 0.16612<br>H 0.78506 3.28552 -0.78960<br>H 0.01406 3.52366 0.79348<br>H 1.49893 2.56408 0.64860<br>S 1.96965 0.11602 -0.92580<br>C 1.97792 -0.76058 0.64173<br>C 3.29152 -1.44843 0.92569<br>H 3.23931 -2.46226 0.51586<br>H 4.13737 -0.93481 0.46309<br>H 3.43035 -1.51531 2.00582<br>O 1.01995 -0.80787 1.37123<br>H -3.24660 -1.81469 -0.01770	C_1	-972.65935 (2.7)	-972.46822 (2.4)	-972.52326 (1.7)	-972.83152 (2.8)	-972.69543 (1.9)
6-adduct, conf5	C 2.20071 -0.94200 0.20774<br>C 2.66849 0.41859 -0.16262<br>C 1.67909 1.49223 -0.18593<br>C 0.37730 1.27618 0.07642<br>C -0.12121 -0.10696 0.41242<br>C 0.91233 -1.18304 0.48296<br>H 2.05577 2.48562 -0.40975<br>H -0.66763 -0.04315 1.36491<br>H 0.58586 -2.18072 0.75754<br>O 3.16380 -1.87717 0.25394<br>O 3.86068 0.57913 -0.40947<br>C -0.62458 2.39217 0.08947<br>H -1.17121 2.40602 1.03776<br>H -0.13706 3.35788 -0.05545<br>H -1.36594 2.25643 -0.70516<br>S -1.43330 -0.64251 -0.77416<br>C -2.90507 -0.29557 0.18105<br>C -4.18671 -0.63101 -0.54182<br>H -4.72888 0.30155 -0.72242<br>H -4.01634 -1.13927 -1.49251	C_1	-972.66359 (0.0)	-972.47202 (0.0)	-972.52605 (0.0)	-972.83599 (0.0)	-972.69844 (0.0)

	H -4.80032 -1.25714 0.11029<br>O -2.87616 0.17180 1.29639<br>H 3.98989 -1.42056 0.01632						
6-adduct, conf6	C 1.85219 -1.14718 0.10704<br>C 2.65024 0.08280 -0.13021<br>C 1.96571 1.36831 -0.00494<br>C 0.65185 1.46113 0.26561<br>C -0.19207 0.21810 0.42726<br>C 0.54365 -1.08128 0.38352<br>H 2.58485 2.25316 -0.11608<br>H -0.71859 0.31733 1.38301<br>H -0.02025 -1.99558 0.53913<br>O 2.54194 -2.29581 0.02501<br>O 3.84299 -0.03380 -0.39262<br>C -0.02686 2.78739 0.44922<br>H -0.54337 2.82442 1.41498<br>H 0.69717 3.60325 0.41388<br>H -0.78230 2.95350 -0.32478<br>S -1.50725 0.25299 -0.88931<br>C -2.97829 -0.45943 -0.14520<br>C -2.98221 -0.88122 1.30561<br>H -2.16930 -1.57578 1.53171<br>H -2.88066 -0.00783 1.95752<br>H -3.93913 -1.36366 1.50613<br>O -3.94428 -0.55819 -0.86149<br>H 3.45711 -2.04589 -0.19122	C_1	-972.65314 (6.6)	-972.46204 (6.3)	-972.51734 (5.5)	-972.82601 (6.3)	-972.69021 (5.2)
5-adduct, conf1	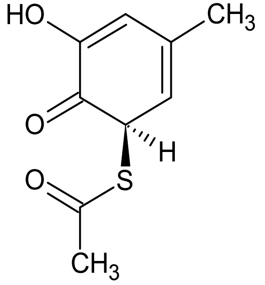<br>*S*-[(1*R*)-5-hydroxy-3-methyl-6-oxocyclohexa-2,4-dien-1-yl] ethanethioate<br><br>C -0.36357 -1.17643 -0.57184<br>C -1.60283 -1.17522 0.23026<br>C -2.27825 -0.03604 0.47604<br>C -1.81968 1.25704 -0.02293<br>C -0.71559 1.34704 -0.78273	C_1	-972.65647 (4.5)	-972.46547 (4.1)	-972.51955 (4.1)	-972.82798 (5.0)	-972.69106 (4.6)

	C 0.07383 0.14228 -1.19595<br>H -3.17736 -0.07728 1.08422<br>H -0.37266 2.30571 -1.16021<br>H -0.05808 -0.00943 -2.27795<br>O 0.22097 -2.23188 -0.76478<br>O -1.97621 -2.37586 0.70489<br>C -2.63379 2.46372 0.35702<br>H -3.66507 2.36256 0.00150<br>H -2.67379 2.57356 1.44623<br>H -2.21247 3.37895 -0.06491<br>S 1.88064 0.39357 -1.05944<br>C 2.05224 0.27852 0.71497<br>C 3.46872 0.44215 1.20806<br>H 3.91039 -0.55363 1.31469<br>H 4.08498 1.02582 0.52116<br>H 3.44454 0.91668 2.19046<br>O 1.11145 0.04927 1.43727<br>H -1.30694 -3.01071 0.39952						
5-adduct, conf2	C 0.43728 1.20704 -0.52668<br>C 1.72006 1.09945 0.19782<br>C 2.31408 -0.09440 0.40139<br>C 1.73942 -1.34164 -0.09623<br>C 0.58843 -1.33063 -0.79168<br>C -0.12830 -0.06191 -1.14332<br>H 3.24285 -0.13022 0.96391<br>H 0.15399 -2.25057 -1.17072<br>H 0.00953 0.10083 -2.22477<br>O -0.08773 2.30518 -0.63354<br>O 2.20372 2.25750 0.67652<br>C 2.48619 -2.61147 0.20870<br>H 3.49077 -2.58055 -0.22670<br>H 2.60440 -2.74421 1.28953<br>H 1.96698 -3.48544 -0.19017<br>S -1.95685 -0.18973 -1.05512<br>C -2.37455 -0.30278 0.69255<br>C -1.31483 -0.09051 1.74279<br>H -0.41483 -0.67567 1.53829<br>H -1.04438 0.96993 1.77050<br>H -1.73508 -0.37491 2.70802<br>O -3.53533 -0.51068 0.94668<br>H 1.58337 2.95138 0.39473	C_1	-972.65138 (7.7)	-972.46048 (7.3)	-972.51504 (6.9)	-972.82336 (7.9)	-972.68703 (7.2)
5-adduct, conf3	C 0.50195 1.10645 -0.09020<br>C 1.96967 1.21091 0.01696	C_1	-972.65703 (4.1)	-972.46592 (3.8)	-972.52099 (3.2)	-972.82853 (4.7)	-972.69248 (3.7)

	C 2.74750 0.10952 0.03927<br>C 2.19000 -1.23679 -0.07673<br>C 0.86428 -1.41828 -0.20272<br>C -0.09839 -0.27559 -0.26689<br>H 3.82166 0.22670 0.15197<br>H 0.44241 -2.41405 -0.29913<br>H -0.58361 -0.25516 -1.25920<br>O -0.16902 2.12796 -0.05646<br>O 2.44845 2.46084 0.13977<br>C 3.16224 -2.38439 -0.04959<br>H 3.89550 -2.29233 -0.85819<br>H 3.71881 -2.39588 0.89406<br>H 2.65120 -3.34358 -0.15746<br>S -1.51010 -0.51284 0.86705<br>C -2.86511 -0.23690 -0.27715<br>C -4.21335 -0.19721 0.39556<br>H -4.96612 -0.58503 -0.29256<br>H -4.22647 -0.75764 1.33285<br>H -4.44578 0.84979 0.61449<br>O -2.70139 -0.08197 -1.46316<br>H 1.67698 3.05167 0.10814						
5-adduct, conf4	C -0.33907 1.06583 0.29430<br>C -1.70040 1.31168 -0.21568<br>C -2.60686 0.31555 -0.27160<br>C -2.29003 -1.04712 0.15683<br>C -1.05189 -1.36087 0.57416<br>C 0.06653 -0.36574 0.58712<br>H -3.60859 0.53345 -0.63082<br>H -0.80577 -2.37251 0.88205<br>H 0.57717 -0.37923 1.55453<br>O 0.42055 2.01509 0.44321<br>O -1.96864 2.57895 -0.57218<br>C -3.40670 -2.05313 0.11624<br>H -4.23977 -1.73263 0.75124<br>H -3.79527 -2.15742 -0.90259<br>H -3.07151 -3.03488 0.45768<br>S 1.28315 -0.92073 -0.69584<br>C 2.91085 -0.33907 -0.16831<br>C 3.09460 0.30813 1.17829<br>H 2.47965 1.20834 1.25511<br>H 2.80367 -0.38344 1.97568<br>H 4.15049 0.55712 1.28823<br>O 3.80825 -0.54571 -0.94681	C_1	-972.65152 (7.6)	-972.46036 (7.3)	-972.51522 (6.8)	-972.82349 (7.8)	-972.68720 (7.1)

	H -1.16400 3.09471 -0.39949						
5-adduct, conf5	C 0.84851 -1.19732 0.31534<br>C 2.24213 -0.85566 -0.02421<br>C 2.62799 0.42877 -0.16249<br>C 1.69736 1.54345 0.01252<br>C 0.40362 1.31421 0.29279<br>C -0.16363 -0.06491 0.41259<br>H 3.66664 0.64314 -0.39779<br>H -0.29198 2.14096 0.40639<br>H -0.64950 -0.20594 1.38279<br>O 0.55162 -2.36512 0.51719<br>O 3.07758 -1.89933 -0.15760<br>C 2.25986 2.93020 -0.13640<br>H 3.05088 3.10831 0.60021<br>H 2.70383 3.06151 -1.12913<br>H 1.48798 3.69139 -0.00316<br>S -1.45764 -0.36123 -0.87742<br>C -3.01465 0.09188 -0.10911<br>C -3.06958 0.49541 1.34569<br>H -2.79677 -0.34917 1.98626<br>H -2.39357 1.32680 1.56293<br>H -4.09512 0.79227 1.56642<br>O -3.99226 0.03356 -0.81424<br>H 2.55468 -2.69856 0.02077	C_1	-972.64691 (10.5)	-972.45567 (10.3)	-972.51149 (9.1)	-972.81917 (10.6)	-972.68376 (9.2)

Table S13.2. Reduced neutral form in water.

Structure	Schematic drawing	Symmetry	G_{PCM}	$H_{PCM,RRHO}$	$G_{PCM,RRHO}$	$G_{SMD,\omega B97X\text{-}D,large}$	$G_{SMD,RRHO,\omega B97X\text{-}D,large}$
6-adduct, conf1	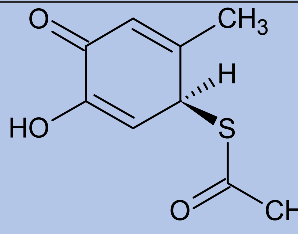<br>*S*-[(1*S*)-5-hydroxy-2-methyl-4-oxocyclohexa-2,5-dien-1-yl] ethanethioate<br><br>C 2.19281 -0.94164 0.20451<br>C 2.66403 0.41432 -0.16443	C_1	-972.67530 (0.0)	-972.48449 (0.0)	-972.53892 (0.0)	-972.84926 (0.0)	-972.71288 (0.2)

	C 1.68559 1.49197 -0.18207<br>C 0.38144 1.28296 0.08449<br>C -0.12312 -0.09789 0.41377<br>C 0.90476 -1.17828 0.48281<br>H 2.06281 2.48563 -0.40342<br>H -0.66598 -0.03912 1.36836<br>H 0.57114 -2.17307 0.75998<br>O 3.15165 -1.89007 0.24789<br>O 3.86199 0.56900 -0.41679<br>C -0.61077 2.40530 0.10830<br>H -1.12754 2.43405 1.07276<br>H -0.12145 3.36567 -0.06009<br>H -1.37557 2.26484 -0.66259<br>S -1.43185 -0.62578 -0.77631<br>C -2.91104 -0.31263 0.18329<br>C -4.18187 -0.60923 -0.56540<br>H -4.54740 0.32750 -0.99808<br>H -4.03124 -1.32914 -1.37157<br>H -4.92904 -0.98082 0.13781<br>O -2.87882 0.11782 1.31589<br>H 3.98425 -1.44617 0.01043						
6-adduct, conf2	C -2.15158 -0.93861 -0.26780<br>C -2.63804 0.38871 0.18159<br>C -1.68232 1.48823 0.21147<br>C -0.38498 1.31952 -0.10631<br>C 0.14804 -0.04581 -0.46444<br>C -0.86706 -1.13578 -0.58800<br>H -2.07527 2.46458 0.47728<br>H 0.69997 0.03275 -1.40598<br>H -0.51868 -2.10864 -0.91891<br>O -3.09350 -1.90103 -0.33596<br>O -3.82841 0.50563 0.47932<br>C 0.57427 2.46939 -0.14694<br>H 0.93293 2.62221 -1.17100<br>H 0.09877 3.39149 0.18998<br>H 1.44771 2.27841 0.48449<br>S 1.37390 -0.59625 0.82300<br>C 3.01816 -0.42722 0.13008<br>C 3.22698 0.03262 -1.28569<br>H 2.76956 -0.66878 -1.98935<br>H 2.79116 1.02083 -1.45056<br>H 4.30006 0.07872 -1.47154<br>O 3.92667 -0.72836 0.87625	C_1	-972.66935 (3.8)	-972.47830 (3.9)	-972.53233 (4.1)	-972.84573 (2.3)	-972.70872 (2.8)

	H -3.92764 -1.48777 -0.05305						
6-adduct, conf3	C 2.13571 -0.95894 0.24905<br>C 2.64871 0.37085 -0.16084<br>C 1.71086 1.48598 -0.17773<br>C 0.40617 1.33057 0.11588<br>C -0.15101 -0.03295 0.44279<br>C 0.84421 -1.14280 0.54837<br>H 2.12287 2.46154 -0.41601<br>H -0.70054 0.04012 1.38623<br>H 0.47591 -2.11739 0.85138<br>O 3.06138 -1.93800 0.30385<br>O 3.84477 0.47589 -0.43999<br>C -0.53930 2.49200 0.15533<br>H -0.97089 2.59628 1.15682<br>H -0.02941 3.42291 -0.09656<br>H -1.36679 2.34848 -0.54679<br>S -1.39101 -0.52922 -0.85197<br>C -3.02371 -0.42650 -0.12110<br>C -3.21343 -0.05055 1.32209<br>H -2.70174 -0.75961 1.97887<br>H -2.82283 0.95048 1.52196<br>H -4.28184 -0.06726 1.53749<br>O -3.94153 -0.69828 -0.86714<br>H 3.90521 -1.53086 0.04135	C_1	-972.66931 (3.8)	-972.47822 (3.9)	-972.53313 (3.6)	-972.84562 (2.3)	-972.70943 (2.4)
6-adduct, conf4	C -1.79401 -0.85548 -0.50389<br>C -2.22547 0.16882 0.47425<br>C -1.41091 1.36512 0.60996<br>C -0.26354 1.53652 -0.07420<br>C 0.17436 0.51807 -1.09648<br>C -0.68390 -0.69696 -1.23322<br>H -1.76028 2.11373 1.31447<br>H 0.18200 1.04063 -2.06442<br>H -0.38566 -1.44384 -1.96159<br>O -2.60318 -1.93238 -0.59837<br>O -3.25989 -0.03684 1.11716<br>C 0.58235 2.76099 0.10225<br>H 0.86629 3.18582 -0.86552<br>H 0.05176 3.51934 0.68018<br>H 1.51008 2.51212 0.62811<br>S 1.95525 0.05396 -0.96305<br>C 2.03193 -0.72592 0.64356<br>C 3.37668 -1.33225 0.94909<br>H 3.30645 -2.41106 0.77807	C_1	-972.67323 (1.3)	-972.48259 (1.2)	-972.53812 (0.5)	-972.84830 (0.6)	-972.71319 (0.0)

	H 4.17186 -0.92962 0.31949<br>H 3.60893 -1.16395 2.00232<br>O 1.08373 -0.77309 1.39543<br>H -3.32573 -1.78159 0.03597						
6-adduct, conf5	C -1.81850 -0.82285 -0.52133<br>C -2.24146 0.23554 0.42597<br>C -1.37676 1.39719 0.57586<br>C -0.21106 1.52262 -0.08810<br>C 0.21724 0.46880 -1.07758<br>C -0.67342 -0.72344 -1.20806<br>H -1.71602 2.16750 1.26151<br>H 0.24285 0.97262 -2.05584<br>H -0.37602 -1.49841 -1.90670<br>O -2.66166 -1.86980 -0.62529<br>O -3.30387 0.08606 1.03412<br>C 0.66536 2.72636 0.08324<br>H 0.97716 3.12347 -0.88760<br>H 0.14420 3.50962 0.63570<br>H 1.57846 2.46688 0.62914<br>S 2.00361 -0.00923 -0.96256<br>C 2.28425 -0.82032 0.61287<br>C 1.18100 -0.96539 1.61909<br>H 0.83609 0.01853 1.94896<br>H 0.32825 -1.48922 1.18075<br>H 1.56273 -1.52398 2.47384<br>O 3.41628 -1.21943 0.79241<br>H -3.40133 -1.68502 -0.02054	C_1	-972.67115 (2.6)	-972.48058 (2.5)	-972.53518 (2.3)	-972.84679 (1.6)	-972.71082 (1.5)
6-adduct, conf6	C 2.18633 -0.94876 0.20192<br>C 2.66766 0.40570 -0.15941<br>C 1.69627 1.48986 -0.17555<br>C 0.38998 1.28809 0.08590<br>C -0.12456 -0.09094 0.40719<br>C 0.89575 -1.17844 0.47425<br>H 2.08081 2.48191 -0.39137<br>H -0.66965 -0.03294 1.36076<br>H 0.55456 -2.17218 0.74593<br>O 3.13885 -1.90368 0.24473<br>O 3.86744 0.55386 -0.40695<br>C -0.59589 2.41606 0.11045<br>H -1.12109 2.43975 1.07054<br>H -0.10001 3.37508 -0.04610<br>H -1.35474 2.28598 -0.66811<br>S -1.43563 -0.59930 -0.78818	C_1	-972.67533 (0.0)	-972.48446 (0.0)	-972.53882 (0.1)	-972.84933 (0.0)	-972.71282 (0.2)

	C -2.91096 -0.31269 0.18236<br>C -4.18552 -0.61710 -0.55750<br>H -4.70516 0.32854 -0.73753<br>H -4.01122 -1.11589 -1.51182<br>H -4.81927 -1.23997 0.07779<br>O -2.87803 0.09885 1.32225<br>H 3.97536 -1.46428 0.01283						
6-adduct, conf7	C 1.88540 -1.12521 0.13405<br>C 2.64772 0.11717 -0.13625<br>C 1.93924 1.38598 -0.02710<br>C 0.62350 1.45330 0.25084<br>C -0.18713 0.19307 0.43389<br>C 0.57799 -1.08916 0.41865<br>H 2.53300 2.28611 -0.15235<br>H -0.72698 0.29427 1.38055<br>H 0.03400 -2.00809 0.61289<br>O 2.60444 -2.26479 0.07542<br>O 3.84559 0.02193 -0.41127<br>C -0.08416 2.76266 0.42693<br>H -0.58394 2.79458 1.40078<br>H 0.61725 3.59588 0.36497<br>H -0.85743 2.89617 -0.33581<br>S -1.48221 0.15258 -0.90205<br>C -2.98606 -0.45674 -0.14572<br>C -3.04110 -0.77056 1.32372<br>H -2.26218 -1.48031 1.61257<br>H -2.91187 0.14292 1.91178<br>H -4.01916 -1.19962 1.54168<br>O -3.93431 -0.58000 -0.89307<br>H 3.51451 -2.00328 -0.14707	C_1	-972.66909 (3.9)	-972.47838 (3.8)	-972.53361 (3.3)	-972.84502 (2.7)	-972.70954 (2.3)
5-adduct, conf1	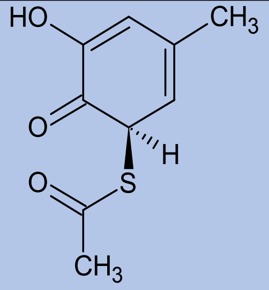<br>*S*-[(1*R*)-5-hydroxy-3-methyl-6-oxocyclohexa-2,4-dien-1-yl] ethanethioate<br><br>C -0.37667 -1.17471 -0.56136	C_1	-972.66906 (3.9)	-972.47861 (3.7)	-972.53304 (3.7)	-972.84288 (4.1)	-972.70686 (4.0)

	C -1.62173 -1.16823 0.22286<br>C -2.28993 -0.02465 0.47245<br>C -1.82280 1.26995 -0.01515<br>C -0.70827 1.35168 -0.76152<br>C 0.06901 0.14052 -1.17761<br>H -3.19686 -0.06128 1.06984<br>H -0.35843 2.30637 -1.14209<br>H -0.07745 -0.00085 -2.25950<br>O 0.21043 -2.23579 -0.74384<br>O -2.01425 -2.37020 0.69659<br>C -2.64086 2.47817 0.35138<br>H -3.66019 2.38413 -0.03804<br>H -2.71746 2.57892 1.43917<br>H -2.20024 3.39270 -0.05111<br>S 1.87799 0.37842 -1.06146<br>C 2.08460 0.26936 0.70720<br>C 3.50397 0.44691 1.17507<br>H 3.94457 -0.54558 1.31296<br>H 4.11297 1.00235 0.46016<br>H 3.49678 0.95834 2.13909<br>O 1.15237 0.03306 1.44607<br>H -1.36393 -3.02031 0.38244						
5-adduct, conf2	C 0.44267 1.21878 -0.50567<br>C 1.72587 1.09851 0.20262<br>C 2.31255 -0.10170 0.39367<br>C 1.73564 -1.34419 -0.11117<br>C 0.58177 -1.32153 -0.80143<br>C -0.12224 -0.04274 -1.13695<br>H 3.24276 -0.14965 0.95296<br>H 0.14479 -2.23349 -1.19555<br>H 0.02129 0.12834 -2.21634<br>O -0.09481 2.31667 -0.59525<br>O 2.23291 2.24698 0.69579<br>C 2.48147 -2.61704 0.17944<br>H 3.48548 -2.58005 -0.25589<br>H 2.60017 -2.75677 1.25892<br>H 1.95979 -3.48525 -0.22793<br>S -1.95096 -0.15267 -1.05207<br>C -2.37836 -0.31321 0.68127<br>C -1.32794 -0.18015 1.74686<br>H -0.43601 -0.76633 1.51412<br>H -1.04266 0.87278 1.83895<br>H -1.75321 -0.51318 2.69396	C_1	-972.66541 (6.2)	-972.47478 (6.1)	-972.52842 (6.6)	-972.83997 (5.9)	-972.70298 (6.4)

	O -3.55571 -0.49050 0.91651<br>H 1.62528 2.96075 0.44163						
5-adduct, conf3	C 0.53996 1.12133 -0.09735<br>C 2.00256 1.17691 0.03282<br>C 2.74496 0.05063 0.05318<br>C 2.15312 -1.27803 -0.08779<br>C 0.82354 -1.41473 -0.23168<br>C -0.10032 -0.24003 -0.28365<br>H 3.82112 0.13118 0.17972<br>H 0.37050 -2.39406 -0.35042<br>H -0.58618 -0.19709 -1.27377<br>O -0.10635 2.16415 -0.07391<br>O 2.53310 2.41014 0.17396<br>C 3.09153 -2.45318 -0.06775<br>H 3.83492 -2.36885 -0.86737<br>H 3.63863 -2.49109 0.88032<br>H 2.55327 -3.39484 -0.19363<br>S -1.51026 -0.45322 0.85994<br>C -2.88141 -0.18561 -0.25757<br>C -4.22823 -0.29783 0.40432<br>H -4.78930 -1.09582 -0.08894<br>H -4.15710 -0.50752 1.47239<br>H -4.76486 0.64215 0.25215<br>O -2.73006 0.05194 -1.43650<br>H 1.79540 3.04094 0.13502	C_1	-972.66858 (4.2)	-972.47783 (4.2)	-972.53389 (3.2)	-972.84130 (5.0)	-972.70662 (4.1)
5-adduct, conf4	C -0.37306 1.06051 0.13687<br>C -1.79893 1.29589 -0.12700<br>C -2.68313 0.27774 -0.13338<br>C -2.28744 -1.10154 0.14886<br>C -1.00253 -1.40000 0.40557<br>C 0.07475 -0.36003 0.43153<br>H -3.72730 0.48479 -0.35075<br>H -0.69901 -2.41789 0.62949<br>H 0.51864 -0.34507 1.43395<br>O 0.40653 2.00778 0.11502<br>O -2.14454 2.57321 -0.38933<br>C -3.37396 -2.14071 0.14751<br>H -4.13465 -1.90388 0.89850<br>H -3.87584 -2.16988 -0.82533<br>H -2.97274 -3.13367 0.35988<br>S 1.39942 -0.87082 -0.74219<br>C 2.95723 -0.29484 -0.06205<br>C 3.01821 0.34714 1.29418	C_1	-972.66416 (7.0)	-972.47373 (6.8)	-972.52903 (6.2)	-972.83807 (7.1)	-972.70294 (6.4)

	H 2.36781 1.22423 1.33073<br>H 2.69339 -0.36057 2.06298<br>H 4.05104 0.63677 1.48839<br>O 3.92999 -0.49254 -0.76040<br>H -1.33460 3.10572 -0.32770						
5-adduct, conf5	C 0.84287 -1.19820 0.28227<br>C 2.24864 -0.86434 0.01822<br>C 2.64604 0.41613 -0.12707<br>C 1.71800 1.54037 -0.01496<br>C 0.41371 1.32067 0.22163<br>C -0.15630 -0.05376 0.37660<br>H 3.69371 0.62347 -0.32668<br>H -0.28102 2.15075 0.30759<br>H -0.61293 -0.16520 1.36615<br>O 0.51887 -2.37108 0.43015<br>O 3.09377 -1.91213 -0.07422<br>C 2.29368 2.92068 -0.17116<br>H 3.06403 3.10464 0.58506<br>H 2.76893 3.03025 -1.15169<br>H 1.52183 3.68680 -0.07329<br>S -1.48934 -0.37375 -0.86242<br>C -3.02687 0.09879 -0.07532<br>C -3.05217 0.57539 1.35051<br>H -2.77044 -0.23753 2.02660<br>H -2.36402 1.40883 1.50977<br>H -4.06842 0.89377 1.58280<br>O -4.01977 -0.00863 -0.76452<br>H 2.56920 -2.71545 0.07677	C_1	-972.66203 (8.3)	-972.47127 (8.3)	-972.52664 (7.7)	-972.83713 (7.7)	-972.70174 (7.2)

Table S13.3. Reduced zwitterionic form in water.

Structure	Schematic drawing	Symmetry	G_{PCM}	$H_{PCM,RRHO}$	$G_{PCM,RRHO}$	$G_{SMD,\omega B97X\text{-}D,large}$	$G_{SMD,RRHO,\omega B97X\text{-}D,large}$
6-adduct, several starting structures	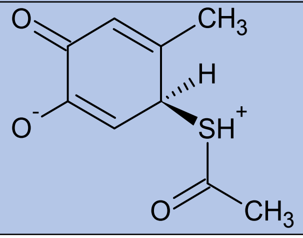	C_1	dissociates to neutral molecules				

	(3*S*)-3-(acetylsulfonio)-4-methyl-6-oxocyclohexa-1,4-dien-1-olate						
5-adduct, several starting structures	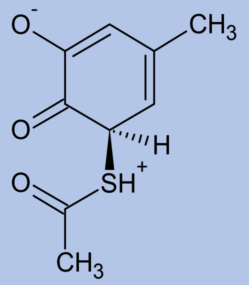<br>(5*R*)-5-(acetylsulfonio)-3-methyl-6-oxocyclohexa-1,3-dien-1-olate	C_1	most starting structures dissociate to neutral molecules				
5-adduct, conf1	C 0.17803 -1.24395 0.55568<br>C 1.44355 -1.51503 -0.23088<br>C 2.20514 -0.38685 -0.51241<br>C 1.96311 0.93815 0.01273<br>C 0.91617 1.22548 0.82071<br>C 0.00766 0.12961 1.23129<br>H 3.06611 -0.51990 -1.16376<br>H 0.78902 2.20043 1.27783<br>H 0.08932 -0.05843 2.31149<br>O -0.71849 -2.06948 0.65812<br>O 1.63842 -2.69752 -0.63833<br>C 2.96684 2.00358 -0.34246<br>H 3.96301 1.72635 0.01830<br>H 3.03750 2.11253 -1.42991<br>H 2.69924 2.97134 0.08746<br>S -1.78795 0.63118 1.09064<br>C -1.93873 0.72008 -0.84657<br>C -2.79049 -0.34859 -1.43137<br>H -2.34059 -1.32389 -1.22066<br>H -3.78166 -0.32445 -0.97032<br>H -2.86681 -0.18506 -2.50589<br>O -1.35503 1.61524 -1.35274<br>H -2.27479 -0.61858 1.26844	C_1	-972.58118 (0.8)	-972.39482 (0.8)	-972.45085 (0.9)	-972.76326 (0.4)	-972.63293 (0.4)
5-adduct, conf2	C 0.18264 1.26136 -0.51899<br>C 1.46932 1.48632 0.25490<br>C 2.22846 0.34191 0.47613<br>C 1.96592 -0.95945 -0.09521<br>C 0.89155 -1.21064 -0.87868<br>C -0.01982 -0.08970 -1.22898<br>H 3.10741 0.44570 1.10825	C_1	-972.58250 (0.0)	-972.39605 (0.0)	-972.45221 (0.0)	-972.76390 (0.0)	-972.63360 (0.0)

	H 0.74136 -2.16838 -1.36485<br>H 0.04062 0.13438 -2.30451<br>O -0.69818 2.10369 -0.58801<br>O 1.68496 2.64928 0.70155<br>C 2.96742 -2.04542 0.19743<br>H 3.95490 -1.76624 -0.18483<br>H 3.06915 -2.18787 1.27851<br>H 2.67663 -2.99660 -0.25381<br>S -1.79468 -0.60726 -1.06493<br>C -2.18487 -0.48509 0.84420<br>C -1.16088 -1.10719 1.71958<br>H -0.99873 -2.14477 1.41724<br>H -0.20672 -0.57891 1.61883<br>H -1.51514 -1.05285 2.74866<br>O -3.21637 0.03340 1.09786<br>H -2.35642 0.58820 -1.33795						
5-adduct, conf3	C -0.91665 -1.30196 -0.36576<br>C -2.06784 -0.61001 0.35472<br>C -2.02872 0.78091 0.34248<br>C -1.07986 1.57330 -0.40096<br>C -0.06245 1.02777 -1.11385<br>C 0.07479 -0.44446 -1.18113<br>H -2.79621 1.30659 0.90541<br>H 0.59920 1.63441 -1.72306<br>H 0.03121 -0.82650 -2.20887<br>O -0.73953 -2.50455 -0.30249<br>O -2.90096 -1.34895 0.94887<br>C -1.27083 3.06674 -0.37280<br>H -2.24489 3.33379 -0.79549<br>H -1.25685 3.43012 0.66025<br>H -0.49174 3.58596 -0.93521<br>S 1.78868 -1.02279 -0.65699<br>C 2.16015 0.27678 0.76330<br>C 1.26891 0.10368 1.93684<br>H 0.22618 0.28022 1.64950<br>H 1.35650 -0.91913 2.31482<br>H 1.56609 0.81958 2.70267<br>O 3.06943 1.00717 0.57175<br>H 2.49512 -0.34618 -1.58033	C_1	-972.58213 (0.2)	-972.39550 (0.4)	-972.45159 (0.4)	-972.76298 (0.6)	-972.63244 (0.7)
5-adduct, conf4	C 0.49469 1.27235 -0.34360<br>C 1.85698 1.18111 0.31196<br>C 2.36656 -0.10666 0.42352<br>C 1.75660 -1.30115 -0.11723	C_1	-972.58224 (0.2)	-972.39608 (0.0)	-972.45117 (0.6)	-972.76384 (0.0)	-972.63277 (0.5)

	C 0.61133 -1.27122 -0.83489<br>C 0.00532 0.05413 -1.14329<br>H 3.29840 -0.22301 0.97287<br>H 0.19965 -2.15494 -1.31013<br>H 0.21334 0.31934 -2.19297<br>O -0.20175 2.27661 -0.29518<br>O 2.36091 2.24990 0.77123<br>C 2.47652 -2.60266 0.12508<br>H 3.47760 -2.57376 -0.31811<br>H 2.60507 -2.77273 1.19933<br>H 1.93466 -3.45113 -0.29896<br>S -1.83016 0.02351 -1.11805<br>C -2.12677 -0.33398 0.79325<br>C -3.54144 -0.69358 1.05703<br>H -4.20545 0.06815 0.64049<br>H -3.76028 -1.64116 0.55547<br>H -3.69020 -0.78887 2.13198<br>O -1.19117 -0.24186 1.50550<br>H -1.93296 1.37470 -0.97465						
5-adduct, conf5	C -0.22314 0.93605 0.01116<br>C -1.59906 1.56509 0.04392<br>C -2.64141 0.64855 -0.03252<br>C -2.49459 -0.78975 0.02779<br>C -1.29474 -1.39810 0.18273<br>C -0.08982 -0.54613 0.35501<br>H -3.64705 1.04864 -0.14183<br>H -1.20337 -2.46800 0.33435<br>H 0.28209 -0.58908 1.39077<br>O 0.78721 1.55741 -0.29471<br>O -1.67552 2.82891 0.02156<br>C -3.76048 -1.60363 -0.04351<br>H -4.44961 -1.31310 0.75616<br>H -4.27414 -1.41927 -0.99326<br>H -3.56046 -2.67415 0.04026<br>S 1.34272 -1.12046 -0.67003<br>C 2.79871 -0.23915 0.34083<br>C 3.82352 0.31994 -0.57239<br>H 4.28119 -0.49686 -1.13836<br>H 3.33878 0.99849 -1.27799<br>H 4.57599 0.84293 0.01693<br>O 2.70899 -0.26518 1.51681<br>H 1.49366 -2.32864 -0.09391	C_1	-972.58193 (0.4)	-972.39563 (0.3)	-972.45146 (0.5)	-972.76269 (0.8)	-972.63222 (0.9)

5-adduct, conf6	C -0.48067 1.07725 -0.05629<br>C -1.96350 1.39787 -0.01907<br>C -2.78937 0.27948 -0.00110<br>C -2.33344 -1.08241 0.15374<br>C -1.02643 -1.41230 0.29989<br>C -0.02131 -0.32084 0.36297<br>H -3.85937 0.44603 -0.10154<br>H -0.71357 -2.42386 0.53609<br>H 0.39641 -0.22504 1.37432<br>O 0.35935 1.88030 -0.43331<br>O -2.30582 2.61107 -0.12067<br>C -3.39345 -2.15189 0.20220<br>H -4.09212 -1.96159 1.02346<br>H -3.97712 -2.14632 -0.72461<br>H -2.96063 -3.14595 0.33434<br>S 1.42713 -0.69639 -0.75468<br>C 3.11840 -0.25716 0.12378<br>C 3.09335 0.96645 0.95953<br>H 2.55507 1.76508 0.44475<br>H 2.56515 0.74972 1.89382<br>H 4.12202 1.24901 1.18247<br>O 3.99252 -1.00824 -0.14076<br>H 1.54023 -2.00853 -0.48419	C_1	-972.57900 (2.2)	-972.39236 (2.3)	-972.44965 (1.6)	-972.76085 (1.9)	-972.63150 (1.3)
5-adduct, conf7	C 0.83430 -1.25378 0.15414<br>C 2.33602 -1.04713 0.21807<br>C 2.75524 0.26422 0.03522<br>C 1.88314 1.41317 -0.06160<br>C 0.53595 1.31730 0.01731<br>C -0.07920 -0.01156 0.26542<br>H 3.82689 0.43792 -0.03071<br>H -0.09833 2.19718 0.02513<br>H -0.49630 -0.08012 1.28016<br>O 0.32681 -2.35807 0.03781<br>O 3.05918 -2.07847 0.33904<br>C 2.54472 2.75667 -0.22433<br>H 3.21777 2.95703 0.61588<br>H 3.15376 2.77053 -1.13459<br>H 1.81170 3.56422 -0.28396<br>S -1.50899 -0.33091 -0.87812<br>C -3.10041 0.13699 0.13597<br>C -3.05351 1.51221 0.69705<br>H -2.24298 1.59742 1.42609<br>H -2.86872 2.22897 -0.10808	C_1	-972.57809 (2.8)	-972.39166 (2.8)	-972.44753 (2.9)	-972.76056 (2.1)	-972.63000 (2.3)

	H -4.00688 1.72134 1.18109<br>O -3.95010 -0.68440 0.15901<br>H -1.57764 -1.66089 -0.63149						
5-adduct, conf8	C 0.81439 -1.24830 0.14695<br>C 2.31820 -1.07182 0.23409<br>C 2.76594 0.22954 0.04702<br>C 1.91844 1.39505 -0.07416<br>C 0.56890 1.32675 -0.01176<br>C -0.07368 0.01283 0.24747<br>H 3.84160 0.38164 -0.00574<br>H -0.04921 2.21811 -0.02081<br>H -0.48109 -0.03862 1.26740<br>O 0.28619 -2.34223 0.02281<br>O 3.01860 -2.11688 0.37285<br>C 2.60866 2.72344 -0.24232<br>H 3.26668 2.92401 0.60970<br>H 3.23765 2.71305 -1.13893<br>H 1.89200 3.54314 -0.32936<br>S -1.51476 -0.28845 -0.88257<br>C -3.11172 0.14958 0.13602<br>C -3.04576 1.47722 0.80012<br>H -2.27226 1.47497 1.57270<br>H -2.79335 2.24473 0.06390<br>H -4.01450 1.68592 1.25278<br>O -3.98038 -0.65008 0.07965<br>H -1.56680 -1.62402 -0.65349	C_1	-972.57804 (2.8)	-972.39174 (2.7)	-972.44842 (2.4)	-972.76056 (2.1)	-972.63094 (1.7)

Table S13.4. Reduced anionic form in water.

Structure	Schematic drawing	Symmetry	G_{PCM}	$H_{PCM,RRHO}$	$G_{PCM,RRHO}$	$G_{SMD,\omega B97X\text{-}D,large}$	$G_{SMD,RRHO,\omega B97X\text{-}D,large}$
6-adduct, conf1	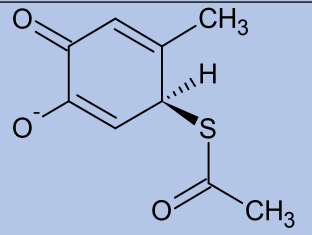<br>(3*S*)-3-(acetylsulfanyl)-4-methyl-6-oxocyclohexa-1,4-dien-1-olate	C_1	-972.19155 (0.0)	-972.01431 (0.1)	-972.06820 (1.3)	-972.37169 (0.0)	-972.24834 (0.6)

	C 2.29714 -1.03794 0.22828<br>C 2.71329 0.36136 -0.19742<br>C 1.71373 1.43972 -0.18462<br>C 0.41748 1.25302 0.11782<br>C -0.06894 -0.12372 0.47615<br>C 0.96712 -1.19185 0.54586<br>H 2.08817 2.43099 -0.43143<br>H -0.63479 -0.04832 1.41461<br>H 0.62065 -2.17284 0.86416<br>O 3.20058 -1.94529 0.27476<br>O 3.87374 0.61011 -0.53238<br>C -0.56507 2.38681 0.14677<br>H -1.06113 2.44004 1.12113<br>H -0.06977 3.33973 -0.04980<br>H -1.35011 2.24491 -0.60385<br>S -1.38636 -0.64896 -0.73591<br>C -2.88830 -0.27370 0.13872<br>C -4.14326 -0.61738 -0.62381<br>H -4.67912 0.31286 -0.83329<br>H -3.94073 -1.13367 -1.56324<br>H -4.77862 -1.24056 0.01054<br>O -2.90832 0.21422 1.25252						
6-adduct, conf2	C -2.26122 -1.02866 -0.28406<br>C -2.68441 0.34931 0.20452<br>C -1.69832 1.44217 0.20091<br>C -0.40883 1.28089 -0.13852<br>C 0.09508 -0.08772 -0.51162<br>C -0.93579 -1.15490 -0.63257<br>H -2.08067 2.42168 0.47968<br>H 0.67564 -0.00545 -1.43605<br>H -0.58185 -2.12084 -0.98600<br>O -3.15545 -1.94173 -0.35173<br>O -3.83716 0.57120 0.57806<br>C 0.55105 2.43311 -0.18459<br>H 0.90527 2.59276 -1.20954<br>H 0.07694 3.35451 0.15870<br>H 1.42946 2.24481 0.44170<br>S 1.33466 -0.63703 0.79430<br>C 2.98459 -0.39730 0.16469<br>C 3.21584 0.08057 -1.24431<br>H 2.79630 -0.63074 -1.96190<br>H 2.74918 1.05293 -1.41647<br>H 4.29095 0.16319 -1.40572	C_1	-972.18697 (2.9)	-972.00994 (2.9)	-972.06412 (3.8)	-972.36848 (2.0)	-972.24563 (2.3)

	O 3.89714 -0.67763 0.92119						
6-adduct, conf3	C -2.30180 -1.03246 -0.22746<br>C -2.71108 0.36832 0.19979<br>C -1.70634 1.44188 0.18752<br>C -0.41143 1.24979 -0.11733<br>C 0.06773 -0.12818 -0.48043<br>C -0.97355 -1.19132 -0.54994<br>H -2.07564 2.43443 0.43701<br>H 0.63159 -0.05299 -1.42004<br>H -0.63219 -2.17340 -0.87039<br>O -3.20879 -1.93642 -0.27163<br>O -3.87013 0.62242 0.53570<br>C 0.57612 2.37921 -0.14544<br>H 1.07024 2.43281 -1.12073<br>H 0.08537 3.33372 0.05479<br>H 1.36223 2.23174 0.60302<br>S 1.38450 -0.66725 0.72657<br>C 2.88790 -0.27687 -0.14121<br>C 4.14001 -0.60712 0.63130<br>H 4.52573 0.32016 1.06661<br>H 3.95993 -1.32252 1.43537<br>H 4.88910 -0.99939 -0.05958<br>O 2.90721 0.22050 -1.25058	C_1	-972.19152 (0.0)	-972.01453 (0.0)	-972.06922 (0.6)	-972.37162 (0.0)	-972.24932 (0.0)
6-adduct, conf4	C -1.85419 -0.95744 -0.57177<br>C -2.21623 0.03796 0.51814<br>C -1.44519 1.28290 0.63607<br>C -0.33280 1.54052 -0.07000<br>C 0.11286 0.56169 -1.12028<br>C -0.74312 -0.64024 -1.31669<br>H -1.80847 1.99408 1.37511<br>H 0.18632 1.12798 -2.06073<br>H -0.44367 -1.31381 -2.11688<br>O -2.60141 -1.99032 -0.72128<br>O -3.15275 -0.16989 1.29473<br>C 0.45597 2.80381 0.11817<br>H 0.66829 3.28207 -0.84382<br>H -0.08788 3.51010 0.74887<br>H 1.42116 2.59310 0.59117<br>S 1.91744 0.11072 -0.93289<br>C 1.99794 -0.72578 0.63541<br>C 3.34588 -1.34714 0.91276<br>H 3.26100 -2.42577 0.74675<br>H 4.13233 -0.95450 0.26597	C_1	-972.18787 (2.3)	-972.01096 (2.2)	-972.06561 (2.9)	-972.36944 (1.4)	-972.24718 (1.3)

	H 3.60385 -1.17993 1.96059<br>O 1.06183 -0.80762 1.40259						
6-adduct, conf5	C -1.88481 -0.95829 -0.52786<br>C -2.27963 0.15649 0.42753<br>C -1.41954 1.34414 0.53537<br>C -0.26135 1.49764 -0.12827<br>C 0.16771 0.43941 -1.10612<br>C -0.70847 -0.76070 -1.21508<br>H -1.77227 2.11753 1.21425<br>H 0.23170 0.94505 -2.08233<br>H -0.39228 -1.51746 -1.92934<br>O -2.65940 -1.97405 -0.62242<br>O -3.30706 0.08730 1.10559<br>C 0.58858 2.72444 0.03193<br>H 0.88068 3.13014 -0.94213<br>H 0.05384 3.49690 0.58824<br>H 1.51425 2.49163 0.56944<br>S 1.98280 -0.00298 -0.95419<br>C 2.26195 -0.78099 0.62758<br>C 1.15610 -0.87608 1.63708<br>H 0.94962 0.11871 2.04389<br>H 0.23835 -1.24262 1.17111<br>H 1.47259 -1.53954 2.44270<br>O 3.38838 -1.19688 0.82911	C_1	-972.18883 (1.7)	-972.01206 (1.5)	-972.06660 (2.3)	-972.36900 (1.7)	-972.24677 (1.6)
6-adduct, conf6	C 1.92267 -1.27631 0.13806<br>C 2.68989 -0.00802 -0.20059<br>C 2.01864 1.29400 -0.05569<br>C 0.71962 1.43221 0.25954<br>C -0.11804 0.20382 0.48106<br>C 0.60191 -1.09647 0.47943<br>H 2.64775 2.16812 -0.20979<br>H -0.69169 0.33057 1.40793<br>H 0.00597 -1.97202 0.72849<br>O 2.55448 -2.39049 0.09629<br>O 3.86583 -0.04794 -0.56908<br>C 0.07713 2.77737 0.43927<br>H -0.38816 2.84942 1.42837<br>H 0.81300 3.57800 0.34096<br>H -0.71490 2.94012 -0.29888<br>S -1.46980 0.22133 -0.81600<br>C -2.83354 -0.44018 0.10898<br>C -4.10485 -0.58042 -0.69029<br>H -4.82985 0.14575 -0.31094	C_1	-972.19038 (0.7)	-972.01339 (0.7)	-972.06889 (0.8)	-972.37017 (1.0)	-972.24868 (0.4)

	H -3.95323 -0.40925 -1.75701<br>H -4.51152 -1.58193 -0.53089<br>O -2.75795 -0.73984 1.28519						
6-adduct, conf7	C 1.86732 -1.29187 0.14221<br>C 2.65609 -0.03542 -0.19514<br>C 2.01377 1.27770 -0.02095<br>C 0.72155 1.43634 0.30939<br>C -0.14655 0.22030 0.50166<br>C 0.55122 -1.09091 0.49275<br>H 2.66031 2.14119 -0.16137<br>H -0.71194 0.36881 1.42777<br>H -0.05008 -1.96636 0.72938<br>O 2.47771 -2.41532 0.08914<br>O 3.82338 -0.09531 -0.58421<br>C 0.10943 2.79023 0.52551<br>H -0.35475 2.84585 1.51623<br>H 0.86390 3.57573 0.44988<br>H -0.67766 2.99189 -0.20802<br>S -1.45273 0.32093 -0.85715<br>C -2.92838 -0.42207 -0.19730<br>C -2.99065 -0.86169 1.24178<br>H -2.18074 -1.55321 1.48458<br>H -2.90877 0.00392 1.90587<br>H -3.95060 -1.35084 1.40862<br>O -3.87219 -0.53031 -0.95967	C_1	-972.18679 (3.0)	-972.00963 (3.1)	-972.06397 (3.9)	-972.36745 (2.7)	-972.24463 (2.9)
6-adduct, conf8	C 2.28717 -1.04643 0.23548<br>C 2.71517 0.34705 -0.19719<br>C 1.72521 1.43430 -0.18861<br>C 0.42734 1.25978 0.11411<br>C -0.07142 -0.11173 0.47554<br>C 0.95568 -1.18780 0.55276<br>H 2.10852 2.42133 -0.43869<br>H -0.64068 -0.02825 1.41115<br>H 0.60080 -2.16444 0.87518<br>O 3.18330 -1.96070 0.28747<br>O 3.87753 0.58394 -0.53398<br>C -0.54578 2.40175 0.13922<br>H -1.04425 2.45955 1.11213<br>H -0.04231 3.35047 -0.05696<br>H -1.33007 2.26523 -0.61316<br>S -1.38809 -0.62838 -0.74192<br>C -2.89138 -0.25617 0.13234<br>C -4.14213 -0.66459 -0.60484	C_1	-972.19149 (0.0)	-972.01424 (0.2)	-972.06859 (1.0)	-972.37169 (0.0)	-972.24879 (0.3)

	H -4.87903 0.13706 -0.52015<br>H -3.95673 -0.88887 -1.65640<br>H -4.54970 -1.55626 -0.11858<br>O -2.91288 0.25192 1.23693						
5-adduct, conf1	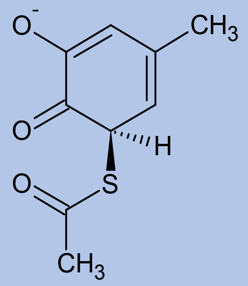<br>(5*R*)-5-(acetylsulfanyl)-3-methyl-6-oxocyclohexa-1,3-dien-1-olate<br><br>C -0.40205 -1.22524 -0.51986<br>C -1.70599 -1.28029 0.26544<br>C -2.35130 -0.06945 0.46034<br>C -1.87075 1.21194 -0.01304<br>C -0.73649 1.32774 -0.73429<br>C 0.03218 0.11160 -1.14003<br>H -3.27612 -0.08329 1.03523<br>H -0.39222 2.28711 -1.10912<br>H -0.11497 -0.05270 -2.21953<br>O 0.25963 -2.23349 -0.72249<br>O -2.08180 -2.41791 0.70182<br>C -2.70228 2.42288 0.32971<br>H -3.71062 2.32800 -0.08917<br>H -2.81533 2.52220 1.41515<br>H -2.25461 3.34236 -0.05647<br>S 1.84758 0.35531 -1.05884<br>C 2.13228 0.26963 0.69352<br>C 3.57428 0.46416 1.09000<br>H 4.02144 -0.52208 1.24973<br>H 4.15134 0.98909 0.32692<br>H 3.61127 1.01460 2.03206<br>O 1.24710 0.03790 1.49141	C_1	-972.18848 (1.9)	-972.01198 (1.6)	-972.06678 (2.2)	-972.36710 (2.9)	-972.24540 (2.5)
5-adduct, conf2	C 0.43433 1.27808 -0.49813<br>C 1.74322 1.22133 0.27649<br>C 2.31158 -0.03630 0.41674<br>C 1.76412 -1.26121 -0.12728<br>C 0.61359 -1.27880 -0.83391	C_1	-972.18795 (2.3)	-972.01146 (1.9)	-972.06561 (2.9)	-972.36556 (3.8)	-972.24322 (3.8)

	C -0.10321 -0.00609 -1.14804<br>H 3.23472 -0.10545 0.98979<br>H 0.21498 -2.19536 -1.25775<br>H 0.01032 0.19804 -2.22461<br>O -0.17210 2.33019 -0.63854<br>O 2.18332 2.31224 0.76315<br>C 2.53653 -2.53037 0.12934<br>H 3.53989 -2.46312 -0.30584<br>H 2.66416 -2.69509 1.20497<br>H 2.03412 -3.40212 -0.29745<br>S -1.93600 -0.15214 -1.03821<br>C -2.33198 -0.35170 0.69098<br>C -1.26471 -0.20822 1.73898<br>H -0.36079 -0.76038 1.47049<br>H -1.00477 0.85047 1.84075<br>H -1.66068 -0.56900 2.68906<br>O -3.50082 -0.56544 0.95425						
5-adduct, conf3	C 0.54588 1.16667 -0.11172<br>C 2.04988 1.30803 0.06356<br>C 2.76682 0.12148 0.08736<br>C 2.19465 -1.19944 -0.08647<br>C 0.87085 -1.38762 -0.26915<br>C -0.06361 -0.22483 -0.32262<br>H 3.84272 0.19080 0.24084<br>H 0.44908 -2.37679 -0.42085<br>H -0.55544 -0.16754 -1.30635<br>O -0.18752 2.14606 -0.10421<br>O 2.51950 2.48402 0.20922<br>C 3.15050 -2.36552 -0.06700<br>H 3.90656 -2.26086 -0.85327<br>H 3.68664 -2.40722 0.88794<br>H 2.63150 -3.31657 -0.21174<br>S -1.47174 -0.47826 0.82609<br>C -2.87070 -0.19863 -0.23932<br>C -4.19864 -0.31221 0.46437<br>H -4.87203 -0.91366 -0.15020<br>H -4.11058 -0.75188 1.45908<br>H -4.62361 0.69182 0.55640<br>O -2.76708 0.05947 -1.42168	C_1	-972.18921 (1.5)	-972.01247 (1.3)	-972.06702 (2.0)	-972.36624 (3.4)	-972.24404 (3.3)
5-adduct, conf4	C -0.31665 1.10654 0.46786<br>C -1.54997 1.42386 -0.37208<br>C -2.48248 0.39968 -0.44406<br>C -2.30556 -0.91001 0.15353	C_1	-972.18686 (2.9)	-972.01006 (2.8)	-972.06610 (2.6)	-972.36343 (5.2)	-972.24268 (4.2)

	C -1.14405 -1.28592 0.73613<br>C 0.02239 -0.35701 0.74273<br>H -3.42087 0.60242 -0.95725<br>H -1.00436 -2.27661 1.15726<br>H 0.60188 -0.42076 1.66579<br>O 0.40405 1.99132 0.90786<br>O -1.64974 2.59231 -0.86460<br>C -3.48202 -1.85034 0.09470<br>H -4.35706 -1.40565 0.58128<br>H -3.76077 -2.04692 -0.94673<br>H -3.26280 -2.80511 0.57930<br>S 1.13896 -0.90841 -0.65426<br>C 2.81140 -0.39591 -0.27557<br>C 3.13623 0.22272 1.05383<br>H 2.50215 1.09274 1.24076<br>H 2.95395 -0.50399 1.85224<br>H 4.18960 0.50501 1.05736<br>O 3.64559 -0.62467 -1.13127						
5-adduct, conf5	C 1.00192 -1.23417 0.48637<br>C 2.27691 -0.85809 -0.24961<br>C 2.48287 0.50522 -0.40715<br>C 1.56756 1.53813 0.03948<br>C 0.35609 1.24319 0.56069<br>C -0.08845 -0.17322 0.65832<br>H 3.41679 0.82141 -0.86916<br>H -0.34166 2.01364 0.87277<br>H -0.61898 -0.38286 1.59168<br>O 0.81510 -2.36235 0.92033<br>O 3.06014 -1.79750 -0.60551<br>C 2.02524 2.96628 -0.11336<br>H 2.96235 3.13431 0.42891<br>H 2.22168 3.19373 -1.16742<br>H 1.27807 3.67249 0.25777<br>S -1.32987 -0.57474 -0.67168<br>C -2.80187 0.11663 0.05152<br>C -4.00608 0.05557 -0.85339<br>H -4.13752 1.04155 -1.31023<br>H -3.89599 -0.68469 -1.64772<br>H -4.89089 -0.16757 -0.25410<br>O -2.82538 0.60942 1.16107	C_1	-972.18851 (1.9)	-972.01153 (1.9)	-972.06614 (2.6)	-972.36538 (4.0)	-972.24301 (4.0)
5-adduct, conf6	C 0.94055 -1.22192 0.51428<br>C 2.25206 -0.90216 -0.18521<br>C 2.50520 0.45021 -0.36519	C_1	-972.18453 (4.4)	-972.00770 (4.3)	-972.06255 (4.8)	-972.36244 (5.8)	-972.24045 (5.6)

	C 1.61003 1.51849 0.03274<br>C 0.37355 1.27408 0.52141<br>C -0.12592 -0.12348 0.62721<br>H 3.46260 0.72777 -0.80277<br>H -0.29556 2.08420 0.79514<br>H -0.66952 -0.31724 1.55473<br>O 0.70243 -2.33191 0.96676<br>O 3.01572 -1.87260 -0.49313<br>C 2.11698 2.92823 -0.13334<br>H 3.05034 3.07355 0.42145<br>H 2.33698 3.13195 -1.18751<br>H 1.38756 3.66416 0.21431<br>S -1.31321 -0.53665 -0.76277<br>C -2.90790 0.07104 -0.24431<br>C -3.10517 0.63953 1.13454<br>H -2.96406 -0.14389 1.88547<br>H -2.39555 1.44202 1.34478<br>H -4.12281 1.02421 1.20531<br>O -3.81000 -0.03356 -1.05356						
5-adduct, conf7	C -0.55427 1.16460 0.03617<br>C -2.06816 1.30155 -0.02114<br>C -2.77925 0.11107 -0.02964<br>C -2.19191 -1.20939 0.08235<br>C -0.86031 -1.39145 0.20463<br>C 0.06434 -0.21987 0.26188<br>H -3.86220 0.17710 -0.12385<br>H -0.42626 -2.37982 0.32255<br>H 0.52257 -0.14646 1.26236<br>O 0.17412 2.14232 -0.06586<br>O -2.55237 2.47710 -0.10996<br>C -3.13997 -2.38198 0.07172<br>H -3.87051 -2.29826 0.88410<br>H -3.70711 -2.40870 -0.86576<br>H -2.61031 -3.33194 0.18038<br>S 1.50848 -0.47260 -0.83494<br>C 2.86795 -0.17664 0.27398<br>C 4.22218 -0.32504 -0.37135<br>H 4.76627 -1.12110 0.14406<br>H 4.15963 -0.55669 -1.43558<br>H 4.77304 0.60899 -0.23362<br>O 2.72271 0.10326 1.44740	C_1	-972.18915 (1.5)	-972.01262 (1.2)	-972.07022 (0.0)	-972.36612 (3.5)	-972.24719 (1.3)
5-adduct, conf8	C -0.38611 1.11038 0.13295<br>C -1.84848 1.41251 -0.15617	C_1	-972.18626 (3.3)	-972.00964 (3.1)	-972.06405 (3.9)	-972.36344 (5.2)	-972.24123 (5.1)

	C -2.70013 0.31859 -0.15428<br>C -2.30099 -1.04251 0.14309<br>C -1.02143 -1.37160 0.42042<br>C 0.04798 -0.32901 0.44464<br>H -3.74965 0.49972 -0.38018<br>H -0.73448 -2.38982 0.66547<br>H 0.50588 -0.29279 1.44007<br>O 0.45717 1.99721 0.12487<br>O -2.16284 2.62188 -0.40093<br>C -3.38872 -2.08633 0.14852<br>H -4.15431 -1.84188 0.89313<br>H -3.88981 -2.12452 -0.82517<br>H -2.99284 -3.08029 0.37174<br>S 1.37512 -0.85407 -0.72826<br>C 2.94062 -0.28982 -0.07756<br>C 3.02719 0.33634 1.28601<br>H 2.38802 1.22097 1.33414<br>H 2.69402 -0.37337 2.04955<br>H 4.06641 0.60660 1.47550<br>O 3.91133 -0.48295 -0.78616						
5-adduct, conf9	C 0.91470 -1.26265 0.13299<br>C 2.39473 -0.90570 0.15813<br>C 2.69045 0.43961 0.00217<br>C 1.70503 1.49625 -0.10612<br>C 0.38065 1.25241 -0.01808<br>C -0.12099 -0.13153 0.24156<br>H 3.74165 0.71799 -0.05196<br>H -0.34669 2.05633 -0.07955<br>H -0.44555 -0.21903 1.28927<br>O 0.54699 -2.42675 0.08141<br>O 3.22697 -1.86410 0.25563<br>C 2.22683 2.89576 -0.31201<br>H 2.89990 3.18090 0.50411<br>H 2.80599 2.95714 -1.24026<br>H 1.41638 3.62738 -0.36247<br>S -1.58209 -0.57433 -0.79021<br>C -2.99514 0.13127 0.03104<br>C -2.85686 0.77652 1.38455<br>H -2.55455 0.03168 2.12693<br>H -2.10663 1.57020 1.37817<br>H -3.82482 1.19110 1.66673<br>O -4.06254 0.03136 -0.54617	C_1	-972.18386 (4.8)	-972.00716 (4.6)	-972.06169 (5.4)	-972.36208 (6.0)	-972.23991 (5.9)

5-adduct, conf10	C 0.55684 -1.17069 0.11632<br>C 2.06152 -1.29814 -0.06315<br>C 2.76730 -0.10498 -0.08795<br>C 2.18324 1.21072 0.08584<br>C 0.85768 1.38714 0.26759<br>C -0.06605 0.21595 0.32121<br>H 3.84373 -0.16408 -0.24211<br>H 0.42691 2.37251 0.41871<br>H -0.56134 0.15732 1.30310<br>O -0.16692 -2.15725 0.11638<br>O 2.54178 -2.46982 -0.20899<br>C 3.12905 2.38499 0.06758<br>H 3.88571 2.28591 0.85399<br>H 3.66514 2.43205 -0.88714<br>H 2.60213 3.33155 0.21305<br>S -1.47318 0.44811 -0.83363<br>C -2.87236 0.19011 0.23575<br>C -4.20205 0.32268 -0.46195<br>H -4.77039 1.12031 0.02369<br>H -4.10068 0.54199 -1.52579<br>H -4.75125 -0.61388 -0.33449<br>O -2.76987 -0.05543 1.42106	C_1	-972.18918 (1.5)	-972.01247 (1.3)	-972.06802 (1.4)	-972.36620 (3.4)	-972.24505 (2.7)

Table S13.5. One-electron oxidized neutral form in vacuo.

Structure	Schematic drawing	Symmetry	E	H_{RRHO}	G_{RRHO}	$E_{\omega B97X\text{-}D,large}$	$G_{RRHO,\omega B97X\text{-}D,large}$
6-adduct, conf1	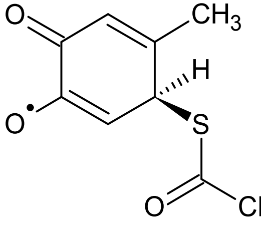<br>[(3*S*)-3-(acetylsulfanyl)-4-methyl-6-oxocyclohexa-1,4-dien-1-yl]oxidanyl<br><br>C 2.25306 -1.05893 0.22821<br>C 2.75115 0.32941 -0.20348<br>C 1.77830 1.43452 -0.15192<br>C 0.47470 1.28025 0.13612	C_1	-972.00378 (5.0)	-971.82643 (5.1)	-971.88328 (5.2)	-972.17382 (4.7)	-972.05332 (5.0)

	C -0.10787 -0.07824 0.43911<br>C 0.86878 -1.18934 0.54434<br>H 2.18739 2.41936 -0.35975<br>H -0.70432 -0.01489 1.36337<br>H 0.51303 -2.16693 0.85653<br>O 3.03354 -2.01208 0.30570<br>O 3.90284 0.49248 -0.54726<br>C -0.47302 2.44152 0.19925<br>H -0.98359 2.46761 1.16772<br>H 0.05074 3.38700 0.04957<br>H -1.24768 2.35035 -0.56961<br>S -1.38651 -0.56208 -0.81018<br>C -2.87888 -0.29406 0.15505<br>C -4.14588 -0.56398 -0.61550<br>H -4.48669 0.37776 -1.05746<br>H -3.99645 -1.28994 -1.41718<br>H -4.91003 -0.91618 0.07922<br>O -2.85641 0.09115 1.29946						
6-adduct, conf2	C -2.21338 -1.05603 -0.28644<br>C -2.72219 0.30441 0.21594<br>C -1.77249 1.43171 0.16898<br>C -0.47693 1.31248 -0.16528<br>C 0.13078 -0.03023 -0.49299<br>C -0.83670 -1.14608 -0.64913<br>H -2.19389 2.40083 0.42102<br>H 0.72240 0.05573 -1.41031<br>H -0.47405 -2.10162 -1.01667<br>O -2.98038 -2.01657 -0.38884<br>O -3.86344 0.43343 0.60325<br>C 0.44347 2.49588 -0.22675<br>H 0.85711 2.60900 -1.23618<br>H -0.07920 3.41820 0.03121<br>H 1.28371 2.37106 0.46457<br>S 1.32315 -0.54352 0.84750<br>C 2.98911 -0.39917 0.17135<br>C 3.21582 0.01133 -1.26318<br>H 2.76520 -0.70900 -1.95264<br>H 2.79111 0.99882 -1.46367<br>H 4.29256 0.04201 -1.43139<br>O 3.88379 -0.65869 0.93547	C_1	-971.99305 (11.7)	-971.81584 (11.7)	-971.87199 (12.3)	-972.16383 (10.9)	-972.04278 (11.6)
6-adduct, conf3	C 1.79289 -0.87987 0.65697<br>C 2.24472 0.05730 -0.47281<br>C 1.45501 1.28371 -0.68318	C_1	-971.99643 (9.6)	-971.81935 (9.5)	-971.87515 (10.3)	-972.16710 (8.9)	-972.04582 (9.7)

	C 0.32305 1.57910 -0.01976<br>C -0.21949 0.66193 1.05059<br>C 0.60745 -0.52786 1.36659<br>H 1.84792 1.96270 -1.43509<br>H -0.31756 1.25571 1.97283<br>H 0.29094 -1.18021 2.17546<br>O 2.43796 -1.89724 0.92667<br>O 3.21400 -0.21449 -1.14912<br>C -0.43773 2.84663 -0.27905<br>H -0.63861 3.37652 0.65859<br>H 0.12248 3.50887 -0.94101<br>H -1.40906 2.63303 -0.73583<br>S -2.01101 0.20194 0.83753<br>C -2.18051 -0.90654 -0.58513<br>C -0.98111 -1.36453 -1.36884<br>H -0.35488 -0.52246 -1.67190<br>H -0.37545 -2.04482 -0.76249<br>H -1.33922 -1.89696 -2.25047<br>O -3.31146 -1.24110 -0.82790						
6-adduct, conf4	C -1.72620 -0.87865 -0.68381<br>C -2.11957 -0.07147 0.56262<br>C -1.40964 1.20116 0.79017<br>C -0.35396 1.61400 0.07199<br>C 0.19725 0.77868 -1.06061<br>C -0.60787 -0.40958 -1.43931<br>H -1.80140 1.81013 1.60025<br>H 0.31124 1.42195 -1.94489<br>H -0.32082 -0.97444 -2.32154<br>O -2.36880 -1.87335 -1.02091<br>O -3.00670 -0.45618 1.29359<br>C 0.31538 2.93436 0.32270<br>H 0.27086 3.56428 -0.57368<br>H -0.16683 3.46833 1.14330<br>H 1.37329 2.79837 0.56812<br>S 1.97182 0.29267 -0.78389<br>C 1.81160 -0.90951 0.54754<br>C 3.13443 -1.49425 0.97619<br>H 3.43345 -2.25829 0.25166<br>H 3.91837 -0.73429 1.01342<br>H 3.00941 -1.96084 1.95394<br>O 0.74735 -1.22127 1.01816	C_1	-972.00108<br>(6.7)	-971.82381<br>(6.7)	-971.87935<br>(7.7)	-972.17096<br>(6.5)	-972.04923<br>(7.5)
6-adduct, conf5	C 1.72541 -0.87883 0.68420<br>C 2.11907 -0.07230 -0.56252	C_1	-972.00110<br>(6.6)	-971.82392<br>(6.6)	-971.87946<br>(7.6)	-972.17096<br>(6.5)	-972.04933<br>(7.5)

	C 1.40919 1.20024 -0.79094<br>C 0.35393 1.61400 -0.07272<br>C -0.19651 0.78022 1.06148<br>C 0.60834 -0.40815 1.44050<br>H 1.80047 1.80812 -1.60206<br>H -0.30941 1.42457 1.94515<br>H 0.32188 -0.97202 2.32357<br>O 2.36699 -1.87423 1.02119<br>O 3.00621 -0.45751 -1.29317<br>C -0.31597 2.93372 -0.32530<br>H -0.27177 3.56512 0.57006<br>H 0.16601 3.46663 -1.14673<br>H -1.37381 2.79701 -0.57073<br>S -1.97089 0.29339 0.78581<br>C -1.81261 -0.90556 -0.54500<br>C -3.13253 -1.49655 -0.97472<br>H -3.24656 -2.46793 -0.48339<br>H -3.98215 -0.86542 -0.70658<br>H -3.10576 -1.65945 -2.05368<br>O -0.75002 -1.21723 -1.02033						
6-adduct, conf6	C 2.24072 -1.07104 0.22519<br>C 2.75533 0.31387 -0.19785<br>C 1.79435 1.42934 -0.14452<br>C 0.48825 1.28797 0.13883<br>C -0.11007 -0.06566 0.43286<br>C 0.85389 -1.18799 0.53516<br>H 2.21484 2.41060 -0.34640<br>H -0.70943 -0.00159 1.35540<br>H 0.48624 -2.16316 0.84102<br>O 3.01062 -2.03289 0.30112<br>O 3.90997 0.46621 -0.53664<br>C -0.44718 2.45913 0.20389<br>H -0.96074 2.48629 1.17083<br>H 0.08716 3.39972 0.06086<br>H -1.21987 2.38010 -0.56818<br>S -1.39068 -0.52446 -0.82355<br>C -2.87970 -0.29559 0.15375<br>C -4.15005 -0.57339 -0.60896<br>H -4.65914 0.37986 -0.77924<br>H -3.96900 -1.05958 -1.56907<br>H -4.79770 -1.19705 0.01118<br>O -2.85743 0.06373 1.30681	C_1	-972.00383 (4.9)	-971.82632 (5.1)	-971.88227 (5.9)	-972.17387 (4.6)	-972.05232 (5.6)

6-adduct, conf7	C 1.85069 -1.27821 0.20844<br>C 2.66407 -0.05270 -0.23811<br>C 2.02715 1.26758 -0.07331<br>C 0.75015 1.44523 0.30367<br>C -0.16689 0.26773 0.54044<br>C 0.50564 -1.05074 0.62948<br>H 2.67236 2.12014 -0.26642<br>H -0.76512 0.46122 1.43615<br>H -0.06065 -1.91630 0.96169<br>O 2.36103 -2.40014 0.22734<br>O 3.78836 -0.18142 -0.67192<br>C 0.15364 2.80599 0.51423<br>H -0.10774 2.94208 1.57048<br>H 0.85516 3.59262 0.23208<br>H -0.76441 2.92853 -0.06875<br>S -1.36195 0.19906 -0.89558<br>C -2.92572 -0.40426 -0.22997<br>C -3.07709 -0.67987 1.24629<br>H -2.31378 -1.37168 1.61157<br>H -3.00192 0.25167 1.81607<br>H -4.06453 -1.11438 1.40376<br>O -3.81789 -0.54386 -1.02720	C_1	-971.99352 (11.4)	-971.81614 (11.5)	-971.87189 (12.4)	-972.16427 (10.7)	-972.04264 (11.7)
6-adduct, conf8	C 2.22795 -1.07957 0.23562<br>C 2.75429 0.29613 -0.20300<br>C 1.80601 1.42256 -0.15084<br>C 0.49962 1.29664 0.13853<br>C -0.11223 -0.04945 0.43922<br>C 0.84135 -1.17954 0.55274<br>H 2.23619 2.39817 -0.35952<br>H -0.71678 0.02641 1.35716<br>H 0.46529 -2.14772 0.87034<br>O 2.98864 -2.04817 0.31765<br>O 3.90778 0.43290 -0.55215<br>C -0.42356 2.47749 0.20156<br>H -0.93154 2.51546 1.17109<br>H 0.11933 3.41186 0.05018<br>H -1.20147 2.40163 -0.56549<br>S -1.38768 -0.50370 -0.82453<br>C -2.88237 -0.27782 0.14475<br>C -4.14322 -0.62255 -0.60631<br>H -4.86775 0.18183 -0.46071<br>H -3.96994 -0.78222 -1.67186<br>H -4.55936 -1.53516 -0.16952	C_1	-972.00382 (4.9)	-971.82636 (5.1)	-971.88274 (5.6)	-972.17387 (4.6)	-972.05280 (5.3)

	O -2.86869 0.10097 1.29165						
6-adduct, conf9	C 1.85748 -1.27862 0.19597<br>C 2.67271 -0.04662 -0.22747<br>C 2.03052 1.27020 -0.05577<br>C 0.74851 1.44184 0.30693<br>C -0.16640 0.26010 0.53147<br>C 0.50785 -1.05866 0.60530<br>H 2.67531 2.12609 -0.23502<br>H -0.75956 0.44251 1.43295<br>H -0.06031 -1.92942 0.92041<br>O 2.36948 -2.40005 0.20615<br>O 3.80166 -0.16762 -0.65133<br>C 0.14380 2.80093 0.50501<br>H -0.23001 2.90645 1.53026<br>H 0.87725 3.58827 0.32395<br>H -0.70577 2.95247 -0.16832<br>S -1.37143 0.20362 -0.89591<br>C -2.93052 -0.40340 -0.22247<br>C -3.06993 -0.69277 1.25247<br>H -2.30413 -1.38827 1.60530<br>H -2.99013 0.23327 1.83055<br>H -4.05621 -1.12857 1.41359<br>O -3.82961 -0.53468 -1.01329	C_1	-971.99351 (11.4)	saddle point			
5-adduct, conf1	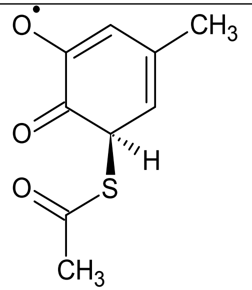<br>[(5*R*)-5-(acetylsulfanyl)-3-methyl-6-oxocyclohexa-1,3-dien-1-yl]oxidanyl<br><br>C -0.30137 -1.29348 -0.60155<br>C -1.52586 -1.30967 0.33658<br>C -2.22104 -0.06468 0.55302<br>C -1.85224 1.15187 -0.05057<br>C -0.77538 1.21440 -0.90354<br>C 0.07733 0.04106 -1.26301<br>H -3.06851 -0.10551 1.23171<br>H -0.51159 2.16023 -1.36883	C_1	-972.01169 (0.0)	-971.83451 (0.0)	-971.88989 (1.1)	-972.18125 (0.0)	-972.05945 (1.1)

	H 0.01203 -0.13841 -2.34499<br>O 0.30447 -2.30506 -0.84594<br>O -1.86464 -2.36318 0.85694<br>C -2.65724 2.38853 0.26420<br>H -3.71977 2.22217 0.06407<br>H -2.55649 2.64415 1.32362<br>H -2.32676 3.24607 -0.32604<br>S 1.86103 0.41779 -1.04225<br>C 1.94627 0.33581 0.74124<br>C 3.32110 0.58078 1.30849<br>H 3.80583 -0.39035 1.45026<br>H 3.94327 1.18916 0.64904<br>H 3.21773 1.06065 2.28313<br>O 0.97996 0.06318 1.41461						
5-adduct, conf2	C 0.38344 1.29352 -0.57207<br>C 1.68662 1.25163 0.25897<br>C 2.32429 -0.02778 0.44477<br>C 1.82266 -1.23482 -0.07949<br>C 0.66558 -1.24522 -0.82612<br>C -0.11692 -0.02244 -1.18054<br>H 3.23422 -0.02448 1.03893<br>H 0.28820 -2.18317 -1.22377<br>H -0.02573 0.13164 -2.26693<br>O -0.19332 2.33592 -0.74532<br>O 2.11497 2.28959 0.74577<br>C 2.57790 -2.51296 0.18836<br>H 3.57801 -2.46386 -0.25303<br>H 2.70047 -2.67277 1.26367<br>H 2.06310 -3.37986 -0.23061<br>S -1.93262 -0.28197 -1.03759<br>C -2.32051 -0.34791 0.72266<br>C -1.27777 -0.00053 1.75396<br>H -0.34667 -0.55007 1.59556<br>H -1.06452 1.07226 1.71015<br>H -1.68396 -0.24104 2.73691<br>O -3.45827 -0.63777 0.99550	C_1	-972.00393 (4.9)	-971.82687 (4.8)	-971.88266 (5.6)	-972.17400 (4.5)	-972.05274 (5.3)
5-adduct, conf3	C 0.48983 1.18540 0.05167<br>C 2.02400 1.33725 -0.03132<br>C 2.80899 0.12776 -0.03882<br>C 2.25115 -1.16451 -0.06856<br>C 0.88617 -1.33745 -0.12708<br>C -0.09001 -0.21079 -0.19105<br>H 3.88738 0.25948 -0.01272	C_1	-972.01078 (0.6)	-971.83381 (0.4)	-971.89162 (0.0)	-972.18014 (0.7)	-972.06098 (0.2)

	H 0.46999 -2.33983 -0.17065<br>H -0.52559 -0.16579 -1.20754<br>O -0.21023 2.13947 0.27287<br>O 2.51716 2.45731 -0.04629<br>C 3.17686 -2.35575 -0.04862<br>H 3.90504 -2.29389 -0.86270<br>H 3.73616 -2.38878 0.89159<br>H 2.62849 -3.29448 -0.15127<br>S -1.54489 -0.52715 0.86039<br>C -2.84685 -0.22831 -0.34085<br>C -4.22755 -0.23886 0.26076<br>H -4.93746 -0.61111 -0.47946<br>H -4.27736 -0.83595 1.17381<br>H -4.49013 0.79468 0.50809<br>O -2.62116 -0.01908 -1.50811						
5-adduct, conf4	C -0.27015 1.14647 0.49907<br>C -1.46924 1.42172 -0.42859<br>C -2.47107 0.38210 -0.49227<br>C -2.33805 -0.85952 0.16275<br>C -1.16226 -1.18924 0.80314<br>C 0.04700 -0.31405 0.77438<br>H -3.37446 0.61631 -1.04858<br>H -1.05781 -2.15413 1.29027<br>H 0.61569 -0.39280 1.70329<br>O 0.37192 2.05009 0.97849<br>O -1.56261 2.49280 -1.00955<br>C -3.49772 -1.82282 0.13502<br>H -4.38534 -1.37221 0.58873<br>H -3.74993 -2.08254 -0.89765<br>H -3.26798 -2.74567 0.67155<br>S 1.09586 -0.95205 -0.62265<br>C 2.80273 -0.44059 -0.28204<br>C 3.13954 0.28440 0.99160<br>H 2.54319 1.19426 1.09930<br>H 2.93634 -0.35769 1.85500<br>H 4.20254 0.52607 0.96691<br>O 3.60561 -0.74909 -1.12454	C_1	-972.00666 (3.2)	-971.82943 (3.2)	-971.88530 (4.0)	-972.17695 (2.7)	-972.05559 (3.5)
5-adduct, conf5	C -0.99517 1.28376 0.51470<br>C -2.26124 0.86646 -0.25799<br>C -2.48603 -0.55114 -0.41041<br>C -1.58212 -1.53480 0.03475<br>C -0.35883 -1.17506 0.55997<br>C 0.10978 0.23683 0.64189	C_1	-972.01058 (0.7)	-971.83343 (0.7)	-971.89140 (0.1)	-972.18042 (0.5)	-972.06124 (0.0)

	H -3.42768 -0.83843 -0.87041<br>H 0.33990 -1.93270 0.90162<br>H 0.65870 0.41899 1.57048<br>O -0.88724 2.38212 0.99800<br>O -3.03105 1.72256 -0.67352<br>C -1.96966 -2.98713 -0.08970<br>H -2.89991 -3.18729 0.45023<br>H -2.13596 -3.24761 -1.13968<br>H -1.19396 -3.64580 0.30621<br>S 1.33071 0.57792 -0.70778<br>C 2.78185 -0.15155 0.04257<br>C 4.03090 -0.01141 -0.79209<br>H 4.48067 -0.99959 -0.91482<br>H 3.84005 0.43359 -1.77022<br>H 4.73676 0.61628 -0.24111<br>O 2.75494 -0.69465 1.12177						
5-adduct, conf6	C 1.01673 -1.28232 0.50950<br>C 2.27072 -0.83881 -0.26831<br>C 2.46985 0.58339 -0.41436<br>C 1.55173 1.54865 0.04177<br>C 0.33858 1.16467 0.57355<br>C -0.10533 -0.25543 0.64956<br>H 3.40342 0.88982 -0.87843<br>H -0.37063 1.90838 0.92422<br>H -0.64518 -0.45409 1.57993<br>O 0.93080 -2.38511 0.98704<br>O 3.05279 -1.67907 -0.69308<br>C 1.91242 3.00829 -0.07800<br>H 2.84034 3.22330 0.46025<br>H 2.07095 3.27604 -1.12734<br>H 1.12595 3.65102 0.32285<br>S -1.32656 -0.60953 -0.69658<br>C -2.78140 0.11760 0.05170<br>C -4.00916 0.04189 -0.82177<br>H -4.15785 1.02090 -1.28764<br>H -3.92019 -0.71227 -1.60603<br>H -4.87352 -0.17253 -0.19033<br>O -2.76274 0.63849 1.14177	C_1	-972.01055 (0.7)	-971.83336 (0.7)	-971.89062 (0.6)	-972.18041 (0.5)	-972.06047 (0.5)
5-adduct, conf7	C -0.48861 1.18075 -0.08514<br>C -2.01881 1.33921 0.04592<br>C -2.80862 0.13278 0.06245<br>C -2.25523 -1.16149 0.05903<br>C -0.88964 -1.33980 0.08271	C_1	-972.01078 (0.6)	-971.83385 (0.4)	-971.89151 (0.1)	-972.18011 (0.7)	-972.06084 (0.3)

	C 0.08908 -0.21591 0.15982<br>H -3.88678 0.26881 0.06579<br>H -0.47586 -2.34402 0.09529<br>H 0.50592 -0.17150 1.18481<br>O 0.20779 2.12915 -0.33877<br>O -2.50573 2.46128 0.08725<br>C -3.18475 -2.34996 0.06129<br>H -3.74896 -2.39127 0.99836<br>H -3.90839 -2.27792 -0.75582<br>H -2.63813 -3.28906 -0.04742<br>S 1.56129 -0.53134 -0.86477<br>C 2.83991 -0.22605 0.35909<br>C 4.23240 -0.23972 -0.21443<br>H 4.92679 -0.61069 0.54102<br>H 4.30015 -0.83926 -1.12465<br>H 4.50129 0.79288 -0.45886<br>O 2.59157 -0.00987 1.52069						
5-adduct, conf8	C -0.47406 1.17093 -0.09868<br>C -2.00122 1.35080 0.04010<br>C -2.80660 0.15468 0.06248<br>C -2.26959 -1.14645 0.06459<br>C -0.90638 -1.34184 0.09183<br>C 0.08596 -0.22961 0.16457<br>H -3.88297 0.30423 0.06549<br>H -0.50534 -2.35116 0.10978<br>H 0.49634 -0.17752 1.19171<br>O 0.23279 2.10604 -0.37173<br>O -2.47296 2.47932 0.08050<br>C -3.21377 -2.32328 0.06906<br>H -3.77901 -2.35581 1.00585<br>H -3.93599 -2.24395 -0.74865<br>H -2.67865 -3.26921 -0.03764<br>S 1.55872 -0.57652 -0.84861<br>C 2.83698 -0.21836 0.36336<br>C 4.22746 -0.24427 -0.21439<br>H 4.94421 -0.43759 0.58462<br>H 4.32527 -0.99192 -1.00498<br>H 4.43307 0.73996 -0.64704<br>O 2.58652 0.03610 1.51637	C_1	-972.01077 (0.6)	-971.83382 (0.4)	-971.89135 (0.2)	-972.18009 (0.7)	-972.06066 (0.4)
5-adduct, conf9	C 0.92292 -1.31009 -0.03762<br>C 2.36372 -0.88622 0.32167<br>C 2.68030 0.51233 0.15612<br>C 1.71647 1.47991 -0.18773	C_1	-971.99953 (7.6)	-971.82259 (7.5)	-971.87861 (8.2)	-972.16978 (7.2)	-972.04886 (7.8)

	C 0.38093 1.14336 -0.24242<br>C -0.13669 -0.21511 0.13204<br>H 3.72670 0.78534 0.26121<br>H -0.35083 1.89050 -0.53580<br>H -0.33855 -0.22716 1.21484<br>O 0.67471 -2.42969 -0.39670<br>O 3.17462 -1.73327 0.66730<br>C 2.16784 2.88974 -0.47739<br>H 2.63702 3.33019 0.40781<br>H 2.90849 2.89965 -1.28206<br>H 1.33215 3.52747 -0.77297<br>S -1.67360 -0.68273 -0.74122<br>C -2.97624 0.17438 0.15135<br>C -2.68785 0.86294 1.46607<br>H -2.35700 0.13416 2.21285<br>H -1.91192 1.62699 1.36641<br>H -3.61272 1.32805 1.80786<br>O -4.07985 0.12809 -0.33473						

Table S13.6. One-electron oxidized neutral form in water.

Structure	Schematic drawing	Symmetry	G_{PCM}	$H_{PCM,RRHO}$	$G_{PCM,RRHO}$	$G_{SMD,\omega B97X\text{-}D,large}$	$G_{SMD,RRHO,\omega B97X\text{-}D,large}$
6-adduct, conf1	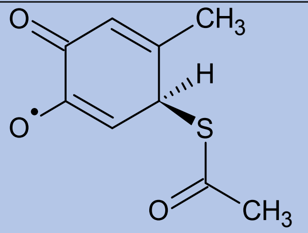<br>[(3*S*)-3-(acetylsulfanyl)-4-methyl-6-oxocyclohexa-1,4-dien-1-yl]oxidanyl<br><br>C 2.20146 -1.08840 0.21701<br>C 2.74592 0.28682 -0.19146<br>C 1.82442 1.42029 -0.13816<br>C 0.51295 1.30529 0.14745<br>C -0.11499 -0.03558 0.43010<br>C 0.81766 -1.18112 0.52708<br>H 2.26201 2.39457 -0.33509<br>H -0.71308 0.03040 1.35335	C_1	-972.02310 (5.1)	-971.84575 (5.2)	-971.90215 (5.3)	-972.19574 (4.8)	-972.07478 (4.6)

	H 0.42330 -2.14520 0.83319<br>O 2.96367 -2.06429 0.27978<br>O 3.91639 0.40052 -0.52022<br>C -0.39083 2.49706 0.22258<br>H -0.87452 2.54171 1.20399<br>H 0.16005 3.42378 0.05772<br>H -1.18636 2.42644 -0.52609<br>S -1.39194 -0.45709 -0.83788<br>C -2.88315 -0.31319 0.15541<br>C -4.14529 -0.56801 -0.62011<br>H -4.63963 0.39419 -0.78455<br>H -3.95811 -1.04040 -1.58529<br>H -4.80533 -1.19494 -0.01727<br>O -2.85583 -0.01823 1.32879						
6-adduct, conf2	C -1.95572 -1.20952 -0.24644<br>C -2.67850 0.06079 0.22552<br>C -1.95821 1.32931 0.10939<br>C -0.66635 1.42124 -0.25954<br>C 0.15912 0.18698 -0.52840<br>C -0.59884 -1.07920 -0.65265<br>H -2.53458 2.22453 0.32407<br>H 0.77292 0.35330 -1.41730<br>H -0.08781 -1.96276 -1.02236<br>O -2.56307 -2.28820 -0.28870<br>O -3.82192 -0.01481 0.64463<br>C 0.02643 2.73607 -0.43858<br>H 0.26752 2.87974 -1.49799<br>H -0.60183 3.56430 -0.10886<br>H 0.96992 2.76195 0.11470<br>S 1.33422 -0.03447 0.90591<br>C 2.95234 -0.41090 0.21453<br>C 3.16372 -0.47277 -1.27073<br>H 2.50541 -1.21254 -1.73330<br>H 2.96646 0.50128 -1.72740<br>H 4.20227 -0.74843 -1.45369<br>O 3.82604 -0.59471 1.03267	C_1	-972.01718 (8.9)	-971.83978 (9.0)	-971.89554 (9.5)	-972.19200 (7.2)	-972.07037 (7.4)
6-adduct, conf3	C 1.82513 -0.92389 0.58789<br>C 2.29263 0.10320 -0.45033<br>C 1.47653 1.30397 -0.62564<br>C 0.32249 1.53549 0.03019<br>C -0.20244 0.56099 1.05726<br>C 0.61178 -0.65726 1.27911<br>H 1.85702 2.03239 -1.33573	C_1	-972.01758 (8.6)	-971.84056 (8.5)	-971.89610 (9.1)	-972.19311 (6.5)	-972.07163 (6.6)

	H -0.24531 1.09909 2.01752<br>H 0.28053 -1.37306 2.02515<br>O 2.49789 -1.94246 0.80234<br>O 3.31472 -0.09954 -1.08544<br>C -0.46593 2.79027 -0.18825<br>H -0.72713 3.25255 0.76911<br>H 0.09987 3.50550 -0.78623<br>H -1.40561 2.57035 -0.70476<br>S -2.00332 0.14528 0.89191<br>C -2.24043 -0.82909 -0.60123<br>C -1.08525 -1.20675 -1.48144<br>H -0.49716 -0.32991 -1.76154<br>H -0.43346 -1.91089 -0.95578<br>H -1.47850 -1.68739 -2.37737<br>O -3.38812 -1.15108 -0.81768						
6-adduct, conf4	C -2.21880 -1.06897 -0.23011<br>C -2.73481 0.30758 0.21008<br>C -1.79870 1.42879 0.15136<br>C -0.49287 1.29656 -0.15204<br>C 0.11232 -0.05172 -0.44877<br>C -0.84027 -1.17965 -0.55755<br>H -2.21968 2.40761 0.36158<br>H 0.71288 0.01362 -1.36996<br>H -0.46498 -2.14494 -0.88327<br>O -2.99884 -2.02992 -0.30192<br>O -3.89625 0.43238 0.56556<br>C 0.42807 2.47478 -0.23080<br>H 0.90162 2.51750 -1.21713<br>H -0.10660 3.40885 -0.05448<br>H 1.23058 2.38767 0.50882<br>S 1.37707 -0.51130 0.81866<br>C 2.88175 -0.31133 -0.14853<br>C 4.13337 -0.56054 0.64507<br>H 4.44772 0.38823 1.09165<br>H 3.97451 -1.28353 1.44705<br>H 4.91738 -0.90787 -0.02900<br>O 2.86423 0.02011 -1.31189	C_1	-972.02305 (5.2)	-971.84587 (5.2)	-971.90321 (4.7)	-972.19569 (4.8)	-972.07586 (4.0)
6-adduct, conf5	C -1.71186 -0.86519 -0.68979<br>C -2.10555 -0.06373 0.55773<br>C -1.41499 1.20294 0.79542<br>C -0.35589 1.61950 0.07733<br>C 0.19759 0.78914 -1.05669<br>C -0.59985 -0.39835 -1.44390	C_1	-972.02278 (5.3)	-971.84549 (5.4)	-971.90076 (6.2)	-972.19567 (4.9)	-972.07365 (5.3)

	H -1.80412 1.81052 1.60736<br>H 0.31029 1.43318 -1.93960<br>H -0.30910 -0.95191 -2.33146<br>O -2.35978 -1.86951 -1.01132<br>O -2.99061 -0.48107 1.28802<br>C 0.30768 2.93767 0.33617<br>H 0.26058 3.56515 -0.56062<br>H -0.17470 3.46545 1.15986<br>H 1.36595 2.80046 0.57757<br>S 1.96426 0.29976 -0.77108<br>C 1.80572 -0.92036 0.53457<br>C 3.12826 -1.48553 0.97667<br>H 3.43864 -2.24978 0.25704<br>H 3.90382 -0.71796 1.01387<br>H 3.00593 -1.94828 1.95643<br>O 0.73388 -1.26188 0.98227						
6-adduct, conf6	C 1.71137 -0.86438 0.69041<br>C 2.10415 -0.06456 -0.55844<br>C 1.41330 1.20170 -0.79750<br>C 0.35531 1.61972 -0.07866<br>C -0.19660 0.79147 1.05773<br>C 0.60112 -0.39551 1.44586<br>H 1.80137 1.80773 -1.61108<br>H -0.30887 1.43696 1.93959<br>H 0.31180 -0.94735 2.33496<br>O 2.35867 -1.86904 1.01208<br>O 2.98890 -0.48273 -1.28864<br>C -0.30853 2.93746 -0.33917<br>H -0.26082 3.56667 0.55633<br>H 0.17308 3.46366 -1.16433<br>H -1.36699 2.79983 -0.57964<br>S -1.96305 0.30002 0.77288<br>C -1.80602 -0.91818 -0.53035<br>C -3.12629 -1.48696 -0.97559<br>H -3.25058 -2.46539 -0.50109<br>H -3.97083 -0.85349 -0.70005<br>H -3.09995 -1.62923 -2.05751<br>O -0.73585 -1.26127 -0.98206	C_1	-972.02282 (5.3)	-971.84561 (5.3)	-971.90098 (6.1)	-972.19576 (4.8)	-972.07392 (5.2)
6-adduct, conf7	C 2.03615 -1.15980 0.28085<br>C 2.69195 0.13313 -0.22550<br>C 1.90419 1.36361 -0.14870<br>C 0.60918 1.39848 0.21914<br>C -0.15143 0.13123 0.52311	C_1	-972.01714 (8.9)	-971.83969 (9.0)	-971.89556 (9.5)	-972.19172 (7.3)	-972.07015 (7.5)

	C 0.67263 -1.09039 0.67993<br>H 2.43280 2.28151 -0.38876<br>H -0.76885 0.28320 1.41214<br>H 0.20517 -1.98895 1.07034<br>O 2.69819 -2.20431 0.35143<br>O 3.84035 0.10702 -0.63737<br>C -0.14877 2.68137 0.35971<br>H -0.40988 2.83743 1.41264<br>H 0.44158 3.53203 0.01736<br>H -1.08568 2.64857 -0.20463<br>S -1.31939 -0.20772 -0.89335<br>C -2.96916 -0.42038 -0.20378<br>C -3.22575 -0.27254 1.26820<br>H -2.62270 -0.97422 1.84979<br>H -2.99345 0.74387 1.59889<br>H -4.28237 -0.47211 1.44688<br>O -3.82857 -0.67222 -1.01892						
6-adduct, conf8	C -1.72126 -0.86939 -0.67968<br>C -2.11827 -0.04697 0.55256<br>C -1.42133 1.21860 0.77600<br>C -0.35122 1.61524 0.06278<br>C 0.19704 0.77098 -1.06309<br>C -0.60159 -0.42119 -1.43313<br>H -1.81037 1.83993 1.57757<br>H 0.30070 1.40539 -1.95429<br>H -0.30854 -0.99020 -2.31015<br>O -2.37289 -1.87503 -0.99023<br>O -3.00941 -0.44876 1.28436<br>C 0.33634 2.92032 0.32840<br>H 0.46039 3.48559 -0.60092<br>H -0.23213 3.52433 1.03681<br>H 1.33678 2.75259 0.73972<br>S 1.96537 0.28776 -0.78411<br>C 1.81234 -0.90899 0.53904<br>C 3.13218 -1.47804 0.98472<br>H 3.25597 -2.45678 0.51059<br>H 3.97676 -0.84531 0.70750<br>H 3.10657 -1.61943 2.06670<br>O 0.74262 -1.23916 1.00169	C_1	-972.02282 (5.3)	-971.84560 (5.3)	-971.90133 (5.8)	-972.19584 (4.7)	-972.07436 (4.9)

5-adduct, conf1	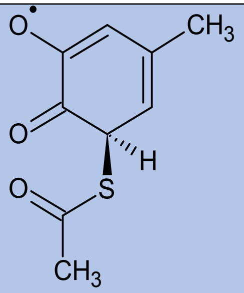<br>[(5*R*)-5-(acetylsulfanyl)-3-methyl-6-oxocyclohexa-1,3-dien-1-yl]oxidanyl<br><br>C -0.29086 -1.25718 -0.62807<br>C -1.51347 -1.31597 0.30580<br>C -2.23244 -0.10070 0.54974<br>C -1.87831 1.14023 -0.01024<br>C -0.78948 1.24371 -0.84687<br>C 0.06995 0.09070 -1.24887<br>H -3.08892 -0.17105 1.21445<br>H -0.53478 2.20470 -1.28314<br>H -0.01309 -0.04852 -2.33570<br>O 0.32357 -2.26526 -0.89256<br>O -1.81889 -2.39815 0.80907<br>C -2.71851 2.34915 0.31263<br>H -3.73449 2.21839 -0.07180<br>H -2.79102 2.48768 1.39506<br>H -2.29856 3.25606 -0.12588<br>S 1.85042 0.47907 -1.03514<br>C 1.96828 0.30370 0.73624<br>C 3.33019 0.58912 1.30503<br>H 3.83640 -0.36828 1.46338<br>H 3.94120 1.20454 0.64331<br>H 3.21147 1.08005 2.27256<br>O 1.01946 -0.05974 1.40068	C_1	-972.03129 (0.0)	-971.85408 (0.0)	-971.90936 (0.8)	-972.20339 (0.0)	-972.08147 (0.4)
5-adduct, conf2	C 0.44196 1.30815 -0.32909<br>C 1.87601 1.20110 0.22407<br>C 2.44348 -0.10906 0.34196<br>C 1.80665 -1.27712 -0.11745<br>C 0.59167 -1.20134 -0.76348<br>C -0.10280 0.08774 -1.06668<br>H 3.41339 -0.17574 0.82646<br>H 0.11719 -2.10441 -1.13441<br>H 0.10521 0.31486 -2.12674	C_1	-972.02482 (4.1)	-971.84790 (3.9)	-971.90387 (4.2)	-972.19883 (2.9)	-972.07787 (2.7)

	O -0.18477 2.33234 -0.19551<br>O 2.44649 2.23069 0.58641<br>C 2.48114 -2.60690 0.09671<br>H 3.45427 -2.62433 -0.40226<br>H 2.65361 -2.77747 1.16322<br>H 1.87809 -3.42873 -0.29276<br>S -1.92871 -0.03044 -1.08986<br>C -2.44777 -0.34358 0.59978<br>C -1.44363 -0.41290 1.71509<br>H -0.66052 -1.14413 1.49951<br>H -0.97426 0.56445 1.85887<br>H -1.96432 -0.69442 2.63046<br>O -3.64216 -0.47797 0.76034						
5-adduct, conf3	C 0.52812 1.20108 -0.05285<br>C 2.06237 1.29385 0.02851<br>C 2.80326 0.06837 0.03880<br>C 2.21203 -1.20356 -0.08089<br>C 0.84672 -1.32919 -0.21804<br>C -0.09345 -0.17338 -0.26204<br>H 3.88098 0.15679 0.14350<br>H 0.40306 -2.31353 -0.33104<br>H -0.57010 -0.13197 -1.25795<br>O -0.14610 2.20237 0.02911<br>O 2.58475 2.40748 0.10795<br>C 3.09968 -2.42101 -0.06699<br>H 3.85488 -2.35409 -0.85519<br>H 3.62656 -2.49394 0.88910<br>H 2.52573 -3.33744 -0.21435<br>S -1.50872 -0.44575 0.86171<br>C -2.87003 -0.20536 -0.28012<br>C -4.22189 -0.32683 0.36695<br>H -4.86900 -0.92313 -0.27940<br>H -4.17168 -0.77466 1.36045<br>H -4.64971 0.67665 0.45135<br>O -2.70014 0.04058 -1.45379	C_1	-972.02940 (1.2)	-971.85227 (1.2)	-971.90873 (1.2)	-972.20095 (1.5)	-972.08028 (1.2)
5-adduct, conf4	C -0.29039 1.14428 0.48214<br>C -1.53035 1.41089 -0.38910<br>C -2.50365 0.35778 -0.45550<br>C -2.32635 -0.89810 0.16137<br>C -1.12769 -1.21615 0.76573<br>C 0.05600 -0.30800 0.74623<br>H -3.42916 0.57898 -0.97938<br>H -0.98932 -2.18542 1.23370	C_1	-972.02533 (3.7)	-971.84805 (3.8)	-971.90388 (4.2)	-972.19782 (3.5)	-972.07637 (3.6)

	H 0.62882 -0.39290 1.67107<br>O 0.36681 2.06259 0.91911<br>O -1.66021 2.50741 -0.93226<br>C -3.46274 -1.88570 0.13560<br>H -4.35110 -1.45867 0.60933<br>H -3.72493 -2.12994 -0.89810<br>H -3.20189 -2.81004 0.65356<br>S 1.12140 -0.88620 -0.66482<br>C 2.81906 -0.41957 -0.26986<br>C 3.15060 0.21727 1.04663<br>H 2.54978 1.11523 1.20979<br>H 2.94411 -0.48379 1.86131<br>H 4.21178 0.46703 1.04854<br>O 3.63247 -0.68721 -1.12587						
5-adduct, conf5	C -0.53990 1.20621 0.06284<br>C -2.07426 1.28267 -0.03176<br>C -2.80248 0.04969 -0.04328<br>C -2.19923 -1.21610 0.08169<br>C -0.83309 -1.32796 0.22253<br>C 0.09556 -0.16295 0.26565<br>H -3.88059 0.12711 -0.15272<br>H -0.37998 -2.30768 0.33838<br>H 0.57812 -0.12000 1.25851<br>O 0.12397 2.21549 -0.00489<br>O -2.60767 2.39053 -0.11827<br>C -3.07521 -2.44197 0.06896<br>H -3.83175 -2.38070 0.85633<br>H -3.60041 -2.52174 -0.88748<br>H -2.49264 -3.35254 0.21875<br>S 1.50748 -0.41441 -0.86787<br>C 2.87221 -0.19298 0.27251<br>C 4.22328 -0.34571 -0.37051<br>H 4.73942 -1.18129 0.10962<br>H 4.16092 -0.52526 -1.44453<br>H 4.79826 0.56466 -0.18366<br>O 2.70716 0.04682 1.44827	C_1	-972.02936 (1.2)	-971.85226 (1.2)	-971.91064 (0.0)	-972.20088 (1.6)	-972.08217 (0.0)
5-adduct, conf6	C -0.55016 1.21102 0.07480<br>C -2.08396 1.27221 -0.03865<br>C -2.80036 0.03238 -0.05168<br>C -2.18663 -1.22732 0.08389<br>C -0.82045 -1.32608 0.23374<br>C 0.09785 -0.15288 0.27419<br>H -3.87822 0.09928 -0.17014	C_1	-972.02932 (1.2)	-971.85227 (1.2)	-971.90930 (0.8)	-972.20077 (1.6)	-972.08075 (0.9)

	H -0.35920 -2.30112 0.35681<br>H 0.58664 -0.10669 1.26352<br>O 0.10390 2.22766 0.02282<br>O -2.62711 2.37442 -0.13622<br>C -3.05174 -2.46087 0.07206<br>H -3.81287 -2.40271 0.85523<br>H -3.57111 -2.54969 -0.88674<br>H -2.46191 -3.36552 0.22917<br>S 1.50442 -0.38877 -0.87025<br>C 2.87624 -0.17858 0.26654<br>C 4.22030 -0.36770 -0.38037<br>H 4.57312 -1.37552 -0.14039<br>H 4.17891 -0.25941 -1.46523<br>H 4.92194 0.35375 0.04203<br>O 2.71639 0.06227 1.44247						
5-adduct, conf7	C 0.85572 -1.30080 0.06684<br>C 2.34981 -0.95600 0.21685<br>C 2.72914 0.41626 0.06049<br>C 1.80276 1.45951 -0.12679<br>C 0.44916 1.20495 -0.09786<br>C -0.13572 -0.14370 0.18085<br>H 3.79412 0.63023 0.07076<br>H -0.25166 2.02236 -0.23696<br>H -0.43981 -0.17801 1.23787<br>O 0.51199 -2.44696 -0.09731<br>O 3.14907 -1.87214 0.41178<br>C 2.31924 2.85934 -0.33465<br>H 2.85358 3.20058 0.55697<br>H 3.02282 2.88658 -1.17127<br>H 1.50827 3.56040 -0.53888<br>S -1.62564 -0.49557 -0.82994<br>C -3.01071 0.16951 0.09302<br>C -2.81132 0.78997 1.44810<br>H -2.46285 0.03976 2.16445<br>H -2.07893 1.60014 1.41546<br>H -3.76973 1.18414 1.78603<br>O -4.09281 0.06768 -0.44527	C_1	-972.02220 (5.7)	saddle point			
5-adduct, conf8	C 0.85515 -1.30049 0.08281<br>C 2.35244 -0.95705 0.20071<br>C 2.73025 0.41581 0.04668<br>C 1.80207 1.46233 -0.11174<br>C 0.44899 1.20908 -0.06121<br>C -0.13570 -0.14273 0.19931	C_1	-972.02222 (5.7)	-971.84504 (5.7)	-971.90154 (5.7)	-972.19644 (4.4)	-972.07576 (4.0)

	H 3.79565 0.62797 0.03955<br>H -0.25236 2.03008 -0.17385<br>H -0.45444 -0.18839 1.25117<br>O 0.50692 -2.44820 -0.06054<br>O 3.15506 -1.87490 0.37280<br>C 2.31593 2.86223 -0.32533<br>H 3.00799 3.14164 0.47386<br>H 2.86200 2.92391 -1.27136<br>H 1.50232 3.58908 -0.34901<br>S -1.61359 -0.48304 -0.83508<br>C -3.01250 0.16504 0.07923<br>C -2.83195 0.77131 1.44330<br>H -2.49190 0.01396 2.15616<br>H -2.10062 1.58293 1.42930<br>H -3.79553 1.16032 1.77247<br>O -4.08746 0.06485 -0.47343						
5-adduct, conf9	C 0.29360 1.26253 -0.62517<br>C 1.51506 1.31168 0.31072<br>C 2.22971 0.09255 0.54785<br>C 1.87310 -1.14356 -0.02124<br>C 0.78778 -1.23756 -0.86342<br>C -0.07082 -0.08028 -1.25478<br>H 3.08532 0.15586 1.21438<br>H 0.53344 -2.19403 -1.30968<br>H 0.00726 0.06647 -2.34088<br>O -0.31768 2.27402 -0.88391<br>O 1.82308 2.38955 0.82151<br>C 2.69928 -2.35979 0.31004<br>H 3.75165 -2.18696 0.06782<br>H 2.63907 -2.57554 1.38106<br>H 2.35616 -3.23885 -0.23829<br>S -1.85115 -0.46863 -1.03597<br>C -1.96251 -0.30561 0.73720<br>C -3.32243 -0.59440 1.30903<br>H -3.82974 0.36188 1.47069<br>H -3.93429 -1.20922 0.64749<br>H -3.20028 -1.08726 2.27512<br>O -1.01124 0.05297 1.40070	C_1	-972.03130 (0.0)	-971.85411 (0.0)	-971.90951 (0.7)	-972.20340 (0.0)	-972.08161 (0.3)

Part 14: 4-Methylcatechol – azide adducts, optimizations using the ωB97X-D functional

Table S14.1. Reduced anionic form in water.

Structura	Schematic drawing	Symmetry	G_{PCM}	$H_{PCM,RRHO}$	$G_{PCM,RRHO}$	$G_{SMD,\omega B97X\text{-}D,large}$	$G_{SMD,RRHO,\omega B97X\text{-}D,large}$
6-adduct, conf1	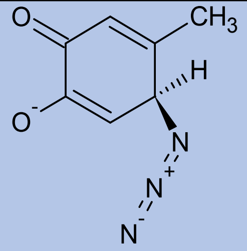<br>(3*S*)-3-azido-4-methyl-6-oxocyclohexa-1,4-dien-1-olate<br><br>C -1.58252 0.58951 0.47880<br>C -1.57124 -0.67686 -0.37564<br>C -0.36257 -1.52093 -0.39525<br>C 0.76762 -1.20324 0.25322<br>C 0.85308 0.09264 1.02673<br>C -0.40719 0.87592 1.13541<br>H -0.43782 -2.44226 -0.96888<br>H 1.26397 -0.12796 2.01755<br>H -0.36558 1.76043 1.76783<br>O -2.67160 1.25578 0.53284<br>O -2.55562 -1.01114 -1.03264<br>C 1.98893 -2.07274 0.23149<br>H 2.28009 -2.34479 1.25198<br>H 1.81489 -2.98750 -0.33870<br>H 2.83609 -1.53789 -0.21025<br>N 1.98852 0.91750 0.41753<br>N 1.70253 1.39084 -0.67773<br>N 1.49604 1.86553 -1.68975	C_1	-584.93417 (0.0)	-584.79519 (0.0)	-584.84428 (0.0)	-585.09109 (0.0)	-585.00120 (0.0)
6-adduct, conf2	C 1.31010 -1.20493 0.16642<br>C 2.04516 0.08972 -0.16510<br>C 1.31135 1.36581 -0.11187<br>C 0.00203 1.45504 0.16349<br>C -0.78409 0.21092 0.48927<br>C -0.02249 -1.06928 0.47896<br>H 1.89694 2.25766 -0.32453	C_1	-584.93181 (1.5)	-584.79283 (1.5)	-584.84258 (1.1)	-585.08901 (1.3)	-584.99977 (0.9)

	H -1.23941 0.37573 1.47950<br>H -0.57806 -1.96339 0.75809<br>O 1.98260 -2.29210 0.14806<br>O 3.23688 0.08598 -0.47235<br>C -0.73874 2.75774 0.18548<br>H -1.22273 2.90836 1.15666<br>H -0.06803 3.59755 -0.00715<br>H -1.53115 2.75736 -0.56971<br>N -1.94100 0.20343 -0.49157<br>N -2.75392 -0.69424 -0.30807<br>N -3.55556 -1.49696 -0.22013						
5-adduct, conf1	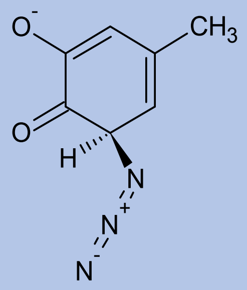<br>(5*R*)-5-azido-3-methyl-6-oxocyclohexa-1,3-dien-1-olate<br><br>C 1.34830 -0.26996 -0.53309<br>C 0.85672 -1.29101 0.48875<br>C -0.51802 -1.46700 0.51607<br>C -1.45669 -0.73075 -0.31448<br>C -1.07505 0.29512 -1.10916<br>C 0.34464 0.74752 -1.10531<br>H -0.91904 -2.23044 1.18018<br>H -1.78653 0.84983 -1.71327<br>H 0.69944 1.02785 -2.09880<br>O 2.51714 -0.20720 -0.87583<br>O 1.72989 -1.91423 1.17203<br>C -2.89760 -1.16779 -0.25376<br>H -3.53208 -0.56203 -0.90548<br>H -2.99539 -2.21836 -0.54887<br>H -3.27729 -1.08789 0.77101<br>N 0.56182 2.01134 -0.29646<br>N -0.11203 2.08723 0.72930<br>N -0.70397 2.25796 1.68309	C_1	-584.92946 (3.0)	-584.79073 (2.8)	-584.84007 (2.6)	-585.08414 (4.4)	-584.99474 (4.1)
5-adduct, conf2	C -0.26659 1.13134 -0.24418<br>C 1.19884 1.39102 0.08078	C_1	-584.93023 (2.5)	-584.79173 (2.2)	-584.84140 (1.8)	-585.08565 (3.4)	-584.99682 (2.7)

	C 1.97173 0.24701 0.22164<br>C 1.49659 -1.10474 -0.00888<br>C 0.26263 -1.35415 -0.49711<br>C -0.61252 -0.20699 -0.90667<br>H 2.99577 0.37343 0.56904<br>H -0.08101 -2.35784 -0.72845<br>H -0.43172 -0.00487 -1.97542<br>O -1.13602 1.95805 -0.01627<br>O 1.55794 2.59167 0.30079<br>C 2.45556 -2.22641 0.29916<br>H 3.35721 -2.14231 -0.31791<br>H 2.77907 -2.17976 1.34499<br>H 2.00369 -3.20514 0.11922<br>N -2.05755 -0.51026 -0.84935<br>N -2.50126 -0.66962 0.28681<br>N -3.01774 -0.84065 1.28309						
5-adduct, conf3	C 0.34836 0.90851 -0.05056<br>C -1.05367 1.48259 0.07993<br>C -2.07099 0.53964 0.06553<br>C -1.86989 -0.89746 0.00769<br>C -0.63977 -1.44933 0.05736<br>C 0.55171 -0.57481 0.28006<br>H -3.09540 0.90875 0.06161<br>H -0.48143 -2.52319 0.05596<br>H 0.74483 -0.54618 1.37119<br>O 1.31099 1.59701 -0.35378<br>O -1.18069 2.74969 0.09596<br>C -3.10602 -1.75454 -0.09348<br>H -2.85804 -2.81765 -0.14850<br>H -3.75665 -1.59579 0.77383<br>H -3.68832 -1.48834 -0.98282<br>N 1.72996 -1.13810 -0.40095<br>N 2.82471 -0.72589 -0.01752<br>N 3.89309 -0.45583 0.25449	C_1	-584.93091 (2.0)	-584.79260 (1.6)	-584.84157 (1.7)	-585.08627 (3.0)	-584.99693 (2.7)
5-adduct, conf4	C 0.33927 -1.28164 -0.01797<br>C 1.80666 -0.89187 0.11405<br>C 2.06069 0.47100 0.06151<br>C 1.04004 1.49817 -0.02640<br>C -0.27588 1.20348 0.02853<br>C -0.71029 -0.20939 0.27385<br>H 3.10200 0.78879 0.05530<br>H -1.03940 1.97631 0.01357<br>H -0.87192 -0.33689 1.36242	C_1	-584.92847 (3.6)	-584.79006 (3.2)	-584.83966 (2.9)	-585.08475 (4.0)	-584.99595 (3.3)

	O 0.00094 -2.42078 -0.29505<br>O 2.66525 -1.82987 0.16747<br>C 1.51555 2.92182 -0.16733<br>H 2.15867 3.19909 0.67535<br>H 2.11352 3.03838 -1.07806<br>H 0.68058 3.62586 -0.21008<br>N -1.96125 -0.57881 -0.42697<br>N -2.96282 0.00821 -0.02878<br>N -3.95153 0.49123 0.25642						

Part 15: 4-Methylcatechol-methanethiol adducts, optimizations at the M06-2X / 6-311++G(2d,2p), SMD level, single-point energies at the DLPNO-CCSD(T)-F12 / cc-pVTZ-F12 level

Table S15.1. One-electron oxidized neutral form in water.

Structure	Schematic drawing	Symmetry	$G_{SMD,M06-2X,large}$	$H_{SMD,RRHO,M06-2X,large}$	$G_{SMD,RRHO,M06-2X,large}$	$E_{vacuo,DLPNO-CCSD(T)-F12/cc-pVTZ-F12}$	$E_{vacuo,M06-2X,large}$	$G_{SMD,RRHO,DLPNO-CCSD(T)-F12/cc-pVTZ-F12}$
6-adduct, conf1	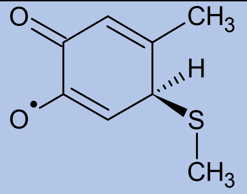<br>[(3*S*)-4-methyl-3-(methylsulfanyl)-6-oxocyclohexa-1,4-dien-1-yl]oxidanyl<br><br>C -1.61858 -0.70062 -0.42040<br>C -1.71278 0.64450 0.29301<br>C -0.57967 1.55211 0.18882<br>C 0.57869 1.21127 -0.39938<br>C 0.79346 -0.15884 -0.96238<br>C -0.39772 -1.01480 -1.06113<br>H -0.71113 2.53681 0.61969<br>H 1.30607 -0.09850 -1.92413<br>H -0.33092 -1.93883 -1.61895<br>O -2.60631 -1.44864 -0.43850<br>O -2.73323 0.92196 0.89867<br>C 1.72946 2.15624 -0.48248<br>H 2.07950 2.23087 -1.51356<br>H 1.45182 3.14276 -0.12119<br>H 2.56582 1.78156 0.11119<br>S 2.01009 -1.10825 0.07757<br>C 1.30967 -0.87992 1.73025<br>H 1.90311 -1.51616 2.38402<br>H 1.40280 0.15380 2.05350<br>H 0.27260 -1.20653 1.76914	C_1	-858.80679 (5.0)	-858.64141 (5.1)	-858.69148 (5.4)	-857.80531 (8.4)	-858.78506 (7.0)	-857.71173 (6.6)
6-adduct, conf2	C 1.44676 -1.09068 0.26227<br>C 2.05167 0.22428 -0.21685<br>C 1.23532 1.42658 -0.12725	C_1	-858.80368 (6.9)	-858.63827 (7.1)	-858.68933 (6.7)	-857.80128 (10.9)	-858.78004 (10.2)	-857.71058 (7.4)

	C -0.04611 1.40931 0.27083<br>C -0.74339 0.12391 0.59765<br>C 0.10594 -1.06922 0.70693<br>H 1.72148 2.35820 -0.38761<br>H -1.39841 0.24881 1.45977<br>H -0.32355 -1.98533 1.08999<br>O 2.15033 -2.11168 0.27206<br>O 3.19822 0.23206 -0.62960<br>C -0.85482 2.65468 0.41303<br>H -1.11807 2.79427 1.46388<br>H -0.30297 3.52495 0.06785<br>H -1.78917 2.57377 -0.14340<br>S -1.86772 -0.29727 -0.82555<br>C -3.12218 -1.24701 0.06806<br>H -3.82553 -1.60597 -0.68011<br>H -2.67106 -2.09881 0.57028<br>H -3.63665 -0.60763 0.78031							
5-adduct, conf1	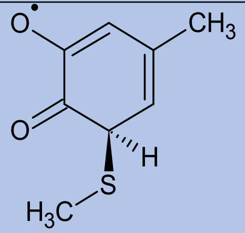<br><br>[(5*R*)-3-methyl-5-(methylsulfanyl)-6-oxocyclohexa-1,3-dien-1-yl]oxidanyl<br><br>C 0.50116 0.90055 0.60155<br>C -0.61091 1.50683 -0.25705<br>C -1.79498 0.72717 -0.41990<br>C -1.93760 -0.56918 0.10269<br>C -0.87310 -1.19483 0.70638<br>C 0.47579 -0.58496 0.78062<br>H -2.62045 1.19400 -0.94291<br>H -0.98102 -2.19838 1.09484<br>H 1.03201 -0.88700 1.66540<br>O 1.36509 1.60672 1.06680<br>O -0.46509 2.64524 -0.70255<br>C -3.26361 -1.26066 -0.02652<br>H -4.03390 -0.70431 0.50789	C_1	-858.81039 (2.7)	-858.64517 (2.8)	-858.69539 (2.9)	-857.81507 (2.3)	-858.79251 (2.4)	-857.71796 (2.7)

	H -3.56222 -1.30745 -1.07391<br>H -3.21908 -2.27096 0.37227<br>S 1.40392 -1.19633 -0.70428<br>C 3.08091 -0.67879 -0.26590<br>H 3.72932 -1.09238 -1.03584<br>H 3.16791 0.40466 -0.26607<br>H 3.35873 -1.08932 0.70158							
5-adduct, conf2	C -0.68863 1.02840 -0.61724<br>C 0.44984 1.57861 0.24410<br>C 1.61270 0.77052 0.38300<br>C 1.74990 -0.49645 -0.20897<br>C 0.70333 -1.05614 -0.90095<br>C -0.63052 -0.42529 -1.01029<br>H 2.43273 1.18907 0.95387<br>H 0.81878 -2.02835 -1.36138<br>H -1.04783 -0.53511 -2.01152<br>O -1.59908 1.75086 -0.94110<br>O 0.31693 2.69578 0.75035<br>C 3.05438 -1.22515 -0.06281<br>H 3.87588 -0.60817 -0.42646<br>H 3.24817 -1.44044 0.98854<br>H 3.04547 -2.16114 -0.61574<br>S -1.82560 -1.36540 0.04103<br>C -1.19118 -1.03492 1.70575<br>H -1.78490 -1.66328 2.36669<br>H -0.14607 -1.32255 1.79231<br>H -1.33453 0.00576 1.98764	C_1	-858.80983 (3.1)	-858.64456 (3.1)	-858.69567 (2.8)	-857.81152 (4.5)	-858.78979 (4.1)	-857.71740 (3.1)
5-adduct, conf3	C 0.38420 -1.29540 0.47022<br>C 1.66530 -0.85473 -0.23863<br>C 1.90336 0.54399 -0.34723<br>C 1.01424 1.51404 0.13279<br>C -0.19436 1.14360 0.67846<br>C -0.67659 -0.25476 0.72499<br>H 2.83985 0.84735 -0.79942<br>H -0.85940 1.90388 1.06548<br>H -1.19124 -0.48398 1.65710<br>O 0.23541 -2.45122 0.78164<br>O 2.43851 -1.72429 -0.64622<br>C 1.39450 2.96261 0.02762<br>H 2.36936 3.13457 0.48320<br>H 1.46695 3.25339 -1.02115<br>H 0.65799 3.59756 0.51392<br>S -1.90517 -0.52646 -0.64380	C_1	-858.80584 (5.6)	-858.64067 (5.6)	-858.69242 (4.8)	-857.80782 (6.8)	-858.78491 (7.1)	-857.71533 (4.4)

	C -3.14958 0.70428 -0.17727<br>H -4.00471 0.51496 -0.82285<br>H -3.44334 0.56295 0.86006<br>H -2.79051 1.71513 -0.34468							

Table S15.2. Transition structures for the addition of methanethiyl radical to 4-methyl-*o*-benzoquinone in water.

Structure	Schematic drawing	Symmetry	$G_{SMD,M06\text{-}2X,large}$	$H_{SMD,RRHO,M06\text{-}2X,large}$	$G_{SMD,RRHO,M06\text{-}2X,large}$	$E_{vacuo,DLPNO\text{-}CCSD(T)\text{-}F12/cc\text{-}pVTZ\text{-}F12}$	$E_{vacuo,M06\text{-}2X,large}$	$G_{SMD,RRHO,DLPNO\text{-}CCSD(T)\text{-}F12/cc\text{-}pVTZ\text{-}F12}$
6-addition, conf1	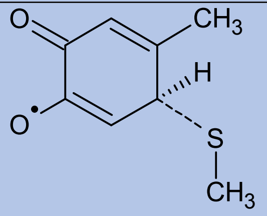<br>C 1.49146 -0.50542 0.66376<br>C 1.62020 0.58152 -0.43098<br>C 0.52281 1.52459 -0.59255<br>C -0.57652 1.44801 0.17750<br>C -0.70241 0.39093 1.20244<br>C 0.28945 -0.49799 1.47467<br>H 0.63634 2.29258 -1.34637<br>H -1.55548 0.45755 1.86579<br>H 0.20339 -1.22474 2.27036<br>O 2.39747 -1.30569 0.80431<br>O 2.63084 0.60297 -1.10480<br>C -1.70402 2.41867 0.07792<br>H -1.81833 2.94146 1.02995<br>H -1.53436 3.14590 -0.71133<br>H -2.63927 1.88935 -0.11004<br>S -2.05420 -1.28954 -0.17628<br>C -0.77681 -1.68149 -1.37712<br>H -1.28865 -2.16840 -2.21000<br>H -0.30540 -0.77736 -1.76170<br>H -0.04249 -2.37478 -0.97608<br><br>ν_{imagin} = 293.2*i* cm^{-1}	C_1	-858.77880 (1.6)	-858.61500 (1.5)	-858.66688 (1.2)	-857.78227 (1.0)	-858.76174 (0.9)	-857.68741 (1.4)
6- addition, conf2	C 1.31999 -1.11450 0.42820<br>C 1.96708 0.04866 -0.36294	C_1	-858.77630 (3.2)	-858.61221 (3.2)	-858.66468 (2.6)	-857.77836 (3.4)	-858.75766 (3.5)	-857.68537 (2.7)

	C 1.31816 1.34984 -0.32058<br>C 0.15363 1.52139 0.32696<br>C -0.50983 0.38037 0.99923<br>C 0.05993 -0.84873 1.09851<br>H 1.81050 2.16765 -0.82999<br>H -1.40153 0.60683 1.57048<br>H -0.40544 -1.65364 1.65053<br>O 1.89199 -2.18651 0.46456<br>O 2.99308 -0.17403 -0.97433<br>C -0.53892 2.83759 0.42006<br>H -0.63270 3.12601 1.46929<br>H 0.00130 3.61203 -0.11757<br>H -1.55109 2.75403 0.02040<br>S -1.90973 -0.33183 -0.98691<br>C -3.13359 -1.21086 0.00452<br>H -3.87423 -1.61046 -0.68924<br>H -2.66988 -2.04768 0.52130<br>H -3.62070 -0.54383 0.70983<br><br>ν_{imagin} = 300.5*i* cm^{-1}							
5-addition, conf1	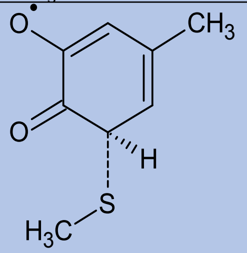<br><br>C 0.54314 0.84162 0.76553<br>C -0.38444 1.45012 -0.30362<br>C -1.62851 0.74805 -0.58778<br>C -1.97712 -0.37751 0.07386<br>C -1.06602 -0.98831 1.03303<br>C 0.16347 -0.48028 1.29171<br>H -2.28677 1.19512 -1.32131<br>H -1.37778 -1.91074 1.50602<br>H 0.83804 -0.94024 2.00256<br>O 1.49050 1.47706 1.18203<br>O -0.06995 2.50345 -0.82524<br>C -3.27631 -1.07386 -0.16803<br>H -3.87819 -0.54875 -0.90478<br>H -3.09108 -2.09242 -0.51416	C_1	-858.78045 (0.5)	-858.61700 (0.2)	-858.66885 (0.0)	-857.78295 (0.6)	-858.76220 (0.7)	-857.68960 (0.0)

	H -3.83537 -1.15162 0.76606<br>S 1.28871 -1.09286 -0.95709<br>C 2.93126 -1.06959 -0.23022<br>H 3.64103 -1.20998 -1.04708<br>H 3.12333 -0.11147 0.25124<br>H 3.05023 -1.88960 0.47378<br><br>ν_{imagin} = 261.6*i* cm^{-1}							
5- addition, conf2	C -1.37424 -0.45531 0.61760<br>C -0.85501 -1.25251 -0.59874<br>C 0.57822 -1.46549 -0.69967<br>C 1.43085 -0.95033 0.21351<br>C 0.94416 -0.11281 1.30518<br>C -0.36326 0.21821 1.45052<br>H 0.93212 -2.06292 -1.52958<br>H 1.67916 0.28867 1.99118<br>H -0.71601 0.80219 2.28924<br>O -2.55889 -0.44558 0.86545<br>O -1.66532 -1.69178 -1.39292<br>C 2.90661 -1.16007 0.13496<br>H 3.17701 -1.78641 -0.71088<br>H 3.40828 -0.19391 0.04404<br>H 3.26635 -1.62024 1.05696<br>S -0.56647 2.06346 -0.35260<br>C 1.16515 2.17376 -0.82961<br>H 1.21399 2.97296 -1.57243<br>H 1.78472 2.45824 0.01679<br>H 1.51677 1.25218 -1.28642<br><br>ν_{imagin} = 289.6*i* cm^{-1}	C_1	-858.77950 (1.1)	-858.61575 (1.0)	-858.66730 (1.0)	-857.78009 (2.4)	-858.75931 (2.5)	-857.68808 (1.0)

Part 16: Methanethiol, optimizations at the M06-2X / 6-311++G(2d,2p), SMD level, single-point energies at the DLPNO-CCSD(T)-F12 / cc-pVTZ-F12 level

Table S16.1. One-electron oxidized neutral form in water.

Structure	Schematic drawing	Symmetry	$G_{SMD,M06\text{-}2X,large}$	$H_{SMD,RRHO,M06\text{-}2X,large}$	$G_{SMD,RRHO,M06\text{-}2X,large}$	$E_{vacuo,DLPNO\text{-}CCSD(T)\text{-}F12/cc\text{-}pVTZ\text{-}F12}$	$E_{vacuo,M06\text{-}2X,large}$	$G_{SMD,RRHO,DLPNO\text{-}CCSD(T)\text{-}F12/cc\text{-}pVTZ\text{-}F12}$
conf1	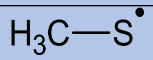<br>methylsulfanyl<br><br>S -0.00447 -0.68954 0.00000<br>C -0.00447 1.10435 0.00000<br>H 1.04560 1.40931 0.00000<br>H -0.47363 1.49859 0.89691<br>H -0.47363 1.49859 -0.89691	C_s	-438.04163 (0.0)	-438.00019 (0.0)	-438.02837 (0.0)	-437.56217 (0.0)	-438.03934 (0.0)	-437.55121 (0.0)

Part 17: 4-Methylcatechol, optimizations at the M06-2X / 6-311++G(2d,2p), SMD level, single-point energies at the DLPNO-CCSD(T)-F12 / cc-pVTZ-F12 level

Table S17.1. Two-electron oxidized neutral form in water.

Structure	Schematic drawing	Symmetry	$G_{SMD,M06-2X,large}$	$H_{SMD,RRHO,M06-2X,large}$	$G_{SMD,RRHO,M06-2X,large}$	$E_{vacuo,DLPNO-CCSD(T)-F12/cc-pVTZ-F12}$	$E_{vacuo,M06-2X,large}$	$G_{SMD,RRHO,DLPNO-CCSD(T)-F12/cc-pVTZ-F12}$
quinone, conf1	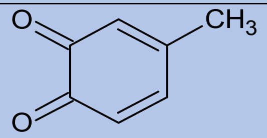<br>4-methylcyclohexa-3,5-diene-1,2-dione<br><br>C 1.35917 0.47118 0.00000<br>C 0.00000 1.22114 0.00000<br>C -1.21568 0.42586 0.00000<br>C -1.15250 -0.91679 0.00000<br>C 0.14726 -1.62023 0.00000<br>C 1.32586 -0.99123 0.00000<br>H -2.15935 0.95474 0.00000<br>H 0.10978 -2.70256 0.00000<br>H 2.26986 -1.51786 0.00000<br>O 2.38162 1.12008 0.00000<br>O 0.01789 2.43535 0.00000<br>C -2.36773 -1.77846 0.00000<br>H -2.35792 -2.43017 0.87625<br>H -3.27881 -1.18620 0.00000<br>H -2.35792 -2.43017 -0.87625	C_s	-420.73193 (0.0)	-420.60977 (0.0)	-420.65133 (0.0)	-420.21633 (0.0)	-420.71665 (0.0)	-420.15101 (0.0)

Part 18: Reference conventional coupled-cluster calculations in vacuo (at the PBE0 / 6-31+G(d,p) geometries)

Table S18.1.

Species	Structure	Schematic drawing	Symmetry	$E_{\mathrm{(RO)CCSD(T)/cc\text{-}pVTZ}}$	$G_{\mathrm{RRHO,(RO)CCSD(T)/cc\text{-}pVTZ}}$	$E_{\mathrm{(RO)CCSD(T)/jun\text{-}cc\text{-}pVTZ}}$	$G_{\mathrm{RRHO,(RO)CCSD(T)/jun\text{-}cc\text{-}pVTZ}}$
Methanethiol, reduced neutral form	conf1	H_3C—SH<br>methanethiol	C_s	-438.16889	-438.14651	-438.17169	-438.14931
Methanethiol, one-electron oxidized neutral form	conf2	H_3C—$S^{\bullet}$<br>methylsulfanyl	C_s	-437.52579	-437.51417	-437.52831	-437.51669
4-Methylcatechol, two-electron oxidized neutral form	quinone, conf1	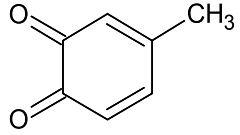<br>4-methylcyclohexa-3,5-diene-1,2-dione	C_s	-420.06249	-419.98193	-420.07534	-419.99478
4-Methylcatechol – methanethiol adduct, reduced neutral form	6-adduct, conf1	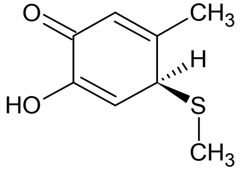<br>(4*S*)-2-hydroxy-5-methyl-4-(methylsulfanyl)cyclohexa-2,5-dien-1-one	C_1	-858.27207	-858.14336	-858.28902	-858.16031
	5-adduct, conf2	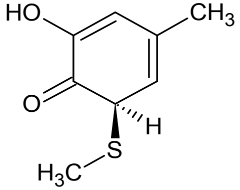<br>(6*R*)-2-hydroxy-4-methyl-6-(methylsulfanyl)cyclohexa-2,4-dien-1-one	C_1	-858.26634	-858.13739	-858.28294	-858.15399

4-Methylcatechol – methanethiol adduct, one-electron oxidized neutral form	6-adduct, conf1	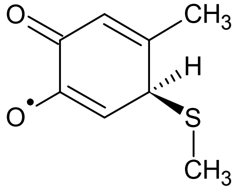 [(3*S*)-4-methyl-3-(methylsulfanyl)-6-oxocyclohexa-1,4-dien-1-yl]oxidanyl	C_1	-857.61181	-857.49786	-857.62855	-857.51460
	5-adduct, conf3	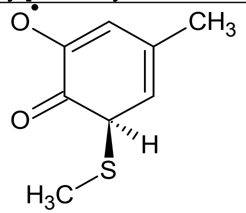 [(5*R*)-3-methyl-5-(methylsulfanyl)-6-oxocyclohexa-1,3-dien-1-yl]oxidanyl	C_1	-857.62133	-857.50778	-857.63797	-857.52442

Part 19: Transition structures for the process of H-atom abstraction form methanethiol by the methanethiyl - 4-methyl-*o*-benzoquinone 5-*S*-adduct (PBE0 / 6-31+G(d,p), PCM geometries)

Table S19.1.

Structure	Schematic drawing	Symmetry	G_{PCM}	$H_{PCM,RRHO}$	$G_{PCM,RRHO}$	$G_{SMD,M06-2X,large}$	$G_{SMD,RRHO,M06-2X,large}$
6-adduct, conf1	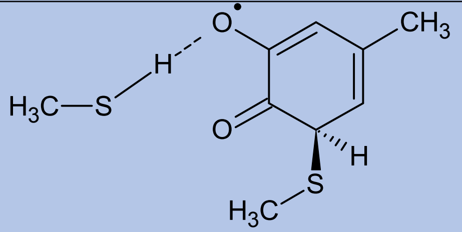<br>C 0.28933 0.25079 0.99657<br>C 0.04204 -1.10719 0.42102<br>C 1.10973 -1.82564 -0.09865<br>C 2.44613 -1.31926 -0.12069<br>C 2.68164 -0.01944 0.23840<br>C 1.58271 0.90536 0.60152<br>H 0.91456 -2.83514 -0.45160<br>H 3.68652 0.39090 0.20390<br>H 1.88726 1.65184 1.34084<br>O -0.55256 0.80270 1.68671<br>O -1.13815 -1.62390 0.52355<br>C 3.55184 -2.23485 -0.55028<br>H 3.61153 -3.10465 0.11251<br>H 3.36554 -2.61199 -1.56158<br>H 4.51741 -1.72576 -0.54281<br>S 1.12782 1.81654 -0.95546<br>C 0.19698 3.20866 -0.27857<br>H -0.14200 3.78758 -1.14112<br>H -0.67190 2.86295 0.28633<br>H 0.83454 3.83510 0.34919<br>H -2.00938 -0.89949 0.50794<br>S -3.37199 0.01165 0.08816<br>C -4.16065 -1.16472 -1.04801<br>H -4.25752 -2.15522 -0.59770<br>H -5.15654 -0.79239 -1.30369<br>H -3.58607 -1.24724 -1.97521	C_1	-1296.69240 (0.0)	-1296.47815 (0.0)	-1296.54207 (0.0)	-1297.46947 (0.0)	-1297.31914 (0.0)

	ν_{imagin} = 2117.2*i* cm^{-1}						

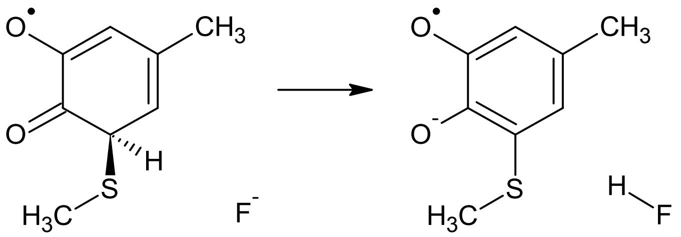
O•
CH3
O
H
S
H3C
F-
O•
CH3
O-
S
H3C
H
F

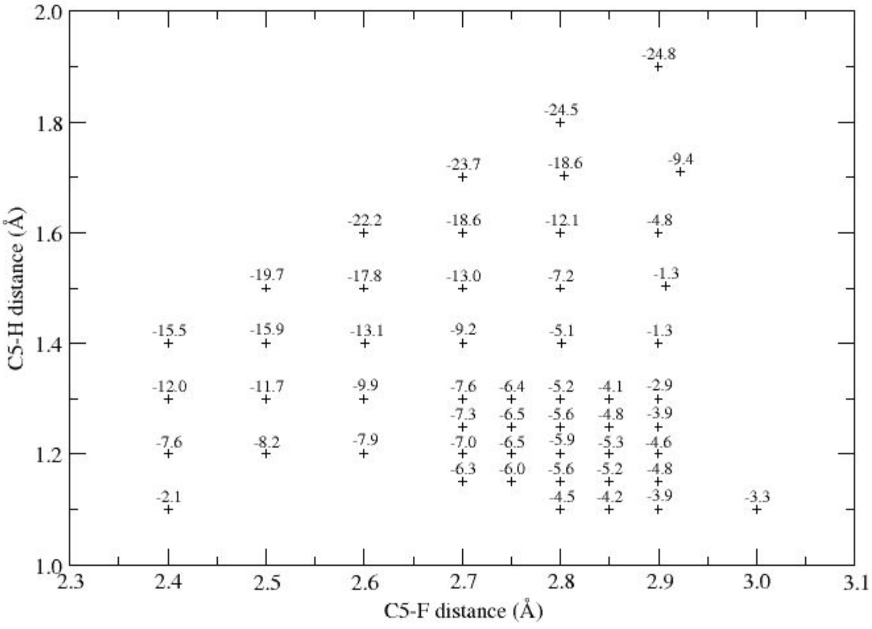
C5-H distance (Å)
C5-F distance (Å)
2.0
1.8
1.6
1.4
1.2
1.0
2.3
2.4
2.5
2.6
2.7
2.8
2.9
3.0
3.1
-24.8
-24.5
-23.7 -18.6 -9.4
-22.2 -18.6 -12.1 -4.8
-19.7 -17.8 -13.0 -7.2 -1.3
-15.5 -15.9 -13.1 -9.2 -5.1 -1.3
-12.0 -11.7 -9.9 -7.6 -6.4 -5.2 -4.1 -2.9
-7.3 -6.5 -5.6 -4.8 -3.9
-7.6 -8.2 -7.9 -7.0 -6.5 -5.9 -5.3 -4.6
-6.3 -6.0 -5.6 -5.2 -4.8
-2.1 -4.5 -4.2 -3.9 -3.3

A

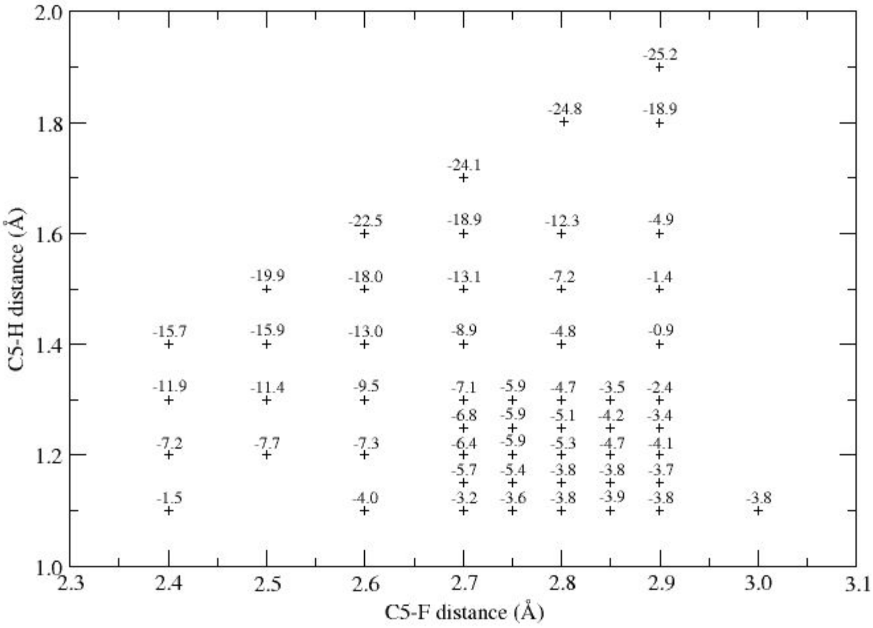
C5-H distance (Å)
C5-F distance (Å)
2.0
1.8
1.6
1.4
1.2
1.0
2.3
2.4
2.5
2.6
2.7
2.8
2.9
3.0
3.1
-25.2
-24.8 -18.9
-24.1
-22.5 -18.9 -12.3 -4.9
-19.9 -18.0 -13.1 -7.2 -1.4
-15.7 -15.9 -13.0 -8.9 -4.8 -0.9
-11.9 -11.4 -9.5 -7.1 -5.9 -4.7 -3.5 -2.4
-6.8 -5.9 -5.1 -4.2 -3.4
-7.2 -7.7 -7.3 -6.4 -5.9 -5.3 -4.7 -4.1
-5.7 -5.4 -3.8 -3.8 -3.7
-1.5 -4.0 -3.2 -3.6 -3.8 -3.9 -3.8 -3.8

B

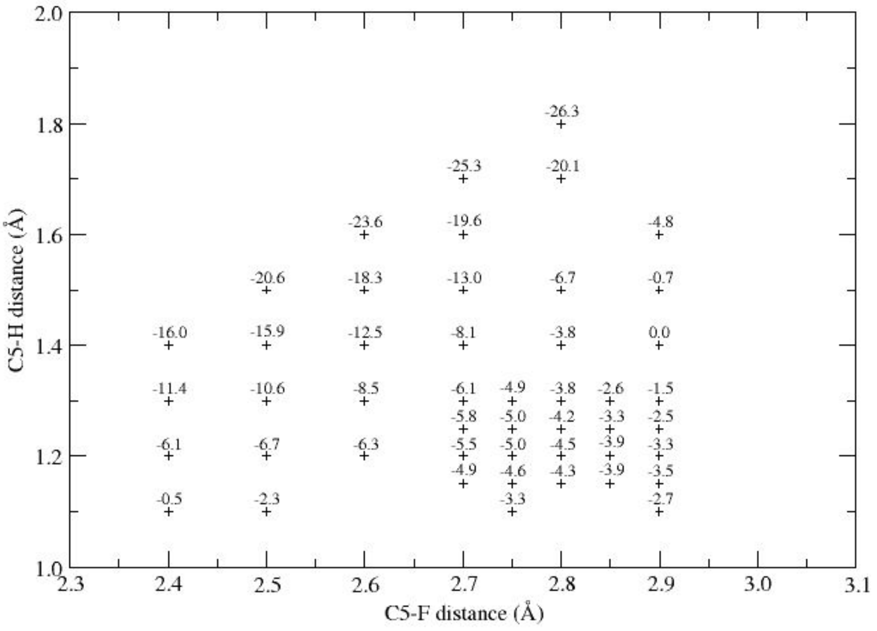

C

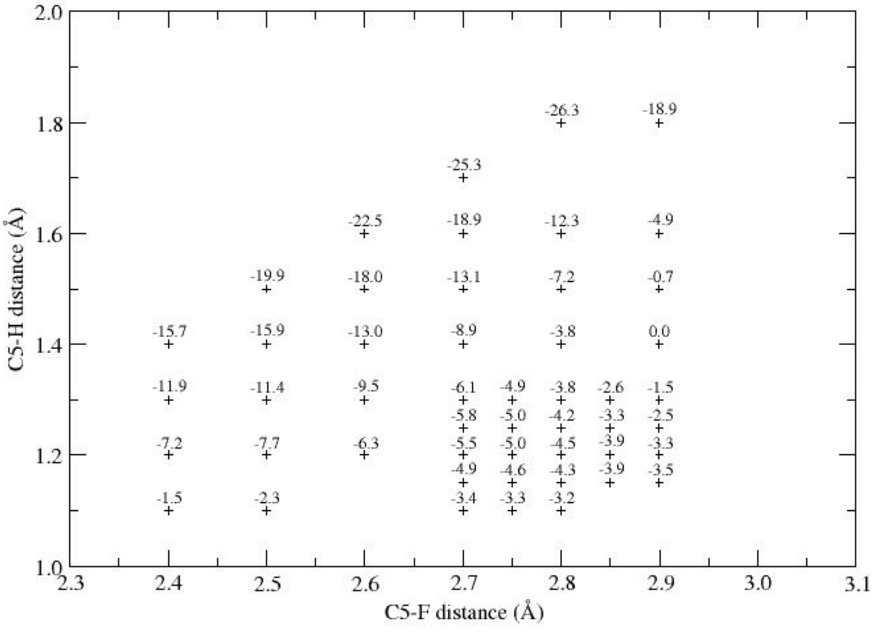

D

Figure S26. Relaxed potential energy scans for the deprotonation of the methanethiyl - 4-methyl-*o*-benzoquinone 5-*S*-adduct by a fluoride ion, performed at the PBE0 / 6-31+G(d,p) level in water (PCM). Panel A: conf1; panel B: conf2; panel C: conf3; panel D: conf4. Structures and nomenclature of the starting conformers as in Table S5.8. All energies (kcal mol^{-1}) are referred to the sum of the energies (G_{PCM}) of the reactants (5-adduct, conf2: -858.21399 Ha; fluoride: -99.89220 Ha).

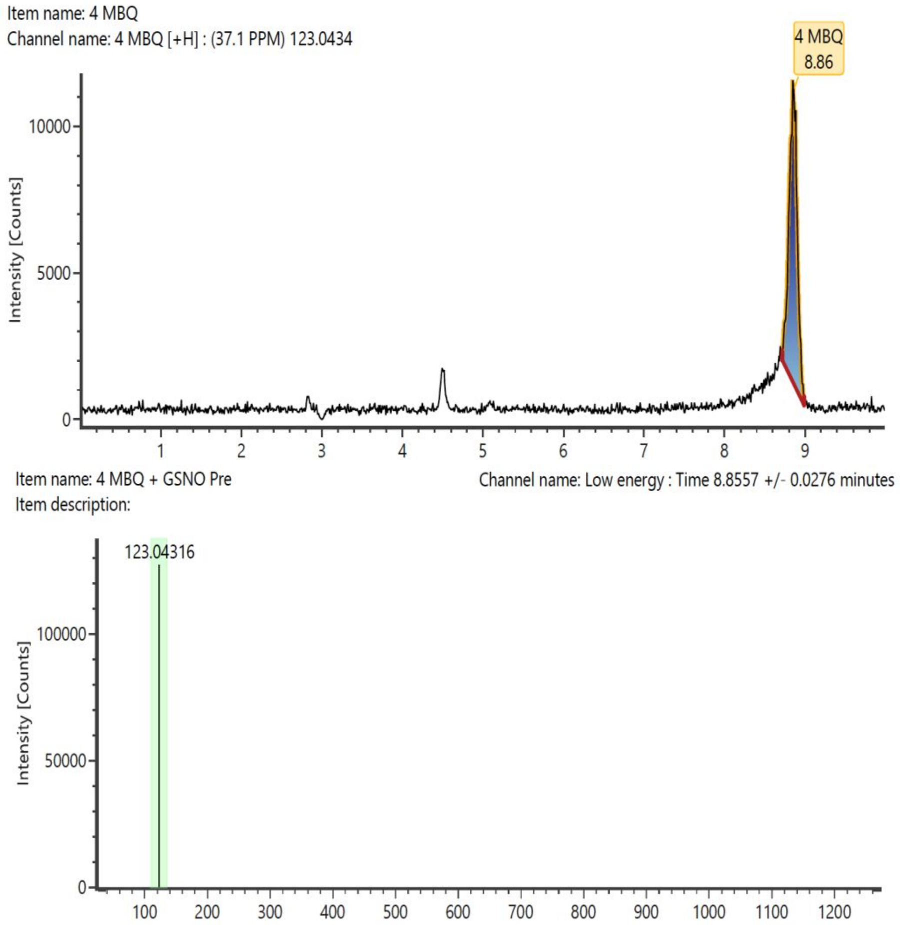

Figure S27. (Top) UPLC-Q-TOF chromatogram of 4-MBQ, in acetate buffer/MeCN (pH 5.0), before reaction with GS-NO, (bottom) corresponding MS spectrum showing only the $[M+H]^+$ ion at *m/z* 123.

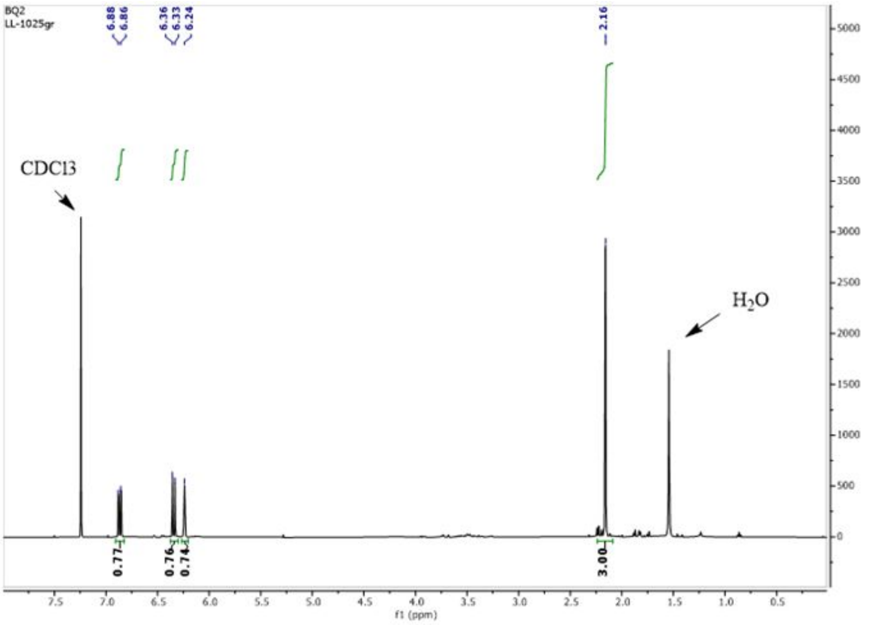

Figure S28. ^{1}H-NMR spectrum of 4-MBQ (400 MHz, $CDCl_3$).

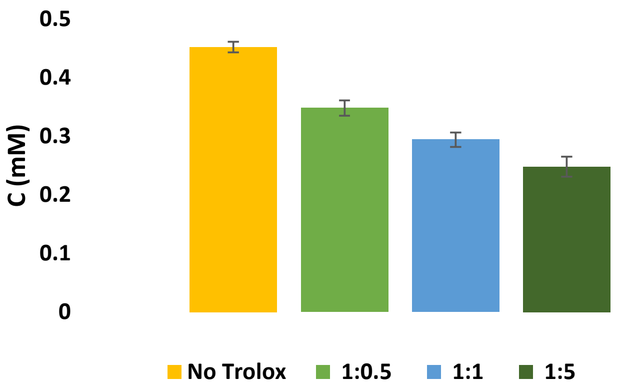

Figure S29. Formation yields of the 5-*S*-adduct at pH 4.0 in air with or without Trolox (0.5-5 mM) at different molar ratios with respect to 4-MBQ-cysteine (1 mM).

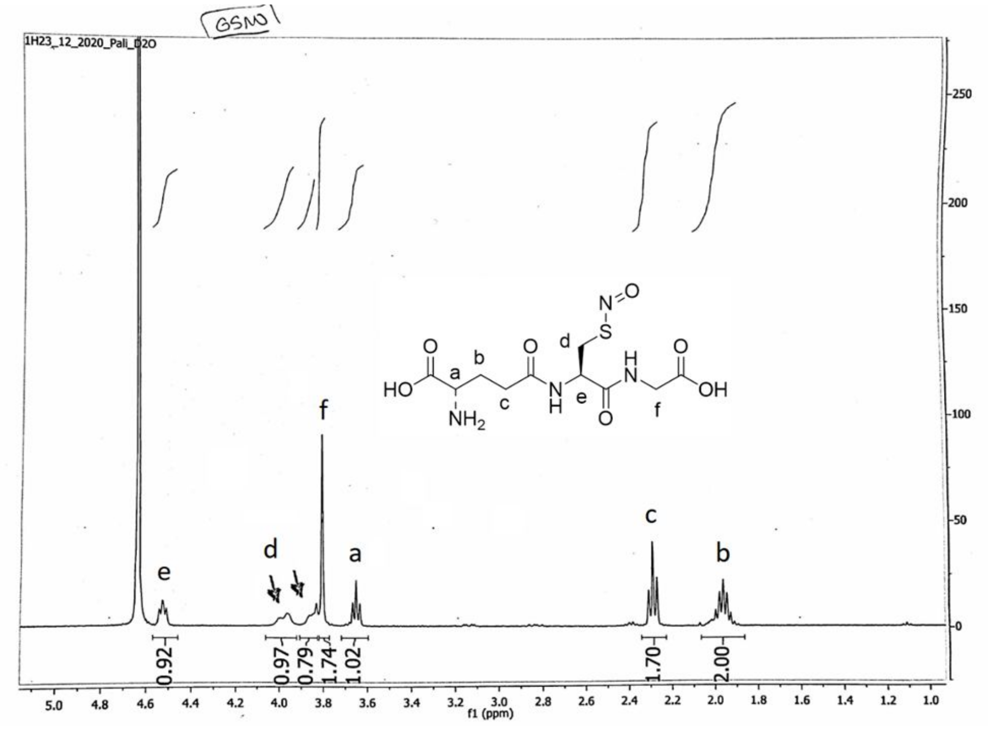

Figure S30. ^{1}H-NMR spectrum of GS-NO in D_2O at 400 MHz.

References

[1] S. Ito, S. Inoue, Y. Yamamoto, K. Fujita, *J. Med. Chem.* **1981**, *24*, 673-677.

[2] E. Adler, R. Magnusson, *Acta Chem. Scand.* **1969**,*13*, 505-519.

[3] A. Napolitano, S. Memoli, O. Crescenzi, G. Prota, *J. Org. Chem.* **1996**, *61*, 598-604.

[4] M. S. Cabeza, S. A. Guerrero, A. A. Iglesias, D. G. Arias, *Biochim. Biophys. Acta* **2015**, *1850*, 1233-1244.

[5] T. W. Hart, Tetrahedron Lett. **1985**, *26*, 2013-2016.

[6] W. M. Jones, J. B. Tapia, R. R. Tuttle, M. M. Reynolds, *Langmuir* **2020**, *36*, 3903-3911.

[7] a) A. J. McGrath, G. E. Garrett, L. Valgimigli, D. A. Pratt, *J. Am. Chem. Soc.* **2010**, *132*, 16759-16761. b) R. Amorati, G. F. Pedulli, D. A. Pratt, L. Valgimigli, *Chem. Commun.* **2010**, *46*, 5139-5141.

[8] L. Valgimigli, K. U. Ingold, J. Lusztyk, *J. Org. Chem.* **1996**, *61*, 7947-795024.

[9] M. J. Frisch, G. W. Trucks, H. B. Schlegel, G. E. Scuseria, M. A. Robb, J. R. Cheeseman, G. Scalmani, V. Barone, B. Mennucci, G. A. Petersson, H. Nakatsuji, M. Caricato, X. Li, H. P. Hratchian, A. F. Izmaylov, J. Bloino, G. Zheng, J. L. Sonnenberg, M. Hada, M. Ehara, K. Toyota, R. Fukuda, J. Hasegawa, M. Ishida, T. Nakajima, Y. Honda, O. Kitao, H. Nakai, T. Vreven, J. A. Montgomery Jr., J. E. Peralta, F. Ogliaro, M. Bearpark, J. J. Heyd, E. Brothers, K. N. Kudin, V. N. Staroverov, T. Keith, R. Kobayashi, J. Normand, K. Raghavachari, A. Rendell, J. C. Burant, S. S. Iyengar, J. Tomasi, M. Cossi, N. Rega, J. M. Millam, M. Klene, J. E. Knox, J. B. Cross, V. Bakken, C. Adamo, J. Jaramillo, R. Gomperts, R. E. Stratmann, O. Yazyev, A. J. Austin, R. Cammi, C. Pomelli, J. W. Ochterski, R. L. Martin, K. Morokuma, V. G. Zakrzewski, G. A. Voth, P. Salvador, J. J. Dannenberg, S. Dapprich, A. D. Daniels, O. Farkas, J. B. Foresman, J. V. Ortiz, J. Cioslowski, D. J. Fox, *Gaussian 09*, Revision D.01; Gaussian, Inc., Wallingford CT, **2013**.

[10] a) Neese, F. *WIREs Comput. Mol. Sci.* **2012**, *2*, 73-78; b) Neese, F. *WIREs Comput. Mol. Sci.* **2017**, *8*, e1327.

[11] C. Adamo, V. Barone, *J. Chem. Phys.* **1999**, *110*, 6158-6169.

[12] J.-D. Chai, M. Head-Gordon, *Phys. Chem. Chem. Phys.* **2008**, *10*, 6615-6620.

[13] J. M. Smith, Y. J. Alahmadi, C. N. Rowley, *J. Chem. Theory Comput.* **2013**, *9*, 4860-4865.

[14] Y. Zhao, D. G. Truhlar, *Theor. Chem. Acc.* **2008**, *120*, 215-241.

[15] a) J. Cížek, in *Advances in Chemical Physics, Vol. 14* (Ed.: P. C. Hariharan), Wiley Interscience, New York, **1969**, pp. 35; b) G. D. Purvis III, R. J. Bartlett, *J. Chem. Phys.* **1982**, *76*, 1910-1918; c) G. E. Scuseria, C. L. Janssen, H. F. Schaefer III, *J. Chem. Phys.* **1988**, *89*, 7382-7387, d) G. E. Scuseria, H. F. Schaefer III, *J. Chem. Phys.* **1989**, *90*, 3700-3703.

[16] J. A. Pople, M. Head-Gordon, K. Raghavachari, *J. Chem. Phys.* **1987**, *87*, 5968-5975.

[17] a) T. H. Dunning Jr., *J. Chem. Phys.* **1989**, *90*, 1007; b) D. E. Woon, T. H. Dunning Jr., *J. Chem. Phys.* **1993**, *98*, 1358; c) E. R. Davidson, *Chem. Phys. Lett.* **1996**, *260*, 514-518.

[18] E. Papajak, J. Zheng, X. Xu, H. R. Leverentz, D. G. Truhlar, *J. Chem. Theory Comput.* **2011**, *7*, 3027-3034.

[19] a) C. Riplinger, P. Pinski, U. Becker, E. F. Valeev, F. Neese, *J. Chem. Phys.* **2016**, *144*, 024109; b) Saitow, M.; Becker, U.; Riplinger, C.; Valeev, E. F.; Neese, F. *J. Chem. Phys.* **2017**, *146*, 164105.

[20] a) W. Klopper, F. R. Manby, S. Ten-No, E. F. Valeev, *Int. Rev. Phys. Chem.* **2006**, *25*, 427-468; b) Knizia, G.; Adler, T. B.; Werner, H.-J. *J. Chem. Phys.* **2009**, *130*, 054104; c) F. Pavosevic, C. Peng, P. Pinski, C. Riplinger, F. Neese, E. F. Valeev, *J. Chem. Phys.* **2017**, *146*, 174108.

[21] [K. A. Peterson, T. B. Adler, H-J. Werner, *J. Chem. Phys.* **2008**, *128*, 084102.

[22] a) S. Miertus, E. Scrocco, J. Tomasi, *J. Chem. Phys.* **1981**, *55*, 117-129; b) M. Cossi, G. Scalmani, N. Rega, V. Barone, *J. Chem. Phys.* **2002**, *117*, 43-54; c) G. Scalmani, V. Barone, K. N. Kudin, C. S. Pomelli, G. E. Scuseria, M. J. Frisch, *Theor. Chem. Acc.* **2004**, *111*, 90-100; d) J. Tomasi, B. Mennucci, R. Cammi, *Chem. Rev.* **2005**, 105, 2999-3093.

[23] a) A. K. Rappé, C. J. Casewit, K. S. Colwell, W. A. Goddard III, W. M. Skiff, *J. Am. Chem. Soc.* **1992**, *114*, 10024-10035; b) D. A. York, M. Karplus, *J. Phys. Chem. A* **1999**, *103*, 11060-11079; c) G. Scalmani, M. J. Frisch, *J. Chem. Phys.* **2010**, *132*, 1-15.

[24] A. V. Marenich, C. J. Cramer, D. G. Truhlar, *J. Phys. Chem. B* **2009**, *113*, 6378-6396.

[25] C. J. Cramer, in *Essentials of Computational Chemistry: Theories and Models*, *2nd edition*, John Wiley & Sons, Chichester, **2004**, pp. 378-379.
